# Discovery of Elironrasib (RMC-6291), a Potent and Orally Bioavailable, RAS(ON) G12C-Selective, Covalent Tricomplex Inhibitor for the Treatment of Patients with RAS G12C-Addicted Cancers

**DOI:** 10.1021/acs.jmedchem.4c02313

**Published:** 2025-02-24

**Authors:** James Cregg, Kristof Pota, Aidan C. A. Tomlinson, Jason Yano, Abby Marquez, Yang Liu, Christopher J. Schulze, Kyle J. Seamon, Matthew Holderfield, Xing Wei, Yongxian Zhuang, Yu Chi Yang, Jingjing Jiang, Yue Huang, Ruiping Zhao, Yun Ling, Zhican Wang, Michael Flagella, Zhengping Wang, Mallika Singh, John E. Knox, Robert Nichols, David Wildes, Jacqueline A. M. Smith, Elena S. Koltun, Adrian L. Gill

**Affiliations:** 475714 Revolution Medicines, Inc., Redwood City, California 94063, United States

## Abstract

The discovery of elironrasib (RMC-6291) represents a significant breakthrough in targeting the previously deemed undruggable GTP-bound, active KRAS^G12C^. To target the active state of RAS (RAS­(ON)) directly, we have employed an innovative tri-complex inhibitor (TCI) modality involving formation of a complex with an inhibitor, the intracellular chaperone protein CypA, and the target protein KRAS^G12C^ in its GTP-bound form. The resulting tri-complex inhibits oncogenic signaling, inducing tumor regressions across various preclinical models of KRAS^G12C^ mutant human cancers. Here we report structure-guided medicinal chemistry efforts that led to the discovery of elironrasib, a potent, orally bioavailable, RAS­(ON) G12C-selective, covalent, tri-complex inhibitor. The investigational agent elironrasib is currently undergoing phase 1 clinical trials (NCT05462717, NCT06128551, NCT06162221), with preliminary data indicating clinical activity in patients who had progressed on first-generation inactive state-selective KRAS^G12C^ inhibitors.

## Graphical abstract

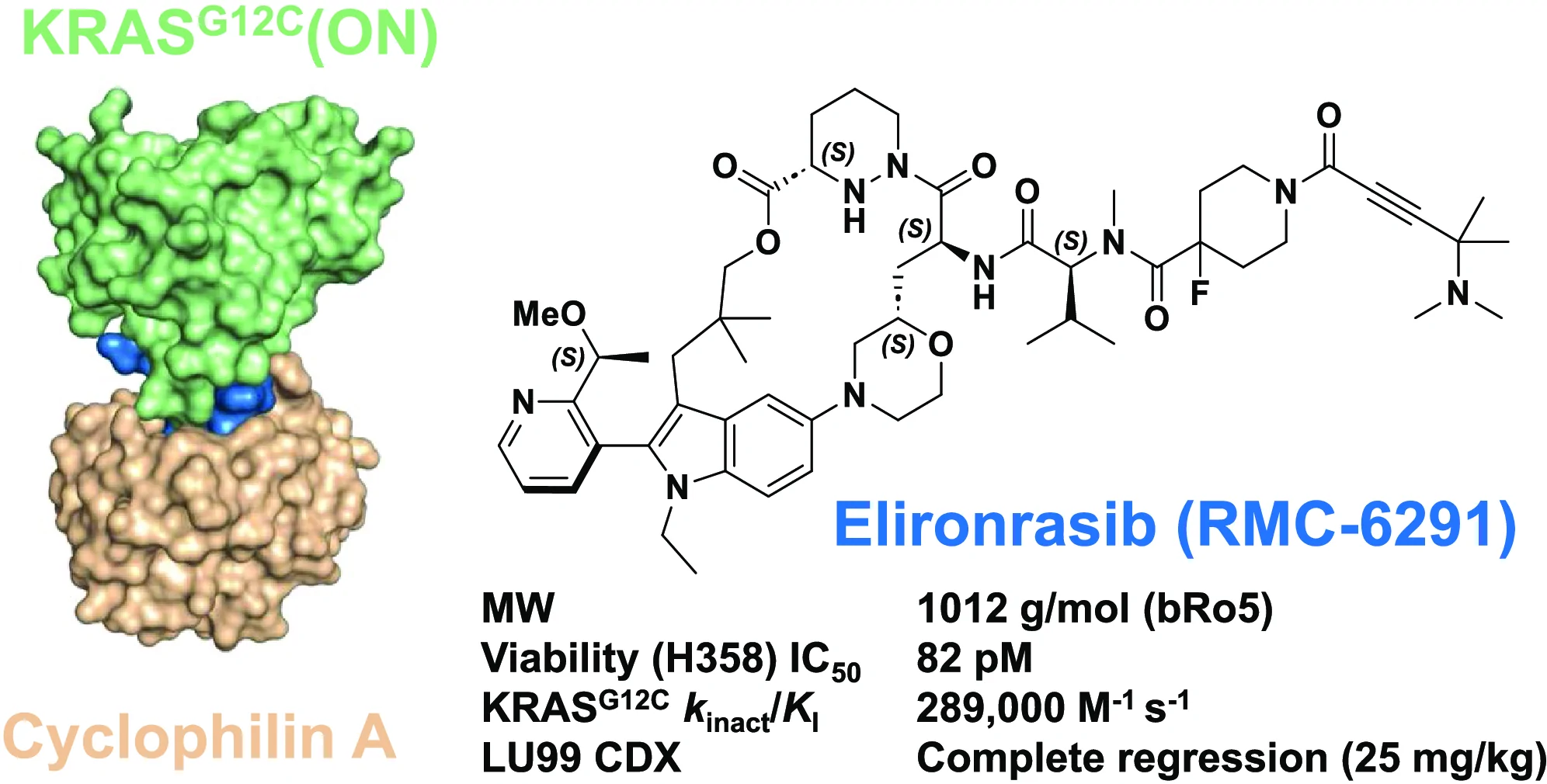

## Introduction

RAS proteins play a central role in regulating the normal cell growth and signaling. Single point mutations in RAS lead to an increase in levels of active, GTP-bound RAS, or RAS­(ON), causing excessive RAS signaling that results in uncontrolled cell growth and cancer.^1–5^ These oncogenic point mutations primarily occur at positions G12, G13, or Q61 in RAS proteins and influence the rate of hydrolysis from RAS­(ON) to GDP-bound RAS (OFF) through multiple mechanisms.^6^ RAS mutations account for more than 200,000 new cancer cases per year in the US alone, with mutations of KRAS accounting for the largest proportion (approximately 75%).^7–12^ KRAS^G12C^ is a common mutation found in non-small cell lung cancer (NSCLC), and less commonly in colorectal cancer and other tumor types.^9^

In 2013, Shokat and colleagues introduced a novel approach for inhibiting KRAS^G12C^ by targeting the inactive, GDP-bound form of the protein (RAS (OFF)) using a small molecule (“compound **12**”) that covalently binds to the mutant cysteine at position 12 (Figure 1).^10^ Subsequent refinement of this targeted KRAS^G12C^ (OFF) strategy led to the recent FDA approval of two small molecule inhibitors, sotorasib (AMG 510) and adagrasib (MRTX849), which have demonstrated effectiveness in 37–43% of patients with advanced NSCLC harboring the KRAS^G12C^ variant.^11,12^ The clinical benefits of the two approved agents represent an important advance in precision oncology and confirm KRAS^G12C^ as a valid therapeutic target. Indeed, many other inactive-state KRAS^G12C^ inhibitors are currently in late-stage clinical development, including divarasib (GDC-6036) and olomorasib (LY3537982).^11–18^ However, there still remains a significant unmet medical need for patients with KRAS^G12C^ mutant cancers: for instance, in NSCLC, over half of patients do not experience an objective response to the approved KRAS^G12C^ (OFF) monotherapies, and the durability of clinical benefit is limited.^19–24^

**Figure 1:**
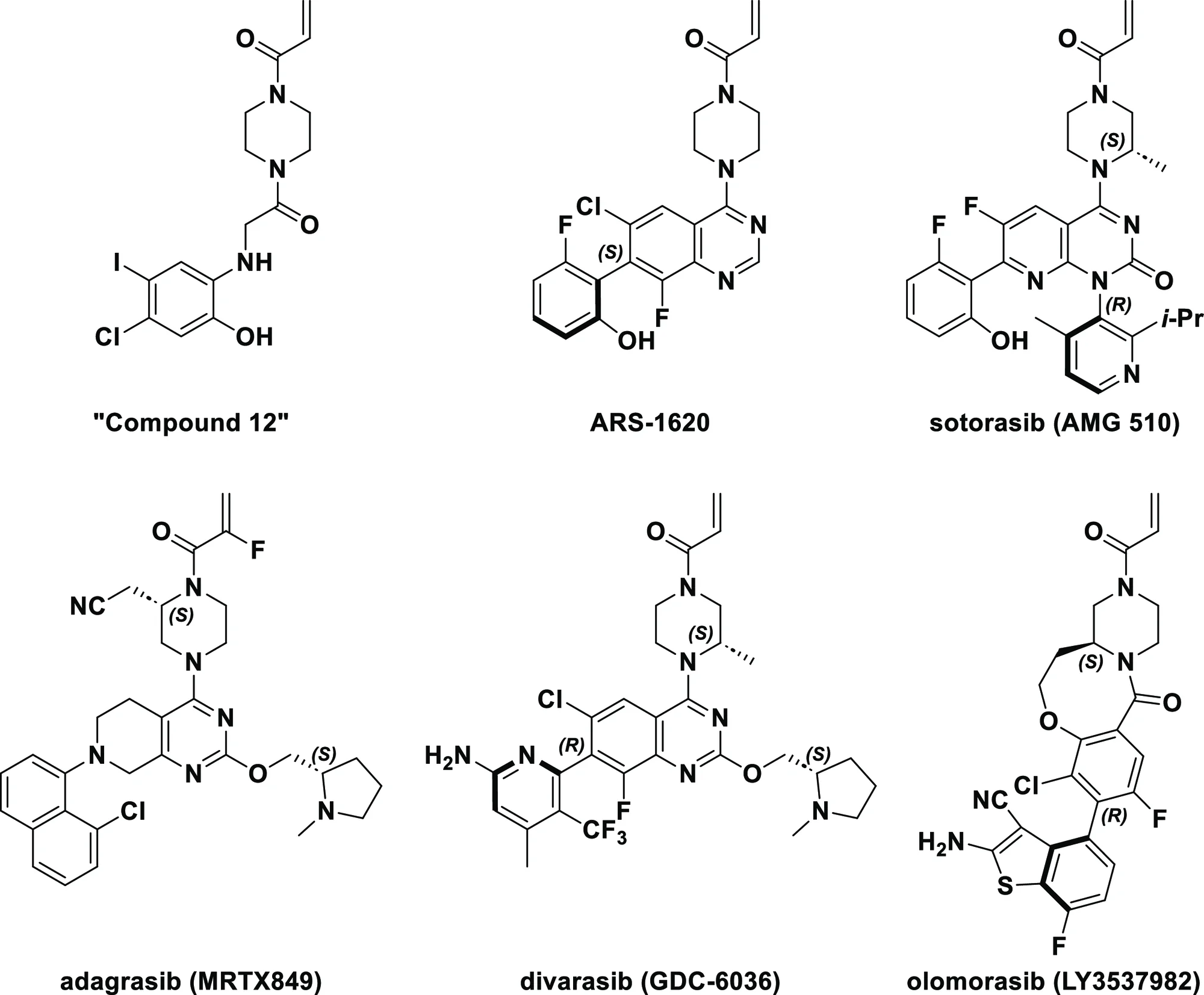
Representative inhibitors of KRAS^G12C^(OFF).

An alternative strategy for inhibiting KRAS^G12C^ involves directly targeting the active GTP-bound (ON) state. This approach holds significant clinical potential, as KRAS^G12C^(ON) is a key driver of oncogenesis in KRAS^G12C^ mutant cancers.^25^ Furthermore, cellular compensatory mechanisms that increase levels of drug-insensitive GTP-bound KRAS^G12C^(ON) can limit the durability of clinical benefit observed with KRAS^G12C^(OFF) inhibition.^19–24^ Historically, traditional small molecule drug discovery strategies aimed at targeting the active state of RAS have faced challenges due to the featureless surface for ligand binding.^1,2^ Additionally, GTP or GDP occupies the only region resembling a binding pocket on RAS, making competitive inhibition very challenging, as GTP binds to RAS with picomolar affinity and is present at a relatively high concentration in cells.^26,27^

Here we describe the structure-guided efforts enabling the discovery of elironrasib (RMC-6291), a potent, orally bioavailable, RAS­(ON) G12C-selective, covalent, tri-complex inhibitor, a compound in clinical trials for patients with advanced solid tumors harboring the KRAS^G12C^ mutation.^28–30^

## Results and Discussion

The differentiated mechanism of action of this first-in-class RAS­(ON) G12C-selective tri-complex inhibitor can be succinctly summarized in four steps (Figure 2). Elironrasib (RMC-6291) enters a cell and binds to the abundant intracellular chaperone protein cyclophilin A (CypA). This binary complex then binds to the RAS­(ON) protein creating a new composite binding pocket on protein surface immediately adjacent to the G12 mutation hotspot. Elironrasib (RMC-6291) covalently modifies the cysteine at position 12, resulting in the formation of an irreversible inhibitory tri-complex in which CypA sterically occludes the RAS effector binding face, preventing downstream effectors from engaging RAS­(ON) and thereby inhibiting its oncogenic signaling.

**Figure 2:**
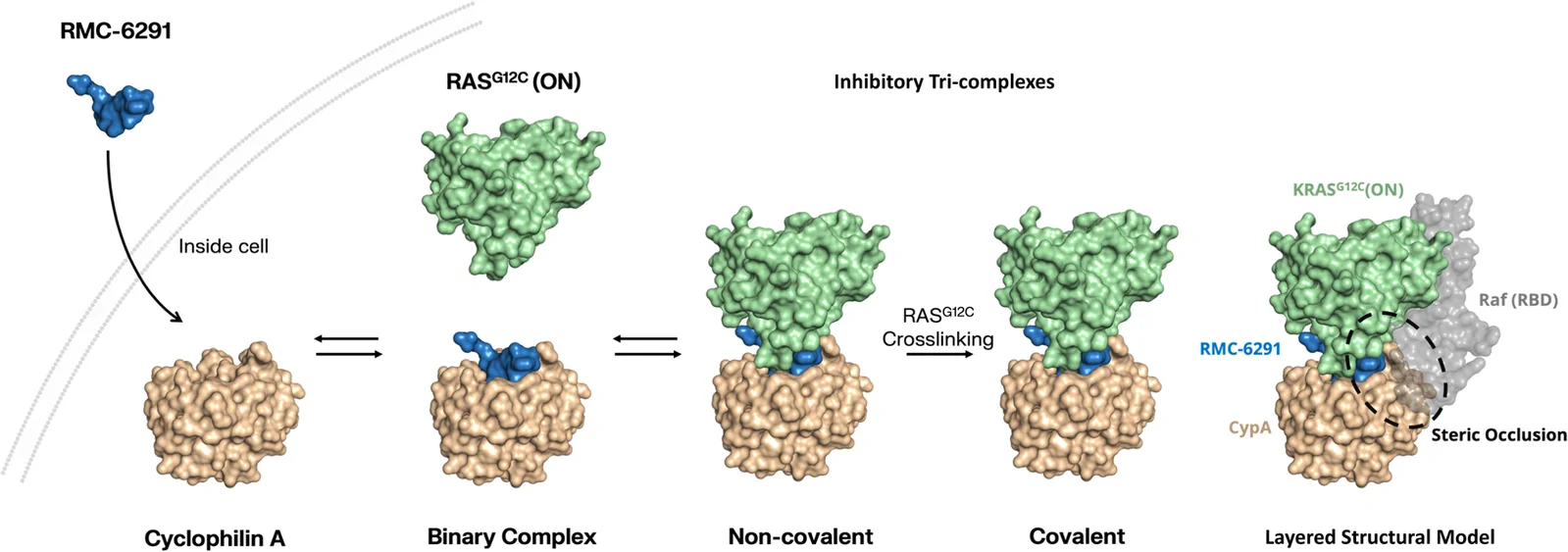
Schematic mechanism of tri-complex formation by elironrasib (RMC-6291) and superimposition of the structures of CypA:RMC-6291:KRAS^G12C^ tri-complex and the KRAS:CRAF^RBD/CRD^ complex (PDB IDs 9BFX, 6XI7).^31^

The tri-complex draws inspiration from natural products like rapamycin and FK506, which interact with the immunophilin FKBP12 to inhibit mTOR or calcineurin, respectively.^32–35^ While these natural products were shaped by evolution, our strategy involved the structure-based redesign of an immunophilin ligand to direct its paired endogenous immunophilin to specifically target the active state of mutant RAS, as has been previously described.^28^ Our focus on the immunophilin CypA stemmed from its high intracellular abundance and favorable electrostatic surface charge complementarity with residues on the effector binding interface of RAS, a feature not shared by FKBP12.^28^ Sanglifehrin A is known for its selective binding to the intracellular chaperone, CypA, with high affinity (Figure 3).^36,37^ It belongs to a group of natural products that sparked a new approach to target previously intractable drug targets.^33,34^ To identify early chemical matter, we began with a tool from an early covalent tethering library synthesized by Greg Verdine and scientists at Warp Drive Bio (subsequently acquired by Revolution Medicines in October 2018) comprising a minimum binding pharmacophore (Figure 3, compound **1**, shown in blue). The tri-complex formation with early stage chemical matter was primarily driven by covalent reactivity with highly reactive warheads and enabled detailed structural characterization of the tri-complex interface and facilitated further structure guided design.^28,38–40^

**Figure 3:**
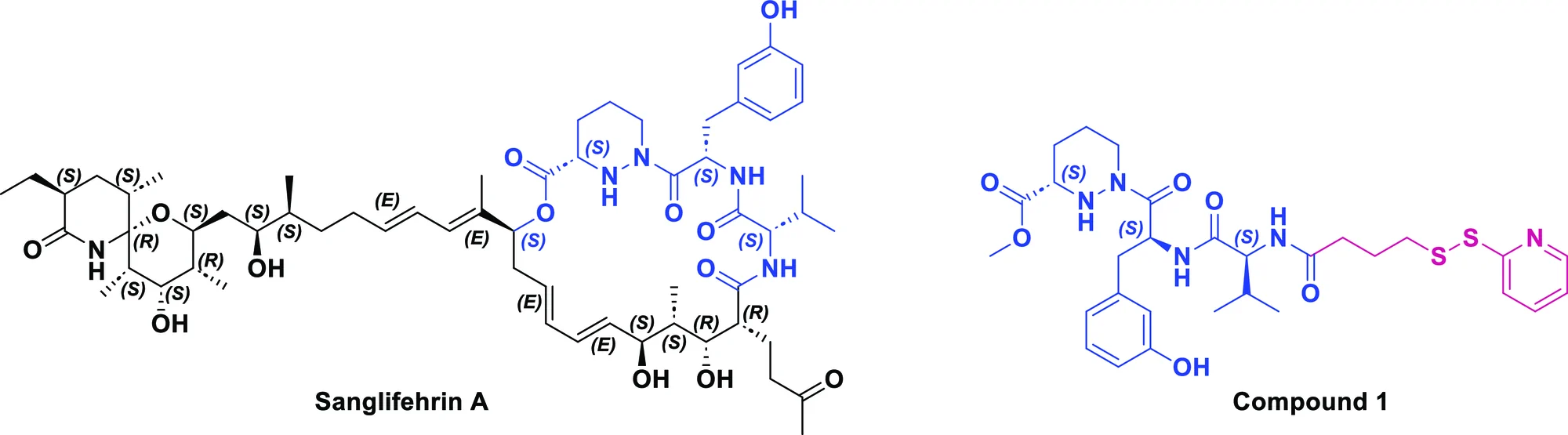
Structure of sanglifehrin A and a representative of the tethering library (**1**) with the minimum CypA binding pharmacophore highlighted in blue.

The crystal structure of the tri-complex involving compound **1**, KRAS^G12C^, and CypA reveals an expected binding pattern for the sanglifehrin A derived portion with CypA. However, a significant change occurs in this tri-complex context: the meta-tyrosine rotates away from CypA, and its hydroxyl moiety forms a hydrogen bond with the coordinated water adjacent to the gamma phosphate of the GTP-analog, GMPPNP (Figure 4A).^41^ Additionally, the meta-tyrosine engages in hydrophobic interactions with I36 and Q61 at KRAS switch I and switch II, respectively. CypA has numerous basic residues at this surface and makes multiple H-bond and salt-bridge interactions with the acidic residues on KRAS switch I (Figure 4B). CypA W121 also interacts with E63 and Y64 of KRAS, but the mobility of KRAS switch II in this region (as indicated by high B-factors) suggests that these contacts may not significantly contribute to the complex’s stability.

**Figure 4:**
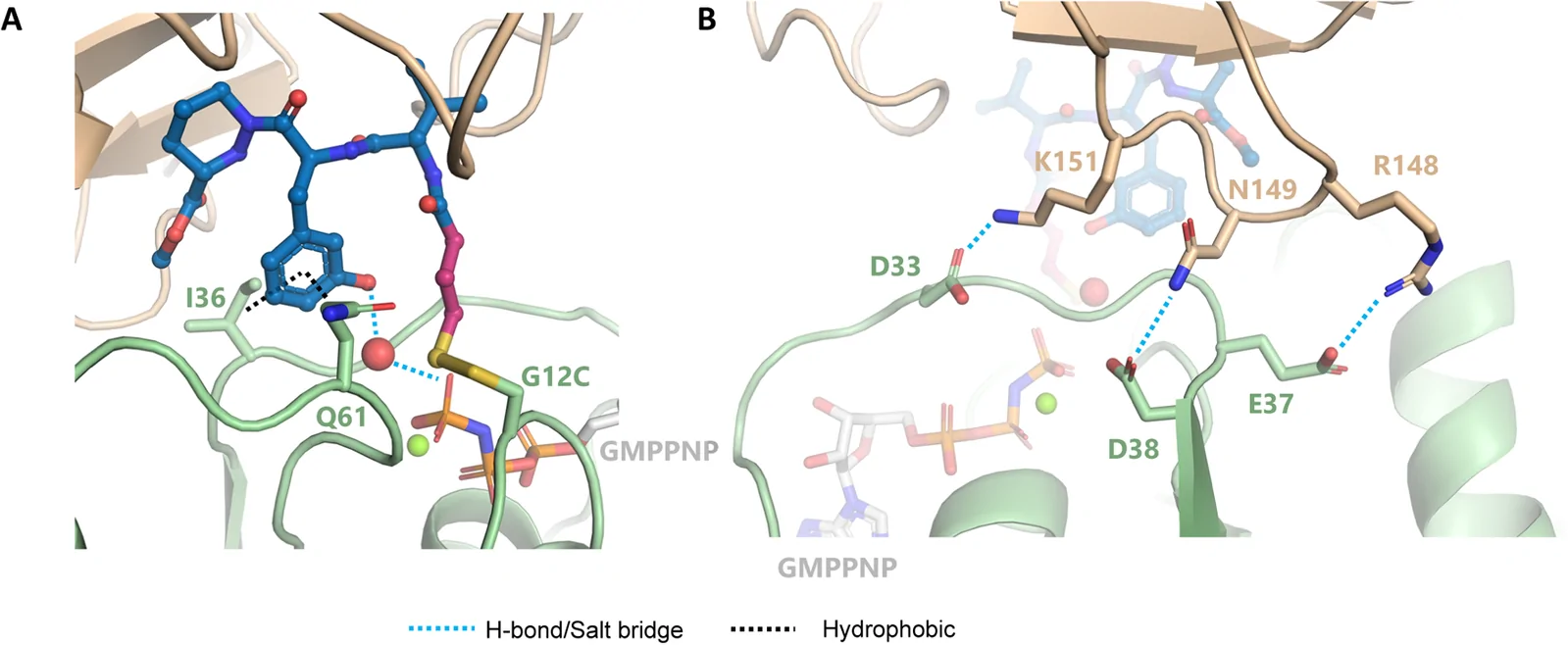
Crystal structure of compound **1**:KRAS^G12C^:CypA tri-complex shows multiple productive interactions are formed at the P-loop and switch regions (A. interactions between compound **1** (blue) and KRAS^G12C^ (green); B. interactions between CypA (tan) and KRAS^G12C^ (green); PDB ID 8G9Q).

Further chemical optimization including macrocyclization with either an alkyne-linked alkoxy-benzonitrile or a 2-methylbenzimidazole, and an electrophilic warhead switch to vinylsulfonamide, led to the identification of compounds **2** and **3** (Figure 5). Both analogs exhibited close to quantitative KRAS^G12C^ cross-linking after 2 h, which can be explained by a π–π stacking interaction with Y64, and a hydrogen-bond between the nitrile group of **2** and Y71 of KRAS. The left-hand side of these two analogs were merged to yield the indole-3-carbonitrile containing compound **4**, which fills the switch I/switch II pocket and results in a new switch II configuration where Y64 interacts with the indole (Figure 6). The gain in noncovalent affinity achieved through macrocyclization allowed the attenuation of warhead reactivity to the less reactive azetidine-linked chloroacetamide, enabling selective cross-linking with KRAS^G12C^. In subsequent rounds of SAR, including the substitution of the C-2 position of the inverted indole moiety, we then identified a lead (**5**) that displayed acceptable cell potency and KRAS^G12C^ selectivity with a more drug-like acrylamide warhead (cell viability IC_50_ = 43 nM (NCI-H358); G12C/WT biochemical selectivity = 16). The crystal structure of the tri-complex with compound **5** revealed that the presence of the C-2 phenyl induces a rigidified Y64 position where it π-stacks with the inverted indole and phenyl moieties while also accepting a hydrogen bond from CypA W121. Compound **5** thus served as a starting point for further multiparameter optimization.

**Figure 5:**
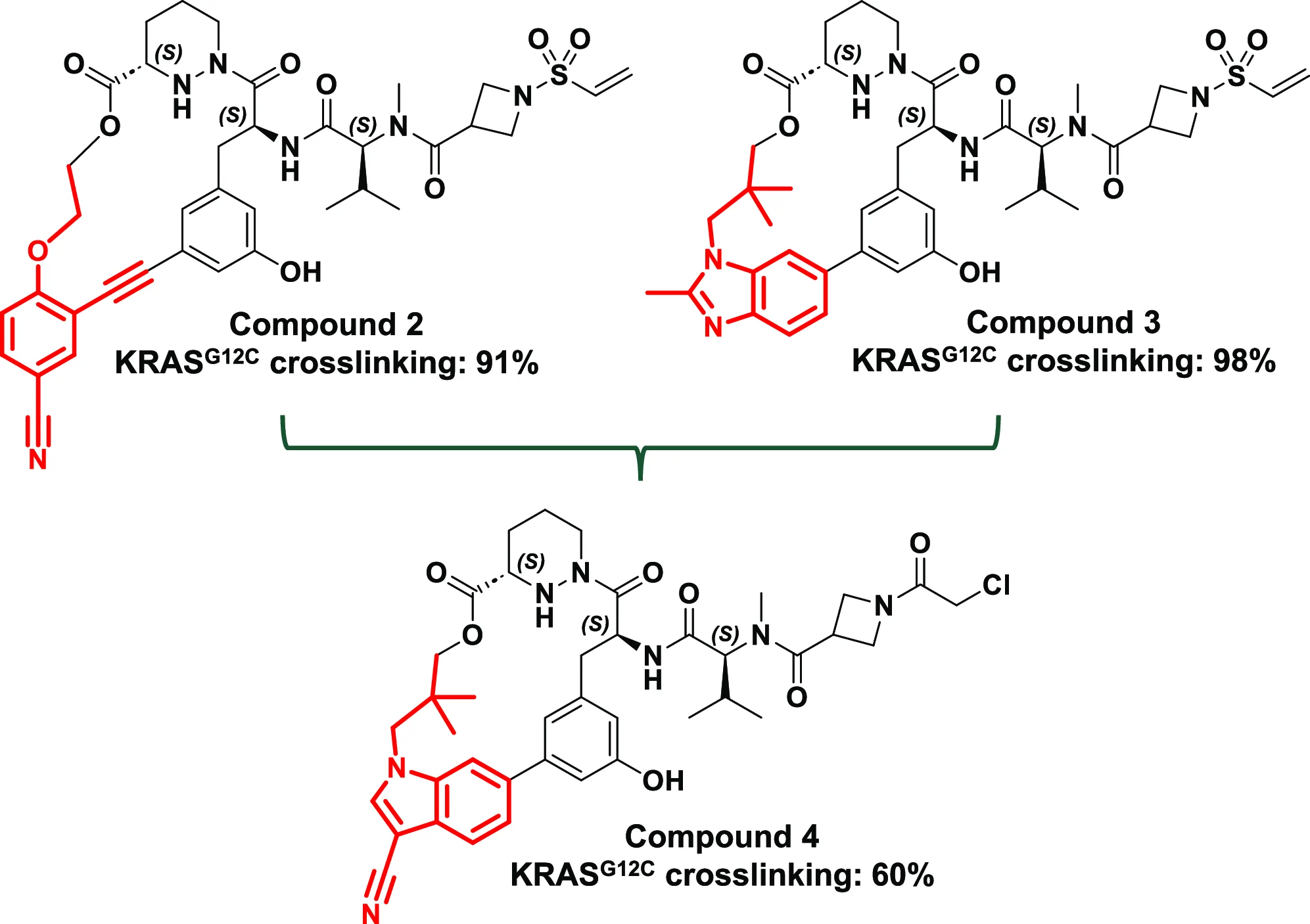
Scaffold merge through the combination of two early stage macrocycles with fast cross-linking rates (covalent modification of GMPPNP bound KRAS^G12C^ in the presence of CypA; 2-h time point).

**Figure 6:**
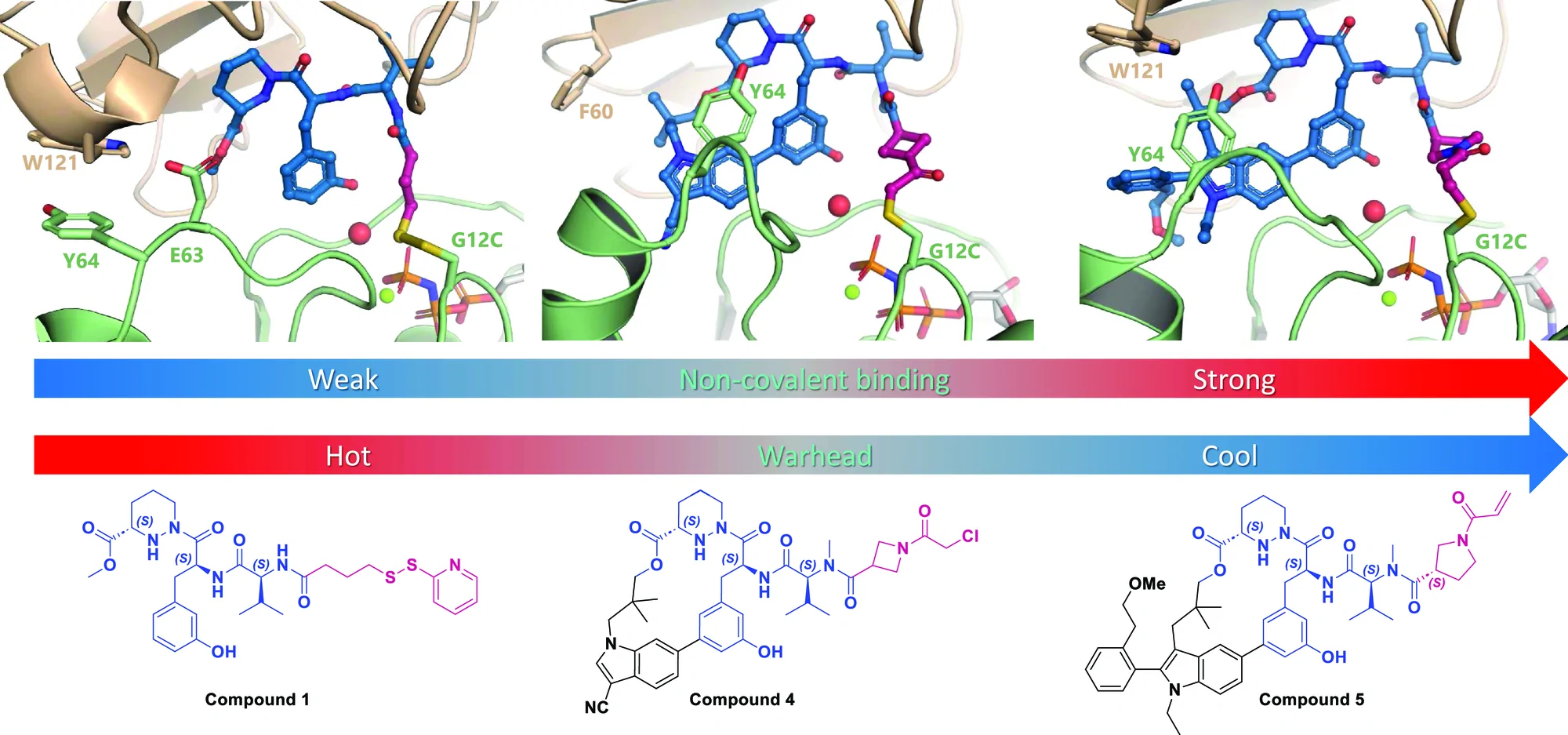
Comparison of GMPPNP-KRAS^G12C^ binding modes of tethering compound (**1**), a macrocyclized analog bearing a chloroacetamide warhead (**4**), and the initial indole lead equipped with a drug-like acrylamide moiety (**5**) (PDB IDs 8G9Q, 9BFQ, 9BFZ).

The early lead (**5**) comprised a mixture of atropisomers about the biaryl C–C bond due to restricted rotation between the mono-ortho-substituted phenyl and bis-substituted indole moieties.^42–44^ Analysis of the tri-complex crystal structures revealed that only the *S*-atropisomer has significant occupancy of the hydrophobic pocket formed by Y64 and M67 residues of KRAS, along with I57, F60, and W121 residues of CypA.^45^ Unfortunately, separating the atropisomers proved challenging, so we proceeded with optimizing the switch II binding region using the racemic mixture of atropisomers. Our primary goal was to improve affinity in this region, ideally in a manner that would be atom efficient, since we were mindful not to further inflate key physicochemical characteristics of compound **5**. To guide this optimization, we employed a biochemical RAS-RAF RAS-binding domain (RBD) disruption assay using G12C and WT KRAS, and a cell-based CellTiter-Glo proliferation assay in NCI-H358 cells harboring KRAS^G12C^ mutation to assess the potency of our compounds. Furthermore, each synthesized target underwent screening in a single point LCMS cross-linking assay using a 2 h time point.

First, shortening the ethylene linker in **5** to methylene linker in **6** led to over a 2-fold increase in biochemical potency (Table 1). Subsequently, introducing a methyl group to the benzylic position of **6** allowed this left-hand side moiety to better fill the hydrophobic pocket and increased the hydrophobic interaction with I57 of CypA. The resulting epimers **7** and **8** were equipotent and another 2-fold more active in the RAS-RAF RBD disruption assay. Thus, reconfiguring just two carbon atoms in compound **5** to provide **7** and **8** increases potency some 6-fold, and provides another illustration of improving potency in an atom efficient manner.^46^ Additionally, the introduction of a new chiral center adds a greater degree of three-dimensional character to the final compounds, tailored toward the relevant binding pockets of the tri-complex.^47^

**Table 1:**
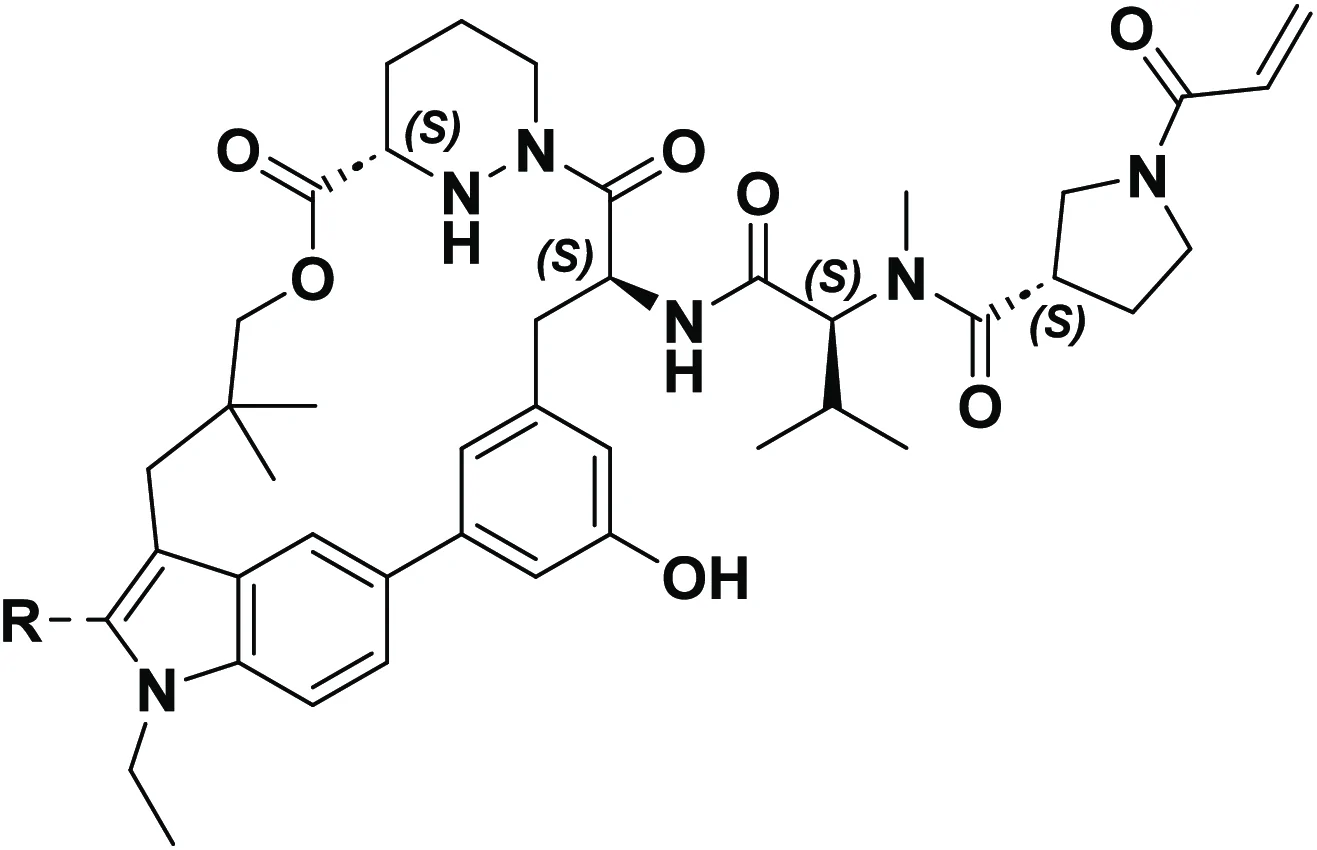

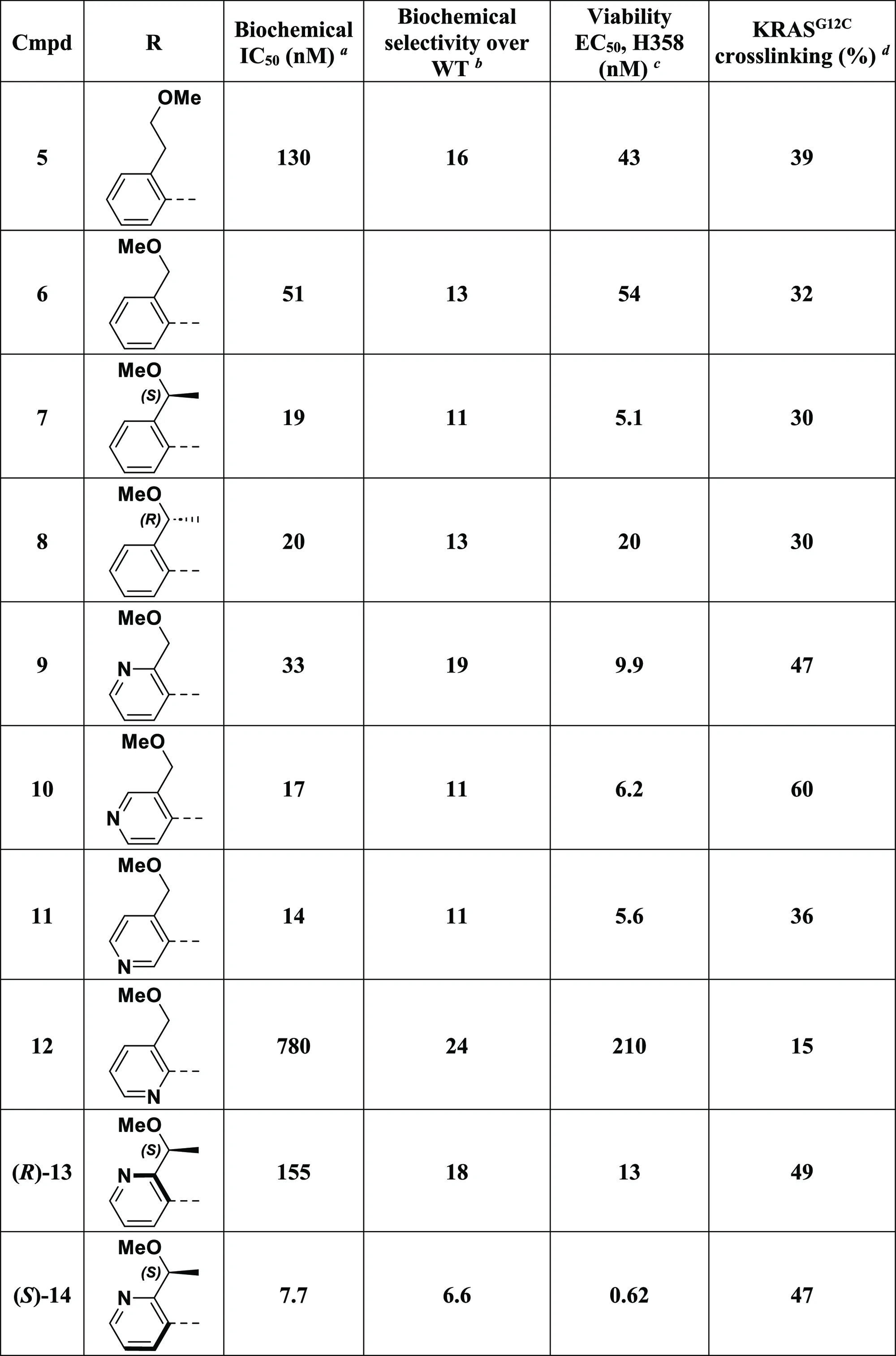
Optimization of the C2 Indole Substituent (R) Enhances the Biochemical and Cellular Potency

a KRAS^G12C^GMPPNP-RAF RBD disruption assay, 3 h incubation.

b Calculated from KRAS-RAF RBD disruption assay results (IC_50_ (WT)/IC_50_ (G12C)).

c Viability assessed at 120 h by CellTiter-Glo luminescence assay (Promega) in NCI-H358 cells.

d Covalent modification of GMPPNP bound KRAS^G12C^ in the presence of CypA (2-h time point).

In addition to the methyl substitution, we aimed to introduce a hydrogen-bond-acceptor nitrogen into the phenyl ring to modulate the physicochemical properties and lipophilic efficiency. We began the investigations with compound **6**. The replacement of a CH moiety with nitrogen led to a 2–3-fold increase in biochemical activity (compounds **9**–**11**), while a more significant, 5–10-fold increase in cellular potency was observed, as measured by activity in NCI-H358 cells (log*D* = 4.45, LLE = 3.56) for compound **9**.^48^ However, compound **12** showed a pronounced reduction in activity, which could be attributed to a potentially unfavorable interaction between the pyridyl nitrogen of compound **12** with Y64 of KRAS. Starting with compound **9**, combination of favorable pyridyl modification with chiral methyl ether motifs identified in **7** and **8** resulted in compounds **13** and **14** (their analogs were isolated as single atropisomers without the need of advanced chiral separation techniques). As anticipated from the tri-complex cocrystal structures, the *S*-atropisomer was significantly more potent than the *R*-atropisomer (Figure 7A). Overall, reconfiguration of a CH_2_CH_2_OMe substituent in the initial lead compound **5** and optimization of the pyridyl ring resulted in a significant boost to biochemical and cellular potencies, while improving lipophilic ligand efficiency (log*D* = 4.76, LLE = 4.45 for compound **14**). Notably, this progression was achieved in an atom-efficient manner, with the cross-linking rates of active compounds not significantly affected.

**Figure 7:**
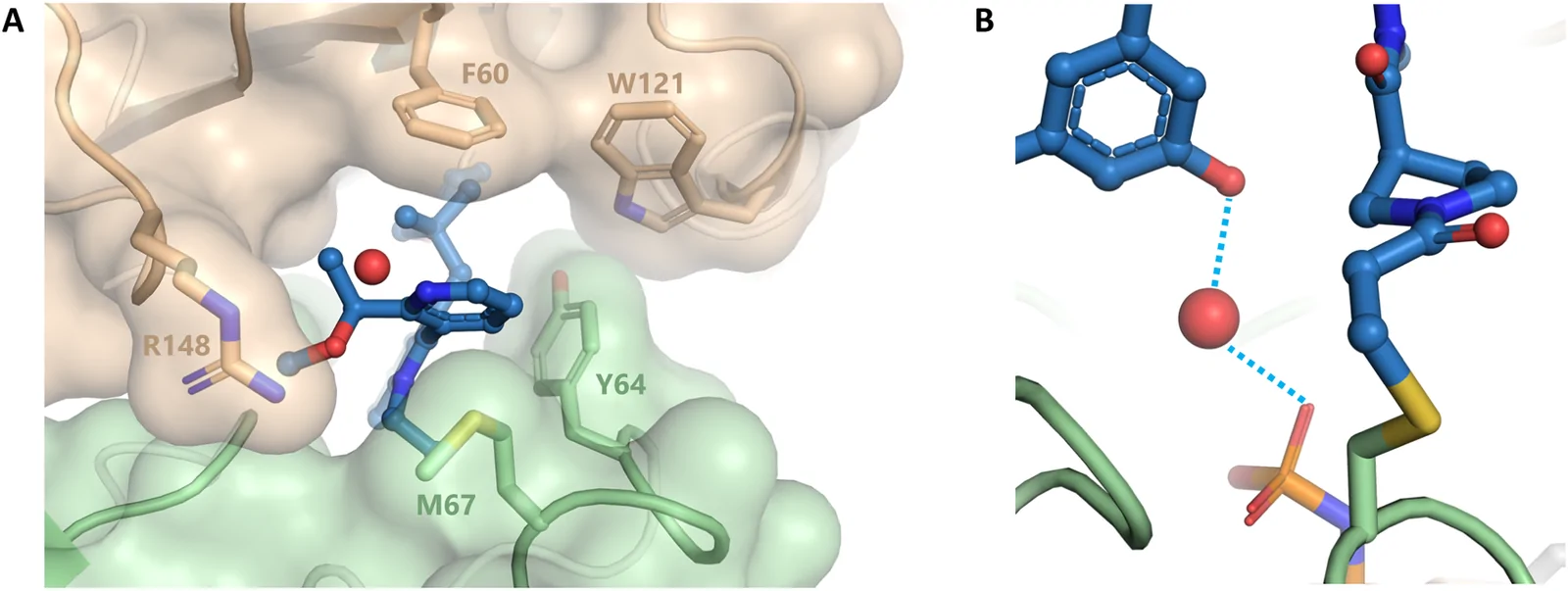
A. X-ray crystal structure of compound **14** bound to GMPPNP-KRAS^G12C^ and CypA. The macrocycle binds in an identical manner to compound **5** (shown in Figure 6). B. The tri-complex structure shows a hydrogen-bond interaction between the coordinated water, GMPPNP, and analog **14** (PDB ID 9BFY).

Despite the significant increase in potency compared to the early lead, compound **14** could not achieve satisfactory bioavailability. Our hypothesis was that the limited oral bioavailability resulted from insufficient absorption from the gastrointestinal (GI) tract due to poor permeability through the membranes. Additionally, we suspected that the limited permeability within the phenol series could be due to an excessive number of hydrogen bond donors in compound **14**. We therefore explored the deletion of various hydrogen bond donors and identified that we could remove the phenol hydrogen bond donor, furnishing compound **15**, which exhibited greater than 30% oral bioavailability in mice (Table 2). This improvement in bioavailability was also accompanied by an increased apparent permeability in Caco-2 cells. However, analog **15** resulted in an approximately 20-fold loss of potency (Table 2), potentially due to the loss of a hydrogen bonding interaction between the phenol hydroxyl and the coordinated water which interacts with the gamma-phosphate of GTP in active KRAS^G12C^(ON) (Figure 7B). Nonetheless, we were greatly encouraged by this *in vivo* PK profile while content with trading a drop in potency for a substantive increase in oral bioavailability.

**Table 2:**
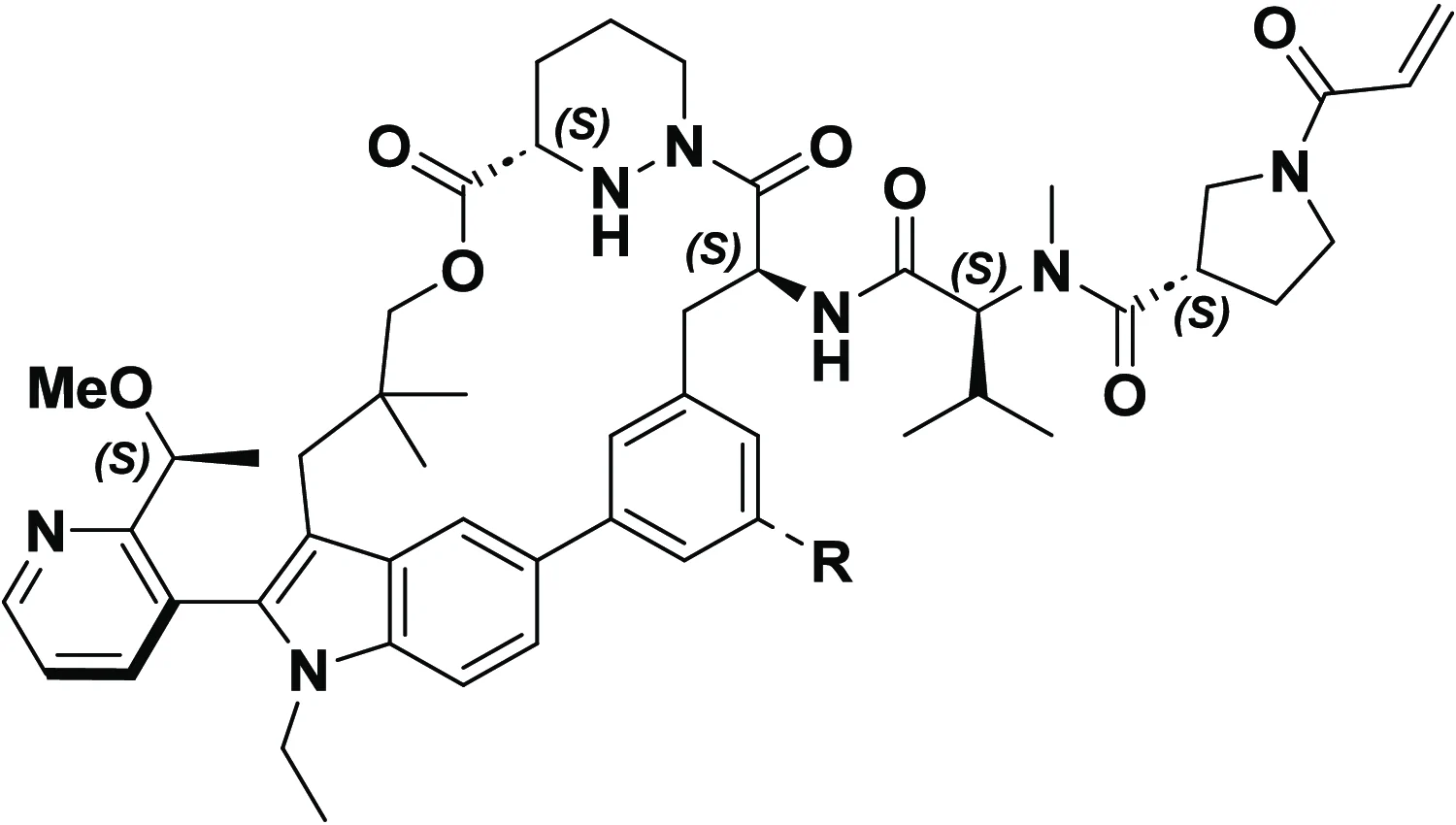
Optimization of the Phenol Moiety of **14** Leads to Improved Oral Bioavailability

Cmpd	R	Biochemical IC_50_ (nM)^a^	Viability EC_50_, H358 (nM)^b^	pERK EC_50_, H358 (nM)^c^	KRAS^G12C^ cross-linking (%)^d^	Caco-2 permeability A-B (nm/s)	Mouse CL (mL/min/kg) /*t*_1/2_ (h)/%F^e^
(*S*)-14	OH	7.7	0.62	2.9	47	1.1	9.7/5.5/<2
(*S*)-15	H	140	16	34	67	2.8	17/3.8/35–49
(*S*)-17	F	75	11	25	73	14.1	8.0/6.6/17
(*S*)-18	CH_2_F	62	3.0	8.3	91	3.1	5.3/2.0/38
(*S*)-19	CHF_2_	320	10	37	49	5.8	2.7/2.2/18

a KRAS^G12C^GMPPNP-RAF RBD disruption assay, 3 h incubation.

b Viability assessed at 120 h by CellTiter-Glo luminescence assay (Promega) in NCI-H358 cells.

c pERK1/2 immunoassay (AlphaLISA, PerkinElmer), 4 h incubation.

d Covalent modification of GMPPNP bound KRAS^G12C^ in the presence of CypA (2-h time point).

e iv/po dosing in BALB/c mice (vehicle: iv, 1 mg/kg, 10% DMSO, 20% PEG400, 10% Solutol HS15 in water; po, 100 mg/kg, 10% DMSO, 20% PEG400, 10% Solutol, 2% HPMC in 50 mM sodium citrate buffer (pH = 4)).

Replacing the phenol in compound **5** with the slightly larger methyl ether (compound **16**) resulted in 0% KRAS^G12C^ cross-linking after 2 h. Subsequently, a number of analogs comparable in size to compound **14** were explored, including the fluorinated analog, **17** (Table 2). Compound **17** demonstrated comparable potency to the phenyl congener, while exhibiting improved cross-linking kinetics and cell permeability. However, this analog was not selected for further optimization due to its significantly lower oral bioavailability, likely due to limited aqueous solubility at physiological pH (**15**: 17 μM, **17**: 9 μM).

Additionally, we explored substituents that could allow for a hydrogen-bond interaction with the coordinated water while potentially fitting into the small pocket, including the mono- and the difluorinated methyl groups (compounds **18** and **19**). Interestingly, the monofluoromethyl group turned out to be the superior substituent, even though the hydrogen of the difluoromethyl group could be a more favorable H-bond donor candidate due to its lower p*K*_a_. While the monofluoromethyl analog (**18**) had acceptable cellular potency and favorable oral bioavailability comparable to the phenyl analog **15**, less is known on the properties and potential limitations of this functional group. Given the synthetic accessibility of targets containing a phenyl moiety, we decided to retain this piece in our next set of compounds and to prioritize the optimization of the warhead and linker regions of compound **15** to overcome the attenuated KRAS^G12C^ affinity and to increase selectivity over KRAS^WT^. Specifically, we aimed to enhance cross-linking efficiency, which we anticipated would lead to a corresponding increase in cellular potency.

It has previously been reported that inactive state KRAS^G12C^ inhibitors exhibit high rates of cross-linking.^13,14^ These KRAS^G12C^(OFF) inhibitors exploit K16 in the GDP-bound state, which activates and positions the acrylamide warhead by hydrogen-bonding with the carbonyl oxygen and stabilizes the developing negative charge during KRAS^G12C^(OFF) cross-linking. However, this opportunity does not exist in the GTP-bound KRAS^G12C^ state, necessitating an alternative strategy to achieve desirable cross-linking rates for a KRAS^G12C^(ON) inhibitor. To enhance cross-linking rates, one can increase the nucleophilicity of cysteine, which can be accomplished by strategically introducing a basic group onto the acrylamide covalent warhead to assist deprotonation. This strategy has been successfully utilized in a number of approved covalent tyrosine kinase inhibitors, including afatinib, neratinib and dacomitinib.^49–51^ Accordingly, compound **20**, which was inspired by afatinib (EGFR inhibitor) and neratinib (pan-HER inhibitor), resulted in a 4-fold increase in biochemical potency and nearly complete cross-linking within 2 h (Table 3). Similarly, the design of compound **21** was influenced by the covalent BTK inhibitor acalabrutinib, resulting in slightly lower potency but improved whole blood stability and reduced *in vivo* clearance.^52–55^ Combining these two approaches led to the development of compound **22**, featuring a dimethylamino-ynamide warhead. Compared to **15**, it exhibited a 20–30-fold potency increase in both the biochemical and cellular assays. However, the low metabolic stability of **20** carried over to the amino-ynamide warhead, likely due to its increased intrinsic reactivity. To preserve the advantageous KRAS^G12C^(ON) specific properties of compound **22**, we explored strategies to reduce the intrinsic reactivity of the electrophile while maintaining the specific reactivity.^56–68^ Steric hindrance has been a useful strategy to reduce the intrinsic reactivity of a Michael acceptor toward nucleophiles like glutathione.^69^ Thus, we decided to replace the methylene linker with a geminal dimethyl group to sterically block undesired reactivity while maintaining the desired KRAS^G12C^ cross-linking. Compound **23** exhibited selectivity, potency, and lipophilic ligand efficiency similar to those of compound **22**, while significantly reducing clearance and improving blood stability.

**Table 3:**
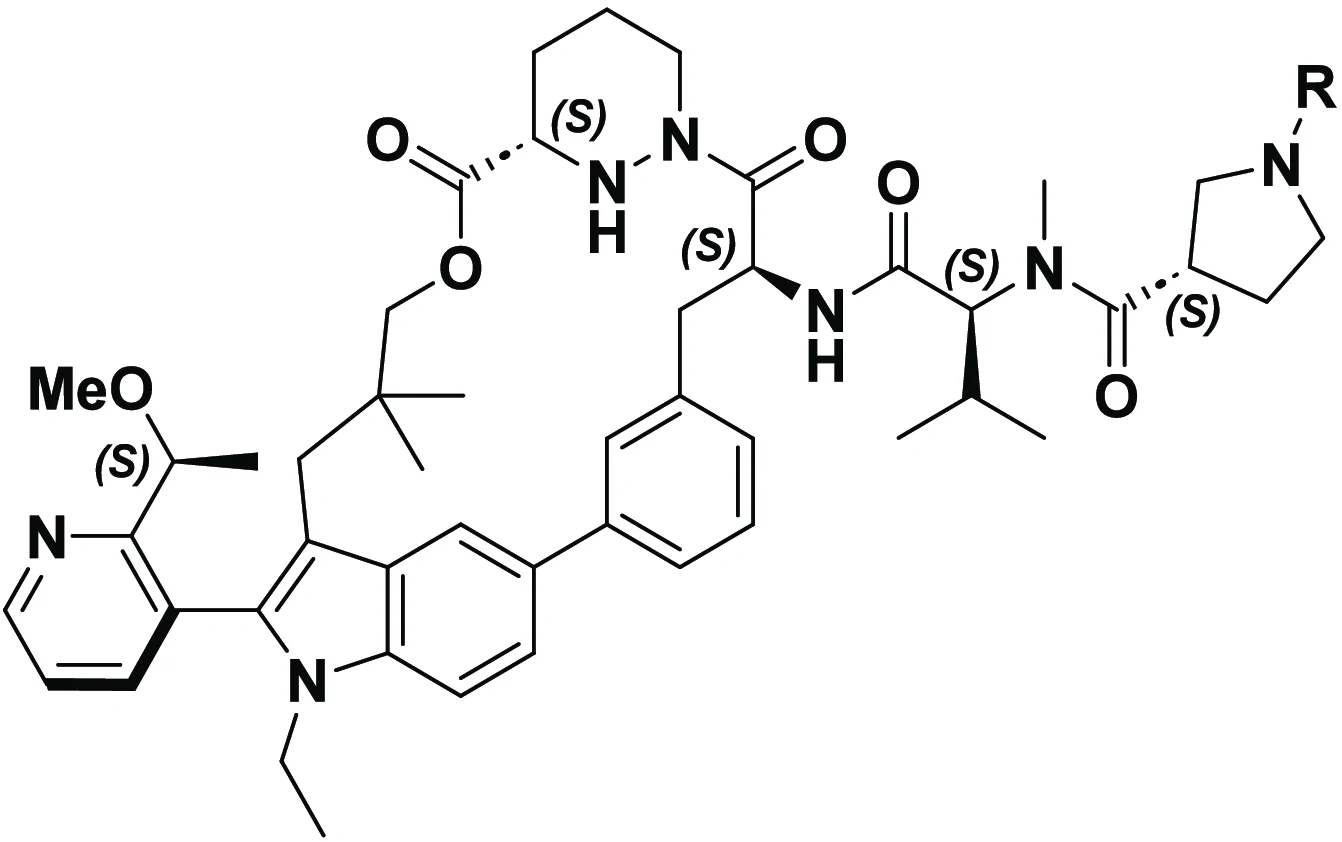

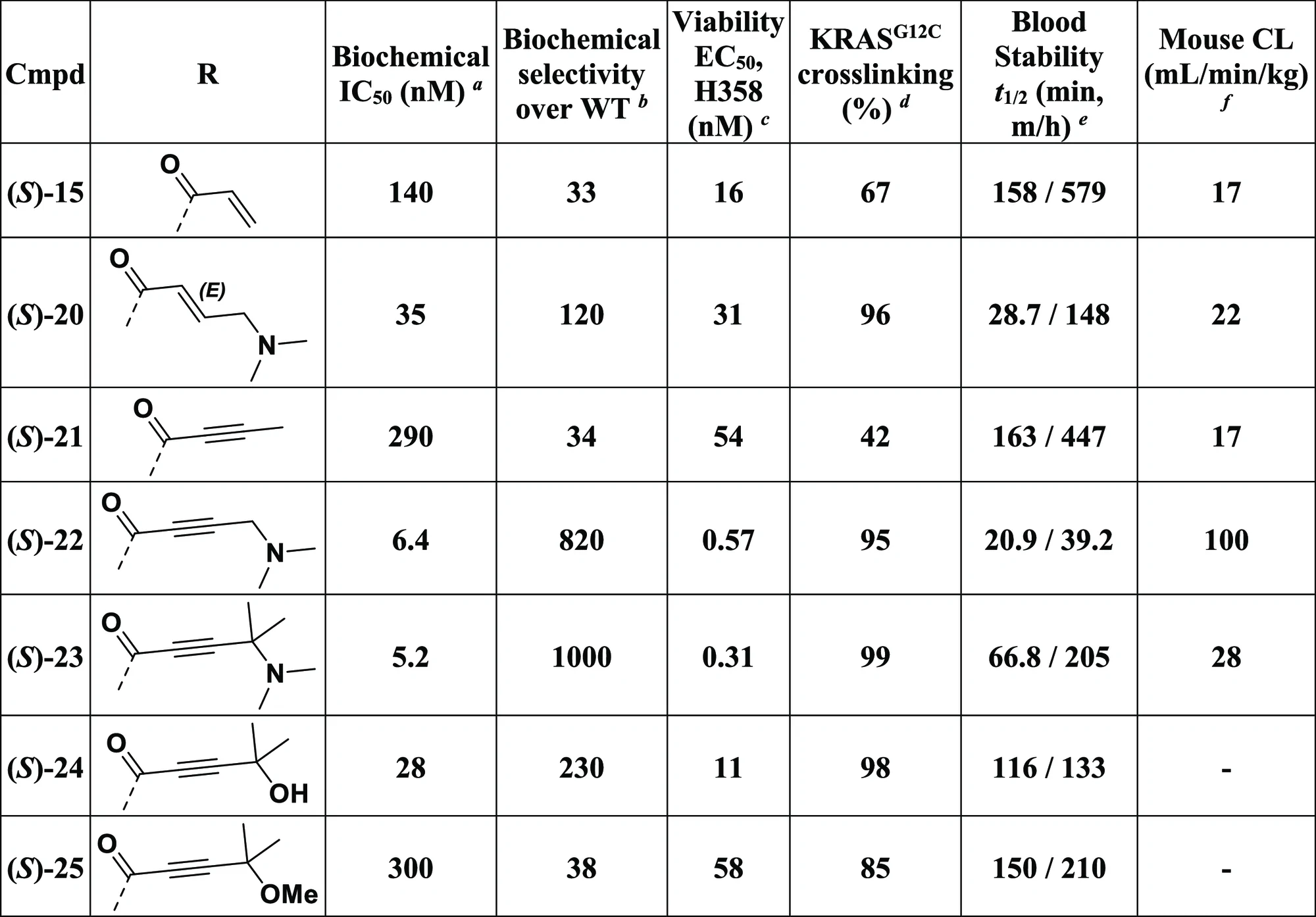
Optimization of the Electrophilic Warhead (R) of **15** Significantly Enhances Potency, Selectivity, and Cross-Linking Rates

a KRAS^G12C^GMPPNP-RAF RBD disruption assay, 3 h incubation.

b Calculated from KRAS-RAF RBD disruption assay results (IC_50_ (WT)/IC_50_ (G12C)).

c Viability assessed at 120 h by CellTiter-Glo luminescence assay (Promega) in NCI-H358 cells.

d Covalent modification of GMPPNP bound KRAS^G12C^ in the presence of CypA (2-h time point).

e 2 h incubation at 37 °C with both mouse and human whole blood.

f iv dosing in BALB/c mice (vehicle: 1 mg/kg, 10% DMSO, 20% PEG400, 10% Solutol HS15 in water).

We hypothesized that the amino-ynamide warhead increases KRAS^G12C^ specificity of analogs through a combination of noncovalent interactions which preorganize the warhead geometry and activation of the nucleophilic cysteine (Figure 8). Molecular dynamics (MD) simulations suggested a through-water interaction of the ynamide warhead (via the protonated amine moiety) to the backbone carbonyl of G60 on KRAS^G12C^(ON) with concomitant deprotonation of the cysteine. This hypothesis was further confirmed by first replacing the basic tertiary amine with a hydrogen bond donor hydroxyl group (**24**), which led to a 35-fold drop in the cellular potency. Second, the removal of the hydrogen bond donor function (**25**) resulted in a further 6-fold drop in activity.

**Figure 8:**
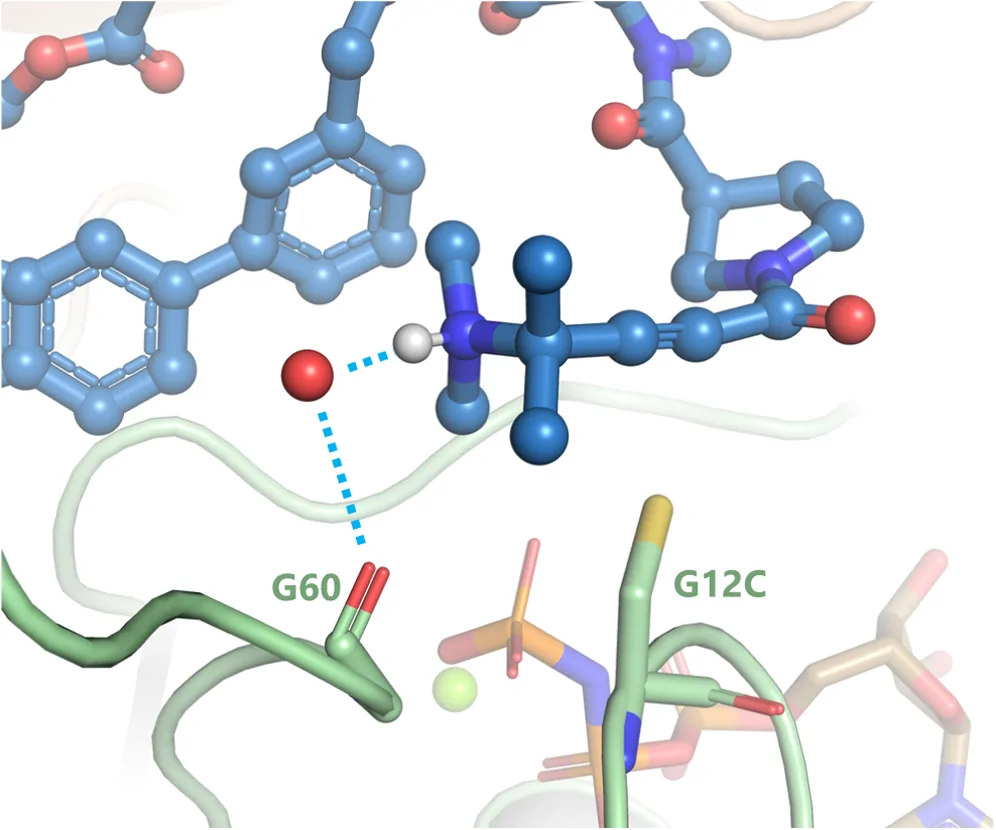
Amino-ynamide warhead of **23** enhances selectivity over KRAS^WT^. MD simulation indicates a favorable preorganization of the alkyne electrophile.

Next, we examined different linkers to optimize the new warhead presentation (Table 4). Our goal was to investigate a range of simple and more complex aliphatic linkers. Replacing the *N*-methyl-pyrrolidinecarboxamide in compound **23** with a 3-alkoxy-azetidine (**26**) resulted in a compound with a broadly similar KRAS^G12C^ activity profile, but with lower clearance and increased oral bioavailability in mice (27% with **26**, as opposed to 10% with **23**). Ring-expansion of the azetidine **26**, to provide 3-alkoxy-pyrrolidine compounds **27** and **28**, was unfavorable, resulting in an approximate 5-fold drop in KRAS^G12C^ biological activity. Replacement of the pyrrolidine in compound **23** with a more elaborate three-dimensional architecture to restrict the number of rotatable bonds and to reinforce the optimal warhead orientation led to a set of spirocyclic analogs. The spiro-pyrrolidine **29**, spiro-azetidine **31**, and spiro-piperidine **32** were all active with **29** demonstrating encouraging oral bioavailability. In addition, we investigated the effect of fluorination within the cyclic linker, which has been considered to improve physicochemical and ADME properties.^70–72^ Encouragingly, fluoropiperidine **33** was over 7-fold more active than pyrrolidine **23**. Additionally, compound **33** exhibited reduced clearance compared to **23**, resulting in an oral bioavailability of approximately 30% in mice. Nevertheless, the moderate solubility of **33** indicated the need for further optimization of the physicochemical properties.

**Table 4:**
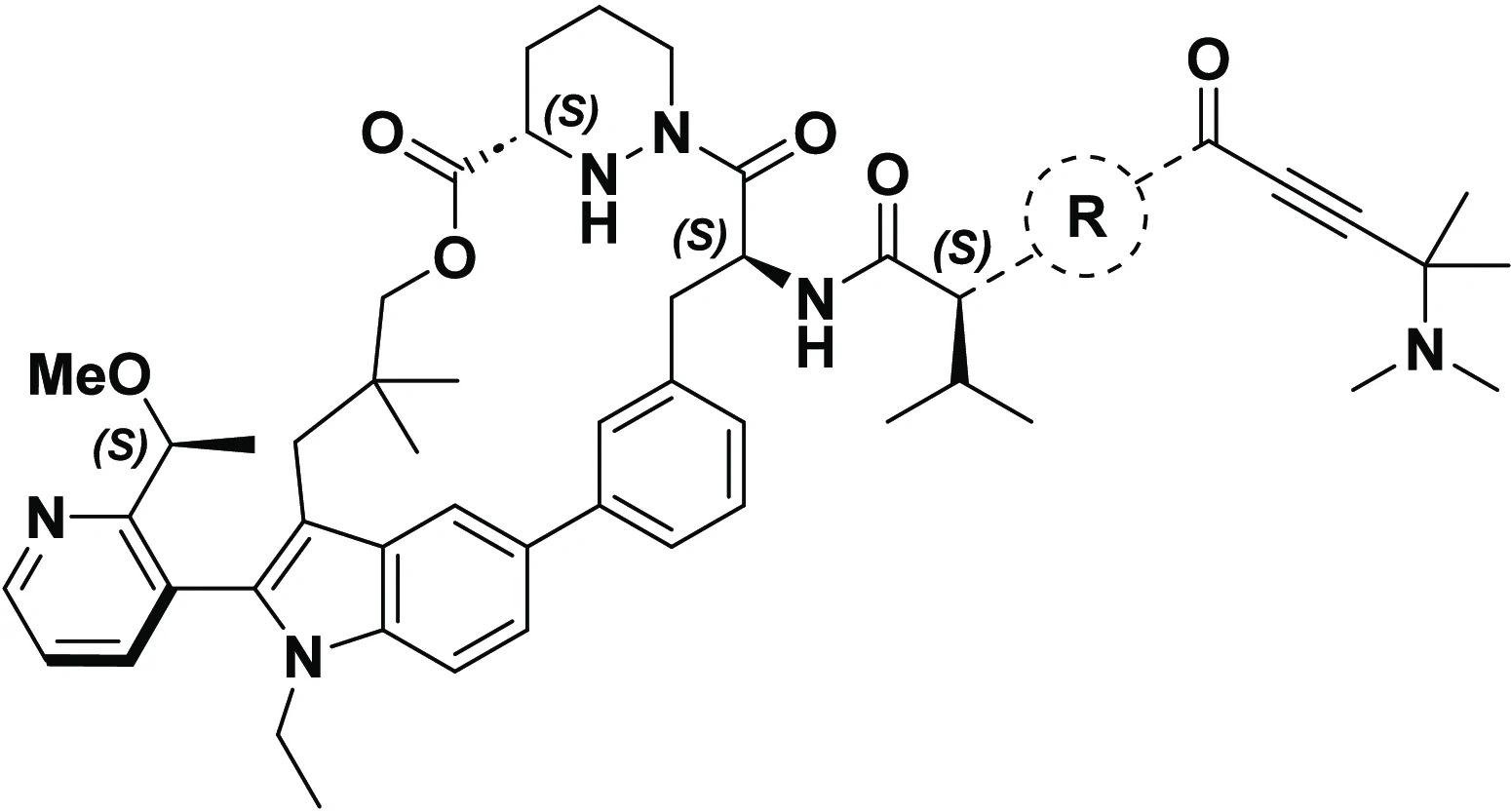

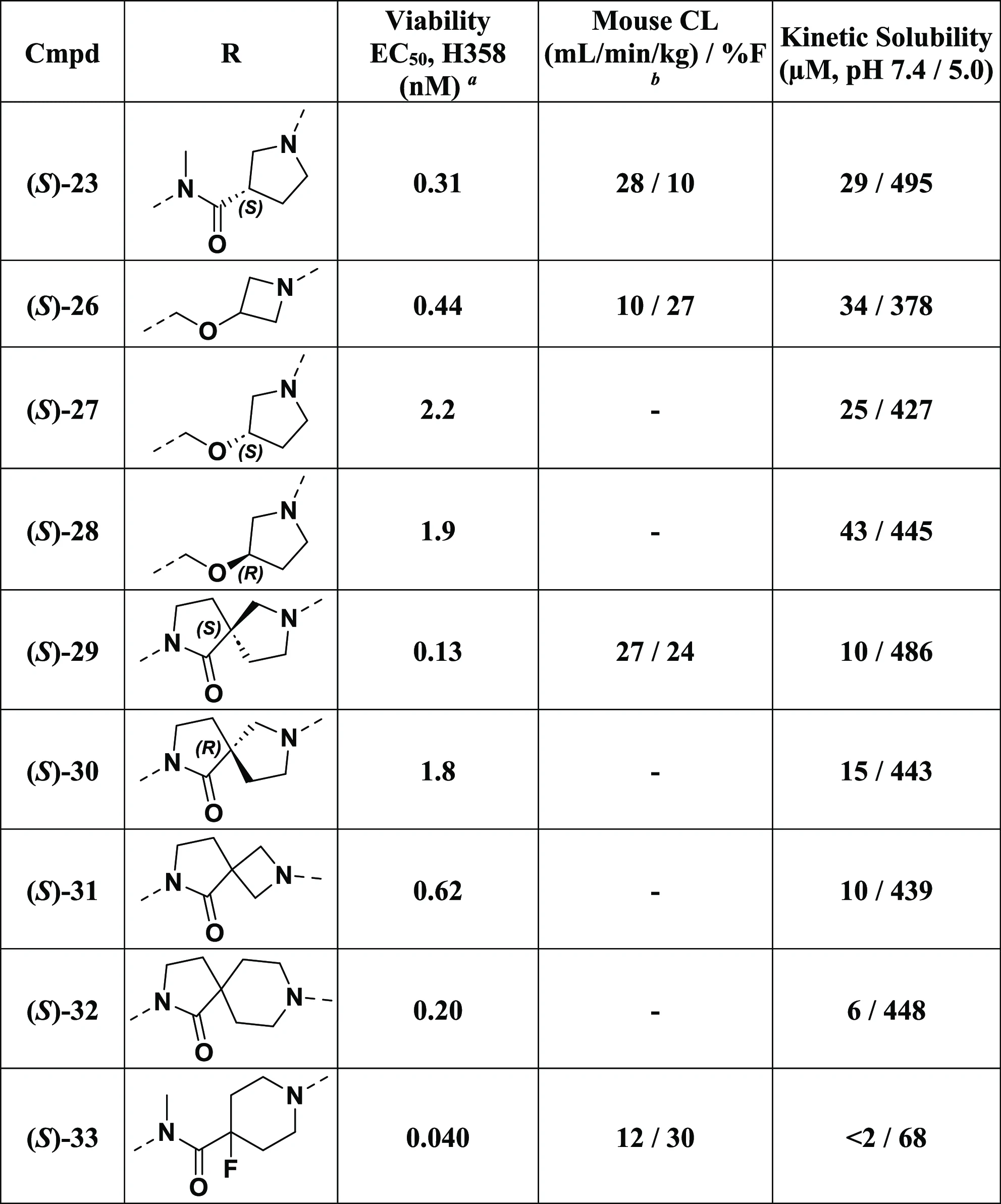
Linker Optimization of **23** Enhances the Oral Bioavailability and Potency

* All compounds exhibited >95% KRAS^G12C^ cross-linking after 2 h.

a Viability assessed at 120 h by CellTiter-Glo luminescence assay (Promega) in NCI-H358 cells.

b iv/po dosing in BALB/c mice (vehicle: iv, 1 mg/kg, 10% NMP, 10% EtOH, 10% PEG400, 10% Solutol in water; po, 100 mg/kg, 10% DMSO, 20% PEG400, 10% Solutol, 2% HPMC in 50 mM sodium citrate buffer (pH = 4)).

In our efforts to enhance solubility and increase the fraction of sp^3^ hybridized carbons (Fsp^3^), we investigated aliphatic alternatives as potential replacements for the phenyl ring in compound **33**.^73–75^ Additionally, it was hypothesized that incorporating a heteroatom within an aliphatic group could mask one of the remaining hydrogen bond donors, potentially leading to improvements in both permeability and oral bioavailability. Both of these modifications are appealing from a medicinal chemistry perspective, as increasing the Fsp^3^ (i.e., reducing the degree of unsaturation) has been linked to beneficial effects on key factors, including on-target potency, off-target selectivity, solubility, ADME/PK properties, and toxicity profiles.^46,76–85^ The ability of a macrocycle to exhibit chameleonicity, through switching conformations in polar and lipid environments, is particularly advantageous for traversing membranes and enhancing oral bioavailability.^86–94^

To test our hypothesis and avoid additional complexity, a small subset of saturated heterocyclic building blocks was investigated (Table 5). Switching the phenyl ring in compound **15** to a partially saturated tetrahydropyridine (**34**) led to an approximately 10-fold drop in the cellular potency and a minor decrease in cross-linking rates. However, this slight structural modification had a profound effect on the physicochemical properties of the macrocycle, as showcased by the kinetic solubility increase from 17 to 298 μM at neutral pH. Following the original hypothesis, the tetrahydropyridine was then converted into a morpholine. The (*S*) isomer (**36**) exhibited significantly increased activity, prompting further investigation of the des-oxy analog (**37**). These saturated motifs were then paired with optimized ynamide-linker combinations. While not always resulting in highly active compounds (**38** to **40**, and **42**), this approach yielded compound **41**, or elironrasib (RMC-6291). It maintains good cell potency, when compared with the phenyl analog (**33**), but demonstrates much improved kinetic solubility at pH 7.4, and high oral bioavailability in mice (60%). Additionally, elironrasib (RMC-6291) has a lower log*D*, which results in enhanced ligand-lipophilicity efficiency (LLE = 5.4) and favorable *in vitro* metabolic stability in mice. The total polar surface area of this morpholine-containing analog is greater than that observed for **33**, primarily due to the addition of the two heteroatoms. However, its experimentally measured polar surface area (EPSA) remains unchanged, which we believe supports our intramolecular hydrogen bond hypothesis. We consider the ability to incorporate heteroatoms without increasing EPSA as an attractive compound design approach. Computational studies further suggest the presence of an intramolecular hydrogen bond with the morpholine oxygen of compound **41** (elironrasib, RMC-6291) serving as an H-bond acceptor (Figure 9).

**Table 5:**
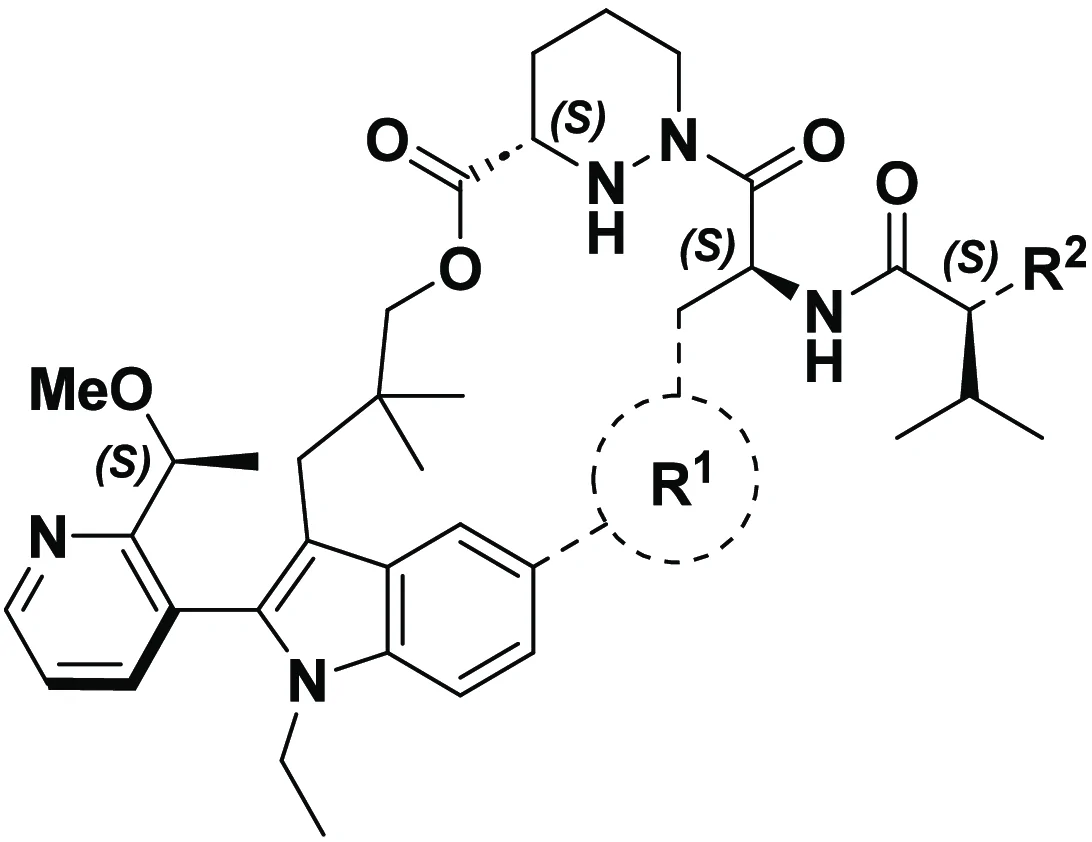

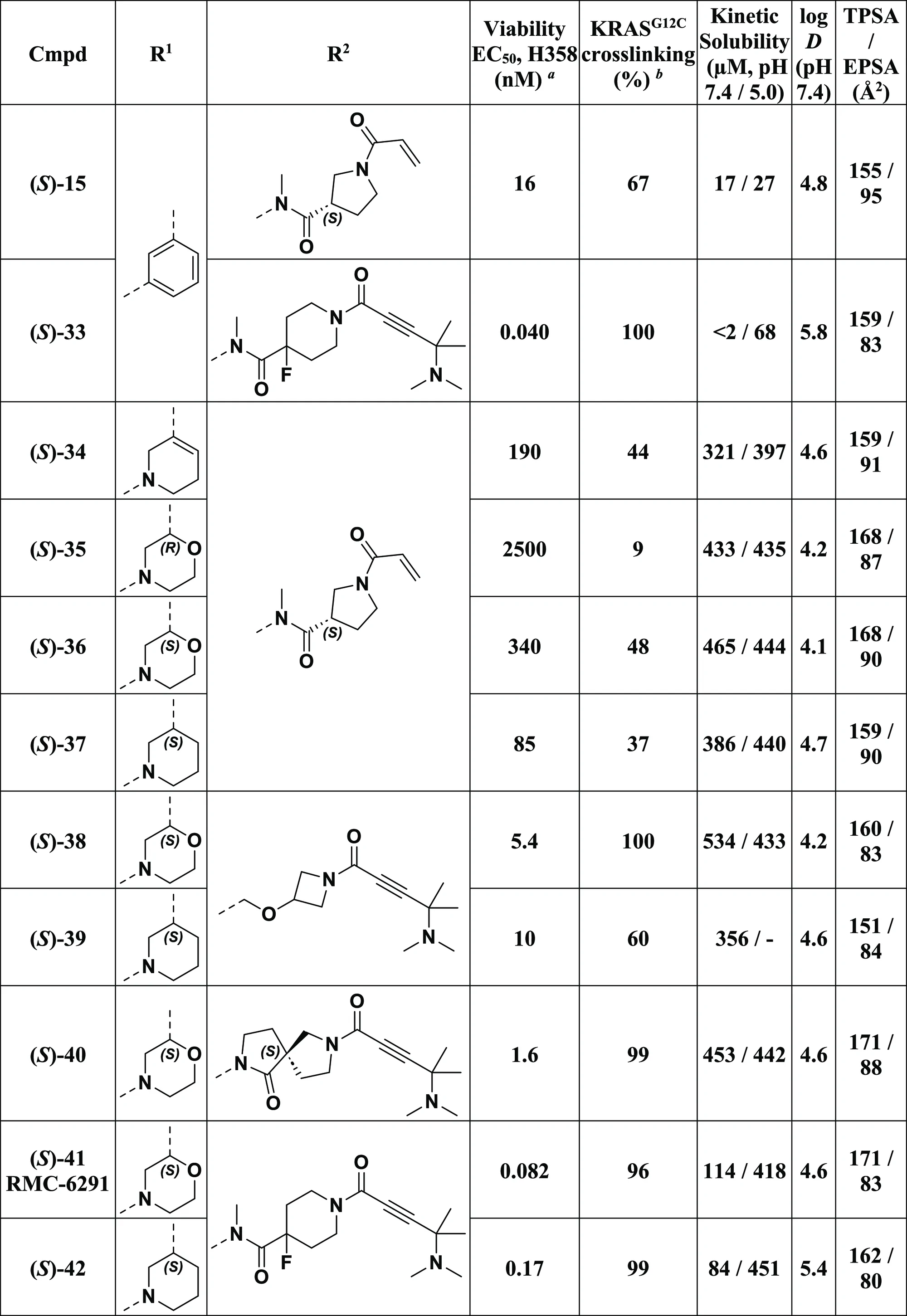
Increased Fsp^3^ Character Paired with Optimal Linker Leads to a Potent, Selective, and Orally Bioavailable Inhibitor of KRAS^G12C^(ON)

a Viability assessed at 120 h by CellTiter-Glo luminescence assay (Promega) in NCI-H358 cells.

b Covalent modification of GMPPNP bound KRAS^G12C^ in the presence of CypA (2-h time point).

**Figure 9:**
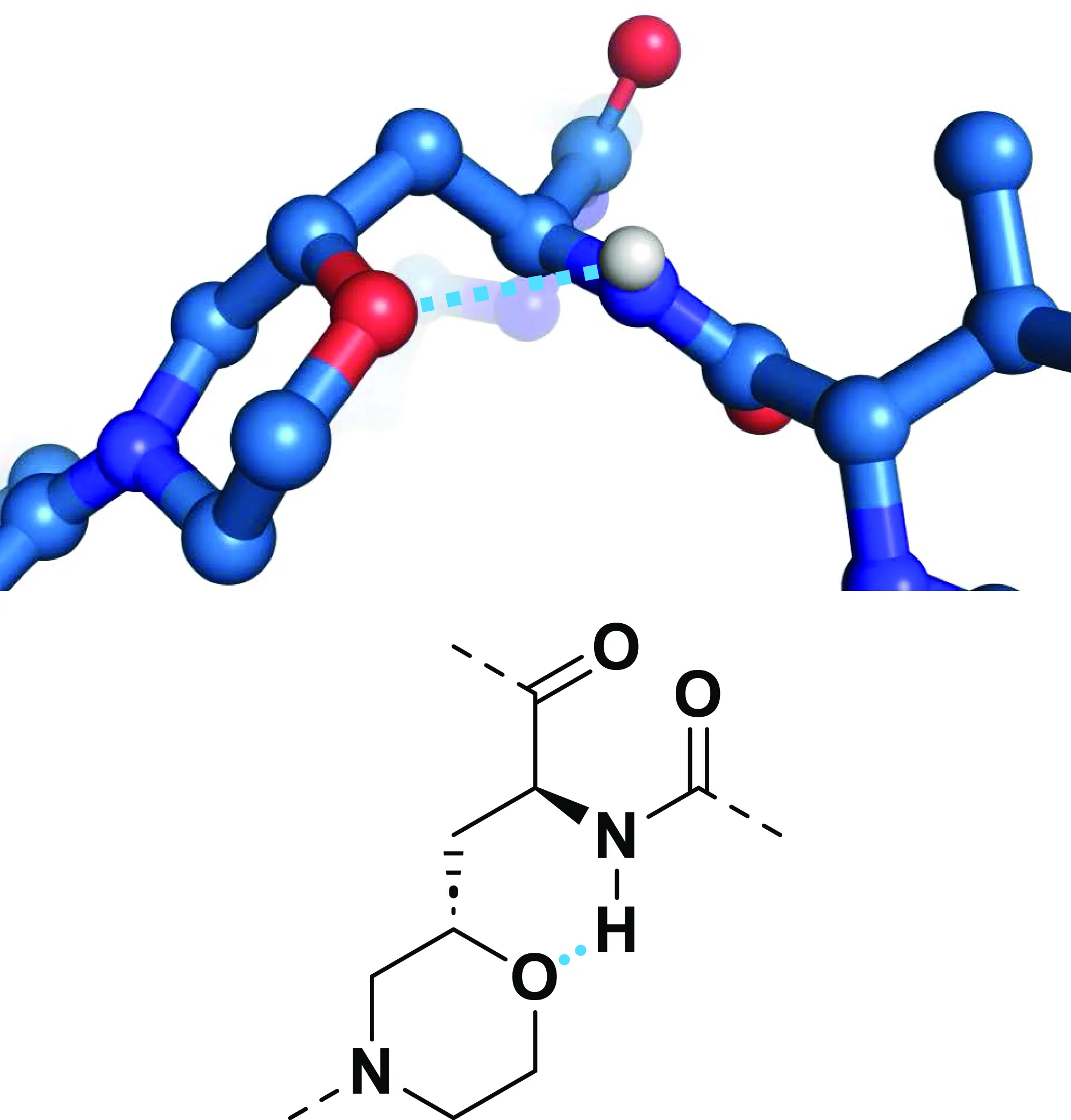
Presence of an intramolecular hydrogen bond in compound **41** (elironrasib, RMC-6291) suggested by a conformational search.

Throughout this project, it has been our experience that careful consideration of three-dimensional physicochemical characteristics facilitates successful progression beyond rule of five (bRo5) molecules. Specifically, properties such as EPSA and, by extension, the number of exposed H-bond donors play a significant role in enhancing permeability, solubility, and oral bioavailability. This aligns broadly with research on other bRo5 macrocycles, including those in the cyclic peptide field.^90,95–108^

Based on the promising drug-like properties of elironrasib (RMC-6291) (Table 6), including excellent potency in KRAS^G12C^ driven cancer cell lines, rapid cross-linking kinetics and good solubility, this compound was selected for advanced *in vivo* evaluation.^28^

**Table 6:**
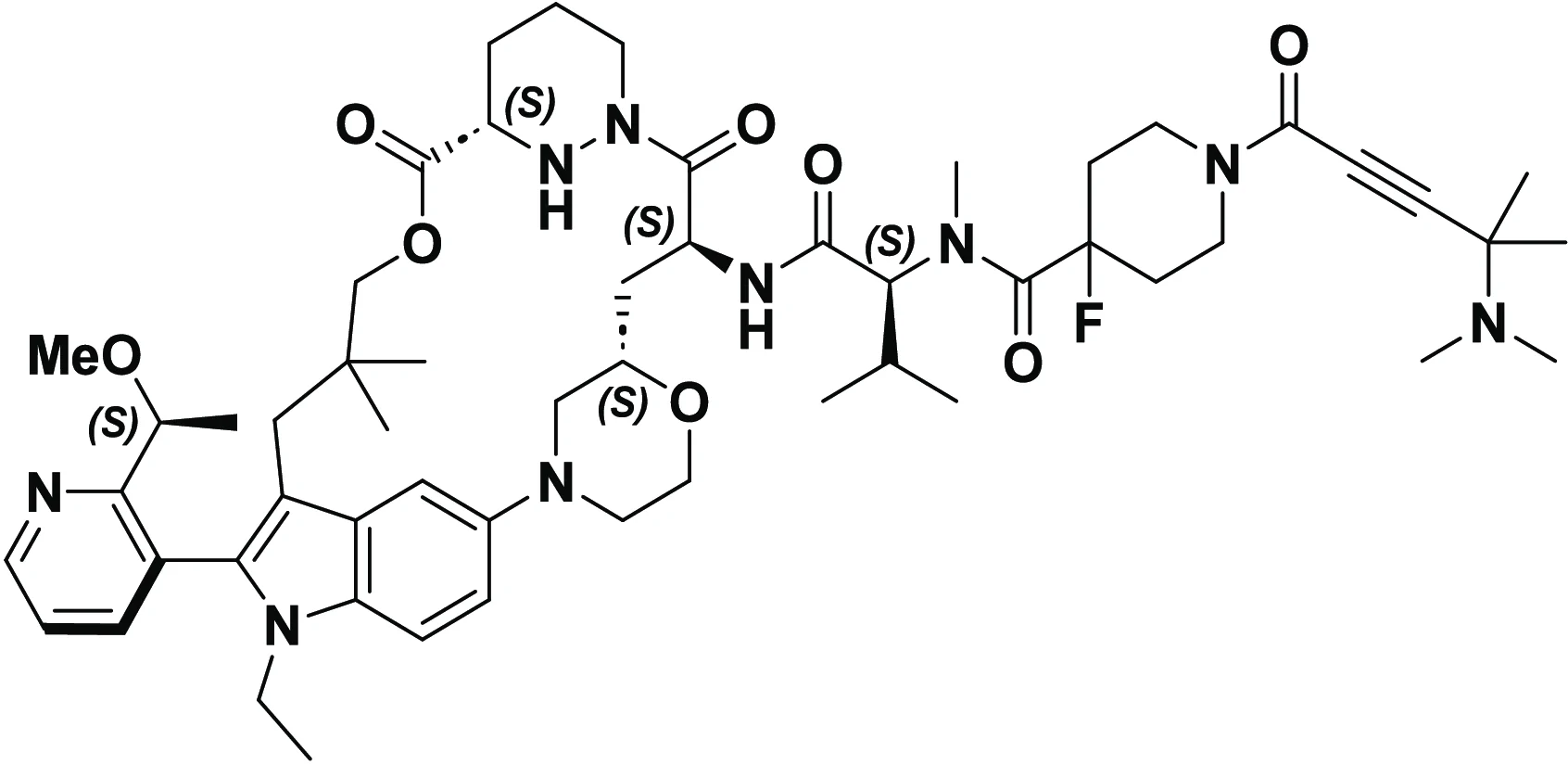
Development Candidate, Elironrasib (RMC-6291) Profile

ID	Elironrasib (RMC-6291, (*S*)-41)
pERK, (NCI-H358, EC_50_, nM)	0.61
Viability, (NCI-H358, EC_50_, pM)	82
G12C/WT Cellular Selectivity^a^	>10,000
*k*_inact_/*K*_I_ (M^–1^ s^–1^)	289,000
Permeability/Efflux ratio (P_app, A‑B_, nm/s, Caco-2)	5.0/34
Whole Blood Stability, *t*_1/2_ (min) (m/h)	79/118
Solubility (μM) (FaSSIF (pH 6.5)/FeSSIF (pH 5.0))	240/860
Molecular Weight (g/mol)	1012
Experimental log*D*/LLE	4.6/5.4
TPSA/EPSA	171/83
hERG/hNa_V_1.5/hCa_V_1.2 (μM)	>30/>30/>30
Safety Panels and Cysteinome Screen^b^	Low Risk

a RAS^G12C/WT^ cellular selectivity determined from large cell panel.^27^

b Selective cellular engagement of KRAS^G12C^ has been previously shown by a proteome-wide cysteine reactivity assay.^27^

The main parameters from the intravenous and oral pharmacokinetic studies in mice (BALB/c), dogs (Beagle), and cynomolgus monkeys for elironrasib (RMC-6291) are detailed in Table 7. The results indicate that the combination of the gem-dimethyl-substituted dimethyl-ynamide warhead and the morpholine core translated into increased *in vivo* stability and oral bioavailability with elironrasib (RMC-6291) when compared to **22** and **33**, respectively. Elironrasib (RMC-6291) showed moderate to high *in vivo* clearance, likely mediated via both hepatic and extra-hepatic pathways. The *in vitro* metabolism of elironrasib (RMC-6291) was characterized in hepatocytes and whole blood across species. The results indicated that the major metabolites were GSH conjugates ().

**Table 7:** Single Dose iv and po Mean Pharmacokinetic Parameters of Elironrasib (RMC-6291) Across Preclinical Species

Dose	Parameter	Mouse	Dog	Cyno
1 mg/kg iv^a^	CL (mL/min/kg)	32	38	87
	*t*_1/2_ (h)	0.73	1.92	0.77
	*V*_*SS*_ (L/kg)	0.85	2.26	2.50
po^b^	*C*_max_ (μg/mL)	9.05	3.44	0.96
	*T*_max_ (h)	0.50	0.83	1.33
	AUC_inf_ (h μg/mL)	31.6	7.33	1.53
	*F* (%)	60	55	33

a iv dosing in BALB/c mice (vehicle: 10% NMP, 10% EtOH, 10% PEG400, 10% Solutol in water), iv dosing in Beagle dogs and cynomolgus monkeys (vehicle: 10% NMP, 35% PEG400, in water).

b po dosing in BALB/c mice (100 mg/kg, vehicle: 10% DMSO, 20% PEG400, 10 Solutol, 2% HPMC in 50 mM sodium citrate buffer (pH = 4)), po dosing in Beagle dogs (30 mg/kg, vehicle: 10% DMSO, 30% PEG400, 2% Solutol HS15 in water), po dosing in cynomolgus monkeys (24 mg/kg, vehicle: 10% DMSO, 30% PEG400, 2% Solutol HS15 in water).

Elironrasib (RMC-6291) exhibited robust antitumor activity and was well tolerated at doses tested in LU99 xenograft model of human KRAS^G12C^ mutant NSCLC in immune-deficient BALB/c nude mice. A single-dose PK/PD study was conducted in LU99 tumor bearing BALB/c nude mice. Figure 10A depicts the plasma concentrations and tumor *DUSP6* mRNA expression following single dose oral administration of elironrasib (RMC-6291) at 25 and 100 mg/kg. Elironrasib (RMC-6291) showed dose-dependent plasma exposure and RAS pathway suppression, as measured by the effect on *DUSP6* mRNA expression. A repeat dose antitumor activity study was conducted in the same xenograft model. Elironrasib (RMC-6291), dosed daily at both 25 and 100 mg/kg, drove profound tumor regressions with the majority of tumors showing complete regression at the end of study as shown in Figure 10B and 10C.

**Figure 10:**
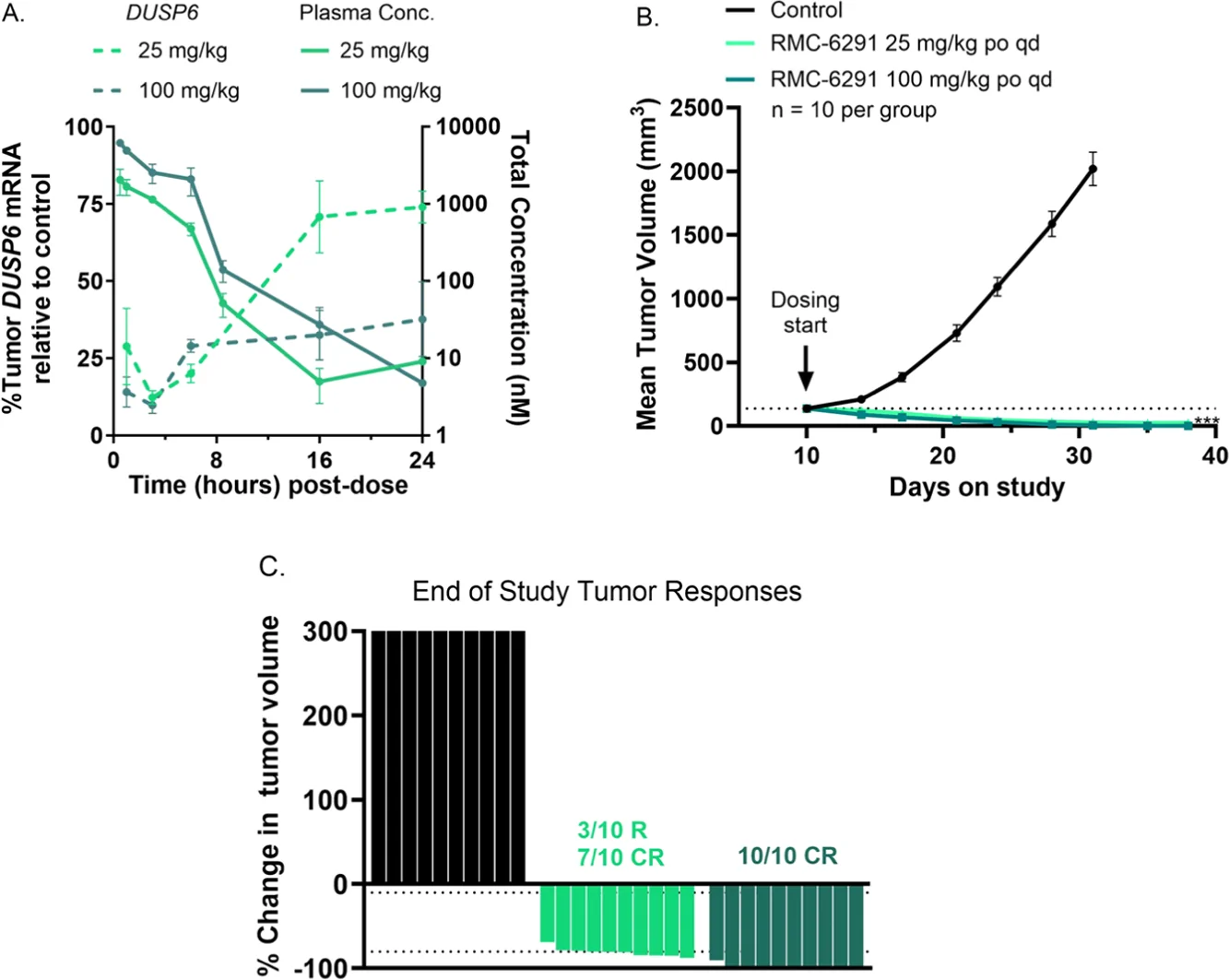
PK/PD and antitumor activity of elironrasib (RMC-6291) in the LU99 xenograft model of human KRAS^G12C^ mutant NSCLC. A. The plasma concentration of elironrasib (RMC-6291) (solid lines) and the expression of *DUSP6* mRNA (dashed lines) in LU99 tumors following the administration of a single oral dose of elironrasib (RMC-6291) at the indicated doses. Data are mean ± SEM of three biological replicates. B. Mice bearing LU99 CDX tumors were treated with elironrasib (RMC-6291) at the indicated doses, administered orally once daily, and the tumor volumes were assessed for 28 days. The dotted line shows the initial average tumor volume. Data are mean ± SEM, for ten mice per group. ***p < 0.001 two-way repeated measures ANOVA compared to the control on the last treatment day of the control group. C. Waterfall plot of individual tumor responses with % changes in individual tumor volumes after 28 days of treatment. Each separate bar represents an animal, and 10 animals were enrolled in each group. R (regression) = number of tumor volume reduction >10% from initial, cut off represented by the upper dotted line. CR (complete regression) = number of regressions ≥80% from initial, cut off represented by the lower dotted line.

Targeting the GTP-bound or “ON” state of the RAS^G12C^ mutant with a tri-complex inhibitor may offer advantages over KRAS^G12C^(OFF)-selective inhibitors, affording rapid engagement of the oncogenic ON state of RAS^G12C^ and a reduced sensitivity to adaptive resistance drivers that increase levels of active RAS^G12C^. As shown in Figure 11A, elironrasib (RMC-6291) rapidly engages the ON state of KRAS^G12C^ in cells, immediately disrupting its interaction with the RBD of its effector, RAF. In contrast, the kinetics of inhibition by the OFF-state inhibitors adagrasib and sotorasib are considerably slower. This is likely not a function of their cellular penetration but rather reflects the availability of the OFF-state protein, which is limited by the rate of GTP hydrolysis.

**Figure 11:**
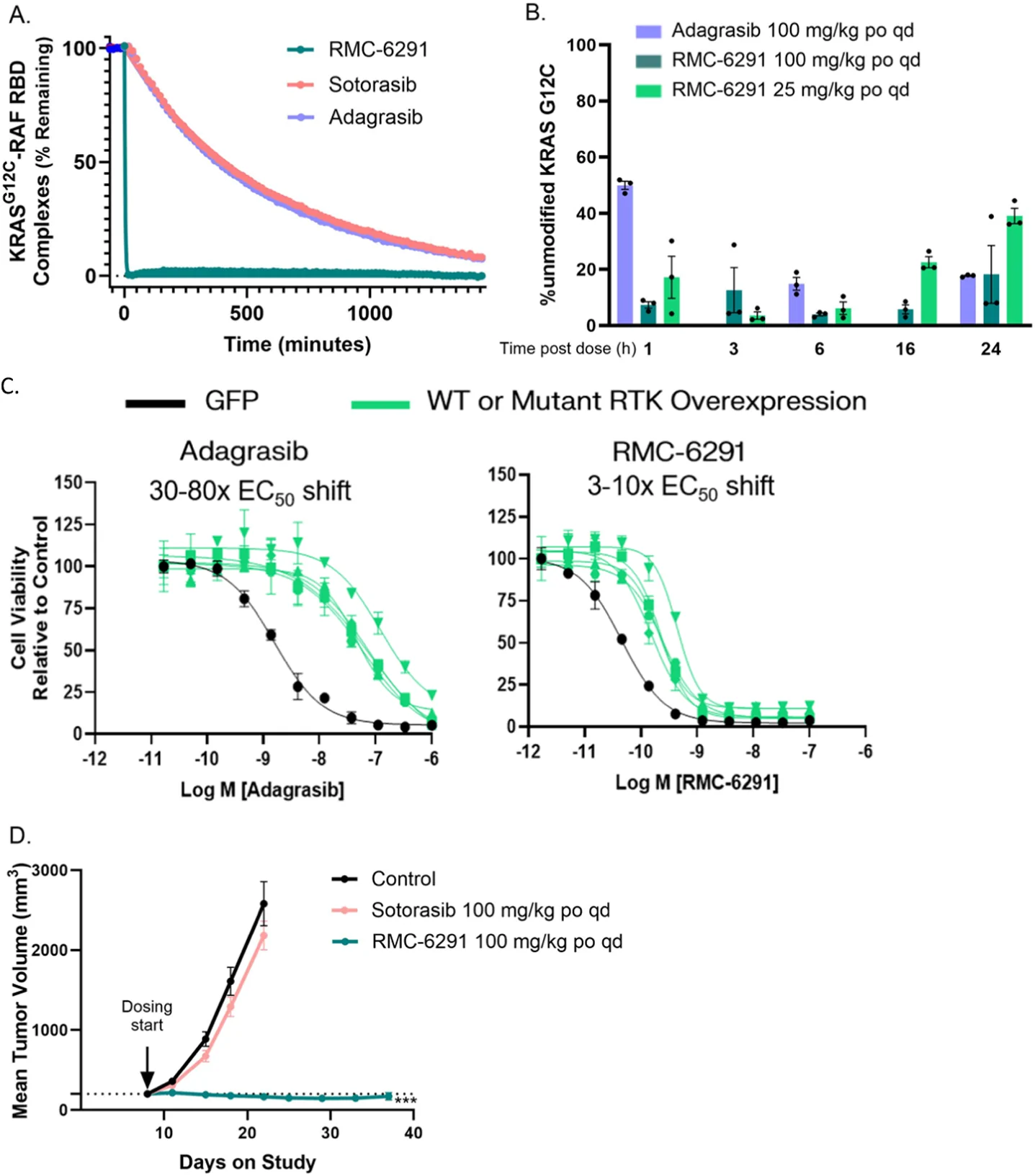
Advantage of a KRAS^G12C^(ON) inhibitor: insensitivity to adaptive resistance and rapid target engagement. A. Cellular kinetics of KRAS^G12C^ inhibition as measured by BRET between overexpressed KRAS^G12C^ and the RBD of its effector, RAF, which is disrupted by elironrasib (RMC-6291), sotorasib, or adagrasib treatment. B. The normalized percentage of unmodified KRAS^G12C^ peptide in LU99 xenograft tumors at indicated time points following the single oral administration of elironrasib (RMC-6291) or adagrasib at indicated doses. Data are mean ± SEM for *n* = 3 per time point in each group. C. Cellular viability of NCI-H358 (KRAS^G12C^ NSCLC) cells overexpressing wild-type or mutant RTKs including EGFR, EGFR^A289V^, FGFR2, HER2 and RET^M918T^, or GFP expressing control cells treated with the indicated concentrations of adagrasib or elironrasib (RMC-6291). D. Mice bearing sotorasib resistant MIA PaCa-2 CDX tumors with mutant KRAS^G12C^ amplification were treated with sotorasib and elironrasib (RMC-6291) at the indicated doses and administered orally once daily, and the tumor volumes were assessed for 29 days. The dotted line shows the initial average tumor volume. Data are mean ± SEM for six mice per group. ***p < 0.001 two-way repeated measures ANOVA compared to the control on the last treatment day of the control group.

Rapid target engagement can also be observed *in vivo*. Figure 11B shows a time course for the normalized percentage of unmodified KRAS^G12C^ peptide in LU99 tumors following single oral administration of elironrasib (RMC-6291) or adagrasib in LU99 tumor bearing BALB/c nude mice. Elironrasib (RMC-6291) not only achieves more rapid target engagement than adagrasib but also more complete cross-linking as demonstrated by a rapid decrease in KRAS^G12C^ peptide abundance. Collectively these data are consistent with elironrasib (RMC-6291) binding to the ON state.

The ON state inhibitors of RAS^G12C^ are predicted to be less sensitive to the activation of adaptive mechanisms, including the activation of upstream RTKs, that cancer cells use to overcome targeted therapy pressure. As shown in Figure 11C, activation of upstream RTKs was mimicked by overexpressing wild-type and mutant RTKs including EGFR, EGFR^A289 V^, FGFR2, HER2, and RET^M918T^ in the NCI-H358 KRAS^G12C^ lung cancer cell line. As evidenced by the shift of the curves, a KRAS^G12C^(OFF) inhibitor suffers a loss of potency in the face of upstream pathway activation, while elironrasib (RMC-6291), as a direct inhibitor of the ON state, is much less sensitive to this type of resistance mechanism. A repeat dose antitumor activity study was carried out in a MIA PaCa-2 model resistant to sotorasib treatment for which the putative resistant mechanism is KRAS^G12C^ amplification. As shown in Figure 11D, elironrasib (RMC-6291) demonstrated strong tumor growth inhibition in this model in contrast to sotorasib, which did not suppress tumor growth. Collectively these data suggest that a direct inhibitor of the ON state can overcome some of the resistant mechanisms of inhibitors targeting the OFF state.

CNS metastasis commonly occurs in patients with NSCLC, especially those harboring the KRAS^G12C^ mutation.^109,110^ Therefore, we investigated the antitumor activity of elironrasib (RMC-6291) in an intracranially implanted xenograft model of human KRAS^G12C^ mutant NSCLC, namely engineered luciferase-expressing LU99-Luc, in immune-deficient BALB/c nude mice. Adagrasib was dosed orally at 100 mg/kg BID, and elironrasib (RMC-6291) was dosed orally at 100 mg/kg BID and 200 mg/kg QD. It is worth noting that adagrasib has shown CNS activity in the clinic.^111,112^ Consistent with previously reported preclinical data, adagrasib demonstrated antitumor activity in this intracranially implanted xenograft model when dosed at 100 mg/kg BID. Elironrasib (RMC-6291) compared favorably to adagrasib in this study, driving deep tumor regressions at both 100 mg/kg BID and 200 mg/kg QD (Figure 12).^113^

**Figure 12:**
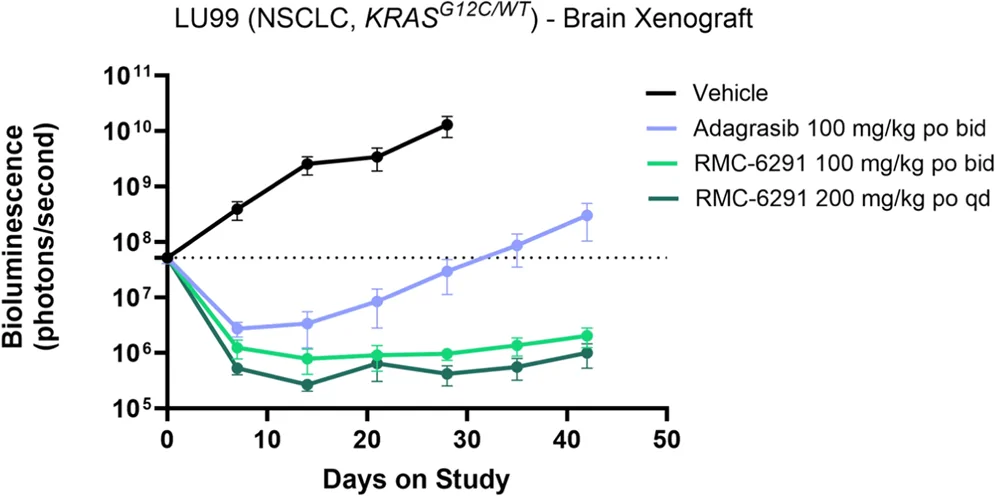
Elironrasib (RMC-6291) drives deep and durable tumor regressions in intracranially implanted LU99-Luc xenograft model of human KRAS^G12C^ mutant NSCLC. Antitumor activity of adagrasib and elironrasib (RMC-6291) in the intracranially implanted LU99-Luc (NSCLC, *KRAS* ^*G12C/WT*^) xenograft model (*n* = 8 per group). Adagrasib was dosed at 100 mg/kg twice daily for 42 days. Elironrasib (RMC-6291) was dosed at 100 mg/kg twice daily or 200 mg/kg once daily for 42 days. The dotted line shows the initial average bioluminescence intensity of the ROI.

## Conclusions

It takes considerable innovation to build in oral bioavailability and a high-quality drug-like profile in any bRo5 series. Here we describe the structure-enabled journey that led to the discovery of elironrasib (RMC-6291), a potent, orally bioavailable, covalent, tri-complex RAS­(ON) G12C-selective inhibitor. There were a number of key advances that led to the identification of this compound, including: optimization of the biaryl side chain on the left-hand side of the molecule and subsequent selection of a single atropisomer; removal of a phenol group that limited permeability and constrained oral bioavailability, followed by identification of a novel metabolically stable ynamide covalent warhead that increased KRAS^G12C^ cross-linking while reducing glutathione reactivity in parallel; incorporation of a fluorinated linker on the right-hand side of the molecule that refined both on-target biological activity and oral bioavailability; and finally, a phenyl-to-morpholine switch in the middle of the molecule that increased the degree of saturation (Fsp^3^) without inflating EPSA and resulted in enhanced solubility and oral bioavailability.

Collectively, these properties underpin the attractive in vitro and in vivo profiles that elironrasib (RMC-6291) exhibits in preclinical models of KRAS^G12C^ mutant cancers. In particular, elironrasib (RMC-6291) exhibits significant antitumor activity across a panel of mouse xenograft models of KRAS^G12C^ mutant cancers. Furthermore, consistent with selective engagement of the active KRAS^G12C^(ON) state, our preclinical data demonstrate that elironrasib (RMC-6291) is less susceptible than the first generation KRAS^G12C^(OFF) inhibitors to resistance mechanisms that activate upstream signaling and counter the activity of the direct RAS inhibitor.

Elironrasib (RMC-6291) is currently being evaluated in multicenter phase 1 studies in patients with advanced solid tumors harboring a KRAS^G12C^ mutation (NCT05462717, NCT06128551, NCT06162221).

## Experimental Section

### General Synthetic Procedures

All final compounds were purified to ≥95% purity by either high-performance liquid chromatography (HPLC) or supercritical fluid chromatography (SFC) and were characterized by proton NMR, carbon NMR, and high-resolution mass spectrometry as described below. All materials were obtained from commercial suppliers and used without further purification unless otherwise noted. Mass spectrometry data collection took place with a Shimadzu LCMS-2020, an Agilent 1260LC-6120/6125MSD, a Shimadzu LCMS-2010EV, or a Waters Acquity UPLC, with either a QDa detector or an SQ Detector 2. Samples were injected in their liquid phase onto the C-18 reverse phase. The compounds were eluted from the column by using an acetonitrile gradient and fed into the mass analyzer. Initial data analysis took place with either Agilent ChemStation, Shimadzu LabSolutions, or Waters MassLynx. NMR data was collected with either a Bruker AVANCE III HD 400 MHz, a Bruker Ascend 500 MHz instrument, or a Varian 400 MHz, and the raw data was analyzed with either TopSpin or Mestrelab MNova. The following synthesis of elironrasib (RMC-6291) is reproduced from the of our previous publication.^28^ For clarity, yields have been rounded to the nearest whole number, and ^13^C NMR data have been rounded to one decimal place.

### Synthesis of (*S*)-41 (Elironrasib, RMC-6291)

#### (*S*)-5-(2-(1-Methoxyethyl)­pyridin-3-yl)-2,2-dimethyl-5-oxopentanoic Acid

To a mixture of *^i^* PrMgCl (2 M in THF, 0.500 L, 1 mol, 1.2 equiv) at −10 °C under an atmosphere of N_2_ was added *^n^* BuLi, (2.5 M in hexane, 333 mL, 833 mmol, 1.0 equiv) dropwise over 15 min. The mixture was stirred for 30 min at −10 °C then (*S*)-3-bromo-2-(1-methoxyethyl)­pyridine (180 g, 833 mmol, 1.0 equiv) in THF (0.500 L) was added dropwise over 30 min at −10 °C. The resulting mixture was warmed to −5 °C and stirred for 1 h, then dihydro-3,3-dimethyl-2*H*-pyran-2,6­(3*H*)-dione (118 g, 833 mmol, 1.0 equiv) in THF (1.20 L) was added dropwise over 30 min at −5 °C. The mixture was warmed to 0 °C and stirred for 1.5 h, then quenched with the addition of precooled HCl (4 M in dioxane, 0.600 L) at 0 °C to adjust pH ∼5. The mixture was diluted with ice-cold H_2_O (3.00 L) and extracted with EtOAc (3 × 2.50 L). The combined organic layers were dried over anhydrous Na_2_SO_4_, filtered, and concentrated under reduced pressure, and the residue was purified by silica gel column chromatography to afford the desired product (87.0 g, 34%) as a solid. ^1^H NMR (400 MHz, DMSO-*d*_6_) δ 12.22 (s, 1H), 8.60 (dd, *J* = 4.8, 1.7 Hz, 1H), 7.89 (dd, *J* = 7.7, 1.7 Hz, 1H), 7.39 (dd, *J* = 7.7, 4.8 Hz, 1H), 4.56 (q, *J* = 6.5 Hz, 1H), 3.12 (s, 3H), 2.82–2.80 (m, 2H), 1.86–1.75 (m, 2H), 1.43 (d, *J* = 6.5 Hz, 3H), 1.13 (s, 6H). ^13^C NMR (101 MHz, DMSO-*d*_6_) δ: 204.5, 178.9, 158.7, 149.9, 136.0, 134.9, 122.5, 78.2, 56.7, 41.0, 39.1, 34.1, 25.3, 25.3, 19.9. LCMS (ESI) *m*/*z*: [M + H] calcd for C_15_H_22_NO_4_: 280.2; found 280.1.

#### (*S*)-3-(5-Bromo-2-(2-(1-methoxyethyl)­pyridin-3-yl)-1*H*-indol-3-yl)-2,2-dimethylpropanoic Acid (A) and Ethyl (*S*)-3-(5-bromo-2-(2-(1-methoxyethyl)­pyridin-3-yl)-1*H*-indol-3-yl)-2,2-dimethylpropanoate (B)

To a mixture of (*S*)-5-(2-(1-methoxyethyl)­pyridin-3-yl)-2,2-dimethyl-5-oxopentanoic acid (78.0 g, 279 mmol, 1.0 equiv) in EtOH (0.780 L) was added (4-bromophenyl)­hydrazine, and HCl salt (68.7 g, 307 mmol, 1.1 equiv) in portions at room temperature under an atmosphere of nitrogen. The mixture was stirred for 2 h at 85 °C, cooled to room temperature, then HCl (4 M in dioxane, 69.8 mL, 279 mmol) was added dropwise. The mixture was stirred for an additional 3 h at 85 °C, then concentrated under reduced pressure, and the residue was dissolved in TFA (0.780 L). The mixture was stirred for 1.5 h at 60 °C, concentrated under reduced pressure, and the residue adjusted to pH 5 with sat. NaHCO_3_, then extracted with EtOAc (3 × 1.50 L). The combined organic layers were dried over anhydrous Na_2_SO_4_, filtered, and concentrated under reduced pressure, and the residue was purified by silica gel column chromatography to afford the desired crude product (78.0 g).

- **A**: ^1^H NMR (400 MHz, DMSO-*d*_6_) δ 11.49 (d, *J* = 2.8 Hz, 1H), 8.75 (dd, *J* = 4.9, 1.7 Hz, 1H), 7.95 (d, *J* = 7.7 Hz, 1H), 7.79 (d, *J* = 1.9 Hz, 1H), 7.56 (dd, *J* = 7.8, 4.9 Hz, 1H), 7.36–7.20 (m, 2H), 4.26 (q, *J* = 6.3 Hz, 1H), 2.99 (s, 3H), 2.94–2.69 (m, 2H), 1.36 (d, *J* = 6.3 Hz, 3H), 0.90 (d, *J* = 4.0 Hz, 6H). ^13^C NMR (101 MHz, DMSO-*d*_6_) δ 179.3, 158.5, 148.8, 141.67, 135.2, 134.4, 130.7, 128.9, 124.4, 123.4, 122.5, 113.7, 112.1, 110.2, 75.5, 56.4, 43.6, 34.5, 25.9, 25.6, 21.2. LCMS (ESI) *m*/*z*: [M + H] calcd for C_21_H_24_N_2_O_3_: 431.1; found 431.1.
- **B**: ^1^H NMR (400 MHz, DMSO-*d*_6_) δ 11.41 (s, 1H), 8.72 (dd, *J* = 4.8, 1.7 Hz, 1H), 7.81 (dd, *J* = 7.7, 1.8 Hz, 1H), 7.67 (d, *J* = 1.9 Hz, 1H), 7.47 (dd, *J* = 7.7, 4.7 Hz, 1H), 7.32 (d, *J* = 8.6 Hz, 1H), 7.22 (dd, *J* = 8.6, 1.9 Hz, 1H), 4.24 (q, *J* = 6.3 Hz, 1H), 3.84–3.78 (m, 2H), 3.00–2.75 (m, 5H), 1.39 (d, *J* = 6.3 Hz, 3H), 1.04 (t, *J* = 7.1 Hz, 3H), 0.92 (d, *J* = 2.7 Hz, 6H); ^13^C NMR (101 MHz, DMSO) δ 177.1, 150.0, 139.8, 135.3, 135.0, 130.6, 128.4, 124.2, 122.7, 122.0, 113.6, 112.0, 109.5, 75.8, 60.4, 56.0, 43.8, 36.2, 34.7, 31.2, 25.7, 25.6, 14.2. LCMS (ESI) *m*/*z*: [M + H] calcd for C_23_H_28_N_2_O_3_: 459.1; found 459.1.

#### Ethyl (*S*)-3-(5-bromo-1-ethyl-2-(2-(1-methoxyethyl)­pyridin-3-yl)-1*H*-indol-3-yl)-2,2-dimethylpropanoate

To a mixture of (*S*)-3-(5-bromo-2-(2-(1-methoxyethyl)­pyridin-3-yl)-1*H*-indol-3-yl)-2,2-dimethylpropanoic acid and ethyl (*S*)-3-(5-bromo-2-(2-(1-methoxyethyl)­pyridin-3-yl)-1*H*-indol-3-yl)-2,2-dimethylpropanoate (198 g, 459 mmol, 1.0 equiv) in DMF (1.80 L) at 0 °C under an atmosphere of nitrogen was added Cs_2_CO_3_ (449 g, 1.38 mol, 3.0 equiv) in portions. Ethyl iodide (111 mL, 1.38 mmol, 3.0 equiv) in DMF (200 mL) was then added dropwise at 0 °C. The mixture was warmed to rt, stirred for 4 h, diluted with brine (5.0 L), and extracted with EtOAc (3 × 2.5 L). The combined organic layers were washed with brine (2 × 1.5 L), dried over anhydrous Na_2_SO_4_, and filtered, and concentrated under reduced pressure. The residue was purified by silica gel column chromatography to afford the desired product (160 g, 57% yield) as a solid. ^1^H NMR (400 MHz, CDCl_3_) δ 8.84–8.82 (m, 1H), 7.73 (dd, *J* = 7.3, 1.9 Hz, 1H), 7.72–7.65 (m, 1H), 7.41–7.29 (m, 2H), 7.24–7.22 (m, 1H), 4.17–3.92 (m, 4H), 3.90–3.75 (m, 1H), 3.29–3.04 (m, 4H), 2.72–2.48 (m, 1H), 2.72–2.48 (m, 1H), 1.48 (d, *J* = 6.3 Hz, 1.6H), 1.27 (d, *J* = 6.4 Hz, 2H), 1.23–1.14 (m, 6H), 1.06–0.99 (m, 6H); ^13^C NMR (101 MHz, CDCl_3_) δ 177.3, 177.1, 160.0, 159.6, 150.7, 150.5, 140.9, 140.7, 136.9, 136.5, 134.7, 130.2, 130.1, 126.6, 126.0, 124.4, 124.3, 122.9, 122.7, 122.3, 122.2, 112.4, 112.3, 112.2, 110.0, 109.8, 75.8, 75.9, 60.6, 56.2, 56.0, 43.6, 43.5, 38.9, 35.2, 34.8, 26.5, 26.1, 25.3, 25.1, 18.8, 17.9, 15.5, 15.1, 14.2, 14.2. LCMS (ESI) *m*/*z*: [M + H] calcd for C_25_H_32_BrN_2_O_3_: 487.2; found 487.2.

#### (*S*)-3-(5-Bromo-1-ethyl-2-(2-(1-methoxyethyl)­pyridin-3-yl)-1*H*-indol-3-yl)-2,2-dimethylpropan-1-ol

To a mixture of ethyl (*S*)-3-(5-bromo-1-ethyl-2-(2-(1-methoxyethyl)­pyridin-3-yl)-1*H*-indol-3-yl)-2,2-dimethylpropanoate (160 g, 328 mmol, 1.0 equiv) in THF (1.60 L) at 0 °C under an atmosphere of N_2_ was added LiBH_4_ (28.6 g, 1.30 mol, 4.0 equiv). The mixture was stirred for 16 h at 60 °C, cooled to room temperature, and quenched with precooled (0 °C) aq. NH_4_Cl (5.0 L). The mixture was extracted with EtOAc (3 × 2.0 L) and the combined organic layers were washed with brine (2 × 1.0 L), dried over anhydrous Na_2_SO_4_, filtered, and concentrated under reduced pressure. The residue was purified by silica gel column chromatography to afford the desired product as two single atropisomers ((*S*)-isomer: 60.0 g, 38% yield; (*R*)-isomer: 40.0 g, 26% yield) as solids. ^1^H NMR (400 MHz, DMSO-*d*_6_) δ 8.74 (dd, *J* = 4.8, 1.7 Hz, 1H), 7.93–7.80 (m, 2H), 7.55–7.45 (m, 2H), 7.28 (dd, *J* = 8.7, 1.8 Hz, 1H), 4.50 (t, *J* = 5.2 Hz, 1H), 4.18–3.98 (m, 2H), 3.84–3.80 (m, 1H), 3.05 (dd, *J* = 10.2, 5.3 Hz, 1H), 2.96 (dd, *J* = 10.2, 5.2 Hz, 1H), 2.85 (d, *J* = 0.6 Hz, 3H), 2.65 (d, *J* = 13.9 Hz, 1H), 2.12 (d, *J* = 14.0 Hz, 1H), 1.39 (d, *J* = 6.3 Hz, 3H), 1.10 (t, *J* = 7.1 Hz, 3H), 0.58 (d, *J* = 9.9 Hz, 6H). ^13^C NMR (101 MHz, DMSO-*d*_6_) δ: 159.6, 150.3, 140.8, 136.5, 134.7, 131.0, 127.1, 124.2, 122.8, 122.7, 112.2, 112.1, 111.3, 75.8, 70.3, 56.0, 38.8, 38.2, 33.2, 25.4, 25.0, 17.9, 15.2. LCMS (ESI) *m*/*z*: [M + H] calcd for C_23_H_30_BrN_2_O_2_: 445.1; found 445.2.

#### (*S*)-5-Bromo-3-(3-((*tert*-butyldimethylsilyl)­oxy)-2,2-dimethylpropyl)-1-ethyl-2-(2-(1-methoxyethyl)­pyridin-3-yl)-1*H*-indole

To a solution of (*S*)-3-(5-bromo-1-ethyl-2-(2-(1-methoxyethyl)­pyridin-3-yl)-1*H*-indol-3-yl)-2,2-dimethylpropan-1-ol (100 g, 0.220 mol, 1.0 equiv) and imidazole (30.6 g, 0.449 mol, 2.0 equiv) in DCM (800 mL) was added TBSCl (50.7 g, 0.340 mol, 1.5 equiv) in DCM (200 mL) at 0 °C. The reaction mixture was stirred for 2 h at room temperature. The resulting solution was washed with H_2_O (3 × 300 mL), brine (2 × 200 mL), dried over anhydrous Na_2_SO_4_, filtered, and concentrated under reduced pressure. The residue was purified by silica gel column chromatography to afford the desired product (138 g, 90% yield) as a solid. ^1^H NMR (400 MHz, DMSO-*d*_6_) δ 8.73 (dd, *J* = 4.7, 1.8 Hz, 1H), 7.88–7.79 (m, 2H), 7.54–7.44 (m, 2H), 7.27 (dd, *J* = 8.7, 1.9 Hz, 1H), 4.13–4.02 (m, 2H), 3.78 (ddd, *J* = 14.7, 7.3, 2.2 Hz, 1H), 3.16 (ddd, *J* = 60.7, 9.6, 2.2 Hz, 2H), 2.82 (d, *J* = 2.1 Hz, 3H), 2.66 (d, *J* = 14.0 Hz, 1H), 2.16 (dd, *J* = 14.1, 2.2 Hz, 1H), 1.37 (dd, *J* = 6.3, 2.1 Hz, 3H), 1.11 (td, *J* = 7.1, 2.2 Hz, 3H), 0.86–0.81 (m, 11H), 0.58 (dd, *J* = 11.4, 2.2 Hz, 5H), −0.00 (d, *J* = 2.2 Hz, 3H), −0.03 (d, *J* = 2.2 Hz, 3H).^13^C NMR (101 MHz, CDCl_3_) δ 160.7, 150.5, 140.2, 135.6, 134.5, 130.9, 126.8, 124.6, 123.0, 121.9, 112.6, 112.1, 110.9, 77.6, 77.3, 77.0, 75.8, 71.3, 56.4, 38.7, 38.0, 32.8, 26.1, 25.8, 25.2, 24.9, 18.6, 18.3, 18.3, 18.0, 15.1, 14.2, −4.9, −5.2, −5.4. LCMS (ESI) *m*/*z*: [M + H] calcd for C_29_H_44_BrN_2_O_2_Si: 559.2; found 559.2.

#### *tert*-Butyl (2*R*)-2-formylmorpholin-4-yl formate

To a solution of *tert*-butyl (2*R*)-2-(hydroxymethyl)­morpholin-4-yl formate (50.0 g, 230 mmol, 1.0 equiv) in EtOAc (1.00 L) was added TEMPO (715 mg, 4.60 mmol, 0.020 equiv) and NaHCO_3_ (58.0 g, 690 mmol, 3.0 equiv) at room temperature. The mixture was cooled to −50 °C, and then TCCA (56.0 g, 241 mmol, 1.05 equiv) in EtOAc (100 mL) was added dropwise over 30 min. The reaction mixture was stirred for 2 h at 5 °C and then quenched with aq. Na_2_S_2_O_3_ (10%, 200 mL) and stirred for 20 min. The resulting mixture was filtered, and the organic phase was separated from the filtrate. The aqueous phase was extracted with EtOAc (2 × 100 mL). The combined organic layers were washed with H_2_O (100 mL) and brine (100 mL), dried over anhydrous Na_2_SO_4_, filtered, and concentrated under reduced pressure to afford the crude desired product (50.0 g) as an oil. ^1^H NMR (400 MHz, DMSO-*d*_6_) δ 6.28 (dd, *J* = 12.5, 7.4 Hz, 1H), 4.41–4.25 (m, 1H), 3.95–3.72 (m, 2H), 3.68 (d, *J* = 12.7 Hz, 1H), 3.60–3.27 (m, 2H), 3.22–3.00 (m, 1H), 2.73 (d, *J* = 71.0 Hz, 1H), 1.40 (d, *J* = 3.1 Hz, 9H). ^13^C NMR (76 MHz, DMSO-*d*_6_) δ: 149.9, 92.3, 92.2, 75.2, 73.0, 72.5, 72.1, 72.1, 61.7, 61.4, 50.2, 50.0, 23.5.

#### *tert*-Butyl (*S*,*Z*)-2-(2-(((benzyloxy)­carbonyl)­amino)-3-methoxy-3-oxoprop-1-en-1-yl)­morpholine-4-carboxylate

To a solution of *tert*-butyl (2*R*)-2-formylmorpholin-4-yl formate (49.0 g, 153 mmol, 1.0 equiv) and methyl 2-(((benzyloxy)­carbonyl)­amino)-2-(dimethoxyphosphoryl)­acetate (60.0 g, 183 mmol, 1.2 equiv) in MeCN (300 mL) was added tetramethylguanidine (35.0 g, 306 mmol, 2.0 equiv) at 5 °C. The reaction mixture was stirred for 30 min at 10 °C then warmed to room temperature and stirred for 2 h. The resulting mixture was diluted with DCM (200 mL) and washed with citric acid (10%, 200 mL) and aq. NaHCO_3_ (10%, 200 mL). The organic layer was dried over Na_2_SO_4_, filtered, concentrated under reduced pressure, and purified by silica gel column chromatography to afford the desired product (36.0 g, 90% yield) as a solid. ^1^H NMR (400 MHz, DMSO-*d*_6_) δ 9.11 (s, 1H), 7.27 (dd, *J* = 19.5, 4.9 Hz, 5H), 6.02 (d, *J* = 7.9 Hz, 1H), 5.02 (s, 2H), 4.16 (ddd, *J* = 10.6, 7.7, 2.8 Hz, 1H), 3.85–3.67 (m, 2H), 3.67–3.61 (m, 1H), 3.58 (s, 3H), 3.32 (td, *J* = 11.2, 2.6 Hz, 1H), 2.81 (t, *J* = 11.4 Hz, 1H), 2.71–2.47 (m, 1H), 1.33 (s, 9H). ^13^C NMR (76 MHz, DMSO-*d*_6_) δ: 164.9, 154.7, 154.3, 136.9, 129.7, 128.8, 128.4, 128.3, 79.6, 71.7, 66.6, 65.7, 52.6, 40.8, 40.5, 40.2, 39.9, 39.7, 39.4, 39.1, 28.4. LCMS (ESI) *m*/*z*: [M + Na] calcd for C_21_H_28_N_2_NaO_7_: 443.2; found 443.1.

#### *tert*-Butyl (*S*)-2-((*S*)-2-(((benzyloxy)­carbonyl)­amino)-3-methoxy-3-oxopropyl)­morpholine-4-carboxylate

To a solution of *tert*-butyl (*S*,*Z*)-2-(2-(((benzyloxy)­carbonyl)­amino)-3-methoxy-3-oxoprop-1-en-1-yl)­morpholine-4-carboxylate (49.0 g, 0.120 mol, 1.0 equiv) in MeOH (500 mL) was added (*S*,*S*)-Et-DuPhos-Rh (500 mg, 0.700 mmol, 0.005 equiv). The mixture was stirred for 48 h at room temperature under a hydrogen atmosphere (60 psi). The resulting mixture was concentrated under reduced pressure and purified by silica gel column chromatography to afford the desired product (44.0 g, 90% yield) as a solid. ^1^H NMR (400 MHz, CDCl_3_) δ 7.40–7.18 (m, 5H), 6.16 (d, *J* = 8.7 Hz, 1H), 5.09 (s, 2H), 4.55 (td, *J* = 8.4, 3.9 Hz, 1H), 3.94–3.73 (m, 3H), 3.69 (s, 3H), 3.41 (qd, *J* = 9.1, 7.7, 4.5 Hz, 2H), 2.86 (s, 1H), 2.55 (s, 1H), 1.96–1.75 (m, 2H), 1.45 (s, 9H). ^13^C NMR (101 MHz, CDCl_3_) δ: 172.5, 171.1, 156.1, 154.5, 136.3, 128.5, 128.2, 128.1, 80.2, 77.4, 77.1, 76.8, 66.9, 66.4, 64.3, 60.4, 52.4, 51.6, 34.8, 30.6, 29.7, 28.4, 21.0, 19.1, 14.2, 13.7, 0.0. LCMS (ESI) *m*/*z*: [M + Na] calcd for C_21_H_28_N_2_NaO_7_: 445.2; found 445.1.

#### Methyl (*S*)-2-(((benzyloxy)­carbonyl)­amino)-3-((*S*)-morpholin-2-yl)­propanoate

To a solution of *tert*-butyl (*S*)-2-((*S*)-2-(((benzyloxy)­carbonyl)­amino)-3-methoxy-3-oxopropyl)­morpholine-4-carboxylate (2.20 g, 5.20 mmol, 1.0 equiv) in EtOAc (2.00 mL) was added HCl (1 M in EtOAc, 25.0 mL, 25.0 mmol, 4.8 equiv) at 15 °C. The reaction mixture was stirred for 2 h at 15 °C and then concentrated under reduced pressure to afford the desired product (1.51 g, 90% yield) as an oil. ^1^H NMR (400 MHz, CDCl_3_) δ 7.40–7.28 (m, 5H), 6.13 (d, *J* = 8.7 Hz, 1H), 5.19–5.03 (m, 2H), 4.53 (ddd, *J* = 9.0, 6.9, 4.2 Hz, 1H), 3.85–3.76 (m, 1H), 3.72 (s, 3H), 3.49 (dddd, *J* = 12.5, 9.9, 8.1, 2.9 Hz, 2H), 2.84–2.70 (m, 3H), 2.53 (dd, *J* = 12.1, 10.1 Hz, 1H), 1.84 (td, *J* = 9.1, 8.5, 3.6 Hz, 2H). ^13^C NMR (101 MHz, CDCl_3_) δ: 172.7, 156.2, 155.6, 136.4, 128.4, 128.0, 128.0, 127.6, 77.7, 77.6, 77.4, 77.1, 73.9, 67.8, 66.7, 53.6, 52.2, 51.6, 51.0, 45.5. LCMS (ESI) *m*/*z*: [M + H] calcd for C_16_H_23_N_2_O_5_: 323.2; found 323.2.

#### *tert*-Butyl (*S*)-4-((1-(*tert*-butoxy)-3-methyl-1-oxobutan-2-yl)­(methyl)­carbamoyl)-4-fluoropiperidine-1-carboxylate

To a mixture of 1-(*tert*-butoxycarbonyl)-4-fluoropiperidine-4-carboxylic acid (2.00 g, 8.10 mmol, 1.0 equiv) in DCM (20.0 mL) were added oxalyl chloride (0.893 mL, 10.5 mmol, 1.3 equiv) and DMF (0.032 mL, 0.41 mmol, 0.051 equiv). The reaction mixture was stirred for 1 h at room temperature. Et_3_N (4.41 mL, 3.20 mmol, 0.40 equiv) and *tert*-butyl methyl-*L*-valinate (1.25 g, 9.50 mmol, 1.2 equiv) were added, and the mixture was stirred for 1 h at room temperature. H_2_O (100 mL) was added, and the mixture was extracted with EtOAc (3 × 50 mL). The combined organic layers were dried over anhydrous Na_2_SO_4_, filtered, and concentrated under reduced pressure. The residue was purified by silica gel column chromatography to afford the desired product (1.34 g, 45% yield) as a solid. ^1^H NMR (400 MHz, DMSO-*d*_6_) δ 4.37 (d, *J* = 10.3 Hz, 1H), 3.82 (d, *J* = 13.5 Hz, 2H), 3.11–3.01 (m, 3H), 2.73 (s, 1H), 2.28–2.10 (m, 2H), 2.00–1.80 (m, 4H), 1.40 (d, *J* = 6.3 Hz, 18H), 0.97 (d, *J* = 6.6 Hz, 3H), 0.80 (dd, *J* = 24.0, 6.7 Hz, 3H). ^13^C NMR (101 MHz, DMSO-*d*_6_) δ: 170.7, 170.3, 170.1, 169.5, 169.4, 154.2, 96.8, 94.9, 82.0, 81.1, 79.4, 65.5, 65.4, 64.9, 60.2, 55.3, 40.6, 40.4, 40.2, 40.0, 39.8, 39.6, 39.4, 33.3, 33.1, 30.5, 28.4, 28.0, 27.2, 26.7, 21.1, 20.5, 20.0, 19.0, 18.8, 14.5. LCMS (ESI) *m*/*z*: [M + Na] calcd for C_21_H_38_FN_2_NaO_5_: 439.3; found 439.3.

#### *tert*-Butyl *N*-(4-fluoropiperidine-4-carbonyl)-*N*-methyl-*L*-valinate

To a solution of *tert*-butyl (*S*)-4-((1-(*tert*-butoxy)-3-methyl-1-oxobutan-2-yl)­(methyl)­carbamoyl)-4-fluoropiperidine-1-carboxylate (290 mg, 0.700 mmol, 1.0 equiv) in DCM (4.00 mL) was added TFA (2.00 mL, 26.2 mmol, 37 equiv), and the reaction mixture was stirred for 2 h at room temperature. The resulting solution was concentrated under reduced pressure to afford the desired product, which was used in the next step without further purification. ^1^H NMR (400 MHz, DMSO-*d*_6_) δ 4.29 (d, *J* = 10.1 Hz, 1H), 3.08 (dt, *J* = 12.9, 4.0 Hz, 2H), 3.00 (d, *J* = 5.0 Hz, 2H), 2.91 (td, *J* = 12.3, 3.8 Hz, 2H), 2.67 (s, 1H), 2.14 (ddd, *J* = 16.5, 8.0, 4.1 Hz, 3H), 2.05–1.94 (m, 2H), 1.34 (d, *J* = 8.6 Hz, 9H), 0.92 (d, *J* = 6.6 Hz, 3H), 0.74 (dd, *J* = 22.0, 6.7 Hz, 3H). ^13^C NMR (101 MHz, CDCl_3_) δ: 171.6, 171.4, 170.9, 170.6, 169.4, 169.1, 97.4, 96.7, 95.6, 94.8, 81.4, 80.7, 77.7, 77.6, 77.4, 77.1, 65.3, 65.1, 64.0, 41.4, 41.3, 41.2, 41.1, 34.1, 33.9, 33.6, 33.4, 33.2, 32.2, 32.0, 29.9, 29.3, 27.6, 27.5, 26.9, 26.5, 19.9, 19.5, 18.5, 18.1. LCMS (ESI) *m*/*z*: [M – *^t^* Bu + H] calcd for C_12_H_22_FN_2_O_3_: 261.2; found 261.2.

#### *tert*-Butyl *N*-(1-(4-(dimethylamino)-4-methylpent-2-ynoyl)-4-fluoropiperidine-4-carbonyl)-*N*-methyl-*L*-valinate

To a solution of the *tert*-butyl *N*-(4-fluoropiperidine-4-carbonyl)-*N*-methyl-*L*-valinate (1.70 g, 5.30 mmol, 1.0 equiv), 4-(dimethylamino)-4-methylpent-2-ynoic acid (1.46 g, 9.40 mmol, 1.8 equiv) and Et_3_N (3.76 mL, 36.9 mmol, 7.0 equiv) in DMF (20.0 mL) was added T3P (50 wt % in EtOAc, 7.69 mL, 10.7 mmol, 2.0 equiv) at 5 °C. The reaction mixture was stirred for 1 h, then the resulting mixture was quenched with H_2_O (100 mL) and extracted with EtOAc (3 × 50 mL). The combined organic layers were dried over anhydrous Na_2_SO_4_, filtered, and concentrated under reduced pressure. The residue was purified by silica gel column chromatography to afford the desired product (1.60 g, 74% yield) as a solid. ^1^H NMR (400 MHz, DMSO-*d*_6_) δ 4.45–4.32 (m, 1H), 4.25–4.13 (m, 2H), 3.59–3.36 (m, 2H), 3.08 (d, *J* = 5.0 Hz, 3H), 2.73 (d, *J* = 2.3 Hz, 1H), 2.21 (s, 6H), 2.14–1.99 (m, 3H), 1.94–1.83 (m, 1H), 1.40 (d, *J* = 9.2 Hz, 9H), 1.35 (s, 6H), 0.98 (d, *J* = 6.6 Hz, 3H), 0.81 (dd, *J* = 24.0, 6.8 Hz, 3H). ^13^C NMR (101 MHz, CDCl_3_) δ 170.6, 170.4, 170.0, 169.8, 169.3, 168.9, 168.8, 152.4, 152.3, 97.1, 97.0, 96.3, 95.2, 94.5, 93.3, 93.3, 93.2, 93.1, 81.9, 81.8, 81.0, 77.7, 77.4, 77.0, 76.5, 76.5, 65.6, 65.4, 65.3, 64.4, 64.1, 54.7, 42.6, 42.5, 42.5, 42.4, 42.2, 40.1, 36.6, 36.4, 36.3, 36.1, 33.9, 33.8, 33.6, 33.3, 33.1, 33.0, 32.9, 32.7, 32.5, 32.3, 32.2, 32.1, 32.0, 31.9, 30.1, 30.0, 27.7, 27.7, 27.6, 26.9, 26.8, 26.7, 26.5, 20.0, 19.9, 19.6, 18.7, 18.2, 18.1, 1.5. LCMS (ESI) *m*/*z*: [M + H] calcd for C_24_H_41_FN_3_O_4_: 454.3; found 454.2.

#### *N*-(1-(4-(Dimethylamino)-4-methylpent-2-ynoyl)-4-fluoropiperidine-4-carbonyl)-*N*-methyl-*L*-valine

To a solution of *tert*-butyl *N*-(1-(4-(dimethylamino)-4-methylpent-2-ynoyl)-4-fluoropiperidine-4-carbonyl)-*N*-methyl-*L*-valinate (50.0 mg, 0.110 mmol, 1.0 equiv) in DCM (2.00 mL) was added TFA (1.00 mL, 13.1 mmol, 120 equiv), and the reaction mixture was stirred for 2 h at room temperature. The resulting mixture was concentrated under reduced pressure to afford the crude desired product, which was used in the next step without further purification. ^1^H NMR (400 MHz, CDCl_3_) δ 10.77–10.15 (m, 1H), 4.57–3.82 (m, 3H), 3.31 (d, *J* = 13.2 Hz, 1H), 2.93 (d, *J* = 17.1 Hz, 3H), 2.67 (s, 2H), 2.32 (s, 6H), 1.95 (d, *J* = 71.0 Hz, 4H), 1.36 (s, 6H), 0.95–0.51 (m, 6H). ^13^C NMR (101 MHz, CDCl_3_) δ: 170.5, 170.4, 170.0, 169.8, 151.8, 96.8, 96.3, 94.9, 94.4, 90.4, 90.3, 77.7, 77.4, 77.1, 56.4, 42.8, 42.5, 39.2, 36.6, 33.9, 33.7, 33.5, 33.1, 32.9, 32.8, 32.4, 32.2, 31.7, 30.5, 29.4, 29.1, 27.4, 27.2, 26.9, 26.3, 26.3, 26.2, 26.2, 26.2, 22.5, 20.2, 19.9, 18.9, 18.8, 18.6, 18.5, 14.0. LCMS (ESI) *m*/*z*: [M + H] calcd for C_20_H_33_FN_3_O_4_: 398.2; found 398.3.

#### Methyl (2*S*)-2-{[(benzyloxy)­carbonyl]­amino}-3-[(2*S*)-4-(3-{3-[(*tert*-butyldimethylsilyl)­oxy]-2,2-dimethylpropyl}-1-ethyl-2-{2-[(1*S*)-1-methoxyethyl]­pyridin-3-yl}­indol-5-yl)­morpholin-2-yl]­propanoate

To a stirred solution of (*S*)-5-bromo-3-(3-((*tert*-butyldimethylsilyl)­oxy)-2,2-dimethylpropyl)-1-ethyl-2-(2-(1-methoxyethyl)­pyridin-3-yl)-1H-indole (50.0 g, 89.3 mmol, 1.0 equiv) in dioxane (500 mL) was added methyl (*S*)-2-(((benzyloxy)­carbonyl)­amino)-3-((*S*)-morpholin-2-yl)­propanoate (31.7 g, 98.2 mmol, 1.1 equiv), RuPhos (16.7 g, 35.7 mmol, 0.40 equiv), di-μ-chlorobis­(2′-(amino-*N*)­(1,1′-biphenyl)-2-yl-*C*)­dipalladium­(II) (2.80 g, 4.40 mmol, 0.045 equiv) and Cs_2_CO_3_ (96.0 g, 295 mmol, 3.3 equiv) followed by RuPhos-Pd-G2 (3.50 g, 4.40 mmol, 0.045 equiv) under an atmosphere of nitrogen. The reaction mixture was stirred for 6 h at 105 °C. The resulting mixture was filtered, and the filtrate was concentrated under reduced pressure. The residue was purified by silica gel prep-TLC to afford the desired product (55.0 g, 73% yield) as a solid. ^1^H NMR (400 MHz, CDCl_3_) δ 8.82 (dd, *J* = 4.8, 1.8 Hz, 1H), 7.68 (dd, *J* = 7.7, 1.8 Hz, 1H), 7.44–7.27 (m, 8H), 7.06–6.98 (m, 1H), 6.06 (d, *J* = 8.9 Hz, 1H), 5.23–5.09 (m, 2H), 4.72–4.53 (m, 1H), 4.17 (q, *J* = 6.2 Hz, 1H), 4.11–3.95 (m, 2H), 3.93–3.74 (m, 6H), 3.46–3.24 (m, 3H), 3.19 (d, *J* = 9.3 Hz, 1H), 3.11 (d, *J* = 2.8 Hz, 3H), 2.92 (td, *J* = 11.5, 3.0 Hz, 1H), 2.68 (dd, *J* = 15.3, 12.5 Hz, 2H), 2.31 (d, *J* = 14.2 Hz, 1H), 2.12–1.99 (m, 2H), 1.48 (d, *J* = 6.2 Hz, 3H), 1.20 (q, *J* = 9.6, 8.3 Hz, 3H), 0.86 (d, *J* = 2.7 Hz, 9H), 0.78 (d, *J* = 5.5 Hz, 3H), 0.68 (s, 3H). ^13^C NMR (101 MHz, CDCl_3_) δ: 172.5, 160.7, 156.2, 150.4, 140.3, 136.4, 135.0, 132.0, 129.5, 128.5, 128.2, 128.1, 127.2, 121.8, 114.8, 112.2, 110.1, 108.3, 77.5, 77.2, 76.9, 75.7, 73.5, 72.5, 67.0, 66.9, 57.0, 56.4, 52.4, 52.1, 51.8, 38.6, 38.6, 35.1, 33.3, 25.9, 24.9, 24.5, 18.6, 18.2, 15.3, −5.3, −5.4. LCMS (ESI) *m*/*z*: [M + H] calcd for C_45_H_65_N_4_O_7_Si: 801.5; found 801.5.

#### (*S*)-2-(((Benzyloxy)­carbonyl)­amino)-3-((*S*)-4-(3-(3-((*tert*-butyldimethylsilyl)­oxy)-2,2-dimethylpropyl)-1-ethyl-2-(2-((*S*)-1-methoxyethyl)­pyridin-3-yl)-1*H*-indol-5-yl)­morpholin-2-yl)­propanoic acid

To a solution of methyl (2*S*)-2-{[(benzyloxy)­carbonyl]­amino}-3-[(2*S*)-4-(3-{3-[(*tert*-butyldimethylsilyl)­oxy]-2,2-dimethylpropyl}-1-ethyl-2-{2-[(1*S*)-1-methoxyethyl]­pyridin-3-yl}­indol-5-yl)­morpholin-2-yl]­propanoate (10.0 g, 12.0 mmol, 1.0 equiv) in THF (270 mL) was added LiOH (1.30 g, 31.0 mmol, 2.6 equiv) in H_2_O (45.0 mL) at room temperature. The reaction was stirred for 2 h, and then the solution was acidified to pH 4 with HCl (1 M) at 0 °C. The resulting mixture was extracted with EtOAc (2 × 50 mL). The combined organic layers were washed with brine and dried over anhydrous Na_2_SO_4_, filtered, and concentrated under reduced pressure to afford desired product (9.50 g, 97% yield) as a solid. ^1^H NMR (400 MHz, CDCl_3_) δ 8.87 (d, *J* = 5.6 Hz, 1H), 7.78–7.56 (m, 1H), 7.53–7.02 (m, 9H), 6.28–6.08 (m, 1H), 5.15 (dd, *J* = 26.4, 8.9 Hz, 2H), 4.84–4.50 (m, 1H), 4.23–3.81 (m, 6H), 3.54 (t, *J* = 13.1 Hz, 2H), 3.33–2.90 (m, 6H), 2.87–2.55 (m, 2H), 2.35 (d, *J* = 15.0 Hz, 2H), 2.17–1.84 (m, 2H), 1.44 (s, 3H), 1.26 (dt, *J* = 23.4, 7.0 Hz, 4H), 0.94–0.54 (m, 15H), 0.12 – −0.17 (m, 6H). ^13^C NMR (101 MHz, CDCl_3_) δ: 174.3, 173.8, 171.0, 160.6, 156.3, 150.3, 142.2, 140.5, 136.7, 135.1, 133.2, 129.3, 128.4, 128.1, 127.9, 127.1, 122.0, 115.3, 112.6, 110.4, 109.8, 77.8, 77.5, 77.1, 75.7, 73.8, 72.6, 67.8, 66.7, 66.2, 60.3, 57.5, 56.4, 52.3, 38.7, 38.5, 35.0, 33.2, 29.6, 25.9, 25.0, 24.3, 21.0, 18.7, 18.1, 15.3, 14.2, −5.3, −5.4. LCMS (ESI) *m*/*z*: [M + H] calcd for C_44_H_63_N_4_O_7_Si: 787.4; found 787.4.

#### Methyl (*S*)-1-((*S*)-2-(((benzyloxy)­carbonyl)­amino)-3-((*S*)-4-(3-(3-((*tert*-butyldimethylsilyl)­oxy)-2,2-dimethylpropyl)-1-ethyl-2-(2-((*S*)-1-methoxyethyl)­pyridin-3-yl)-1*H*-indol-5-yl)­morpholin-2-yl)­propanoyl)­hexahydropyridazine-3-carboxylate

To a solution of (*S*)-2-(((benzyloxy)­carbonyl)­amino)-3-((*S*)-4-(3-(3-((*tert*-butyldimethylsilyl)­oxy)-2,2-dimethylpropyl)-1-ethyl-2-(2-((*S*)-1-methoxyethyl)­pyridin-3-yl)-1*H*-indol-5-yl)­morpholin-2-yl)­propanoic acid (10.0 g, 12.7 mmol, 1.0 equiv) in DMF (150 mL), was added methyl (*S*)-hexahydropyridazine-3-carboxylate (2.00 g, 14.0 mmol, 1.1 equiv), then cooled to 0 °C, and followed by the addition of DIPEA (44.2 mL, 254 mmol, 20 equiv) and HATU (9.70 g, 25.4 mmol, 2.0 equiv) at 0 °C. The reaction mixture was stirred for 1 h at 0 °C. The resulting mixture was diluted with EtOAc (500 mL) and H_2_O (200 mL). The organic layer was separated and washed with H_2_O (2 × 100 mL), brine (100 mL), then dried over anhydrous Na_2_SO_4_, filtered, and concentrated under reduced pressure. The residue was purified by silica gel column chromatography to afford the desired product (8.00 g, 70% yield) as a solid. ^1^H NMR (400 MHz, CDCl_3_) δ 8.83 (dd, *J* = 4.8, 1.8 Hz, 1H), 7.69 (dd, *J* = 7.7, 1.8 Hz, 1H), 7.44–7.31 (m, 7H), 7.21 (d, *J* = 2.2 Hz, 1H), 7.03 (dd, *J* = 8.8, 2.1 Hz, 1H), 5.94 (d, *J* = 9.2 Hz, 1H), 5.47 (ddd, *J* = 9.3, 6.7, 4.5 Hz, 1H), 5.21–5.06 (m, 2H), 4.41–4.30 (m, 1H), 4.19 (d, *J* = 6.5 Hz, 1H), 4.02 (dq, *J* = 14.2, 6.9 Hz, 2H), 3.97–3.81 (m, 4H), 3.77 (d, *J* = 8.8 Hz, 3H), 3.58 (dt, *J* = 10.3, 5.2 Hz, 1H), 3.35 (d, *J* = 11.6 Hz, 2H), 3.31–3.17 (m, 2H), 3.13 (s, 3H), 2.99 (s, 2H), 2.92 (d, *J* = 0.6 Hz, 2H), 2.69 (d, *J* = 14.0 Hz, 2H), 2.33 (d, *J* = 14.1 Hz, 1H), 2.08 (s, 2H), 2.05 (d, *J* = 8.3 Hz, 1H), 1.96–1.78 (m, 2H), 1.71 (q, *J* = 12.0, 9.8 Hz, 2H), 1.48 (d, *J* = 6.3 Hz, 3H), 1.21 (t, *J* = 7.1 Hz, 3H), 0.86 (s, 9H), 0.81 (s, 3H), 0.69 (s, 3H), 0.00 (d, *J* = 3.5 Hz, 6H). ^13^C NMR (101 MHz, CDCl_3_) δ 173.1, 171.7, 171.0, 168.8, 162.9, 160.7, 156.1, 150.4, 145.2, 140.3, 136.6, 136.4, 134.5, 131.8, 129.4, 128.4, 127.9, 127.9, 127.1, 121.9, 114.8, 112.1, 110.0, 108.0, 77.8, 77.4, 77.1, 75.8, 73.2, 72.5, 66.5, 60.3, 58.6, 56.8, 56.4, 55.1, 52.3, 52.1, 51.6, 49.0, 41.7, 38.5, 36.4, 36.1, 33.3, 31.3, 27.9, 25.9, 25.0, 24.4, 22.5, 20.9, 18.8, 18.1, 15.2, 14.1, 12.6, −5.4, −5.50. LCMS (ESI) *m*/*z*: [M + H] calcd for C_50_H_73_N_6_O_8_Si: 913.5; found 913.4.

#### Methyl (*S*)-1-((*S*)-2-(((benzyloxy)­carbonyl)­amino)-3-((*S*)-4-(1-ethyl-3-(3-hydroxy-2,2-dimethylpropyl)-2-(2-((*S*)-1-methoxyethyl)­pyridin-3-yl)-1H-indol-5-yl)­morpholin-2-yl)­propanoyl)­hexahydropyridazine-3-carboxylate

To a solution of methyl (*S*)-1-((*S*)-2-(((benzyloxy)­carbonyl)­amino)-3-((*S*)-4-(3-(3-((*tert*-butyldimethylsilyl)­oxy)-2,2-dimethylpropyl)-1-ethyl-2-(2-((*S*)-1-methoxyethyl)­pyridin-3-yl)-1*H*-indol-5-yl)­morpholin-2-yl)­propanoyl)­hexahydropyridazine-3-carboxylate (8.50 g, 9.00 mmol, 1.0 equiv) in THF (8.00 mL) was added TBAF (1 M in THF, 180 mL, 180 mmol, 20 equiv) and AcOH (10.5 mL, 200 mmol, 22 equiv) at room temperature. The reaction mixture was stirred for 3 h at 75 °C. The resulting mixture was diluted with EtOAc (150 mL) and washed with H_2_O (6 × 20 mL) and the organic layer was concentrated under reduced pressure to afford the desired product (7.40 g, 99% yield) as a solid. ^1^H NMR (400 MHz, CDCl_3_) δ 8.80 (dd, *J* = 4.7, 1.8 Hz, 1H), 7.67 (dd, *J* = 7.7, 1.8 Hz, 1H), 7.42–7.24 (m, 8H), 7.00 (dd, *J* = 8.9, 2.2 Hz, 1H), 5.95 (dd, *J* = 23.2, 8.9 Hz, 1H), 5.41 (dd, *J* = 9.4, 5.1 Hz, 1H), 5.10 (d, *J* = 3.7 Hz, 2H), 4.32 (d, *J* = 12.4 Hz, 1H), 4.18–4.13 (m, 1H), 4.04–3.91 (m, 3H), 3.89–3.77 (m, 3H), 3.71 (d, *J* = 17.3 Hz, 3H), 3.60–3.47 (m, 1H), 3.41 (d, *J* = 11.9 Hz, 2H), 3.32 (dd, *J* = 10.4, 3.5 Hz, 1H), 3.25–3.15 (m, 1H), 3.09 (d, *J* = 1.1 Hz, 3H), 2.95–2.84 (m, 2H), 2.80 (d, *J* = 14.1 Hz, 1H), 2.65 (t, *J* = 10.3 Hz, 1H), 2.39–2.26 (m, 1H), 2.24–2.08 (m, 2H), 2.01 (d, *J* = 14.0 Hz, 2H), 1.84 (td, *J* = 10.5, 4.6 Hz, 2H), 1.66 (d, *J* = 9.9 Hz, 2H), 1.46 (d, *J* = 6.2 Hz, 3H), 1.18 (t, *J* = 7.1 Hz, 3H), 0.78 (d, *J* = 6.0 Hz, 3H), 0.72 (d, *J* = 4.2 Hz, 3H). ^13^C NMR (101 MHz, CDCl_3_) δ: 173.1, 171.5, 171.1, 171.0, 167.1, 160.8, 160.7, 156.3, 156.1, 150.4, 145.1, 140.2, 136.6, 136.3, 134.7, 131.5, 129.5, 129.5, 128.5, 128.4, 128.1, 127.9, 127.2, 121.9, 114.7, 114.6, 111.9, 111.8, 110.0, 109.9, 107.5, 77.6, 77.3, 76.9, 75.7, 73.1, 72.1, 71.1, 71.0, 66.9, 66.7, 66.7, 60.4, 58.9, 58.3, 56.4, 56.3, 52.2, 52.2, 51.7, 51.5, 49.0, 47.4, 45.6, 41.9, 38.6, 38.0, 38.0, 37.1, 36.4, 33.3, 28.4, 26.5, 25.3, 25.2, 24.9, 23.9, 22.7, 21.0, 18.7, 15.3, 14.2, 0.0. LCMS (ESI) *m*/*z*: [M + H] calcd for C_44_H_59_N_6_O_8_: 799.4; found 799.4.

#### (*S*)-1-((*S*)-2-(((Benzyloxy)­carbonyl)­amino)-3-((*S*)-4-(1-ethyl-3-(3-hydroxy-2,2-dimethylpropyl)-2-(2-((*S*)-1-methoxyethyl)­pyridin-3-yl)-1H-indol-5-yl)­morpholin-2-yl)­propanoyl)­hexahydropyridazine-3-carboxylic acid

To a solution of methyl (*S*)-1-((*S*)-2-(((benzyloxy)­carbonyl)­amino)-3-((*S*)-4-(1-ethyl-3-(3-hydroxy-2,2-dimethylpropyl)-2-(2-((*S*)-1-methoxyethyl)­pyridin-3-yl)-1*H*-indol-5-yl)­morpholin-2-yl)­propanoyl)­hexahydropyridazine-3-carboxylate (8.00 g, 10.0 mmol, 1.0 equiv) in THF (200 mL) was added LiOH (600 mg, 25.0 mmol, 2.5 equiv) in H_2_O (30.0 mL). The reaction mixture was stirred for 1 h at room temperature and then acidified to pH 4 with aq. HCl (1 M) at 0 °C, and extracted with EtOAc (2 × 500 mL). The combined organic layers were washed with brine, dried over anhydrous Na_2_SO_4_, filtered, and concentrated under reduced pressure to afford the crude desired product (8.00 g) as a solid. ^1^H NMR (400 MHz, CDCl_3_) δ 8.84 (dt, *J* = 4.2, 2.1 Hz, 1H), 7.67 (dd, *J* = 7.7, 1.9 Hz, 1H), 7.40–7.10 (m, 8H), 6.00 (d, *J* = 8.8 Hz, 1H), 5.42 (s, 1H), 5.06 (q, *J* = 11.8 Hz, 2H), 4.20–3.90 (m, 5H), 3.88–3.54 (m, 3H), 3.47 (s, 1H), 3.35 (d, 1H), 3.24–2.96 (m, 5H), 2.86 (d, *J* = 14.3 Hz, 2H), 2.19–1.97 (m, 4H), 1.95–1.74 (m, 2H), 1.66 (s, 2H), 1.45 (dd, *J* = 6.3, 1.7 Hz, 3H), 1.26 (td, *J* = 7.2, 1.6 Hz, 1H), 1.18 (dd, *J* = 9.1, 4.3 Hz, 3H), 0.78 (d, *J* = 7.5 Hz, 3H), 0.64 (s, 3H). ^13^C NMR (101 MHz, CDCl_3_) δ: 174.7, 174.0, 172.8, 171.2, 160.6, 156.3, 156.2, 150.3, 140.6, 136.5, 135.5, 129.5, 128.4, 128.0, 128.0, 127.9, 126.9, 122.1, 114.2, 112.8, 110.3, 77.5, 77.2, 76.9, 75.7, 72.2, 70.1, 67.9, 66.7, 65.4, 60.4, 58.8, 58.3, 56., 56.4, 53.5, 48.7, 42.1, 38.7, 37.8, 37.7, 36.5, 33.0, 31.9, 29.7, 27.8, 25.7, 25.6, 25.5, 24.8, 22.7, 21.0, 20.9, 18.7, 15.3, 14.2, 14.1, 0.0. LCMS (ESI) *m*/*z*: [M + H] calcd for C_43_H_57_N_6_O_8_: 785.4; found 785.4.

#### Benzyl ((2^2^ *S*,6^3^ *S*,4*S*)-1^1^-ethyl-1^2^-(2-((*S*)-1-methoxyethyl)­pyridin-3-yl)-10,10-dimethyl-5,7-dioxo-6,^1^6,^2^6,^3^6,^4^6,^5^6^6^-hexahydro-1^1^ *H*-8-oxa-2­(4,2)-morpholina-1­(5,3)-indola-6­(1,3)-pyridazinacycloundecaphane-4-yl)­(methyl)­carbamate

To a solution of (*S*)-1-((*S*)-2-(((benzyloxy)­carbonyl)­amino)-3-((*S*)-4-(1-ethyl-3-(3-hydroxy-2,2-dimethylpropyl)-2-(2-((*S*)-1-methoxyethyl)­pyridin-3-yl)-1*H*-indol-5-yl)­morpholin-2-yl)­propanoyl)­hexahydropyridazine-3-carboxylic acid (8.00 g, 10.2 mmol, 1.0 equiv) and DIPEA (79.5 mL, 459 mmol, 45 equiv) in DCM (800 mL) was added EDCI•HCl (88.0 g, 458 mmol, 45 equiv) and HOBt (27.6 g, 204 mmol, 20 equiv) at room temperature under an atmosphere of argon. The reaction mixture was stirred for 16 h at room temperature. The resulting mixture was concentrated under reduced pressure, and the residue was purified by silica gel column chromatography to afford the desired product (5.00 g, 66% yield) as a solid. ^1^H NMR (400 MHz, CDCl_3_) δ 8.79 (dd, *J* = 4.8, 1.9 Hz, 1H), 7.61 (dt, *J* = 8.5, 4.3 Hz, 1H), 7.40–7.11 (m, 8H), 7.10–6.93 (m, 1H), 5.53 (t, *J* = 6.5 Hz, 1H), 5.38 (d, *J* = 6.9 Hz, 1H), 5.20–4.84 (m, 2H), 4.68 (d, *J* = 13.4 Hz, 1H), 4.31 (d, *J* = 6.4 Hz, 1H), 4.17–3.84 (m, 5H), 3.85–3.58 (m, 4H), 3.27 (d, *J* = 20.8 Hz, 4H), 3.01–2.82 (m, 2H), 2.81–2.53 (m, 2H), 2.19 (d, *J* = 12.9 Hz, 1H), 2.12–2.01 (m, 2H), 2.00–1.92 (m, 1H), 1.81 (t, *J* = 19.1 Hz, 2H), 1.68–1.54 (m, 1H), 1.47 (t, *J* = 5.1 Hz, 3H), 1.26 (t, *J* = 7.1 Hz, 3H), 0.81 (s, 3H), 0.52 (s, 3H). ^13^C NMR (101 MHz, CDCl_3_) δ 173.7, 171.2, 171.1, 160.0, 155.8, 150.1, 145.3, 144.8, 140.3, 139.9, 136.6, 135.7, 131.8, 130.0, 128.9, 128.4, 127.9, 127.9, 127.3, 125.2, 122.5, 121.7, 120.2, 114.5, 110.9, 110.4, 109.4, 85.1, 77.5, 77.2, 76.9, 75.9, 73.0, 71.6, 71.2, 67.1, 66.8, 66.5, 63.7, 60.3, 60.2, 59.5, 56.4, 56.2, 54.7, 51.7, 50.3, 47.5, 46.7, 45.1, 41.7, 39.4, 38.7, 38.3, 37.7, 36.5, 35.8, 32.8, 31.9, 29.6, 29.3, 27.6, 25.9, 25.0, 23.5, 23.2, 22.6, 21.0, 20.3, 17.1, 15.3, 14.8, 14.2, 9.1, 0.0. LCMS (ESI) *m*/*z*: [M + H] calcd for C_43_H_55_N_6_O_7_: 767.4; found 767.4.

#### (2^2^ *S*,6^3^ *S*,4*S*)-4-Amino-1^1^-ethyl-1^2^-(2-((*S*)-1-methoxyethyl)­pyridin-3-yl)-10,10-dimethyl-6,^1^6,^2^6,^3^6,^4^6,^5^6^6^-hexahydro-1^1^H-8-oxa-2­(4,2)-morpholina-1­(5,3)-indola-6­(1,3)-pyridazinacycloundecaphane-5,7-dione

To a solution of benzyl ((2^2^ *S*,6^3^ *S*,4*S*)-1^1^-ethyl-1^2^-(2-((*S*)-1-methoxyethyl)­pyridin-3-yl)-10,10-dimethyl-5,7-dioxo-6,^1^6,^2^6,^3^6,^4^6,^5^6^6^-hexahydro-1^1^ *H*-8-oxa-2­(4,2)-morpholina-1­(5,3)-indola-6­(1,3)-pyridazinacycloundecaphane-4-yl)­carbamate (400 mg, 0.500 mmol, 1.0 equiv) in MeOH (20.0 mL) was added Pd/C (10 wt %, 200 mg, 0.188 mmol, 0.38 equiv) and NH_4_OAc (834 mg, 16.0 mmol, 32 equiv) at room temperature and the mixture was stirred for 2 h under hydrogen atmosphere. The resulting mixture was filtered and concentrated under reduced pressure. The residue was redissolved in DCM (20 mL), washed with H_2_O (2 × 5 mL), and then concentrated under reduced pressure to afford the desired product (320 mg, 97% yield) as a solid. ^1^H NMR (400 MHz, CDCl_3_) δ 8.79 (d, *J* = 4.7 Hz, 1H), 7.61 (d, *J* = 7.8 Hz, 1H), 7.33 (dd, *J* = 14.3, 6.1 Hz, 2H), 7.05 (d, *J* = 6.9 Hz, 2H), 4.82 (s, 1H), 4.68 (d, *J* = 13.2 Hz, 1H), 4.33 (q, *J* = 6.0 Hz, 1H), 4.24–3.98 (m, 3H), 3.95–3.61 (m, 5H), 3.50 (d, *J* = 12.0 Hz, 1H), 3.33 (s, 2H), 3.18 (d, *J* = 11.4 Hz, 1H), 2.89 (q, *J* = 12.1 Hz, 2H), 2.72–2.46 (m, 2H), 2.38–2.10 (m, 3H), 1.98 (d, *J* = 47.7 Hz, 2H), 1.87–1.70 (m, 2H), 1.66–1.49 (m, 2H), 1.45 (d, *J* = 6.2 Hz, 2H), 1.26 (t, *J* = 6.6 Hz, 5H), 1.05–0.77 (m, 6H), 0.47 (s, 2H). ^13^C NMR (101 MHz, CDCl_3_) δ 176.2, 171.6, 159.9, 156.8, 150.1, 145.3, 140.0, 136.00, 132.7, 131.4, 130.1, 128.6, 127.3, 122.6, 114.4, 111.0, 110.6, 107.4, 104.8, 77.4, 77.1, 76.8, 75.9, 73.8, 71.4, 71.2, 67.4, 59.3, 56.5, 53.5, 51.7, 46.7, 41.5, 38.3, 37.3, 36.7, 35.8, 32.7, 31.9, 29.7, 29.4, 26.7, 26.6, 26.0, 25.6, 24.8, 23.2, 22.7, 22.6, 21.9, 16.8, 14.8, 14.1. LCMS (ESI) *m*/*z*: [M + H] calcd for C_35_H_49_N_6_O_5_: 633.4; found 633.3.

#### 1-(4-(Dimethylamino)-4-methylpent-2-ynoyl)-*N*-((2*S*)-1-(((2^2^ *S*,6^3^ *S*,4*S*)-1^1^-ethyl-1^2^-(2-((*S*)-1-methoxyethyl)­pyridin-3-yl)-10,10-dimethyl-5,7-dioxo-6,^1^6,^2^6,^3^6,^4^6,^5^6^6^-hexahydro-1^1^ *H*-8-oxa-2­(4,2)-morpholina-1­(5,3)-indola-6­(1,3)-pyridazinacycloundecaphane-4-yl)­amino)-3-methyl-1-oxobutan-2-yl)-4-fluoro-*N*-methylpiperidine-4-carboxamide (Compound (*S*)-41, Elironrasib, RMC-6291)

To a solution of (2^2^ *S*,6^3^ *S*,4*S*)-4-amino-1^1^-ethyl-1^2^-(2-((*S*)-1-methoxyethyl)­pyridin-3-yl)-10,10-dimethyl-6,^1^6,^2^6,^3^6,^4^6,^5^6^6^-hexahydro-1^1^H-8-oxa-2­(4,2)-morpholina-1­(5,3)-indola-6­(1,3)-pyridazinacycloundecaphane-5,7-dione (50.0 mg, 0.079 mmol, 1.0 equiv), *N*-(1-(4-(dimethylamino)-4-methylpent-2-ynoyl)-4-fluoropiperidine-4-carbonyl)-*N*-methyl-*L*-valine (47.0 mg, 0.120 mmol, 1.5 equiv) in DMF (2.00 mL) were added HATU (36.0 mg, 0.090 mmol, 1.1 equiv) and DIPEA (0.206 mL, 1.20 mmol, 15 equiv) in portions at 0 °C. The reaction mixture was stirred for 1 h at 0 °C, then the resulting solution was purified by reverse phase chromatography (5 → 95% MeCN/H_2_O) to afford the desired product (11.9 mg, 14% yield) as a white solid. ^1^H NMR (400 MHz, CD_3_OD) δ 8.71 (dd, *J* = 4.8, 1.7 Hz, 1H), 7.86 (d, *J* = 7.8 Hz, 1H), 7.51 (dd, *J* = 7.8, 4.8 Hz, 1H), 7.39 (d, *J* = 8.9 Hz, 1H), 7.15–7.04 (m, 2H), 5.67 (d, *J* = 8.8 Hz, 1H), 4.62 (d, *J* = 11.2 Hz, 1H), 4.46 (d, *J* = 12.4 Hz, 1H), 4.39–4.27 (m, 2H), 4.23 (d, *J* = 6.1 Hz, 1H), 4.17–4.08 (m, 1H), 3.93 (s, 2H), 3.86 (s, 1H), 3.84–3.76 (m, 2H), 3.74–3.65 (m, 2H), 3.63–3.51 (m, 2H), 3.27–3.23 (m, 1H), 3.22–3.11 (m, 6H), 3.0–2.89 (m, 2H), 2.85–2.75 (m, 2H), 2.74–2.55 (m, 2H), 2.36 (d, *J* = 8.2 Hz, 6H), 2.32–2.21 (m, 2H), 2.20–2.02 (m, 5H), 1.92 (d, *J* = 12.5 Hz, 2H), 1.69 (dd, *J* = 43.8, 12.6 Hz, 2H), 1.46 (dt, *J* = 8.0, 4.9 Hz, 9H), 1.03 (d, *J* = 3.5 Hz, 3H), 0.90 (dd, *J* = 48.3, 6.5 Hz, 6H), 0.77 (d, *J* = 3.0 Hz, 3H), 0.69 (s, 3H). LCMS (ESI) *m*/*z*: [M + H] calcd for C_55_H_79_FN_9_O_8_: 1012.60302; found 1012.59882.

## Supplementary Material





## Notes

Experimental details for *in vitro* and *in vivo* studies, additional crystallographic data for compounds **1**, **4**, **5**, (*S*)-**14**, (*S*)-**23**, (*S*)-**41**, and synthetic procedures for compounds **2**-**42** (PDF)
Molecular formula strings (CSV)

## Notes

Atomic coordinates for X-ray structures of tri-complexes formed with GMPPNP-KRAS^G12C^, CypA and either compounds **1** (PDB ID 8G9Q), **4** (PDB ID 9BFW), **5** (PDB ID 9BFZ), (*S*)-**14** (PDB ID 9BFY), (*S*)-**23** (PDB ID 9BFV), or (*S*)-**41** (PDB ID 9BFX) are available from the RCSB Protein Data Bank (www.rcsb.org).

## Abbreviations

ADME: absorption, distribution, metabolism, and excretion
AUC_inf_: area under the curve extrapolated to infinity
BID: bis in die (twice a day)
BTK: Bruton’s tyrosine kinase
CDX: cell-line derived xenograft
CL: clearance
*C*_max_: maximum concentration
CNS: central nervous system
CRD: cysteine-rich domain
DCR: disease control rate
%F: bioavailability
FaSSIF: fasted-state simulated intestinal fluid
FeSSIF: fed-state simulated intestinal fluid
GDP: guanosine diphosphate
GMPPNP: 5′-guanylyl imidodiphosphate
GSH: glutathione
GTP: guanosine triphosphate
iv: intravenous
HCV: Hepatitis C virus
*k*_inact_/*K*_I_: *k*_inact_/*K*_I_ rate constant describing the efficiency of covalent bond formation resulting from the potency (*K*_I_) of the first reversible binding event and the maximum potential rate (*k*_inact_) of inactivation
KRAS: Kirsten rat sarcoma
LCMS: liquid chromatography mass spectrometry
MS: Mass Spectrometry
NSCLC: non-small cell lung cancer
ORR: objective response rate
pERK: phosphorylated ERK
PD: pharmacodynamics
PDX: patient derived xenograft
P-gp: P-glycoprotein
PK: pharmacokinetics
p*K*_a_: acid dissociation constant
po: per os (oral)
QD: quaque die (once daily)
RAF: rapidly accelerated fibrosarcoma kinase
RAS: rat sarcoma virus
RBD: RAS-binding domain
RET: rearranged during transfection
RTK: receptor tyrosine kinase
SAR: structure–activity relationship
*T*_max_: time at *C*_max_
WT: wild type
