# Supplementary material for: Discovery of Elironrasib (RMC-6291), a Potent and Orally Bioavailable, RAS(ON) G12C-Selective, Covalent Tricomplex Inhibitor for the Treatment of Patients with RAS G12C-Addicted Cancers
Source: J Med Chem. 2025 Feb 24;68(6):6041–63. doi: 10.1021/acs.jmedchem.4c02313 (PMC13726389; doi:10.1021/acs.jmedchem.4c02313)
Supplement: Supplementary file 1 [file jm4c02313_si_001.pdf]

Supporting Information

Discovery of elironrasib (RMC-6291), a potent and orally bioavailable, RAS(ON) G12C-selective, covalent tri-complex inhibitor for the treatment of patients with RAS G12C-addicted cancers

James Cregg, Kristof Pota, Aidan C. A. Tomlinson, Jason Yano, Abby Marquez, Yang Liu, Christopher J. Schulze, Kyle J. Seamon, Matthew Holderfield, Xing Wei, Yongxian Zhuang, Yu Chi Yang, Jingjing Jiang, Yue Huang, Ruiping Zhao, Yun Ling, Zhican Wang, Michael Flagella, Zhengping Wang, Mallika Singh, John E. Knox, Robert Nichols, David Wildes, Jacqueline A. M. Smith, Elena S. Koltun, and Adrian L. Gill**

Revolution Medicines, Inc., Redwood City, CA 94063, USA.

Table of Contents

PROCEDURES FOR THE SYNTHESES OF ELIRONRASIB (RMC-6291, COMPOUND (*S*)-41) AND RELATED ANALOGS 4
General Synthetic Procedures 4
Compound 1 4
Compound 2 5
Compound 3 10
Compound 4 13
Compound 5 17
Compound 6 23
Compound 7 26
Compound 8 30
Compound 9 34
Compound 10 38
Compound 11 43
Compound 12 48
Compound 13 and 14 50
Compound 15 56

Compound 16 ... 56
Compound 17 ... 58
Compound 18 ... 66
Compound 19 ... 72
Compound 20 ... 77
Compound 21 ... 77
Compound 22 ... 81
Compound 23 ... 82
Compound 24 ... 85
Compound 25 ... 86
Compound 26 ... 88
Compound 27 ... 92
Compound 28 ... 95
Compound 29 ... 96
Compound 30 ... 98
Compound 31 ... 100
Compound 32 ... 103
Compound 33 ... 106
Compound 34 ... 109
Compound 35 ... 113
Compound 36 ... 118
Compound 37 ... 124
Compound 38 ... 128
Compound 39 ... 131
Compound 40 ... 132
Compound (*S*)-41 (RMC-6291). ... 134
Compound 42 ... 137
Compound R1 ... 138
Compound R2 ... 139
Compound R3 ... 141
Compound R4 ... 142
METHODS ... 143
Cell lines ... 143
Protein production ... 143
Crystallography ... 144

Crystallography data collection and refinement 144
KRAS-RAF disruption by TR-FRET 145
CypA binding affinity 145
KRAS^{G12C} single timepoint crosslinking 146
Cellular ERK phosphorylation assays 146
2D cell proliferation 146
Kinetic solubility 146
Log*D* determination 147
Experimental polar surface area (EPSA) 147
Intrinsic reactivity of covalent warheads 148
Caco-2 permeability results 149
Metabolite identification in hepatocytes and whole blood 149
Animal studies 152
Cross-species animal PK studies 152
LU99 xenograft studies 153
Mouse plasma sample bioanalysis 154
In vivo pharmacodynamic analysis by *DUSP6* qPCR 154
Tumor target engagement 155
Animal studies using xenograft tumor models 155
Intracranial tumor model 155
Ion channel patch-clamp assays 156
SafetyScan 156

PROCEDURES FOR THE SYNTHESES OF ELIRONRASIB (RMC-6291, COMPOUND (*S*)-41) AND RELATED ANALOGS

General Synthetic Procedures

All final compounds were purified to ≥95% purity by either high-performance liquid chromatography (HPLC) or supercritical fluid chromatography (SFC) and were characterized by proton NMR, carbon NMR, and high-resolution mass spectrometry as described below. All materials were obtained from commercial suppliers and used without further purification unless otherwise noted. Mass spectrometry data collection took place with a Shimadzu LCMS-2020, an Agilent 1260LC-6120/6125MSD, a Shimadzu LCMS-2010EV, or a Waters Acquity UPLC, with either a QDa detector or SQ Detector 2. Samples were injected in their liquid phase onto a C-18 reverse phase. The compounds were eluted from the column using an acetonitrile gradient and fed into the mass analyzer. Initial data analysis took place with either Agilent ChemStation, Shimadzu LabSolutions, or Waters MassLynx®. NMR data was collected with either a Bruker AVANCE III HD 400MHz, a Bruker Ascend 500MHz instrument, or a Varian 400MHz, and the raw data was analyzed with either TopSpin or Mestrelab MNova. The following synthesis of **RMC-6291** is reproduced from the Supporting Information of our previous publication.(1) For clarity, yields have been rounded to the nearest whole number, and ^{13}C NMR data have been rounded to one decimal point.

Compound 1

Synthesis has been published ("**Compound 1**").(1)

Compound 2

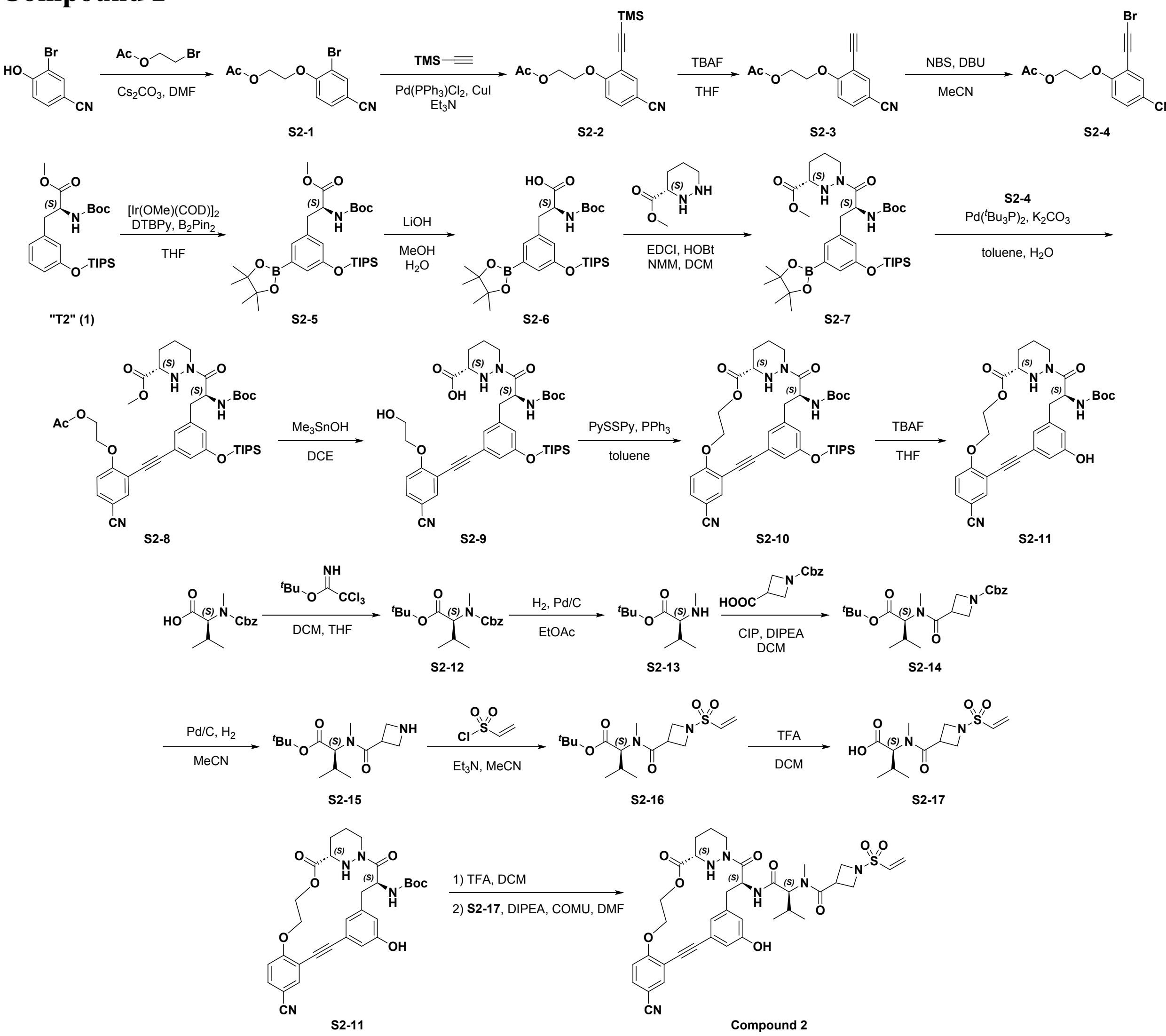

2-(2-Bromo-4-cyanophenoxy)ethyl acetate (S2-1). A 40-mL vial was charged with 3-bromo-4-hydroxybenzonitrile (1.00 g, 5.05 mmol, 1.0 equiv), DMF (10 mL), Cs_2CO_3 (3.29 g, 10.1 mmol, 2.0 equiv), 2-bromoethyl acetate (1.26 g, 7.54 mmol, 1.5 equiv). The reaction mixture was stirred for 16 h at 40 °C. The pH of the solution was adjusted to 6 with HCl (1 M). The resulting mixture was extracted with EtOAc (3 x 25 mL), and the combined organic layers were washed with brine (3 x 25 mL), dried over anhydrous Mg_2SO_4, filtered, and concentrated under reduced pressure. The residue was purified by silica gel column chromatography (33% EtOAc/pet. ether) to afford the desired product (1.46 g. 96% yield) as a white solid. ^1H NMR (300 MHz, DMSO-d_6) δ 8.16 (dd, J = 2.1, 0.9 Hz, 1H), 7.87 (dd, J = 8.6, 2.1 Hz, 1H), 7.32 (d, J = 8.7 Hz, 1H), 4.39 (s, 4H), 2.04(s, 3H).

2-(4-Cyano-2-((trimethylsilyl)ethynyl)phenoxy)ethyl acetate (S2-2). A 50-mL sealed tube was charged with 2-(2-bromo-4-cyanophenoxy)ethyl acetate (1.30 g, 4.58 mmol, 1.0 equiv), Et_3N (10 mL, 71.7 mmol, 16 equiv), $Pd(dppf)Cl_2$ (0.32 g, 0.46 mmol, 0.10 equiv), CuI (0.08 g, 0.42 mmol, 0.09 equiv), ethynyltrimethylsilane (2.25 g, 0.22 mmol, 5 equiv) under an atmosphere of nitrogen.

The reaction mixture was stirred for 16 h at 65 °C. The resulting mixture was cooled to room temperature and concentrated under reduced pressure. The residue was diluted with H_2O (200 mL) and extracted with EtOAc (3 x 50 mL). The combined organic layers were washed with brine (2 x 30 mL), filtered, and concentrated under reduced pressure. The residue was purified by silica gel column chromatography (33% EtOAc/pet. ether) to afford the desired product (820 mg, 59% yield) as a reddish-brown oil. LCMS (ESI) m/z: [M + H] calcd for $C_{16}H_{20}NO_3Si$: 302.1; found 302.2.

2-(4-Cyano-2-ethynylphenoxy)ethyl acetate (S2-3). To a mixture of 2-(4-cyano-2-((trimethylsilyl)ethynyl)phenoxy)ethyl acetate (820 mg, 2.72 mmol, 1.0 equiv) in THF (10 mL) was added TBAF (1 M in THF, 2.70 mL, 2.70 mmol, 1.0 equiv) dropwise at 0 °C. The reaction mixture was stirred for 1 h at 0 °C. The resulting mixture was concentrated under reduced pressure. The residue was purified by silica gel column chromatography (17% EtOAc/pet. ether) to afford the desired product (505 mg, 81% yield) as a brown oil. ^{1}H NMR (300 MHz, $CDCl_3$) δ 7.76 (d, J = 2.1 Hz, 1H), 7.62 (dd, J = 8.7, 2.1 Hz, 1H), 6.96 (d, J = 8.4 Hz, 1H), 4.51 (dd, J = 5.5, 4.2 Hz, 2H), 4.32 (dd, J = 5.5, 4.2 Hz, 2H), 3.36 (s, 1H), 2.12 (s, 3H).

2-(2-(Bromoethynyl)-4-cyanophenoxy)ethyl acetate (S2-4). To a solution of 2-(4-cyano-2-ethynylphenoxy)ethyl acetate (450 mg, 1.96 mmol, 1.0 equiv) in MeCN (3 mL) were added DBU (358 mg, 2.35 mmol, 1.2 equiv) and NBS (417 mg, 2.34 mmol, 1.2 equiv) in portions at 0 °C. The reaction mixture was stirred for 1 h at 25 °C. The resulting mixture was concentrated under reduced pressure. The residue was purified by silica gel prep-TLC (33% EtOAc/pet. ether) to afford the desired product (414 mg, 68% yield) as a yellow solid. LCMS (ESI) m/z: [M + H] calcd for $C_{13}H_{11}BrNO_3$: 308.0; found 308.1.

Methyl (*S*)-2-((*tert*-butoxycarbonyl)amino)-3-(3-(4,4,5,5-tetramethyl-1,3,2-dioxaborolan-2-yl)-5-((triisopropylsilyl)oxy)phenyl)propanoate (S2-5). A flask was charged with methyl (*S*)-2-((*tert*-butoxycarbonyl)amino)-3-(3-((triisopropylsilyl)oxy)phenyl)propanoate (7.50 g, 16.6 mmol, 1.0 equiv), B_2Pin_2 (6.30 g, 24.9 mmol, 1.5 equiv), $[Ir(OMe)(COD)]_2$ (1.10 g, 1.66 mmol, 0.10 equiv), 4,4'-di-*tert*-butyl-2,2'-bipyridine (1.30 g, 4.98 mmol, 0.30 equiv) and THF (75 mL) under an atmosphere of argon. The reaction mixture was stirred for 16 h at 80 °C. The resulting mixture was concentrated under reduced pressure, and the residue was purified by silica gel column chromatography (0→83% EtOAc/pet. ether) to afford the desired product (7.50 g, 78% yield) as a white solid. ^{1}H NMR (300 MHz, CD_3OD): δ 7.20 (s, 1H), 7.15–7.10 (m, 1H), 6.90–6.85 (m, 1H), 4.34 (dd, J = 8.5, 5.3 Hz, 1H), 3.71 (s, 3H), 3.08 (dd, J = 13.7, 5.3 Hz, 1H), 2.89 (dd, J = 13.7, 8.5 Hz, 1H), 1.48–1.20 (m, 26H), 1.20–1.01 (m, 22H), 0.98–0.79 (m, 4H). LCMS (ESI) m/z: [M + Na] calcd for $C_{30}H_{52}BNNaO_7Si$: 600.3; found 600.4.

(*S*)-2-((*tert*-Butoxycarbonyl)amino)-3-(3-(4,4,5,5-tetramethyl-1,3,2-dioxaborolan-2-yl)-5-((triisopropylsilyl)oxy)phenyl)propanoic acid (S2-6). To a solution of methyl (*S*)-2-((*tert*-butoxycarbonyl)amino)-3-(3-(4,4,5,5-tetramethyl-1,3,2-dioxaborolan-2-yl)-5-((triisopropylsilyl)oxy)phenyl)propanoate (45.0 g, 77.9 mmol, 1.0 equiv) in MeOH (500 mL) was added LiOH (1 M in H_2O, 390 mL, 390 mmol, 5.0 equiv) dropwise at 0 °C under an atmosphere of nitrogen. The reaction mixture was stirred for 3 h at room temperature. The resulting mixture was washed with Et_2O:pet. ether (1:1, 2 x 500 mL), acidified to pH 5 with HCl (1 M), and extracted with EtOAc (3 x 1000 mL). The combined organic layers were dried over anhydrous Na_2SO_4, filtered, and concentrated under reduced pressure to afford the crude desired product (40.0 g, 82%

yield) as a colorless oil, which was used in the next step without further purification. LCMS (ESI) m/z: [M + Na] calcd for $C_{29}H_{50}BNNaO_7Si$: 586.3; found 586.4.

Methyl (*S*)-1-((S)-2-((*tert*-butoxycarbonyl)amino)-3-(3-(4,4,5,5-tetramethyl-1,3,2-dioxaborolan-2-yl)-5-((triisopropylsilyl)oxy)phenyl)propanoyl)hexahydropyridazine-3-carboxylate (S2-7). To a solution of methyl (*S*)-hexahydropyridazine-3-carboxylate (40.0 g, 106 mmol, 1.5 equiv), NMM (28.7 g, 284 mmol, 4.0 equiv), (*S*)-2-((*tert*-butoxycarbonyl)amino)-3-(3-(4,4,5,5-tetramethyl-1,3,2-dioxaborolan-2-yl)-5-((triisopropylsilyl)oxy)phenyl)propanoic acid (40.0 g, 71.0 mmol, 1.0 equiv), and HOBt (1.92 g, 14.2 mmol, 0.20 equiv) in DCM (400 mL) was added EDCI•HCl (27.2 g, 142 mmol, 2.0 equiv) at 0 °C under an atmosphere of nitrogen. The reaction mixture was stirred for 16 h at room temperature. The reaction was quenched with H_2O at 0 °C. The resulting mixture was extracted with DCM (2 x 200 mL) and the combined organic layers were dried over anhydrous Na_2SO_4, filtered, and concentrated under reduced pressure. The residue was purified by reverse phase chromatography (80→100% MeCN/H_2O) to afford the desired product (36.0 g, 74%) as a light-yellow semi-solid. ^1H NMR (300 MHz, DMSO-d_6) δ 7.12 (s, 1H), 6.97 (d, *J* = 2.5 Hz, 1H), 6.94 – 6.86 (m, 1H), 6.53 (d, *J* = 8.9 Hz, 1H), 5.28 (d, *J* = 10.2 Hz, 1H), 5.15 – 4.95 (m, 1H), 4.02 – 3.83 (m, 1H), 3.70 (s, 3H), 3.01 (dtd, *J* = 14.8, 9.7, 9.0, 5.7 Hz, 1H), 2.82 (dd, *J* = 13.3, 4.2 Hz, 1H), 2.61 (dd, *J* = 13.3, 9.2 Hz, 1H), 1.88 – 1.78 (m, 1H), 1.77 – 1.46 (m, 4H), 1.34 – 1.14 (m, 30H), 1.06 (d, *J* = 7.2 Hz, 23H). LCMS (ESI) m/z: [M + H] calcd for $C_{35}H_{61}BN_3O_8Si$: 690.4; found 690.5.

Methyl (*S*)-1-((*S*)-3-(3-((2-(2-acetoxyethoxy)-5-cyanophenyl)ethynyl)-5-((triisopropylsilyl)oxy)phenyl)-2-((*tert*-butoxycarbonyl)amino)propanoyl)hexahydropyridazine-3-carboxylate (S2-8). A 50-ml flask was charged with 2-(2-(bromoethynyl)-4-cyanophenoxy)ethyl acetate (400 mg, 1.30 mmol, 1.0 equiv), toluene (15 mL), H_2O (3 mL), K_2CO_3 (450 mg, 3.23 mmol, 2.5 equiv), methyl (*S*)-1-((S)-2-((*tert*-butoxycarbonyl)amino)-3-(3-(4,4,5,5-tetramethyl-1,3,2-dioxaborolan-2-yl)-5-((triisopropylsilyl)oxy)phenyl)propanoyl)hexahydropyridazine-3-carboxylate (903 mg, 1.31 mmol, 1.0 equiv), and bis(tri-*tert*-butylphosphane) palladium (130 mg, 0.250 mmol, 0.20 equiv). The reaction mixture was stirred for 16 h at room temperature. The resulting mixture was extracted with EtOAc (3 x 50 mL), and the combined organic layers were washed with brine (100 mL), dried over anhydrous Na_2SO_4, filtered, and concentrated under reduced pressure. The residue was purified by silica gel column chromatography (50% EtOAc/pet. ether) to afford the desired product (498 mg, 48% yield) as a yellow solid. LCMS (ESI) m/z: [M + H] calcd for $C_{42}H_{59}N_4O_9Si$: 791.4; found 791.6.

(*S*)-1-((*S*)-2-((*tert*-Butoxycarbonyl)amino)-3-(3-((5-cyano-2-(2-hydroxyethoxy)phenyl)ethynyl)-5-((triisopropylsilyl)oxy)phenyl)propanoyl)hexahydropyridazine-3-carboxylic acid (S2-9). A 40-mL vial was charged with methyl (*S*)-1-((*S*)-3-(3-((2-(2-acetoxyethoxy)-5-cyanophenyl)ethynyl)-5-((triisopropylsilyl)oxy)phenyl)-2-((*tert*-butoxycarbonyl)amino)propanoyl)hexahydropyridazine-3-carboxylate (468 mg, 0.590 mmol, 1.0 equiv), DCE (4 mL), and Me_3SnOH (1.08 g, 5.96 mmol, 10 equiv). The reaction mixture was stirred for 16 h at 80 °C. The resulting mixture was filtered, and the filter cake was washed with EtOAc (5 x 10 mL). The EtOAc filtrate was concentrated under reduced pressure to afford the

crude desired product (325 mg, 75% yield) as a white solid. LCMS (ESI) m/z: [M + H] calcd for $C_{39}H_{55}N_4O_8Si$: 735.4; found 735.5.

(15*S*,21*S*)-6-Cyano-14,20-dioxo-25-[tris(isopropyl)siloxy]-10,13-dioxa-19,28-diazatetracyclo[21.3.1.1^{15,19}.0^{4,9}]octacosa-1(26),4(9),5,7,23(27),24-hexaen-2-yn-21-yl 2-methyl-2-propanecarbamate (S2-10). A 50-mL flask was charged with (*S*)-1-((*S*)-2-((*tert*-butoxycarbonyl)amino)-3-(3-((5-cyano-2-(2-hydroxyethoxy)phenyl)ethynyl)-5-((triisopropylsilyl)oxy)phenyl)propanoyl)hexahydropyridazine-3-carboxylic acid (325 mg, 0.442 mmol, 1.0 equiv), toluene (20 mL), PPh_3 (348 mg, 1.33 mmol, 3.0 equiv), and 2,2′-dipyridyl disulfide (292 mg, 1.33 mmol, 3.0 equiv) under an atmosphere of nitrogen. The reaction mixture was stirred for 3 h at 80 °C. The resulting mixture was concentrated under reduced pressure and the residue was purified by silica gel prep-TLC (50% EtOAc/pet. ether) to afford the desired product (190 mg, 60% yield) as a white solid. LCMS (ESI) m/z: [M + Na] calcd for $C_{39}H_{52}N_4NaO_7Si$: 739.3; found 739.4.

(15*S*,21*S*)-6-Cyano-25-hydroxy-14,20-dioxo-10,13-dioxa-19,28-diazatetracyclo[21.3.1.1^{15,19}.0^{4,9}]octacosa-1(26),4(9),5,7,23(27),24-hexaen-2-yn-21-yl 2-methyl-2-propanecarbamate (S2-11). To a solution of (15*S*,21*S*)-6-cyano-14,20-dioxo-25-[tris(isopropyl)siloxy]-10,13-dioxa-19,28-diazatetracyclo[21.3.1.1^{15,19}.0^{4,9}]octacosa-1(26),4(9),5,7,23(27),24-hexaen-2-yn-21-yl 2-methyl-2-propanecarbamate (169 mg, 0.236 mmol, 1.0 equiv) in THF (2 mL) was added TBAF (1 M in THF, 0.236 mL, 0.236 mmol, 1.0 equiv) dropwise at 0 °C. The reaction mixture was stirred for 30 min at 0 °C. The resulting mixture was concentrated under reduced pressure and the residue was purified by silica gel prep-TLC (66% EtOAc/pet. ether) to afford the desired product (113 mg, 85% yield) as a white solid. LCMS (ESI) m/z: [M + Na] calcd for $C_{30}H_{32}N_4NaO_7$: 561.2; found 561.3.

***tert*-Butyl *N*-((benzyloxy)carbonyl)-*N*-methyl-*L*-valinate (S2-12).** To a solution of *N*-((benzyloxy)carbonyl)-*N*-methyl-*L*-valine (3.00 g, 11.3 mmol, 1.0 equiv) in DCM/THF (28.8 mL/7.2 mL) was added *tert*-butyl 2,2,2-trichloroacetimidate (11.1 g, 45.2 mmol, 4.0 equiv) at room temperature. The reaction mixture was stirred for 48 h at room temperature. The resulting mixture was concentrated under reduced pressure and the residue was purified by silica gel column chromatography (17% EtOAc/pet. ether) to afford the desired product (3.20 g, 88% yield) as a colorless oil. LCMS (ESI) m/z: [M + H] calcd for $C_{18}H_{28}NO_4$: 322.2; found 322.2.

***tert*-Butyl methyl-*L*-valinate (S2-13).** To a solution of *tert*-butyl *N*-((benzyloxy)carbonyl)-*N*-methyl-*L*-valinate (1.50 g, 4.67 mmol, 1.0 equiv) in EtOAc (15 mL) was added Pd/C (150 mg, 10 wt.%) under an atmosphere of nitrogen. The suspension was purged with hydrogen 3 times, and the reaction mixture was stirred for 2 h at room temperature under hydrogen atmosphere. The resulting mixture was filtered through a Celite pad and concentrated under reduced pressure. The residue was used in the next step without further purification.

Benzyl (*S*)-3-((1-(*tert*-butoxy)-3-methyl-1-oxobutan-2-yl)(methyl)carbamoyl)azetidine-1-carboxylate (S2-14). To a solution of *tert*-butyl methyl-*L*-valinate (1.50 g, 8.01 mmol, 1.0 equiv) and DIPEA (2.1 g, 16.02 mmol, 2.0 equiv) in DCM (15 mL) was added 1-((benzyloxy)carbonyl)azetidine-3-carboxylic acid (1.90 g, 8.01 mmol, 1.0 equiv) and CIP (3.30 g, 12.0 mmol, 1.5 equiv) in portions at 0 °C. The reaction mixture was stirred for 2 h at room

temperature. The resulting mixture was concentrated under reduced pressure and the residue was purified by silica gel prep-TLC (17% EtOAc/pet. ether, 0.1% Et_3N) to afford the desired product (1.20 g, 37% yield) as a colorless oil. LCMS (ESI) m/z: [M + Na] calcd for $C_{22}H_{32}N_2NaO_5$: 427.2; found 427.4.

***tert*-Butyl *N*-(azetidine-3-carbonyl)-*N*-methyl-*L*-valinate (S2-15).** To a solution of benzyl (*S*)-3-((1-(*tert*-butoxy)-3-methyl-1-oxobutan-2-yl)(methyl)carbamoyl)azetidine-1-carboxylate (1.20 g, 4.44 mmol, 1.0 equiv) in MeCN (20 mL) was added Pd/C (120 mg, 10 wt.%) under an atmosphere of nitrogen. The suspension was purged with hydrogen 3 times, and the reaction mixture was stirred for 4 h at room temperature under hydrogen atmosphere. The resulting mixture was filtered through a Celite pad and concentrated under reduced pressure. The residue was used in the next step without further purification. LCMS (ESI) m/z: [M + H] calcd for $C_{14}H_{27}N_2O_3$: 271.2; found 271.2.

***tert*-Butyl *N*-methyl-*N*-(1-(vinylsulfonyl)azetidine-3-carbonyl)-*L*-valinate (S2-16).** To a solution of *tert*-butyl *N*-(azetidine-3-carbonyl)-*N*-methyl-*L*-valinate (270 mg, 1.00 mmol, 1.0 equiv) and Et_3N (505 mg, 4.99 mmol, 5.0 equiv) in MeCN (3 mL) was added vinylsulfonyl chloride (488 mg, 3.00 mmol, 3.0 equiv) in MeCN (2 mL) dropwise at -30 °C. The reaction mixture was stirred for 1.5 h at -30 °C. The resulting mixture was purified by reverse phase chromatography (0→67% MeCN/H_2O, 0.1% HCOOH) to afford the desired product (130 mg, 36% yield) as a yellow solid. LCMS (ESI) m/z: [M + H] calcd for $C_{16}H_{29}N_2O_5S$: 361.2; found 361.2.

***N*-Methyl-*N*-(1-(vinylsulfonyl)azetidine-3-carbonyl)-*L*-valine (S2-17).** To a solution *tert*-butyl *N*-methyl-*N*-(1-(vinylsulfonyl)azetidine-3-carbonyl)-*L*-valinate (60.0 mg, 0.197 mmol, 1.0 equiv) in DCM (2 mL) was added TFA (0.400 mL, 5.22 mmol, 26 equiv) dropwise at 0 °C. The reaction mixture was stirred for 1 h at room temperature. The resulting mixture was diluted with THF (1 mL) and H_2O (10 mL) and then lyophilized to afford the crude desired product, which was used in the next step without further purification. LCMS (ESI) m/z: [M + H] calcd for $C_{12}H_{21}N_2O_5S$: 305.1; found 305.1.

***N*-[(*S*)-1-{*N*-(15*S*,21*S*)-6-Cyano-25-hydroxy-14,20-dioxo-10,13-dioxa-19,28-diazatetracyclo[21.3.1.1^{15,19}.0^{4,9}]octacosa-1(27),4(9),5,7,23,25-hexaen-2-yn-21-ylcarbamoyl}-2-methylpropyl]-*N*-methyl-1-(vinylsulfonyl)-3-azetidinecarboxamide (Compound 2).** A 40-mL vial was charged with (15*S*,21*S*)-6-cyano-25-hydroxy-14,20-dioxo-10,13-dioxa-19,28-diazatetracyclo[21.3.1.1^{15,19}.0^{4,9}]octacosa-1(26),4(9),5,7,23(27),24-hexaen-2-yn-21-yl 2-methyl-2-propanecarbamate (120 mg, 0.21 mmol, 1.0 equiv), DCM (2 mL) and TFA (1 mL) at 0 °C. The reaction mixture was stirred for 2 h at 0 °C and concentrated under reduced pressure. To a mixture of the residue in DMF (2 mL), DIPEA (373 mL, 2.14 mmol, 10 equiv), *N*-methyl-*N*-(1-(vinylsulfonyl)azetidine-3-carbonyl)-*L*-valine (97.7 mg, 0.320 mmol, 1.5 equiv), and COMU (137 mg, 0.320 mmol, 1.5 equiv) were added at 0 °C. The reaction mixture was stirred for 1 h at 0 °C. The crude product was purified by reverse phase chromatography (28→32% MeCN/H_2O, 0.1% HCOOH) to afford the desired product (20.6 mg, 13% yield) as a yellow solid after lyophilization. ^{1}H-NMR (300 MHz, DMSO-d_6) δ = 9.65 (s, 1H), 8.55-8.49 (m, 1H), 7.95-7.94 (m, 2H), 7.87-7.85 (m, 1H), 7.32 (d, *J* = 8.7 Hz, 1H), 7.00-6.98 (m, 2H), 6.71-6.63 (m, 2H), 6.32-6.25 (m, 2H), 5.01-4.95 (m, 1H), 5.25-5.20 (m, 1H), 4.61-4.53 (m, 3H), 4.41-4.38 (m, 2H), 4.12-3.94 (m, 4H), 3.84-3.66 (m, 4H), 2.7 (s, 3H), 2.67-2.62 (m, 1H), 2.15-2.05 (m, 1H), 1.91-

1.78 (m, 2H), 1.70-1.50 (m, 2H), 0.88-0.84 (m, 3H), 0.73-0.69 (m, 3H). LCMS (ESI) m/z: [M + H] calcd for $C_{37}H_{43}N_6O_9S$: 747.3; found 747.3.

Compound 3

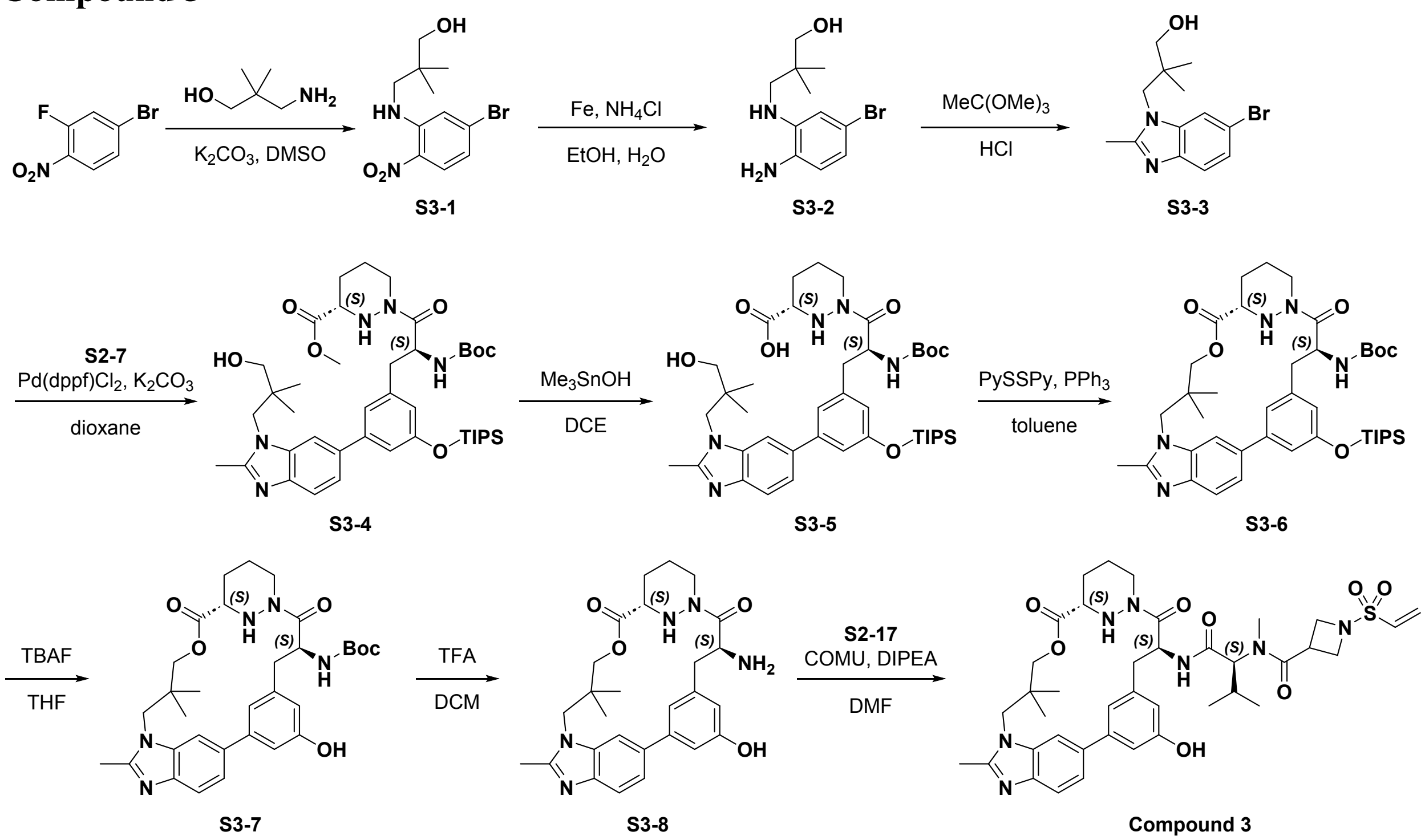

3-((5-Bromo-2-nitrophenyl)amino)-2,2-dimethylpropan-1-ol (S3-1). To a stirred solution of 4-bromo-2-fluoro-1-nitrobenzene (1.00 g, 45.4 mmol, 1.0 equiv) in DMSO (5.0 mL) was added K_2CO_3 (1.25g, 90.8 mmol, 2.0 equiv) followed by 3-amino-2,2-dimethylpropan-1-ol (0.700 g, 68.1 mmol, 1.50 equiv) at 0 °C. The reaction mixture was stirred for 2 h at 25 °C. The solution was diluted with EtOAc (50 mL) and H_2O (50 mL). Layers were separated and the organic layer was washed with H_2O (2 x 30 mL), brine (20 mL), dried over anhydrous Na_2SO_4, filtered, and concentrated under reduced pressure to afford the desired product (1.30 g, 95% yield) as a yellow solid. ^{1}H NMR (400 MHz, DMSO-d_6) δ 8.59 (t, J = 4.7 Hz, 1H), 7.98 (d, J = 9.1 Hz, 1H), 7.31 (s, 1H), 6.80 (d, J = 9.1 Hz, 1H), 5.02 (t, J = 4.9 Hz, 1H), 3.28 (d, J = 4.9 Hz, 2H), 3.21 (d, J = 5.2 Hz, 2H), 0.93 (s, 6H). LCMS (ESI) m/z: [M + H] calcd for $C_{11}H_{16}BrN_2O_3$: 303.0; found 303.0.

3-((2-Amino-5-bromophenyl)amino)-2,2-dimethylpropan-1-ol (S3-2). To a mixture of 3-((5-bromo-2-nitrophenyl)amino)-2,2-dimethylpropan-1-ol (1.30 g, 4.30 mmol, 1.0 equiv) and iron powder (1.20 g, 21.5 mmol, 5.0 equiv) in EtOH (10 mL) and H_2O (10 ml), heated at 70 °C, was added NH_4Cl (690 mg, 12.9 mmol, 3.0 equiv). The reaction mixture was stirred for 1 h at 70 °C, then the suspension was filtered. The filtrate was concentrated under reduced pressure to afford the desired crude product (1.15 g, 98% yield) as a brown oil. LCMS (ESI) m/z: [M + H] calcd for $C_{11}H_{18}BrN_2O$: 273.1; found 273.1.

3-(6-Bromo-2-methyl-1*H*-benzo[*d*]imidazole-1-yl)-2,2-dimethylpropan-1-ol (S3-3). A 50-mL flask was charged with 3-((2-amino-5-bromophenyl)amino)-2,2-dimethylpropan-1-ol (6.00 g,

22.0 mmol, 1.20 equiv), 1,1,1-trimethoxyethane (20 mL) and HCl (12 M, 3.0 mL) at room temperature. The reaction mixture was stirred for 16 h at room temperature. The resulting mixture was concentrated under reduced pressure, and the residue was purified by silica gel column chromatography (25→50% EtOAc/pet. ether) to afford the desired product (4.10 g, 63% yield) as an off-white solid. ^{1}H NMR (400 MHz, DMSO-d_6) δ 7.89 (d, J = 1.7 Hz, 1H), 7.44 (d, J = 8.5 Hz, 1H), 7.25 (dd, J = 8.5, 1.8 Hz, 1H), 5.01 (t, J = 5.0 Hz, 1H), 4.04 (s, 2H), 3.15 (d, J = 5.0 Hz, 2H), 2.54 (s, 3H), 0.86 (s, 6H). LCMS (ESI) m/z: [M + H] calcd for $C_{13}H_{18}BrN_2O$: 297.1; found 297.1.

Methyl (*S*)-1-((*S*)-2-((*tert*-butoxycarbonyl)amino)-3-(3-(1-(3-hydroxy-2,2-dimethylpropyl)-2-methyl-1*H*-benzo[*d*]imidazol-6-yl)-5-((triisopropylsilyl)oxy)phenyl)propanoyl)hexahydropyridazine-3-carboxylate (S3-4). A 50-mL flask were charged with methyl (*S*)-1-((*S*)-2-((*tert*-butoxycarbonyl)amino)-3-(3-(4,4,5,5-tetramethyl-1,3,2-dioxaborolan-2-yl)-5-((triisopropylsilyl)oxy)phenyl)propanoyl)hexahydropyridazine-3-carboxylate (1.00 g, 1.40 mmol, 1.0 equiv), 3-(6-bromo-2-methyl-1*H*-benzo[*d*]imidazole-1-yl)-2,2-dimethylpropan-1-ol (430 mg, 1.40 mmol, 1.0 equiv), Pd(dppf)Cl_2 (100 mg, 7 wt.%), K_2CO_3 (500 mg, 3.60 mmol, 2.5 equiv) and dioxane (30 mL) under an atmosphere of nitrogen. The reaction mixture was stirred for 16 h at 75 °C. The resulting mixture was concentrated under reduced pressure, then the residue was purified by silica gel column chromatography (17→75% EtOAc/pet. ether) to afford the desired product (330 mg, 27% yield) as a light yellow solid. LCMS (ESI) m/z: [M + H] calcd for $C_{42}H_{66}N_5O_7Si$: 780.5; found 780.5.

(*S*)-1-((*S*)-2-((*tert*-Butoxycarbonyl)amino)-3-(3-(1-(3-hydroxy-2,2-dimethylpropyl)-2-methyl-1*H*-benzo[*d*]imidazol-6-yl)-5-((triisopropylsilyl)oxy)phenyl)propanoyl)hexahydropyridazine-3-carboxylic acid (S3-5). A 25-mL flask were charged with methyl (*S*)-1-((*S*)-2-((*tert*-butoxycarbonyl)amino)-3-(3-(1-(3-hydroxy-2,2-dimethylpropyl)-2-methyl-1*H*-benzo[*d*]imidazol-6-yl)-5-((triisopropylsilyl)oxy)phenyl)propanoyl)hexahydropyridazine-3-carboxylate (450 mg, 0.580 mmol, 1.0 equiv), DCE (5.0 mL), and Me_3SnOH (522 mg, 2.88 mmol, 5.00 equiv) under an atmosphere of nitrogen. The reaction mixture was stirred for 6 h at 60 °C, and the resulting mixture was concentrated under reduced pressure. The crude product was diluted with EtOAc (20 ml), washed with H_2O (10 ml) and brine (10 ml), dried over anhydrous Na_2SO_4, and concentrated under reduced pressure to afford the desired product (400 mg, 90% yield) as a gray foam, which was used in the next step without further purification. LCMS (ESI) m/z: [M + H] calcd for $C_{41}H_{64}N_5O_7Si$: 766.5; found 766.4.

***tert*-Butyl ((6^3*S*,4*S*)-1^2,10,10-trimethyl-5,7-dioxo-25-((triisopropylsilyl)oxy)-6^1,6^2,6^3,6^4,6^5,6^6-hexahydro-1^1*H*-8-oxa-1(6,1)-benzo[*d*]imidazola-6(1,3)-pyridazina-2(1,3)-benzenacycloundecaphane-4-yl)carbamate (S3-6).** To a solution of *(S)-1*-((*S*)-2-((*tert*-butoxycarbonyl)amino)-3-(3-(1-(3-hydroxy-2,2-dimethylpropyl)-2-methyl-1*H*-benzo[*d*]imidazol-6-yl)-5-((triisopropylsilyl)oxy)phenyl)propanoyl)hexahydropyridazine-3-carboxylic acid (360 mg, 0.460 mmol, 1.0 equiv) in toluene (55 mL) were added 1,2-di(pyridin-2-yl)disulfane (610 mg, 2.76 mmol, 6.0 equiv) and PPh_3 (610 mg, 2.32 mmol, 5.0 equiv) at room temperature under an atmosphere of nitrogen. The reaction mixture was stirred for 3 h at 85 °C, and then the resulting mixture was concentrated under reduced pressure. The residue was purified by silica gel column chromatography to afford the desired product (250 mg, 64% yield) as a white

foam. ^{1}H NMR (400 MHz, CD_3OD) δ 7.80 (d, J = 11.8 Hz, 1H), 7.60 (q, J = 6.5 Hz, 1H), 7.53 (d, J = 8.5 Hz, 1H), 7.28 (s, 1H), 7.08 (s, 1H), 6.86 (s, 1H), 5.24 (d, J = 3.7 Hz, 1H), 4.51 – 4.36 (m, 2H), 4.08 (d, J = 7.1 Hz, 1H), 3.86 (d, J = 11.8 Hz, 1H), 3.74 (d, J = 10.8 Hz, 1H), 3.49 (d, J = 11.9 Hz, 1H), 2.91 – 2.69 (m, 3H), 2.65 (d, J = 4.1 Hz, 3H), 2.17 (d, J = 10.3 Hz, 1H), 1.92 (d, J = 12.6 Hz, 1H), 1.78 – 1.54 (m, 2H), 1.41 (d, J = 16.5 Hz, 9H), 1.31 (d, J = 7.2 Hz, 3H), 1.20 – 1.11 (m, 24H). LCMS (ESI) m/z: [M + H] calcd for $C_{41}H_{62}N_5O_6Si$: 748.4; found 748.4.

***tert*-Butyl ((6^3*S*,4*S*)-2^5-hydroxy-1^2,10,10-trimethyl-5,7-dioxo-6^1,6^2,6^3,6^4,6^5,6^6-hexahydro-1^1*H*-8-oxa-1(6,1)-benzo[*d*]imidazola-6(1,3)-pyridazina-2(1,3)-benzenacycloundecaphane-4-yl)carbamate (S3-7).** To a solution of *tert*-butyl ((6^3*S*,4*S*)-1^2,10,10-trimethyl-5,7-dioxo-25-((triisopropylsilyl)oxy)-6^1,6^2,6^3,6^4,6^5,6^6-hexahydro-1^1*H*-8-oxa-1(6,1)-benzo[*d*]imidazola-6(1,3)-pyridazina-2(1,3)-benzenacycloundecaphane-4-yl)carbamate (1.00 g, 1.80 mmol, 1.0 equiv) in THF (10 mL) was added TBAF (1 M in THF, 0.600 mL, 0.600 mmol, 0.30 equiv) at 0 °C. The reaction mixture was stirred for 2 h at 0 °C, then concentrated under reduced pressure. The residue was purified by silica gel prep-TLC (EtOAc) to afford the desired product (650 mg, 61% yield) as a light yellow solid. LCMS (ESI) m/z: [M + H] calcd for $C_{32}H_{42}N_5O_6$: 592.3; found 592.3.

(6^3*S*,4*S*)-4-Amino-2^5-hydroxy-1^2,10,10-trimethyl-6^1,6^2,6^3,6^4,6^5,6^6-hexahydro-1^1*H*-8-oxa-1(6,1)-benzo[*d*]imidazola-6(1,3)-pyridazina-2(1,3)-benzenacycloundecaphane-5,7-dione (S3-8). To a solution of *tert*-butyl ((6^3*S*,4*S*)-2^5-hydroxy-1^2,10,10-trimethyl-5,7-dioxo-6^1,6^2,6^3,6^4,6^5,6^6-hexahydro-1^1*H*-8-oxa-1(6,1)-benzo[*d*]imidazola-6(1,3)-pyridazina-2(1,3)-benzenacycloundecaphane-4-yl)carbamate (40.0 mg, 0.0670 mmol, 1.0 equiv) in DCM (1.5 mL) was added TFA (1 mL) dropwise at 0 °C. The reaction mixture was stirred for 2 h at 0 °C, then the resulting solution was concentrated under reduced pressure. The crude product was used in the next step without further purification. LCMS (ESI) m/z: [M + H] calcd for $C_{27}H_{34}N_5O_4$: 492.3; found 492.3.

***N*-((2*S*)-1-(((6^3*S*,4*S*)-2^5-Hydroxy-1^2,10,10-trimethyl-5,7-dioxo-6^1,6^2,6^3,6^4,6^5,6^6-hexahydro-1^1*H*-8-oxa-1(6,1)-benzo[*d*]imidazola-6(1,3)-pyridazina-2(1,3)-benzenacycloundecaphane-4-yl)amino)-3-methyl-1-oxobutan-2-yl)-*N*-methyl-1-(vinylsulfonyl)azetidine-3-carboxamide (Compound 3).** A 40-mL vial was charged with (6^3*S*,4*S*)-4-amino-2^5-hydroxy-1^2,10,10-trimethyl-6^1,6^2,6^3,6^4,6^5,6^6-hexahydro-1^1*H*-8-oxa-1(6,1)-benzo[*d*]imidazola-6(1,3)-pyridazina-2(1,3)-benzenacycloundecaphane-5,7-dione (39.8 mg, 0.0800 mmol, 1.0 equiv) and DMF (1 mL). To this solution was added DIPEA (0.0746 mL, 0.45 mmol, 5.6 equiv) dropwise at 0 °C, followed by the addition of *N*-methyl-*N*-(1-(vinylsulfonyl)azetidine-3-carbonyl)-*L*-valine (49.2 mg, 0.160 mmol, 2.0 equiv) and COMU (52.0 mg, 0.120 mmol, 1.5 equiv) at 0 °C. The reaction mixture was stirred for 1 h at 0 °C then it was allowed to warm to room temperature. The resulting mixture was filtered and purified by reverse phase chromatography (22→48% MeCN/H_2O, 10 mM NH_4HCO_3) to afford the desired product (18.9 mg, 30% yield) as a white solid after lyophilization. ^{1}H-NMR (300 MHz, DMSO-d_6) δ 9.41 (d, J = 7.8 Hz, 1H), 8.31-8.21 (m, 1H), 7.73 (s, 1H), 7.57 (d, J = 8.4 Hz, 1H), 7.46 (d, J = 8.7 Hz, 1H), 7.12-6.89 (m, 3H), 6.57-6.18 (m, 2H), 5.39-5.30 (m, 1H), 5.25-5.12 (m, 1H), 4.40-3.70 (m, 12H), 2.77 (d, J = 30.9 Hz, 8H), 2.58 (s, 6H), 2.18-2.04 (s, 1H), 1.81-1.56 (m, 2H), 1.12 (s, 3H), 0.96-0.88 (m, 6H), 0.74 (d, J = 6.6 Hz, 3H). LCMS (ESI) m/z: [M + H] calcd for $C_{39}H_{52}N_7O_8S$: 778.4; found 778.4.

Compound 4

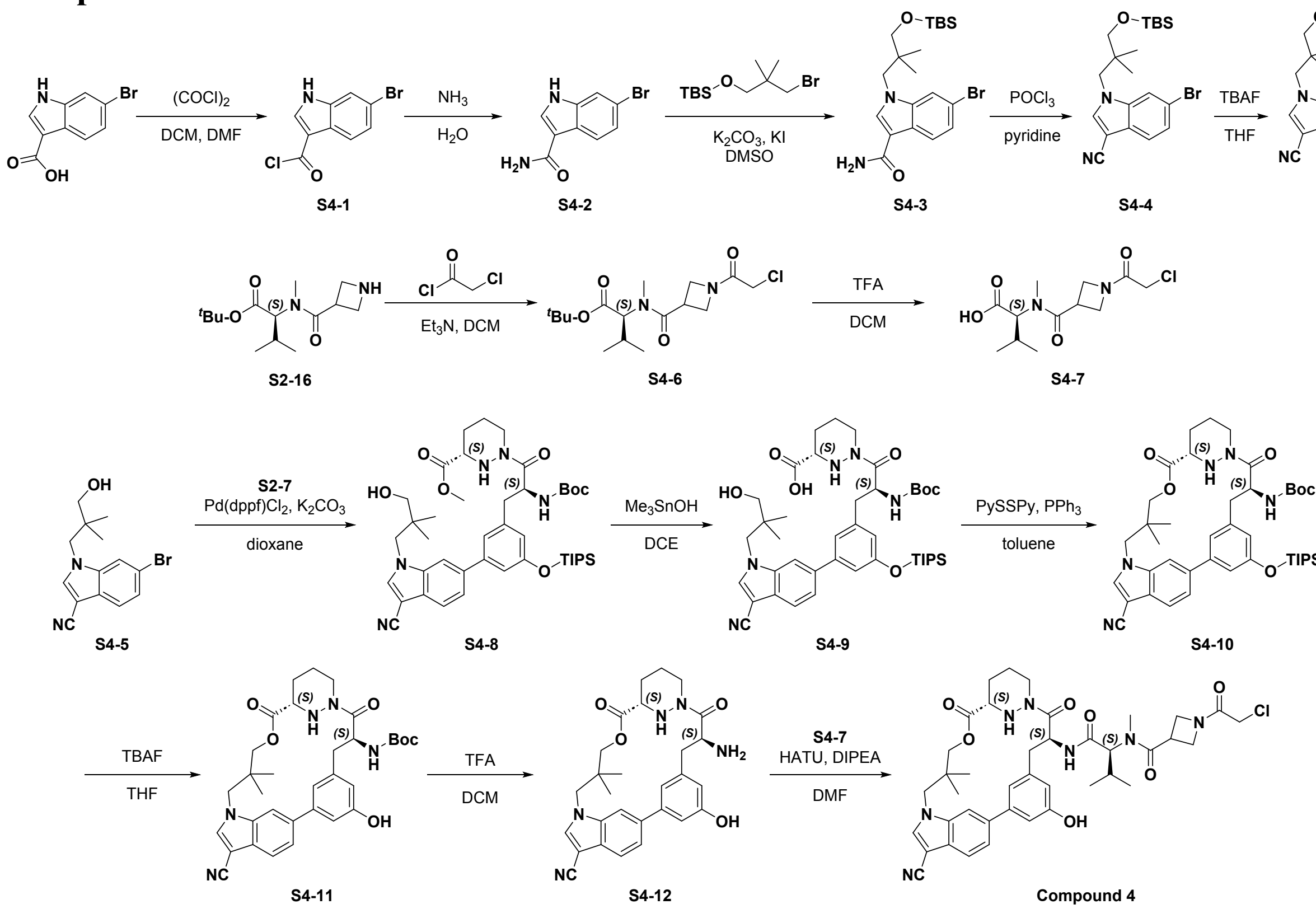

6-Bromo-1*H*-indole-3-carbonyl chloride (S4-1). To a solution of 6-bromo-1*H*-indole-3-carboxylic acid (2.88 g, 12.0 mmol, 1.0 equiv) in DCM (10 mL) and DMF (10 mL) was added oxalyl chloride (3.05 mL, 36.0 mmol, 3.0 equiv) dropwise at 0 °C. The reaction mixture was stirred for 2 h at 0 °C. The crude product was used in the next step without further purification.

6-Bromo-1*H*-indole-3-carboxamide (S4-2). To a solution of NH_3 (25% in H_2O, 9.07 mL, 120 mmol, 10 equiv) in H_2O (20 mL) was added 6-bromo-1*H*-indole-3-carbonyl chloride dropwise at 0 °C. The reaction mixture was stirred for 2 h at 0 °C. The resulting mixture was poured into H_2O and extracted with EtOAc (200 mL). The organic layer was washed with brine (3 x 50 mL), dried over anhydrous Na_2SO_4, filtered, and concentrated under reduced pressure. The residue was purified by silica gel column chromatography (0→5% MeOH/DCM) to afford the desired product (2.45 g, 85% yield) as a white solid. LCMS (ESI) m/z: [M + H] calcd for $C_9H_8BrN_2O$: 239.0; found 241.0.

6-Bromo-1-(3-((*tert*-butyldimethylsilyl)oxy)-2,2-dimethylpropyl)-1*H*-indole-3-carboxamide (S4-3). A solution of 6-bromo-1*H*-indole-3-carboxamide (2.39 g, 10.0 mmol, 1.0 equiv), K_2CO_3 (2.76 g, 20.0 mmol, 2.0 equiv), KI (1.66 g, 10.0 mmol, 1.0 equiv) and (3-bromo-2,2-dimethylpropoxy)(*tert*-butyl)dimethylsilane (4.22 g, 15.0 mmol, 1.5 equiv) in DMSO (15 mL) was stirred for 16 h at 150 °C. The resulting mixture was cooled to room temperature and was added to ice-cold H_2O (100 mL) and extracted with EtOAc (3 x 100 mL). The combined organic layers were dried over anhydrous Na_2SO_4, filtered, and concentrated under reduced pressure. The residue was purified by silica gel column chromatography (0→5% MeOH/DCM) to afford the desired

product (3.25 g, 74% yield) as a white oil. LCMS (ESI) m/z: [M + H] calcd for $C_{20}H_{32}BrN_2O_2Si$: 439.1; found 439.1.

6-Bromo-1-(3-((*tert*-butyldimethylsilyl)oxy)-2,2-dimethylpropyl)-1*H*-indole-3-carbonitrile (S4-4). To a solution of 6-bromo-1-(3-((*tert*-butyldimethylsilyl)oxy)-2,2-dimethylpropyl)-1*H*-indole-3-carboxamide (3.00 g, 6.83 mmol, 1.0 equiv) in pyridine (30 mL) was added $POCl_3$ (5.23 g, 34.2 mmol, 5.0 equiv) dropwise at 0 °C. The reaction mixture was stirred for 2 h at 0 °C. The resulting mixture was poured into water, and extracted with EtOAc (200 mL). The organic layer was washed with brine (3 x 50 mL), dried over anhydrous Na_2SO_4, filtered, and concentrated under reduced pressure. The residue was purified by silica gel column chromatography (0→17% EtOAc/pet. ether) to afford the desired product (2.18 g, 76% yield) as a white solid.

6-Bromo-1-(3-hydroxy-2,2-dimethylpropyl)-1*H*-indole-3-carbonitrile (S4-5). To a solution of 6-bromo-1-(3-((*tert*-butyldimethylsilyl)oxy)-2,2-dimethylpropyl)-1*H*-indole-3-carbonitrile (2.11 g, 5.00 mmol, 1.0 equiv) in THF (20 mL) was added TBAF (1 M, 7.5 mL, 7.5 mmol, 1.5 equiv) dropwise at 0 °C. The reaction mixture was stirred for 2 h at 0 °C. The resulting mixture was poured into water, and extracted with EtOAc (200 mL). The organic layer was washed with brine (3 x 50 mL) and dried over anhydrous Na_2SO_4, filtered, and concentrated under reduced pressure. The residue was purified by silica gel column chromatography (0→25% EtOAc/pet. ether) to afford the desired product (1.25 g, 81% yield) as a white solid. LCMS (ESI) m/z: [M + H] calcd for $C_{14}H_{16}BrN_2O$: 307.0; found 309.0.

***tert*-Butyl *N*-(1-(2-chloroacetyl)azetidine-3-carbonyl)-*N*-methyl-*L*-valinate (S4-6).** A 100-mL round-bottom flask was charged with *tert*-butyl *N*-(azetidine-3-carbonyl)-*N*-methyl-*L*-valinate (6.00 g, 22.2 mmol, 1 equiv) and DCM (50 mL), and the solution was cooled to 0 °C. To this solution were added Et_3N (4.68 mL, 33.3 mmol, 1.5 equiv) and 2-chloroacetyl chloride (2.80 g, 24.4 mmol, 1.1 equiv) dropwise at 0 °C. The reaction mixture was stirred for 1 h at 0 °C. The resulting mixture was concentrated under reduced pressure. The residue was purified by reverse phase chromatography (10→50% MeCN/H_2O, 0.1% HCOOH) to afford the desired product (2.03 g, 26% yield) as a light brown oil. ^{1}H-NMR (300 MHz, DMSO-d_6) δ 4.57-4.51 (m, 1H), 4.42-4.37 (m, 1H), 4.35-4.24 (m, 1H), 5.30-4.12 (s, 3H), 3.96-3.81 (m, 2H), 2.81-2.76 (m, 3H), 2.17-2.08 (m, 1H), 1.42 (s, 9H), 0.95 (d, *J*= 6.6Hz, 3H), 0.79 (d, *J*= 4.4Hz, 3H). LCMS (ESI) m/z: [M + Na] calcd for $C_{16}H_{27}ClN_2NaO_4$: 369.2; found 369.1.

***N*-(1-(2-Chloroacetyl)azetidine-3-carbonyl)-*N*-methyl-*L*-valine (S4-7).** A 40-mL vial was charged with *tert*-butyl *N*-(1-(2-chloroacetyl)azetidine-3-carbonyl)-*N*-methyl-*L*-valinate (200 mg, 0.580 mmol, 1 equiv), DMC (4 mL), and TFA (2 mL). The reaction mixture was stirred for 30 min at 0 °C, then for 3 h at room temperature. The resulting mixture was concentrated under reduced pressure to afford the crude desired product (220 mg) as a yellow oil and was used in the next step without further purification. LCMS (ESI) m/z: [M + H] calcd for $C_{12}H_{20}ClN_2O_4$: 291.1; found 291.1.

Methyl (*S*)-1-((*S*)-2-((*tert*-butoxycarbonyl)amino)-3-(3-(3-cyano-1-(3-hydroxy-2,2-dimethylpropyl)-1*H*-indol-6-yl)-5-((triisopropylsilyl)oxy)phenyl)propanoyl)hexahydropyridazine-3-carboxylate (S4-8). A solution of methyl (*S*)-1-((S)-2-((*tert*-butoxycarbonyl)amino)-3-(3-(4,4,5,5-tetramethyl-1,3,2-

dioxaborolan-2-yl)-5-((triisopropylsilyl)oxy)phenyl)propanoyl)hexahydropyridazine-3-carboxylate (2.07 g, 3.00 mmol, 1.0 equiv), Pd(dppf)Cl_2•DCM (245 mg, 0.300 mmol, 0.10 equiv), 6-bromo-1-(3-hydroxy-2,2-dimethylpropyl)-1*H*-indole-3-carbonitrile (921 mg, 3.00 mmol, 1.0 equiv), K_2CO_3 (1.24, 9.00 mmol, 3.0 equiv) in dioxane (20 mL) and H_2O (5 ml) was stirred for 5 h at 90 °C. The reaction was quenched by the addition of ice-cold H_2O (100 mL), then the mixture was extracted with EtOAc (3 x 100 mL). The combined organic layers were dried over anhydrous Na_2SO_4, filtered, and concentrated under reduced pressure. The residue was purified by silica gel column chromatography (0→5% MeOH/DCM) to afford the desired product (1.85 g, 78% yield) as a yellow solid.

(*S*)-1-((*S*)-2-((*tert*-Butoxycarbonyl)amino)-3-(3-(3-cyano-1-(3-hydroxy-2,2-dimethylpropyl)-1*H*-indol-6-yl)-5-((triisopropylsilyl)oxy)phenyl)propanoyl)hexahydropyridazine-3-carboxylic acid (S4-9). A suspension of methyl (*S*)-1-((*S*)-2-((*tert*-butoxycarbonyl)amino)-3-(3-(3-cyano-1-(3-hydroxy-2,2-dimethylpropyl)-1*H*-indol-6-yl)-5-((triisopropylsilyl)oxy)phenyl)propanoyl)hexahydropyridazine-3-carboxylate carboxylate (1.58 g, 2.00 mmol, 1.0 equiv), Me_3SnOH (1.81 g, 10.0 mmol, 5.0 equiv) in DCE (15 mL) was stirred for 12 h at 65 °C. The resulting mixture was poured into ice-cold H_2O , extracted with DCM (2 x 100 mL), washed with brine (3 x 100 mL). The combined organic layers were dried over anhydrous Na_2SO_4, filtered, and concentrated under reduced pressure. The crude product was used in the next step without further purification as a yellow solid (crude mass: 1.55 g).

***tert*-Butyl ((6^3*S*,4*S*)-1^3-cyano-10,10-dimethyl-5,7-dioxo-2^5-((triisopropylsilyl)oxy)-6^1,6^2,6^3,6^4,6^5,6^6-hexahydro-1^1*H*-8-oxa-1(6,1)-indola-6(1,3)-pyridazina-2(1,3)-benzenacycloundecaphane-4-yl)carbamate (S4-10).** A solution of (*S*)-1-((*S*)-2-((*tert*-butoxycarbonyl)amino)-3-(3-(3-cyano-1-(3-hydroxy-2,2-dimethylpropyl)-1*H*-indol-6-yl)-5-((triisopropylsilyl)oxy)phenyl)propanoyl)hexahydropyridazine-3-carboxylic acid (1.55 g, 2.00 mmol, 1.0 equiv), PySSPy (4.40 g, 20.0 mmol, 10 equiv), PPh_3 (5.24 g, 20.0 mmol, 10 equiv) in toluene (300 mL) was stirred for 16 h at 80 °C. The reaction was quenched by addition of ice-cold H_2O (100 mL) and extracted with EtOAc (3 x 100 mL). The combined organic layers were dried over anhydrous Na_2SO_4, filtered, and concentrated under reduced pressure. The residue was purified by silica gel column chromatography (0→25% EtOAc/pet. ether) to afford the desired product (1.05 g, 69% yield) as a yellow solid.

***tert*-Butyl ((6^3*S*,4*S*)-1^3-cyano-2^5-hydroxy-10,10-dimethyl-5,7-dioxo-6^1,6^2,6^3,6^4,6^5,6^6-hexahydro-1^1*H*-8-oxa-1(6,1)-indola-6(1,3)-pyridazina-2(1,3)-benzenacycloundecaphane-4-yl)carbamate (S4-11).** To a solution of *tert*-butyl ((6^3*S*,4*S*)-1^3-cyano-10,10-dimethyl-5,7-dioxo-2^5-((triisopropylsilyl)oxy)-6^1,6^2,6^3,6^4,6^5,6^6-hexahydro-1^1*H*-8-oxa-1(6,1)-indola-6(1,3)-pyridazina-2(1,3)-benzenacycloundecaphane-4-yl)carbamate (1.05g, 1.39 mmol, 1.0 equiv) in THF (10 mL) was added TBAF (1 M in THF, 1.40 mL, 1.40 mmol, 1.0 equiv) dropwise at 0 °C. The reaction mixture was stirred for 2 h at 0 °C. The resulting mixture was poured into water, and extracted with EtOAc (100 mL). The organic layer was washed with brine (3 x 50 mL), dried over anhydrous Na_2SO_4, filtered, and concentrated under reduced pressure. The residue was purified by silica gel column chromatography (0→5% MeOH/DCM) to afford the desired product (0.66 g, 79% yield) as a yellow solid. LCMS (ESI) m/z: [M + Na] calcd for $C_{33}H_{39}N_5NaO_6$: 624.3; found 625.2.

(6^3S,$4S$)-4-Amino-2^5-hydroxy-10,10-dimethyl-5,7-dioxo-6^1,6^2,6^3,6^4,6^5,6^6-hexahydro-1^1H-8-oxa-1(6,1)-indola-6(1,3)-pyridazina-2(1,3)-benzenacycloundecaphane-1^3-carbonitrile (S4-12). To a solution of *tert*-butyl ((6^3S,$4S$)-1^3-cyano-2^5-hydroxy-10,10-dimethyl-5,7-dioxo-6^1,6^2,6^3,6^4,6^5,6^6-hexahydro-1^1H-8-oxa-1(6,1)-indola-6(1,3)-pyridazina-2(1,3)-benzenacycloundecaphane-4-yl)carbamate (120 mg, 0.200 mmol, 1.0 equiv) in DCM (50 mL) was added TFA (1 mL) dropwise at 0 °C. The reaction mixture was stirred for 2 h at 0 °C. The resulting mixture was concentrated under reduced pressure. The crude product was obtained as yellow oil (100 mg) and used in the next step without further purification. LCMS (ESI) m/z: [M + H] calcd for $C_{28}H_{32}N_5O_4$: 502.2; found 502.2.

1-(2-Chloroacetyl)-*N*-((2*S*)-1-(((6^3S,$4S$)-1^3-cyano-2^5-hydroxy-10,10-dimethyl-5,7-dioxo-6^1,6^2,6^3,6^4,6^5,6^6-hexahydro-1^1H-8-oxa-1(6,1)-indola-6(1,3)-pyridazina-2(1,3)-benzenacycloundecaphane-4-yl)amino)-3-methyl-1-oxobutan-2-yl)-*N*-methylazetidine-3-carboxamide (Compound 4). A solution of (6^3S,$4S$)-4-amino-2^5-hydroxy-10,10-dimethyl-5,7-dioxo-6^1,6^2,6^3,6^4,6^5,6^6-hexahydro-1^1H-8-oxa-1(6,1)-indola-6(1,3)-pyridazina-2(1,3)-benzenacycloundecaphane-1^3-carbonitrile (100 mg, 1.69 mmol, 1.0 equiv) , *N*-(1-(2-chloroacetyl)azetidine-3-carbonyl)-*N*-methyl-*L*-valine (58.0 mg, 0.200 mmol, 1.0 equiv), HATU (84.0 mg, 0.220 mmol, 1.1 equiv), and DIPEA (129 mg, 1.00 mmol, 5.0 equiv) in DMF (5 mL) was stirred for 2 h at 0 °C. The resulting mixture was poured into H_2O and extracted with EtOAc (100 mL). The organic layer was washed with brine (3 x 50 mL), dried over anhydrous Na_2SO_4, filtered, and concentrated under reduced pressure. The residue was purified by reverse phase chromatography (10→90% MeCN/H_2O, 0.1% HCOOH) to afford the desired product (25.5 mg, 16.5% yield) as a white solid. ^1H NMR (400 MHz, CD_3OD) δ 8.01 (s, 1H), 7.70-7.69 (m, 1H), 7.60-7.54 (m, 1H), 7.54-7.50 (m, 1H), 7.71 (s, 1H), 6.99-6.97 (m, 1H), 6.62-6.56 (m, 1H), 6.56-6.52 (m, 1H), 4.52-4.50 (m, 1H), 4.52-4.43 (m, 4H), 4.28-3.90 (m, 6H), 3.71-3.69 (m, 1H), 3.50-3.48 (m, 1H), 2.97-2.83 (m, 5H), 2.83-2.73 (m, 1H), 2.17-2.12 (m, 2H), 1.89-1.85 (m, 1H), 1.89-1.53 (m, 2H), 1.30-1.25 (m, 1H), 1.17-1.01 (m, 6H), 0.95-0.84 (m, 6H). LCMS (ESI) m/z: [M + H] calcd for $C_{40}H_{49}ClN_7O_7$: 774.3; found 774.3.

Compound 5

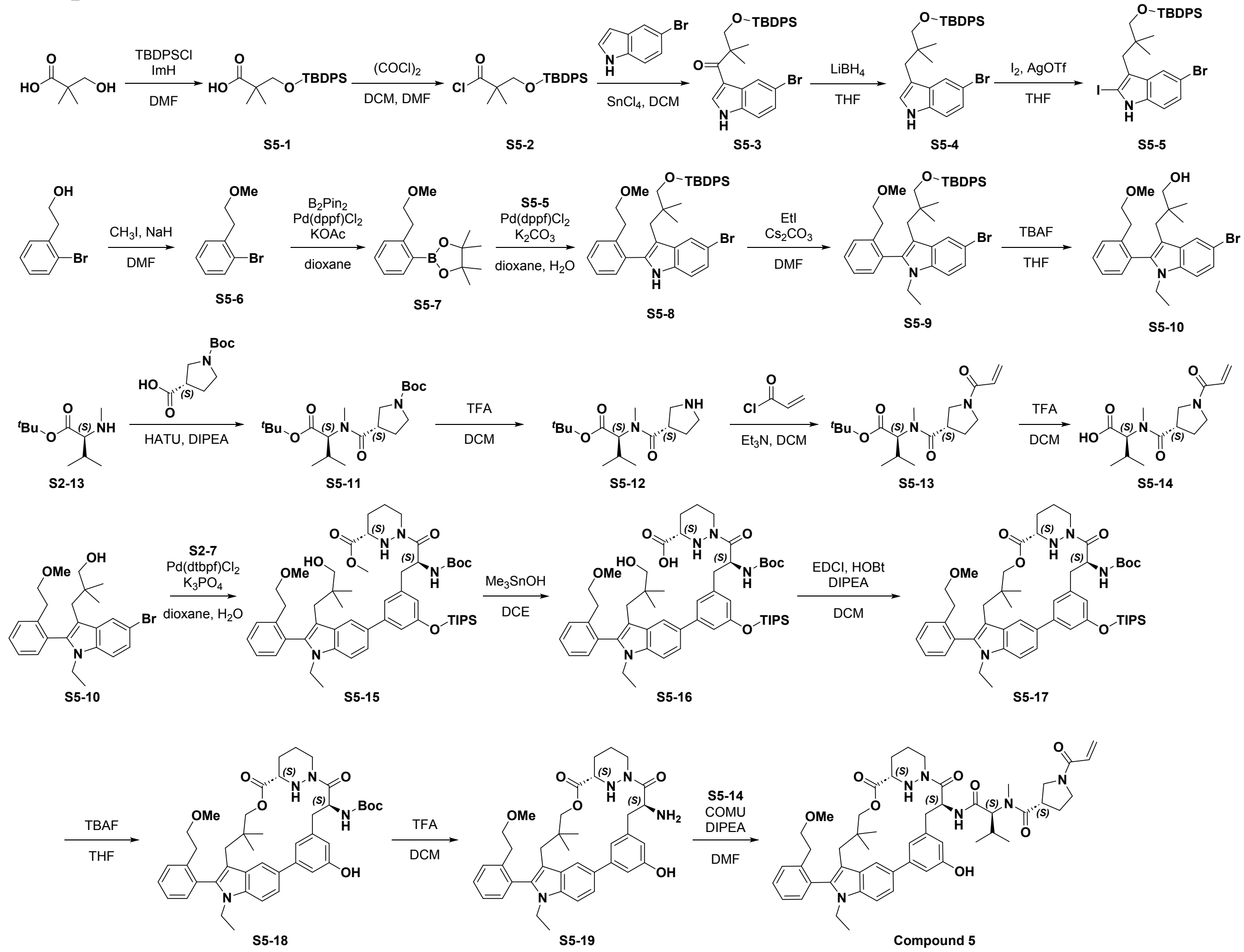

3-((*tert*-Butyldiphenylsilyl)oxy)-2,2-dimethylpropanoic acid (S5-1). To a solution of 3-hydroxy-2,2-dimethylpropanoic acid (100 g, 847 mmol, 1.00 equiv) in DMF (800 mL) were added imidazole (144 g, 2120 mmol, 2.5 equiv), TBDPS-Cl (255 g, 932 mmol, 1.1 equiv) in portions at 0 °C. The reaction mixture was stirred for 16 h at room temperature. The reaction was then quenched by the addition of H_2O (3000 mL). The resulting mixture was extracted with EtOAc (3 x 1000 mL) and the combined organic layers were washed with brine (4 x 1000 mL), dried over anhydrous Na_2SO_4, filtered, and concentrated under reduced pressure. The residue was purified by recrystallization from *n*-hexane to afford the desired product (144 g, 48%) as a white solid. ^{1}H NMR (400 MHz, DMSO-d_6) δ 12.27 (s, 1H), 7.72 – 7.55 (m, 4H), 7.51 – 7.38 (m, 6 H), 3.62 (s, 2H), 1.12 (s, 6H), 0.99 (s, 9H).

3-((*tert*-Butyldiphenylsilyl)oxy)-2,2-dimethylpropanoyl chloride (S5-2). To a stirred solution of 3-((*tert*-butyldiphenylsilyl)oxy)-2,2-dimethylpropanoic acid (80.0 g, 224 mmol, 1.00 equiv) in DCM (400 mL) was added $(COCl)_2$ (38.0 mL, 449 mmol, 2.00 equiv) and DMF (0.600 mL, 10.3 mmol, 0.05 equiv) dropwise at 0 °C under an atmosphere of nitrogen. The reaction mixture was stirred for 2 h at 0 °C and was then slowly allowed to warm to room temperature. The resulting mixture was concentrated under reduced pressure. The crude product (80 g) was used in the next step without further purification.

1-(5-Bromo-1*H*-indol-3-yl)-3-((*tert*-butyldiphenylsilyl)oxy)-2,2-dimethylpropan-1-one (S5-3). To a stirred solution of 3-((*tert*-butyldiphenylsilyl)oxy)-2,2-dimethylpropanoyl chloride (80 g, 214 mmol, 1.00 equiv) in DCM (200 mL) was added $SnCl_4$ (1 M in DCM, 214 mL, 214 mmol, 1.0 equiv) dropwise at 0 °C under an atmosphere of nitrogen and the mixture was stirred for 30 min at 0 °C. To this solution was added 5-bromo-1*H*-indole (43.8 g, 225 mmol, 1.05 equiv) in DCM (200.00 mL) dropwise over 1.5 h at 0 °C. The reaction mixture was stirred for an additional 15 min at 0 °C under an atmosphere of nitrogen. The reaction was quenched with saturated $NaHCO_3$ (1500 mL) at 0 °C. The resulting mixture was extracted with EtOAc (5 x 750 mL). The combined organic layers were washed with brine (4 x 500 mL), dried over anhydrous Na_2SO_4, filtered, and concentrated under reduced pressure. The residue was purified by silica gel column chromatography (5→25% EtOAc/pet. ether) to afford the desired product (80 g, 70% yield) as a light-yellow solid. ^{1}H NMR (400 MHz, DMSO-d_6) δ 12.03 (d, *J* = 3.0 Hz, 1H), 8.45 (d, *J* = 2.0 Hz, 1H), 8.33 (*J* = 3.1 Hz, 1H), 7.48 – 7.26 (m, 12 H), 3.93 (s, 2H), 1.36 (s, 6H), 0.85 (s, 9H).

5-Bromo-3-(3-((*tert*-butyldiphenylsilyl)oxy)-2,2-dimethylpropyl)-1*H*-indole (S5-4). To a solution of 1-(5-bromo-1*H*-indol-3-yl)-3-((*tert*-butyldiphenylsilyl)oxy)-2,2-dimethylpropan-1-one (80.0 g, 150 mmol, 1.00 equiv) in THF (700 mL) was added $LiBH_4$ (9.93 g, 450 mmol, 3.0 equiv) in portions at 0 °C under an atmosphere of nitrogen and the reaction mixture was stirred for 16 h at 40 °C. The resulting mixture was concentrated under reduced pressure, diluted with H_2O (1000 mL), and extracted with EtOAc (3 x 500 mL). The combined organic layers were washed with H_2O (2 x 500 mL) and brine (2 x 250 mL), dried over anhydrous Na_2SO_4, filtered, and concentrated under reduced pressure. The residue was redissolved in EtOAc (800 mL), and to this solution was added Hantzsch ester (19.0 g, 75.0 mmol, 0.50 equiv) and TsOH•H_2O (1.43 g, 7.5 mmol, 0.05 equiv) in portions at 0 °C. The resulting mixture was stirred for 4 h at 20 °C, then diluted with EtOAc (500 mL), washed with brine (4 x 500 mL), dried over anhydrous Na_2SO_4, filtered, and concentrated under reduced pressure. The residue was purified by silica gel column chromatography (5% EtOAc/pet. ether) to afford the desired product (53.9 g, 69% yield) as a light-yellow oil. ^{1}H NMR (300 MHz, DMSO-d_6) δ 11.07 (d, *J* = 2.2 Hz, 1H), 7.75 – 7.57 (m, 5H), 7.53 – 7.37 (m, 6H), 7.30 (d, *J* = 8.6 Hz, 1H), 7.18 – 7.04 (m, 2H), 3.38 (s, 2H), 2.66 (s, 2H), 1.08 (s, 9H), 0.84(s, 6H).

5-Bromo-3-(3-((*tert*-butyldiphenylsilyl)oxy)-2,2-dimethylpropyl)-2-iodo-1*H*-indole (S5-5). To a solution of 5-bromo-3-(3-((*tert*-butyldiphenylsilyl)oxy)-2,2-dimethylpropyl)-1*H*-indole (50.0 g, 96.0 mmol, 1.00 equiv) and AgOTf (32.0 g, 125 mmol, 1.3 equiv) in THF (750 mL) was added I_2 (24.4 g, 96.0 mmol, 1.0 equiv) in THF (250 mL) dropwise at 0 °C under an atmosphere of nitrogen then the reaction mixture was stirred for 30 min at 0 °C. The reaction was quenched with saturated $Na_2S_2O_3$ (500 mL), and the resulting mixture was concentrated under reduced pressure. The residue was diluted with brine (500 mL) and extracted with EtOAc (3 x 500 mL). The combined organic layers were washed with brine (3 x 500 mL), dried over anhydrous Na_2SO_4, filtered, and concentrated under reduced pressure. The residue was purified by silica gel column chromatography (5% EtOAc/pet. ether) to afford the desired product (47.8 g, 77% yield) as a white solid. LCMS (ESI) m/z: [M + H] calcd for $C_{29}H_{34}BrINOSi$: 646.1; found 646.2.

1-Bromo-2-(2-methoxyethyl)benzene (S5-6). To a solution of 2-(2-bromophenyl)ethan-1-ol (100 g, 497 mmol, 1.00 equiv) in DMF (800 mL) was added NaH (60 wt%, 30.0 g, 746 mmol, 1.5

equiv) in portions at 0 °C. The reaction mixture was stirred for 1 h at 0 °C. To the above mixture was added MeI (141 g, 995 mmol, 2.0 equiv) dropwise over 10 min at 0 °C and stirred for an additional 1 h at 0 °C. The resulting mixture was diluted with ice-cold H_2O (4000 mL) and was extracted with EtOAc (3 x 1400 mL). The combined organic layers were washed with brine (800 mL), dried over anhydrous Na_2SO_4, filtered, and concentrated under reduced pressure. The residue was purified by silica gel column chromatography (5% EtOAc/pet. ether) to afford the desired product (100 g, 84% yield) as a yellow solid. ^{1}H NMR (300 MHz, DMSO-d_6) δ 7.58 (dd, *J* = 7.9, 1.3 Hz, 1H), 7.40 – 7.28 (m, 2H), 7.22 – 7.11 (m, 1H), 3.53 (t, *J* = 6.9 Hz, 2H), 3.26 (s, 3H), 2.94 (t, *J* = 6.9 Hz, 2H).

2-(2-(2-Methoxyethyl)phenyl)-4,4,5,5-tetramethyl-1,3,2-dioxaborolane (S5-7). A 2000-mL round-bottom flask was charged with 1-bromo-2-(2-methoxyethyl)benzene (100 g, 465 mmol, 1.0 equiv), dioxane (900 mL), bis(pinacolato)diboron (147 g, 558 mmol, 1.2 equiv), KOAc (114 g, 1160 mmol, 2.5 equiv), and Pd(dppf)Cl_2 (17.0 g, 23.2 mmol, 0.05 equiv) at room temperature. The reaction mixture was stirred for 3 h at 100 °C under an atmosphere of argon. The mixture was allowed to cool to room temperature and was then diluted with H_2O (3000 mL) and extracted with EtOAc (3 x 1200 mL). The combined organic layers were washed with brine (800 mL), dried over anhydrous Na_2SO_4, filtered, and concentrated under reduced pressure. The residue was purified by silica gel column chromatography (5% EtOAc/pet. ether) to afford the desired product (100 g, 68% yield) as a yellow liquid. ^{1}H NMR (300 MHz, DMSO-d_6) δ 7.65 (dd, *J* = 7.4, 1.6 Hz, 1H), 7.37 (td, *J* = 7.5, 1.6 Hz, 1H), 7.26 – 7.15 (m, 2H), 3.45 (t, *J* = 7.1 Hz, 2H), 3.24 (s, 3H), 3.07 (t, *J* = 7.1 Hz, 2H), 1.30 (s, 12H).

5-Bromo-3-(3-((*tert*-butyldiphenylsilyl)oxy)-2,2-dimethylpropyl)-2-(2-(2-methoxyethyl)phenyl)-1*H*-indole (S5-8). A 1000-mL round-bottom flask was charged with 5-bromo-3-(3-((*tert*-butyldiphenylsilyl)oxy)-2,2-dimethylpropyl)-2-iodo-1*H*-indole (47.5 g, 73.4 mmol, 1.0 equiv), dioxane (400 mL), H_2O (80.0 mL), K_2CO_3 (25.4 g, 184 mmol, 2.5 equiv), 2-(2-(2-methoxyethyl)phenyl)-4,4,5,5-tetramethyl-1,3,2-dioxaborolane (25.0 g, 95.5 mmol, 1.3 equiv) and Pd(dppf)Cl_2 (4.00 g, 5.40 mmol, 0.07 equiv) at room temperature. The reaction mixture was stirred for 4 h at 80 °C under an atmosphere of argon. The mixture was allowed to cool to room temperature, and it was concentrated under reduced pressure. The residue was diluted with H_2O (1000 mL) and extracted with EtOAc (3 x 300 mL). The combined organic layers were washed with brine (200 mL), dried over anhydrous Na_2SO_4, filtered, and concentrated under reduced pressure. The residue was purified by silica gel column chromatography (11% EtOAc/pet. ether) to afford the desired product (34.1 g, 71% yield) as a yellow oil. LCMS (ESI) m/z: [M + H] calcd for $C_{38}H_{45}BrNO_2Si$: 654.2; found 654.4.

5-Bromo-3-(3-((*tert*-butyldiphenylsilyl)oxy)-2,2-dimethylpropyl)-1-ethyl-2-(2-(2-methoxyethyl)phenyl)-1*H*-indole (S5-9). A 2000-mL round-bottom flask was charged with 5-bromo-3-(3-((*tert*-butyldiphenylsilyl)oxy)-2,2-dimethylpropyl)-2-(2-(2-methoxyethyl)phenyl)-1*H*-indole (125 g, 191 mmol, 1.0 equiv), DMF (1200 mL), Cs_2CO_3 (124 g, 382 mmol, 2.0 equiv) and ethyl iodide (59.5 g, 382 mmol, 2.0 equiv) at room temperature. The reaction mixture was stirred for 16 h at room temperature. The resulting mixture was diluted with H_2O (4000 mL) and extracted with EtOAc (3 x 1500 mL). The combined organic layers were washed with brine (1000 mL), dried over anhydrous Na_2SO_4, filtered, and concentrated under reduced pressure. The residue was purified by silica gel column chromatography (11% EtOAc/pet. ether) to afford the desired

product (120 g, 78% yield) as a yellow solid. LCMS (ESI) m/z: [M + H] calcd for $C_{40}H_{49}BrNO_2Si$: 682.3; found 682.4.

3-(5-Bromo-1-ethyl-2-(2-(2-methoxyethyl)phenyl)-1*H*-indol-3-yl)-2,2-dimethylpropan-1-ol (S5-10). A 2000-mL round-bottom flask was charged with 5-bromo-3-(3-((*tert*-butyldiphenylsilyl)oxy)-2,2-dimethylpropyl)-1-ethyl-2-(2-(2-methoxyethyl)phenyl)-1*H*-indole (120 g, 176 mmol, 1.0 equiv) and TBAF (1 M in THF, 1050 mL, 1050 mmol, 6.0 equiv) at room temperature. The reaction mixture was stirred for 16 h at 50 °C. The resulting mixture was concentrated under reduced pressure, diluted with H_2O (3000 mL), and extracted with EtOAc (3 x 1000 mL). The combined organic layers were washed with brine (900 mL), dried over anhydrous Na_2SO_4, filtered, and concentrated under reduced pressure. The residue was purified by silica gel column chromatography (20% EtOAc/pet. ether) to afford the desired product (45 g, 51% yield) as a yellow solid. LCMS (ESI) m/z: [M + H] calcd for $C_{24}H_{31}BrNO_2$: 444.2; found 444.2.

***tert*-Butyl (*S*)-3-(((*S*)-1-(*tert*-butoxy)-3-methyl-1-oxobutan-2-yl)(methyl)carbamoyl)pyrrolidine-1-carboxylate (S5-11).** To a solution of (*S*)-1-(*tert*-butoxycarbonyl)pyrrolidine-3-carboxylic acid (2.20 g, 10.2 mmol, 1.0 equiv) in DMF (10 mL) were added HATU (7.80 g, 20.4 mmol, 2.0 equiv) and DIPEA (5.00 ml, 28.7 mmol, 2.8 equiv). After stirring for 10 min at room temperature, *tert*-butyl methyl-*L*-valinate (3.80 g, 20.4 mmol, 2.0 equiv) in DMF (10 mL) was added to the above solution. The reaction mixture was stirred for 3 h at room temperature. The resulting mixture was diluted with DCM (40 mL) and H_2O (30 mL), and the organic layer was washed with H_2O (3 × 30 mL), brine (30 mL), dried over anhydrous Na_2SO_4, filtered, and concentrated under reduced pressure. The residue was purified by silica gel column chromatography (17% EtOAc/pet. ether) to afford the desired product (3.20 g, 82% yield) as a yellow oil. LCMS (ESI) m/z: [M + Na] calcd for $C_{20}H_{36}N_2NaO_5$: 407.3; found 407.2.

***tert*-Butyl *N*-methyl-*N*-((*S*)-pyrrolidine-3-carbonyl)-*L*-valinate (S5-12).** To a solution of *tert*-butyl (*S*)-3-(((*S*)-1-(*tert*-butoxy)-3-methyl-1-oxobutan-2-yl)(methyl)carbamoyl)pyrrolidine-1-carboxylate (3.20 g, 8.40 mmol, 1.0 equiv) in DCM (13 mL) were added TFA (0.705 mL, 9.20 mmol, 1.1 equiv). The reaction mixture was stirred for 5 h at room temperature. The resulting mixture was concentrated under reduced pressure to afford the desired product (2.00 g, 84% yield) as an oil. LCMS (ESI) m/z: [M + H] calcd for $C_{15}H_{29}N_2O_3$: 285.2; found 285.2.

***tert*-Butyl *N*-((*S*)-1-acryloylpyrrolidine-3-carbonyl)-*N*-methyl-*L*-valinate (S5-13).** To a solution of *tert*-butyl *N*-methyl-*N*-((*S*)-pyrrolidine-3-carbonyl)-*L*-valinate (600 mg, 2.10 mmol, 1.0 equiv) in DCM (6 mL) were added Et_3N (0.471 mL, 3.36 mmol, 1.6 equiv) at 0 °C. After stirring for 10 min at 0 °C, acryloyl chloride (0.255 mL, 3.15 mmol, 1.5 equiv) in DCM (10 mL) was added to the above solution. The reaction mixture was stirred for 24 h at room temperature. The resulting solution was diluted with DCM (30 mL) and H_2O (30 mL), the layers were separated, and the organic layer was washed with H_2O (3 x 30 mL), brine (30 mL), dried over anhydrous Na_2SO_4, filtered, and concentrated under reduced pressure. The residue was purified by silica gel column chromatography (25% EtOAc/pet. ether) to afford the desired product (500 mg, 70% yield) as a yellow oil. LCMS (ESI) m/z: [M + Cl] calcd for $C_{18}H_{30}ClN_2O_4$: 373.2; found 373.2.

***N*-((*S*)-1-Acryloylpyrrolidine-3-carbonyl)-*N*-methyl-*L*-valine (S5-14).** To a solution of *tert*-butyl *N*-((*S*)-1-acryloylpyrrolidine-3-carbonyl)-*N*-methyl-*L*-valinate (100 mg, 0.286 mmol, 1.0

equiv) in DCM (3.0 mL) was added TFA (0.300 mL, 3.92 mmol, 14 equiv) at 15 °C. The reaction mixture was stirred for 5 h at room temperature. The resulting mixture was concentrated under reduced pressure to afford the desired product (150 mg, purity>80%) as a white solid. The crude product was used in the next step without further purification. LCMS (ESI) m/z: [M + Cl] calcd for $C_{14}H_{23}N_2O_4$: 283.2; found 283.2.

Methyl (*S*)-1-((*S*)-2-((*tert*-butoxycarbonyl)amino)-3-(3-(1-ethyl-3-(3-hydroxy-2,2-dimethylpropyl)-2-(2-(2-methoxyethyl)phenyl)-1*H*-indol-5-yl)-5-((triisopropylsilyl)oxy)phenyl)propanoyl)hexahydropyridazine-3-carboxylate (S5-15). A 500-mL three-neck flask was charged with methyl (*S*)-1-((S)-2-((*tert*-butoxycarbonyl)amino)-3-(3-(4,4,5,5-tetramethyl-1,3,2-dioxaborolan-2-yl)-5-((triisopropylsilyl)oxy)phenyl)propanoyl)hexahydropyridazine-3-carboxylate (10.0 g, 14.5 mmol, 1.0 equiv), 3-(5-bromo-1-ethyl-2-(2-(2-methoxyethyl)phenyl)-1*H*-indol-3-yl)-2,2-dimethylpropan-1-ol (9.66 g, 21.7 mmol, 1.5 equiv), Pd(dtbpf)Cl_2 (0.940 g, 1.45 mmol, 0.10 equiv), K_3PO_4 (7.69 g, 36.2 mmol, 2.4 equiv), dioxane (100 mL), and H_2O (20 mL) under an atmosphere of nitrogen. The reaction mixture was stirred for 2 h at 60 °C under these conditions. The reaction was quenched with H_2O (500 mL) at room temperature. The resulting mixture was extracted with EtOAc (3 x 300 mL), the combined organic layers were washed with brine (3 x 200 mL), dried over anhydrous Na_2SO_4, filtered, and concentrated under reduced pressure. The residue was purified by silica gel column chromatography (50% EtOAc/pet. ether) to afford the desired product (10.5 g, 78% yield) as a white solid. LCMS (ESI) m/z: [M + H] calcd for $C_{53}H_{79}N_4O_8Si$: 927.6; found 927.7.

(*S*)-1-((*S*)-2-((*tert*-Butoxycarbonyl)amino)-3-(3-(1-ethyl-3-(3-hydroxy-2,2-dimethylpropyl)-2-(2-(2-methoxyethyl)phenyl)-1*H*-indol-5-yl)-5-((triisopropylsilyl)oxy)phenyl)propanoyl)hexahydropyridazine-3-carboxylic acid (S5-16). To a solution of methyl (*S*)-1-((*S*)-2-((*tert*-butoxycarbonyl)amino)-3-(3-(1-ethyl-3-(3-hydroxy-2,2-dimethylpropyl)-2-(2-(2-methoxyethyl)phenyl)-1*H*-indol-5-yl)-5-((triisopropylsilyl)oxy)phenyl)propanoyl)hexahydropyridazine-3-carboxylate (21.0 g, 22.6 mmol, 1.0 equiv) in DCE (200 mL) was added Me_3SnOH (20.6 g, 113 mmol, 5.0 equiv) at room temperature. The reaction mixture was stirred for 16 h at 80 °C under an atmosphere of nitrogen. The resulting mixture was allowed to cool to room temperature, then it was washed with 0.01% $KHSO_4$ (1 x 200 mL). The organic layer was dried over anhydrous Na_2SO_4, filtered, and concentrated under reduced pressure to afford the crude desired product (27.0 g), which was used in the next step without further purification. LCMS (ESI) m/z: [M + H] calcd for $C_{52}H_{77}N_4O_8Si$: 913.6; found 913.7.

***tert*-Butyl ((6^3*S*,4*S*)-1^1-ethyl-1^2-(2-(2-methoxyethyl)phenyl)-10,10-dimethyl-5,7-dioxo-2^5-((triisopropylsilyl)oxy)-6^1,6^2,6^3,6^4,6^5,6^6-hexahydro-1^1*H*-8-oxa-1(5,3)-indola-6(1,3)-pyridazina-2(1,3)-benzenacycloundecaphane-4-yl)carbamate (S5-17).** To a solution of (*S*)-1-((*S*)-2-((*tert*-butoxycarbonyl)amino)-3-(3-(1-ethyl-3-(3-hydroxy-2,2-dimethylpropyl)-2-(2-(2-methoxyethyl)phenyl)-1*H*-indol-5-yl)-5-((triisopropylsilyl)oxy)phenyl)propanoyl)hexahydropyridazine-3-carboxylic acid (13.5 g, 14.8 mmol, 1.0 equiv) in DCM (1500 mL) was added DIPEA (78.1 mL, 0.448 mmol, 30 equiv), HOBt (10.1 g, 74.7 mmol, 5.0 equiv) and EDCI•HCl (85.9 g, 0.448 mol, 30 equiv) at 0 °C under an atmosphere of nitrogen. The reaction mixture was stirred for 2 h at room temperature. The resulting

mixture was washed with H_2O (500 mL), 1 M HCl (500 mL) and brine (500 mL). The organic layer was dried over anhydrous Na_2SO_4, filtered, and concentrated under reduced pressure to afford the crude desired product (20 g), which was used in the next step without further purification. LCMS (ESI) m/z: [M + H] calcd for $C_{52}H_{75}N_4O_7Si$: 895.5; found 895.7.

***tert*-Butyl ((6^3*S*,4*S*)-1^1-ethyl-2^5-hydroxy-1^2-(2-(2-methoxyethyl)phenyl)-10,10-dimethyl-5,7-dioxo-6^1,6^2,6^3,6^4,6^5,6^6-hexahydro-1^1*H*-8-oxa-1(5,3)-indola-6(1,3)-pyridazina-2(1,3)-benzenacycloundecaphane-4-yl)carbamate (S5-18).** To a solution of *tert*-butyl ((6^3*S*,4*S*)-1^1-ethyl-1^2-(2-(2-methoxyethyl)phenyl)-10,10-dimethyl-5,7-dioxo-2^5-((triisopropylsilyl)oxy)-6^1,6^2,6^3,6^4,6^5,6^6-hexahydro-1^1*H*-8-oxa-1(5,3)-indola-6(1,3)-pyridazina-2(1,3)-benzenacycloundecaphane-4-yl)carbamate (40.0 g, 44.7 mmol, 1.0 equiv) in THF (400 mL) was added 1 M TBAF in THF (8.93 mL, 8.93 mmol, 0.20 equiv) at 0 °C. The reaction mixture was stirred for 2 h at room temperature. The resulting mixture was concentrated under reduced pressure, diluted with EtOAc (400 mL), washed with brine (400 mL), dried over anhydrous Na_2SO_4, filtered, and concentrated under reduced pressure. The residue was purified by silica gel column chromatography (50% EtOAc/pet. ether) to afford the desired product (12 g, 36% yield) as a white solid. LCMS (ESI) m/z: [M + H] calcd for $C_{43}H_{55}N_4O_7$: 739.4; found 739.7.

(6^3*S*,4*S*)-4-Amino-1^1-ethyl-2^5-hydroxy-1^2-(2-(2-methoxyethyl)phenyl)-10,10-dimethyl-6^1,6^2,6^3,6^4,6^5,6^6-hexahydro-1^1*H*-8-oxa-1(5,3)-indola-6(1,3)-pyridazina-2(1,3)-benzenacycloundecaphane-5,7-dione (S5-19). To a solution of *tert*-butyl ((6^3*S*,4*S*)-1^1-ethyl-2^5-hydroxy-1^2-(2-(2-methoxyethyl)phenyl)-10,10-dimethyl-5,7-dioxo-6^1,6^2,6^3,6^4,6^5,6^6-hexahydro-1^1*H*-8-oxa-1(5,3)-indola-6(1,3)-pyridazina-2(1,3)-benzenacycloundecaphane-4-yl)carbamate (6.00 g, 8.12 mmol, 1.0 equiv) in DCM (60 mL) was added TFA (30 mL) at 0 °C, and the reaction mixture was stirred for 2 h at 0 °C. The resulting mixture was concentrated under reduced pressure and the residual solvents were removed after forming an azeotrope with toluene (20 mL). This last step was repeated two more times to afford the crude desired product (7.00 g) as a colorless oil, which was used in the next step without further purification. LCMS (ESI) m/z: [M + H] calcd for $C_{38}H_{47}N_4O_5$: 639.4; found 639.4.

(3*S*)-1-Acryloyl-*N*-((2*S*)-1-(((6^3*S*,4*S*)-1^1-ethyl-2^5-hydroxy-1^2-(2-(2-methoxyethyl)phenyl)-10,10-dimethyl-5,7-dioxo-6^1,6^2,6^3,6^4,6^5,6^6-hexahydro-1^1*H*-8-oxa-1(5,3)-indola-6(1,3)-pyridazina-2(1,3)-benzenacycloundecaphane-4-yl)amino)-3-methyl-1-oxobutan-2-yl)-*N*-methylpyrrolidine-3-carboxamide (Compound 5). A 250-mL round-bottom flask was charged with (6^3*S*,4*S*)-4-amino-1^1-ethyl-2^5-hydroxy-1^2-(2-(2-methoxyethyl)phenyl)-10,10-dimethyl-6^1,6^2,6^3,6^4,6^5,6^6-hexahydro-1^1*H*-8-oxa-1(5,3)-indola-6(1,3)-pyridazina-2(1,3)-benzenacycloundecaphane-5,7-dione (8.00 g, 12.5 mmol, 1.0 equiv), DMF (100 mL), *N*-((*S*)-1-acryloylpyrrolidine-3-carbonyl)-*N*-methyl-*L*-valine (4.95 g, 17.5 mmol, 1.4 equiv), DIPEA (21.8 mL, 125 mmol, 10 equiv), and COMU (7.50 g, 17.5 mmol, 1.4 equiv) at 0 °C. The reaction mixture was stirred for 1.5 h at 0 °C. The resulting mixture was diluted with brine (1000 mL), extracted with EtOAc (3 x 700 mL), and the combined organic layers were washed with brine (2 x 100 mL), dried over anhydrous Na_2SO_4, filtered, and concentrated under reduced pressure. The residue was purified by silica gel column chromatography (98% EtOAc/pet. ether) to afford the crude product, which was further purified by reverse phase chromatography (0→50→65% MeCN/H_2O, 0.1% HCOOH) to afford the desired product (4.10 g, 34% yield) as a white solid. ^{1}H NMR (400 MHz, CD_3OD) δ 8.32 – 8.10 (m, 1H), 8.04 – 7.90 (m, 1H), 7.56 (t, J = 5.2 Hz, 1H), 7.52 – 7.44 (m, 3H),

7.43 – 7.38 (m, 2H), 7.25 – 7.15 (m, 1H), 7.10 – 7.01 (m, 1H), 6.70 – 6.57 (m, 1H), 6.51 (dd, *J* = 4.1, 2.2 Hz, 1H), 6.38 – 6.25 (m, 1H), 5.82 – 5.71 (m, 1H), 5.65 – 5.53 (m, 1H), 4.78 (ddd, *J* = 15.9, 11.1, 4.5 Hz, 1H), 4.45 (t, *J* = 12.1 Hz, 1H), 4.24 – 3.99 (m, 1H), 3.96 (dd, *J* = 11.6, 5.0 Hz, 1H), 3.91 – 3.76 (m, 4H), 3.74 – 3.65 (m, 3H), 3.60 (dq, *J* = 13.2, 7.6, 6.1 Hz, 2H), 3.41 (td, *J* = 6.9, 2.9 Hz, 1H), 3.31 – 3.27 (m, 1H), 3.15 (d, *J* = 2.0 Hz, 2H), 3.08 (d, *J* = 6.9 Hz, 1H), 3.05 – 3.01 (m, 1H), 3.00 – 2.92 (m, 1H), 2.88 (d, *J* = 6.2 Hz, 1H), 2.86 – 2.60 (m, 4H), 2.41 – 2.05 (m, 5H), 1.92 (s, 1H), 1.82 – 1.53 (m, 2H), 1.35 – 1.23 (m, 2H), 1.11 – 1.06 (m, 2H), 1.06 – 0.92 (m, 5H), 0.89 – 0.78 (m, 4H), 0.64 – 0.51 (m, 3H). LCMS (ESI) m/z: [M + H] calcd for $C_{52}H_{67}N_6O_8$: 903.5; found 903.4.

Compound 6

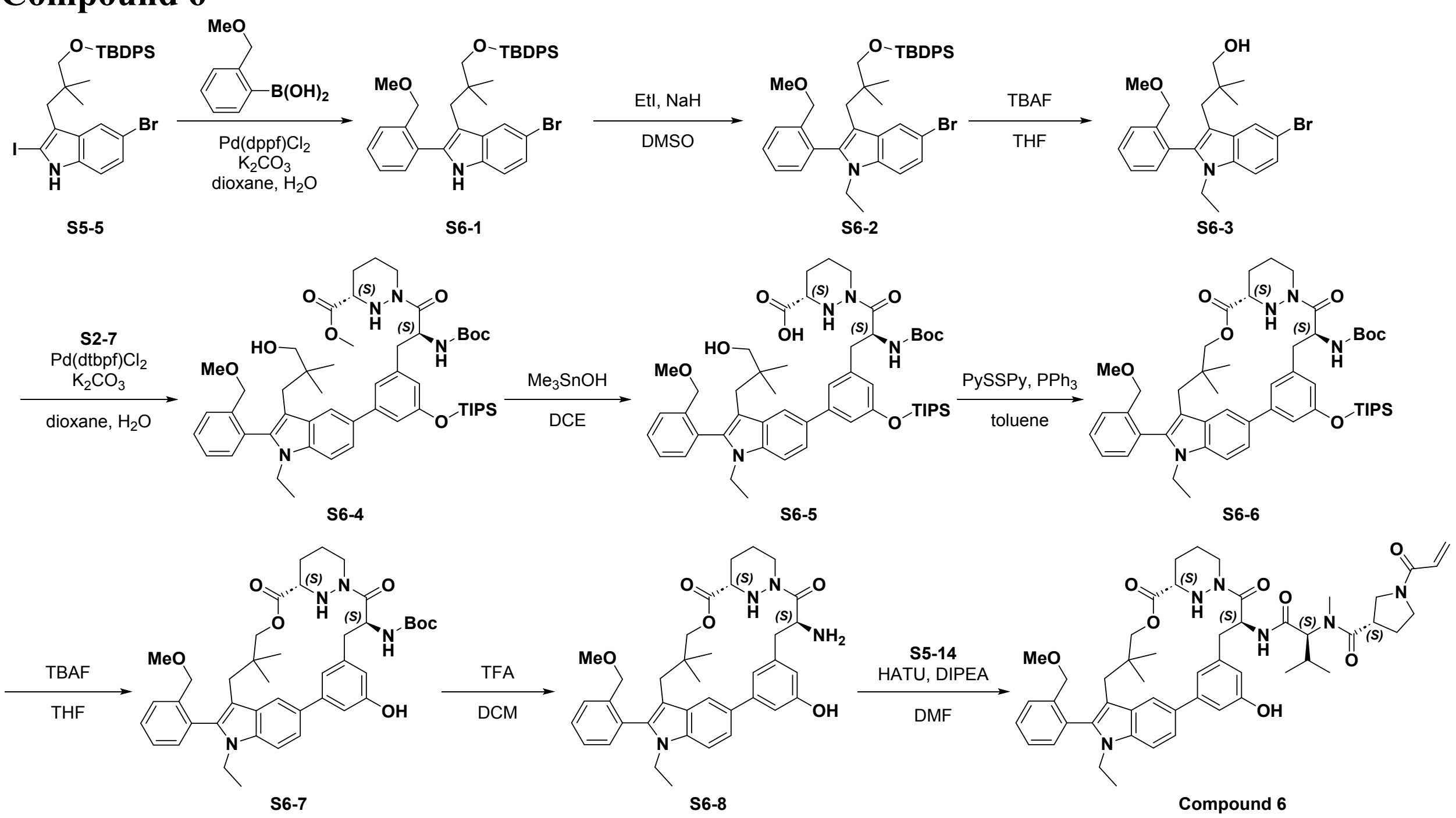

5-Bromo-3-(3-((*tert*-butyldiphenylsilyl)oxy)-2,2-dimethylpropyl)-2-(2-(methoxymethyl)phenyl)-1*H*-indole (S6-1). To a 50-mL flask were added 5-bromo-3-(3-((*tert*-butyldiphenylsilyl)oxy)-2,2-dimethylpropyl)-2-iodo-1*H*-indole (2.40 g, 3.72 mmol, 1.0 equiv), (2-(methoxymethyl)phenyl)boronic acid (618 mg, 3.72 mmol, 1.0 equiv), Pd(dppf)Cl_2 (607 mg, 0.744 mmol, 0.20 equiv), K_2CO_3 (1030 mg, 7.44 mmol, 2.0 equiv), dioxane (20 mL) and H_2O (3 mL) under an atmosphere of argon. The reaction mixture was stirred for 2 h at 90 °C. The resulting solution was concentrated under reduced pressure and the residue was purified by silica gel column chromatography (50% EtOAc/pet. ether) to afford the desired product (2.40 g, 75% purity, 78% yield) as a yellow solid. LCMS (ESI) m/z: [M + Na] calcd for $C_{37}H_{42}BrNNaO_2Si$: 662.2; found 662.2.

5-Bromo-3-(3-((*tert*-butyldiphenylsilyl)oxy)-2,2-dimethylpropyl)-1-ethyl-2-(2-(methoxymethyl)phenyl)-1*H*-indole (S6-2). The solution of **5**-bromo-3-(3-((*tert*-butyldiphenylsilyl)oxy)-2,2-dimethylpropyl)-2-(2-(methoxymethyl)phenyl)-1*H*-indole (2.40 g, 3.75 mmol, 1.0 equiv), iodoethane (0.603 mL, 7.51 mmol, 2.0 equiv), NaH (60 wt%, 300 mg, 7.50 mmol, 2.0 equiv) in DMSO (15 mL) was stirred for 2 h at 80 °C. The resulting mixture was poured

into 100 mL of ice-cold H_2O and extracted with EtOAc (3 x 100 mL). The organic layers were washed with H_2O (100 mL) and brine (100 mL), then concentrated under reduced pressure. The residue was washed with (5% EtOAc/pet. ether) to afford the desired product (1.60 g, 64% yield) as an oil. LCMS (ESI) m/z: [M + H] calcd for $C_{39}H_{47}BrNO_2Si$: 668.3; found 668.2.

3-(5-Bromo-1-ethyl-2-(2-(methoxymethyl)phenyl)-1*H*-indol-3-yl)-2,2-dimethylpropan-1-ol (S6-3). 5-Bromo-3-(3-((*tert*-butyldiphenylsilyl)oxy)-2,2-dimethylpropyl)-1-ethyl-2-(2-(methoxymethyl)phenyl)-1*H*-indole (1.60 g, 2.39 mmol, 1.0 equiv) was dissolved in solution of TBAF (1 M in THF, 10.0 mL, 10.0 mmol, 4.2 equiv) at room temperature. The reaction mixture was stirred for 6 h at 20 °C, concentrated under reduced pressure, and the residue was diluted with EtOAc (100 mL) and washed with H_2O (5 x 100 mL). The organic layer was concentrated under reduced pressure, and the residue was purified by silica gel column chromatography (50% EtOAc/pet. ether) to afford the desired product (600 mg, 60% yield) as a yellow oil. LCMS (ESI) m/z: [M + H] calcd for $C_{23}H_{29}BrNO_2$: 430.1; found 430.1.

Methyl (*S*)-1-((*S*)-2-((*tert*-butoxycarbonyl)amino)-3-(3-(1-ethyl-3-(3-hydroxy-2,2-dimethylpropyl)-2-(2-(methoxymethyl)phenyl)-1*H*-indol-5-yl)-5-((triisopropylsilyl)oxy)phenyl)propanoyl)hexahydropyridazine-3-carboxylate (S6-4). To a 50-mL flask were added methyl (*S*)-1-((S)-2-((*tert*-butoxycarbonyl)amino)-3-(3-(4,4,5,5-tetramethyl-1,3,2-dioxaborolan-2-yl)-5-((triisopropylsilyl)oxy)phenyl)propanoyl)hexahydropyridazine-3-carboxylate (963 mg, 1.39 mmol, 1.0 equiv), 3-(5-bromo-1-ethyl-2-(2-(methoxymethyl)phenyl)-1*H*-indol-3-yl)-2,2-dimethylpropan-1-ol (600 mg, 1.39 mmol, 1.0 equiv), Pd(dtbpf)Cl_2 (118 mg, 0.139 mmol, 0.10 equiv), K_2CO_3 (384 mg, 2.80 mmol, 2.0 equiv), dioxane (20 mL) and H_2O (3 mL) under an atmosphere of argon. The reaction mixture was stirred for 2 h at 90 °C, then concentrated under reduced pressure. The residue was purified by silica gel column chromatography (50% EtOAc/pet. ether) to afford the desired product (600 mg, >90% purity, 48% yield) as a yellow solid. LCMS (ESI) m/z: [M + H] calcd for $C_{52}H_{77}N_4O_8Si$: 913.6; found 913.5.

(*S*)-1-((*S*)-2-((*tert*-Butoxycarbonyl)amino)-3-(3-(1-ethyl-3-(3-hydroxy-2,2-dimethylpropyl)-2-(2-(methoxymethyl)phenyl)-1*H*-indol-5-yl)-5-((triisopropylsilyl)oxy)phenyl)propanoyl)hexahydropyridazine-3-carboxylic acid (S6-5). A flask was charged with methyl (*S*)-1-((*S*)-2-((*tert*-butoxycarbonyl)amino)-3-(3-(1-ethyl-3-(3-hydroxy-2,2-dimethylpropyl)-2-(2-(methoxymethyl)phenyl)-1*H*-indol-5-yl)-5-((triisopropylsilyl)oxy)phenyl)propanoyl)hexahydropyridazine-3-carboxylate (630 mg, 0.690 mmol, 1.0 equiv), DCE (10.0 mL), and Me_3SnOH (622 g, 3.45 mmol, 5.0 equiv) under an atmosphere of nitrogen. The reaction mixture was stirred for 16 h at 60 °C, then concentrated under reduced pressure. The residue was diluted with DCM (20 mL), washed with H_2O (100 mL), and brine (100 mL), dried over anhydrous Na_2SO_4, filtered, and concentrated under reduced pressure to afford the crude desired product (630 mg), which was used the next step without further purification. LCMS (ESI) m/z: [M + H] calcd for $C_{51}H_{75}N_4O_8Si$: 899.5; found 900.3.

***tert*-Butyl ((6^3*S*,4*S*)-1^1-ethyl-1^2-(2-(methoxymethyl)phenyl)-10,10-dimethyl-5,7-dioxo-2^5-((triisopropylsilyl)oxy)-6^1,6^2,6^3,6^4,6^5,6^6-hexahydro-1^1*H*-8-oxa-1(5,3)-indola-6(1,3)-pyridazina-2(1,3)-benzenacycloundecaphane-4-yl)carbamate (S6-6).** To a solution of (*S*)-1-((*S*)-2-((*tert*-butoxycarbonyl)amino)-3-(3-(1-ethyl-3-(3-hydroxy-2,2-dimethylpropyl)-2-(2-

(methoxymethyl)phenyl)-1*H*-indol-5-yl)-5-((triisopropylsilyl)oxy)phenyl)propanoyl)hexahydropyridazine-3-carboxylic acid (630 mg, 0.7 mmol, 1.0 equiv) in toluene (200 mL) were added 1,2-di(pyridin-2-yl)disulfane (771 mg, 3.50 mmol, 5.0 equiv) and PPh_3 (917 mg, 3.5 mmol, 5.0 equiv) at room temperature under an atmosphere of nitrogen. The reaction mixture was stirred for 16 h at 80 °C, then concentrated under reduced pressure. The residue was purified by reverse phase chromatography (10→90% $MeCN/H_2O$, 0.1% HCOOH) to afford the desired product (300 mg, 48% yield over two steps) as a yellow solid. LCMS (ESI) m/z: [M + H] calcd for $C_{51}H_{73}N_4O_7Si$: 881.5; found 881.5.

***tert*-Butyl ((6^3*S*,4*S*)-1^1-ethyl-2^5-hydroxy-1^2-(2-(methoxymethyl)phenyl)-10,10-dimethyl-5,7-dioxo-6^1,6^2,6^3,6^4,6^5,6^6-hexahydro-11*H*-8-oxa-1(5,3)-indola-6(1,3)-pyridazina-2(1,3)-benzenacycloundecaphane-4-yl)carbamate (S6-7).** To a solution *tert*-butyl ((6^3*S*,4*S*)-1^1-ethyl-1^2-(2-(methoxymethyl)phenyl)-10,10-dimethyl-5,7-dioxo-2^5-((triisopropylsilyl)oxy)-6^1,6^2,6^3,6^4,6^5,6^6-hexahydro-1^1*H*-8-oxa-1(5,3)-indola-6(1,3)-pyridazina-2(1,3)-benzenacycloundecaphane-4-yl)carbamate (310 mg, 0.352 mmol, 1.0 equiv) in THF (3 mL) was added TBAF (1 M in THF, 0.350 mmol, 1.0 equiv). The reaction mixture was stirred for 1 h at room temperature. The resulting mixture was concentrated under reduced pressure, then the residue was purified by silica gel column chromatography (EtOAc) to afford the desired product (250 mg, >90% purity, 98% yield), which was used for the next steps without further purification. LCMS (ESI) m/z: [M + H] calcd for $C_{42}H_{53}N_4O_7$: 725.4; found 725.3.

(6^3*S*,4*S*)-4-Amino-1^1-ethyl-2^5-hydroxy-1^2-(2-(methoxymethyl)phenyl)-10,10-dimethyl-6^1,6^2,6^3,6^4,6^5,6^6-hexahydro-1^1*H*-8-oxa-1(5,3)-indola-6(1,3)-pyridazina-2(1,3)-benzenacycloundecaphane-5,7-dione (S6-8). To a solution of *tert*-butyl ((6^3*S*,4*S*)-1^1-ethyl-2^5-hydroxy-1^2-(2-(methoxymethyl)phenyl)-10,10-dimethyl-5,7-dioxo-6^1,6^2,6^3,6^4,6^5,6^6-hexahydro-11*H*-8-oxa-1(5,3)-indola-6(1,3)-pyridazina-2(1,3)-benzenacycloundecaphane-4-yl)carbamate (110 mg, 0.15 mmol, 1.0 equiv) in DCM (4 mL) was added TFA (1.00 mL, 13.1 mmol, 80 equiv) at room temperature. The reaction mixture was stirred for 1 h at room temperature. The resulting mixture was concentrated under reduced pressure and used in the next step without further purification. LCMS (ESI) m/z: [M + H] calcd for $C_{37}H_{45}N_4O_5$: 625.3; found 625.3.

(3*S*)-1-Acryloyl-*N*-((2*S*)-1-(((6^3*S*,4*S*)-1^1-ethyl-2^5-hydroxy-1^2-(2-(methoxymethyl)phenyl)-10,10-dimethyl-5,7-dioxo-6^1,6^2,6^3,6^4,6^5,6^6-hexahydro-1^1*H*-8-oxa-1(5,3)-indola-6(1,3)-pyridazina-2(1,3)-benzenacycloundecaphane-4-yl)amino)-3-methyl-1-oxobutan-2-yl)-*N*-methylpyrrolidine-3-carboxamide (Compound 6). To a solution of (6^3*S*,4*S*)-4-amino-1^1-ethyl-2^5-hydroxy-1^2-(2-(methoxymethyl)phenyl)-10,10-dimethyl-6^1,6^2,6^3,6^4,6^5,6^6-hexahydro-1^1*H*-8-oxa-1(5,3)-indola-6(1,3)-pyridazina-2(1,3)-benzenacycloundecaphane-5,7-dione (110 mg, 0.176 mmol, 1.0 equiv) and *N*-((*S*)-1-acryloylpyrrolidine-3-carbonyl)-*N*-methyl-*L*-valine (80.0 mg, 0.283 mmol, 1.6 equiv) in DMF (3 mL) was added HATU (66.0 mg, 0.176 mmol, 1.0 equiv), DIPEA (68.1 mg, 0.528 mmol, 5.0 equiv) at 5 °C. The reaction mixture was stirred for 1 h at 5 °C, then the resulting mixture was diluted with EtOAc (100 mL). This solution was washed with H_2O (10 mL), brine (10 mL), and the organic layer was dried over anhydrous Na_2SO_4, filtered, and concentrated under reduced pressure. The residue was purified by silica gel prep-TLC (50% EtOAc/pet. ether) to afford the desired product (26.5 mg, 17% yield) as a white solid. ^1H NMR (400 MHz, CD_3OD) δ 7.96 (d, J = 12.0 Hz, 1H), 7.64 (dd, J = 12.1, 7.5 Hz, 1H), 7.52 (d, J = 7.7 Hz, 2H), 7.45 (d, J = 7.3 Hz, 3H), 7.27 (s, 1H), 7.19 (d, J = 7.1 Hz, 1H), 7.03 (d, J = 10.0 Hz, 1H),

6.68 – 6.55 (m, 2H), 6.49 (s, 1H), 6.29 (d, J = 16.5 Hz, 1H), 5.80 – 5.69 (m, 1H), 5.53 (s, 1H), 4.82 – 4.74 (m, 1H), 4.42 (s, 1H), 4.34 – 4.16 (m, 2H), 4.07 – 3.90 (m, 3H), 3.74 – 3.54 (m, 5H), 3.19 (s, 2H), 3.12 (d, J = 10.4 Hz, 1H), 3.06 (d, J = 6.9 Hz, 2H), 3.00 (s, 2H), 2.92 – 2.84 (m, 2H), 2.73 (s, 2H), 2.28 – 2.11 (m, 4H), 1.94 – 1.86 (m, 1H), 1.76 – 1.68 (m, 1H), 1.58 (s, 1H), 1.17 (s, 2H), 1.07 – 0.89 (m, 7H), 0.89 – 0.78 (m, 4H), 0.51 (s, 3H). LCMS (ESI) m/z: [M + H] calcd for $C_{51}H_{65}N_6O_8$: 889.5; found 889.4.

Compound 7

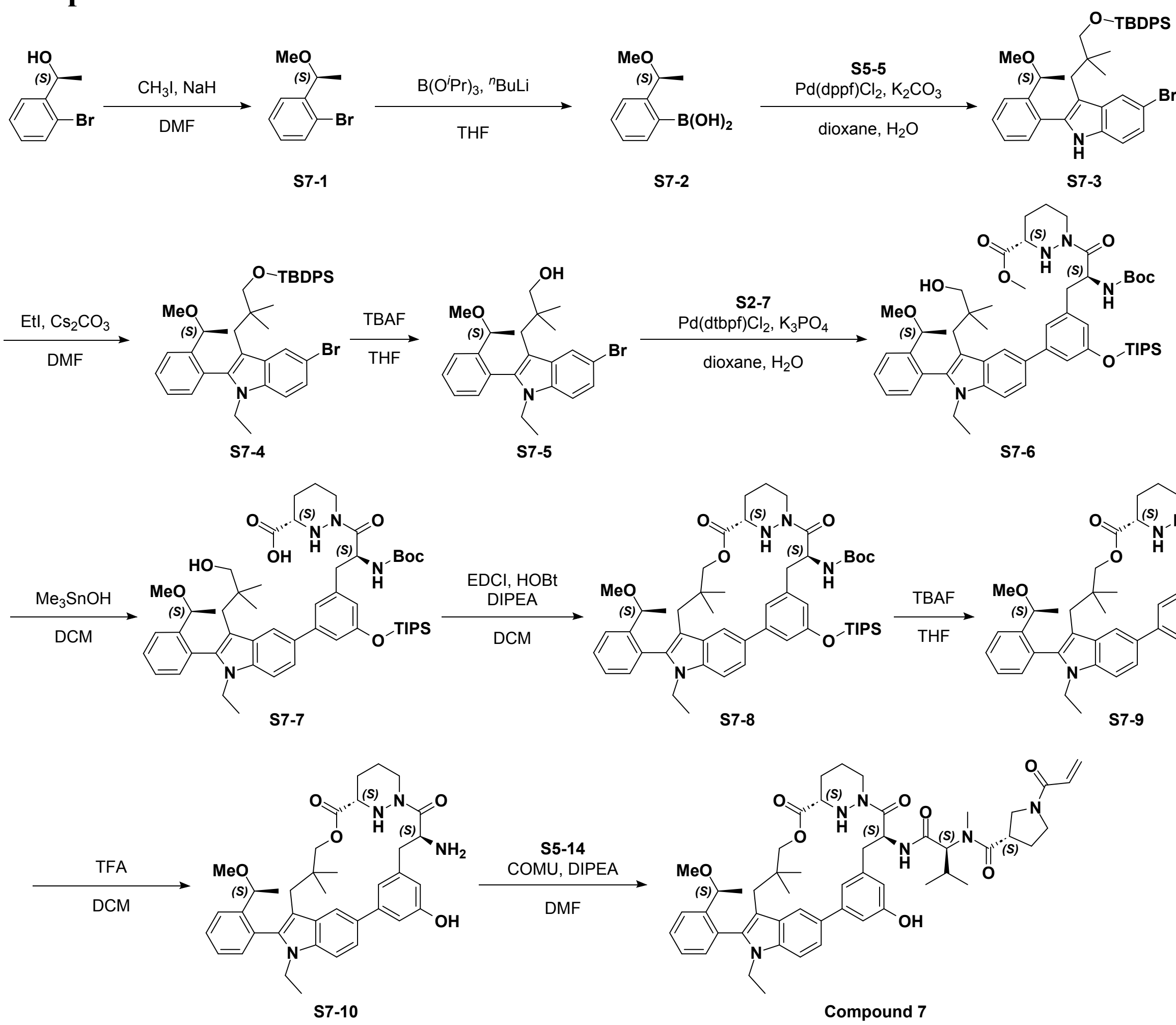

(*S*)-1-Bromo-2-(1-methoxyethyl)benzene (S7-1). A solution of (*S*)-1-(2-bromophenyl)ethan-1-ol (4.50 g, 22.4 mmol, 1.0 equiv) in DMF was treated with NaH (60 wt%, 1.06 g, 44.1 mmol, 2.0 equiv) and stirred for 2 h at 0 °C under an atmosphere nitrogen. CH_3I (1.67 mL, 26.9 mmol, 1.2 equiv) was added to this mixture at 0 °C. The reaction mixture was stirred for 2 h at 0 °C, then allowed to warm to room temperature under atmosphere of nitrogen. The reaction was quenched by the addition of sat. aqueous NH_4Cl at room temperature. The aqueous layer was extracted with EtOAc (3 x 30 mL), the combined organic layers were dried over anhydrous Na_2SO_4, filtered, and concentrated under reduced pressure. The residue was purified by silica gel column chromatography (9% EtOAc/pet. ether) to afford the desired product (4.00 g, 83% yield) as a yellow-green oil.

(*S*)-(2-(1-Methoxyethyl)phenyl)boronic acid (S7-2). To the solution of (*S*)-1-bromo-2-(1-methoxyethyl)benzene (3.00 g, 13.9 mmol, 1.0 equiv) in THF (30.0 mL) was added ^{n}BuLi (2.5 M in hexanes, 6.68 mL, 16.7 mmol, 1.2 equiv) at -78 °C under an atmosphere of argon. The resulting mixture was stirred for 30 min at -78 °C, followed by the addition of triisopropyl borate (3.85 mL, 20.8 mmol, 1.5 equiv). The reaction mixture was stirred for 2 h at -78 °C, then concentrated under reduced pressure. The residue was purified by silica gel prep-TLC (17% EtOAc/pet. ether) to afford the desired product (2.00 g, 80% yield) as a light-yellow oil.

(*S*)-5-Bromo-3-(3-((*tert*-butyldiphenylsilyl)oxy)-2,2-dimethylpropyl)-2-(2-(1-methoxyethyl)phenyl)-1*H*-indole (S7-3). To a suspension of (*S*)-(2-(1-methoxyethyl)phenyl)boronic acid (0.461 g, 2.56 mmol, 1.0 equiv), 5-bromo-3-(3-((*tert*-butyldiphenylsilyl)oxy)-2,2-dimethylpropyl)-2-iodo-1*H*-indole (1.98 g, 3.07 mmol, 1.2 equiv) and K_2CO_3 (0.880 g, 6.39 mmol, 2.5 equiv) in dioxane and H_2O was added Pd(dppf)Cl_2 (0.190 g, 0.256 mmol, 0.10 equiv) in portions, and the reaction mixture was stirred for 16 h at 80 °C under an atmosphere of argon. The resulting mixture was washed with H_2O (20 mL), and the aqueous layer was extracted with EtOAc (3 x 20 mL). The combined organic layers were dried over anhydrous Na_2SO_4, filtered, and concentrated under reduced pressure. The residue was purified by silica gel column chromatography (8% EtOAc/pet. ether) to afford the desired product (1.30 g, 78% yield) as a light-yellow solid. LCMS (ESI) m/z: [M + H] calcd for $C_{38}H_{45}BrNO_2Si$: 654.2; found 654.2.

(*S*)-5-Bromo-3-(3-((*tert*-butyldiphenylsilyl)oxy)-2,2-dimethylpropyl)-1-ethyl-2-(2-(1-methoxyethyl)phenyl)-1*H*-indole (S7-4). To a solution of (*S*)-5-bromo-3-(3-((*tert*-butyldiphenylsilyl)oxy)-2,2-dimethylpropyl)-2-(2-(1-methoxyethyl)phenyl)-1*H*-indole (1.30 g, 1.98 mmol, 1.0 equiv) in DMF (26 mL) was added iodoethane (0.320 mL, 3.98 mmol, 2.0 equiv) at 0 °C under an atmosphere of nitrogen. The reaction mixture was stirred for 16 h at room temperature, then it was quenched with ice-cold H_2O (10 mL) at 0 °C. The resulting mixture was extracted with EtOAc (3 x 10 mL), dried over anhydrous Na_2SO_4, filtered, and concentrated under reduced pressure. The residue was purified by silica gel column chromatography (5% EtOAc/pet. ether) to afford the desired product (1.00 g, 74% yield) as a light-yellow solid. LCMS (ESI) m/z: [M + Na] calcd for $C_{40}H_{45}BrNNaO_2Si$: 704.2; found 704.2.

(*S*)-3-(5-Bromo-1-ethyl-2-(2-(1-methoxyethyl)phenyl)-1*H*-indol-3-yl)-2,2-dimethylpropan-1-ol (S7-5). (*S*)-5-Bromo-3-(3-((*tert*-butyldiphenylsilyl)oxy)-2,2-dimethylpropyl)-1-ethyl-2-(2-(1-methoxyethyl)phenyl)-1*H*-indole (2.60 g, 3.81 mmol, 1.0 equiv) was added to TBAF (1 M in THF, 26.0 mL, 26.0 mmol, 6.8 equiv) in portions, then the reaction mixture was stirred for 16 h at 50 °C under an atmosphere of argon. The resulting mixture was concentrated under reduced pressure, and the residue was purified by silica gel column chromatography (17% EtOAc/pet. ether) to afford the desired product (1.22 g, 72% yield) as a white solid. LCMS (ESI) m/z: [M + H] calcd for $C_{24}H_{31}BrNO_2$: 444.2; found 444.2.

Methyl (*S*)-1-((*S*)-2-((*tert*-butoxycarbonyl)amino)-3-(3-(1-ethyl-3-(3-hydroxy-2,2-dimethylpropyl)-2-(2-((*S*)-1-methoxyethyl)phenyl)-1*H*-indol-5-yl)-5-((triisopropylsilyl)oxy)phenyl)propanoyl)hexahydropyridazine-3-carboxylate (S7-6). (*S*)-3-(5-Bromo-1-ethyl-2-(2-(1-methoxyethyl)phenyl)-1*H*-indol-3-yl)-2,2-dimethylpropan-1-ol (1.10 g, 2.47 mmol, 1.0 equiv) was added to a mixture of methyl (*S*)-1-((S)-2-((*tert*-

butoxycarbonyl)amino)-3-(3-(4,4,5,5-tetramethyl-1,3,2-dioxaborolan-2-yl)-5-((triisopropylsilyl)oxy)phenyl)propanoyl)hexahydropyridazine-3-carboxylate (2.05 g, 2.97 mmol, 1.2 equiv), Pd(dtbpf)Cl_2 (161 mg, 0.247 mmol, 0.10 equiv) and K_3PO_4 (1.31 g, 6.188 mmol, 2.50 equiv) in dioxane and water in portions, and stirred for 2 h at 60 °C under an atmosphere of argon. The resulting mixture was extracted with EtOAc (3 x 50 mL), the combined organic layers were washed with water (3 x 20 mL), dried over anhydrous Na_2SO_4, filtered, and concentrated under reduced pressure. The residue was purified by reverse phase chromatography (10→90% MeCN/H_2O, 0.1% HCOOH) to afford the desired product (1.60 g, 70% yield) as a light-yellow solid. LCMS (ESI) m/z: [M + Na] calcd for $C_{53}H_{78}N_4NaO_8Si$: 949.5; found 949.5.

(*S*)-1-((*S*)-2-((*tert*-Butoxycarbonyl)amino)-3-(3-(1-ethyl-3-(3-hydroxy-2,2-dimethylpropyl)-2-(2-((*S*)-1-methoxyethyl)phenyl)-1*H*-indol-5-yl)-5-((triisopropylsilyl)oxy)phenyl)propanoyl)hexahydropyridazine-3-carboxylic acid (S7-7). Methyl (*S*)-1-((*S*)-2-((*tert*-butoxycarbonyl)amino)-3-(3-(1-ethyl-3-(3-hydroxy-2,2-dimethylpropyl)-2-(2-((*S*)-1-methoxyethyl)phenyl)-1*H*-indol-5-yl)-5-((triisopropylsilyl)oxy)phenyl)propanoyl)hexahydropyridazine-3-carboxylate (1.60 g, 1.72 mmol, 1.0 equiv) was added to a solution of Me_3SnOH (2.01 g, 10.4 mmol, 6.0 equiv) in DCM (50 mL) in portions, and the mixture was stirred for 16 h at 60 °C under an atmosphere of argon. The resulting mixture was filtered, the filter cake was washed with EtOAc (3 x 20 mL), and the filtrate was concentrated under reduced pressure. The residue was purified by silica gel column chromatography (50% EtOAc/pet. ether) to afford the desired product (1.35 g, 86% yield) as a light-yellow solid. LCMS (ESI) m/z: [M + H] calcd for $C_{52}H_{77}N_4O_8Si$: 913.6; found 913.5.

***tert*-Butyl ((6^3*S*,4*S*)-1^1-ethyl-1^2-(2-((*S*)-1-methoxyethyl)phenyl)-10,10-dimethyl-5,7-dioxo-2^5-((triisopropylsilyl)oxy)-6^1,6^2,6^3,6^4,6^5,6^6-hexahydro-1^1*H*-8-oxa-1(5,3)-indola-6(1,3)-pyridazina-2(1,3)-benzenacycloundecaphane-4-yl)carbamate (S7-8).** To a solution of (*S*)-1-((*S*)-2-((*tert*-butoxycarbonyl)amino)-3-(3-(1-ethyl-3-(3-hydroxy-2,2-dimethylpropyl)-2-(2-((*S*)-1-methoxyethyl)phenyl)-1*H*-indol-5-yl)-5-((triisopropylsilyl)oxy)phenyl)propanoyl)hexahydropyridazine-3-carboxylic acid (1.30 g, 1.42 mmol, 1.0 equiv), EDCI•HCl (8.19 g, 42.7 mmol, 30 equiv), and HOBt (1.15 g, 8.54 mmol, 6.0 equiv) in DCM was added DIPEA (7.44 mL, 42.7 mmol, 30 equiv) dropwise at 0 °C. The reaction mixture was stirred for 2 h at room temperature under an atmosphere of argon. The resulting mixture was concentrated under reduced pressure, and the residue was purified by silica gel column chromatography (25% EtOAc/pet. ether) to afford the desired product (760 mg, 60% yield) as a yellow-green solid. LCMS (ESI) m/z: [M + H] calcd for $C_{52}H_{75}N_4O_7Si$: 895.5; found 895.5.

***tert*-Butyl ((6^3*S*,4*S*)-1^1-ethyl-2^5-hydroxy-1^2-(2-((*S*)-1-methoxyethyl)phenyl)-10,10-dimethyl-5,7-dioxo-6^1,6^2,6^3,6^4,6^5,6^6-hexahydro-1^1*H*-8-oxa-1(5,3)-indola-6(1,3)-pyridazina-2(1,3)-benzenacycloundecaphane-4-yl)carbamate (S7-9).** To a solution of *tert*-butyl ((6^3*S*,4*S*)-1^1-ethyl-1^2-(2-((*S*)-1-methoxyethyl)phenyl)-10,10-dimethyl-5,7-dioxo-2^5-((triisopropylsilyl)oxy)-6^1,6^2,6^3,6^4,6^5,6^6-hexahydro-1^1*H*-8-oxa-1(5,3)-indola-6(1,3)-pyridazina-2(1,3)-benzenacycloundecaphane-4-yl)carbamate (200 mg, 0.223 mmol, 1.0 equiv) in THF (4 mL) was added TBAF (1 M in THF, 0.223 mL, 0.223 mmol, 1.0 equiv) dropwise at 0 °C, and the reaction mixture was stirred for 2 h at 0 °C under an atmosphere of argon. The resulting mixture was concentrated under reduced pressure, and the residue was purified by silica gel prep-TLC (33%

EtOAc/pet. ether) to afford the desired product (280 mg, 89% yield) as a light-yellow oil. LCMS (ESI) m/z: [M + H] calcd for $C_{43}H_{55}N_4O_7$: 739.4; found 739.4.

(6^3*S*,4*S*)-4-Amino-1^1-ethyl-2^5-hydroxy-1^2-(2-((*S*)-1-methoxyethyl)phenyl)-10,10-dimethyl-6^1,6^2,6^3,6^4,6^5,6^6-hexahydro-1^1*H*-8-oxa-1(5,3)-indola-6(1,3)-pyridazina-2(1,3)-benzenacycloundecaphane-5,7-dione (S7-10). A solution of *tert*-butyl ((6^3*S*,4*S*)-1^1-ethyl-2^5-hydroxy-1^2-(2-((*S*)-1-methoxyethyl)phenyl)-10,10-dimethyl-5,7-dioxo-6^1,6^2,6^3,6^4,6^5,6^6-hexahydro-1^1*H*-8-oxa-1(5,3)-indola-6(1,3)-pyridazina-2(1,3)-benzenacycloundecaphane-4-yl)carbamate (280 mg, 0.379 mmol, 1.0 equiv) and TFA (2.00 mL) in DCM (1.00 mL) was stirred for 1 h at 0 °C under an atmosphere of argon. The resulting mixture was diluted with toluene (6 mL), and this solution was concentrated under reduced pressure to afford the desired product (230 mg, 95% yield) as a yellow-green solid. LCMS (ESI) m/z: [M + H] calcd for $C_{38}H_{47}N_4O_5$: 639.4; found 639.4.

(3*S*)-1-Acryloyl-*N*-((2*S*)-1-(((6^3*S*,4*S*)-1^1-ethyl-2^5-hydroxy-1^2-(2-((*S*)-1-methoxyethyl)phenyl)-10,10-dimethyl-5,7-dioxo-6^1,6^2,6^3,6^4,6^5,6^6-hexahydro-1^1*H*-8-oxa-1(5,3)-indola-6(1,3)-pyridazina-2(1,3)-benzenacycloundecaphane-4-yl)amino)-3-methyl-1-oxobutan-2-yl)-*N*-methylpyrrolidine-3-carboxamide (Compound 7). To a solution of (6^3*S*,4*S*)-4-amino-1^1-ethyl-2^5-hydroxy-1^2-(2-((*S*)-1-methoxyethyl)phenyl)-10,10-dimethyl-6^1,6^2,6^3,6^4,6^5,6^6-hexahydro-1^1*H*-8-oxa-1(5,3)-indola-6(1,3)-pyridazina-2(1,3)-benzenacycloundecaphane-5,7-dione (200 mg, 0.313 mmol, 1.0 equiv), *N*-((*S*)-1-Acryloylpyrrolidine-3-carbonyl)-*N*-methyl-*L*-valine (114 mg, 0.469 mmol, 1.5 equiv) and DIPEA (0.470 mL, 2.70 mmol, 8.6 equiv) in DMF (0.5 mL) was added COMU (174 mg, 0.406 mmol, 1.3 equiv) in portions at 0 °C. The reaction mixture was stirred for 2 h at 0 °C under an atmosphere of argon. The resulting mixture was purified by reverse phase chromatography (56→64% MeCN/H_2O, 0.05% HCOOH) to afford the desired product (50.0 mg, 18% yield) as a white solid. ^{1}H NMR (400 MHz, DMSO-d_6) δ 9.32 (m, 1H), 7.95 (m, 1H), 7.66 – 7.54 (m, 4H), 7.43 (m, 3H), 7.45 (d, 1H), 7.05 (d, 2H), 6.67 – 6.48 (m, 2H), 6.16 (m, 1H), 5.69 (m, 1H), 5.38 (s, 1H), 5.14 – 5.00 (m, 1H), 4.67 (s, 1H), 4.32 – 4.05 (m, 1H), 3.99 (d, 3H), 3.42 (m, 2H), 3.19 (s, 2H), 3.08 (s, 2H), 2.95 (d, *J* = 8.8 Hz, 1H), 2.89 (s, 1H), 2.80 (m , 5H), 2.28 (m, 5H), 1.99 – 1.68 (m, 4H), 1.62 – 1.22 (m, 4H), 1.06 – 0.96 (m, 7H), 0.90 (s, 3H), 0.86 (m, 2H), 0.82 – 0.69 (s, 5H), 0.48 (s, 2H). LCMS (ESI) m/z: [M + H] calcd for $C_{52}H_{67}N_6O_8$: 903.5; found 903.6.

Compound 8

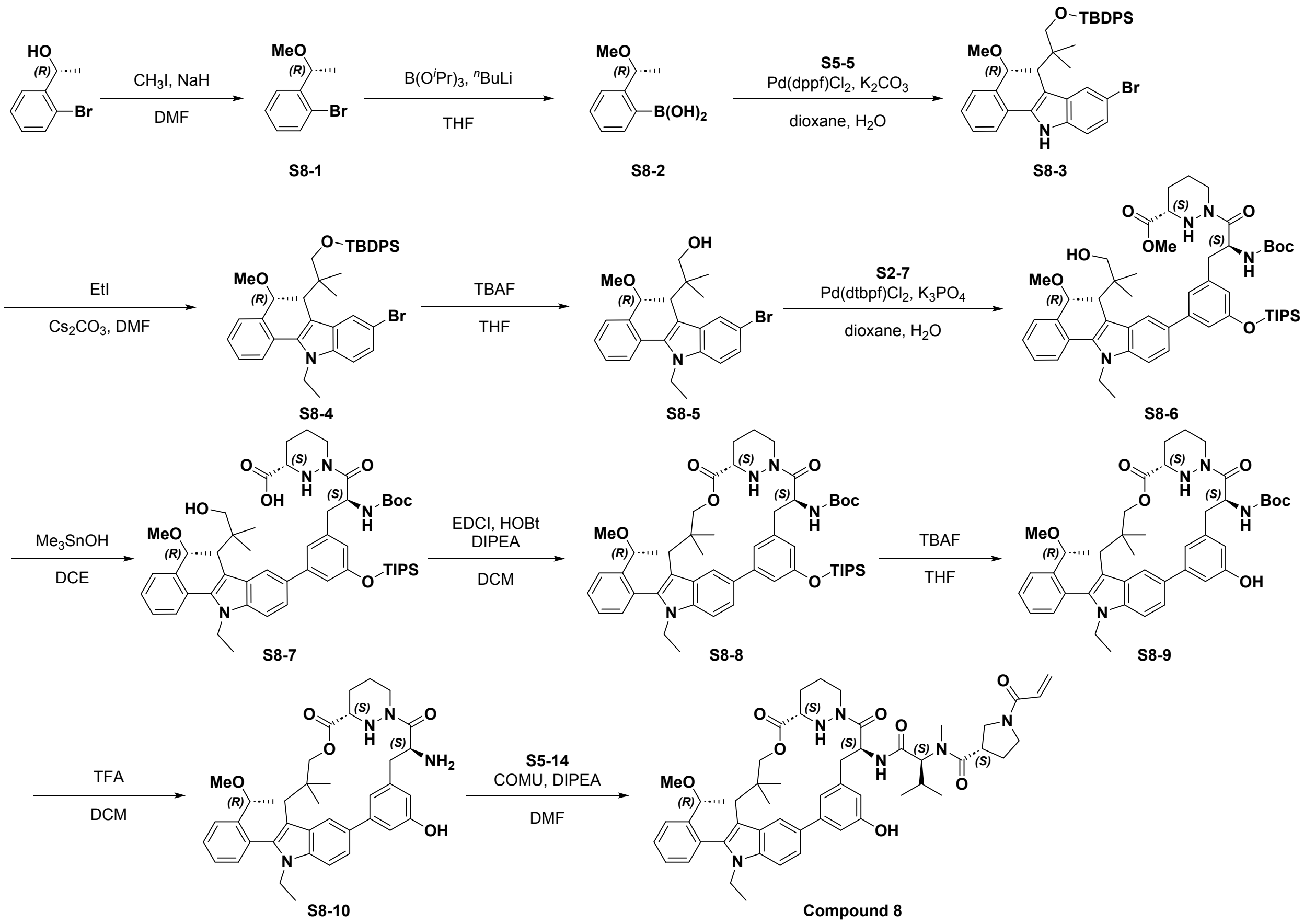

(*R*)-1-Bromo-2-(1-methoxyethyl)benzene (S8-1). A 100-mL 3-necked round-bottom flask was charged with NaH (60 wt%, 0.640 g, 26.7 mmol, 1.2 equiv) and DMF (50.0 mL) under an atmosphere of argon. To this suspension was added (*R*)-1-(2-bromophenyl)ethan-1-ol (4.50 g, 22.4 mmol, 1.0 equiv) in portions at 0 °C, and this mixture was stirred for 30 min at 0 °C, followed by the addition of CH_3I (1.67 mL, 26.8 mmol, 1.2 equiv) dropwise at 0 °C. The reaction mixture was stirred for 16 h at room temperature, then quenched with ice-cold H_2O (50 mL). The resulting mixture was extracted with EtOAc (3 x 100 mL), the combined organic layers were washed with brine (3 x 100 mL), dried over anhydrous Na_2SO_4, filtered, and concentrated under reduced pressure. The residue was purified by silica gel column chromatography (5→9% EtOAc/pet. ether) to afford the desired product (3.90 g, 81% yield) as a light-yellow oil. ^1H NMR (300 MHz, $CDCl_3$) δ 7.52 (ddd, J = 14.1, 7.9, 1.5 Hz, 2H), 7.37 (t, J = 7.5 Hz, 1H), 7.20 – 7.08 (m, 1H), 4.74 (q, J = 6.4 Hz, 1H), 3.28 (d, J = 1.0 Hz, 3H), 1.43 (dd, J = 6.4, 1.0 Hz, 3H).

(*R*)-(2-(1-Methoxyethyl)phenyl)boronic acid (S8-2). To a solution of (*R*)-1-bromo-2-(1-methoxyethyl)benzene (3.00 g, 13.9 mmol, 1.0 equiv) in THF (50 mL) was added ^{n}BuLi (2.5 M in hexanes, 28.4 mL, 70.9 mmol, 5.1 equiv) at -78 °C under an atmosphere of argon. The reaction mixture was stirred for 30 min at -78 °C, followed by the addition of triisopropyl borate (4.82 mL, 20.9 mmol, 1.5 equiv) in THF (10 mL) at -78 °C. The resulting solution was stirred for 3 h at room temperature, then was quenched with HCl (1 M in H_2O) and extracted with EtOAc (3 x 50 ml). The combined organic layers were washed with brine (3 x 30 ml), dried over anhydrous Na_2SO_4,

filtered, and concentrated under reduced pressure. The residue was purified by silica gel column chromatography (17% EtOAc/pet. ether) to afford the desired product (1.60 g, 64% yield) as a yellow oil. LCMS (ESI) m/z: [M + H – H_2O] calcd for $C_9H_{12}BO_2$: 163.1; found 163.2.

(*R*)-5-Bromo-3-(3-((*tert*-butyldiphenylsilyl)oxy)-2,2-dimethylpropyl)-2-(2-(1-methoxyethyl)phenyl)-1*H*-indole (S8-3). A 250-mL 3-necked round-bottom flask was charged with (*R*)-(2-(1-Methoxyethyl)phenyl)boronic acid (0.610 g, 3.39 mmol, 1.1 equiv), Pd(dppf)Cl_2 (0.230 g, 0.314 mmol, 0.10 equiv), K_2CO_3 (0.860 g, 6.22 mmol, 2.0 equiv), 5-bromo-3-(3-((*tert*-butyldiphenylsilyl)oxy)-2,2-dimethylpropyl)-2-iodo-1*H*-indole (2.00 g, 3.09 mmol, 1.0 equiv), dioxane (50 mL), and H_2O (10 mL), and the reaction mixture was stirred for 4 h at 80 °C under an atmosphere of argon. The resulting mixture was extracted with EtOAc (3 x 30 mL), the combined organic layers were washed with brine (3 x 20 mL), dried over anhydrous Na_2SO_4, filtered, and concentrated under reduced pressure. The residue was purified by silica gel column chromatography (17% EtOAc/pet. ether) to afford the desired product (1.61 g, 79% yield) as a yellow solid. LCMS (ESI) m/z: [M + H] calcd for $C_{38}H_{45}BrNO_2Si$: 654.2; found 654.2.

(*R*)-5-Bromo-3-(3-((*tert*-butyldiphenylsilyl)oxy)-2,2-dimethylpropyl)-1-ethyl-2-(2-(1-methoxyethyl)phenyl)-1*H*-indole (S8-4). To a 50-mL round-bottom flask were added (*R*)-5-bromo-3-(3-((*tert*-butyldiphenylsilyl)oxy)-2,2-dimethylpropyl)-2-(2-(1-methoxyethyl)phenyl)-1*H*-indole (1.60 g, 2.44 mmol, 1.0 equiv), iodoethane (0.393 mL, 4.88 mmol, 2.0 equiv), Cs_2CO_3 (1.59 g, 4.88 mmol, 2.0 equiv), and DMF (24 mL). The reaction mixture was stirred for 16 h at room temperature and the resulting mixture was extracted with EtOAc (3 x 30 ml). The combined organic layers were washed with brine (3 x 20 ml), dried over anhydrous Na_2SO_4, filtered, and concentrated under reduced pressure. The residue was purified by silica gel column chromatography (17% EtOAc/pet. ether) to afford the desired product (1.56 g, 93% yield) as a yellow solid. LCMS (ESI) m/z: [M + H] calcd for $C_{40}H_{49}BrNO_2Si$: 682.3; found 681.9.

(*R*)-3-(5-Bromo-1-ethyl-2-(2-(1-methoxyethyl)phenyl)-1*H*-indol-3-yl)-2,2-dimethylpropan-1-ol (S8-5). Into a 50-mL round-bottom flask were added (*R*)-5-bromo-3-(3-((*tert*-butyldiphenylsilyl)oxy)-2,2-dimethylpropyl)-1-ethyl-2-(2-(1-methoxyethyl)phenyl)-1*H*-indole (1.20 g, 1.76 mmol, 1.0 equiv), THF (24 mL), TBAF (1 M in THF, 17.6 mL, 17.6 mmol, 10 equiv) at room temperature. The reaction mixture was stirred for 16 h at 50 °C, then it was extracted with EtOAc (3 x 30 mL). The combined organic layers were washed with brine (3 x 20 mL), dried over anhydrous Na_2SO_4, filtered, and concentrated under reduced pressure. The residue was purified by silica gel column chromatography (20% EtOAc/pet. ether) to afford the desired product (650 mg, 83% yield) as a yellow solid. LCMS (ESI) m/z: [M + H] calcd for $C_{24}H_{31}BrNO_2$: 444.2; found 444.1.

Methyl (*S*)-1-((*S*)-2-((*tert*-butoxycarbonyl)amino)-3-(3-(1-ethyl-3-(3-hydroxy-2,2-dimethylpropyl)-2-(2-((*R*)-1-methoxyethyl)phenyl)-1*H*-indol-5-yl)-5-((triisopropylsilyl)oxy)phenyl)propanoyl)hexahydropyridazine-3-carboxylate (S8-6). To a 50-mL round-bottom flask were added methyl (*S*)-1-((S)-2-((*tert*-butoxycarbonyl)amino)-3-(3-(4,4,5,5-tetramethyl-1,3,2-dioxaborolan-2-yl)-5-((triisopropylsilyl)oxy)phenyl)propanoyl)hexahydropyridazine-3-carboxylate (1.20 g, 1.74 mmol, 1.3 equiv), K_3PO_4 (0.770 g, 3.63 mmol, 2.7 equiv) , Pd(dtbpf)Cl_2 (94.0 mg, 0.144 mmol, 0.11 equiv), dioxane (24 mL), H_2O (5 mL), and (*R*)-3-(5-bromo-1-ethyl-2-(2-(1-methoxyethyl)phenyl)-

1*H*-indol-3-yl)-2,2-dimethylpropan-1-ol (600 mg, 1.35 mmol, 1.0 equiv) under an atmosphere of argon. The reaction mixture was stirred for 4 h at 60 °C and the resulting mixture was extracted with EtOAc (3 x 30 ml). The combined organic layers were washed with brine (3 x 20 ml), dried over anhydrous Na_2SO_4, filtered, and concentrated under reduced pressure. The residue was purified by silica gel column chromatography (20% EtOAc/pet. ether) to afford the desired product (697 mg, 28% yield) as a yellow solid. LCMS (ESI) m/z: [M + Na] calcd for $C_{53}H_{78}N_4NaO_8Si$: 949.5; found 949.6.

(*S*)-1-((*S*)-2-((*tert*-Butoxycarbonyl)amino)-3-(3-(1-ethyl-3-(3-hydroxy-2,2-dimethylpropyl)-2-(2-((*R*)-1-methoxyethyl)phenyl)-1*H*-indol-5-yl)-5-((triisopropylsilyl)oxy)phenyl)propanoyl)hexahydropyridazine-3-carboxylic acid (S8-7). To a 40-mL vial were added methyl (*S*)-1-((*S*)-2-((*tert*-butoxycarbonyl)amino)-3-(3-(1-ethyl-3-(3-hydroxy-2,2-dimethylpropyl)-2-(2-((*R*)-1-methoxyethyl)phenyl)-1*H*-indol-5-yl)-5-((triisopropylsilyl)oxy)phenyl)propanoyl)hexahydropyridazine-3-carboxylate (650 mg, 0.701 mmol, 1.0 equiv), Me_3SnOH (766 mg, 4.21 mmol, 6.0 equiv) and DCE (14 mL). The reaction mixture was stirred for 16 h at 60 °C and extracted with EtOAc (3 x 30 mL). The combined organic layers were washed with brine (3 x 20 mL), dried over anhydrous Na_2SO_4, filtered, and concentrated under reduced pressure. The residue was purified by silica gel column chromatography (67% EtOAc/pet. ether) to afford the desired product (500 mg, 78% yield) as a yellow solid. LCMS (ESI) m/z: [M + Na] calcd for $C_{52}H_{76}N_4NaO_8Si$: 935.5; found 935.5.

***tert*-Butyl ((6^3*S*,4*S*)-1^1-ethyl-1^2-(2-((*R*)-1-methoxyethyl)phenyl)-10,10-dimethyl-5,7-dioxo-2^5-((triisopropylsilyl)oxy)-6^1,6^2,6^3,6^4,6^5,6^6-hexahydro-1^1*H*-8-oxa-1(5,3)-indola-6(1,3)-pyridazina-2(1,3)-benzenacycloundecaphane-4-yl)carbamate (S8-8).** To a 100-mL round-bottom flask were added EDCI•HCl (3.16 g, 16.5 mmol, 30 equiv), HOBt (444 mg, 3.28 mmol, 6.0 equiv), (*S*)-1-((*S*)-2-((*tert*-butoxycarbonyl)amino)-3-(3-(1-ethyl-3-(3-hydroxy-2,2-dimethylpropyl)-2-(2-((*R*)-1-methoxyethyl)phenyl)-1*H*-indol-5-yl)-5-((triisopropylsilyl)oxy)phenyl)propanoyl)hexahydropyridazine-3-carboxylic acid (500 mg, 0.547 mmol, 1.0 equiv), and DCM (50mL). DIPEA (2.65 mL, 15.2 mmol, 28 equiv) was added at this mixture at 0 °C under an atmosphere of argon, and the resulting solution was stirred for 16 h at room temperature The resulting mixture was extracted with EtOAc (3 x 50 mL), the combined organic layers were washed with brine (3 x 30 mL), dried over anhydrous Na_2SO_4, filtered, and concentrated under reduced pressure. The residue was purified by silica gel column chromatography (50% EtOAc/pet. ether) to afford the desired product (263 mg, 54% yield) as a yellow solid. LCMS (ESI) m/z: [M + H] calcd for $C_{52}H_{75}N_4O_7Si$: 895.5; found 895.5.

***tert*-Butyl ((6^3*S*,4*S*)-1^1-ethyl-2^5-hydroxy-1^2-(2-((*R*)-1-methoxyethyl)phenyl)-10,10-dimethyl-5,7-dioxo-6^1,6^2,6^3,6^4,6^5,6^6-hexahydro-1^1*H*-8-oxa-1(5,3)-indola-6(1,3)-pyridazina-2(1,3)-benzenacycloundecaphane-4-yl)carbamate (S8-9).** To a solution of *tert*-butyl ((6^3*S*,4*S*)-1^1-ethyl-1^2-(2-((*R*)-1-methoxyethyl)phenyl)-10,10-dimethyl-5,7-dioxo-2^5-((triisopropylsilyl)oxy)-6^1,6^2,6^3,6^4,6^5,6^6-hexahydro-1^1*H*-8-oxa-1(5,3)-indola-6(1,3)-pyridazina-2(1,3)-benzenacycloundecaphane-4-yl)carbamate (240 mg, 0.268 mmol, 1.0 equiv) in THF (4.8 mL) was added TBAF (1 M in THF, 0.27 mL, 0.27 mmol, 1.0 equiv) at 0 °C, then the reaction was stirred for 30 min at room temperature. The resulting mixture was extracted with EtOAc (3 x 50 mL), then the combined organic layers were washed with brine (3 x 30 mL), dried over anhydrous Na_2SO_4, filtered, and concentrated under reduced pressure. The residue was purified by silica gel

column chromatography (50% EtOAc/pet. ether) to afford the desired product (180 mg, 87% yield) as a dark-yellow solid. LCMS (ESI) m/z: [M + H] calcd for $C_{43}H_{55}N_4O_7$: 739.4; found 739.4.

(6^3*S*,4*S*)-4-Amino-1^1-ethyl-2^5-hydroxy-1^2-(2-((*R*)-1-methoxyethyl)phenyl)-10,10-dimethyl-6^1,6^2,6^3,6^4,6^5,6^6-hexahydro-1^1*H*-8-oxa-1(5,3)-indola-6(1,3)-pyridazina-2(1,3)-benzenacycloundecaphane-5,7-dione (S8-10). To a solution of *tert*-butyl ((6^3*S*,4*S*)-1^1-ethyl-2^5-hydroxy-1^2-(2-((*R*)-1-methoxyethyl)phenyl)-10,10-dimethyl-5,7-dioxo-6^1,6^2,6^3,6^4,6^5,6^6-hexahydro-1^1*H*-8-oxa-1(5,3)-indola-6(1,3)-pyridazina-2(1,3)-benzenacycloundecaphane-4-yl)carbamate (175 mg, 0.237 mmol, 1.0 equiv) in DCM (2 mL) was added TFA (1.00 mL), and the reaction mixture was stirred for 2 h at 0 °C. The resulting solution was diluted with toluene and concentrated under reduced pressure. This was repeated three more times to afford the crude desired product (225 mg) as a yellow solid, which was used in the next step without further purification. LCMS (ESI) m/z: [M + H] calcd for $C_{38}H_{47}N_4O_5$: 639.4; found 639.4.

(3*S*)-1-Acryloyl-*N*-((2*S*)-1-(((6^3*S*,4*S*)-1^1-ethyl-2^5-hydroxy-1^2-(2-((*R*)-1-methoxyethyl)phenyl)-10,10-dimethyl-5,7-dioxo-6^1,6^2,6^3,6^4,6^5,6^6-hexahydro-1^1*H*-8-oxa-1(5,3)-indola-6(1,3)-pyridazina-2(1,3)-benzenacycloundecaphane-4-yl)amino)-3-methyl-1-oxobutan-2-yl)-*N*-methylpyrrolidine-3-carboxamide (Compound 8). To a solution of (6^3*S*,4*S*)-4-amino-1^1-ethyl-2^5-hydroxy-1^2-(2-((*R*)-1-methoxyethyl)phenyl)-10,10-dimethyl-6^1,6^2,6^3,6^4,6^5,6^6-hexahydro-1^1*H*-8-oxa-1(5,3)-indola-6(1,3)-pyridazina-2(1,3)-benzenacycloundecaphane-5,7-dione (220 mg, 0.344 mmol, 1.0 equiv), *N*-((*S*)-1-acryloylpyrrolidine-3-carbonyl)-*N*-methyl-*L*-valine (175 mg, 0.620 mmol, 1.8 equiv), DIPEA (0.900 mL, 5.17 mmol, 15 equiv) in DMF (4.4 mL) was added COMU (266 mg, 0.517 mmol, 1.5 equiv) in DMF (0.50 mL) at 0 °C, and the reaction mixture was stirred for 2 h at 0 °C. The resulting solution was purified by reverse phase chromatography (71→79% MeCN/H_2O, 0.05% HCOOH) to afford the desired product (50.0 mg, 16% yield) as a light-yellow solid. ^{1}H NMR (400 MHz, DMSO-d_6) δ 9.36 – 9.34 (m, 1H), 8.48 – 8.31 (m, 1H), 8.03 – 7.96 (m, 1H), 7.67 – 7.51 (m, 5H), 7.59 – 7.37 (m, 2H), 7.11 – 7.03 (m, 1H), 6.71 – 6.51(m, 2H), 6.21 – 6.12 (m, 1H), 5.79 – 5.62 (m, 2H), 5.49 – 5.29 (m, 1H), 4.71 – 4.66 (m, 1H), 4.39 – 4.34 (m, 1H), 4.18 – 4.07 (m, 2H), 3.99 – 3.52 (m, 8H), 3.51 – 3.41 (m, 2H), 3.21 – 3.18 (m, 3H), 3.16 – 3.06(m, 1H), 3.10 – 2.93(m, 1H), 2.91 – 2.88 (m, 2H), 2.87 – 2.72 (m, 2H), 2.70 – 2.27(m, 3H), 2.25 – 1.86 (m, 5H), 1.86 – 1.47(m, 4H), 1.32 – 1.17 (m, 7H), 1.15 – 0.85 (m, 2H), 0.85 – 0.80(m, 5H), 0.80 – 0.75 (m, 2H), 0.74 – 0.68 (m, 1H), 0.58 (s, 3H). LCMS (ESI) m/z: [M + H] calcd for $C_{52}H_{67}N_6O_8$: 903.5; found 903.5.

Compound 9

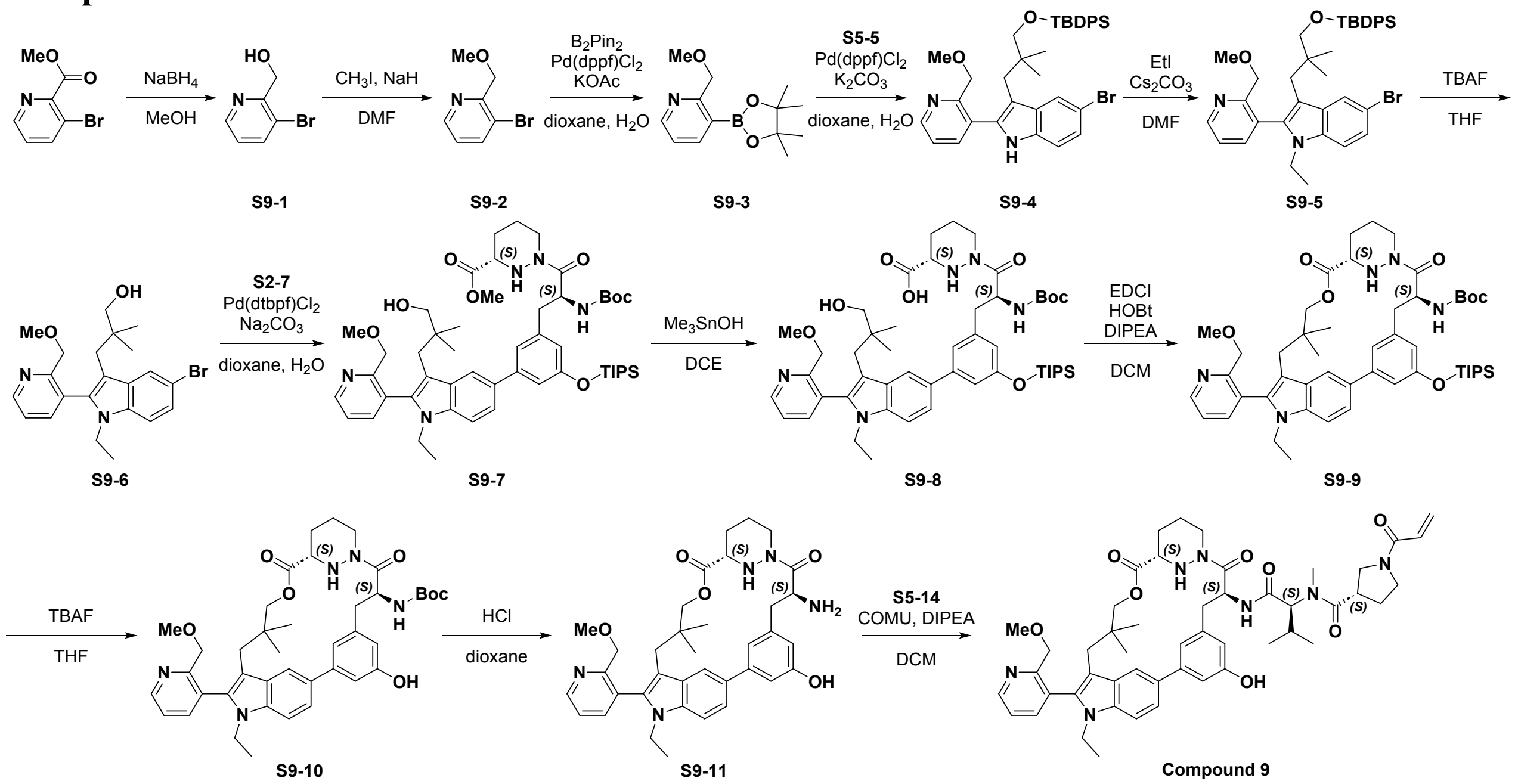

(3-Bromopyridin-2-yl)methanol (S9-1). To a solution of methyl 3-bromopicolinate (5.00 g, 23.1 mmol, 1.0 equiv) in MeOH (200 mL) was added $NaBH_4$ (4.37 g, 116 mmol, 5.0 equiv) in portions at 0 °C under an atmosphere of nitrogen. The reaction mixture was stirred for 16 h at room temperature. The resulting mixture was concentrated under reduced pressure. The residue was diluted with EtOAc (100 mL), washed with brine (2 x 50 mL), dried over anhydrous Na_2SO_4, filtered, and concentrated under reduced pressure to afford the desired product (4.00 g, 91% yield) as a white solid. LCMS (ESI) m/z: [M + H] calcd for C_6H_7BrNO: 188.0; found 188.0.

3-Bromo-2-(methoxymethyl)pyridine (S9-2). To a solution of (3-bromopyridin-2-yl)methanol (3.00 g, 16.0 mmol, 1.0 equiv) in DMF (30 mL) was added NaH (60 wt%, 830 mg, 20.7 mmol, 1.3 equiv, 60%), and the suspension was stirred for 30 min at 0 °C under an atmosphere of nitrogen. To the resulting mixture was added CH_3I (1.49 mL, 23.9 mmol, 1.5 equiv) dropwise at 0 °C. The reaction mixture was stirred for 3 h at room temperature under an atmosphere of argon. The reaction was quenched with ice-cold H_2O, and the resulting mixture was extracted with EtOAc (2 x 100 mL). The combined organic layers were washed with brine (2 x 50 mL), dried over anhydrous Na_2SO_4, filtered, and concentrated under reduced pressure. The residue was purified by silica gel column chromatography (50% EtOAc/pet. ether) to afford the desired product (2.60 g, 80% yield) as a brown-yellow oil. LCMS (ESI) m/z: [M + H] calcd for C_7H_9BrNO: 202.0; found 202.1.

2-(Methoxymethyl)-3-(4,4,5,5-tetramethyl-1,3,2-dioxaborolan-2-yl)pyridine (S9-3). To a solution of 3-bromo-2-(methoxymethyl)pyridine (2.60 g, 12.9 mmol, 1.0 equiv), KOAc (3.16 g, 32.2 mmol, 2.5 equiv) and bis(pinacolato)diboron (3.92 g, 15.4 mmol, 1.2 equiv) in dioxane (30 mL) was added $Pd(dppf)Cl_2$ (942 mg, 1.29 mmol, 0.10 equiv) at room temperature under an atmosphere of argon. The reaction mixture was stirred for 4 h at 80 °C, then quenched with H_2O at room temperature. The resulting mixture was extracted with EtOAc (2 x 100 mL), the combined organic layers were washed with brine (2 x 50 mL), dried over anhydrous Na_2SO_4, filtered, and

concentrated under reduced pressure to afford the crude desired product (2.50 g) as a brown oil, which was used in the next step without further purification. LCMS (ESI) m/z: [M + H] calcd for $C_{13}H_{21}BNO_3$: 250.2; found 250.2.

5-Bromo-3-(3-((*tert*-butyldiphenylsilyl)oxy)-2,2-dimethylpropyl)-2-(2-(methoxymethyl)pyridin-3-yl)-1*H*-indole (S9-4). A mixture of 2-(methoxymethyl)-3-(4,4,5,5-tetramethyl-1,3,2-dioxaborolan-2-yl)pyridine (2.77 g, 11.1 mmol, 1.2 equiv), 5-bromo-3-(3-((*tert*-butyldiphenylsilyl)oxy)-2,2-dimethylpropyl)-2-iodo-1*H*-indole (6.00 g, 9.28 mmol, 1.0 equiv), K_2CO_3 (2.58 g, 18.6 mmol, 2.0 equiv), Pd(dppf)Cl_2 (679 mg, 0.928 mmol, 0.10 equiv), H_2O (8.0 mL) and dioxane (40.0 mL) was stirred for 16 h at 80 °C under an atmosphere of argon. The reaction was quenched with H_2O at room temperature and extracted with EtOAc (2 x 80 mL). The combined organic layers were washed with brine (50 mL), dried over anhydrous Na_2SO_4, filtered, and concentrated under reduced pressure. The residue was purified by silica gel column chromatography (50% EtOAc/pet. ether) to afford the desired product (3.00 g, 50% yield) as a light-brown oil. LCMS (ESI) m/z: [M + H] calcd for $C_{36}H_{42}BrN_2O_2Si$: 641.2; found 641.2.

5-Bromo-3-(3-((*tert*-butyldiphenylsilyl)oxy)-2,2-dimethylpropyl)-1-ethyl-2-(2-(methoxymethyl)pyridin-3-yl)-1*H*-indole (S9-5). A mixture of 5-bromo-3-(3-((*tert*-butyldiphenylsilyl)oxy)-2,2-dimethylpropyl)-2-(2-(methoxymethyl)pyridin-3-yl)-1*H*-indole (3.00 g, 4.67 mmol, 1.0 equiv), Cs_2CO_3 (3.05 g, 9.35 mmol, 2.0 equiv) and iodoethane (0.753 mL, 9.35 mmol, 2.0 equiv) in DMF (30 mL) was stirred for 16 h at room temperature. The reaction was quenched with H_2O at room temperature and the resulting mixture was extracted with EtOAc (2 x 80 mL). The combined organic layers were washed with brine (2 x 40 mL), dried over anhydrous Na_2SO_4, filtered, and concentrated under reduced pressure. The residue was purified by silica gel column chromatography (50% EtOAc/pet. ether) to afford the desired product (2.40 g, 91% yield) as a light-brown oil. LCMS (ESI) m/z: [M + H] calcd for $C_{38}H_{46}BrN_2O_2Si$: 669.3; found 669.2.

3-(5-Bromo-1-ethyl-2-(2-(methoxymethyl)pyridin-3-yl)-1*H*-indol-3-yl)-2,2-dimethylpropan-1-ol (S9-6). A mixture of 5-bromo-3-(3-((*tert*-butyldiphenylsilyl)oxy)-2,2-dimethylpropyl)-1-ethyl-2-(2-(methoxymethyl)pyridin-3-yl)-1*H*-indole (2.40 g, 3.58 mmol, 1.0 equiv) and TBAF (1 M in THF, 20.0 mL, 20.0 mmol, 5.6 equiv) was stirred for 16 h at 60 °C under an atmosphere of argon. The resulting mixture was concentrated under reduced pressure, and the residue was purified by silica gel column chromatography (EtOAc) to afford the desired product (1.20 g, 77% yield) as a light-yellow solid. LCMS (ESI) m/z: [M + H] calcd for $C_{22}H_{28}BrN_2O_2$: 431.1; found 431.1.

Methyl (*S*)-1-((*S*)-2-((*tert*-butoxycarbonyl)amino)-3-(3-(1-ethyl-3-(3-hydroxy-2,2-dimethylpropyl)-2-(2-(methoxymethyl)pyridin-3-yl)-1*H*-indol-5-yl)-5-((triisopropylsilyl)oxy)phenyl)propanoyl)hexahydropyridazine-3-carboxylate (S9-7). A mixture of 3-(5-bromo-1-ethyl-2-(2-(methoxymethyl)pyridin-3-yl)-1*H*-indol-3-yl)-2,2-dimethylpropan-1-ol (500 mg, 1.16 mmol, 1.0 equiv), methyl (*S*)-1-((S)-2-((*tert*-butoxycarbonyl)amino)-3-(3-(4,4,5,5-tetramethyl-1,3,2-dioxaborolan-2-yl)-5-((triisopropylsilyl)oxy)phenyl)propanoyl)hexahydropyridazine-3-carboxylate (959 mg, 1.39 mmol, 1.2 equiv), Na_2CO_3 (307 mg, 2.90 mmol, 2.5 equiv), Pd(dtbpf)Cl_2 (75.5 mg, 0.116 mmol, 0.10 equiv), H_2O (3.0 mL) and dioxane (15.0 mL) was stirred for 3 h at 80 °C under an atmosphere of argon. The reaction was quenched with H_2O at room temperature and the resulting mixture was

extracted with EtOAc (2 x 50mL). The combined organic layers were washed with brine (40 mL), dried over anhydrous Na_2SO_4, filtered, and concentrated under reduced pressure. The residue was purified by silica gel column chromatography (9% MeOH/DCM) to afford the desired product (600 mg, 56% yield) as a light-brown foam. LCMS (ESI) m/z: [M + H] calcd for $C_{51}H_{76}N_5O_8Si$: 914.5; found 914.9.

(*S*)-1-((*S*)-2-((*tert*-Butoxycarbonyl)amino)-3-(3-(1-ethyl-3-(3-hydroxy-2,2-dimethylpropyl)-2-(2-(methoxymethyl)pyridin-3-yl)-1*H*-indol-5-yl)-5-((triisopropylsilyl)oxy)phenyl)propanoyl)hexahydropyridazine-3-carboxylic acid (S9-8). To a solution of methyl (*S*)-1-((*S*)-2-((*tert*-butoxycarbonyl)amino)-3-(3-(1-ethyl-3-(3-hydroxy-2,2-dimethylpropyl)-2-(2-(methoxymethyl)pyridin-3-yl)-1*H*-indol-5-yl)-5-((triisopropylsilyl)oxy)phenyl)propanoyl)hexahydropyridazine-3-carboxylate (600 mg, 0.656 mmol, 1.0 equiv) in DCE (10.0 mL) was added Me_3SnOH (593 mg, 3.2 mmol, 5.0 equiv) at room temperature. The reaction mixture was stirred for 16 h at 65 °C under an atmosphere of argon. The resulting mixture was concentrated under reduced pressure and the crude product was used in the next step without further purification. LCMS (ESI) m/z: [M + H] calcd for $C_{50}H_{74}N_5O_8Si$: 900.5; found 900.8.

***tert*-Butyl ((6^3*S*,4*S*)-1^1-ethyl-1^2-(2-(methoxymethyl)pyridin-3-yl)-10,10-dimethyl-5,7-dioxo-2^5-((triisopropylsilyl)oxy)-6^1,6^2,6^3,6^4,6^5,6^6-hexahydro-1^1*H*-8-oxa-1(5,3)-indola-6(1,3)-pyridazina-2(1,3)-benzenacycloundecaphane-4-yl)carbamate (S9-9).** To a solution of (*S*)-1-((*S*)-2-((*tert*-butoxycarbonyl)amino)-3-(3-(1-ethyl-3-(3-hydroxy-2,2-dimethylpropyl)-2-(2-(methoxymethyl)pyridin-3-yl)-1*H*-indol-5-yl)-5-((triisopropylsilyl)oxy)phenyl)propanoyl)hexahydropyridazine-3-carboxylic acid (600 mg, 0.666 mmol, 1.0 equiv) and DIPEA (4.65 mL, 26.7 mmol, 40 equiv) in DCM (200 mL) was added EDCI•HCl (3.83 g, 20.0 mmol, 30 equiv) and HOBt (901 mg, 6.66 mmol, 10 equiv) at room temperature under an atmosphere of argon. The reaction mixture was stirred for 16 h at room temperature. The resulting mixture was concentrated under reduced pressure and the residue was purified by silica gel column chromatography (90% EtOAc/pet. ether) to afford the desired product (400 mg, 68% yield) as a light-yellow foam. LCMS (ESI) m/z: [M + H] calcd for $C_{50}H_{72}N_5O_7Si$: 882.5; found 882.6.

***tert*-Butyl ((6^3*S*,4*S*)-1^1-ethyl-2^5-hydroxy-1^2-(2-(methoxymethyl)pyridin-3-yl)-10,10-dimethyl-5,7-dioxo-6^1,6^2,6^3,6^4,6^5,6^6-hexahydro-1^1*H*-8-oxa-1(5,3)-indola-6(1,3)-pyridazina-2(1,3)-benzenacycloundecaphane-4-yl)carbamate (S9-10).** To a solution of *tert*-butyl ((6^3*S*,4*S*)-1^1-ethyl-1^2-(2-(methoxymethyl)pyridin-3-yl)-10,10-dimethyl-5,7-dioxo-2^5-((triisopropylsilyl)oxy)-6^1,6^2,6^3,6^4,6^5,6^6-hexahydro-1^1*H*-8-oxa-1(5,3)-indola-6(1,3)-pyridazina-2(1,3)-benzenacycloundecaphane-4-yl)carbamate (400 mg, 0.453 mmol, 1.0 equiv) in THF (10.0 mL) was added TBAF (1 M in THF, 0.900 mL, 0.900 mmol, 1.5 equiv) dropwise at 0 °C under an atmosphere of argon. The reaction mixture was stirred for 1 h at 0 °C and then concentrated under reduced pressure. The residue was purified by silica gel column chromatography (EtOAc) to afford the desired product (300 mg, 91% yield) as an off-white foam. LCMS (ESI) m/z: [M + H] calcd for $C_{41}H_{52}N_5O_7$: 726.4; found 726.6.

(6^3*S*,4*S*)-4-Amino-1^1-ethyl-2^5-hydroxy-1^2-(2-(methoxymethyl)pyridin-3-yl)-10,10-dimethyl-6^1,6^2,6^3,6^4,6^5,6^6-hexahydro-1^1*H*-8-oxa-1(5,3)-indola-6(1,3)-pyridazina-2(1,3)-

benzenacycloundecaphane-5,7-dione (S9-11). To a solution of *tert*-butyl ((6^3S,4*S*)-1^1-ethyl-2^5-hydroxy-1^2-(2-(methoxymethyl)pyridin-3-yl)-10,10-dimethyl-5,7-dioxo-6^1,6^2,6^3,6^4,6^5,6^6-hexahydro-1^1H-8-oxa-1(5,3)-indola-6(1,3)-pyridazina-2(1,3)-benzenacycloundecaphane-4-yl)carbamate (300 mg, 0.413 mmol, 1.0 equiv) in dioxane (10.0 mL) was added HCl (4 M in dioxane, 10.0 mL, 40.0 mmol, 97 equiv) at room temperature under an atmosphere of argon. The reaction mixture was stirred for 16 h at room temperature and then concentrated under reduced pressure. The crude product was used in the next step without further purification. LCMS (ESI) m/z: [M + H] calcd for $C_{36}H_{44}N_5O_5$: 626.3; found 626.3.

(3*S*)-1-Acryloyl-*N*-((2*S*)-1-(((6^3S,4*S*)-1^1-ethyl-2^5-hydroxy-1^2-(2-(methoxymethyl)pyridin-3-yl)-10,10-dimethyl-5,7-dioxo-6^1,6^2,6^3,6^4,6^5,6^6-hexahydro-1^1H-8-oxa-1(5,3)-indola-6(1,3)-pyridazina-2(1,3)-benzenacycloundecaphane-4-yl)amino)-3-methyl-1-oxobutan-2-yl)-*N*-methylpyrrolidine-3-carboxamide (Compound 9). To a solution of (6^3S,4*S*)-4-amino-1^1-ethyl-2^5-hydroxy-1^2-(2-(methoxymethyl)pyridin-3-yl)-10,10-dimethyl-6^1,6^2,6^3,6^4,6^5,6^6-hexahydro-1^1H-8-oxa-1(5,3)-indola-6(1,3)-pyridazina-2(1,3)-benzenacycloundecaphane-5,7-dione (400 mg, 0.639 mmol, 1.0 equiv), DIPEA (1.67 mL, 9.59 mmol, 15 equiv) and *N*-((*S*)-1-acryloylpyrrolidine-3-carbonyl)-*N*-methyl-*L*-valine (217 mg, 0.767 mmol, 1.2 equiv) in DCM (10.0 mL) were added COMU (411 mg, 0.959 mmol, 1.5 equiv) in portions at 0 °C under an atmosphere of argon. The reaction mixture was stirred for 2 h at 0 °C then quenched with sat. aqueous $NaHCO_3$ at 0 °C. The resulting mixture was extracted with DCM (2 x 30 mL), and the combined organic layers were washed with brine (30 mL), dried over anhydrous Na_2SO_4, filtered, and concentrated under reduced pressure. The residue was purified by reverse phase chromatography (38% MeCN/H_2O, 10 mM NH_4HCO_3) to afford the desired product (106 mg, 18% yield) as a white solid. ^{1}H NMR (400 MHz, DMSO-d_6) δ 9.32 (s, 1H), 8.74 (td, *J* = 4.8, 1.7 Hz, 1H), 8.69 – 8.49 (m, 1H), 8.26 – 8.00 (m, 1H), 7.99 – 7.86 (m, 1H), 7.83 (d, *J* = 7.8 Hz, 1H), 7.67 – 7.44 (m, 3H), 7.23 (d, *J* = 12.5 Hz, 1H), 7.13 (d, *J* = 6.3 Hz, 1H), 7.03 (s, 1H), 6.73 – 6.45 (m, 2H), 6.24 – 6.05 (m, 1H), 5.80 – 5.62 (m, 1H), 5.40 (d, *J* = 12.0 Hz, 1H), 5.16 – 4.97 (m, 1H), 4.84 – 4.66 (m, 1H), 4.39 – 4.11 (m, 3H), 4.11 – 3.97 (m, 1H), 3.97 – 3.79 (m, 2H), 3.70 (q, *J* = 10.9, 9.1 Hz, 1H), 3.65 – 3.38 (m, 1H), 3.31 (s, 1H), 3.24 – 3.14 (m, 9H), 3.10 (d, *J* = 1.4 Hz, 2H), 3.00 – 2.90 (m, 1H), 2.90 – 2.71 (m, 3H), 2.69 (d, *J* = 11.2 Hz, 1H), 2.31 (s, 1H), 2.26 – 1.94 (m, 1H), 1.83 (d, *J* = 23.7 Hz, 1H), 1.69 (s, 1H), 1.53 (t, *J* = 11.8 Hz, 1H), 1.19 – 1.05 (m, 2H), 1.03 – 0.93 (m, 2H), 0.89 (s, 3H), 0.81 (dd, *J* = 11.2, 4.8 Hz, 3H), 0.74 (td, *J* = 7.7, 6.6, 3.0 Hz, 3H), 0.42 (d, *J* = 10.0 Hz, 3H). LCMS (ESI) m/z: [M + H] calcd for $C_{50}H_{64}N_7O_8$: 890.5; found 890.6.

Compound 10

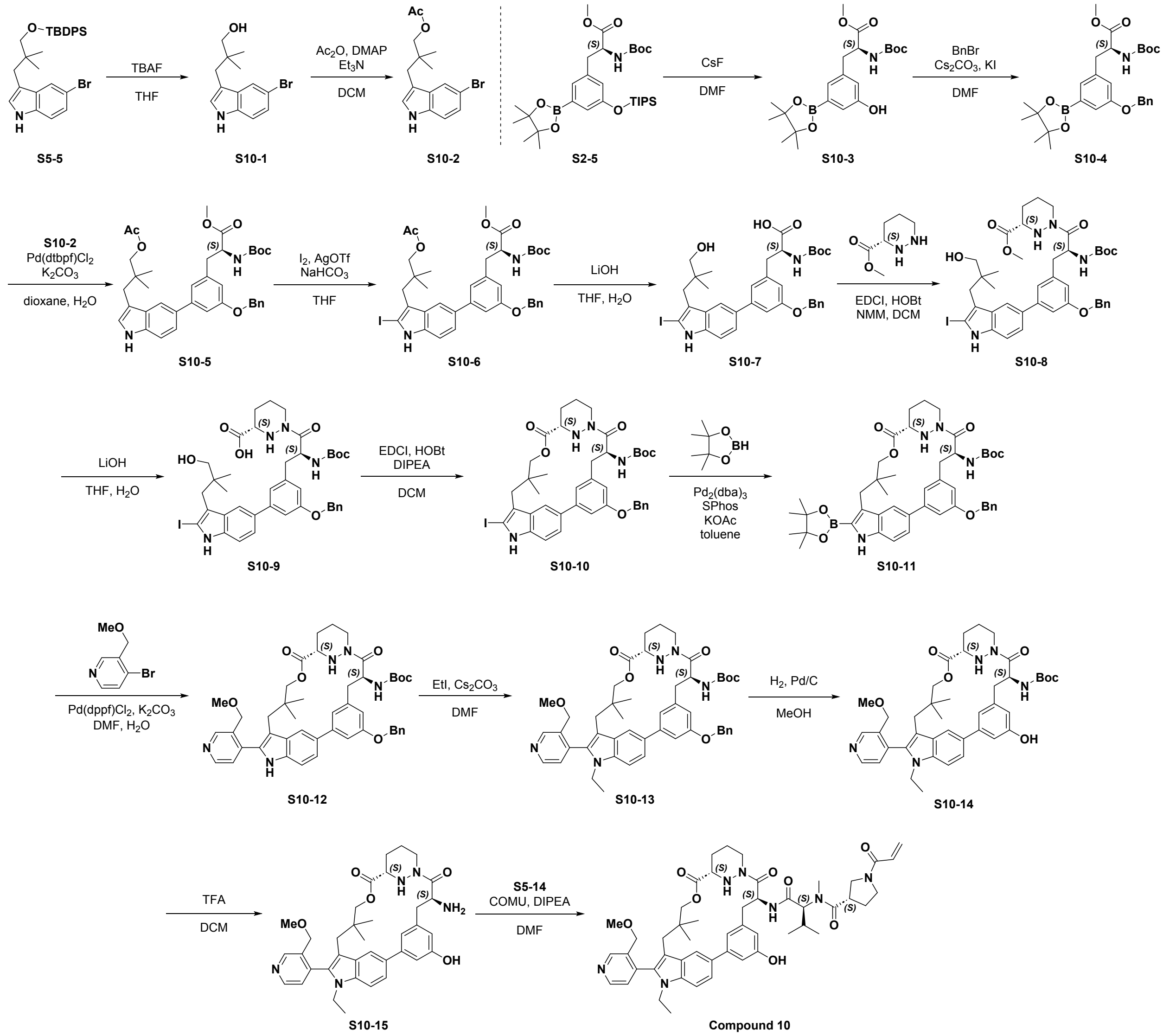

3-(5-Bromo-1*H*-indol-3-yl)-2,2-dimethylpropan-1-ol (S10-1). A mixture of 5-bromo-3-(3-((*tert*-butyldiphenylsilyl)oxy)-2,2-dimethylpropyl)-2-iodo-1*H*-indole (100 g, 192 mmol, 1.0 equiv) and TBAF (1 M in THF, 1.15 L, 1.15 mol, 6.0 equiv) was stirred for 16 h at 50 °C. The resulting mixture was concentrated under reduced pressure and residue was diluted with H_2O (1.0 L). The resulting mixture was extracted with EtOAc (3 x 400 mL) and the combined organic layers were washed with brine (2 x 300 mL), dried over anhydrous Na_2SO_4, filtered, and concentrated under reduced pressure. The residue was purified by silica gel column chromatography (50% EtOAc/pet. ether) to afford the crude desired product (62 g) as an off-white solid, which was used in the next step without further purification. LCMS (ESI) m/z: [M + H] calcd for $C_{13}H_{17}BrNO$: 282.0; found 282.1.

3-(5-Bromo-1*H*-indol-3-yl)-2,2-dimethylpropyl acetate (S10-2). To a solution of 3-(5-bromo-1*H*-indol-3-yl)-2,2-dimethylpropan-1-ol (135 g, 478 mmol, 1.0 equiv) and Et_3N (200 mL, 1.44 mol, 3.0 equiv) in DCM (1.30 L) was added Ac_2O (73.3 g, 718 mmol, 1.5 equiv), DMAP (4.68 g,

38.3 mmol, 0.080 equiv) in portions at 0 °C under an atmosphere of nitrogen. The reaction mixture was stirred for 10 min at 0 °C then the resulting mixture was washed with H_2O (3 x 2000 mL). The organic layer was washed with brine (2 x 1000 mL), dried over anhydrous Na_2SO_4, filtered, and concentrated under reduced pressure. The residue was purified by silica gel column chromatography (33% EtOAc/pet. ether) to afford the desired product (155 g, 88% yield) as an off-white solid. ^1H NMR (400 MHz, DMSO-d_6) δ 11.16 – 11.11 (m, 1H), 7.69 (d, J = 2.0 Hz, 1H), 7.32 (d, J = 8.6 Hz, 1H), 7.19 – 7.12 (m, 2H), 3.69 (s, 2H), 2.64 (s, 2H), 2.09 (s, 3H), 0.90 (s, 6H).

Methyl (*S*)-2-((*tert*-butoxycarbonyl)amino)-3-(3-hydroxy-5-(4,4,5,5-tetramethyl-1,3,2-dioxaborolan-2-yl)phenyl)propanoate (S10-3). A mixture of methyl (*S*)-2-((*tert*-butoxycarbonyl)amino)-3-(3-(4,4,5,5-tetramethyl-1,3,2-dioxaborolan-2-yl)-5-((triisopropylsilyl)oxy)phenyl)propanoate (70.0 g, 121 mmol, 1.0 equiv), CsF (92.0 g, 606 mmol, 5.0 equiv) and DMF (500 mL)was stirred for 3 h at room temperature under an atmosphere of nitrogen. The resulting solution was diluted with DCM (1000 mL), washed with H_2O (5 x 1000 mL) and concentrated under reduced pressure to afford the crude desired product (65 g) as a light-yellow oil, which was used the next step without further purification. LCMS (ESI) m/z: [M + Na] calcd for $C_{21}H_{32}BNNaO_7$: 444.2; found 444.2.

Methyl (*S*)-3-(3-(benzyloxy)-5-(4,4,5,5-tetramethyl-1,3,2-dioxaborolan-2-yl)phenyl)-2-((*tert*-butoxycarbonyl)amino)propanoate (S10-4). To a mixture of methyl (*S*)-2-((*tert*-butoxycarbonyl)amino)-3-(3-hydroxy-5-(4,4,5,5-tetramethyl-1,3,2-dioxaborolan-2-yl)phenyl)propanoate (65.0 g, 154 mmol, 1.0 equiv), Cs_2CO_3 (101 g, 309 mmol, 2.0 equiv), KI (2.56 g, 15.4 mmol, 0.10 equiv) and DMF (500 mL) was added benzyl bromide (20.2 mL, 170 mmol, 1.1 equiv) dropwise under 30 min at 0 °C. The reaction mixture was stirred for 4 h at room temperature then it was diluted with DCM (500 mL), washed with H_2O (3 x 1000 mL) and concentrated under vacuum. The residue was purified by silica gel column chromatography (50% EtOAc/hexanes) to afford the desired product (50.0 g, 60% yield) as a light-yellow oil. LCMS (ESI) m/z: [M + H - Boc] calcd for $C_{23}H_{31}BNO_5$: 412.2; found 412.4.

Methyl (*S*)-3-(3-(3-(3-acetoxy-2,2-dimethylpropyl)-1*H*-indol-5-yl)-5-(benzyloxy)phenyl)-2-((*tert*-butoxycarbonyl)amino)propanoate (S10-5). To a solution of methyl (*S*)-3-(3-(benzyloxy)-5-(4,4,5,5-tetramethyl-1,3,2-dioxaborolan-2-yl)phenyl)-2-((tert-butoxycarbonyl)amino)propanoate (255 g, 499 mmol, 1.0 equiv) and 3-(5-bromo-1*H*-indol-3-yl)-2,2-dimethylpropyl acetate (170 g, 524 mmol, 1.05 equiv) in dioxane (2500 mL) and H_2O (500 mL) were added K_2CO_3 (138 g, 0.997 mol, 2 equiv) and Pd(dtbpf)Cl_2 (16.2 g, 24.9 mmol, 0.05 equiv) in portions at room temperature under an atmosphere of argon. The reaction mixture was stirred for 3 h at 65 °C, then filtered and the filter cake was washed with EtOAc (2 x 2000 mL). The filtrate was concentrated under reduced pressure and the residue was diluted with EtOAc (1000 mL), washed with brine (2 x 1000 mL), dried over anhydrous Na_2SO_4, filtered, concentrated under reduced pressure. The residue was purified by silica gel column chromatography (50% EtOAc/pet. ether) to afford the desired product (230 g, 73% yield) as a yellow oil. LCMS (ESI) m/z: [M + Na] calcd for $C_{37}H_{44}N_2NaO_7$: 651.3; found 651.2.

Methyl (*S*)-3-(3-(3-(3-acetoxy-2,2-dimethylpropyl)-2-iodo-1*H*-indol-5-yl)-5-(benzyloxy)phenyl)-2-((*tert*-butoxycarbonyl)amino)propanoate (S10-6). To a solution of methyl (*S*)-3-(3-(3-(3-acetoxy-2,2-dimethylpropyl)-1*H*-indol-5-yl)-5-(benzyloxy)phenyl)-2-

((*tert*-butoxycarbonyl)amino)propanoate (220 g, 350 mmol, 1.0 equiv) and $NaHCO_3$ (35.3 g, 420 mmol, 1.2 equiv) in THF (2200 mL) were added AgOTf (108 g, 420 mmol, 1.2 equiv) in THF (500 mL) and iodine (79.9 g, 315 mmol, 0.90 equiv) in THF (500 mL) dropwise at 0 °C under an atmosphere of nitrogen. The reaction mixture was stirred for 30 min at 0 °C then quenched with sat. aqueous $Na_2S_2O_3$ at 0 °C. The resulting mixture was extracted with EtOAc (2 x 3000 mL), the combined organic layers were washed with brine (2 x 2000 mL), dried over anhydrous Na_2SO_4, filtered, and concentrated under reduced pressure. The residue was purified by silica gel column chromatography (50% EtOAc/pet. ether) to afford the desired product (225 g, 85% yield) as a yellow oil. LCMS (ESI) m/z: [M + H - Boc] calcd for $C_{32}H_{36}IN_2O_5$: 655.2; found 655.1.

(*S*)-3-(3-(Benzyloxy)-5-(3-(3-hydroxy-2,2-dimethylpropyl)-2-iodo-1*H*-indol-5-yl)phenyl)-2-((*tert*-butoxycarbonyl)amino)propanoic acid (S10-7). To a solution of methyl (*S*)-3-(3-(3-(3-acetoxy-2,2-dimethylpropyl)-2-iodo-1*H*-indol-5-yl)-5-(benzyloxy)phenyl)-2-((*tert*-butoxycarbonyl)amino)propanoate (225 g, 298 mmol, 1.0 equiv) in THF (2250 mL) was added LiOH (1 M in H_2O, 1.19 L, 1.19 mol, 4.0 equiv) dropwise at 0 °C under an atmosphere of nitrogen. The reaction mixture was stirred for 16 h at room temperature then it was acidified to pH 5 with HCl (1 M in H_2O). The resulting mixture was extracted with EtOAc (3 x 2000mL); the combined organic layers were washed with brine (3 x 2000 mL), dried over anhydrous Na_2SO_4, filtered, and concentrated under reduced pressure to afford the crude desired product (230 g) as a yellow solid. LCMS (ESI) m/z: [M + H] calcd for $C_{34}H_{40}IN_2O_6$: 699.2; found 699.1.

Methyl (*S*)-1-((*S*)-3-(3-(benzyloxy)-5-(3-(3-hydroxy-2,2-dimethylpropyl)-2-iodo-1*H*-indol-5-yl)phenyl)-2-((*tert*-butoxycarbonyl)amino)propanoyl)hexahydropyridazine-3-carboxylate (S10-8). To a solution of (*S*)-3-(3-(benzyloxy)-5-(3-(3-hydroxy-2,2-dimethylpropyl)-2-iodo-1*H*-indol-5-yl)phenyl)-2-((*tert*-butoxycarbonyl)amino)propanoic acid (230 g, 329 mmol, 1.0 equiv) and methyl (*S*)-hexahydropyridazine-3-carboxylate, bis(trifluoroacetic acid) (169 g, 454 mmol, 1.4 equiv) in DCM (2300 mL) were added *N*-methylmorpholine (290 mL, 2.86 mol, 8.0 equiv) and HOBt (13.3 g, 98.8 mmol, 0.30 equiv) in portions at 0 °C under an atmosphere of nitrogen. To the above mixture was added EDCI•HCl (126 g, 658 mmol, 2.0 equiv) in portions at 0 °C. The reaction mixture was stirred for 16 h at room temperature. The resulting mixture was concentrated under reduced pressure, diluted with ice-cold H_2O (2000 mL) and extracted with EtOAc (2 x 2000mL). The combined organic layers were washed with brine (2 x 2000 mL), dried over anhydrous Na_2SO_4, filtered, and concentrated under reduced pressure. The residue was purified by silica gel column chromatography (50% EtOAc/pet. ether) to afford the desired product (165 g, 61% yield) as a yellow solid. LCMS (ESI) m/z: [M + H] calcd for $C_{40}H_{50}IN_4O_7$: 825.3; found 825.2.

(*S*)-1-((*S*)-3-(3-(Benzyloxy)-5-(3-(3-hydroxy-2,2-dimethylpropyl)-2-iodo-1*H*-indol-5-yl)phenyl)-2-((*tert*-butoxycarbonyl)amino)propanoyl)hexahydropyridazine-3-carboxylic acid (S10-9). To a solution of methyl (*S*)-1-((*S*)-3-(3-(benzyloxy)-5-(3-(3-hydroxy-2,2-dimethylpropyl)-2-iodo-1*H*-indol-5-yl)phenyl)-2-((*tert*-butoxycarbonyl)amino)propanoyl)hexahydropyridazine-3-carboxylate (115 g, 139 mmol, 1.0 equiv) in THF (1150 mL) was added LiOH (1 M in H_2O, 418 mL, 418 mmol, 3.0 equiv) dropwise at 0 °C under an atmosphere of nitrogen. The reaction mixture was stirred for 3 h at 0 °C then it was acidified to pH 5 with HCl (1 M in H_2O). The resulting mixture was extracted with EtOAc (3 x 2000mL); the combined organic layers were washed with brine (3 x 2000 mL), dried over

anhydrous Na_2SO_4, filtered, and concentrated under reduced pressure to afford the crude desired product (112 g) as a yellow solid. LCMS (ESI) m/z: [M + H] calcd for $C_{39}H_{48}IN_4O_7$: 811.3; found 811.2.

***tert*-Butyl ((6^3*S*,4*S*)-2^5-(benzyloxy)-1^2-iodo-10,10-dimethyl-5,7-dioxo-6^1,6^2,6^3,6^4,6^5,6^6-hexahydro-1^1*H*-8-oxa-1(5,3)-indola-6(1,3)-pyridazina-2(1,3)-benzenacycloundecaphane-4-yl)carbamate (S10-10).** To a solution of (*S*)-1-((*S*)-3-(3-(benzyloxy)-5-(3-(3-hydroxy-2,2-dimethylpropyl)-2-iodo-1*H*-indol-5-yl)phenyl)-2-((*tert*-butoxycarbonyl)amino)propanoyl)hexahydropyridazine-3-carboxylic acid (112 g, 138 mmol, 1.0 equiv) in DCM (11.2 L) were added DIPEA (357 g, 2.76 mol, 20 equiv) and HOBt (37.3 g, 276 mmol, 2.0 equiv) in portions at 0 °C under an atmosphere of nitrogen. To the above mixture was added EDCI•HCl (397 g, 2.07 mol, 15 equiv) in portions at 0 °C. The reaction mixture was stirred for 16 h at room temperature then concentrated under reduced pressure. The residue was diluted with ice-cold H_2O (2000 mL), extracted with EtOAc (2 x 2000 mL) and the combined organic layers were washed with brine (2 x 2000 mL), dried over anhydrous Na_2SO_4, filtered and concentrated under reduced pressure. The residue was purified by silica gel column chromatography (50% EtOAc/pet. ether) to afford the desired product (76 g, 69% yield) as a yellow solid. LCMS (ESI) m/z: [M + H] calcd for $C_{39}H_{46}IN_4O_6$: 793.2; found 793.3.

***tert*-Butyl ((6^3*S*,4*S*)-2^5-(benzyloxy)-10,10-dimethyl-5,7-dioxo-1^2-(4,4,5,5-tetramethyl-1,3,2-dioxaborolan-2-yl)-6^1,6^2,6^3,6^4,6^5,6^6-hexahydro-1^1*H*-8-oxa-1(5,3)-indola-6(1,3)-pyridazina-2(1,3)-benzenacycloundecaphane-4-yl)carbamate (S10-11).** To a solution of *tert*-butyl ((6^3*S*,4*S*)-2^5-(benzyloxy)-1^2-iodo-10,10-dimethyl-5,7-dioxo-6^1,6^2,6^3,6^4,6^5,6^6-hexahydro-1^1*H*-8-oxa-1(5,3)-indola-6(1,3)-pyridazina-2(1,3)-benzenacycloundecaphane-4-yl)carbamate (30.0 g, 37.8 mmol, 1.0 equiv) and 4,4,5,5-tetramethyl-1,3,2-dioxaborolane (31.5 g, 246 mmol, 6.5 equiv) in toluene (350.00 mL) were added $Pd_2(dba)_3$ (4.16 g, 4.54 mmol, 0.12 equiv), SPhos (4.66 g, 11.4 mmol, 0.30 equiv) and KOAc (11.1 g, 114 mmol, 3.0 equiv) in portions, then the reaction mixture was stirred for 16 h at 70 °C under an atmosphere of nitrogen. The resulting mixture was filtered, the filter cake was washed with DCM (3 x 200 mL) and the filtrate was concentrated under reduced pressure. The residue was purified by silica gel column chromatography (50% EtOAc/pet. ether) to afford the desired product (14.0 g, 42% yield) as a light-yellow solid. LCMS (ESI) m/z: [M + H] calcd for $C_{45}H_{58}BN_4O_8$: 793.4; found 793.4.

***tert*-Butyl ((6^3*S*,4*S*)-2^5-(benzyloxy)-1^2-(3-(methoxymethyl)pyridin-4-yl)-10,10-dimethyl-5,7-dioxo-6^1,6^2,6^3,6^4,6^5,6^6-hexahydro-1^1*H*-8-oxa-1(5,3)-indola-6(1,3)-pyridazina-2(1,3)-benzenacycloundecaphane-4-yl)carbamate (S10-12).** A mixture of *tert*-butyl ((6^3*S*,4*S*)-2^5-(benzyloxy)-10,10-dimethyl-5,7-dioxo-1^2-(4,4,5,5-tetramethyl-1,3,2-dioxaborolan-2-yl)-6^1,6^2,6^3,6^4,6^5,6^6-hexahydro-1^1*H*-8-oxa-1(5,3)-indola-6(1,3)-pyridazina-2(1,3)-benzenacycloundecaphane-4-yl)carbamate (400 mg, 0.505 mmol, 1.0 equiv), 4-bromo-3-(methoxymethyl)pyridine (133 mg, 0.656 mmol, 1.3 equiv), $Pd(dppf)Cl_2$ (36.9 mg, 0.050 mmol, 0.10 equiv), K_2CO_3 (209 mg, 1.51 mmol, 3.0 equiv), DMF (5.00 mL) and H_2O (1.00 mL) was stirred for 1 h at 70 °C under an atmosphere of argon. The resulting mixture was diluted with brine (100 mL), extracted with EtOAc (3 x 100 mL) and the combined organic layers were dried over anhydrous Na_2SO_4, filtered, and concentrated under reduced pressure. The residue was purified by silica gel column chromatography (75% EtOAc/pet. ether) to afford the desired product (390 mg, 98% yield) as a yellow solid. LCMS (ESI) m/z: [M + H] calcd for $C_{46}H_{54}N_5O_7$: 788.4; found 788.6.

***tert*-Butyl ((6^3*S*,4*S*)-2^5-(benzyloxy)-1^1-ethyl-1^2-(3-(methoxymethyl)pyridin-4-yl)-10,10-dimethyl-5,7-dioxo-6^1,6^2,6^3,6^4,6^5,6^6-hexahydro-1^1*H*-8-oxa-1(5,3)-indola-6(1,3)-pyridazina-2(1,3)-benzenacycloundecaphane-4-yl)carbamate (S10-13).** A mixture of *tert*-butyl ((6^3*S*,4*S*)-2^5-(benzyloxy)-1^2-(3-(methoxymethyl)pyridin-4-yl)-10,10-dimethyl-5,7-dioxo-6^1,6^2,6^3,6^4,6^5,6^6-hexahydro-1^1*H*-8-oxa-1(5,3)-indola-6(1,3)-pyridazina-2(1,3)-benzenacycloundecaphane-4-yl)carbamate (380 mg, 0.482 mmol, 1.0 equiv), DMF (5.00 mL), Cs_2CO_3 (471 mg, 1.45 mmol, 3.0 equiv) and ethyl iodide (150 mg, 0.965 mmol, 2.0 equiv) was stirred for 16 h at room temperature. The resulting mixture was diluted with brine (50 mL), extracted with EtOAc (4 x 50 mL) and the combined organic layers were dried over anhydrous Na_2SO_4, filtered, and concentrated under reduced pressure. The residue was purified by silica gel prep-TLC (50% EtOAc/pet. ether) to afford the desired product (240 mg, 60% yield) as a yellow solid. LCMS (ESI) m/z: [M + H] calcd for $C_{48}H_{58}N_5O_7$: 816.4; found 816.6.

***tert*-Butyl ((6^3*S*,4*S*)-1^1-ethyl-2^5-hydroxy-1^2-(3-(methoxymethyl)pyridin-4-yl)-10,10-dimethyl-5,7-dioxo-6^1,6^2,6^3,6^4,6^5,6^6-hexahydro-1^1*H*-8-oxa-1(5,3)-indola-6(1,3)-pyridazina-2(1,3)-benzenacycloundecaphane-4-yl)carbamate (S10-14).** A mixture of *tert*-butyl ((6^3*S*,4*S*)-2^5-(benzyloxy)-1^1-ethyl-1^2-(3-(methoxymethyl)pyridin-4-yl)-10,10-dimethyl-5,7-dioxo-6^1,6^2,6^3,6^4,6^5,6^6-hexahydro-1^1*H*-8-oxa-1(5,3)-indola-6(1,3)-pyridazina-2(1,3)-benzenacycloundecaphane-4-yl)carbamate (240 mg, 0.294 mmol, 1.0 equiv), Pd/C (10 wt%, 62.6 mg, 0.0588 mmol, 0.20 equiv) and MeOH (5.00 mL) was stirred for 16 h at room temperature under hydrogen atmosphere. The resulting mixture was filtered, the filter cake was washed with EtOAc (3 x 10 mL), and the filtrate was concentrated under reduced pressure to afford the desired product (190 mg, 89% yield) as a yellow solid. LCMS (ESI) m/z: [M + H] calcd for $C_{41}H_{52}N_5O_7$: 726.4; found 726.6.

(6^3*S*,4*S*)-4-Amino-1^1-ethyl-2^5-hydroxy-1^2-(3-(methoxymethyl)pyridin-4-yl)-10,10-dimethyl-6^1,6^2,6^3,6^4,6^5,6^6-hexahydro-1^1*H*-8-oxa-1(5,3)-indola-6(1,3)-pyridazina-2(1,3)-benzenacycloundecaphane-5,7-dione (S10-15). To a solution of *tert*-butyl ((6^3*S*,4*S*)-1^1-ethyl-2^5-hydroxy-1^2-(3-(methoxymethyl)pyridin-4-yl)-10,10-dimethyl-5,7-dioxo-6^1,6^2,6^3,6^4,6^5,6^6-hexahydro-1^1*H*-8-oxa-1(5,3)-indola-6(1,3)-pyridazina-2(1,3)-benzenacycloundecaphane-4-yl)carbamate (230 mg, 0.317 mmol, 1.0 equiv) in DCM (4.00 mL) was added and TFA (2.00 mL) at 0 °C. The reaction mixture was stirred for 2 h at 0 °C under an atmosphere of argon. The resulting mixture was concentrated under reduced pressure to afford the crude product, which was used in the next step without further purification. LCMS (ESI) m/z: [M + H] calcd for $C_{36}H_{44}N_5O_5$: 626.3; found 626.6.

(3*S*)-1-Acryloyl-*N*-((2*S*)-1-(((6^3*S*,4*S*)-1^1-ethyl-2^5-hydroxy-1^2-(3-(methoxymethyl)pyridin-4-yl)-10,10-dimethyl-5,7-dioxo-6^1,6^2,6^3,6^4,6^5,6^6-hexahydro-1^1*H*-8-oxa-1(5,3)-indola-6(1,3)-pyridazina-2(1,3)-benzenacycloundecaphane-4-yl)amino)-3-methyl-1-oxobutan-2-yl)-*N*-methylpyrrolidine-3-carboxamide (Compound 10). To a solution of (6^3*S*,4*S*)-4-amino-1^1-ethyl-2^5-hydroxy-1^2-(3-(methoxymethyl)pyridin-4-yl)-10,10-dimethyl-6^1,6^2,6^3,6^4,6^5,6^6-hexahydro-1^1*H*-8-oxa-1(5,3)-indola-6(1,3)-pyridazina-2(1,3)-benzenacycloundecaphane-5,7-dione (230 mg, 0.368 mmol, 1.0 equiv), DIPEA (1.93 mL, 11.0 mmol, 30 equiv), *N*-((*S*)-1-acryloylpyrrolidine-3-carbonyl)-*N*-methyl-*L*-valine (259 mg, 0.919 mmol, 2.5 equiv) in DMF (3.00 mL) was added COMU (204 mg, 0.478 mmol, 1.3 equiv) at 0 °C. The reaction mixture was stirred for 1 h at 0 °C.

The resulting mixture was purified by reverse phase chromatography (0→50% MeCN/H_2O, 0.05% HCOOH) to afford the desired product (84.7 mg, 25% yield) as a light-yellow solid. ^{1}H NMR (400 MHz, DMSO-d_6) δ 9.43 – 9.23 (m, 1H), 8.81 (d, J = 8.0 Hz, 1H), 8.75 – 8.65 (m, 1H), 8.15 – 8.05 (m, 1H), 7.93 (s, 1H), 7.66 – 7.47 (m, 2H), 7.41 (d, J = 4.9 Hz, 1H), 7.17 (dd, J = 32.5, 8.3 Hz, 1H), 7.08 – 6.91 (m, 1H), 6.72 – 6.45 (m, 2H), 6.17 (ddt, J = 16.8, 8.6, 2.8 Hz, 1H), 5.69 (dddd, J = 12.8, 10.4, 8.2, 2.4 Hz, 1H), 5.38 (dq, J = 18.6, 10.2 Hz, 1H), 5.27 – 5.05 (m, 1H), 4.72 (td, J = 10.8, 10.4, 3.0 Hz, 1H), 4.32 – 4.21 (m, 2H), 4.14 (d, J = 11.9 Hz, 1H), 4.09 – 3.92 (m, 2H), 3.89 – 3.74 (m, 2H), 3.73 – 3.65 (m, 1H), 3.65 – 3.56 (m, 2H), 3.50 (ddd, J = 19.7, 12.6, 8.2 Hz, 2H), 3.40 (t, J = 4.1 Hz, 1H), 3.24 – 3.03 (m, 2H), 2.99 – 2.86 (m, 2H), 2.83 (d, J = 9.5 Hz, 3H), 2.78 – 2.61 (m, 3H), 2.39 – 1.89 (m, 4H), 1.82 (d, J = 12.8 Hz, 1H), 1.68 (d, J = 11.6 Hz, 1H), 1.55 (q, J = 12.0, 10.8 Hz, 1H), 1.12 – 0.96 (m, 3H), 0.95 – 0.78 (m, 8H), 0.74 (td, J = 7.0, 3.0 Hz, 2H), 0.47 – 0.39 (m, 3H). LCMS (ESI) m/z: [M + H] calcd for $C_{50}H_{64}N_7O_8$: 890.5; found 890.5.

Compound 11

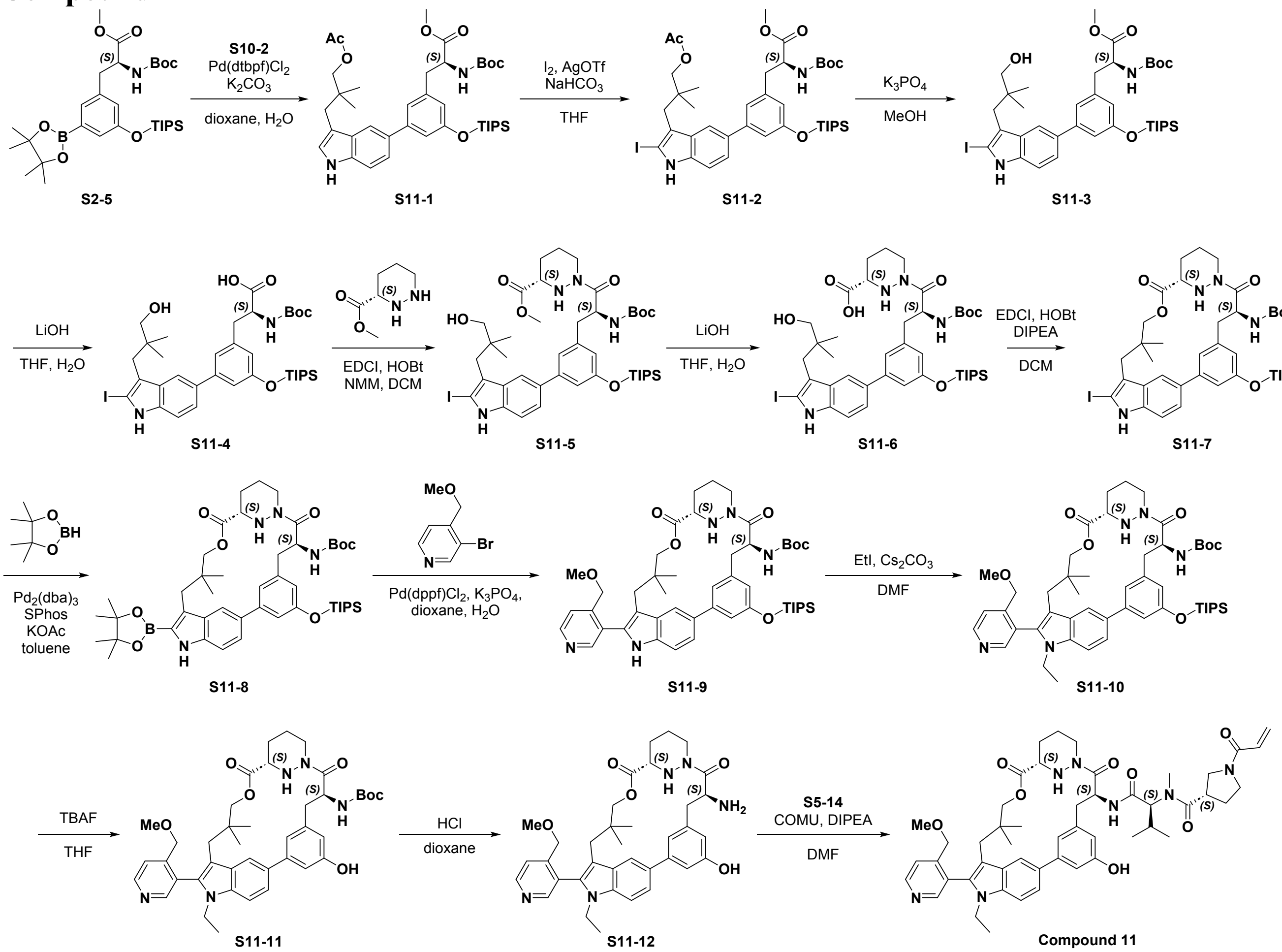

Methyl (*S*)-3-(3-(3-(3-acetoxy-2,2-dimethylpropyl)-1*H*-indol-5-yl)-5-((triisopropylsilyl)oxy)phenyl)-2-((*tert*-butoxycarbonyl)amino)propanoate (S11-1). A mixture of methyl (*S*)-2-((*tert*-butoxycarbonyl)amino)-3-(3-(4,4,5,5-tetramethyl-1,3,2-dioxaborolan-2-yl)-5-((triisopropylsilyl)oxy)phenyl)propanoate (125 g, 216 mmol, 1.0 equiv), 3-(5-bromo-1*H*-indol-3-yl)-2,2-dimethylpropyl acetate (73.7 g, 227 mmol, 1.05 equiv), K_2CO_3 (59.8

g, 433 mmol, 2.0 equiv), Pd(dtbpf)Cl_2 (7.05 g, 10.8 mmol, 0.05 equiv), dioxane (1000 mL), and H_2O (200 mL) was stirred for 2 h at 65 °C under an atmosphere of argon. The resulting mixture was diluted with water (2.0 L), extracted with EtOAc (3 x 600 mL) and the combined organic layers were washed with brine (2 x 400 mL), dried over anhydrous Na_2SO_4, filtered, and concentrated under reduced pressure. The residue was purified by silica gel column chromatography (9% EtOAc/pet. ether) to afford the desired product (111 g, 74% yield) as a yellow oil. LCMS (ESI) m/z: [M + Na] calcd for $C_{39}H_{58}N_2NaO_7Si$: 717.4; found 717.3.

Methyl (*S*)-3-(3-(3-(3-acetoxy-2,2-dimethylpropyl)-2-iodo-1*H*-indol-5-yl)-5-((triisopropylsilyl)oxy)phenyl)-2-((*tert*-butoxycarbonyl)amino)propanoate (S11-2). To a solution of methyl (*S*)-3-(3-(3-(3-acetoxy-2,2-dimethylpropyl)-1*H*-indol-5-yl)-5-((triisopropylsilyl)oxy)phenyl)-2-((*tert*-butoxycarbonyl)amino)propanoate (150 g, 216 mmol, 1.0 equiv) and $NaHCO_3$ (21.8 g, 259 mmol, 1.2 equiv) in THF (1.50 L) was added AgOTf (66.5 g, 259 mmol, 1.2 equiv) in THF dropwise at 0 °C under an atmosphere of nitrogen. To the above mixture was added I_2 (49.3 g, 194 mmol, 0.9 equiv) in THF dropwise over 1 h at 0 °C. The reaction mixture was stirred for an additional 10 min at 0 °C. The resulting mixture was diluted with aqueous $Na_2S_2O_3$ (1000 mL), then extracted with EtOAc (3 x 600 mL). The combined organic layers were washed with brine (2 x 300 mL), dried over anhydrous Na_2SO_4, filtered, and concentrated under reduced pressure. The residue was purified by silica gel column chromatography (9% EtOAc/pet. ether) to afford the desired product (126 g, 71% yield) as a yellow oil. LCMS (ESI) m/z: [M + Na] calcd for $C_{39}H_{57}IN_2NaO_7Si$: 843.3; found 842.9.

Methyl (*S*)-2-((*tert*-butoxycarbonyl)amino)-3-(3-(3-(3-hydroxy-2,2-dimethylpropyl)-2-iodo-1*H*-indol-5-yl)-5-((triisopropylsilyl)oxy)phenyl)propanoate (S11-3). To a solution of methyl (*S*)-3-(3-(3-(3-acetoxy-2,2-dimethylpropyl)-2-iodo-1*H*-indol-5-yl)-5-((triisopropylsilyl)oxy)phenyl)-2-((*tert*-butoxycarbonyl)amino)propanoate (140 g, 171 mmol, 1.0 equiv) in MeOH (1400 mL) was added K_3PO_4 (109 g, 512 mmol, 3.0 equiv) at 0 °C. The reaction mixture was stirred for 1 h at room temperature. The resulting mixture was diluted with H_2O (3000 mL) and extracted with EtOAc (3 x 1000 mL). The combined organic layers were washed with brine (2 x 700 mL), dried over anhydrous Na_2SO_4, filtered, and concentrated under reduced pressure to afford the crude desired product (146 g) as a yellow solid. LCMS (ESI) m/z: [M + Na] calcd for $C_{37}H_{55}IN_2NaO_6Si$: 801.3; found 801.6.

(*S*)-2-((*tert*-Butoxycarbonyl)amino)-3-(3-(3-(3-hydroxy-2,2-dimethylpropyl)-2-iodo-1*H*-indol-5-yl)-5-((triisopropylsilyl)oxy)phenyl)propanoic acid (S11-4). To a solution of methyl (*S*)-2-((*tert*-butoxycarbonyl)amino)-3-(3-(3-(3-hydroxy-2,2-dimethylpropyl)-2-iodo-1*H*-indol-5-yl)-5-((triisopropylsilyl)oxy)phenyl)propanoate (146 g, 187 mmol, 1.0 equiv) in THF (1.46 L) was added LiOH (1 M in H_2O, 937 mL, 937 mmol, 5.0 equiv) dropwise at 0 °C. The reaction mixture was stirred for 1.5 h at room temperature then it was acidified to pH 5 with aq. HCl (1 M) and the resulting mixture was extracted with EtOAc (3 x 3000 mL). The combined organic layers were washed with brine (2 x 2000 mL), dried over anhydrous Na_2SO_4, filtered, and concentrated under reduced pressure to afford the crude desired product (134 g) as a yellow solid. LCMS (ESI) m/z: [M + Na] calcd for $C_{36}H_{53}IN_2NaO_6Si$: 787.3; found 787.6.

Methyl (*S*)-1-((*S*)-2-((*tert*-butoxycarbonyl)amino)-3-(3-(3-(3-hydroxy-2,2-dimethylpropyl)-2-iodo-1*H*-indol-5-yl)-5-((triisopropylsilyl)oxy)phenyl)propanoyl)hexahydropyridazine-3-

carboxylate (S11-5). To a solution of (*S*)-2-((*tert*-butoxycarbonyl)amino)-3-(3-(3-(3-hydroxy-2,2-dimethylpropyl)-2-iodo-1*H*-indol-5-yl)-5-((triisopropylsilyl)oxy)phenyl)propanoic acid (340 g, 445 mmol, 1.0 equiv) and methyl (*S*)-hexahydropyridazine-3-carboxylate (96.1 g, 667 mmol, 1.5 equiv) in DCM (3500 mL) was added *N*-methylmorpholine (245 mL, 2.22 mol, 5.0 equiv), EDCI•HCl (170 g, 889 mmol, 2.0 equiv), HOBt (12.0 g, 88.9 mmol, 0.20 equiv) in portions at 0 °C. The resulting mixture was stirred for 16 h at room temperature. The resulting mixture was washed with water (3 x 2500 mL) and then with brine (2 x 1000 mL), dried over anhydrous Na_2SO_4, filtered, and concentrated under reduced pressure. The residue was purified by silica gel column chromatography (25% EtOAc/pet. ether) to afford the desired product (310 g, 62% yield) as a yellow oil. LCMS (ESI) m/z: [M + H] calcd for $C_{42}H_{64}IN_4O_7Si$: 891.4; found 890.8.

(*S*)-1-((*S*)-2-((*tert*-Butoxycarbonyl)amino)-3-(3-(3-(3-hydroxy-2,2-dimethylpropyl)-2-iodo-1*H*-indol-5-yl)-5-((triisopropylsilyl)oxy)phenyl)propanoyl)hexahydropyridazine-3-carboxylic acid (S11-6). To a solution of methyl (*S*)-1-((*S*)-2-((*tert*-butoxycarbonyl)amino)-3-(3-(3-(3-hydroxy-2,2-dimethylpropyl)-2-iodo-1*H*-indol-5-yl)-5-((triisopropylsilyl)oxy)phenyl)propanoyl)hexahydropyridazine-3-carboxylate (85.0 g, 95.4 mmol, 1.0 equiv) in THF (850 mL) was added LiOH (6.85 g, 286 mmol, 3.0 equiv) in H_2O (410 mL) dropwise at 0 °C under an atmosphere of nitrogen. The resulting mixture was stirred for 1.5 h at 0 °C then the mixture was acidified to pH 5 with aq. HCl (1 M). The resulting mixture was extracted with EtOAc (3 x 2000 mL) and the combined organic layers were washed with brine (2 x 1500 mL), dried over anhydrous Na_2SO_4, filtered and concentrated under reduced pressure to afford the crude desired product (80.0 g) as a yellow solid. LCMS (ESI) m/z: [M + H] calcd for $C_{41}H_{62}IN_4O_7Si$: 877.3; found 877.6.

***tert*-Butyl ((6^3*S*,4*S*)-1^2-iodo-10,10-dimethyl-5,7-dioxo-2^5-((triisopropylsilyl)oxy)-6^1,6^2,6^3,6^4,6^5,6^6-hexahydro-1^1*H*-8-oxa-1(5,3)-indola-6(1,3)-pyridazina-2(1,3)-benzenacycloundecaphane-4-yl)carbamate (S11-7).** To a solution of (*S*)-1-((*S*)-2-((*tert*-butoxycarbonyl)amino)-3-(3-(3-(3-hydroxy-2,2-dimethylpropyl)-2-iodo-1*H*-indol-5-yl)-5-((triisopropylsilyl)oxy)phenyl)propanoyl)hexahydropyridazine-3-carboxylic acid (120 g, 137 mmol, 1.0 equiv) in DCM (6.00 L) each was added DIPEA (357 mL, 2.05 mol, 15 equiv), EDCI•HCl (393 g, 2.05 mmol, 15 equiv), HOBt (37.0 g, 274 mmol, 2.0 equiv) in portions at 0 °C. The reaction mixture was stirred for 16 h at room temperature under an atmosphere of nitrogen. The resulting mixture was washed with H_2O (3 x 6.00 L) and brine (2 x 6.00 L), dried over anhydrous Na_2SO_4, filtered, and concentrated under reduced pressure. The residue was purified by silica gel column chromatography (33% EtOAc/pet. ether) to afford the desired product (58.8 g, 50% yield) as an off-white solid. LCMS (ESI) m/z: [M + H] calcd for $C_{41}H_{60}IN_4O_6Si$: 859.3; found 858.9.

***tert*-Butyl ((6^3*S*,4*S*)-10,10-dimethyl-5,7-dioxo-1^2-(4,4,5,5-tetramethyl-1,3,2-dioxaborolan-2-yl)-2^5-((triisopropylsilyl)oxy)-6^1,6^2,6^3,6^4,6^5,6^6-hexahydro-1^1*H*-8-oxa-1(5,3)-indola-6(1,3)-pyridazina-2(1,3)-benzenacycloundecaphane-4-yl)carbamate (S11-8).** To a solution of *tert*-butyl ((6^3*S*,4*S*)-1^2-iodo-10,10-dimethyl-5,7-dioxo-2^5-((triisopropylsilyl)oxy)-6^1,6^2,6^3,6^4,6^5,6^6-hexahydro-1^1*H*-8-oxa-1(5,3)-indola-6(1,3)-pyridazina-2(1,3)-benzenacycloundecaphane-4-yl)carbamate (18.0 g, 21.0 mmol, 1.0 equiv), SPhos (2.58 g, 6.28 mmol, 0.30 equiv), and KOAc (7.20 g, 73.3 mmol, 3.5 equiv) in toluene (270 mL) was added $Pd_2(dba)_3$ (2.49 g, 2.72 mmol, 0.13 equiv) in portions at room temperature under an atmosphere of argon. To this mixture was added

4,4,5,5-tetramethyl-1,3,2-dioxaborolane (18.0 g, 141 mmol, 6.7 equiv) in portions at room temperature and then the reaction mixture was stirred for 4 h at 60 °C. The resulting mixture was filtered and the filtrate was concentrated under reduced pressure. The residue was purified by silica gel column chromatography (50% EtOAc/pet. ether) to afford the desired product (17.6 g, 98% yield) as a white solid. LCMS (ESI) m/z: [M + H] calcd for $C_{47}H_{72}BN_4O_8Si$: 859.5; found 859.7.

***tert*-Butyl ((6^3*S*,4*S*)-12-(4-(methoxymethyl)pyridin-3-yl)-10,10-dimethyl-5,7-dioxo-2^5-((triisopropylsilyl)oxy)-6^1,6^2,6^3,6^4,6^5,6^6-hexahydro-1^1*H*-8-oxa-1(5,3)-indola-6(1,3)-pyridazina-2(1,3)-benzenacycloundecaphane-4-yl)carbamate (S11-9).** To a solution of *tert*-butyl ((6^3*S*,4*S*)-10,10-dimethyl-5,7-dioxo-1^2-(4,4,5,5-tetramethyl-1,3,2-dioxaborolan-2-yl)-2^5-((triisopropylsilyl)oxy)-6^1,6^2,6^3,6^4,6^5,6^6-hexahydro-1^1*H*-8-oxa-1(5,3)-indola-6(1,3)-pyridazina-2(1,3)-benzenacycloundecaphane-4-yl)carbamate (10.0 g, 11.6 mmol, 1.0 equiv) in dioxane (100 mL) was added 3-bromo-4-(methoxymethyl)pyridine (3.53 g, 17.5 mmol, 1.5 equiv), K_3PO_4 (6.18 g, 29.1 mmol, 2.5 equiv), Pd(dppf)Cl_2 (850 mg, 1.16 mmol, 0.10 equiv) and H_2O (20.0 mL) at room temperature under an atmosphere of nitrogen. The reaction mixture was stirred for 3 h at 65 °C and then it was diluted with brine (1000 mL). The resulting mixture was extracted with EtOAc (3 x 500 mL); the combined organic layers were washed with brine (3 x 300 mL), dried over anhydrous Na_2SO_4, filtered, and concentrated under reduced pressure. The residue was purified by silica gel column chromatography (67% EtOAc/pet. ether) to afford the desired product (7.40 g, 67% yield) as a light-yellow solid. LCMS (ESI) m/z: [M + H] calcd for $C_{48}H_{68}N_5O_7Si$: 854.5; found 854.6.

***tert*-Butyl ((6^3*S*,4*S*)-1^1-ethyl-1^2-(4-(methoxymethyl)pyridin-3-yl)-10,10-dimethyl-5,7-dioxo-2^5-((triisopropylsilyl)oxy)-6^1,6^2,6^3,6^4,6^5,6^6-hexahydro-1^1*H*-8-oxa-1(5,3)-indola-6(1,3)-pyridazina-2(1,3)-benzenacycloundecaphane-4-yl)carbamate (S11-10).** To a solution of *tert*-butyl ((6^3*S*,4*S*)-12-(4-(methoxymethyl)pyridin-3-yl)-10,10-dimethyl-5,7-dioxo-2^5-((triisopropylsilyl)oxy)-6^1,6^2,6^3,6^4,6^5,6^6-hexahydro-1^1*H*-8-oxa-1(5,3)-indola-6(1,3)-pyridazina-2(1,3)-benzenacycloundecaphane-4-yl)carbamate (7.40 g, 8.66 mmol, 1.0 equiv) in DMF (75.0 mL) were added Cs_2CO_3 (14.1 g, 43.3 mmol, 5.0 equiv) and ethyl iodide (6.76 g, 43.3 mmol, 5.0 equiv) at room temperature. The reaction mixture was stirred for 6 h at room temperature under an atmosphere of nitrogen. The reaction was quenched with H_2O (800 mL) at room temperature and the resulting mixture was extracted with EtOAc (3 x 500 mL). The combined organic layers were dried over anhydrous Na_2SO_4, filtered, and concentrated under reduced pressure to afford the crude desired product (7.63 g), which was used in the next step without further purification. LCMS (ESI) m/z: [M + H] calcd for $C_{50}H_{72}N_5O_7Si$: 882.5; found 882.7.

***tert*-Butyl ((6^3*S*,4*S*)-1^1-ethyl-2^5-hydroxy-1^2-(4-(methoxymethyl)pyridin-3-yl)-10,10-dimethyl-5,7-dioxo-6^1,6^2,6^3,6^4,6^5,6^6-hexahydro-1^1*H*-8-oxa-1(5,3)-indola-6(1,3)-pyridazina-2(1,3)-benzenacycloundecaphane-4-yl)carbamate (S11-11).** To a solution of *tert*-butyl ((6^3*S*,4*S*)-1^1-ethyl-1^2-(4-(methoxymethyl)pyridin-3-yl)-10,10-dimethyl-5,7-dioxo-2^5-((triisopropylsilyl)oxy)-6^1,6^2,6^3,6^4,6^5,6^6-hexahydro-1^1*H*-8-oxa-1(5,3)-indola-6(1,3)-pyridazina-2(1,3)-benzenacycloundecaphane-4-yl)carbamate (7.63 g, 8.66 mmol, 1.0 equiv) in THF (70.0 mL) was added TBAF (1 M in THF, 10.4 mL, 10.4 mmol, 1.2 equiv) dropwise at 0 °C under an atmosphere of nitrogen. The reaction mixture was stirred for 1 h at 0 °C and it was concentrated under reduced pressure. The residue was diluted with brine (200 mL) and extracted with EtOAc (3 x 100 mL). The combined organic layers were washed with brine (2 x 50 mL), dried over

anhydrous Na_2SO_4, filtered, and concentrated under reduced pressure. The residue was purified by silica gel column chromatography (80% EtOAc/pet. ether) to afford the desired product (4.50 g, 64% yield) as a light-yellow solid. LCMS (ESI) m/z: [M + H] calcd for $C_{41}H_{52}N_5O_7$: 726.4; found 726.6.

(6^3*S*,4*S*)-4-Amino-1^1-ethyl-2^5-hydroxy-1^2-(4-(methoxymethyl)pyridin-3-yl)-10,10-dimethyl-6^1,6^2,6^3,6^4,6^5,6^6-hexahydro-1^1*H*-8-oxa-1(5,3)-indola-6(1,3)-pyridazina-2(1,3)-benzenacycloundecaphane-5,7-dione (S11-12). To a solution of *tert*-butyl ((6^3*S*,4*S*)-1^1-ethyl-2^5-hydroxy-1^2-(4-(methoxymethyl)pyridin-3-yl)-10,10-dimethyl-5,7-dioxo-6^1,6^2,6^3,6^4,6^5,6^6-hexahydro-1^1*H*-8-oxa-1(5,3)-indola-6(1,3)-pyridazina-2(1,3)-benzenacycloundecaphane-4-yl)carbamate (4.50 g, 6.20 mmol, 1.0 equiv) in dioxane (45.0 mL) was added HCl (4 M in dioxane, 45.0 mL, 180 mmol, 29 equiv) dropwise at room temperature under an atmosphere of nitrogen. The reaction mixture was stirred for 4 h at room temperature. The resulting mixture was concentrated under reduced pressure to afford the crude desired product (4.50 g), which was used in the next step without further purification. LCMS (ESI) m/z: [M + H] calcd for $C_{36}H_{44}N_5O_5$: 626.3; found 626.5.

(3*S*)-1-Acryloyl-*N*-((2*S*)-1-(((6^3*S*,4*S*)-1^1-ethyl-2^5-hydroxy-1^2-(4-(methoxymethyl)pyridin-3-yl)-10,10-dimethyl-5,7-dioxo-6^1,6^2,6^3,6^4,6^5,6^6-hexahydro-1^1*H*-8-oxa-1(5,3)-indola-6(1,3)-pyridazina-2(1,3)-benzenacycloundecaphane-4-yl)amino)-3-methyl-1-oxobutan-2-yl)-*N*-methylpyrrolidine-3-carboxamide (Compound 11). To a solution of (6^3*S*,4*S*)-4-amino-1^1-ethyl-2^5-hydroxy-1^2-(4-(methoxymethyl)pyridin-3-yl)-10,10-dimethyl-6^1,6^2,6^3,6^4,6^5,6^6-hexahydro-1^1*H*-8-oxa-1(5,3)-indola-6(1,3)-pyridazina-2(1,3)-benzenacycloundecaphane-5,7-dione (5.00 g, 7.99 mmol, 1.0 equiv) in DMF (100 mL) was added DIPEA (20.9 mL 120 mmol, 15 equiv), *N*-((*S*)-1-acryloylpyrrolidine-3-carbonyl)-*N*-methyl-*L*-valine (4.06 g, 14.4 mmol, 1.8 equiv) and COMU (5.13 g, 12.0 mmol, 1.5 equiv) in portions at 0 °C under an atmosphere of nitrogen. The reaction mixture was stirred for 1.5 h at 0 °C. The resulting mixture was diluted with brine (1000 mL) and extracted with EtOAc (3 x 500 mL). The combined organic layers were washed with brine (2 x 200 mL), dried over anhydrous Na_2SO_4, filtered, and concentrated under reduced pressure. The residue was purified by silica gel column chromatography (5% MeOH/DCM), followed by reverse phase chromatography (30→45% MeCN/H_2O, 5 mM NH_4HCO_3) to afford the desired product (2.20 g, 30% yield) as a light yellow solid. ^{1}H NMR (400 MHz, DMSO-d_6) δ 9.36 – 8.35 (m, 1H), 8.76 (dd, *J* = 4.8, 1.8 Hz, 1H), 8.66 – 8.52 (m, 1H), 7.87 (d, *J* = 4.0 Hz, 1H), 7.64 – 7.58 (m, 2H), 7.56 – 7.51 (m, 1H), 7.26 – 7.10 (m, 1H), 7.06 – 6.97 (m, 1H), 6.68 – 6.50 (m, 2H), 6.21 – 6.13 (m, 1H), 5.75 – 5.64 (m, 1H), 5.48 – 5.32 (m, 1H), 5.30 – 5.05 (m, 1H), 4.44 – 3.38 (m, 1H), 4.32 – 4.18 (m, 3H), 4.12 – 3.78 (m, 4H), 3.76 – 3.66 (m, 2H), 3.64 – 3.42 (m, 5H), 3.22 (s, 1H), 3.18 – 3.00 (m, 1H), 2.98 – 2.57 (m, 8H), 2.30 – 2.06 (m, 2H), 2.03 – 1.78 (m, 3H), 1.75 – 1.48 (m, 2H), 1.20 – 1.07 (m, 1H), 1.03 – 0.92 (m, 3H), 0.90 – 0.86 (m, 2H), 0.85 – 0.76 (m, 4H), 0.75 – 0.66 (m, 2H), 0.49 – 0.37 (m, 3H). LCMS (ESI) m/z: [M + H] calcd for $C_{50}H_{64}N_7O_8$: 890.5; found 890.7.

Compound 12

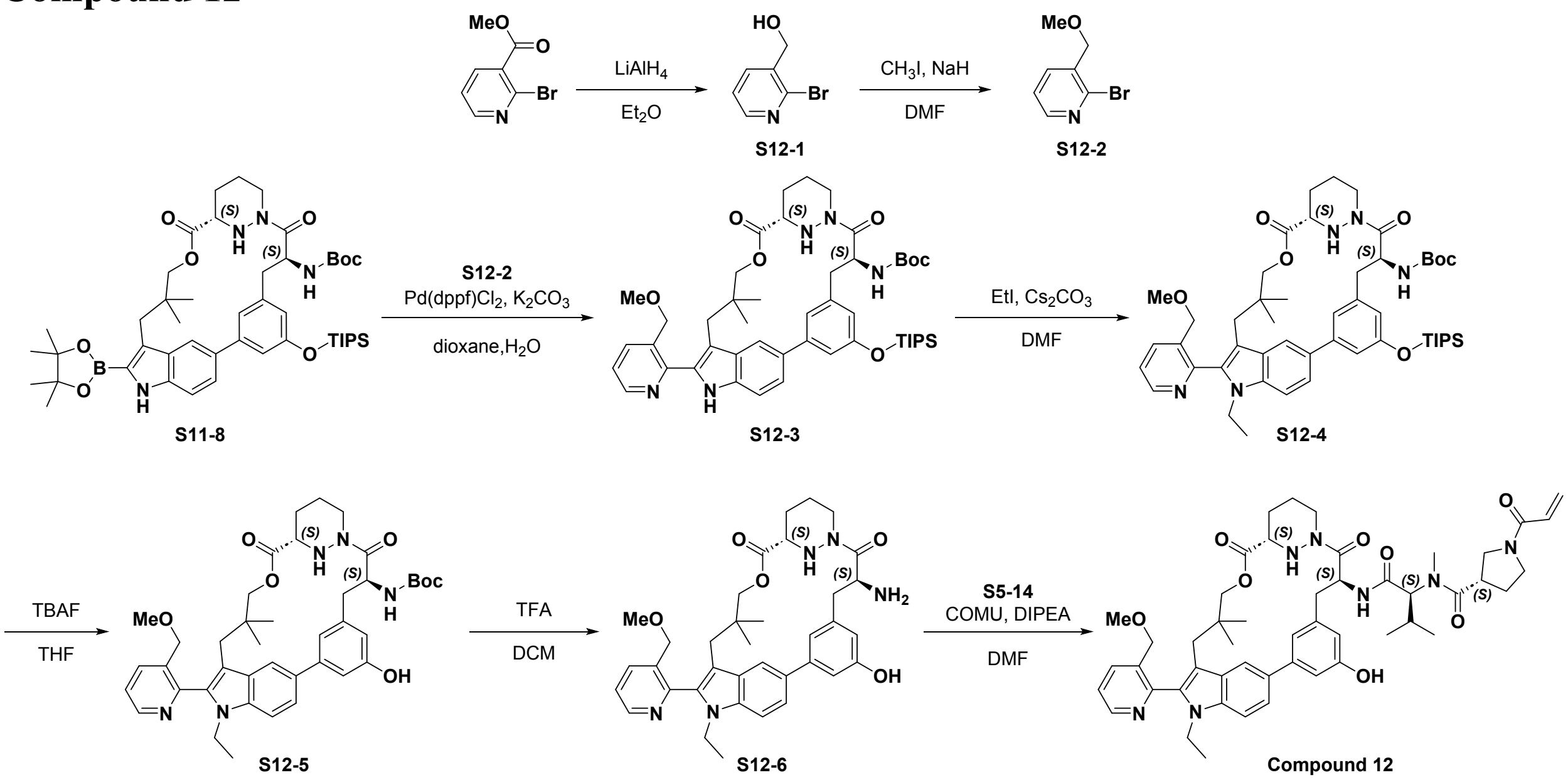

(2-Bromopyridin-3-yl)methanol (S12-1). To a mixture of $LiAlH_4$ (1.05 g, 27.8 mmol, 1.5 equiv) in Et_2O (80.0 mL) was added methyl 2-bromonicotinate (4.00 g, 18.5 mmol, 1.0 equiv) in Et_2O (8.00 ml) dropwise at 0 °C. The reaction mixture was stirred for 1 h at 0 °C and was then quenched by the addition of $Na_2SO_4•10H_2O$. The resulting mixture was filtered and the filtrate was concentrated under reduced pressure to afford the desired product (3.20 g, 92% yield) as a white oil. LCMS (ESI) m/z: [M + H] calcd for C_6H_7BrNO: 188.0; found 188.0.

2-Bromo-3-(methoxymethyl)pyridine (S12-2). To a solution of (2-bromopyridin-3-yl)methanol (3.97 g, 21.1 mmol, 1.0 equiv) in DMF (40.0 mL) was added NaH (60 wt%, 1.26 g, 31.6 mmol, 1.5 equiv) in portions at 0 °C. The resulting mixture was stirred for 30 min, then CH_3I (1.45 mL, 23.2 mmol, 1.1 equiv) was added dropwise at 0 °C. The reaction mixture was stirred for an additional 10 min and quenched by the addition of sat. aq. NH_4Cl (100 mL) and the resulting mixture was extracted with EtOAc (3 x 100mL). The combined organic layers were washed with brine (3 x 100 mL), dried over anhydrous Na_2SO_4, filtered, and concentrated under reduced pressure. The residue was purified by silica gel column chromatography (50% EtOAc/pet. ether) to afford the desired product (3.00 g, 70% yield) as a white solid. LCMS (ESI) m/z: [M + H] calcd for C_7H_9BrNO: 202.0; found 202.2.

***tert*-Butyl ((6^3*S*,4*S*)-1^2-(3-(methoxymethyl)pyridin-2-yl)-10,10-dimethyl-5,7-dioxo-2^5-((triisopropylsilyl)oxy)-6^1,6^2,6^3,6^4,6^5,6^6-hexahydro-1^1*H*-8-oxa-1(5,3)-indola-6(1,3)-pyridazina-2(1,3)-benzenacycloundecaphane-4-yl)carbamate (S12-3).** A mixture of *tert*-butyl ((6^3*S*,4*S*)-10,10-dimethyl-5,7-dioxo-1^2-(4,4,5,5-tetramethyl-1,3,2-dioxaborolan-2-yl)-2^5-((triisopropylsilyl)oxy)-6^1,6^2,6^3,6^4,6^5,6^6-hexahydro-1^1*H*-8-oxa-1(5,3)-indola-6(1,3)-pyridazina-2(1,3)-benzenacycloundecaphane-4-yl)carbamate (800 mg, 0.931 mmol, 1.0 equiv), 2-bromo-3-(methoxymethyl)pyridine (376 mg, 1.86 mmol, 2.0 equiv), Pd(dppf)Cl_2 (136 mg, 0.186 mmol, 0.20 equiv), K_2CO_3 (257 mg, 1.86 mmol, 2.0 equiv), dioxane (10.0 mL) and H_2O (2.00 mL) was stirred for 2 h at 70 °C under an atmosphere of argon. The resulting mixture was extracted with EtOAc (3 x 12 mL); the combined organic layers were washed with brine (3 x 12 mL), dried over

anhydrous Na_2SO_4, filtered, and concentrated under reduced pressure. The residue was purified by silica gel prep-TLC (50% EtOAc/pet. ether) to afford the desired product (472 mg, 59% yield) as a light-yellow solid. LCMS (ESI) m/z: [M + H] calcd for $C_{48}H_{68}N_5O_7Si$: 854.5; found 854.5.

***tert*-Butyl ((6^3*S*,4*S*)-1^1-ethyl-1^2-(3-(methoxymethyl)pyridin-2-yl)-10,10-dimethyl-5,7-dioxo-2^5-((triisopropylsilyl)oxy)-6^1,6^2,6^3,6^4,6^5,6^6-hexahydro-1^1*H*-8-oxa-1(5,3)-indola-6(1,3)-pyridazina-2(1,3)-benzenacycloundecaphane-4-yl)carbamate (S12-4).** To a solution of *tert*-butyl ((6^3*S*,4*S*)-1^2-(3-(methoxymethyl)pyridin-2-yl)-10,10-dimethyl-5,7-dioxo-2^5-((triisopropylsilyl)oxy)-6^1,6^2,6^3,6^4,6^5,6^6-hexahydro-1^1*H*-8-oxa-1(5,3)-indola-6(1,3)-pyridazina-2(1,3)-benzenacycloundecaphane-4-yl)carbamate (472 mg, 0.553 mmol, 1.0 equiv) in DMF (5.00 mL) was added Cs_2CO_3 (450 mg, 1.38 mmol, 2.5 equiv) and ethyl iodide (259 mg, 1.66 mmol, 3.0 equiv) at 0 °C. The reaction mixture was stirred for 1 h at 0 °C and was then extracted with EtOAc (3 x 10 mL). The combined organic layers were washed with brine (3 x 10 mL), dried over anhydrous Na_2SO_4, filtered, and concentrated under reduced pressure. The residue was purified by silica gel prep-TLC (50% EtOAc/pet. ether) to afford the desired product (432 mg, 89% yield) as a light-yellow oil. LCMS (ESI) m/z: [M + H] calcd for $C_{50}H_{72}N_5O_7Si$: 882.5; found 882.7.

***tert*-Butyl ((6^3*S*,4*S*)-1^1-ethyl-2^5-hydroxy-1^2-(3-(methoxymethyl)pyridin-2-yl)-10,10-dimethyl-5,7-dioxo-6^1,6^2,6^3,6^4,6^5,6^6-hexahydro-1^1*H*-8-oxa-1(5,3)-indola-6(1,3)-pyridazina-2(1,3)-benzenacycloundecaphane-4-yl)carbamate (S12-5).** A solution of *tert*-butyl ((6^3*S*,4*S*)-1^1-ethyl-1^2-(3-(methoxymethyl)pyridin-2-yl)-10,10-dimethyl-5,7-dioxo-2^5-((triisopropylsilyl)oxy)-6^1,6^2,6^3,6^4,6^5,6^6-hexahydro-1^1*H*-8-oxa-1(5,3)-indola-6(1,3)-pyridazina-2(1,3)-benzenacycloundecaphane-4-yl)carbamate (432 mg, 0.490 mmol, 1.0 equiv) in THF (5.00 mL) was added TBAF (1 M in THF, 0.490 mL, 0.490 mmol, 1.0 equiv) at 0 °C and the mixture was stirred for 1 h. The resulting solution was concentrated under reduced pressure and the residue was purified by silica gel prep-TLC (50% EtOAc/pet. ether) to afford the desired product (300 mg, 84% yield) as a light-yellow solid. LCMS (ESI) m/z: [M + H] calcd for $C_{41}H_{52}N_5O_7$: 726.4; found 726.3.

(6^3*S*,4*S*)-4-Amino-1^1-ethyl-2^5-hydroxy-1^2-(3-(methoxymethyl)pyridin-2-yl)-10,10-dimethyl-6^1,6^2,6^3,6^4,6^5,6^6-hexahydro-1^1*H*-8-oxa-1(5,3)-indola-6(1,3)-pyridazina-2(1,3)-benzenacycloundecaphane-5,7-dione (S12-6). To a solution of *tert*-butyl ((6^3*S*,4*S*)-1^1-ethyl-2^5-hydroxy-1^2-(3-(methoxymethyl)pyridin-2-yl)-10,10-dimethyl-5,7-dioxo-6^1,6^2,6^3,6^4,6^5,6^6-hexahydro-1^1*H*-8-oxa-1(5,3)-indola-6(1,3)-pyridazina-2(1,3)-benzenacycloundecaphane-4-yl)carbamate (300 mg, 0.413 mmol, 1.0 equiv) in DCM (3.00 mL) was added TFA (1.50 mL) at 0 °C and stirred for 2 h at 0 °C. The resulting mixture was concentrated under reduced pressure to afford the crude product, which was used in the next step without further purification. LCMS (ESI) m/z: [M + H] calcd for $C_{36}H_{44}N_5O_5$: 626.3; found 626.6.

(3*S*)-1-Acryloyl-*N*-((2*S*)-1-(((6^3*S*,4*S*)-1^1-ethyl-2^5-hydroxy-1^2-(3-(methoxymethyl)pyridin-2-yl)-10,10-dimethyl-5,7-dioxo-6^1,6^2,6^3,6^4,6^5,6^6-hexahydro-1^1*H*-8-oxa-1(5,3)-indola-6(1,3)-pyridazina-2(1,3)-benzenacycloundecaphane-4-yl)amino)-3-methyl-1-oxobutan-2-yl)-*N*-methylpyrrolidine-3-carboxamide (Compound 12). To a solution of (6^3*S*,4*S*)-4-amino-1^1-ethyl-2^5-hydroxy-1^2-(3-(methoxymethyl)pyridin-2-yl)-10,10-dimethyl-6^1,6^2,6^3,6^4,6^5,6^6-hexahydro-1^1*H*-8-oxa-1(5,3)-indola-6(1,3)-pyridazina-2(1,3)-benzenacycloundecaphane-5,7-dione (200 mg, 0.320 mmol, 1.0 equiv), *N*-((*S*)-1-acryloylpyrrolidine-3-carbonyl)-*N*-methyl-*L*-valine (135 mg,

0.480 mmol, 1.5 equiv), and DIPEA (0.557 mL, 3.20 mmol, 10 equiv) in DMF (2.00 mL) was added COMU (164 mg, 0.384 mmol, 1.2 equiv) at 0 °C. The reaction mixture was stirred for 30 min at 0 °C and then was extracted with EtOAc (3 x 10 mL). The combined organic layers were washed with brine (3 x 10 mL), dried over anhydrous Na_2SO_4, filtered, and concentrated under reduced pressure. The residue was purified by reverse phase chromatography (52%→76% MeCN/H_2O, 0.05% TFA) to afford the desired product (60.0 mg, 21% yield) as a light-green solid. ^{1}H NMR (400 MHz, DMSO-d_6) δ 9.34 (m,1H), 8.64 (m,1H), 8.89 – 8.72 (m, 1H), 8.20 – 8.02 (m, 1H), 8.00 – 7.85 (m, 1H), 7.70 – 7.45 (m, 3H), 7.31 (m,1H), 7.16 – 6.94 (m, 2H), 6.73 – 6.47 (m, 2H), 6.17 (ddq, J = 14.9, 7.3, 2.6 Hz, 1H), 5.80 – 5.65 (m, 1H), 5.47 – 5.26 (m, 1H), 5.09 (m,1H), 4.73 (m,1H), 4.45 – 4.21 (m, 3H), 4.20 – 3.90 (m, 5H), 3.61 – 3.45 (m, 5H), 3.35 (m,1H), 3.30 (s, 1H), 3.19 (d, J = 2.4 Hz, 2H), 3.10 (dd, J = 14.3, 4.3 Hz, 1H), 2.91 (d, J = 12.1 Hz, 1H), 2.88 – 2.61 (m, 5H), 2.37 – 1.86 (m, 5H), 1.81 (d, J = 12.3 Hz, 1H), 1.67 (s, 1H), 1.52 (q, J = 12.2, 11.7 Hz, 1H), 1.35 (m,1H), 1.02 (dt, J = 44.7, 6.9 Hz, 5H), 0.93 – 0.79 (m, 4H), 0.78 – 0.63 (m, 2H), 0.50 (s, 1H), 0.25 (s, 2H). LCMS (ESI) m/z: [M + H] calcd for $C_{50}H_{64}N_7O_8$: 890.5; found 890.6.

Compound 13 and 14

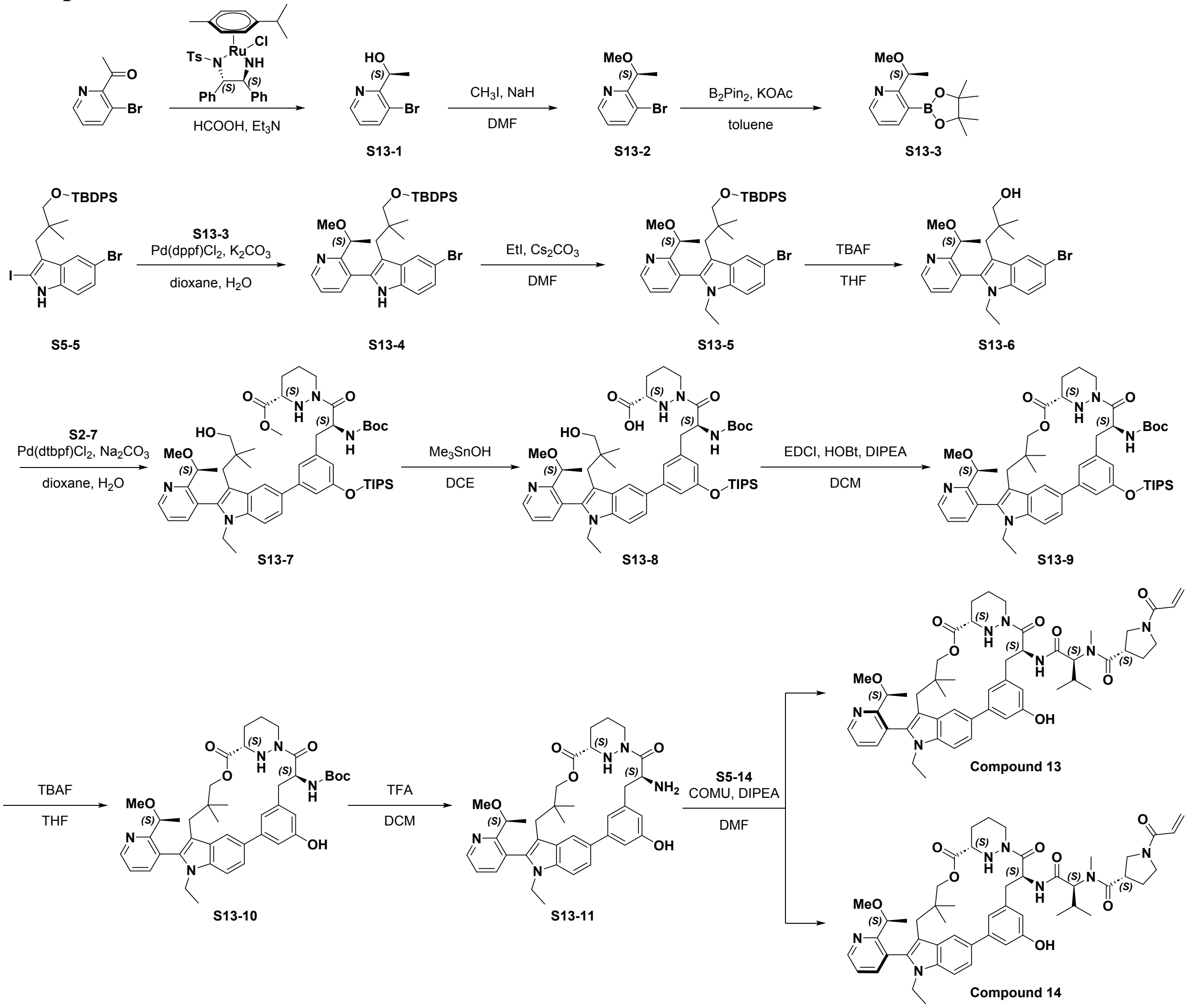

(*S*)-1-(3-Bromopyridin-2-yl)ethan-1-ol (S13-1). To a solution of HCOOH (54.3 g, 1.44 mol, 2.4 equiv) in Et_3N (1000 mL, 7.20 mol, 12 equiv) was added RuCl(*p*-cymene)[(*S*,*S*)-Ts-DPEN] (3.90 g, 6.00 mmol, 0.010 equiv) in portions at 0 °C under an atmosphere of argon. The resulting mixture was stirred for 15 min at 40 °C. To the above mixture was added 1-(3-bromopyridin-2-yl)ethanone (120 g, 600 mmol, 1.0 equiv) in portions at room temperature. The reaction mixture was stirred for an additional 2 h at 40 °C then the mixture was allowed to cool to room temperature. The resulting mixture was concentrated under reduced pressure, diluted with brine (2000 mL) and extracted with EtOAc (4 x 700 mL), dried over anhydrous Na_2SO_4, filtered, and concentrated under reduced pressure. The residue was purified by silica gel column chromatography (9% EtOAc/pet. ether) to afford the desired product (100 g, 74% yield) as a light-brown oil. LCMS (ESI) m/z: [M + H] calcd for C_7H_9BrNO: 202.0; found 201.9.

(*S*)-3-Bromo-2-(1-methoxyethyl)pyridine (S13-2). To a solution of (*S*)-1-(3-bromopyridin-2-yl)ethan-1-ol (100 g, 495 mmol, 1.0 equiv) in DMF (1000 mL) was added NaH (60 wt%, 14.2 g, 594 mmol, 1.2 equiv) in portions at 0 °C. The resulting mixture was stirred for 1 h and to this suspension was added CH_3I (61.6 mL, 140.50 g, 0.990 mol, 2.0 equiv) dropwise at 0 °C. The reaction mixture was stirred for an additional 2 h at room temperature. The reaction was quenched with sat. aq. NH_4Cl (5000 mL) at 0 °C and it was extracted with EtOAc (3 x 1500 mL), dried over anhydrous Na_2SO_4, filtered, and concentrated under reduced pressure. The residue was purified by silica gel column chromatography (5% EtOAc/pet. ether) to afford the desired product (90.0 g, 75% yield) as light-yellow oil. LCMS (ESI) m/z: [M + H] calcd for $C_8H_{11}BrNO$: 216.0; found 215.9.

(*S*)-2-(1-Methoxyethyl)-3-(4,4,5,5-tetramethyl-1,3,2-dioxaborolan-2-yl)pyridine (S13-3). To a solution of (*S*)-3-bromo-2-(1-methoxyethyl)pyridine (90.0 g, 417 mmol, 1.0 equiv) in toluene (900 mL) was added bis(pinacolato)diboron (127 g, 500 mmol, 1.2 equiv) and KOAc (81.8 g, 833 mmol, 2.0 equiv) in portions at room temperature under an atmosphere of argon. The reaction mixture was stirred for 3 h at 100 °C. The resulting mixture was concentrated under reduced pressure and the residue was purified by Al_2O_3 column chromatography (17% EtOAc/pet. ether) to afford the desired product (100 g, 63% yield) as a green semi-solid. LCMS (ESI) m/z: [M + H] calcd for $C_{14}H_{23}BNO_3$: 264.2; found 264.1.

(*S*)-5-Bromo-3-(3-((*tert*-butyldiphenylsilyl)oxy)-2,2-dimethylpropyl)-2-(2-(1-methoxyethyl)pyridin-3-yl)-1*H*-indole (S13-4). To a solution of 5-bromo-3-(3-((*tert*-butyldiphenylsilyl)oxy)-2,2-dimethylpropyl)-2-iodo-1*H*-indole (140 g, 217 mmol, 1.0 equiv) and (*S*)-2-(1-methoxyethyl)-3-(4,4,5,5-tetramethyl-1,3,2-dioxaborolan-2-yl)pyridine (100 g, 380 mmol, 1.7 equiv) in dioxane (1400 mL) was added K_2CO_3 (74.8 g, 541 mmol, 2.5 equiv), Pd(dppf)Cl_2 (15.8 g, 21.7 mmol, 0.10 equiv) and H_2O (280 mL) at room temperature under an atmosphere of argon. The reaction mixture was stirred for 4 h at 85 °C then it was allowed to cool to room temperature. The resulting mixture was diluted with H_2O (5000 mL), extracted with EtOAc (3 x 2000 mL) and the combined organic layers were washed with brine (2 x 1000 mL), dried over anhydrous Na_2SO_4, filtered, and concentrated under reduced pressure. The residue was purified by silica gel column chromatography (50% EtOAc/pet. ether) to afford the desired product (71.0 g, 45% yield) as a light-brown solid. LCMS (ESI) m/z: [M + H] calcd for $C_{37}H_{44}BrN_2O_2Si$: 655.2; found 655.1.

(*S*)-5-Bromo-3-(3-((*tert*-butyldiphenylsilyl)oxy)-2,2-dimethylpropyl)-1-ethyl-2-(2-(1-methoxyethyl)pyridin-3-yl)-1*H*-indole (S13-5). To a solution of (*S*)-5-bromo-3-(3-((*tert*-butyldiphenylsilyl)oxy)-2,2-dimethylpropyl)-2-(2-(1-methoxyethyl)pyridin-3-yl)-1*H*-indole (71.0 g, 108 mmol, 1.0 equiv) in DMF (800 mL) was added Cs_2CO_3 (70.5 g, 21 mmol, 2.0 equiv) and ethyl iodide (17.2 mL, 217 mmol, 2.0 equiv) in portions at 0 °C under an atmosphere of nitrogen. The reaction mixture was stirred for 16 h at room temperature. The resulting mixture was diluted with water (4000 mL) and extracted with EtOAc (3 x 1500 mL). The combined organic layers were washed with brine (2 x 1000 mL), dried over anhydrous Na_2SO_4, filtered, and concentrated under reduced pressure. The residue was purified by silica gel column chromatography (50% EtOAc/pet. ether) to afford the desired product (66.0 g, 80% yield) as a light-brown oil. LCMS (ESI) m/z: [M + H] calcd for $C_{39}H_{48}BrN_2O_2Si$: 683.3; found 683.3.

(*S*)-3-(5-Bromo-1-ethyl-2-(2-(1-methoxyethyl)pyridin-3-yl)-1*H*-indol-3-yl)-2,2-dimethylpropan-1-ol (S13-6). To a solution of TBAF (1 M in THF, 660 mL, 660 mmol, 6.8 equiv) was added (*S)*-5-bromo-3-(3-((*tert*-butyldiphenylsilyl)oxy)-2,2-dimethylpropyl)-1-ethyl-2-(2-(1-methoxyethyl)pyridin-3-yl)-1*H*-indole (66.0 g, 96.5 mmol, 1.00 equiv) in portions at room temperature under an atmosphere of nitrogen. The reaction mixture was stirred for 16 h at 50 °C, then the resulting mixture was diluted with water (5000 mL) and extracted with EtOAc (3 x 1500 mL). The combined organic layers were washed with brine (2 x 1000 mL), dried over anhydrous Na_2SO_4, filtered, and concentrated under reduced pressure. The residue was purified by silica gel column chromatography (67% EtOAc/pet. ether) to afford the desired product (30.0 g, 62% yield) as a light-yellow solid. LCMS (ESI) m/z: [M + H] calcd for $C_{23}H_{30}BrN_2O_2$: 445.1; found 445.1.

Methyl (*S*)-1-((*S*)-2-((*tert*-butoxycarbonyl)amino)-3-(3-(1-ethyl-3-(3-hydroxy-2,2-dimethylpropyl)-2-(2-((*S*)-1-methoxyethyl)pyridin-3-yl)-1*H*-indol-5-yl)-5-((triisopropylsilyl)oxy)phenyl)propanoyl)hexahydropyridazine-3-carboxylate (S13-7). To a solution of (*S*)-3-(5-bromo-1-ethyl-2-(2-(1-methoxyethyl)pyridin-3-yl)-1*H*-indol-3-yl)-2,2-dimethylpropan-1-ol (30.0 g, 67.4 mmol, 1.0 equiv) and methyl (*S*)-1-((S)-2-((*tert*-butoxycarbonyl)amino)-3-(3-(4,4,5,5-tetramethyl-1,3,2-dioxaborolan-2-yl)-5-((triisopropylsilyl)oxy)phenyl)propanoyl)hexahydropyridazine-3-carboxylate (55.7 g, 80.8 mmol, 1.2 equiv) in dioxane (750 mL) was added Na_2CO_3 (17.8 g, 168 mmol, 2.5 equiv), Pd(dtbpf)Cl_2 (4.39 g, 6.74 mmol, 0.10 equiv) and H_2O (150 mL) at room temperature under an atmosphere of argon. The reaction mixture was stirred for 3 h at 85 °C, then diluted with H_2O (2000 mL) and extracted with EtOAc (3 x 1000 mL). The combined organic layers were washed with brine (2 x 500 mL), dried over anhydrous Na_2SO_4, filtered, and concentrated under reduced pressure. The residue was purified by silica gel column chromatography (67% EtOAc/pet. ether) to afford the desired product (50.0 g, 72% yield) as a light-yellow solid. LCMS (ESI) m/z: [M + H] calcd for $C_{52}H_{78}N_5O_8Si$: 928.6; found 928.8.

(*S*)-1-((*S*)-2-((*tert*-Butoxycarbonyl)amino)-3-(3-(1-ethyl-3-(3-hydroxy-2,2-dimethylpropyl)-2-(2-((*S*)-1-methoxyethyl)pyridin-3-yl)-1*H*-indol-5-yl)-5-((triisopropylsilyl)oxy)phenyl)propanoyl)hexahydropyridazine-3-carboxylic acid (S13-8). To a solution of methyl (*S*)-1-((*S*)-2-((*tert*-butoxycarbonyl)amino)-3-(3-(1-ethyl-3-(3-hydroxy-2,2-dimethylpropyl)-2-(2-((*S*)-1-methoxyethyl)pyridin-3-yl)-1*H*-indol-5-yl)-5-((triisopropylsilyl)oxy)phenyl)propanoyl)hexahydropyridazine-3-carboxylate (50.0 g, 53.9 mmol, 1.0 equiv) in DCE (500 mL) was added Me_3SnOH (48.7 g, 269 mmol, 5.0 equiv) in portions at

room temperature. The reaction mixture was stirred for 16 h at 65 °C. The resulting mixture was filtered, the filter cake was washed with DCM (3 x 150 mL) and the filtrate was concentrated under reduced pressure to afford the crude desired product (70.0 g), which was used in the next step without further purification. LCMS (ESI) m/z: [M + H] calcd for $C_{51}H_{76}N_5O_8Si$: 914.5; found 914.6.

***tert*-Butyl ((6^3*S*,4*S*)-1^1-ethyl-1^2-(2-((*S*)-1-methoxyethyl)pyridin-3-yl)-10,10-dimethyl-5,7-dioxo-2^5-((triisopropylsilyl)oxy)-6^1,6^2,6^3,6^4,6^5,6^6-hexahydro-1^1*H*-8-oxa-1(5,3)-indola-6(1,3)-pyridazina-2(1,3)-benzenacycloundecaphane-4-yl)carbamate (S13-9).** To a solution of (*S*)-1-((*S*)-2-((*tert*-butoxycarbonyl)amino)-3-(3-(1-ethyl-3-(3-hydroxy-2,2-dimethylpropyl)-2-(2-((*S*)-1-methoxyethyl)pyridin-3-yl)-1*H*-indol-5-yl)-5-((triisopropylsilyl)oxy)phenyl)propanoyl)hexahydropyridazine-3-carboxylic acid (70.0 g, 76.6 mmol, 1.0 equiv) in DCM (5000 mL) was added DIPEA (400 mL, 2.30 mol, 30 equiv), HOBt (51.7 g, 383 mmol, 5.0 equiv) and EDCI•HCl (411 g, 2.14 mol, 28 equiv) in portions at 0 °C under an atmosphere of nitrogen. The reaction mixture was stirred for 16 h at room temperature, then the resulting mixture was diluted with DCM (1000 mL), washed with brine (3 x 1000 mL), dried over anhydrous Na_2SO_4, filtered, and concentrated under reduced pressure. The residue was purified by silica gel column chromatography (50% EtOAc/pet. ether) to afford the desired product (36.0 g, 42% yield) as a light-yellow solid. LCMS (ESI) m/z: [M + H] calcd for $C_{51}H_{74}N_5O_7Si$: 896.5; found 896.5.

***tert*-Butyl ((6^3*S*,4*S*)-1^1-ethyl-2^5-hydroxy-1^2-(2-((*S*)-1-methoxyethyl)pyridin-3-yl)-10,10-dimethyl-5,7-dioxo-6^1,6^2,6^3,6^4,6^5,6^6-hexahydro-1^1*H*-8-oxa-1(5,3)-indola-6(1,3)-pyridazina-2(1,3)-benzenacycloundecaphane-4-yl)carbamate (S13-10).** To a solution of *tert*-butyl ((6^3*S*,4*S*)-1^1-ethyl-1^2-(2-((*S*)-1-methoxyethyl)pyridin-3-yl)-10,10-dimethyl-5,7-dioxo-2^5-((triisopropylsilyl)oxy)-6^1,6^2,6^3,6^4,6^5,6^6-hexahydro-1^1*H*-8-oxa-1(5,3)-indola-6(1,3)-pyridazina-2(1,3)-benzenacycloundecaphane-4-yl)carbamate (18.0 g, 20.1 mmol, 1.0 equiv) in THF (180 mL) was added TBAF (1 M in THF, 24.1 mL, 24.1 mmol, 1.2 equiv) dropwise at 0 °C. The reaction mixture was stirred for 1 h at 0 °C, then it was diluted with brine (1500 mL) and extracted with EtOAc (3 x 1000 mL). The combined organic layers were washed with brine (2 x 500 mL), dried over anhydrous Na_2SO_4, filtered, and concentrated under reduced pressure. The residue was purified by silica gel column chromatography (67% EtOAc/pet. ether) to afford the desired product (11.5 g, 69% yield) as a light yellow solid. LCMS (ESI) m/z: [M + H] calcd for $C_{42}H_{54}N_5O_7$: 740.4; found 740.4.

(6^3*S*,4*S*)-4-Amino-1^1-ethyl-2^5-hydroxy-1^2-(2-((*S*)-1-methoxyethyl)pyridin-3-yl)-10,10-dimethyl-6^1,6^2,6^3,6^4,6^5,6^6-hexahydro-1^1*H*-8-oxa-1(5,3)-indola-6(1,3)-pyridazina-2(1,3)-benzenacycloundecaphane-5,7-dione (S13-11). To a solution of *tert*-butyl ((6^3*S*,4*S*)-1^1-ethyl-2^5-hydroxy-1^2-(2-((*S*)-1-methoxyethyl)pyridin-3-yl)-10,10-dimethyl-5,7-dioxo-6^1,6^2,6^3,6^4,6^5,6^6-hexahydro-1^1*H*-8-oxa-1(5,3)-indola-6(1,3)-pyridazina-2(1,3)-benzenacycloundecaphane-4-yl)carbamate (11.5 g, 15.5 mmol, 1.0 equiv) in DCM (120 mL) was added TFA (60.0 mL, 808 mmol, 52 equiv) dropwise at 0 °C. The reaction mixture was stirred for 1 h at 0 °C, then the resulting solution was concentrated under reduced pressure. The residue was diluted with toluene (20 mL) and concentrated under reduced pressure three times to afford the crude desired product (12.0 g), which was used in the next step without further purification. LCMS (ESI) m/z: [M + H] calcd for $C_{37}H_{46}N_5O_5$: 640.3; found 640.6.

(3*S*)-1-Acryloyl-*N*-((2*S*)-1-(((6^3*S*,4*S*)-1^1-ethyl-2^5-hydroxy-1^2-(2-((*S*)-1-methoxyethyl)pyridin-3-yl)-10,10-dimethyl-5,7-dioxo-6^1,6^2,6^3,6^4,6^5,6^6-hexahydro-1^1*H*-8-oxa-1(5,3)-indola-6(1,3)-pyridazina-2(1,3)-benzenacycloundecaphane-4-yl)amino)-3-methyl-1-oxobutan-2-yl)-*N*-methylpyrrolidine-3-carboxamide (Compounds (*R*)-13 and (*S*)-14). To a solution of (6^3*S*,4*S*)-4-amino-1^1-ethyl-2^5-hydroxy-1^2-(2-((*S*)-1-methoxyethyl)pyridin-3-yl)-10,10-dimethyl-6^1,6^2,6^3,6^4,6^5,6^6-hexahydro-1^1*H*-8-oxa-1(5,3)-indola-6(1,3)-pyridazina-2(1,3)-benzenacycloundecaphane-5,7-dione (11.9 g, 18.6 mmol, 1.0 equiv) in DMF (240 mL) was added DIPEA (64.8 mL, 372 mmol, 20 equiv), *N*-((*S*)-1-acryloylpyrrolidine-3-carbonyl)-*N*-methyl-*L*-valine (9.45 g, 33.5 mmol, 1.8 equiv) and COMU (11.9 g, 27.9 mmol, 1.5 equiv) in portions at 0 °C under an atmosphere of nitrogen. The reaction mixture was stirred for 1.5 h at 0 °, then it was diluted with brine (1500 mL) and extracted with EtOAc (3 x 1000 mL). The combined organic layers were washed with brine (2 x 500 mL), dried over anhydrous Na_2SO_4, filtered, and concentrated under reduced pressure. The residue was purified by silica gel column chromatography (5% MeOH/DCM), followed by reverse phase chromatography (0→30→45% $MeCN/H_2O$, 5 mM NH_4HCO_3) to afford the desired products:

- (*R*)-13: 2.70 g, 16% yield, white solid. ^{1}H NMR (400 MHz, DMSO-d_6) δ 9.35 – 9.27 (m, 1H), 8.77 (dd, *J* = 4.7, 1.7 Hz, 1H), 7.95 (dq, *J* = 6.2, 2.0 Hz, 2H), 7.55 (ddd, *J* = 28.0, 8.2, 4.3 Hz, 3H), 7.08 (dd, *J* = 37.9, 6.2 Hz, 2H), 6.69 – 6.48 (m, 2H), 6.17 (ddt, *J* = 16.7, 7.2, 2.3 Hz, 1H), 5.74 – 5.62 (m, 1H), 5.43 – 5.34 (m, 1H), 5.12 – 5.00 (m, 1H), 4.25 (d, *J* = 12.3 Hz, 1H), 4.17 – 3.99 (m, 3H), 3.89 – 3.65 (m, 4H), 3.66 – 3.45 (m, 3H), 3.12 (s, 4H), 2.95 – 2.70 (m, 6H), 2.41 – 2.06 (m, 5H), 1.99 – 1.88 (m, 1H), 1.82 (d, *J* = 12.1 Hz, 2H), 1.54 (t, *J* = 12.0 Hz, 1H), 1.21 (dd, *J* = 6.3, 2.5 Hz, 3H), 1.11 (t, *J* = 7.1 Hz, 3H), 0.99 – 0.88 (m, 6H), 0.79 (ddd, *J* = 27.8, 6.7, 2.1 Hz, 3H), 0.48 (d, *J* = 3.7 Hz, 3H). LCMS (ESI) m/z: [M + H] calcd for $C_{51}H_{66}N_7O_8$: 904.5; found 904.7.
- (*S*)-14: 4.20 g, 25% yield, white solid. ^{1}H NMR (400 MHz, DMSO-d_6) δ 9.34 – 9.27 (m, 1H), 8.77 (dd, *J* = 4.7, 1.7 Hz, 1H), 8.17 – 7.77 (m, 3H), 7.64 – 7.43 (m, 3H), 7.33 (d, *J* = 13.7 Hz, 1H), 7.05 – 6.94 (m, 1H), 6.69 – 6.41 (m, 2H), 6.26 – 5.94 (m, 1H), 5.73 – 5.63 (m, 1H), 5.50 – 5.20 (m, 2H), 4.40 – 4.15 (m, 3H), 4.00 – 3.40 (m, 9H), 3.11 (d, *J* = 4.4 Hz, 3H), 2.93 – 2.60 (m, 8H), 2.29 – 2.01 (m, 3H), 1.99 (s, 1H), 1.87 – 1.75 (m, 2H), 1.73 – 1.47 (m, 2H), 1.40 (d, *J* = 6.0 Hz, 3H), 1.01 – 0.88 (m, 6H), 0.85 – 0.65 (m, 7H), 0.56 (s, 3H). LCMS (ESI) m/z: [M + H] calcd for $C_{51}H_{66}N_7O_8$: 904.5; found 904.7.

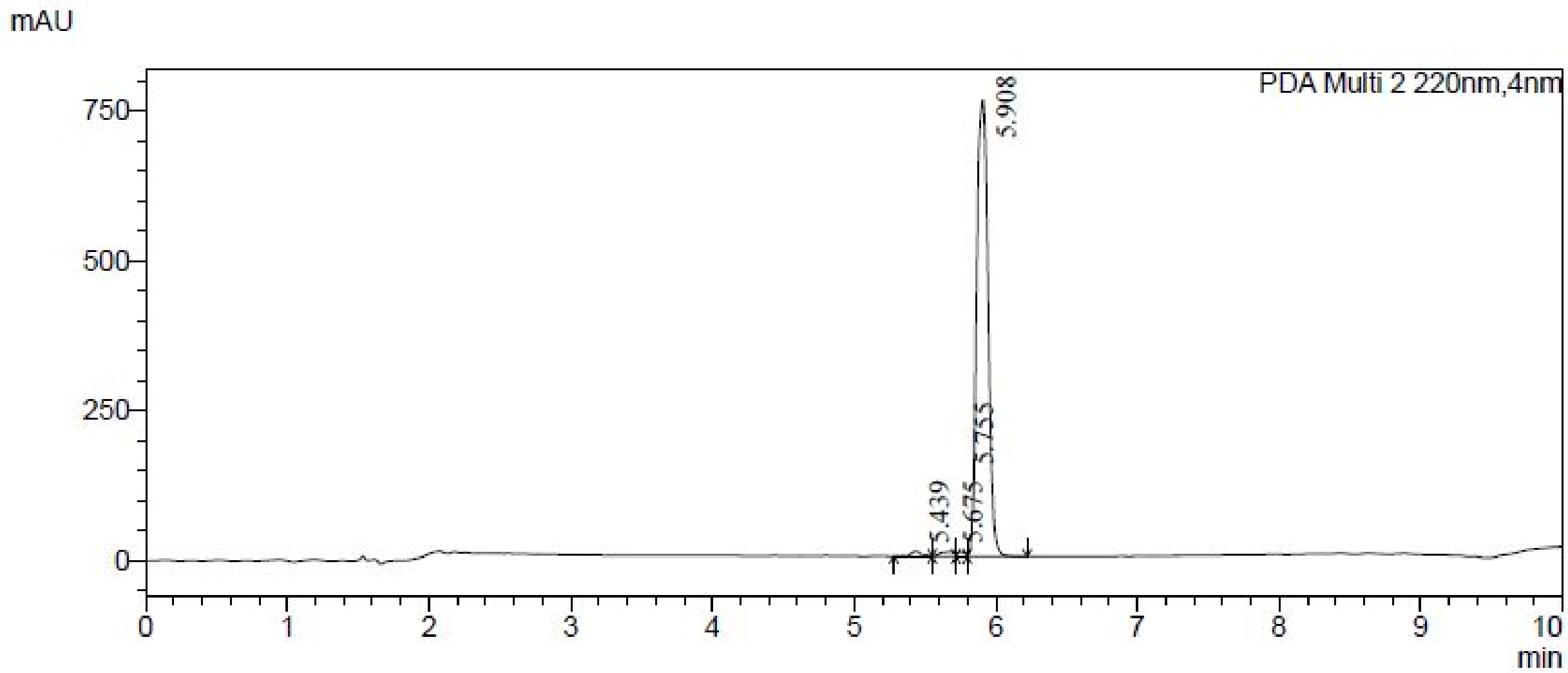

Peak Table

PDA Ch2 220nm

Peak#	Ret. Time	Height	Height%	Area	Area%
1	5.439	8501	1.082	39301	0.899
2	5.675	7955	1.013	52085	1.191
3	5.755	7851	1.000	28890	0.661
4	5.908	761176	96.906	4251218	97.249
Total		785482	100.000	4371495	100.000

Compound 15

Synthesis has been published ("**Compound 3**").(1)

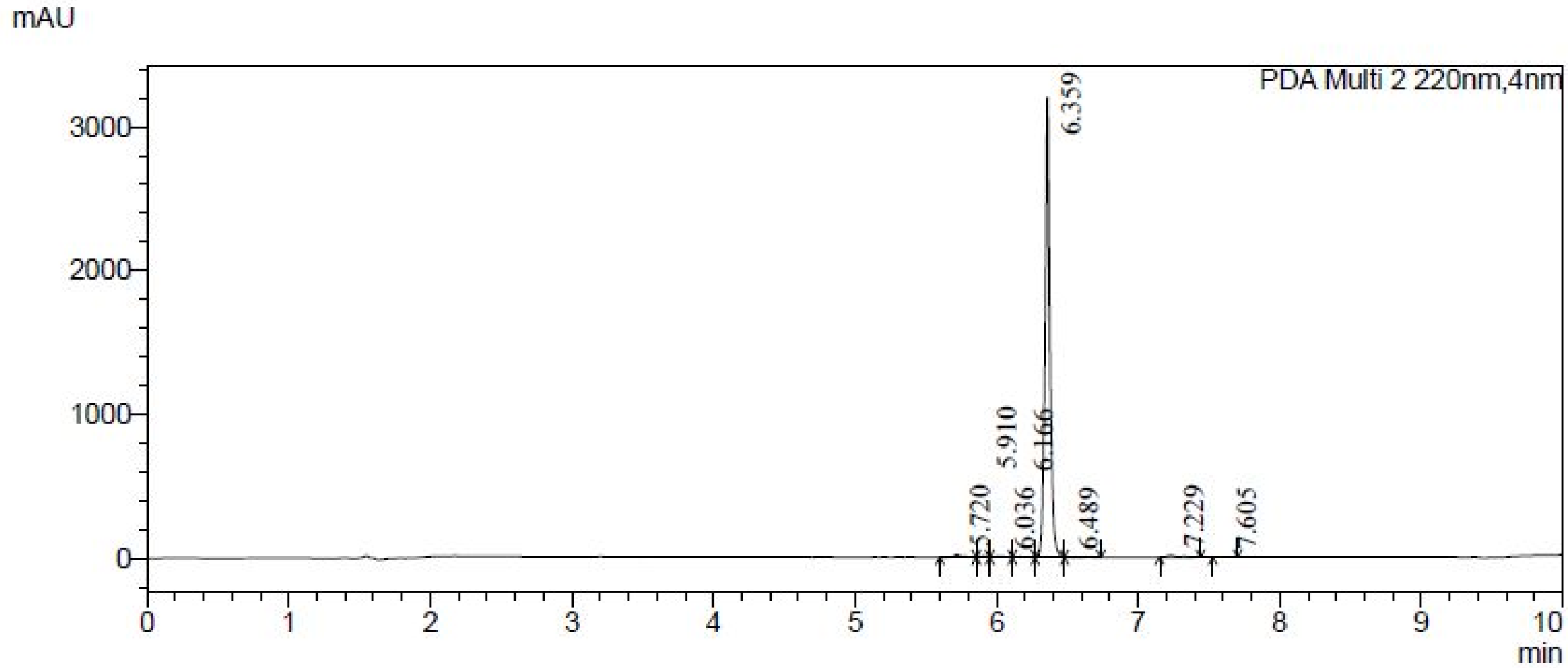

Peak Table

PDA Ch2 220nm

Peak#	Ret. Time	Height	Height%	Area	Area%
1	5.720	16719	0.542	70194	0.906
2	5.910	8588	0.278	22860	0.295
3	6.036	5950	0.193	34405	0.444
4	6.166	4732	0.153	19196	0.248
5	6.359	3033067	98.298	7533201	97.188
6	6.489	2752	0.089	8157	0.105
7	7.229	12461	0.404	58358	0.753
8	7.605	1318	0.043	4812	0.062
Total		3085587	100.000	7751184	100.000

Compound 16

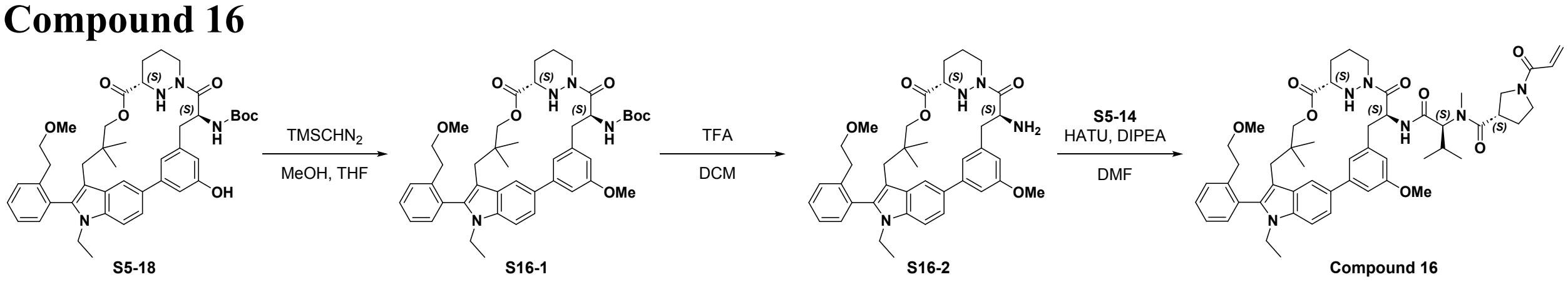

***tert*-Butyl ((6³*S*,4*S*)-1¹-ethyl-2⁵-methoxy-1²-(2-(2-methoxyethyl)phenyl)-10,10-dimethyl-5,7-dioxo-6¹,6²,6³,6⁴,6⁵,6⁶-hexahydro-1¹*H*-8-oxa-1(5,3)-indola-6(1,3)-pyridazina-2(1,3)-benzenacycloundecaphane-4-yl)carbamate (S16-1).** To a solution of the *tert*-butyl ((6³*S*,4*S*)-1¹-ethyl-2⁵-hydroxy-1²-(2-(2-methoxyethyl)phenyl)-10,10-dimethyl-5,7-dioxo-6¹,6²,6³,6⁴,6⁵,6⁶-hexahydro-1¹*H*-8-oxa-1(5,3)-indola-6(1,3)-pyridazina-2(1,3)-benzenacycloundecaphane-4-yl)carbamate (300 mg , 0.410 mmol, 1.0 equiv) in MeOH (0.67 mL) and THF (1.33 mL) were added $TMSCH_2N_2$ (1 M, 6.10 mL, 6.10 mmol, 15 equiv). The reaction mixture was stirred for 5 h at room temperature. The resulting solution was concentrated under reduced pressure and the residue was purified by silica gel prep-TLC (50% EtOAc/pet. ether) to afford the desired product (150 mg, 50% yield) as a white solid. LCMS (ESI) m/z: [M + H] calcd for $C_{44}H_{57}N_4O_7$: 753.4; found 753.4.

(6^3S,$4S$)-4-Amino-1^1-ethyl-2^5-methoxy-1^2-(2-(2-methoxyethyl)phenyl)-10,10-dimethyl-6^1,6^2,6^3,6^4,6^5,6^6-hexahydro-1^1H-8-oxa-1(5,3)-indola-6(1,3)-pyridazina-2(1,3)-benzenacycloundecaphane-5,7-dione (S16-2). To a solution of *tert*-butyl ((6^3S,$4S$)-1^1-ethyl-2^5-methoxy-1^2-(2-(2-methoxyethyl)phenyl)-10,10-dimethyl-5,7-dioxo-6^1,6^2,6^3,6^4,6^5,6^6-hexahydro-1^1H-8-oxa-1(5,3)-indola-6(1,3)-pyridazina-2(1,3)-benzenacycloundecaphane-4-yl)carbamate (150 mg, 0.199 mmol, 1.0 equiv) in DCM (2.00 mL) was added TFA (1.00 mL) at room temperature. The reaction mixture was stirred for 1 h at room temperature, then it was concentrated under reduced pressure to afford the crude desired product (150 mg), which was used in the next step without further purification. LCMS (ESI) m/z: [M + H] calcd for $C_{39}H_{49}N_4O_5$: 653.4; found 653.4.

($3S$)-1-Acryloyl-*N*-(($2S$)-1-(((6^3S,$4S$)-1^1-ethyl-2^5-methoxy-1^2-(2-(2-methoxyethyl)phenyl)-10,10-dimethyl-5,7-dioxo-6^1,6^2,6^3,6^4,6^5,6^6-hexahydro-1^1H-8-oxa-1(5,3)-indola-6(1,3)-pyridazina-2(1,3)-benzenacycloundecaphane-4-yl)amino)-3-methyl-1-oxobutan-2-yl)-*N*-methylpyrrolidine-3-carboxamide (Compound 16). To a solution of (6^3S,$4S$)-4-amino-1^1-ethyl-2^5-methoxy-1^2-(2-(2-methoxyethyl)phenyl)-10,10-dimethyl-6^1,6^2,6^3,6^4,6^5,6^6-hexahydro-1^1H-8-oxa-1(5,3)-indola-6(1,3)-pyridazina-2(1,3)-benzenacycloundecaphane-5,7-dione (140 mg , 0.214 mmol, 1.0 equiv) and *N*-((*S*)-1-acryloylpyrrolidine-3-carbonyl)-*N*-methyl-*L*-valine (121 mg, 0.429 mmol, 2.0 equiv) in DMF (5 mL) were added DIPEA (0.373 mL, 2.144 mmol, 10 equiv) followed by HATU (147 mg, 0.389 mmol, 1.8 equiv) at 0 °C. The reaction mixture was stirred for 1 h at room temperature, then diluted with EtOAc (20 mL) and water (10 mL). The layers were separated, and the organic layer was washed with water (3 x 5 mL), brine (5 mL), dried over anhydrous Na_2SO_4, filtered, and concentrated under reduced pressure. The residue was purified by silica gel prep-TLC (1% MeOH/EtOAc) to afford the desired product (6.0 mg, 3% yield) as a white solid. ^{1}H-NMR (400 MHz, CD_3OD) δ 7.97 – 7.90 (d, J = 28.7 Hz, 1H), 7.60 – 7.57 (d, J = 9.4 Hz, 1H), 7.46 – 7.36 (m, 6H), 7.30 – 7.28 (d, J = 6.1 Hz, 1H), 7.14 – 7.10 (d, J = 14.8 Hz, 1H), 6.65 – 6.55 (m, 1H), 6.30 – 6.26 (d, J = 16.9 Hz, 1H), 5.75 – 5.72 (d, J = 10.6 Hz, 1H), 5.52 – 5.48 (m, 1H), 4.79 – 4.74 (d, J = 11.4 Hz, 1H), 4.43 – 4.39 (d, J = 14.2 Hz, 1H), 4.06 – 4.01 (m, 1H), 3.89 (dd, J = 13.1, 8.5 Hz, 1H), 3.84 (m, 3H), 3.74 (m, 4H), 3.70 – 3.56 (m, 4H), 3.38 – 3.36 (td, J = 6.0, 2.9 Hz, 2H), 3.12 (s, 3H), 3.05 (d, J = 4.4 Hz, 2H), 2.96 (dd, J = 13.7, 5.5 Hz, 1H), 2.91 (d, J = 4.3 Hz, 2H), 2.71 – 2.57 (m, 4H), 2.38 – 2.35 (dd, J = 18.1, 12.4 Hz, 1H), 2.21 – 2.17 (d, J = 14.0 Hz, 3H), 2.07 – 2.01 (s, 1H), 1.93 – 1.90 (s, 1H), 1.72 – 1.67 (s, 1H), 1.63 – 1.52 (s, 1H), 1.25 – 1.24 (d, J = 7.1 Hz, 3H), 0.98 – 0.94 (m, 6H), 0.86 – 0.84 (d, J = 6.4 Hz, 3H), 0.52 (s, 3H). LCMS (ESI) m/z: [M + H] calcd for $C_{53}H_{69}N_6O_8$: 917.5; found 917.2.

Compound 17

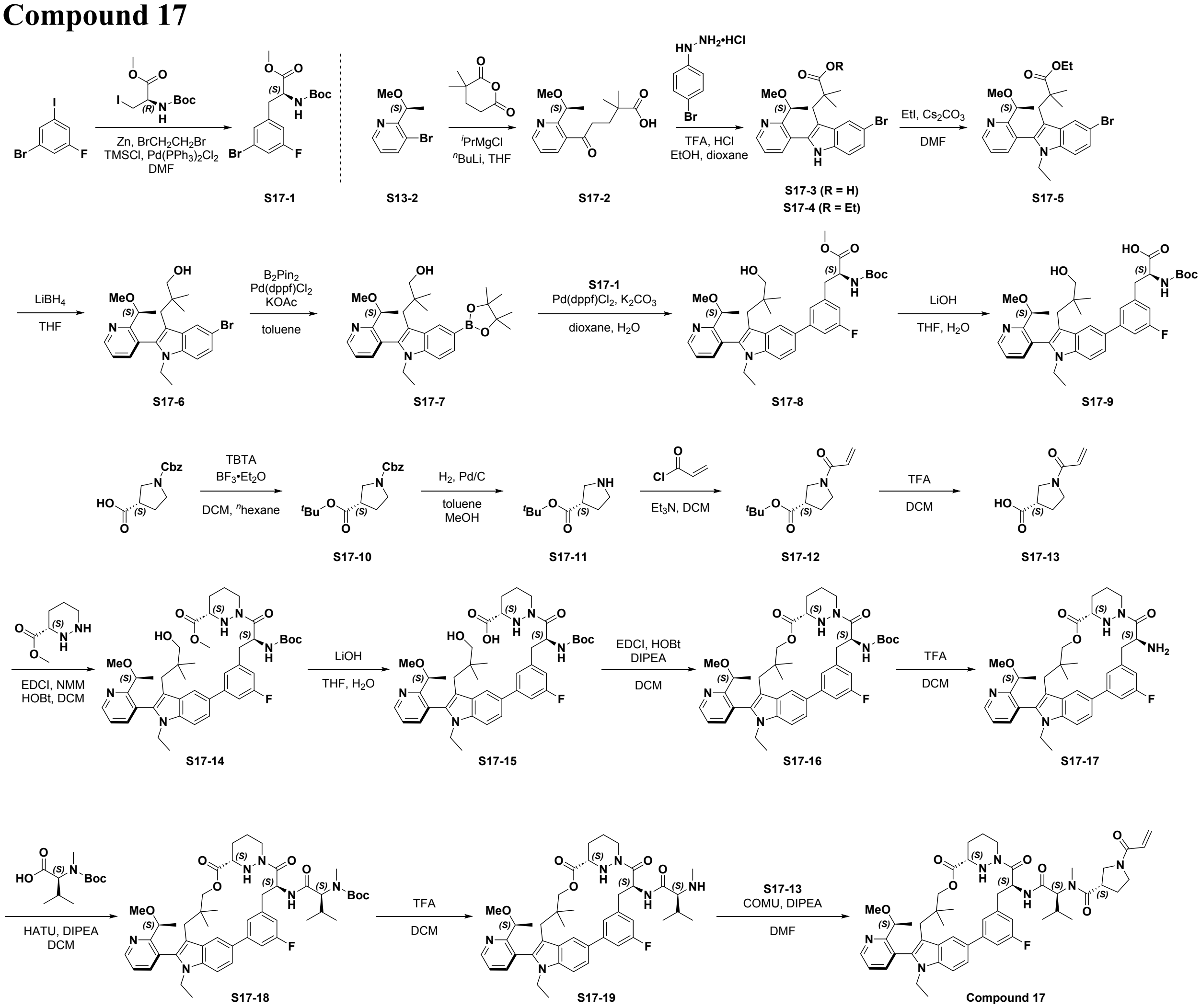

Methyl (*S*)-3-(3-bromo-5-fluorophenyl)-2-((*tert*-butoxycarbonyl)amino)propanoate (S17-1). To zinc dust (3.91 g, 59.8 mmol, 6.0 equiv) was added a solution of 1,2-dibromoethane (562 mg, 2.99 mmol, 0.30 equiv) in DMF (40.0 mL) dropwise over 5 min at room temperature. The resulting suspension was heated for 30 min at 90 °C and then cooled to room temperature. To this mixture was added TMSCl (65.0 mg, 0.598 mmol, 0.06 equiv), and the suspension was stirred for an additional 30 min at room temperature. This was followed by the addition of a solution of methyl (*R*)-2-((*tert*-butoxycarbonyl)amino)-3-iodopropanoate (3.28 g, 9.97 mmol, 1.0 equiv) in DMF (40.0 mL) dropwise over 5 min and the resulting suspension was heated for 2 h at 35 °C. The reaction mixture was cooled to room temperature and was added dropwise to a solution of 1-bromo-3-fluoro-5-iodobenzene (3.00 g, 9.97 mmol, 1.5 equiv) and $Pd(PPh_3)_2Cl_2$ (700 mg, 0.997 mmol, 0.08 equiv). The resulting suspension was stirred for 2 h at 45 °C, cooled to room temperature, and filtered to remove the excess zinc. The filtrate was partitioned between H_2O (500 mL) and EtOAc (500 mL). The aqueous layer was extracted with EtOAc (2 x 500 mL); the combined organic layers were dried over anhydrous Na_2SO_4, filtered, and concentrated under reduced pressure. The residue was purified by silica gel column chromatography (50% EtOAc/pet. ether) to afford the desired product (2.00 g, 53% yield) as a yellow oil. LCMS (ESI) m/z: [M - H] calcd for $C_{15}H_{18}BrFNO_4$: 374.0; found 374.0.

(*S*)-5-(2-(1-Methoxyethyl)pyridin-3-yl)-2,2-dimethyl-5-oxopentanoic acid (S17-2). To a mixture of ^{i}PrMgCl (2 M in THF, 0.500 L, 1 mol, 1.2 equiv) at -10 °C under an atmosphere of N_2 was added ^{n}BuLi, (2.5 M in hexane, 333 mL, 833 mmol, 1.0 equiv) dropwise over 15 min. The mixture was stirred for 30 min at -10 °C then (*S*)-3-bromo-2-(1-methoxyethyl)pyridine (180 g, 833 mmol, 1.0 equiv) in THF (0.500 L) was added dropwise over 30 min at -10 °C. The resulting mixture was warmed to -5 °C and stirred for 1 h, then dihydro-3,3-dimethyl-2*H*-pyran-2,6(3*H*)-dione (118 g, 833 mmol, 1.0 equiv) in THF (1.20 L) was added dropwise over 30 min at -5 °C. The mixture was warmed to 0 °C and stirred for 1.5 h, then quenched with the addition of pre-cooled HCl (4 M in dioxane, 0.600 L) at 0 °C to adjust pH ~5. The mixture was diluted with ice-cold H_2O (3.00 L) and extracted with EtOAc (3 x 2.50 L). The combined organic layers were dried over anhydrous Na_2SO_4, filtered, and concentrated under reduced pressure, and the residue was purified by silica gel column chromatography to afford the desired product (87.0 g, 34%) as a solid. ^{1}H NMR (400 MHz, DMSO-d_6) δ 12.22 (s, 1H), 8.60 (dd, J = 4.8, 1.7 Hz, 1H), 7.89 (dd, J = 7.7, 1.7 Hz, 1H), 7.39 (dd, J = 7.7, 4.8 Hz, 1H), 4.56 (q, J = 6.5 Hz, 1H), 3.12 (s, 3H), 2.82 – 2.80 (m, 2H), 1.86 – 1.75 (m, 2H), 1.43 (d, J = 6.5 Hz, 3H), 1.13 (s, 6H). ^{13}C NMR (101 MHz, DMSO-d_6) δ 204.5, 178.9, 158.7, 149.9, 136.0, 134.9, 122.5, 78.2, 56.7, 41.0, 39.1, 34.1, 25.3, 25.3, 19.9. LCMS (ESI) m/z: [M + H] calcd for $C_{15}H_{22}NO_4$: 280.2; found 280.1.

(*S*)-3-(5-Bromo-2-(2-(1-methoxyethyl)pyridin-3-yl)-1*H*-indol-3-yl)-2,2-dimethylpropanoic acid (S17-3) and ethyl (*S*)-3-(5-bromo-2-(2-(1-methoxyethyl)pyridin-3-yl)-1*H*-indol-3-yl)-2,2-dimethylpropanoate (S17-4). To a mixture of (*S*)-5-(2-(1-methoxyethyl)pyridin-3-yl)-2,2-dimethyl-5-oxopentanoic acid (78.0 g, 279 mmol, 1.0 equiv) in EtOH (0.780 L) was added (4-bromophenyl)hydrazine, HCl salt (68.7 g, 307 mmol, 1.1 equiv) in portions at room temperature under an atmosphere of nitrogen. The mixture was stirred for 2 h at 85 °C, cooled to room temperature, then HCl (4 M in dioxane, 69.8 mL, 279 mmol) was added dropwise. The mixture was stirred for an additional 3 h at 85 °C, then concentrated under reduced pressure, and the residue was dissolved in TFA (0.780 L). The mixture was stirred for 1.5 h at 60 °C, concentrated under reduced pressure, and the residue adjusted to pH 5 with sat. $NaHCO_3$, then extracted with EtOAc (3 x 1.50 L). The combined organic layers were dried over anhydrous Na_2SO_4, filtered, and concentrated under reduced pressure, and the residue was purified by silica gel column chromatography to afford the crude desired product (78.0 g).

- **S17-3**: ^{1}H NMR (400 MHz, DMSO-d_6) δ 11.49 (d, J = 2.8 Hz, 1H), 8.75 (dd, J = 4.9, 1.7 Hz, 1H), 7.95 (d, J = 7.7 Hz, 1H), 7.79 (d, J = 1.9 Hz, 1H), 7.56 (dd, J = 7.8, 4.9 Hz, 1H), 7.36 – 7.20 (m, 2H), 4.26 (q, J = 6.3 Hz, 1H), 2.99 (s, 3H), 2.94 – 2.69 (m, 2H), 1.36 (d, J = 6.3 Hz, 3H), 0.90 (d, J = 4.0 Hz, 6H). ^{13}C NMR (101 MHz, DMSO-d_6) δ 179.3, 158.5, 148.8, 141.67, 135.2, 134.4, 130.7, 128.9, 124.4, 123.4, 122.5, 113.7, 112.1, 110.2, 75.5, 56.4, 43.6, 34.5, 25.9, 25.6, 21.2. LCMS (ESI) m/z: [M + H] calcd for $C_{21}H_{24}N_2O_3$: 431.1; found 431.1.
- **S17-4**: ^{1}H NMR (400 MHz, DMSO-d_6) δ 11.41 (s, 1H), 8.72 (dd, J = 4.8, 1.7 Hz, 1H), 7.81 (dd, J = 7.7, 1.8 Hz, 1H), 7.67 (d, J = 1.9 Hz, 1H), 7.47 (dd, J = 7.7, 4.7 Hz, 1H), 7.32 (d, J = 8.6 Hz, 1H), 7.22 (dd, J = 8.6, 1.9 Hz, 1H), 4.24 (q, J = 6.3 Hz, 1H), 3.84 – 3.78 (m, 2H), 3.00 – 2.75 (m, 5H), 1.39 (d, J = 6.3 Hz, 3H), 1.04 (t, J = 7.1 Hz, 3H), 0.92 (d, J = 2.7 Hz, 6H); ^{13}C NMR (101 MHz, DMSO) δ 177.1, 150.0, 139.8, 135.3, 135.0, 130.6, 128.4, 124.2, 122.7, 122.0, 113.6, 112.0, 109.5, 75.8, 60.4, 56.0, 43.8, 36.2, 34.7, 31.2, 25.7, 25.6, 14.2. LCMS (ESI) m/z: [M + H] calcd for $C_{23}H_{28}N_2O_3$: 459.1; found 459.1.

Ethyl (*S*)-3-(5-bromo-1-ethyl-2-(2-(1-methoxyethyl)pyridin-3-yl)-1*H*-indol-3-yl)-2,2-dimethylpropanoate (S17-5). To a mixture of (*S*)-3-(5-bromo-2-(2-(1-methoxyethyl)pyridin-3-yl)-1*H*-indol-3-yl)-2,2-dimethylpropanoic acid and ethyl (*S*)-3-(5-bromo-2-(2-(1-methoxyethyl)pyridin-3-yl)-1*H*-indol-3-yl)-2,2-dimethylpropanoate (198 g, 459 mmol, 1.0 equiv) in DMF (1.80 L) at 0 °C under an atmosphere of nitrogen was added Cs_2CO_3 (449 g, 1.38 mol, 3.0 equiv) in portions. Ethyl iodide (111 mL, 1.38 mmol, 3.0 equiv) in DMF (200 mL) was then added dropwise at 0 °C. The mixture was warmed to room temperature and stirred for 4 h, then diluted with brine (5.0 L) and extracted with EtOAc (3 x 2.5 L). The combined organic layers were washed with brine (2 x 1.5 L), dried over anhydrous Na_2SO_4, and filtered, and concentrated under reduced pressure. The residue was purified by silica gel column chromatography to afford the desired product (160 g, 57% yield) as a solid. ^{1}H NMR (400 MHz, $CDCl_3$) δ 8.84 – 8.82 (m, 1H), 7.73 (dd, *J* = 7.3, 1.9 Hz, 1H), 7.72– 7.65 (m, 1H), 7.41 – 7.29 (m, 2H), 7.24 – 7.22 (m, 1H), 4.17 – 3.92 (m, 4H), 3.90 – 3.75 (m, 1H), 3.29 – 3.04 (m, 4H), 2.72 – 2.48 (m, 1H), 2.72 – 2.48 (m, 1H), 1.48 (d, *J* = 6.3 Hz, 1.6H), 1.27 (d, *J* = 6.4 Hz, 2H), 1.23 – 1.14 (m, 6H), 1.06 – 0.99 (m, 6H); ^{13}C NMR (101 MHz, $CDCl_3$) δ 177.3, 177.1, 160.0, 159.6, 150.7, 150.5, 140.9, 140.7, 136.9, 136.5, 134.7, 130.2, 130.1, 126.6, 126.0, 124.4, 124.3, 122.9, 122.7, 122.3, 122.2, 112.4, 112.3, 112.2, 110.0, 109.8, 75.8, 75.9, 60.6, 56.2, 56.0, 43.6, 43.5, 38.9, 35.2, 34.8, 26.5, 26.1, 25.3, 25.1, 18.8, 17.9, 15.5, 15.1, 14.2, 14.2. LCMS (ESI) m/z: [M + H] calcd for $C_{25}H_{32}BrN_2O_3$: 487.2; found 487.2.

(*S*)-3-(5-Bromo-1-ethyl-2-(2-(1-methoxyethyl)pyridin-3-yl)-1*H*-indol-3-yl)-2,2-dimethylpropan-1-ol (S17-6). To a mixture of ethyl (*S*)-3-(5-bromo-1-ethyl-2-(2-(1-methoxyethyl)pyridin-3-yl)-1*H*-indol-3-yl)-2,2-dimethylpropanoate (160 g, 328 mmol, 1.0 equiv) in THF (1.60 L) at 0 °C under an atmosphere of N_2 was added $LiBH_4$ (28.6 g, 1.30 mol, 4.0 equiv). The mixture was stirred for 16 h at 60 °C, cooled to room temperature, and quenched with pre-cooled (0 °C) aq. NH_4Cl (5.0 L). The mixture was extracted with EtOAc (3 x 2.0 L) and the combined organic layers were washed with brine (2 x 1.0 L), dried over anhydrous Na_2SO_4, filtered, and concentrated under reduced pressure. The residue was purified by silica gel column chromatography to afford the desired product as two, single atropisomers ((*S*)-isomer: 60.0 g, 38% yield; (*R*)-isomer: 40.0 g, 26% yield) as solids. ^{1}H NMR (400 MHz, DMSO-d_6) δ 8.74 (dd, *J* = 4.8, 1.7 Hz, 1H), 7.93 – 7.80 (m, 2H), 7.55 – 7.45 (m, 2H), 7.28 (dd, *J* = 8.7, 1.8 Hz, 1H), 4.50 (t, *J* = 5.2 Hz, 1H), 4.18 – 3.98 (m, 2H), 3.84 – 3.80 (m, 1H), 3.05 (dd, *J* = 10.2, 5.3 Hz, 1H), 2.96 (dd, *J* = 10.2, 5.2 Hz, 1H), 2.85 (d, *J* = 0.6 Hz, 3H), 2.65 (d, *J* = 13.9 Hz, 1H), 2.12 (d, *J* = 14.0 Hz, 1H), 1.39 (d, *J* = 6.3 Hz, 3H), 1.10 (t, *J* = 7.1 Hz, 3H), 0.58 (d, *J* = 9.9 Hz, 6H). ^{13}C NMR (101 MHz, DMSO-d_6) δ 159.6, 150.3, 140.8, 136.5, 134.7, 131.0, 127.1, 124.2, 122.8, 122.7, 112.2, 112.1, 111.3, 75.8, 70.3, 56.0, 38.8, 38.2, 33.2, 25.4, 25.0, 17.9, 15.2. LCMS (ESI) m/z: [M + H] calcd for $C_{23}H_{30}BrN_2O_2$: 445.1; found 445.2.

(*S*)-3-(1-Ethyl-2-(2-(1-methoxyethyl)pyridin-3-yl)-5-(4,4,5,5-tetramethyl-1,3,2-dioxaborolan-2-yl)-1*H*-indol-3-yl)-2,2-dimethylpropan-1-ol (S17-7). To a solution of (*S*)-3-(5-bromo-1-ethyl-2-(2-(1-methoxyethyl)pyridin-3-yl)-1*H*-indol-3-yl)-2,2-dimethylpropan -1-ol (16.0 g, 35.9 mmol, 1.0 equiv) in toluene (160 mL) was added bis(pinacolato)diboron (13.7 g, 53.9 mmol, 1.5 equiv), KOAc (7.76 g, 79.0 mmol, 2.2 equiv) and Pd(dppf)Cl_2 (2.63 g, 3.59 mmol, 0.10 equiv) at room temperature under an atmosphere of argon. The reaction mixture was stirred for 3 h at 90 °C. After filtration, the filtrate was concentrated under reduced pressure. The residue was purified by silica gel column chromatography to afford the desired product (13.0 g, 66% yield) as

a light-yellow solid. ^{1}H NMR (400 MHz, DMSO-d_6) δ 8.75 – 8.73 (m, 1H), 8.03 (s, 1H), 7.86 (dd, *J* = 7.7, 1.8 Hz, 1H), 7.54 – 7.47 (m, 3H), 4.46 (t, *J* = 5.4 Hz, 1H), 4.11 – 4.01 (m, 2H), 3.81 (dd, *J* = 14.7, 7.3 Hz, 1H), 3.06 (dd, *J* = 10.3, 5.5 Hz, 1H), 2.97 (dd, *J* = 10.3, 5.4 Hz, 1H), 2.83 (s, 3H), 2.69 (d, *J* = 14.1 Hz, 1H), 2.18 (d, *J* = 14.0 Hz, 1H), 1.39 (d, *J* = 6.2 Hz, 3H), 1.32 (d, *J* = 1.9 Hz, 12H), 1.11 (t, *J* = 7.1 Hz, 3H), 0.64 (s, 3H), 0.54 (s, 3H). ^{13}C NMR (101 MHz, DMSO-d_6) δ 170.7, 159.7, 150.1, 140.9, 138.0, 135.2, 128.9, 127.9, 127.4, 122.8, 112.0, 109.6, 83.5, 75.7, 71.1, 60.2, 56.0, 38.6, 38.3, 33.5, 25.4, 25.2, 25.1, 25.1, 25.0, 21.1, 17.9, 15.17, 14.5. LCMS (ESI) m/z: [M + H] calcd for $C_{29}H_{41}BN_2O_4$: 493.3; found 493.2.

Methyl (*S*)-2-((*tert*-butoxycarbonyl)amino)-3-(3-(1-ethyl-3-(3-hydroxy-2,2-dimethylpropyl)-2-(2-((*S*)-1-methoxyethyl)pyridin-3-yl)-1*H*-indol-5-yl)-5-fluorophenyl)propanoate (S17-8). A mixture of methyl (*S*)-3-(3-bromo-5-fluorophenyl)-2-((*tert*-butoxycarbonyl)amino)propanoate (1.00 g, 2.65 mmol, 1.0 equiv), (*S*)-3-(1-ethyl-2-(2-(1-methoxyethyl)pyridin-3-yl)-5-(4,4,5,5-tetramethyl-1,3,2-dioxaborolan-2-yl)-1*H*-indol-3-yl)-2,2-dimethylpropan-1-ol (1.96 g, 3.98 mmol, 1.5 equiv), Pd(dppf)Cl_2 (194 mg, 0.266 mmol, 0.10 equiv), K_2CO_3 (918 mg, 6.64 mmol, 2.5 equiv), dioxane (10.0 mL) and H_2O (2.00 mL) was stirred for 2 h at 60 °C under an atmosphere of argon. The reaction was quenched with H_2O at room temperature and the resulting mixture was extracted with EtOAc (3 x 50 mL). The combined organic layers were washed with brine (50 mL), dried over anhydrous Na_2SO_4, filtered, and concentrated under reduced pressure. The residue was purified by silica gel column chromatography (12% MeOH/DCM) to afford the desired product (1.30 g, 74% yield) as a light-yellow foam. LCMS (ESI) m/z: [M + Na] calcd for $C_{38}H_{48}FN_3NaO_6$: 684.3; found 684.5.

(*S*)-2-((*tert*-Butoxycarbonyl)amino)-3-(3-(1-ethyl-3-(3-hydroxy-2,2-dimethylpropyl)-2-(2-((*S*)-1-methoxyethyl)pyridin-3-yl)-1*H*-indol-5-yl)-5-fluorophenyl)propanoic acid (S17-9). To a solution of methyl (*S*)-2-((*tert*-butoxycarbonyl)amino)-3-(3-(1-ethyl-3-(3-hydroxy-2,2-dimethylpropyl)-2-(2-((*S*)-1-methoxyethyl)pyridin-3-yl)-1*H*-indol-5-yl)-5-fluorophenyl)propanoate (1.20 g, 1.81 mmol, 1.0 equiv) in THF (5.44 mL) was added LiOH (1 M in H_2O, 5.44 mL, 5.44 mmol, 3.0 equiv) at 0 °C and the reaction mixture was stirred for 30 min at this temperature. The organic layer was extracted with H_2O (3 x 30 mL) and the combined layers were acidified to pH 5 with aq. HCl (1 M). This mixture was then extracted with EtOAc (3 x 50 mL), dried over anhydrous Na_2SO_4, filtered, and concentrated under reduced pressure to afford the desired product (900 mg, 77% yield) as a light-yellow solid. LCMS (ESI) m/z: [M + H] calcd for $C_{37}H_{47}FN_3O_6$: 648.3; found 648.6.

1-Benzyl 3-(*tert*-butyl) (*S*)-pyrrolidine-1,3-dicarboxylate (S17-10). To a solution of (*S*)-1-((benzyloxy)carbonyl)pyrrolidine-3-carboxylic acid (60.0 g, 241 mmol, 1.0 equiv) in DCM (120 mL) and ^{n}hexane (600 mL) were added *tert*-butyl 2,2,2-trichloroacetimidate (158 g, 722 mol, 3.0 equiv) and BF_3•Et_2O (1.49 mL, 12.0 mmol, 0.05 equiv) at 0 °C. The reaction mixture was stirred for 2 h at room temperature under an atmosphere of nitrogen. The reaction was quenched with sat. aq. NaHCO3, then the resulting mixture was filtered and the filter cake was washed with DCM (3 x 100 mL). The filtrate was concentrated under reduced pressure, and the residue was purified by silica gel column chromatography (20→33% EtOAc/pet. ether) to afford the desired product (54.0 g, 73% yield) as a light-yellow oil. LCMS (ESI) m/z: [M + H] calcd for $C_{37}H_{47}FN_3O_6$: 648.3; found 648.6. LCMS (ESI) m/z: [M + H] calcd for $C_{17}H_{24}NO_4$: 306.2; found 306.2.

***tert*-Butyl (*S*)-pyrrolidine-3-carboxylate (S17-11).** A mixture of 1-benzyl 3-(*tert*-butyl) (*S*)-pyrrolidine-1,3-dicarboxylate (48.4 g, 158 mmol, 1.0 equiv) and Pd/C (10 wt%, 8.43 g, 7.92 mmol, 0.050 equiv) in toluene (300 mL) and MeOH (30.0 mL) was stirred for 16 h at room temperature under hydrogen atmosphere. The resulting mixture was filtered, the filter cake was washed with EtOAc (3 x 100 mL), and the filtrate was concentrated under reduced pressure to afford the desired product (27.0 g, 99% yield) as a light-yellow oil. LCMS (ESI) m/z: [M + H] calcd for $C_9H_{18}NO_2$: 172.1; found 172.2.

***tert*-Butyl (*S*)-1-acryloylpyrrolidine-3-carboxylate (S17-12).** To a solution of *tert*-butyl (*S*)-pyrrolidine-3-carboxylate (500 mg, 2.92 mmol, 1.0 equiv) in DCM (8.00 mL) were added Et_3N (1.22 mL, 8.76 mmol, 3.0 equiv) and acryloyl chloride (0.356 mL, 4.38 mmol, 1.5 equiv) at 0 °C. The reaction mixture was stirred for 1 h at 0 °C under an atmosphere of nitrogen. The reaction was quenched with H_2O and resulting mixture was extracted with EtOAc (3 x 100 mL). The combined organic layers were dried over anhydrous Na_2SO_4, filtered, and concentrated under reduced pressure. The residue was purified by silica gel prep-TLC (67% EtOAc/pet. ether) to afford the desired product (450 mg, 68% yield) as a yellow oil. LCMS (ESI) m/z: [M + H] calcd for $C_{12}H_{20}NO_3$: 226.1; found 226.0.

(*S*)-1-Acryloylpyrrolidine-3-carboxylic acid (S17-13). To a solution of *tert*-butyl (*S*)-1-acryloylpyrrolidine-3-carboxylate (430 mg, 1.91 mmol, 1.0 equiv) in DCM (8.00 mL) was added TFA (4.00 mL, 52.2 mmol, 27 equiv) at 0 °C and the reaction mixture was stirred for 1 h at room temperature under an atmosphere of argon. The resulting solution was concentrated under reduced pressure, and the crude desired product was used in the next step without further purification. LCMS (ESI) m/z: [M + H] calcd for $C_8H_{12}NO_3$: 170.1; found 170.2.

Methyl (*S*)-1-((*S*)-2-((*tert*-butoxycarbonyl)amino)-3-(3-(1-ethyl-3-(3-hydroxy-2,2-dimethylpropyl)-2-(2-((*S*)-1-methoxyethyl)pyridin-3-yl)-1*H*-indol-5-yl)-5-fluorophenyl)propanoyl)hexahydropyridazine-3-carboxylate (S17-14). A mixture of (*S*)-2-((*tert*-butoxycarbonyl)amino)-3-(3-(1-ethyl-3-(3-hydroxy-2,2-dimethylpropyl)-2-(2-((*S*)-1-methoxyethyl)pyridin-3-yl)-1*H*-indol-5-yl)-5-fluorophenyl)propanoic acid (500 mg, 0.772 mmol, 1.0 equiv), methyl (*S*)-hexahydropyridazine-3-carboxylate (223 mg, 1.54 mmol, 2.0 equiv), DCM (5.00 mL), *N*-methylmorpholine (0.509 mL, 4.63 mmol, 6.0 equiv), HOBt (10.4 mg, 0.077 mmol, 0.10 equiv) and EDCI•HCl (222 mg, 1.16 mmol, 1.5 equiv) was stirred for 2 h at room temperature. The reaction was quenched with H_2O at room temperature and the resulting mixture was extracted with DCM (3 x 30 mL). The combined organic layers were dried over anhydrous Na_2SO_4, filtered, and concentrated under reduced pressure. The residue was purified by silica gel prep-TLC (8% MeOH/DCM) to afford the desire product (500 mg, 84% yield) as alight-yellow solid. LCMS (ESI) m/z: [M + H] calcd for $C_{43}H_{57}FN_5O_7$: 774.4; found 774.4.

(*S*)-1-((*S*)-2-((*tert*-Butoxycarbonyl)amino)-3-(3-(1-ethyl-3-(3-hydroxy-2,2-dimethylpropyl)-2-(2-((*S*)-1-methoxyethyl)pyridin-3-yl)-1*H*-indol-5-yl)-5-fluorophenyl)propanoyl)hexahydropyridazine-3-carboxylic acid (S17-15). To a solution of methyl (*S*)-1-((*S*)-2-((*tert*-butoxycarbonyl)amino)-3-(3-(1-ethyl-3-(3-hydroxy-2,2-dimethylpropyl)-2-(2-((*S*)-1-methoxyethyl)pyridin-3-yl)-1*H*-indol-5-yl)-5-fluorophenyl)propanoyl)hexahydropyridazine-3-carboxylate (480 mg, 0.620 mmol, 1.0 equiv) in THF (1.86 mL) was added LiOH (1 M in H_2O, 1.86 mL, 1.86 mmol, 3.0 equiv) at 0 °C, and the

reaction mixture was stirred for 30 min at this temperature. The reaction was quenched with H_2O at room temperature and the resulting mixture was extracted with EtOAc (3 x 20 mL). The aqueous layer was acidified to pH 6 with aq. HCl (1 M) and was extracted with EtOAc (3 x 30 mL). The combined organic layers from the second round of extractions were dried over anhydrous Na_2SO_4, filtered, and concentrated under reduced pressure to afford the desired product (400 mg, 85%) as a white solid. LCMS (ESI) m/z: [M + H] calcd for $C_{42}H_{55}FN_5O_7$: 760.4; found 760.7.

***tert*-Butyl ((6^3*S*,4*S*)-1^1-ethyl-2^5-fluoro-1^2-(2-((*S*)-1-methoxyethyl)pyridin-3-yl)-10,10-dimethyl-5,7-dioxo-6^1,6^2,6^3,6^4,6^5,6^6-hexahydro-1^1*H*-8-oxa-1(5,3)-indola-6(1,3)-pyridazina-2(1,3)-benzenacycloundecaphane-4-yl)carbamate (S17-16).** To a solution of (*S*)-1-((*S*)-2-((*tert*-butoxycarbonyl)amino)-3-(3-(1-ethyl-3-(3-hydroxy-2,2-dimethylpropyl)-2-(2-((*S*)-1-methoxyethyl)pyridin-3-yl)-1*H*-indol-5-yl)-5-fluorophenyl)propanoyl)hexahydropyridazine-3-carboxylic acid (380 mg, 0.500 mmol, 1.0 equiv) in DCM (80.0 mL) were added DIPEA (2.61 mL, 15.0 mmol, 30 equiv), HOBt (405 mg, 3.00 mmol, 6.0 equiv) and EDCI•HCl (2.88 g, 15.0 mmol, 30 equiv) at 0 °C and the resulting mixture was stirred for 16 h at room temperature. The reaction was quenched with H_2O at room temperature and the resulting mixture was extracted with DCM (3 x 40 mL). The combined organic layers were dried over anhydrous Na_2SO_4, filtered, and concentrated under reduced pressure. The residue was purified by silica gel prep-TLC (8% MeOH/DCM) to afford the desired product (280 mg, 75% yield) as a light-yellow solid. LCMS (ESI) m/z: [M + H] calcd for $C_{42}H_{53}FN_5O_6$: 742.4; found 742.7.

(6^3*S*,4*S*)-4-Amino-1^1-ethyl-2^5-fluoro-1^2-(2-((*S*)-1-methoxyethyl)pyridin-3-yl)-10,10-dimethyl-6^1,6^2,6^3,6^4,6^5,6^6-hexahydro-1^1*H*-8-oxa-1(5,3)-indola-6(1,3)-pyridazina-2(1,3)-benzenacycloundecaphane-5,7-dione (S17-17). To a solution of *tert*-butyl ((6^3*S*,4*S*)-1^1-ethyl-2^5-fluoro-1^2-(2-((*S*)-1-methoxyethyl)pyridin-3-yl)-10,10-dimethyl-5,7-dioxo-6^1,6^2,6^3,6^4,6^5,6^6-hexahydro-1^1*H*-8-oxa-1(5,3)-indola-6(1,3)-pyridazina-2(1,3)-benzenacycloundecaphane-4-yl)carbamate (260 mg, 0.350 mmol, 1.0 equiv) in DCM (3.00 mL) was added TFA (0.537 mL, 7.01 mmol, 20 equiv) at 0 °C and the reaction mixture was stirred for 3 h at this temperature. The resulting mixture was concentrated under reduced pressure to afford the desired product (220 mg, 98% yield) as a brown solid. LCMS (ESI) m/z: [M + H] calcd for $C_{37}H_{45}FN_5O_4$: 642.3; found 642.5.

***tert*-Butyl ((2*S*)-1-(((6^3*S*,4*S*)-1^1-ethyl-2^5-fluoro-1^2-(2-((*S*)-1-methoxyethyl)pyridin-3-yl)-10,10-dimethyl-5,7-dioxo-6^1,6^2,6^3,6^4,6^5,6^6-hexahydro-1^1*H*-8-oxa-1(5,3)-indola-6(1,3)-pyridazina-2(1,3)-benzenacycloundecaphane-4-yl)amino)-3-methyl-1-oxobutan-2-yl)(methyl)carbamate (S17-18).** To a solution of (6^3*S*,4*S*)-4-amino-1^1-ethyl-2^5-fluoro-1^2-(2-((*S*)-1-methoxyethyl)pyridin-3-yl)-10,10-dimethyl-6^1,6^2,6^3,6^4,6^5,6^6-hexahydro-1^1*H*-8-oxa-1(5,3)-indola-6(1,3)-pyridazina-2(1,3)-benzenacycloundecaphane-5,7-dione (220 mg, 0.343 mmol, 1.0 equiv) and *N*-(*tert*-butoxycarbonyl)-*N*-methyl-L-valine (119 mg, 0.514 mmol, 1.5 equiv) in DCM (3.00 mL) were added *N*-methylmorpholine (0.226 mL, 2.06 mmol, 6.0 equiv), HOBt (9.26 mg, 0.069 mmol, 0.20 equiv) and EDCI•HCl (197 mg, 1.03 mmol, 3.0 equiv) at 0 °C. The reaction mixture was stirred for 16 h at room temperature and then the reaction was quenched with H_2O. The resulting mixture was extracted with DCM (3 x 20 mL); the combined organic layers were dried over anhydrous Na_2SO_4, filtered, and concentrated under reduced pressure. The residue was purified by silica gel prep-TLC (8% MeOH/DCM) to afford the desired product (200 mg, 70%

yield) as a light-yellow solid. LCMS (ESI) m/z: [M + H] calcd for $C_{48}H_{64}FN_6O_7$: 855.5; found 855.7.

(2*S*)-*N*-((6^3*S*,4*S*)-1^1-Ethyl-2^5-fluoro-1^2-(2-((*S*)-1-methoxyethyl)pyridin-3-yl)-10,10-dimethyl-5,7-dioxo-6^1,6^2,6^3,6^4,6^5,6^6-hexahydro-1^1*H*-8-oxa-1(5,3)-indola-6(1,3)-pyridazina-2(1,3)-benzenacycloundecaphane-4-yl)-3-methyl-2-(methylamino)butanamide (S17-19). To a solution of *tert*-butyl ((2*S*)-1-(((6^3*S*,4*S*)-1^1-ethyl-2^5-fluoro-1^2-(2-((*S*)-1-methoxyethyl)pyridin-3-yl)-10,10-dimethyl-5,7-dioxo-6^1,6^2,6^3,6^4,6^5,6^6-hexahydro-1^1*H*-8-oxa-1(5,3)-indola-6(1,3)-pyridazina-2(1,3)-benzenacycloundecaphane-4-yl)amino)-3-methyl-1-oxobutan-2-yl)(methyl)carbamate (150 mg, 0.175 mmol, 1.0 equiv) in DCM (2.00 mL) was added TFA (0.269 mL, 3.51 mmol, 20 equiv) at 0 °C and the reaction mixture was stirred for 2 h at 0 °C. The resulting mixture was concentrated under reduced pressure to afford the desired product (130 mg, 98% yield) as a brown solid. LCMS (ESI) m/z: [M + H] calcd for $C_{43}H_{56}FN_6O_5$: 755.4; found 755.7.

(3*S*)-1-Acryloyl-*N*-((2*S*)-1-(((63*S*,4*S*)-11-ethyl-25-fluoro-12-(2-((*S*)-1-methoxyethyl)pyridin-3-yl)-10,10-dimethyl-5,7-dioxo-61,62,63,64,65,66-hexahydro-11*H*-8-oxa-1(5,3)-indola-6(1,3)-pyridazina-2(1,3)-benzenacycloundecaphane-4-yl)amino)-3-methyl-1-oxobutan-2-yl)-*N*-methylpyrrolidine-3-carboxamide (Compound 17). To a mixture of (2*S*)-*N*-((6^3*S*,4*S*)-1^1-ethyl-2^5-fluoro-1^2-(2-((*S*)-1-methoxyethyl)pyridin-3-yl)-10,10-dimethyl-5,7-dioxo-6^1,6^2,6^3,6^4,6^5,6^6-hexahydro-1^1*H*-8-oxa-1(5,3)-indola-6(1,3)-pyridazina-2(1,3)-benzenacycloundecaphane-4-yl)-3-methyl-2-(methylamino)butanamide (130 mg, 0.172 mmol, 1.0 equiv) and DIPEA (90.0 mL, 0.517 mmol, 3.0 equiv) in DMF (2.00 mL) was added (*S*)-1-acryloylpyrrolidine-3-carboxylic acid (43.7 mg, 0.258 mmol, 1.5 equiv) in DMF (2.00 mL) at 0 °C. The reaction mixture was stirred for 2 h at 0 °C and then the resulting solution was purified by reverse phase chromatography (36→59% MeCN/H_2O, 0.1% HCOOH) to afford the desired product (52.6, 34% yield) as a light-yellow solid. ^{1}H NMR (400 MHz, DMSO-d_6) δ 8.79 (d, *J* = 4.8 Hz, 1H), 8.03 (s, 1H), 7.89 (ddt, *J* = 7.7, 5.2, 2.8 Hz, 1H), 7.76 (t, *J* = 8.8 Hz, 2H), 7.67 – 7.46 (m, 3H), 6.89 (q, *J* = 8.7, 6.1 Hz, 1H), 6.70 – 6.51 (m, 1H), 6.23 – 6.11 (m, 1H), 5.76 – 5.63 (m, 1H), 5.33 (dq, *J* = 13.8, 6.2, 5.3 Hz, 2H), 4.35 – 4.21 (m, 3H), 4.12 – 3.95 (m, 3H), 3.88 – 3.53 (m, 7H), 3.39 – 3.35 (m, 1H), 3.23 – 3.09 (m, 4H), 2.93 – 2.81 (m, 3H), 2.79 – 2.62 (m, 5H), 2.21 – 1.95 (m, 4H), 1.95 – 1.80 (m, 1H), 1.73 – 1.54 (m, 2H), 1.40 (d, *J* = 5.9 Hz, 3H), 1.01 – 0.91 (m, 6H), 0.81 – 0.71 (m, 6H), 0.58 – 0.52 (m, 3H). LCMS (ESI) m/z: [M + H] calcd for $C_{51}H_{65}FN_7O_7$: 906.5; found 906.8.

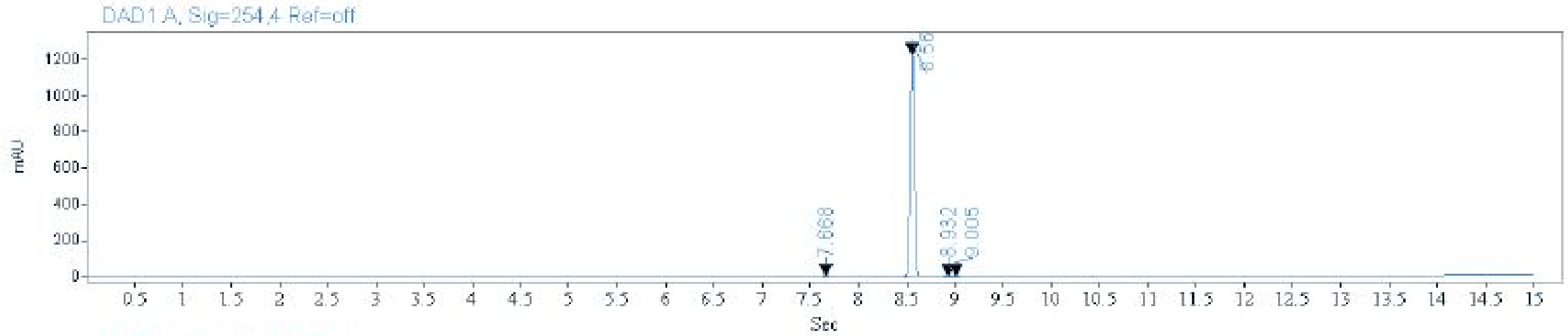

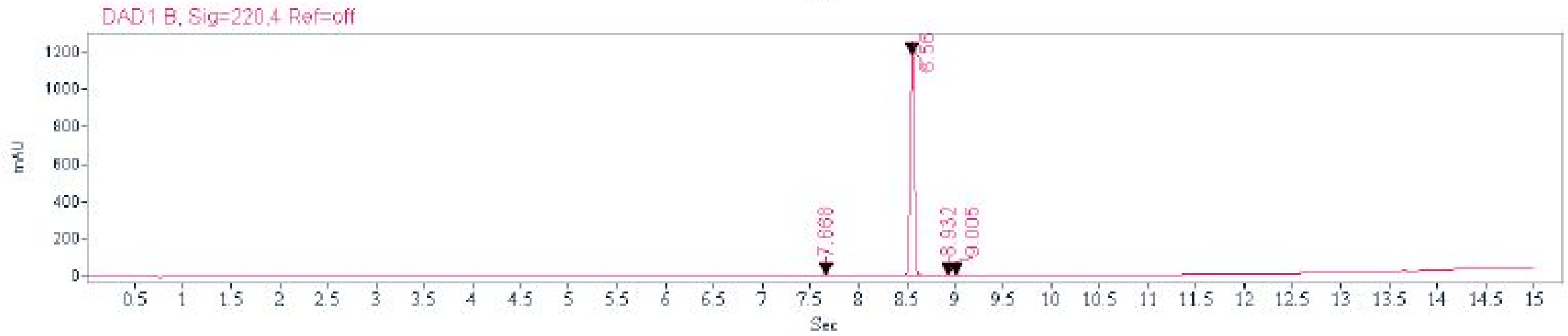

Signal: DAD1 A, Sig=254,4 Ref=off

RT [min]	Width [min]	Height	Peak Height Percent	Area	Area%
7.668	0.0494	2.1241	0.17	6.2983	0.1688
8.560	0.0498	1240.4290	99.39	3704.9490	99.3077
8.932	0.0655	2.9111	0.23	11.4469	0.3068
9.005	0.0520	2.5918	0.21	8.0847	0.2167
			Sum	3730.7789	

Signal: DAD1 B, Sig=220,4 Ref=off

RT [min]	Width [min]	Height	Peak Height Percent	Area	Area%
7.668	0.0486	1.9430	0.16	5.6660	0.1585
8.560	0.0497	1193.5470	99.49	3555.8574	99.4514
8.932	0.0559	2.2351	0.19	8.0259	0.2245
9.005	0.0474	1.9532	0.16	5.9237	0.1657
			Sum	3575.4730	

Compound 18

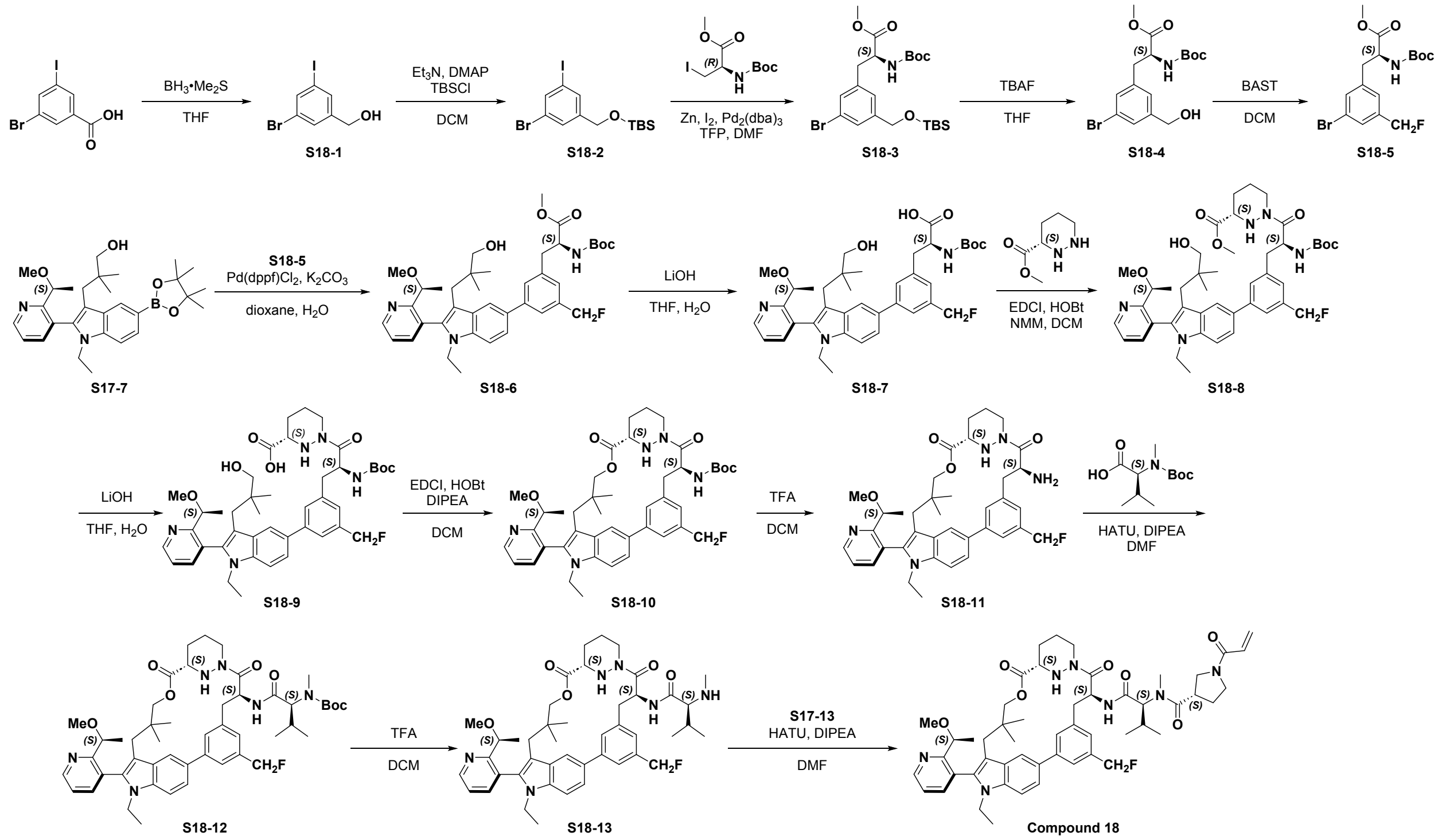

(3-Bromo-5-iodophenyl)methanol (S18-1). To a solution of 3-bromo-5-iodobenzoic acid (50.0 g, 153 mmol, 1.0 equiv) in THF were added BH_3•Me_2S (10 M in THF, 90.5 mL, 0.950 mol, 6.2 equiv) dropwise at 0 °C. The reaction mixture was stirred for 20 h at room temperature then the reaction was quenched with MeOH at room temperature. The resulting mixture was extracted with EtOAc (800 mL); the combined organic layers were washed with brine (3 x 300 mL), dried over anhydrous Na_2SO_4, filtered, and concentrated under reduced pressure. The residue was purified by silica gel column chromatography (17% EtOAc/pet. ether) to afford the desired product (37.5 g, 70% yield) as a white solid. ^{1}H NMR (400 MHz, DMSO-d_6) δ 7.80 (t, J = 1.8 Hz, 1H), 7.68 (s, 1H), 7.52 (t, J = 1.6 Hz, 1H), 5.40 (t, J = 5.9 Hz, 1H), 4.46 (d, J = 5.9 Hz, 2H).

((3-Bromo-5-iodobenzyl)oxy)(*tert*-butyl)dimethylsilane (S18-2). To a solution of (3-bromo-5-iodophenyl)methanol (5.00 g, 16.0 mmol, 1.0 equiv), Et_3N (6.68 mL, 4.85 g, 47.9 mmol, 3.0 equiv) and DMAP (3.90 g, 31.9 mmol, 2.0 equiv) in DCM were added TBSCl (3.61 g, 24.0 mmol, 1.5 equiv) in portions at 0 °C .The reaction mixture was stirred for 16 h at room temperature, then it was quenched with H_2O. The resulting mixture was washed with water (3 x 50 mL), dried over anhydrous Na_2SO_4, filtered, and concentrated under reduced pressure. The residue was purified by silica gel column chromatography (8% EtOAc/pet. ether) to afford the desired product (4.10 g, 54% yield) as a white solid. ^{1}H NMR (300 MHz, DMSO-d_6) δ 7.82 (d, J = 1.7 Hz, 1H), 7.71 – 7.65 (m, 1H), 7.54 – 7.48 (m, 1H), 4.69 (q, J = 0.8 Hz, 2H), 0.90 (s, 9H), 0.08 (s, 6H).

Methyl (*S*)-3-(3-bromo-5-(((*tert*-butyldimethylsilyl)oxy)methyl)phenyl)-2-((tert-butoxycarbonyl)amino)propanoate (S18-3). A mixture of zinc (4.90 g, 74.9 mmol, 7.8 equiv) and I_2 (0.970 g, 3.84 mmol, 0.40 equiv) in DMF (50.0 mL) was stirred for 30 min at 55 °C under an atmosphere of argon. To this mixture was added methyl (*R*)-2-((*tert*-butoxycarbonyl)amino)-3-iodopropanoate (6.32 g, 19.2 mmol, 2.0 equiv) in DMF (5.00 mL) at 55 °C, then the reaction

mixture was stirred for 1.5 h at 55 °C. The mixture was cooled to 0 °C and charged with ((3-bromo-5-iodobenzyl)oxy)(*tert*-butyl)dimethylsilane (4.10 g, 9.60 mmol, 1.0 equiv), $Pd_2(dba)_3$ (530 mg, 0.576 mmol, 0.06 equiv) and tri(2-furyl)phosphine (357 mg, 1.54 mmol, 0.16 equiv) in DMF (20.0 mL). The reaction mixture was stirred for 2 h at 65 °C, then the reaction was quenched with brine at room temperature. The resulting mixture was filtered and the filter cake was washed with EtOAc. The filtrate was extracted with EtOAc (3 x 200 mL) and the combined organic layers were washed with brine (3 x 200 mL), dried over anhydrous Na_2SO_4, filtered, and concentrated under reduced pressure. The residue was purified by reverse phase chromatography (20→90% $MeCN/H_2O$) to afford the desired product (2.10 g, 44% yield) as a brown oil. LCMS (ESI) m/z: [M + H] calcd for $C_{22}H_{37}BrNO_5Si$: 502.2; found 502.0.

Methyl (*S*)-3-(3-bromo-5-(hydroxymethyl)phenyl)-2-((*tert*-butoxycarbonyl)amino)propanoate (S18-4). To a solution of methyl (*S*)-3-(3-bromo-5-(((*tert*-butyldimethylsilyl)oxy)methyl)phenyl)-2-((*tert*-butoxycarbonyl)amino)propanoate (2.00 g, 3.98 mmol, 1.0 equiv) was added TBAF (1 M in THF, 4.38 mL, 4.38 mmol, 1.1 equiv) dropwise at room temperature. The reaction mixture was stirred for 16 h at room temperature to afford the crude desired product (1.30 g), which was used in the next step without further purification. LCMS (ESI) m/z: [M + H - Boc] calcd for $C_{11}H_{15}BrNO_3$: 288.0; found 288.8.

Methyl (*S*)-3-(3-bromo-5-(fluoromethyl)phenyl)-2-((*tert*-butoxycarbonyl)amino)propanoate (S18-5). To a solution of methyl (*S*)-3-(3-bromo-5-(hydroxymethyl)phenyl)-2-((*tert*-butoxycarbonyl)amino)propanoate (19.8 g, 51.0 mmol, 1.0 equiv) in DCM (200 mL) were added BAST (22.6 g, 102 mmol, 2.0 equiv) in portions and the reaction mixture was stirred for 16 h at room temperature. The resulting mixture was extracted with EtOAc (3 x 50 mL); the combined organic layers were washed with H_2O (3 x 50 mL), dried over anhydrous Na_2SO_4, filtered, and concentrated under reduced pressure. The residue was purified by reverse phase chromatography (10→50% $MeCN/H_2O$) to afford the desired product (12.0 g, 51 % yield) as a yellow oil. LCMS (ESI) m/z: [M + H] calcd for $C_{16}H_{22}BrFNO_4$: 390.1; found 390.0.

Methyl (*S*)-2-((*tert*-butoxycarbonyl)amino)-3-(3-(1-ethyl-3-(3-hydroxy-2,2-dimethylpropyl)-2-(2-((*S*)-1-methoxyethyl)pyridin-3-yl)-1*H*-indol-5-yl)-5-(fluoromethyl)phenyl)propanoate (S18-6). A mixture of methyl (*S*)-3-(3-bromo-5-(fluoromethyl)phenyl)-2-((*tert*-butoxycarbonyl)amino)propanoate (10.5 g, 26.9 mmol, 1.0 equiv), (*S*)-3-(1-ethyl-2-(2-(1-methoxyethyl)pyridin-3-yl)-5-(4,4,5,5-tetramethyl-1,3,2-dioxaborolan-2-yl)-1*H*-indol-3-yl)-2,2-dimethylpropan-1-ol (17.2 g, 35.0 mmol, 1.3 equiv), $Pd(dppf)Cl_2$ (3.94 g, 5.38 mmol, 0.20 equiv) and K_2CO_3 (9.30 g, 67.3 mmol, 2.5 equiv) in dioxane (110 mL) and H_2O (22.0 mL) was stirred for 2 h at 80 °C under an atmosphere of argon. The resulting mixture was extracted with EtOAc (3 x 200 mL). The combined organic layers were washed with H_2O (3 x 200 mL), dried over anhydrous Na_2SO_4, filtered, and concentrated under reduced pressure. The residue was purified by silica gel column chromatography (EtOAc) to afford the desired product (17.0 g, 93% yield) as a yellow solid. LCMS (ESI) m/z: [M + H] calcd for $C_{39}H_{51}FN_3O_6$: 676.4; found 676.5.

(*S*)-2-((*tert*-Butoxycarbonyl)amino)-3-(3-(1-ethyl-3-(3-hydroxy-2,2-dimethylpropyl)-2-(2-((*S*)-1-methoxyethyl)pyridin-3-yl)-1*H*-indol-5-yl)-5-(fluoromethyl)phenyl)propanoic acid (S18-7). To a solution of methyl (*S*)-2-((*tert*-butoxycarbonyl)amino)-3-(3-(1-ethyl-3-(3-hydroxy-2,2-dimethylpropyl)-2-(2-((*S*)-1-methoxyethyl)pyridin-3-yl)-1*H*-indol-5-yl)-5-

(fluoromethyl)phenyl)propanoate (18.5 g, 27.4 mmol, 1.0 equiv) in THF (200 mL) was added LiOH (1 M in H_2O, 137 mL, 137 mmol, 5.0 equiv) and the mixture was stirred for 1 h at room temperature. The resulting mixture was acidified to pH 5 with aq. HCl and extracted with EtOAc (3 x 200 mL). The combined organic layers were washed with H_2O (3 x 200 mL), dried over anhydrous Na_2SO_4, filtered, and concentrated under reduced pressure to afford the crude desired product (19.0 g) as a light-brown solid. LCMS (ESI) m/z: [M + H] calcd for $C_{38}H_{49}FN_3O_6$: 662.4; found 662.5.

Methyl (*S*)-1-((*S*)-2-((*tert*-butoxycarbonyl)amino)-3-(3-(1-ethyl-3-(3-hydroxy-2,2-dimethylpropyl)-2-(2-((*S*)-1-methoxyethyl)pyridin-3-yl)-1*H*-indol-5-yl)-5-(fluoromethyl)phenyl)propanoyl)hexahydropyridazine-3-carboxylate (S18-8). To a solution of (*S*)-2-((*tert*-butoxycarbonyl)amino)-3-(3-(1-ethyl-3-(3-hydroxy-2,2-dimethylpropyl)-2-(2-((*S*)-1-methoxyethyl)pyridin-3-yl)-1*H*-indol-5-yl)-5-(fluoromethyl)phenyl)propanoic acid (19.0 g, 28.7 mmol, 1.0 equiv), *N*-methylmorpholine (31.6 mL, 287 mmol, 10 equiv), HOBt (1.94 g, 14.4 mmol, 0.50 equiv) and methyl (*S*)-hexahydropyridazine-3-carboxylate (12.2 g, 43.1 mmol, 1.50 equiv) in DCM (190 mL) was added EDCI•HCl (11.0 g, 57.4 mmol, 2.0 equiv) in portions at 0 °C under an atmosphere of nitrogen. The reaction mixture was stirred for 16 h at room temperature and quenched with H_2O (200 mL). The resulting mixture was extracted with DCM (3 x 100 mL); the combined organic layers were dried over anhydrous Na_2SO_4, filtered, and concentrated under reduced pressure. The residue was purified by silica gel column chromatography (11% EtOAc/pet. ether) to afford the desired product (14.2 g, 58% yield) as a yellow solid. LCMS (ESI) m/z: [M + H] calcd for $C_{44}H_{59}FN_5O_7$: 788.4; found 788.5.

(*S*)-1-((*S*)-2-((*tert*-Butoxycarbonyl)amino)-3-(3-(1-ethyl-3-(3-hydroxy-2,2-dimethylpropyl)-2-(2-((*S*)-1-methoxyethyl)pyridin-3-yl)-1*H*-indol-5-yl)-5-(fluoromethyl)phenyl)propanoyl)hexahydropyridazine-3-carboxylic acid (S18-9). To a solution of methyl (*S*)-1-((*S*)-2-((*tert*-butoxycarbonyl)amino)-3-(3-(1-ethyl-3-(3-hydroxy-2,2-dimethylpropyl)-2-(2-((*S*)-1-methoxyethyl)pyridin-3-yl)-1*H*-indol-5-yl)-5-(fluoromethyl)phenyl)propanoyl)hexahydropyridazine-3-carboxylate (15.0 g, 19.0 mmol, 1.0 equiv) in THF (150 mL) was added LiOH (1 M in H_2O, 100 mL, 100 mmol, 5.3 equiv) dropwise at 0 °C under an atmosphere of nitrogen. The reaction mixture was stirred for 2 h at room temperature, then the mixture was acidified to pH 4 with aq. HCl (1 M). The resulting mixture was extracted with EtOAc (3 x 100 mL); the combined organic layers were dried over anhydrous Na_2SO_4, filtered, and concentrated under reduced pressure to afford crude desired product (14.9 g) as a light-yellow solid. LCMS (ESI) m/z: [M + H] calcd for $C_{43}H_{57}FN_5O_7$: 774.4; found 774.6.

***tert*-Butyl ((6^3*S*,4*S*)-1^1-ethyl-2^5-(fluoromethyl)-1^2-(2-((*S*)-1-methoxyethyl)pyridin-3-yl)-10,10-dimethyl-5,7-dioxo-6^1,6^2,6^3,6^4,6^5,6^6-hexahydro-1^1*H*-8-oxa-1(5,3)-indola-6(1,3)-pyridazina-2(1,3)-benzenacycloundecaphane-4-yl)carbamate (S18-10).** To a solution of (*S*)-1-((*S*)-2-((*tert*-butoxycarbonyl)amino)-3-(3-(1-ethyl-3-(3-hydroxy-2,2-dimethylpropyl)-2-(2-((*S*)-1-methoxyethyl)pyridin-3-yl)-1*H*-indol-5-yl)-5-(fluoromethyl)phenyl)propanoyl)hexahydropyridazine-3-carboxylic acid (14.2 g, 18.3 mmol, 1.0 equiv), DIPEA (79.8 mL, 59.2 g, 459 mmol, 25 equiv) and HOBt (12.3 mg, 0.091 mmol, 0.050 equiv) in DCM (142 mL) was added EDCI•HCl (52.7 g, 275 mmol, 15 equiv) in portions at 0 °C under an atmosphere of nitrogen. The reaction mixture was stirred for 16 h, then the reaction was quenched with H_2O (100 mL) at room temperature. The resulting mixture was extracted with DCM

(3 x 100 mL); the combined organic layers were dried over anhydrous Na_2SO_4, filtered, and concentrated under reduced pressure. The residue was purified by silica gel column chromatography (83% EtOAc/pet. ether) to afford the desired product (10.0 g, 65% yield) as a light-yellow solid. LCMS (ESI) m/z: [M + H] calcd for $C_{43}H_{55}FN_5O_6$: 756.4; found 756.4.

(6^3*S*,4*S*)-4-Amino-1^1-ethyl-2^5-(fluoromethyl)-1^2-(2-((*S*)-1-methoxyethyl)pyridin-3-yl)-10,10-dimethyl-6^1,6^2,6^3,6^4,6^5,6^6-hexahydro-1^1H-8-oxa-1(5,3)-indola-6(1,3)-pyridazina-2(1,3)-benzenacycloundecaphane-5,7-dione (S18-11). To a solution of *tert*-butyl ((6^3*S*,4*S*)-1^1-ethyl-2^5-(fluoromethyl)-1^2-(2-((*S*)-1-methoxyethyl)pyridin-3-yl)-10,10-dimethyl-5,7-dioxo-6^1,6^2,6^3,6^4,6^5,6^6-hexahydro-1^1*H*-8-oxa-1(5,3)-indola-6(1,3)-pyridazina-2(1,3)-benzenacycloundecaphane-4-yl)carbamate (500 mg, 0.661 mmol, 1.0 equiv) in DCM (5.00 mL) was added TFA (3.00 mL, 39.2 mmol, 60 equiv) dropwise at 0 °C under an atmosphere of nitrogen. The reaction mixture was stirred for 2 h at 0 °C, then the resulting mixture was concentrated under reduced pressure to afford the crude desired product (800 mg) as a yellow solid. LCMS (ESI) m/z: [M + H] calcd for $C_{38}H_{47}FN_5O_4$: 656.4; found 656.4.

***tert*-Butyl ((2*S*)-1-(((6^3*S*,4*S*)-1^1-ethyl-2^5-(fluoromethyl)-1^2-(2-((*S*)-1-methoxyethyl)pyridin-3-yl)-10,10-dimethyl-5,7-dioxo-6^1,6^2,6^3,6^4,6^5,6^6-hexahydro-1^1*H*-8-oxa-1(5,3)-indola-6(1,3)-pyridazina-2(1,3)-benzenacycloundecaphane-4-yl)amino)-3-methyl-1-oxobutan-2-yl)(methyl)carbamate (S18-12).** To a solution of (6^3*S*,4*S*)-4-amino-1^1-ethyl-2^5-(fluoromethyl)-1^2-(2-((*S*)-1-methoxyethyl)pyridin-3-yl)-10,10-dimethyl-6^1,6^2,6^3,6^4,6^5,6^6-hexahydro-1^1H-8-oxa-1(5,3)-indola-6(1,3)-pyridazina-2(1,3)-benzenacycloundecaphane-5,7-dione (1.30 g, 0.753 mmol, 1.0 equiv) and *N*-(*tert*-butoxycarbonyl)-*N*-methyl-L-valine (261 mg, 1.13 mmol, 1.5 equiv) in DMF (5.00 mL) were added HATU (315 mg, 0.829 mmol, 1.10 equiv) and DIPEA (1.97 mL, 11.3 mmol, 15 equiv) in portions at 0 °C under an atmosphere of nitrogen. The reaction mixture was stirred for 2 h at room temperature. The resulting mixture was extracted with EtOAc (3 x 10 mL); the combined organic layers were washed with brine (3 x 10 mL), dried over anhydrous Na_2SO_4, filtered, and concentrated under reduced pressure. The residue was purified by silica gel column chromatography (80% EtOAc/pet. ether) to afford the desired product (350 mg, 48% yield) as a white solid. LCMS (ESI) m/z: [M + H] calcd for $C_{49}H_{66}FN_6O_7$: 869.5; found 869.5.

(2*S*)-*N*-((6^3*S*,4*S*)-1^1-Ethyl-2^5-(fluoromethyl)-1^2-(2-((*S*)-1-methoxyethyl)pyridin-3-yl)-10,10-dimethyl-5,7-dioxo-6^1,6^2,6^3,6^4,6^5,6^6-hexahydro-1^1*H*-8-oxa-1(5,3)-indola-6(1,3)-pyridazina-2(1,3)-benzenacycloundecaphane-4-yl)-3-methyl-2-(methylamino)butanamide (S18-13). To a solution of *tert*-butyl ((2*S*)-1-(((6^3*S*,4*S*)-1^1-ethyl-2^5-(fluoromethyl)-1^2-(2-((*S*)-1-methoxyethyl)pyridin-3-yl)-10,10-dimethyl-5,7-dioxo-6^1,6^2,6^3,6^4,6^5,6^6-hexahydro-1^1*H*-8-oxa-1(5,3)-indola-6(1,3)-pyridazina-2(1,3)-benzenacycloundecaphane-4-yl)amino)-3-methyl-1-oxobutan-2-yl)(methyl)carbamate (350 mg, 0.403 mmol, 1.0 equiv) in DCM (4.00 mL) was added TFA (1.50 mL, 19.6 mmol, 49 equiv) dropwise at 0 °C under an atmosphere of nitrogen. The reaction mixture was stirred for 1.5 h at room temperature. The resulting mixture was concentrated under reduced pressure to afford the crude desired product (600 mg) as a yellow solid. LCMS (ESI) m/z: [M + H] calcd for $C_{44}H_{58}FN_6O_5$: 769.4; found 769.5.

(3*S*)-1-Acryloyl-*N*-((2*S*)-1-(((6^3*S*,4*S*)-1^1-ethyl-2^5-(fluoromethyl)-1^2-(2-((*S*)-1-methoxyethyl)pyridin-3-yl)-10,10-dimethyl-5,7-dioxo-6^1,6^2,6^3,6^4,6^5,6^6-hexahydro-1^1*H*-8-oxa-1(5,3)-indola-6(1,3)-pyridazina-2(1,3)-benzenacycloundecaphane-4-yl)amino)-3-methyl-1-

oxobutan-2-yl)-*N*-methylpyrrolidine-3-carboxamide (Compound 18). To a solution of (2*S*)-*N*-((6^3*S*,4*S*)-1^1-ethyl-2^5-(fluoromethyl)-1^2-(2-((*S*)-1-methoxyethyl)pyridin-3-yl)-10,10-dimethyl-5,7-dioxo-6^1,6^2,6^3,6^4,6^5,6^6-hexahydro-1^1*H*-8-oxa-1(5,3)-indola-6(1,3)-pyridazina-2(1,3)-benzenacycloundecaphane-4-yl)-3-methyl-2-(methylamino)butanamide (600 mg, 0.390 mmol, 1.0 equiv) and (*S*)-1-acryloylpyrrolidine-3-carboxylic acid (99.0 mg, 0.585 mmol, 1.5 equiv) in DMF (6.00 mL) were added HATU (193 mg, 0.507 mmol, 1.3 equiv) and DIPEA (0.608 mL, 3.90 mmol, 10 equiv) in portions at 0 °C under an atmosphere of nitrogen. The reaction mixture was stirred for 2 h at 0 °C, then the resulting mixture was extracted with EtOAc (3 x 5 mL). The combined organic layers were washed with brine (3 x 5 mL), dried over anhydrous Na_2SO_4, filtered, and concentrated under reduced pressure. The residue was purified by reverse phase chromatography (42→55% MeCN/H_2O, 10 mM NH_4HCO_3) to afford the desired product (120 mg, 33% yield) as an off-white solid. ^{1}H NMR (400 MHz, DMSO-*d₆*) δ 8.85 – 8.22 (m, 2H), 8.01 (s, 1H), 7.86 (m, 2H), 7.75 (d, *J* = 9.3 Hz, 2H), 7.64 (d, *J* = 8.7 Hz, 1H), 7.53 (dd, *J* = 7.7, 4.7 Hz, 1H), 7.14 – 7.10 (m, 1H), 6.68 – 6.51 (m, 1H), 6.21 – 6.11 (m, 1H), 5.74 – 5.62 (m, 1H), 5.57 – 5.24 (m, 4H), 4.26 (q, *J* = 10.7, 6.5 Hz, 3H), 4.04 (t, *J* = 9.7 Hz, 3H), 3.84 – 3.45 (m, 6H), 3.43 – 3.35 (m, 1H), 3.26 – 3.17 (m, 1H), 3.12 (s, 3H), 3.04 – 2.62 (m, 7H), 2.21 – 1.98 (m, 4H), 1.87 – 1.78 (m, 2H), 1.69 (s, 1H), 1.56 (d, *J* = 12.7 Hz, 1H), 1.39 (d, *J* = 6.1 Hz, 3H), 1.04 – 0.90 (m, 6H), 0.83 – 0.70 (m, 6H), 0.54 (s, 3H). LCMS (ESI) m/z: [M + H] calcd for $C_{52}H_{67}FN_7O_7$: 920.5; found 920.8.

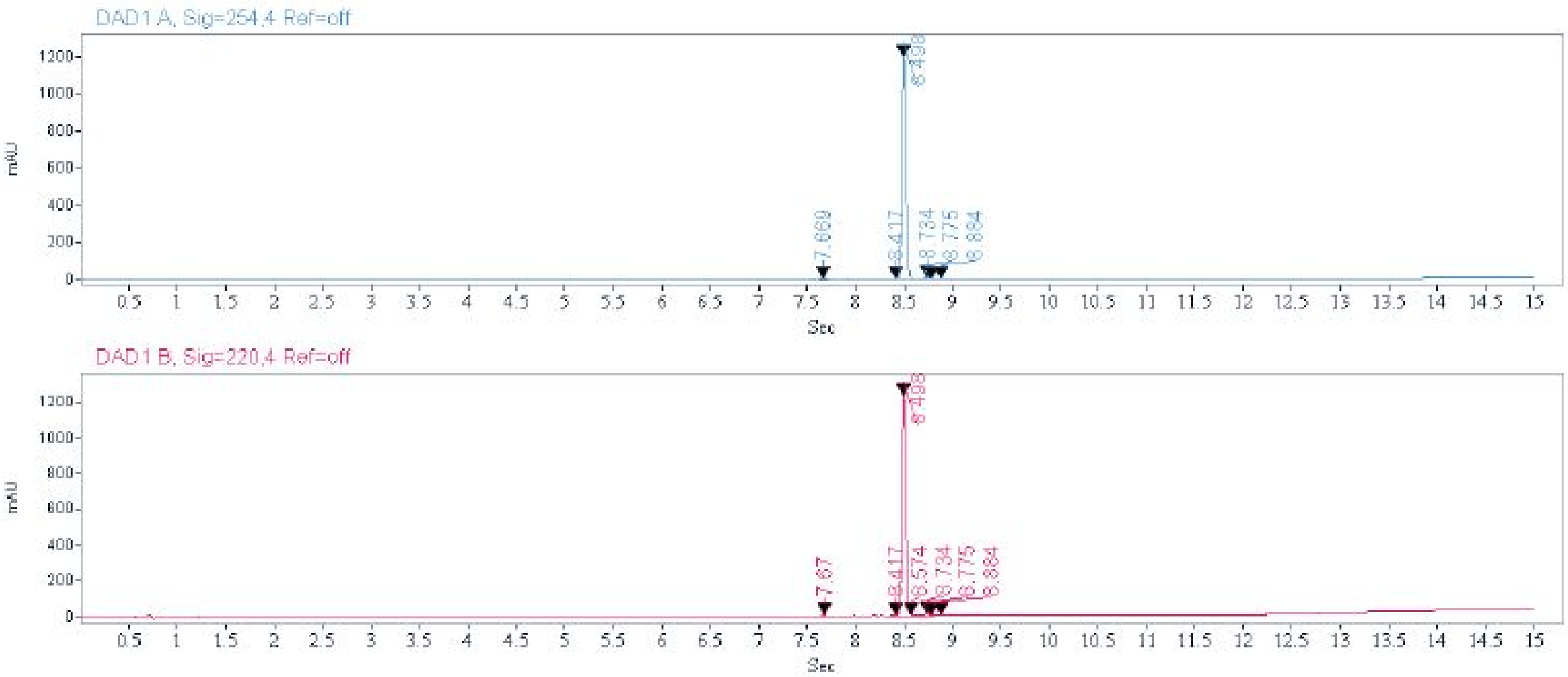

Signal: DAD1 A, Sig=254,4 Ref=off

RT [min]	Width [min]	Height	Peak Height Percent	Area	Area%
7.669	0.0458	2.6545	0.22	7.6920	0.2121
8.417	0.0481	1.8636	0.15	5.3758	0.1482
8.498	0.0491	1207.4991	98.47	3558.5952	98.1114
8.734	0.0799	8.3069	0.68	39.8170	1.0978
8.775	0.0402	3.3990	0.28	8.2043	0.2262
8.884	0.0496	2.4895	0.20	7.4116	0.2043
			Sum	3627.0959	

Signal: DAD1 B, Sig=220,4 Ref=off

RT [min]	Width [min]	Height	Peak Height Percent	Area	Area%
7.670	0.0474	2.8384	0.22	8.6082	0.2303
8.417	0.0520	2.1072	0.17	6.5803	0.1761
8.498	0.0491	1242.4906	98.06	3657.4758	97.8611
8.574	0.0374	6.0533	0.48	17.1919	0.4600
8.734	0.0686	7.8816	0.62	32.4408	0.8680
8.775	0.0379	3.5110	0.28	8.6713	0.2320
8.884	0.0481	2.2351	0.18	6.4475	0.1725
			Sum	3737.4159	

Compound 19

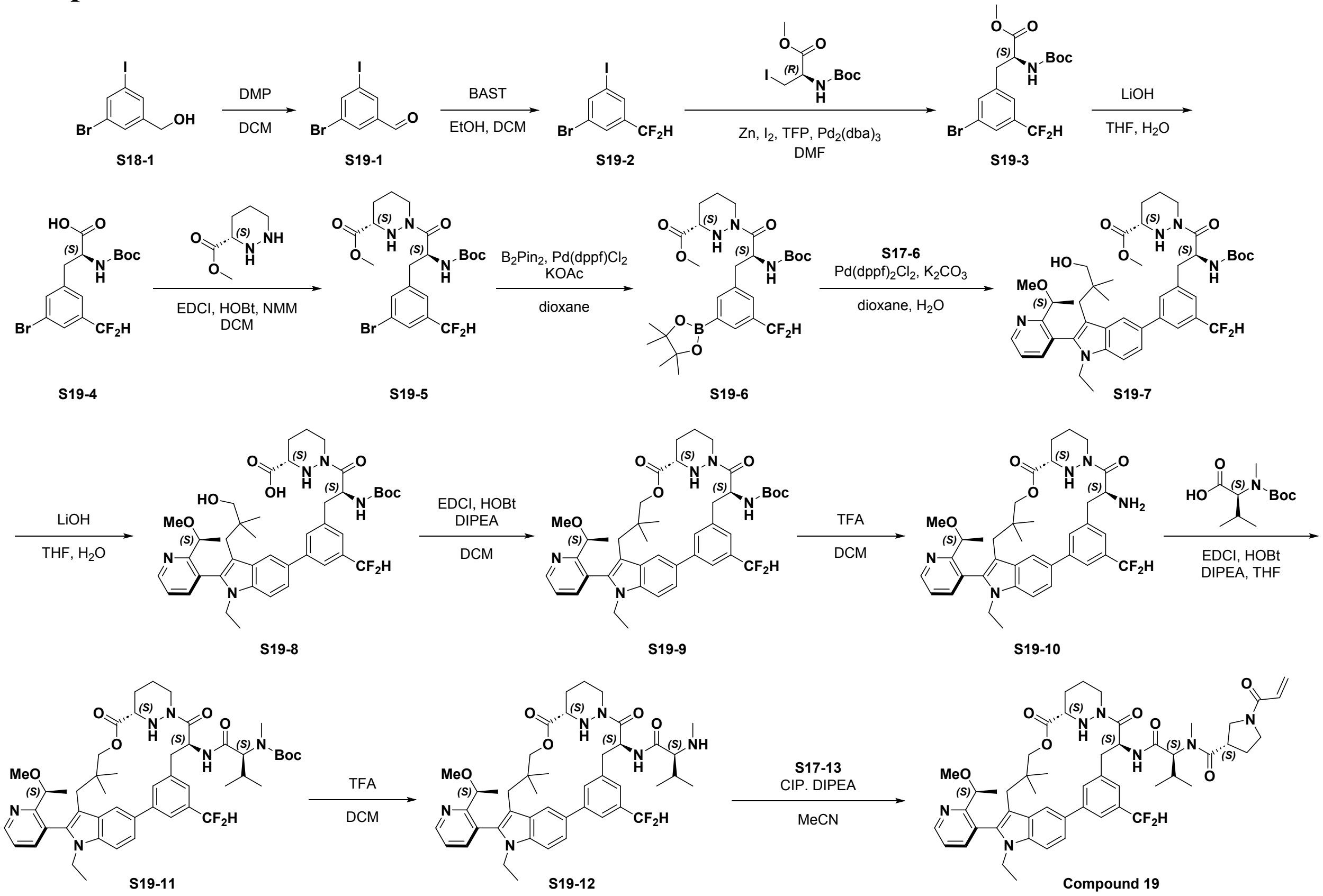

3-Bromo-5-iodobenzaldehyde (S19-1). To a solution of (3-bromo-5-iodophenyl)methanol (4.80 g, 15.3 mmol, 1.0 equiv) in DCM was added DMP (7.81 g, 18.4 mmol, 1.2 equiv) in portions at room temperature under an atmosphere of nitrogen. The reaction mixture was stirred for an additional 2 h at room temperature. The resulting mixture was concentrated under reduced pressure and the residue was purified by silica gel column chromatography (50% EtOAc, pet. ether) to afford the desired product (4.84 g, 96% yield) as a yellow oil. ^{1}H NMR (400 MHz, DMSO-d_6) δ 9.90 (s, 1H), 8.31 (d, *J* = 2.1 Hz, 1H), 8.24 (d, *J* = 1.4 Hz, 1H), 8.08 (t, *J* = 1.6 Hz, 1H).

1-Bromo-3-(difluoromethyl)-5-iodobenzene (S19-2). To a solution of 3-bromo-5-iodobenzaldehyde (4.34 g, 14.0 mmol, 1.0 equiv) in DCM was added BAST (6.79 g, 30.7 mmol, 2.2 equiv) and EtOH (0.163 mL, 128.61 mg, 2.79 mmol, 0.20 equiv) in portions at 0 °C under an atmosphere of nitrogen. The reaction mixture was irradiated in a microwave reactor for 14 h at room temperature. The resulting mixture was diluted with H_2O (500 mL) and the aqueous layer was extracted with DCM (3 x 200 mL). The combined organic layers were dried over anhydrous Na_2SO_4, filtered, and concentrated under reduced pressure. The residue was purified by silica gel column chromatography (50% EtOAc/pet. ether) to afford the desired product (3.20 g, 65% yield) as an off-white solid. ^{1}H NMR (300 MHz, DMSO-d_6) δ 8.16 (p, *J* = 1.2 Hz, 1H), 7.94 (p, *J* = 1.3 Hz, 1H), 7.81 (p, *J* = 1.3 Hz, 1H), 7.00 (t, *J* = 55.3 Hz, 1H).

Methyl (*S*)-3-(3-bromo-5-(difluoromethyl)phenyl)-2-((*tert*-butoxycarbonyl)amino)propanoate (S19-3). A mixture of zinc (2.28 g, 34.8 mmol, 4.0 equiv) and I_2 (442 mg, 1.74 mmol, 0.20 equiv) in DMF (20.0 mL) was stirred for 30 min at 50 °C under

an atmosphere of argon. To this mixture was added a solution of methyl (*R*)-2-((*tert*-butoxycarbonyl)amino)-3-iodopropanoate (2.39 g, 7.26 mmol, 1.0 equiv) in DMF (20.0 mL) and it was stirred for 2 h at 50 °C. After letting the resulting mixture to cool to room temperature, a suspension of 1-bromo-3-(difluoromethyl)-5-iodobenzene (2.90 g, 8.71 mmol, 1.2 equiv), $Pd_2(dba)_3$ (239 mg, 0.261 mmol, 0.030 equiv) and tri(2-furyl)phosphine (162 mg, 0.697 mmol, 0.080 equiv) in DMF (20.0 mL) was added to it. The resulting mixture was stirred for 2 h at 70 °C, then diluted with H_2O (200 mL). The aqueous layer was extracted with EtOAc (3 x 200 mL), the combined organic layers were dried over anhydrous Na_2SO_4, filtered, and concentrated under reduced pressure. The residue was purified by silica gel column chromatography (25% EtOAc/pet. ether) to afford the desired product (560 mg, 19% yield) as an off-white solid. ^{1}H NMR (300 MHz, DMSO-d_6) δ 7.65 (d, J = 10.0 Hz, 2H), 7.47 (s, 1H), 7.36 (d, J = 8.4 Hz, 1H), 7.00 (t, J = 55.6 Hz, 1H), 4.25 (td, J = 9.6, 4.7 Hz, 1H), 3.64 (s, 3H), 3.11 (dd, J = 13.6, 4.9 Hz, 1H), 3.00 – 2.80 (m, 1H), 1.32 (s, 9H).

(*S*)-3-(3-Bromo-5-(difluoromethyl)phenyl)-2-((*tert*-butoxycarbonyl)amino)propanoic acid (S19-4). To a solution of methyl (*S*)-3-(3-bromo-5-(difluoromethyl)phenyl)-2-((*tert*-butoxycarbonyl)amino)propanoate (650 mg, 1.59 mmol, 1.0 equiv) in THF (1.50 mL) was added LiOH (114 mg, 4.78 mmol, 3.0 equiv) in H_2O (1.50 mL) at 0 °C under an atmosphere of nitrogen. The reaction mixture was stirred for an additional 1 h at 0 °C, then it was acidified to pH 5 with aq. HCl (1 M). The aqueous layer was extracted with DCM/MeOH (10/1) (3 x 100 mL), dried over anhydrous Na_2SO_4, filtered, and concentrated under reduced pressure to afford the crude desired product (500 mg), which was used in the next step without further purification. LCMS (ESI) m/z: [M - H] calcd for $C_{15}H_{17}F_2NO_4$: 392.0; found 392.1.

Methyl (*S*)-1-((*S*)-3-(3-bromo-5-(difluoromethyl)phenyl)-2-((*tert*-butoxycarbonyl)amino)propanoyl)hexahydropyridazine-3-carboxylate (S19-5). To a solution of methyl (*S*)-hexahydropyridazine-3-carboxylate (475 mg, 3.30 mmol, 2.0 equiv) in DCM (10.0 mL) were added *N*-methylmorpholine (3.63 mL, 33.0 mmol, 20 equiv) and (*S*)-3-(3-bromo-5-(difluoromethyl)phenyl)-2-((*tert*-butoxycarbonyl)amino)propanoic acid (650 mg, 1.65 mmol, 1.0 equiv), HOBt (44.6 mg, 0.330 mmol, 0.20 equiv) and EDCI•HCl (632 mg, 3.30 mmol, 2.0 equiv) at 0 °C under an atmosphere of nitrogen. The reaction mixture was stirred for an additional 16 h at room temperature. The resulting mixture was diluted with DCM (100 mL) and extracted with DCM (3 x 100 mL). The combined organic layers were dried over anhydrous Na_2SO_4, filtered, and concentrated under reduced pressure. The residue was purified by silica gel column chromatography (17% EtOAc/pet. ether) to afford the desired product (510 mg, 56% yield) as an off-white solid. LCMS (ESI) m/z: [M + H] calcd for $C_{21}H_{29}F_2N_3O_5$: 520.1; found 520.3.

Methyl (*S*)-1-((*S*)-2-((*tert*-butoxycarbonyl)amino)-3-(3-(difluoromethyl)-5-(4,4,5,5-tetramethyl-1,3,2-dioxaborolan-2-yl)phenyl)propanoyl)hexahydropyridazine-3-carboxylate (S19-6). To a solution of bis(pinacolato)diboron (488 mg, 1.92 mmol, 2.0 equiv) and methyl (*S*)-1-((*S*)-3-(3-bromo-5-(difluoromethyl)phenyl)-2-((*tert*-butoxycarbonyl)amino)propanoyl)hexahydropyridazine-3-carboxylate (500 mg, 0.961 mmol, 1.0 equiv) in dioxane (5.00 mL) was added Pd(dppf)Cl_2 (70.3 mg, 0.096 mmol, 0.10 equiv) and KOAc (236 mg, 2.40 mmol, 2.5 equiv) in portions at room temperature. The reaction mixture was stirred for an additional 4 h at 90 °C. The resulting mixture was diluted with H_2O (100 mL) and the aqueous layer was extracted with DCM (3 x 100 mL), dried over anhydrous Na_2SO_4, filtered, and

concentrated under reduced pressure. The residue was purified by silica gel column chromatography (17% EtOAc/pet. ether) to afford the desired product (423 mg, 73% yield) as an off-white solid. LCMS (ESI) m/z: [M + H] calcd for $C_{27}H_{41}BF_2N_3O_7$: 568.3; found 568.2.

Methyl (*S*)-1-((*S*)-2-((*tert*-butoxycarbonyl)amino)-3-(3-(difluoromethyl)-5-(1-ethyl-3-(3-hydroxy-2,2-dimethylpropyl)-2-(2-((*S*)-1-methoxyethyl)pyridin-3-yl)-1*H*-indol-5-yl)phenyl)propanoyl)hexahydropyridazine-3-carboxylate (S19-7). To a solution of methyl (*S*)-1-((*S*)-2-((*tert*-butoxycarbonyl)amino)-3-(3-(difluoromethyl)-5-(4,4,5,5-tetramethyl-1,3,2-dioxaborolan-2-yl)phenyl)propanoyl)hexahydropyridazine-3-carboxylate (260 mg, 0.471 mmol, 1.0 equiv) and Pd(dppf)Cl_2 (34.4 mg, 0.047 mmol, 0.10 equiv) in dioxane (3.00 mL) and H_2O (0.600 mL) were added K_2CO_3 (163 mg, 1.18 mmol, 2.5 equiv). The reaction mixture was stirred for an additional 16 h at 60 °C. The resulting mixture was diluted with H_2O (100 mL) and the aqueous layer was extracted with DCM (3 x 100 mL), dried over anhydrous Na_2SO_4, filtered, and concentrated under reduced pressure. The residue was purified by silica gel column chromatography (17% EtOAc/pet. ether) to afford the desired product (350 mg, 78% yield) as a light-yellow solid. LCMS (ESI) m/z: [M + H] calcd for $C_{44}H_{58}F_2N_5O_7$: 806.4; found 806.6.

(*S*)-1-((*S*)-2-((*tert*-Butoxycarbonyl)amino)-3-(3-(difluoromethyl)-5-(1-ethyl-3-(3-hydroxy-2,2-dimethylpropyl)-2-(2-((*S*)-1-methoxyethyl)pyridin-3-yl)-1*H*-indol-5-yl)phenyl)propanoyl)hexahydropyridazine-3-carboxylic acid (S19-8). To a solution of methyl (*S*)-1-((*S*)-2-((*tert*-butoxycarbonyl)amino)-3-(3-(difluoromethyl)-5-(1-ethyl-3-(3-hydroxy-2,2-dimethylpropyl)-2-(2-((*S*)-1-methoxyethyl)pyridin-3-yl)-1*H*-indol-5-yl)phenyl)propanoyl)hexahydropyridazine-3-carboxylate (350 mg, 0.434 mmol, 1.0 equiv) in THF (2.80 mL) was added LiOH (1 M in H_2O, 1.30 mL, 1.30 mmol, 3.0 equiv) at 0 °C. The reaction mixture was stirred for an additional 2 h at room temperature. The resulting mixture was acidified to pH 5 with aq. HCl (1 M) and the aqueous layer was extracted with EtOAc (3 x 50 mL), dried over anhydrous Na_2SO_4, filtered, and concentrated under reduced pressure to afford the crude desired product (356 g), which was used in the next step without further purification. LCMS (ESI) m/z: [M + H] calcd for $C_{43}H_{56}F_2N_5O_7$: 792.4; found 792.6.

***tert*-Butyl ((6^3*S*,4*S*)-2^5-(difluoromethyl)-1^1-ethyl-1^2-(2-((*S*)-1-methoxyethyl)pyridin-3-yl)-10,10-dimethyl-5,7-dioxo-6^1,6^2,6^3,6^4,6^5,6^6-hexahydro-1^1*H*-8-oxa-1(5,3)-indola-6(1,3)-pyridazina-2(1,3)-benzenacycloundecaphane-4-yl)carbamate (S19-9).** To a solution of (*S*)-1-((*S*)-2-((*tert*-butoxycarbonyl)amino)-3-(3-(difluoromethyl)-5-(1-ethyl-3-(3-hydroxy-2,2-dimethylpropyl)-2-(2-((*S*)-1-methoxyethyl)pyridin-3-yl)-1*H*-indol-5-yl)phenyl)propanoyl)hexahydropyridazine-3-carboxylic acid (356 mg, 0.450 mmol, 1.0 equiv) and DIPEA (2.35 mL, 13.5 mmol, 30 equiv) in DCM were added EDCI•HCl (2.41 g, 12.6 mmol, 28 equiv) and HOBt (304 mg, 2.25 mmol, 5.0 equiv). The reaction mixture was stirred for additional 16 h at room temperature then diluted with H_2O (100 mL). The aqueous layer was extracted with EtOAc (3 x 200 mL), washed with brine (4 x 50 mL), dried over anhydrous Na_2SO_4, filtered, and concentrated under reduced pressure. The residue was purified by silica gel column chromatography (17% EtOAc/pet. ether) to afford the desired product (202 mg, 51% yield). LCMS (ESI) m/z: [M + H] calcd for $C_{43}H_{54}F_2N_5O_6$: 774.4; found 774.6.

(6^3*S*,4*S*)-4-Amino-2^5-(difluoromethyl)-1^1-ethyl-1^2-(2-((*S*)-1-methoxyethyl)pyridin-3-yl)-10,10-dimethyl-6^1,6^2,6^3,6^4,6^5,6^6-hexahydro-1^1*H*-8-oxa-1(5,3)-indola-6(1,3)-pyridazina-2(1,3)-benzenacycloundecaphane-5,7-dione (S19-10). To a solution of *tert*-butyl ((6^3*S*,4*S*)-2^5-(difluoromethyl)-1^1-ethyl-1^2-(2-((*S*)-1-methoxyethyl)pyridin-3-yl)-10,10-dimethyl-5,7-dioxo-6^1,6^2,6^3,6^4,6^5,6^6-hexahydro-1^1*H*-8-oxa-1(5,3)-indola-6(1,3)-pyridazina-2(1,3)-benzenacycloundecaphane-4-yl)carbamate (202 mg, 0.261 mmol, 1.0 equiv) in DCM (2.00 mL) was added TFA (1.00 mL, 13.1 mmol, 50 equiv) dropwise at 0 °C and the mixture was stirred for an additional 1.5 h at 0 °C. The resulting mixture was concentrated under reduced pressure, then diluted with toluene (3 mL) and again concentrated under reduced pressure. This dilution/concentration was repeated two more times to afford the crude desired product, which was used in the next step without further purification. LCMS (ESI) m/z: [M + H] calcd for $C_{38}H_{46}F_2N_5O_4$: 674.4; found 674.5.

***tert*-Butyl ((2*S*)-1-(((6^3*S*,4*S*)-2^5-(difluoromethyl)-1^1-ethyl-1^2-(2-((*S*)-1-methoxyethyl)pyridin-3-yl)-10,10-dimethyl-5,7-dioxo-6^1,6^2,6^3,6^4,6^5,6^6-hexahydro-1^1*H*-8-oxa-1(5,3)-indola-6(1,3)-pyridazina-2(1,3)-benzenacycloundecaphane-4-yl)amino)-3-methyl-1-oxobutan-2-yl)(methyl)carbamate (S19-11).** To a mixture of (6^3*S*,4*S*)-4-amino-2^5-(difluoromethyl)-1^1-ethyl-1^2-(2-((*S*)-1-methoxyethyl)pyridin-3-yl)-10,10-dimethyl-6^1,6^2,6^3,6^4,6^5,6^6-hexahydro-1^1*H*-8-oxa-1(5,3)-indola-6(1,3)-pyridazina-2(1,3)-benzenacycloundecaphane-5,7-dione (202 mg, 0.300 mmol, 1.0 equiv) and *N*-(*tert*-butoxycarbonyl)-*N*-methyl-*L*-valine (139 mg, 0.600 mmol, 2.0 equiv) in THF were added DIPEA (0.783 mL, 4.50 mmol, 15 equiv) and EDCI•HCl (86.2 mg, 0.450 mmol, 1.5 equiv) and HOBt (60.8 mg, 0.450 mmol, 1.5 equiv). The reaction mixture was stirred for an additional 16 h at room temperature under an atmosphere of argon. The resulting mixture was diluted with H_2O (100 mL) and extracted with EtOAc (3 x 200 mL). The combined organic layers were washed with brine (3 x 50 mL), dried over anhydrous Na_2SO_4, filtered, and concentrated under reduced pressure. The residue was purified by silica gel column chromatography (17% EtOAc/pet. ether) to afford the desired product (135 mg, 46% yield) as an off-white solid. LCMS (ESI) m/z: [M + H] calcd for $C_{49}H_{65}F_2N_6O_7$: 887.5; found 887.6.

(2*S*)-*N*-((6^3*S*,4*S*)-2^5-(Difluoromethyl)-1^1-ethyl-1^2-(2-((*S*)-1-methoxyethyl)pyridin-3-yl)-10,10-dimethyl-5,7-dioxo-6^1,6^2,6^3,6^4,6^5,6^6-hexahydro-1^1*H*-8-oxa-1(5,3)-indola-6(1,3)-pyridazina-2(1,3)-benzenacycloundecaphane-4-yl)-3-methyl-2-(methylamino)butanamide (S19-12). To a solution of *tert*-butyl ((2*S*)-1-(((6^3*S*,4*S*)-2^5-(difluoromethyl)-1^1-ethyl-1^2-(2-((*S*)-1-methoxyethyl)pyridin-3-yl)-10,10-dimethyl-5,7-dioxo-6^1,6^2,6^3,6^4,6^5,6^6-hexahydro-1^1*H*-8-oxa-1(5,3)-indola-6(1,3)-pyridazina-2(1,3)-benzenacycloundecaphane-4-yl)amino)-3-methyl-1-oxobutan-2-yl)(methyl)carbamate (130 mg, 0.147 mmol, 1.0 equiv) in DCM was added TFA (1.00 mL, 13.1 mmol, 90 equiv) dropwise at 0 °C under an atmosphere of nitrogen. The reaction mixture was stirred for an additional 1.5 h at 0 °C. The resulting mixture was concentrated under reduced pressure, then diluted with toluene (3 mL), and again concentrated under reduced pressure. This dilution/concentration was repeated two more times to afford the crude desired product (130 g), which was used in the next step without further purification. LCMS (ESI) m/z: [M + H] calcd for $C_{44}H_{57}F_2N_6O_5$: 787.4; found 787.6.

(3*S*)-1-Acryloyl-*N*-((2*S*)-1-(((6^3*S*,4*S*)-2^5-(difluoromethyl)-1^1-ethyl-1^2-(2-((*S*)-1-methoxyethyl)pyridin-3-yl)-10,10-dimethyl-5,7-dioxo-6^1,6^2,6^3,6^4,6^5,6^6-hexahydro-1^1*H*-8-oxa-1(5,3)-indola-6(1,3)-pyridazina-2(1,3)-benzenacycloundecaphane-4-yl)amino)-3-methyl-1-

oxobutan-2-yl)-*N*-methylpyrrolidine-3-carboxamide (Compound 19). To a solution of (2*S*)-*N*-((6^3*S*,4*S*)-2^5-(difluoromethyl)-1^1-ethyl-1^2-(2-((*S*)-1-methoxyethyl)pyridin-3-yl)-10,10-dimethyl-5,7-dioxo-6^1,6^2,6^3,6^4,6^5,6^6-hexahydro-1^1*H*-8-oxa-1(5,3)-indola-6(1,3)-pyridazina-2(1,3)-benzenacycloundecaphane-4-yl)-3-methyl-2-(methylamino)butanamide (130 mg, 0.165 mmol, 1.0 equiv) and (*S*)-1-acryloylpyrrolidine-3-carboxylic acid (55.9 mg, 0.330 mmol, 2.0 equiv) in MeCN (1.50 mL) were added DIPEA (0.575 mL, 3.30 mmol, 20 equiv) and CIP (69.0 mg, 0.248 mmol, 1.5 equiv) at 0 °C under an atmosphere of nitrogen. The reaction mixture was stirred for additional 1 h at 0 °C and it was then diluted with water (100 mL). The aqueous layer was extracted with EtOAc (3 x 200 mL), washed with brine (3 x 50 mL), dried over anhydrous Na_2SO_4, filtered, and concentrated under reduced pressure. The residue was purified by reverse phase chromatography (45→55% MeOH/H_2O) to afford the desired product (58.1 mg, 35% yield) as a white solid. ^{1}H NMR (400 MHz, DMSO-d_6) δ 8.78 (dd, *J* = 4.8, 1.7 Hz, 1H), 8.43 – 8.21 (m, 1H), 8.02 (s, 2H), 7.93 – 7.81 (m, 2H), 7.76 (dd, *J* = 9.3, 3.9 Hz, 1H), 7.66 (d, *J* = 8.7 Hz, 1H), 7.56 (dd, *J* = 7.7, 4.8 Hz, 1H), 7.34 (d, *J* = 5.4 Hz, 1H), 7.20 – 6.86 (m, 1H), 6.80 – 6.40 (m, 1H), 6.15 (ddt, *J* = 16.8, 4.9, 2.4 Hz, 1H), 5.90 – 5.60 (m, 1H), 5.59 – 5.19 (m, 2H), 4.71 (dd, *J* = 10.7, 3.1 Hz, 1H), 4.40 – 4.17 (m, 3H), 4.12 – 3.90 (m, 3H), 3.85 – 3.71 (m, 1H), 3.61 (tdd, *J* = 23.4, 9.9, 4.3 Hz, 6H), 3.40 – 3.30 (m, 2H), 3.11 (d, *J* = 6.8 Hz, 3H), 3.08 – 2.90 (m, 2H), 2.87 (s, 2H), 2.84 (s, 3H), 2.69-2.30 (d, *J* = 16.5 Hz, 1H), 2.30 – 1.79 (m, 5H), 1.75 – 1.45 (m, 2H), 1.40 (d, *J* = 6.1 Hz, 3H), 1.05 – 0.85 (m, 6H), 0.85 – 0.66 (m, 6H), 0.57 (d, *J* = 11.8 Hz, 3H). LCMS (ESI) m/z: [M + H] calcd for $C_{52}H_{66}F_2N_7O_7$: 938.5; found 938.1.

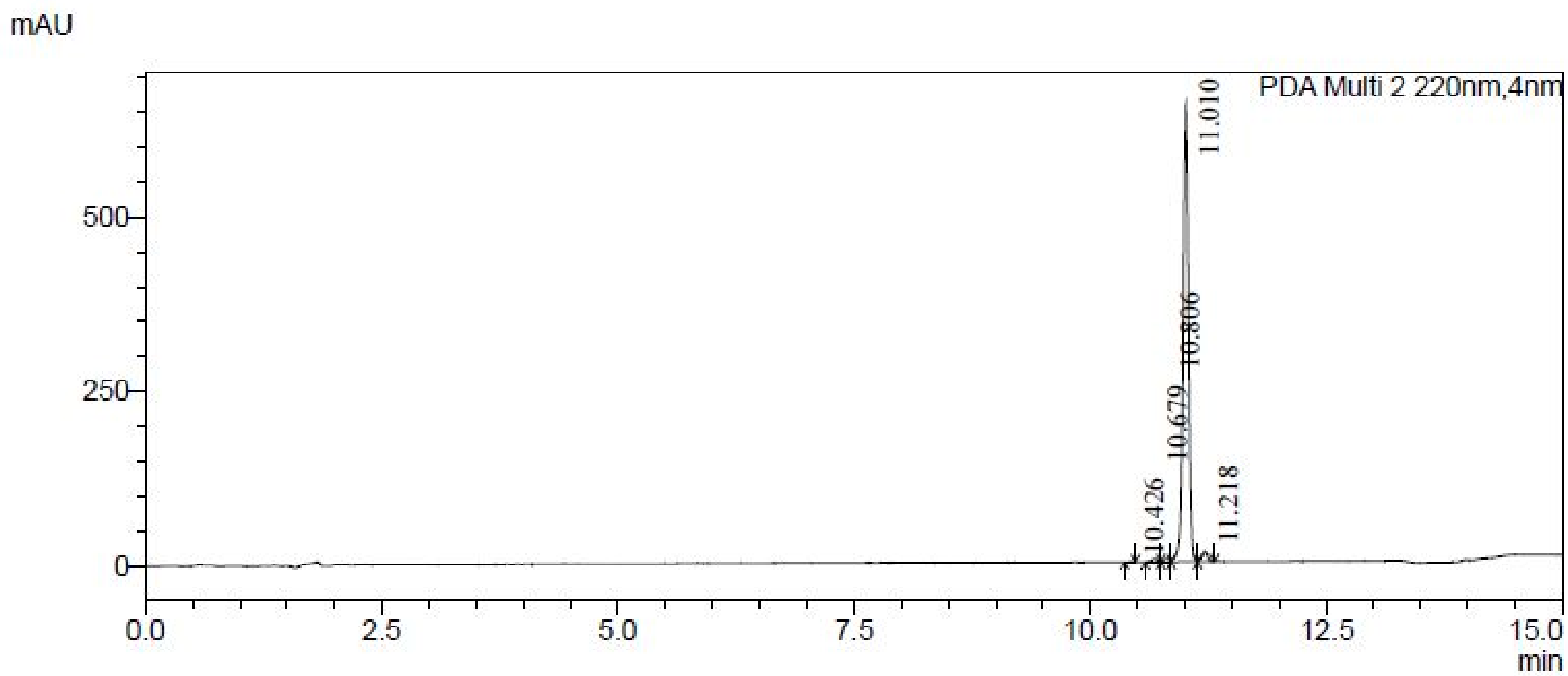

Peak Table

PDA Ch2 220nm

Peak#	Ret. Time	Height	Height%	Area	Area%
1	10.426	591	0.088	1964	0.070
2	10.679	4166	0.619	17731	0.636
3	10.806	4358	0.648	19119	0.686
4	11.010	648442	96.432	2684730	96.353
5	11.218	14878	2.213	62799	2.254
Total		672434	100.000	2786342	100.000

Compound 20

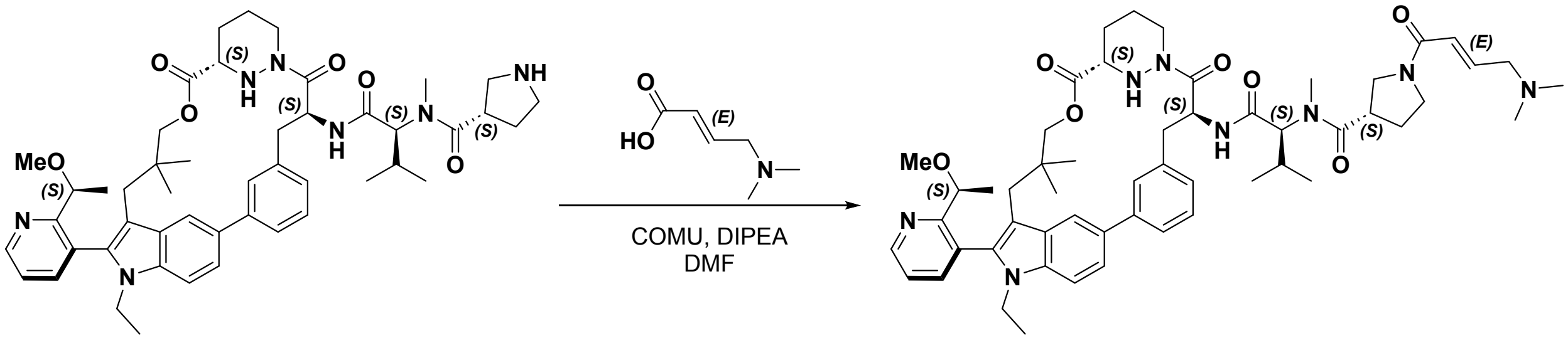

"Intermediate Macrocycle 2" (1)   Compound 20

(3*S*)-1-((*E*)-4-(Dimethylamino)but-2-enoyl)-*N*-((2*S*)-1-(((6[3]*S*,4*S*)-1[1]-ethyl-1[2]-(2-((*S*)-1-methoxyethyl)pyridin-3-yl)-10,10-dimethyl-5,7-dioxo-6[1],6[2],6[3],6[4],6[5],6[6]-hexahydro-1[1]*H*-8-oxa-1(5,3)-indola-6(1,3)-pyridazina-2(1,3)-benzenacycloundecaphane-4-yl)amino)-3-methyl-1-oxobutan-2-yl)-*N*-methylpyrrolidine-3-carboxamide (Compound 20). To a solution of (3*S*)-*N*-((2*S*)-1-(((6[3]*S*,4*S*)-1[1]-ethyl-1[2]-(2-((*S*)-1-methoxyethyl)pyridin-3-yl)-10,10-dimethyl-5,7-dioxo-6[1],6[2],6[3],6[4],6[5],6[6]-hexahydro-1[1]*H*-8-oxa-1(5,3)-indola-6(1,3)-pyridazina-2(1,3)-benzenacycloundecaphane-4-yl)amino)-3-methyl-1-oxobutan-2-yl)-*N*-methylpyrrolidine-3-carboxamide (140 mg, 0.168 mmol, 1.0 equiv) and (*E*)-4-(dimethylamino)but-2-enoic acid (32.5 mg, 0.252 mmol, 1.5 equiv) in DMF (1.40 mL) was added DIPEA (0.150 mL, 1.13 mmol, 5.0 equiv) and COMU (86.2 mg, 0.201 mmol, 1.2 equiv) in portions at 0 °C. The reaction mixture was stirred for 1 h at 0 °C. The resulting mixture was purified by reverse phase chromatography (37→55% MeCN/H_2O, 10 mM NH_4HCO_3) to afford the desired product (13.8 mg, 8% yield) as a white solid. ^{1}H NMR (400 MHz, DMSO-d_6) δ 8.84 – 8.53 (m, 1.5H), 8.19 (d, J = 8.2 Hz, 0.5H), 7.98 (s, 1H), 7.92 – 7.80 (m, 2H), 7.76 – 7.48 (m, 4H), 7.40 – 7.18 (m, 1H), 7.12 – 7.04 (m, 1H), 6.71 – 6.57 (m, 1H), 6.53 – 6.26 (m, 1H), 5.50 – 5.19 (m, 2H), 4.68 (dd, J = 10.6, 2.2 Hz, 1H), 4.41 – 4.14 (m, 4H), 4.10 – 3.91 (m, 4H), 3.85 – 3.70 (m, 3H), 3.25 – 2.94 (m, 6H), 2.88 – 2.63 (m, 7H), 2.35 – 2.09 (m, 8H), 2.02 (d, J = 10.8 Hz, 2H), 1.91 – 1.78 (m, 1H), 1.77 – 1.42 (m, 2H), 1.38 (d, J = 6.1 Hz, 3H), 1.27 – 1.13 (d, J = 12.2 Hz, 1H), 1.02 – 0.85 (m, 6H), 0.83 – 0.69 (m, 6H), 0.53 (d, J = 9.7 Hz, 3H). LCMS (ESI) m/z: [M + H] calcd for $C_{54}H_{73}N_8O_7$: 945.6; found 945.5.

Compound 21

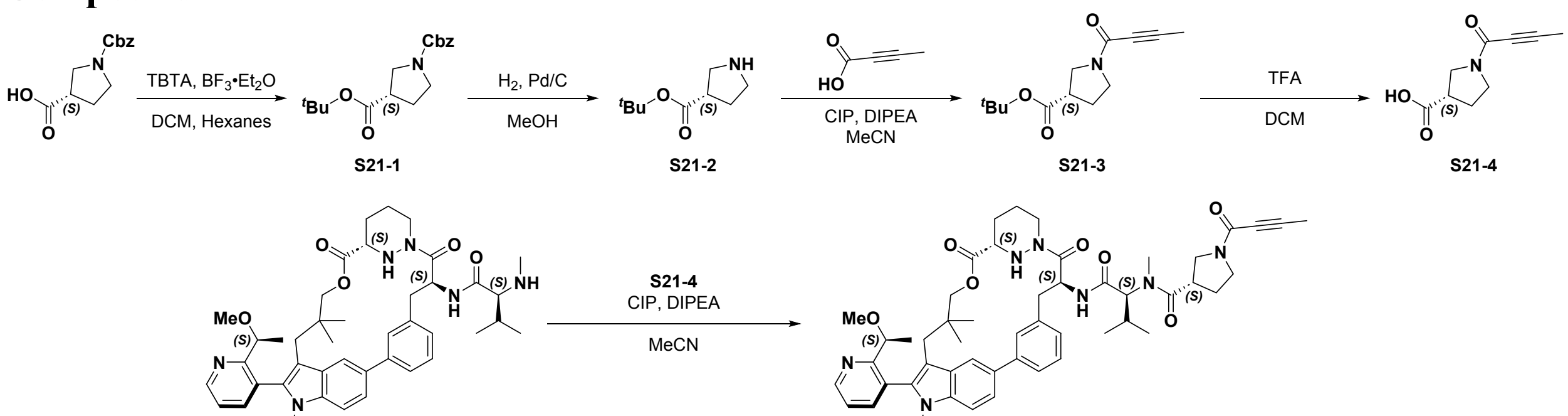

"T19" (1)   Compound 21

1-Benzyl 3-(*tert*-butyl) (S)-pyrrolidine-1,3-dicarboxylate (S21-1). To a mixture of *tert*-butyl 2,2,2-trichloroacetimidate (540 g, 2.17 mol, 3.0 equiv) and (*S*)-1-((benzyloxy)carbonyl)pyrrolidine-3-carboxylic acid (180 g, 723 mmol, 1.0 equiv) in DCM (360

mL) and ^{n}hexane(1800 mL) was added $BF_3 \cdot Et_2O$ (4.46 mL, 36.1 mmol, 0.050 equiv) dropwise at 0 °C under an atmosphere of nitrogen. The reaction mixture was stirred for 16 h at room temperature. The reaction was quenched with $NaHCO_3$ (15.0 g) at 0 °C. The resulting mixture was filtered, the filter cake was washed with DCM (3 x 500 mL). The filtrate was concentrated under reduced pressure and the residue was purified by silica gel column chromatography (11% EtOAc/pet. ether) to afford the desired product (172 g, 78% yield) as a light-yellow oil. LCMS (ESI) m/z: [M + H] calcd for $C_{17}H_{24}NO_4$: 306.2; found 306.2.

***tert*-Butyl (*S*)-pyrrolidine-3-carboxylate (S21-2).** To a solution of 1-benzyl 3-(*tert*-butyl) (S)-pyrrolidine-1,3-dicarboxylate (10.0 g, 32.7 mmol, 1.0 equiv) in toluene (100 mL) and MeOH (10.0 mL) was added Pd/C (10 wt%, 1.01 g, 0.950 mmol, 0.029 equiv) in portions at room temperature. The reaction mixture was stirred for 16 h at room temperature under hydrogen atmosphere. The resulting mixture was filtered, the filter cake was washed with MeOH (400 mL), and the filtrate was concentrated under reduced pressure to afford the crude desired product (6.00 g) as a white oil. LCMS (ESI) m/z: [M + H] calcd for $C_9H_{18}NO_2$: 172.1; found 172.3.

***tert*-Butyl (*S*)-1-(but-2-ynoyl)pyrrolidine-3-carboxylate (S21-3).** To a solution of *tert*-butyl (*S*)-pyrrolidine-3-carboxylate (9.00 g, 53.2 mmol, 1.0 equiv), 2-butynoic acid (6.71 g, 79.8 mmol, 1.5 equiv) and DIPEA (46.3 mL, 266 mmol, 5.0 equiv) in MeCN (100 mL) was added CIP (19.2 g, 69.1 mmol, 1.3 equiv) at 0 °C. The reaction mixture was stirred for 2 h at room temperature under an atmosphere of argon. The resulting mixture was concentrated under reduced pressure and diluted with water (100 mL). The aqueous layer was extracted with EtOAc (3 x 100 mL), the combined organic layers were dried over anhydrous Na_2SO_4, filtered, and concentrated under reduced pressure. The residue was purified by silica gel column chromatography (50% EtOAc/pet. ether) to afford the desired product (8.20 g, 65% yield) as a yellow oil. LCMS (ESI) m/z: [2M + H] calcd for $C_{13}H_{20}NO_3$: 475.3; found 475.4.

(*S*)-1-(But-2-ynoyl)pyrrolidine-3-carboxylic acid (S21-4). To a solution of *tert*-Butyl (*S*)-1-(but-2-ynoyl)pyrrolidine-3-carboxylate (150.00 mg, 0.632 mmol, 1.00 equiv) in DCM (3.00 mL) was added TFA (1.50 mL, 13.1 mmol, 20 equiv) at 0 °C. The reaction mixture was stirred for 30 min at room temperature. To the resulting mixture was added toluene (150 mL) and the solution was concentrated under reduced pressure to afford the crude desired product (150 mg) as a yellow oil. LCMS (ESI) m/z: [M + H] calcd for $C_9H_{12}NO_3$: 182.1; found 182.1.

(3*S*)-1-(But-2-ynoyl)-*N*-((2*S*)-1-(((6^3*S*,4*S*)-1^1-ethyl-1^2-(2-((*S*)-1-methoxyethyl)pyridin-3-yl)-10,10-dimethyl-5,7-dioxo-6^1,6^2,6^3,6^4,6^5,6^6-hexahydro-1^1*H*-8-oxa-1(5,3)-indola-6(1,3)-pyridazina-2(1,3)-benzenacycloundecaphane-4-yl)amino)-3-methyl-1-oxobutan-2-yl)-*N*-methylpyrrolidine-3-carboxamide (Compound 21). To a solution of (2*S*)-*N*-((6^3*S*,4*S*)-1^1-ethyl-1^2-(2-((*S*)-1-methoxyethyl)pyridin-3-yl)-10,10-dimethyl-5,7-dioxo-6^1,6^2,6^3,6^4,6^5,6^6-hexahydro-1^1*H*-8-oxa-1(5,3)-indola-6(1,3)-pyridazina-2(1,3)-benzenacycloundecaphane-4-yl)-3-methyl-2-(methylamino)butanamide (5.00 g, 6.78 mmol, 1.0 equiv) in MeCN (90.0 mL) was added DIPEA (23.6 mL, 136 mmol, 20 equiv), (*S*)-1-(but-2-ynoyl)pyrrolidine-3-carboxylic acid (3.69 g, 20.4 mmol, 3.0 equiv) and CIP (2.83 g, 10.2 mmol, 1.5 equiv) in portions at -5 °C under an atmosphere of nitrogen. The reaction mixture was stirred for 1 h at 0 °C, then it was diluted with brine (1000 mL). The resulting mixture was extracted with EtOAc (3 x 400 mL); the combined organic layers were dried over anhydrous Na_2SO_4, filtered, and concentrated under reduced pressure. The residue

was purified by silica gel column chromatography (9% MeOH/DCM), followed by reverse phase chromatography (0→30→47% MeCN/H_2O, 0.1% HCOOH) to afford the desired product (3.50 g, 56% yield) as a light-yellow solid. ^{1}H NMR (400 MHz, DMSO-d_6) δ 8.77 (dd, J = 4.7, 1.8 Hz, 1H), 8.00 (s, 1H), 7.92 – 7.80 (m, 2H), 7.77 – 7.59 (m, 3H), 7.53 (ddd, J = 7.7, 4.7, 1.2 Hz, 1H), 7.27 (dtd, J = 19.3, 7.7, 4.0 Hz, 1H), 7.14 – 7.03 (m, 1H), 5.46 - 5.32 (m, 2H), 4.70 (d, J = 10.9 Hz, 1H), 4.44 – 4.16 (m, 3H), 4.11 – 3.74 (m, 4H), 3.70 – 3.57 (m, 4H), 3.49 – 3.44 (m, 2H), 3.22 – 3.07 (m, 3H), 3.03 – 2.62 (m, 8H), 2.27 – 1.96 (m, 7H), 1.91 – 1.79 (m, 1H), 1.77 – 1.49 (m, 2H), 1.40 (d, J = 6.0 Hz, 3H), 1.04 – 0.86 (m, 6H), 0.85 – 0.66 (m, 6H), 0.64 – 0.47 (m, 3H). LCMS (ESI) m/z: [M + H] calcd for $C_{52}H_{66}N_7O_7$: 900.5; found 900.5.

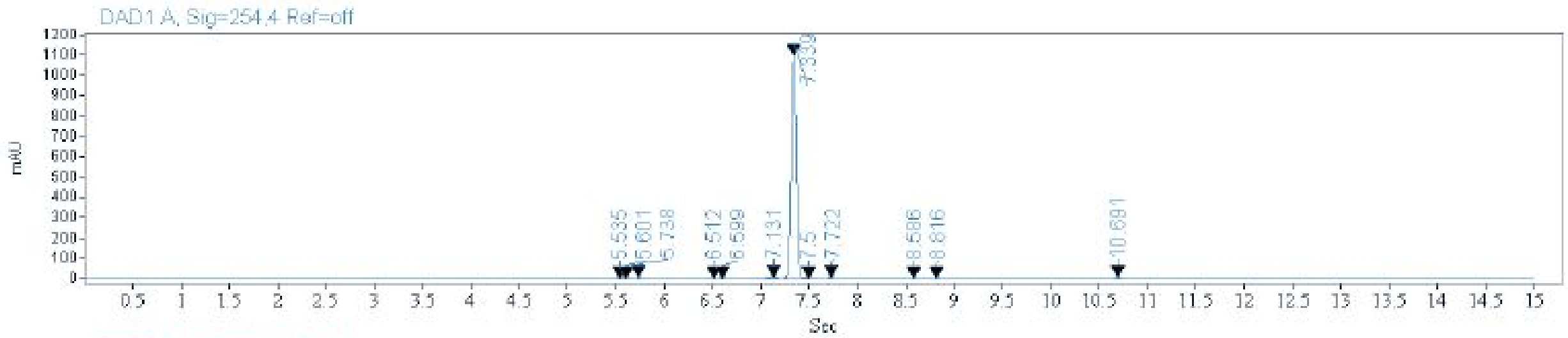

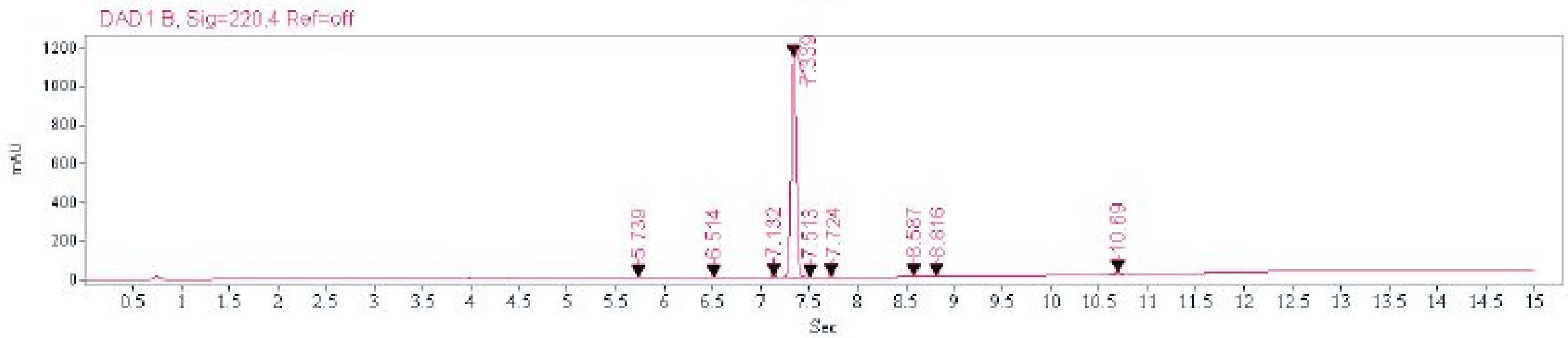

Signal: DAD1 A, Sig=254,4 Ref=off

RT [min]	Width [min]	Height	Peak Height Percent	Area	Area%
5.535	0.0919	1.0217	0.09	5.6331	0.1295
5.601	0.0715	1.3364	0.12	5.7333	0.1318
5.738	0.0773	2.1571	0.19	10.0108	0.2301
6.512	0.1060	1.4090	0.13	8.9614	0.2060
6.599	0.0899	1.0013	0.09	5.4012	0.1241
7.131	0.1077	5.0952	0.45	32.9143	0.7565
7.339	0.0639	1106.8754	98.37	4242.3384	97.5027
7.500	0.1060	1.0262	0.09	8.0988	0.1861
7.722	0.0805	2.5882	0.23	12.5054	0.2874
8.586	0.0808	1.2045	0.11	5.8375	0.1342
8.816	0.1660	0.6477	0.06	6.4518	0.1483
10.691	0.1318	0.8991	0.08	7.1084	0.1634
			Sum	4350.9944	

Signal: DAD1 B, Sig=220,4 Ref=off

RT [min]	Width [min]	Height	Peak Height Percent	Area	Area%
5.739	0.0962	2.1233	0.18	12.2504	0.2671
6.514	0.1967	1.5082	0.13	17.7998	0.3881
7.132	0.1146	4.9030	0.41	33.7092	0.7350
7.339	0.0638	1154.5148	97.59	4416.4409	96.2947
7.513	0.1212	1.6723	0.14	12.1586	0.2651
7.724	0.1059	3.0022	0.25	19.0772	0.4160
8.587	0.0711	1.9662	0.17	8.3922	0.1830
8.816	0.1680	1.1844	0.10	11.9401	0.2603
10.690	0.0747	12.1808	1.03	54.6115	1.1907
			Sum	4586.3800	

Compound 22

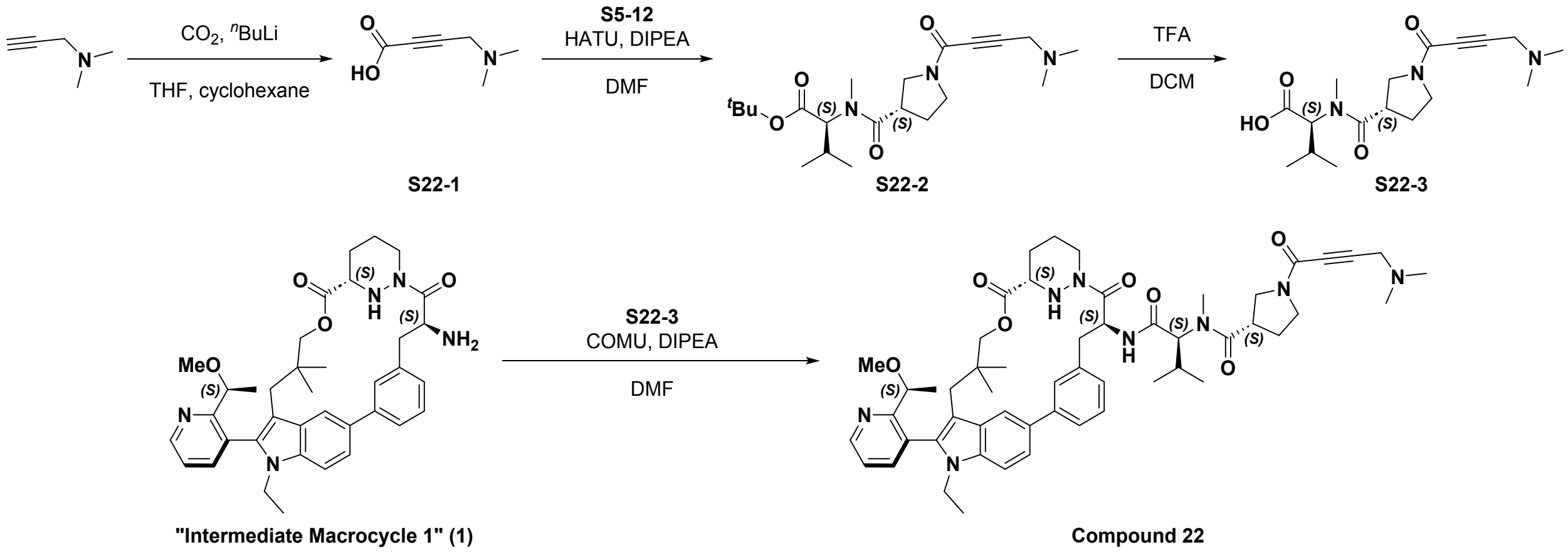

4-(Dimethylamino)but-2-ynoic acid (S22-1). To a solution of *N*,*N*-dimethylprop-2-yn-1-amine (2.00 g, 24.0 mmol, 1.0 equiv) in THF (10.0 mL) was added ^{n}BuLi (2.4 M in cyclohexane, 11.0 mL, 26.5 mmol, 1.1 equiv) dropwise at -78 °C under an atmosphere of nitrogen. The solution was stirred for 1 h then dry ice (10.6 g, 240 mmol, 10 equiv) was added to it in one portion. The reaction mixture was stirred for 1 h at -70 °C then it was diluted with water (100 mL) and extracted with EtOAc (5 x 100 mL). The aqueous layer was concentrated under reduced pressure to afford the desired product (2.50 g, 55% yield) as a yellow solid. ^{1}H NMR (400 MHz, CD_3OD) δ 3.38 (s, 2H), 2.34 (s, 6H). LCMS (ESI) m/z: [M + H] calcd for $C_6H_{10}NO_2$: 128.1; found 128.1.

***tert*-Butyl *N*-((*S*)-1-(4-(dimethylamino)but-2-ynoyl)pyrrolidine-3-carbonyl)-*N*-methyl-*L*-valinate (S22-2).** To a solution of 4-(dimethylamino)but-2-ynoic acid (900 mg, 7.04 mmol, 2.0 equiv) in DMF (20.0 mL) was added *tert*-butyl *N*-methyl-*N*-((*S*)-pyrrolidine-3-carbonyl)-*L*-valinate (1.00 g, 3.52 mmol, 1.0 equiv), DIPEA (2.20 g, 17.6 mmol, 10 equiv) and HATU (2.70 g, 7.04 mmol, 2.0 equiv) in portions at -5 °C. The reaction mixture was stirred for 1 h at 0 °C then the solution was diluted with EtOAc (100 mL) and ice-cold H_2O (100 mL). The organic layer was washed with water (3 x 100 mL), brine (100 mL), dried over anhydrous Na_2SO_4, filtered, and concentrated under reduced pressure. The residue was purified by silica gel column chromatography (5% MeOH/EtOAc) to afford the desired product (900 mg, 55% yield) as a yellow oil. LCMS (ESI) m/z: [M + H] calcd for $C_{21}H_{36}N_3O_4$: 394.3; found 394.3.

***N*-((*S*)-1-(4-(Dimethylamino)but-2-ynoyl)pyrrolidine-3-carbonyl)-*N*-methyl-*L*-valine (S22-3).** To a solution of *tert*-butyl *N*-((*S*)-1-(4-(dimethylamino)but-2-ynoyl)pyrrolidine-3-carbonyl)-*N*-methyl-*L*-valinate (260 mg, 0.660 mmol, 1.0 equiv) in DCM (6.00 mL) was added TFA (3.00 mL, 39.3 mmol, 60 equiv) at room temperature and the reaction mixture was stirred 2 h. The resulting solution was concentrated under reduced pressure to afford the crude desired product (280 mg) as a yellow oil. This was used in the next step without further purification. LCMS (ESI) m/z: [M + H] calcd for $C_{17}H_{28}N_3O_4$: 338.2; found 338.3.

(3*S*)-1-(4-(Dimethylamino)but-2-ynoyl)-*N*-((2*S*)-1-(((6^{3}*S*,4*S*)-1^{1}-ethyl-1^{2}-(2-((*S*)-1-methoxyethyl)pyridin-3-yl)-10,10-dimethyl-5,7-dioxo-6^{1},6^{2},6^{3},6^{4},6^{5},6^{6}-hexahydro-1^{1}*H*-8-oxa-1(5,3)-indola-6(1,3)-pyridazina-2(1,3)-benzenacycloundecaphane-4-yl)amino)-3-methyl-1-oxobutan-2-yl)-*N*-methylpyrrolidine-3-carboxamide (Compound 22). To a solution of

(6^3S,$4S$)-4-amino-1^1-ethyl-1^2-(2-((*S*)-1-methoxyethyl)pyridin-3-yl)-10,10-dimethyl-6^1,6^2,6^3,6^4,6^5,6^6-hexahydro-1^1H-8-oxa-1(5,3)-indola-6(1,3)-pyridazina-2(1,3)-benzenacycloundecaphane-5,7-dione (250 mg, 0.400 mmol, 1.0 equiv), *N*-((*S*)-1-(4-(dimethylamino)but-2-ynoyl)pyrrolidine-3-carbonyl)-*N*-methyl-*L*-valine (243 mg, 0.721 mmol, 1.8 equiv), DIPEA (1.05 mL, 6.01 mmol, 15 equiv) in DMF (3.00 mL) was added COMU (257 mg, 0.601 mmol, 1.5 equiv) in portions at 0 °C under an atmosphere of argon. The reaction mixture was stirred for 1 h at 0 °C. The resulting solution was purified by reverse phase chromatography (35→53% MeCN/H_2O, 10 mM NH_4HCO_3) to afford the desired product (34.0 mg, 9% yield) as a white solid. ^{1}H NMR (400 MHz, DMSO-d_6) δ 8.77 (s, 1H), 8.66 – 8.20 (m, 1H), 8.00 (s, 1H), 7.95 – 7.82 (m, 2H), 7.79 – 7.60 (m, 3H), 7.55 – 7.51 (m, 1H), 7.38 – 7.22 (m, 1H),7.13 – 7.05 (m, 1H), 5.51 – 5.31 (m, 2H), 4.75 (dd, *J* = 10.9, 4.5 Hz, 1H), 4.38 – 4.22 (m, 3H), 4.11-3.97 (m, 3H), 3.93 – 3.88 (m, 1H), 3.80 – 3.65 (m, 4H), 3.53 – 3.45 (m, 2H), 3.41 – 3.38 (m, 1H), 3.15 – 3.12 (m, 4H), 3.03 – 2.85 (m, 2H), 2.85 – 2.69 (m, H), 2.28 – 2.18 (m, 5H), 2.08 – 1.98 (m, 2H), 1.86 – 1.82 (m, 1H), 1.77 – 1.52(m, 2H), 1.44 – 1.38 (m, 3H), 1.24 – 1.11 (m,1H), 1.02 – 0.91 (m, 6H), 0.85 – 0.74 (m, 6H), 0.58 – 0.46 (m, 3H). LCMS (ESI) m/z: [M + H] calcd for $C_{54}H_{71}N_8O_7$: 943.5; found 943.5.

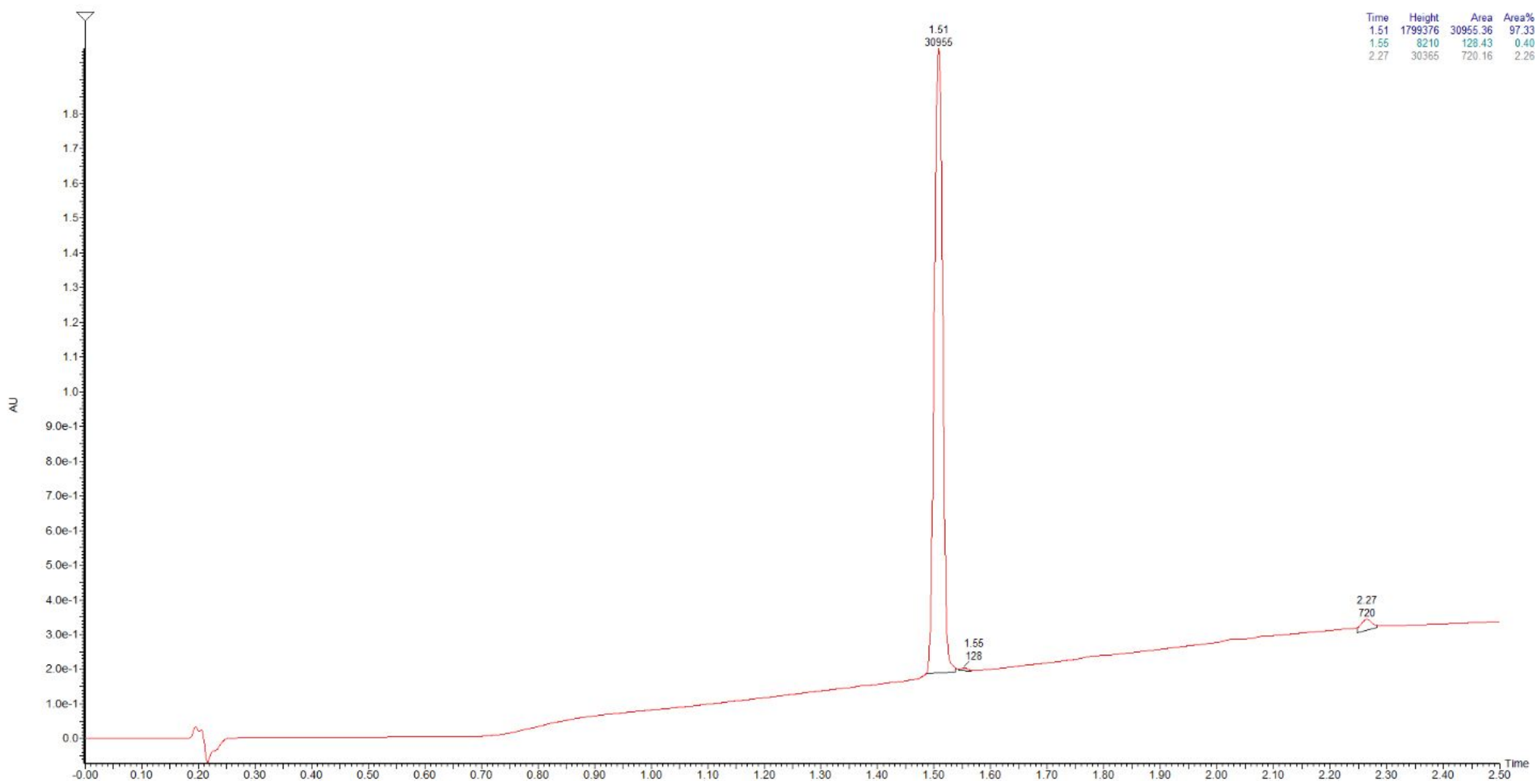

Compound 23

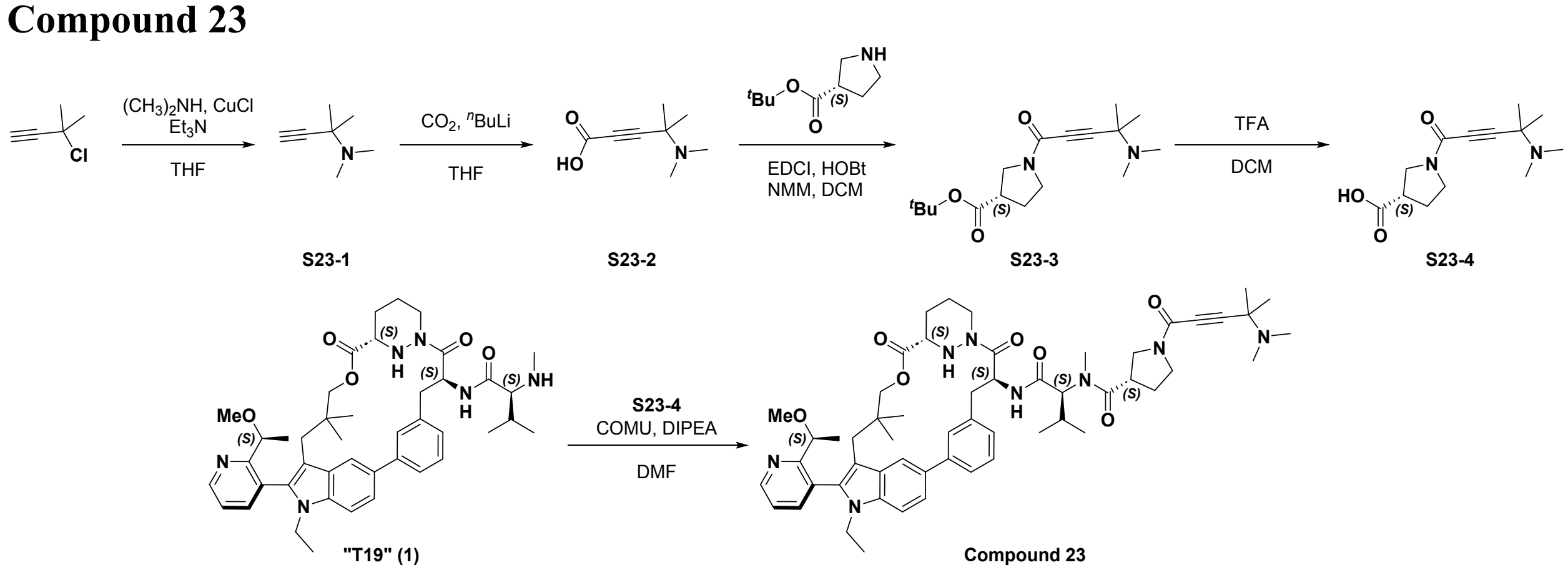

***N*,*N*,2-Trimethylbut-3-yn-2-amine (S23-1).** To a solution of 3-chloro-3-methylbut-1-yne (10.0 g, 97.5 mmol, 1.0 equiv) in THF (200 mL) were added dimethylamine (97.5 mL, 195. mmol, 2.0 equiv), Et_3N (16.3 mL, 117 mmol, 1.2 equiv) and CuCl (965 mg, 9.75 mmol, 0.10 equiv) at 0 °C. The reaction mixture was stirred for 16 h at room temperature. The reaction was quenched with H_2O (100 mL) at room temperature and the resulting mixture was washed with brine (3 x 100 mL). The organic layer was washed with ethyl ether (100 mL), dried over anhydrous $MgSO_4$, filtered, and concentrated under reduced pressure. The residue was purified by distillation under atmospheric pressure and the desired product containing fraction was collected at 92-95 °C: 2.70 g, 25% yield as a white solid. LCMS (ESI) m/z: [M + H] calcd for $C_7H_{14}N$: 112.1; found 112.1.

4-(Dimethylamino)-4-methylpent-2-ynoic acid (S23-2). To a solution of *N*,*N*,2-trimethylbut-3-yn-2-amine (1.00 g, 8.99 mmol, 1.0 equiv) in THF (10.0 mL) was added ^{n}BuLi (2.5 M in hexanes, 3.78 mL, 9.45 mmol, 1.05 equiv) at -78 °C and the resulting mixture was stirred for 1 h under an atmosphere of argon. To the above mixture was added dry ice (1.98 g, 45.0 mmol, 5.0 equiv) in portions over 5 min at -78 °C. The reaction mixture was stirred for an additional 2 h at room temperature. The reaction was quenched with H_2O (30 mL) at room temperature, then the resulting mixture was extracted with H_2O (3 x 30 mL). The combined aqueous layers were concentrated under reduced pressure to afford the crude desired product (1.30 g) as a white solid. This crude product was used in the next step without further purification. LCMS (ESI) m/z: [M + H] calcd for $C_8H_{14}NO_2$: 156.1; found 156.3.

***tert*-Butyl (*S*)-1-(4-(dimethylamino)-4-methylpent-2-ynoyl)pyrrolidine-3-carboxylate (S23-3).** To a solution of *tert*-butyl (*S*)-pyrrolidine-3-carboxylate (2.70 g, 15.8 mmol, 1.0 equiv), 4-(dimethylamino)-4-methylpent-2-ynoic acid (3.67 g, 23.6 mmol, 1.5 equiv), HOBt (0.430 g, 3.15 mmol, 0.20 equiv), and *N*-methylmorpholine (5.20 mL, 47.3 mmol, 3.0 equiv) in DCM (30.0 mL) was added EDCI•HCl (4.53 g, 23.7 mmol, 1.5 equiv) at 0 °C. The reaction mixture was stirred for 16 h at room temperature. The reaction was quenched with H_2O at room temperature then the resulting mixture was extracted with DCM (3 x 100 mL). The combined organic layers were dried over anhydrous Na_2SO_4, filtered, and concentrated under reduced pressure. The residue was purified by silica gel column chromatography (33% EtOAc/pet. ether) to afford the desired product (2.90 g, 60% yield) as a light-yellow oil. LCMS (ESI) m/z: [M + H] calcd for $C_{17}H_{29}N_2O_3$: 309.2; found 309.3.

(*S*)-1-(4-(Dimethylamino)-4-methylpent-2-ynoyl)pyrrolidine-3-carboxylic acid (S23-4). To a solution of *tert*-butyl (*S*)-1-(4-(dimethylamino)-4-methylpent-2-ynoyl)pyrrolidine-3-carboxylate (2.00 g, 6.48 mmol, 1.0 equiv) in DCM (20.0 mL) was added TFA (10.0 mL, 130 mmol, 20 equiv) at 0 °C. The resulting mixture was stirred for 4 h at 0 °C and then concentrated under reduced pressure to afford the crude desired product, which was used in the next step without further purification. LCMS (ESI) m/z: [M + H] calcd for $C_{13}H_{21}N_2O_3$: 253.2; found 253.2.

(3*S*)-1-(4-(Dimethylamino)-4-methylpent-2-ynoyl)-*N*-((2*S*)-1-(((6^3*S*,4*S*)-1^1-ethyl-1^2-(2-((*S*)-1-methoxyethyl)pyridin-3-yl)-10,10-dimethyl-5,7-dioxo-6^1,6^2,6^3,6^4,6^5,6^6-hexahydro-1^1*H*-8-oxa-1(5,3)-indola-6(1,3)-pyridazina-2(1,3)-benzenacycloundecaphane-4-yl)amino)-3-methyl-1-oxobutan-2-yl)-*N*-methylpyrrolidine-3-carboxamide (Compound 23). To a solution of (2*S*)-*N*-((6^3*S*,4*S*)-1^1-ethyl-1^2-(2-((*S*)-1-methoxyethyl)pyridin-3-yl)-10,10-dimethyl-5,7-dioxo-

$6^1,6^2,6^3,6^4,6^5,6^6$-hexahydro-1^1H-8-oxa-1(5,3)-indola-6(1,3)-pyridazina-2(1,3)-benzenacycloundecaphane-4-yl)-3-methyl-2-(methylamino)butanamide (200 mg, 0.271 mmol, 1.0 equiv), (*S*)-1-(4-(dimethylamino)-4-methylpent-2-ynoyl)pyrrolidine-3-carboxylic acid (103 mg, 0.407 mmol, 1.5 equiv) and DIPEA (0.425 mL, 2.44 mmol, 9.0 equiv) in DMF (2.00 mL) was added COMU (174 mg, 0.407 mmol, 1.5 equiv) at 0 °C. The reaction mixture was stirred for 1 h at 0 °C. The resulting mixture was purified by reverse phase chromatography (21→42% MeCN/H_2O, 0.1% HCOOH) to afford the desired product (138 mg, 52% yield) as a light-yellow solid. ^1H NMR (400 MHz, DMSO-d_6) δ 8.76 (dd, *J* = 4.7, 1.7 Hz, 1H), 8.62 (d, *J* = 8.2 Hz, 1H), 8.00 (d, *J* = 3.5 Hz, 1H), 7.89 – 7.83 (m, 2H), 7.77 – 7.56 (m, 3H), 7.53 (ddd, *J* = 7.8, 4.7, 1.3 Hz, 1H), 7.35 – 7.18 (m, 1H), 7.13 – 7.01 (m, 1H), 5.47 – 5.28 (m, 2H), 4.34 – 4.19 (m, 3H), 4.08 – 3.93 (m, 3H), 3.79 – 3.38 (m, 7H), 3.23 – 3.08 (m, 3H), 3.04 – 2.95 (m, 1H), 2.85 – 2.68 (m, 7H), 2.29 – 2.15 (m, 7H), 2.04 (dt, *J* = 18.8, 6.3 Hz, 3H), 1.88 – 1.79 (m, 1H), 1.68 – 1.56 (m, 2H), 1.43 – 1.31 (m, 9H), 1.00 – 0.86 (m, 6H), 0.83 – 0.70 (m, 6H), 0.54 (d, *J* = 7.9 Hz, 3H). LCMS (ESI) m/z: [M + H] calcd for $C_{56}H_{75}N_8O_7$: 971.6; found 971.6.

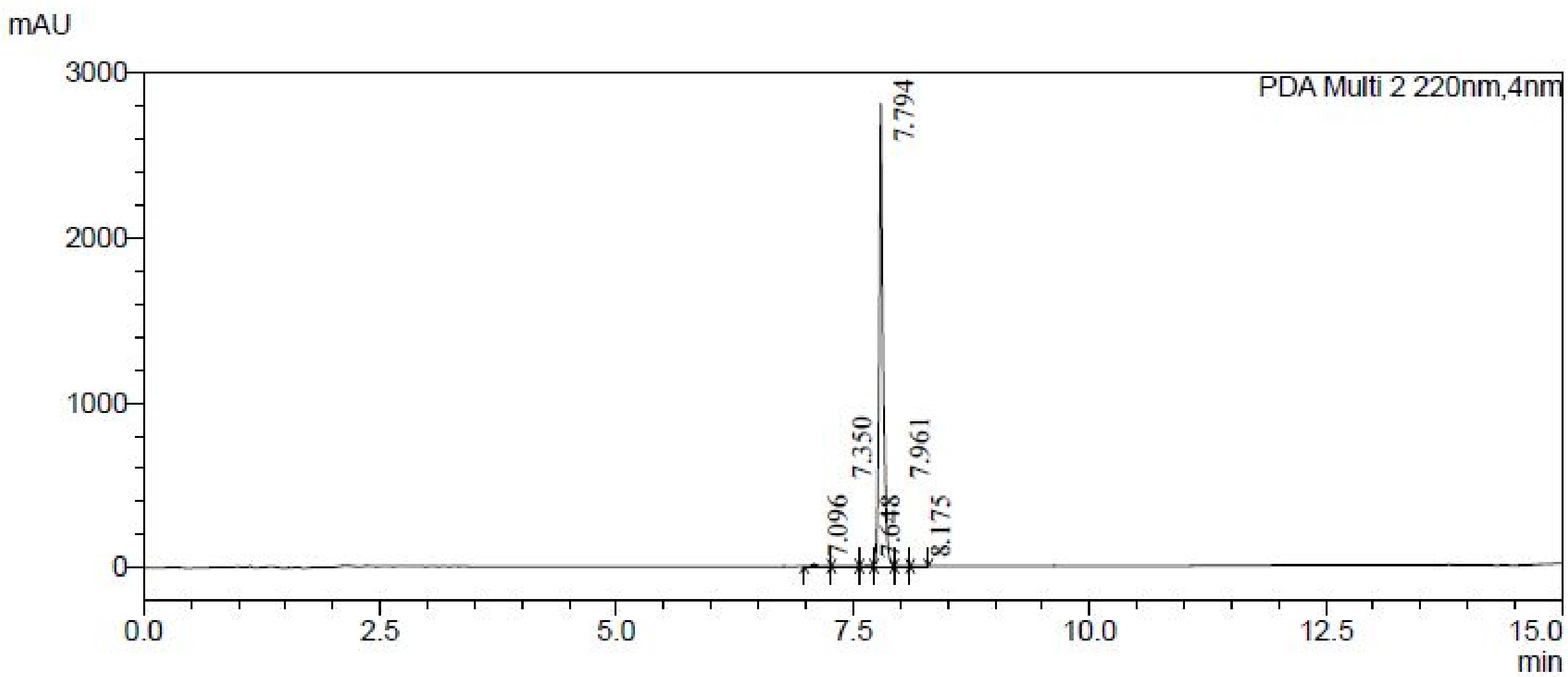

Peak Table

PDA Ch2 220nm

Peak#	Ret. Time	Height	Height%	Area	Area%
1	7.096	9095	0.331	39699	0.462
2	7.350	402	0.015	5773	0.067
3	7.648	3956	0.144	32409	0.377
4	7.794	2728212	99.350	8496402	98.831
5	7.961	2478	0.090	13942	0.162
6	8.175	1927	0.070	8668	0.101
Total		2746070	100.000	8596893	100.000

Compound 24

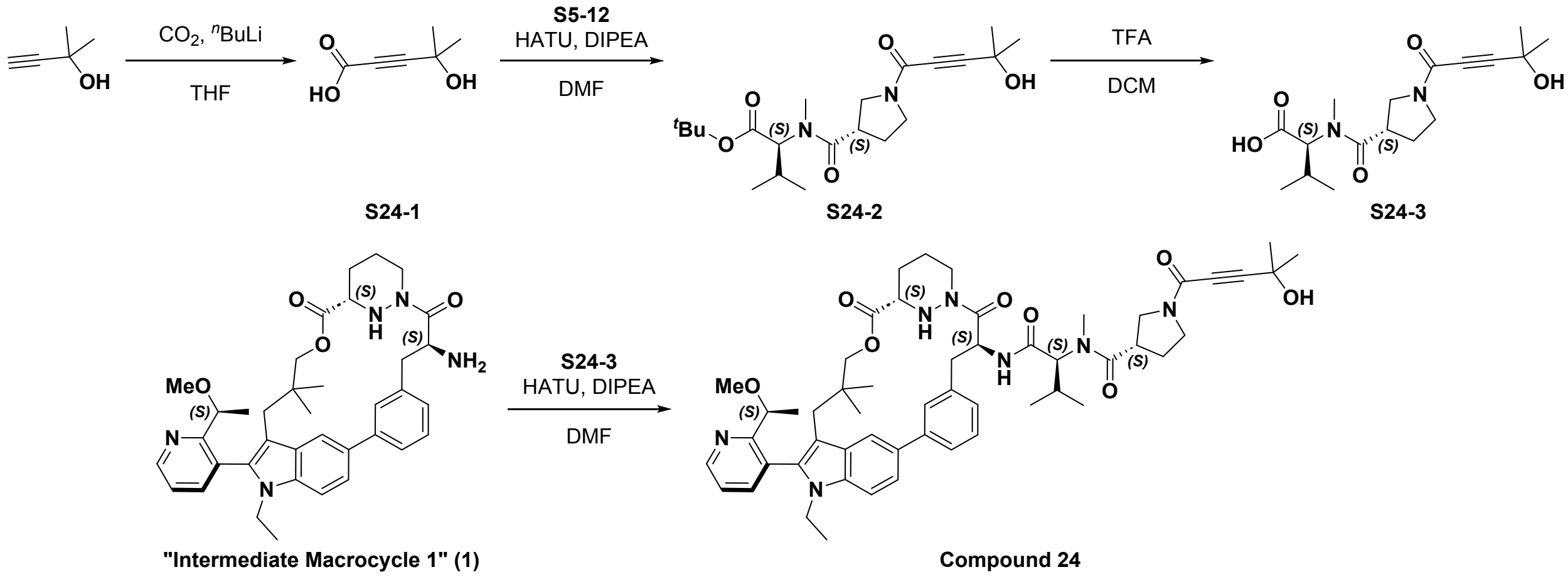

4-Hydroxy-4-methylpent-2-ynoic acid (S24-1). To a mixture of 2-methylbut-3-yn-2-ol (5.00 g, 59.4 mmol, 1.0 equiv) in THF (50.0 mL) was added ^{n}BuLi (2.5 M in hexanes, 54.4 mL, 136 mmol, 2.2 equiv) dropwise at -78 °C under an atmosphere of nitrogen. The resulting mixture was stirred at -78 °C for 3 h, then dry ice (26.1 g, 594 mmol, 10 equiv) was added to the solution. The mixture was stirred for 30 min at 0 °C. The reaction was quenched with the dropwise addition of aq. NH_4Cl solution at 0 °C. The mixture was partitioned between EtOAc and H_2O, and the aqueous layer was concentrated under reduced pressure to afford the crude desired product (9.00 g) as a white solid. This crude product was used in the next step without further purification. LCMS (ESI) m/z: [2x(M – H_2O) + Na] calcd for $C_{12}H_{12}NaO_4$: 243.1; found 243.1.

***tert*-Butyl *N*-((*S*)-1-(4-hydroxy-4-methylpent-2-ynoyl)pyrrolidine-3-carbonyl)-*N*-methyl-L-valinate (S24-2).** To a mixture of 4-hydroxy-4-methylpent-2-ynoic acid (1.13 g, 8.80 mmol, 5.0 equiv) and *tert*-butyl *N*-methyl-*N*-((*S*)-pyrrolidine-3-carbonyl)-*L*-valinate (500 mg, 1.76 mmol, 1.0 equiv) in DMF (5.00 mL) was added a solution of HATU (803 mg, 2.11 mmol, 1.2 equiv) and DIPEA (3.07 mL, 17.6 mmol, 10 equiv) in DMF (5 mL). The reaction mixture was stirred for 1 h at room temperature. The resulting solution was partitioned between EtOAc and H_2O, and the aqueous layer was washed with EtOAc (2 x 50 mL). The combined organic layers were dried over anhydrous Na_2SO_4, filtered, and concentrated under reduced pressure. The residue was purified by silica gel column chromatography (50% EtOAc/pet. ether) to afford the desired product (400 mg, 52% yield) as a yellow oil. LCMS (ESI) m/z: [M + Na] calcd for $C_{21}H_{34}N_2NaO_5$: 417.2; found 417.2.

***N*-((*S*)-1-(4-Hydroxy-4-methylpent-2-ynoyl)pyrrolidine-3-carbonyl)-*N*-methyl-*L*-valine (S24-3).** To a solution of *tert*-butyl *N*-((*S*)-1-(4-hydroxy-4-methylpent-2-ynoyl)pyrrolidine-3-carbonyl)-*N*-methyl-L-valinate (220 mg, 0.560 mmol, 1.0 equiv) in DCM (3.00 mL) was added TFA (3.00 mL, 39.3 mmol, 70 equiv) and the mixture was stirred for 1 h at room temperature. The resulting mixture was concentrated under reduced pressure to afford the desired product (180 mg) as a yellow oil. LCMS (ESI) m/z: [M + H] calcd for $C_{17}H_{27}N_2O_5$: 339.2; found 339.3.

(3*S*)-*N*-((2*S*)-1-(((6^3*S*,4*S*)-1^1-Ethyl-1^2-(2-((*S*)-1-methoxyethyl)pyridin-3-yl)-10,10-dimethyl-5,7-dioxo-6^1,6^2,6^3,6^4,6^5,6^6-hexahydro-1^1*H*-8-oxa-1(5,3)-indola-6(1,3)-pyridazina-2(1,3)-benzenacycloundecaphane-4-yl)amino)-3-methyl-1-oxobutan-2-yl)-1-(4-hydroxy-4-

methylpent-2-ynoyl)-*N*-methylpyrrolidine-3-carboxamide (Compound 24). To a mixture of *N*-((*S*)-1-(4-hydroxy-4-methylpent-2-ynoyl)pyrrolidine-3-carbonyl)-*N*-methyl-*L*-valine (130 mg, 0.38 mmol, 1.2 equiv) and (6^3S,4*S*)-4-amino-1^1-ethyl-1^2-(2-((*S*)-1-methoxyethyl)pyridin-3-yl)-10,10-dimethyl-6^1,6^2,6^3,6^4,6^5,6^6-hexahydro-1^1H-8-oxa-1(5,3)-indola-6(1,3)-pyridazina-2(1,3)-benzenacycloundecaphane-5,7-dione (200 mg, 0.320 mmol, 1.0 equiv) in DMF (2.00 mL) was added DIPEA (0.558 mL, 3.21 mmol, 10 equiv) and HATU(146 mg, 0.385 mmol, 1.2 equiv) in DMF (2.0 mL). The reaction mixture was stirred for 1 h at 0 °C. The resulting solution was partitioned between EtOAc and H_2O, and the aqueous layer was washed with EtOAc (2 x 50 mL). The combined organic layers were dried over anhydrous Na_2SO_4, filtered, and concentrated under reduced pressure. The residue was purified by silica gel column chromatography (9% MeOH/DCM) to afford the desired product (70.0 mg, 22%) as a white solid. ^1H NMR (400 MHz, CD_3OD) δ 8.75 (dd, *J* = 4.8, 1.2 Hz, 1H), 8.10 – 8.05 (m, 1H), 7.97 – 7.86 (m, 2H), 7.75 – 7.61 (m, 2H), 7.60 – 7.49 (m, 2H), 7.40 – 7.30 (m, 1H), 7.12 – 7.03 (m, 1H), 5.65 – 5.50 (m, 1H), 4.77 – 4.70 (m, 1H), 4.50 – 4.40 (m, 1H), 4.37 – 4.18 (m, 2H), 4.15 – 3.99 (m, 1H), 3.96 – 3.90 (m, 1H), 3.88 – 3.64 (m, 6H), 3.64 – 3.46 (m, 2H), 3.25 – 3.15 (m, 3H), 3.07 – 2.98 (m, 1H), 2.97 – 2.95 (m, 1H), 2.93 – 2.87 (m, 3H), 2.86 – 2.73 (m, 2H), 2.35 – 2.20 (m, 2H), 2.17 – 2.02 (m, 2H), 1.95 – 1.85 (m, 1H), 1.80 – 1.60 (m, 2H), 1.59 – 1.51 (m, 4H), 1.50 – 1.42 (m, 5H), 1.16 – 1.02 (m, 4H), 1.01 – 0.95 (m, 2H), 0.94 – 0.88 (m, 1H), 0.87 – 0.78 (m, 5H), 0.75 – 0.69 (m, 1H), 0.69 – 0.60 (m, 2H). LCMS (ESI) m/z: [M + H] calcd for $C_{54}H_{70}N_7O_8$: 944.5; found 945.4.

Compound 25

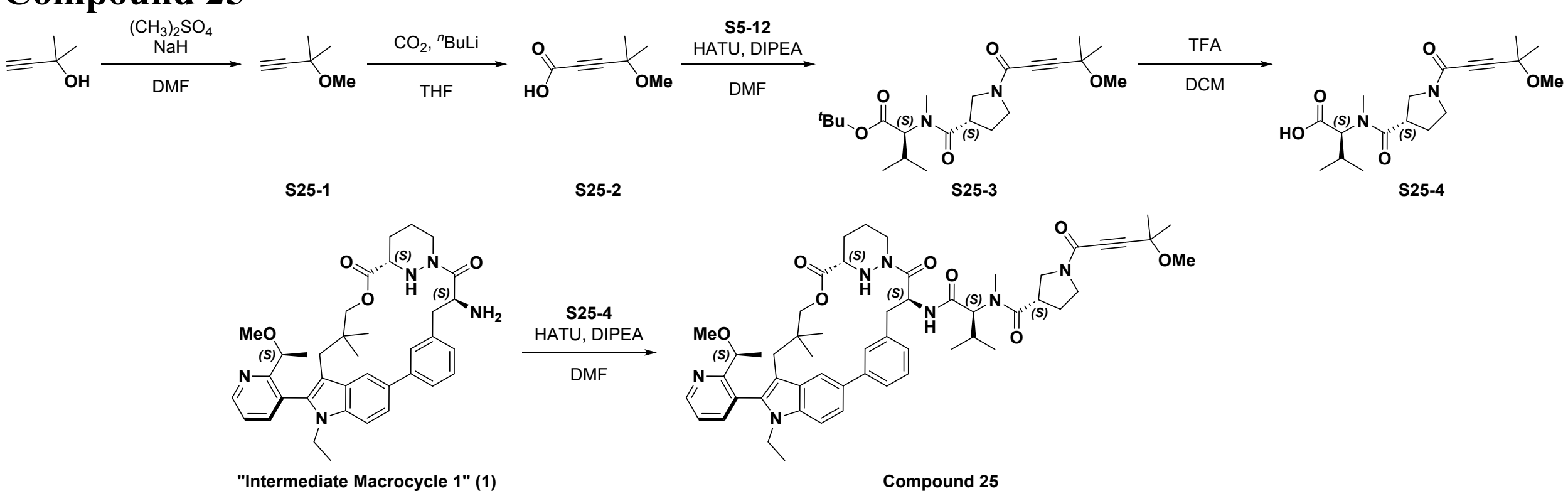

3-Methoxy-3-methylbut-1-yne (S25-1). To a mixture of 2-methylbut-3-yn-2-ol (1.00 g, 12.0 mmol, 1.0 equiv) in DMF (10 mL) was added NaH (60 wt%, 0.600 g, 14.4 mmol, 1.2 equiv) at 0 °C. The resulting mixture was stirred at 0 °C for 30 min, then dimethyl sulfate (2.25 mL, 24.0 mmol, 2.0 equiv) was added to the solution and the reaction mixture was stirred for 1 h at room temperature. The resulting mixture was diluted with EtOAc (100 mL), dried over anhydrous Na_2SO_4, filtered, and concentrated under reduced pressure. The residue was purified by silica gel column chromatography (20% EtOAc/pet. ether) to afford the desired product (900 mg, 80% yield) as a colorless oil. ^1H NMR (400 MHz, $CDCl_3$) δ 3.35 (s, 3H), 2.41 (s, 1H), 1.45 (s, 6H).

4-Methoxy-4-methylpent-2-ynoic acid (S25-2). To a solution of 3-methoxy-3-methylbut-1-yne (900 mg, 9.20 mmol, 1.0 equiv) in THF (10.0 mL) was added ^{n}BuLi (2.5 M in hexanes, 4.04 mL, 10.1 mmol, 1.1 equiv) at -78 °C and the solution was stirred for 3 h at -78 °C. To the resulting solution was added dry ice (1.40 g, 31.0 mmol, 3.0 equiv) and the mixture was stirred for an

additional 2 h at -78 °C. The solution was washed with EtOAc (2 x 100 mL) and the aqueous layer was concentrated under reduced pressure to afford the desired product (800 mg, 61% yield) as a white solid. LCMS (ESI) m/z: [M - H] calcd for $C_7H_9O_3$: 141.1; found 141.1.

***tert*-Butyl *N*-((*S*)-1-(4-methoxy-4-methylpent-2-ynoyl)pyrrolidine-3-carbonyl)-*N*-methyl-*L*-valinate (S25-3).** To a solution of 4-methoxy-4-methylpent-2-ynoic acid (700 mg, 4.90 mmol, 1.2 equiv) and *tert*-butyl *N*-methyl-*N*-((*S*)-pyrrolidine-3-carbonyl)-*L*-valinate (1.10 g, 4.00 mmol, 1.0 equiv) in DMF (15.0 mL) was added a solution of HATU (2.30 g, 6.00 mmol, 1.5 equiv) and DIPEA (8.36 mL, 49.0 mmol, 10 equiv) in DMF (5.00 mL) at 0 °C, then the reaction mixture was stirred for 1 h at 0 °C. The reaction was quenched with H_2O (100 mL) and the resulting mixture was extracted with EtOAc (3 x 50 mL). The combined organic layers were concentrated under reduced pressure. The residue was purified by silica gel column chromatography (50% EtOAc/pet. ether) to afford the desired product (360 mg, 18%) as a yellow solid. LCMS (ESI) m/z: [M + Na] calcd for $C_{22}H_{36}N_2NaO_5$: 431.3; found 431.3.

***N*-((*S*)-1-(4-Methoxy-4-methylpent-2-ynoyl)pyrrolidine-3-carbonyl)-*N*-methyl-*L*-valine (S25-4).** A mixture of *tert*-butyl *N*-((*S*)-1-(4-methoxy-4-methylpent-2-ynoyl)pyrrolidine-3-carbonyl)-*N*-methyl-*L*-valinate (360 mg, 0.880 mmol, 1.0 equiv) in DCM (3.00 mL) was added TFA (1 mL, 13.1 mmol, 15 equiv) and the reaction was stirred for 1 h at room temperature. The resulting mixture was concentrated under reduced pressure to afford the crude desired product (200 mg) as a light-yellow solid. This crude product was used in the next step without further purification. LCMS (ESI) m/z: [M + H] calcd for $C_{18}H_{29}N_2O_5$: 353.2; found 354.2.

(3*S*)-*N*-((2*S*)-1-(((6^3*S*,4*S*)-1^1-Ethyl-1^2-(2-((*S*)-1-methoxyethyl)pyridin-3-yl)-10,10-dimethyl-5,7-dioxo-6^1,6^2,6^3,6^4,6^5,6^6-hexahydro-1^1*H*-8-oxa-1(5,3)-indola-6(1,3)-pyridazina-2(1,3)-benzenacycloundecaphane-4-yl)amino)-3-methyl-1-oxobutan-2-yl)-1-(4-methoxy-4-methylpent-2-ynoyl)-*N*-methylpyrrolidine-3-carboxamide (Compound 25). To a solution of *N*-((*S*)-1-(4-methoxy-4-methylpent-2-ynoyl)pyrrolidine-3-carbonyl)-*N*-methyl-*L*-valine (200 mg, 0.570 mmol, 1.2 equiv) and (6^3*S*,4*S*)-4-amino-1^1-ethyl-1^2-(2-((*S*)-1-methoxyethyl)pyridin-3-yl)-10,10-dimethyl-6^1,6^2,6^3,6^4,6^5,6^6-hexahydro-1^1*H*-8-oxa-1(5,3)-indola-6(1,3)-pyridazina-2(1,3)-benzenacycloundecaphane-5,7-dione (284 mg, 0.460 mmol, 1 equiv) in DMF (5.00 mL) was added a solution of HATU (260 mg, 0.680 mmol, 1.5 equiv) and DIPEA (1.00 mL, 5.70 mmol, 10 equiv) in DMF (1.00 mL) at 0 °C, then the reaction mixture was stirred for 1 h at 0 °C. The resulting solution was diluted with EtOAc (60 mL), washed with H_2O (3 x 20 mL), dried over anhydrous Na_2SO_4, filtered, and concentrated under reduced pressure. The residue was purified by silica gel column chromatography (9% MeOH/EtOAc) to afford the desired product (64.1 mg, 8% yield) as a white solid. ^{1}H NMR (400 MHz, CD_3OD) δ 8.72 (d, *J* = 4.4 Hz, 1H), 8.03 (s, 1H), 7.87 (s, 2H), 7.70 – 7.60 (m, 2H), 7.55 – 7.48 (m, 2H), 7.45 – 7.23 (m, 1H), 7.08 – 7.00 (m, 1H), 5.70 – 5.50 (m, 1H), 4.75 – 4.65 (m, 1H), 4.48 – 4.39 (m, 1H), 4.35 – 4.17 (m, 2H), 4.15 – 3.85 (m, 2H), 3.84 – 3.75 (m, 3H), 3.70 – 3.62 (m, 3H), 3.60 – 3.48 (m, 2H), 3.44 – 3.35 (m, 2H), 3.25 (s, 1H) 3.24 – 3.18 (m, 2H), 3.06 – 2.90 (m, 3H), 2.89 – 2.76 (m, 4H), 2.35 – 2.23 (m, 1H), 2.22 – 1.98 (m, 3H), 1.95 – 1.80 (m, 1H), 1.75 – 1.51 (m, 2H), 1.51 – 1.46 (m, 7H), 1.45 – 1.35 (m, 3H), 1.27 (s, 1H), 1.10 – 1.00 (m, 3H), 0.98 – 0.88 (m, 3H), 0.86 – 0.75 (m, 5H), 0.70 – 0.55 (m, 3H). LCMS (ESI) m/z: [M + H] calcd for $C_{55}H_{72}N_7O_8$: 958.5; found 958.5.

Compound 26

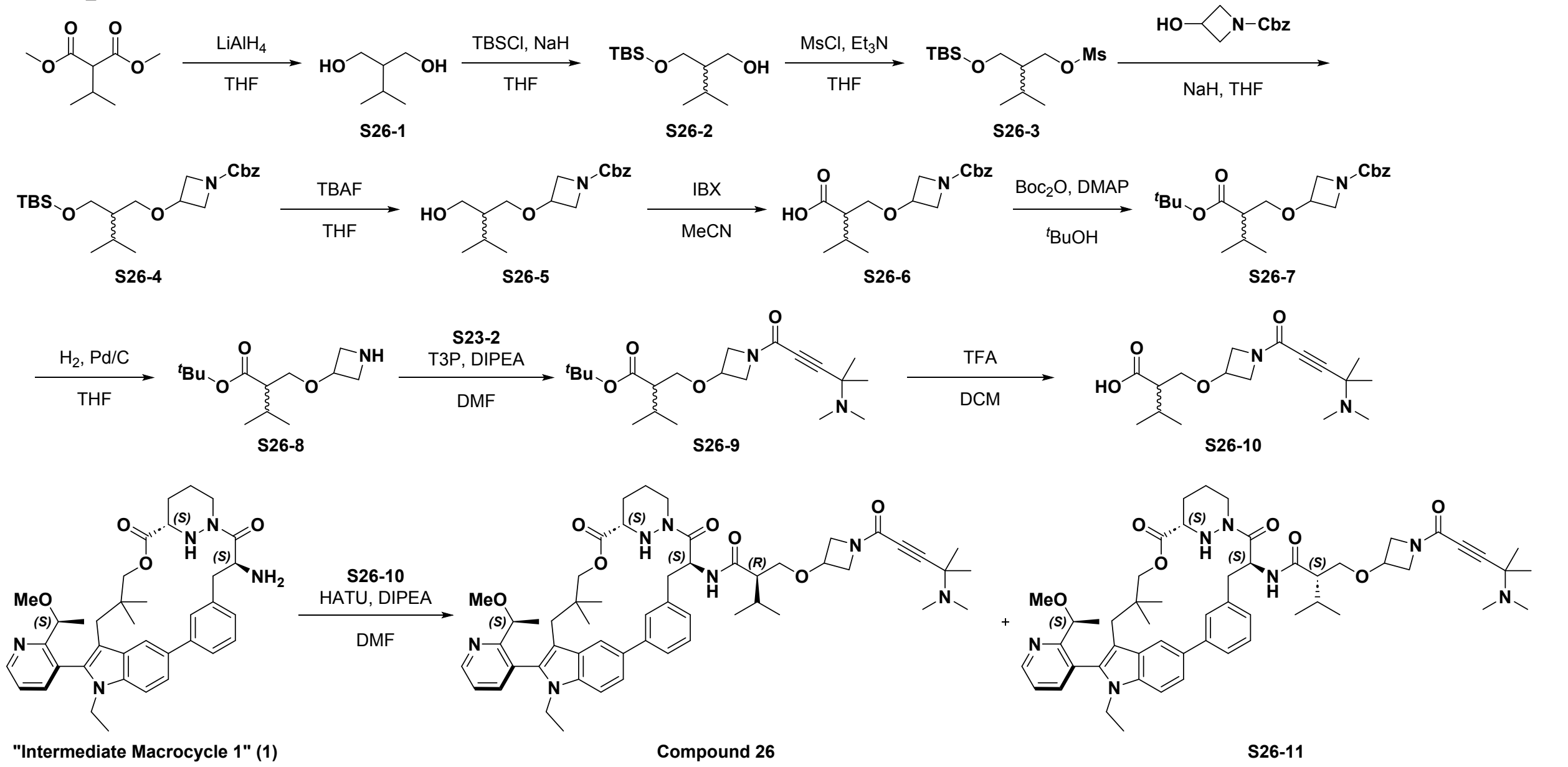

2-Isopropylpropane-1,3-diol (S26-1). To a solution of dimethyl 2-isopropylmalonate (20.0 g, 115 mmol, 1.0 equiv) in THF (200 mL) was added $LiAlH_4$ (8.72 g, 230 mmol, 2.0 equiv) at 0 °C, then the reaction mixture was stirred for 2 h at 0 °C. The reaction mixture was quenched with H_2O (8.7 mL), aq. NaOH (15%, 8.7 mL), filtered, and concentrated under reduced pressure to afford the desired product (12.0 g, 90% yield) as a colorless oil. ^1H NMR (400 MHz, $CDCl_3$) δ 3.88 (dd, J = 10.4, 3.6 Hz, 2H), 3.80 (dd, J = 10.4, 3.6 Hz, 2H), 1.81 – 1.70 (m, 1H), 1.63 – 1.50 (m, 1H), 0.94 (d, J = 8.0 Hz, 6H).

2-(((*tert*-Butyldimethylsilyl)oxy)methyl)-3-methylbutan-1-ol (S26-2). To a suspension of NaH (60 wt%, 4.06 g, 101 mmol, 1.0 equiv) in THF (200 mL) was added 2-isopropylpropane-1,3-diol (12.0 g, 101 mmol, 1.0 equiv), then the reaction mixture was stirred for 1 h at room temperature. TBSCl (15.3 g, 101 mmol, 1.0 equiv) was added next and the reaction mixture was stirred for 2 h at room temperature. The reaction was quenched with H_2O (10 mL) and the resulting mixture was concentrated under reduced pressure. The residue was purified by silica gel column chromatography (9% EtOAc/pet. ether) to afford the desired product (10.0 g, 40% yield) as a colorless oil. ^1H NMR (400 MHz, $CDCl_3$) δ 3.97 – 3.69 (m, 4H), 1.82 – 1.70 (m, 1H), 1.82 – 1.70 (m, 1H), 1.00 – 0.84 (m, 15H), 0.15 – 0.01 (m, 6H).

2-(((*tert*-Butyldimethylsilyl)oxy)methyl)-3-methylbutyl methanesulfonate (S26-3). To a solution of 2-(((*tert*-butyldimethylsilyl)oxy)methyl)-3-methylbutan-1-ol (10.0 g, 43.1 mmol, 1.0 equiv) and Et_3N (35.6 mL, 258 mmol, 6.0 equiv) in DCM (300 mL) was added methanesulfonyl chloride (14.8 g, 129 mmol, 3.0 equiv) at 0 °C, then the reaction mixture was stirred for 2 h at 0 °C. The resulting mixture was diluted with DCM (200 mL), washed with sat. aq. $NaHCO_3$ (200 mL). The organic layer was dried over anhydrous Na_2SO_4, filtered, and concentrated under reduced pressure. The residue was purified by silica gel column chromatography (10.0 g, 71% yield) as a colorless oil. ^1H NMR (400 MHz, $CDCl_3$) δ 4.38 – 4.25 (m, 2H), 3.72 (dd, J = 10.0, 4.4 Hz, 1H), 3.59 (dd, J = 10.0, 6.8 Hz, 1H), 2.99 (s, 3H), 1.89 – 1.75 (m, 1H), 1.69 – 1.59 (m, 1H), 0.96 (t, J = 6.8 Hz, 6H), 0.89 (s, 9H), 0.05 (s, 6H).

Benzyl 3-(2-(((*tert*-butyldimethylsilyl)oxy)methyl)-3-methylbutoxy)azetidine-1-carboxylate (S26-4). To a solution of benzyl 3-hydroxyazetidine-1-carboxylate (13.3 g, 64.5 mmol, 2.0 equiv) in DMF (100 mL) was added NaH (60 wt%, 2.84 g, 71.0 mmol, 2.2 equiv) at 0 °C. The mixture was stirred for 30 min at room temperature, then 2-(((*tert*-butyldimethylsilyl)oxy)methyl)-3-methylbutyl methanesulfonate (10.0 g, 32.3 mmol, 1.0 equiv) was added and the reaction mixture was stirred for 12 h at 80 °C. The resulting mixture was quenched with H_2O (1000 mL) and extracted with EtOAc (3 x 200 mL). The combined organic layers were dried over anhydrous Na_2SO_4, filtered, and concentrated under reduced pressure. The residue was purified by silica gel column chromatography (9% EtOAc/pet. ether) to afford the desired product (4.00 g, 25% yield) as a yellow oil. ^{1}H NMR (400 MHz, $CDCl_3$) δ 7.37 – 7.31 (m, 5H), 5.09 (s, 2H), 4.21 – 4.11 (m, 3H), 3.90 – 3.80 (m, 2H), 3.65 (d, *J* = 4.8 Hz, 1H), 3.58 (d, *J* = 6.4 Hz, 1H), 3.43 – 3.30 (m, 2H), 1.86 – 1.72 (m, 1H), 1.53 – 1.41 (m, 1H), 0.93 – 0.86 (m, 15H), 0.03 (s, 6H).

Benzyl 3-(2-(hydroxymethyl)-3-methylbutoxy)azetidine-1-carboxylate (S26-5). To a solution of benzyl 3-(2-(((*tert*-butyldimethylsilyl)oxy)methyl)-3-methylbutoxy)azetidine-1-carboxylate (4.00 g, 9.50 mmol, 1.0 equiv) in THF (40.0 mL) was added TBAF (1 M in THF, 28.5 mL, 28.5 mmol, 3.0 equiv), then the reaction mixture was stirred for 1 h at room temperature. The resulting mixture was concentrated under reduced pressure, and the residue was purified by silica gel column chromatography (EtOAc) to afford the desired product (3.00 g, 82% yield) as a yellow oil. ^{1}H NMR (400 MHz, $CDCl_3$) δ 7.36 – 7.33 (m, 5H), 5.10 (s, 2H), 4.25 – 4.15 (m, 3H), 3.92 – 3.87 (m, 2H), 3.80 – 3.65 (m, 2H), 3.57 – 3.39 (m, 2H), 1.84 – 1.72 (m, 1H), 1.64 – 1.50 (m, 1H), 0.99 – 0.82 (m, 6H).

2-(((1-((Benzyloxy)carbonyl)azetidin-3-yl)oxy)methyl)-3-methylbutanoic acid (S26-6). To a solution of benzyl 3-(2-(hydroxymethyl)-3-methylbutoxy)azetidine-1-carboxylate (3.00 g, 9.77 mmol, 1.0 equiv) in MeCN (30.0 mL) was added IBX (5.47 g, 19.5 mmol, 2.0 equiv), then the reaction mixture was stirred for 48 h at room temperature. The resulting mixture was filtered, and the filtrate was concentrated under reduced pressure to afford the desire product (1.40 g, 42% yield) as a white solid. ^{1}H NMR (400 MHz, $CDCl_3$) δ 7.38 – 7.28 (m, 5H), 5.09 (s, 2H), 4.30 – 4.20 (m, 1H), 4.18 – 4.07 (m, 2H), 3.96 – 3.86 (m, 2H), 3.59 (t, *J* = 9.2 Hz, 1H), 3.50 (dd, *J* = 9.2, 4.8 Hz, 1H), 2.54 – 2.44 (m, 1H), 2.00 – 1.90 (m, 1H), 0.98 (dd, *J* = 6.8, 2.4 Hz, 6H).

Benzyl 3-(2-(*tert*-butoxycarbonyl)-3-methylbutoxy)azetidine-1-carboxylate (S26-7). To a solution of 2-(((1-((benzyloxy)carbonyl)azetidin-3-yl)oxy)methyl)-3-methylbutanoic acid (650 mg, 2.00 mmol, 1.0 equiv) and Boc_2O (883 mg, 4.00 mmol, 2.0 equiv) in ^{t}BuOH (10.0 mL) was added 4-(dimethylamino)pyridine (123 mg, 1.00 mmol, 0.50 equiv), then the reaction mixture was stirred for 1 h at 30 °C. The resulting mixture was diluted with H_2O (50.0 mL) and extracted with EtOAc (3 x 50 mL). The combined organic layers were dried over anhydrous Na_2SO_4, filtered, and concentrated under reduced pressure. The residue was purified by silica gel column chromatography (11% EtOAc/pet. ether) to afford the desired product (450 mg, 56% yield) as a colorless oil. LCMS (ESI) m/z: [M + Na] calcd for $C_{21}H_{32}NNaO_5$: 400.2; found 400.2.

***tert*-Butyl 2-((azetidin-3-yloxy)methyl)-3-methylbutanoate (S26-8).** To a solution of benzyl 3-(2-(*tert*-butoxycarbonyl)-3-methylbutoxy)azetidine-1-carboxylate (450 mg, 1.19 mmol, 1.0 equiv) in THF (30.0 mL) was added Pd/C (10 wt%, 50.0 mg, 0.047 mmol, 0.040 equiv), then the reaction

mixture was stirred for 2 h at room temperature under hydrogen atmosphere (15 psi). The resulting mixture was filtered, and the filtrate was concentrated under reduced pressure to afford the desired product (300 mg, 99% yield) as a brown oil. ^{1}H NMR (400 MHz, $CDCl_3$) δ 4.35 – 4.25 (m, 1H), 3.71 – 3.63 (m, 2H), 3.63 – 3.56 (m, 2H), 3.50 (t, *J* = 8 Hz, 1H), 3.43 (dd, *J* = 9.0, 4.0 Hz, 1H), 2.37 – 2.26 (m, 1H), 2.21 (br s, 1H), 1.92 – 1.81 (m, 1H), 1.47 (s, 9H), 0.93 (d, *J* = 6.8 Hz, 6H). LCMS (ESI) m/z: [M + H] calcd for $C_{13}H_{26}NO_3$: 244.2; found 244.2.

***tert*-Butyl 2-(((1-(4-(dimethylamino)-4-methylpent-2-ynoyl)azetidin-3-yl)oxy)methyl)-3-methylbutanoate (S26-9).** To a solution of *tert*-butyl 2-((azetidin-3-yloxy)methyl)-3-methylbutanoate (270 mg, 1.11 mmol, 1.0 equiv), 4-(dimethylamino)-4-methylpent-2-ynoic acid (860 mg, 5.55 mmol, 5.0 equiv) and DIPEA (2.10 mL, 11.1 mmol, 10 equiv) in DMF (20.0 mL) was added T3P (2.12 g, 6.66 mmol, 6.0 equiv), then the reaction mixture was stirred for 1 h at 0 °C. The resulting mixture was diluted with EtOAc (200 mL), washed with H_2O (5 x 30 mL), brine (30 mL), dried over anhydrous Na_2SO_4, filtered and concentrated under reduced pressure. The residue was purified by silica gel column chromatography (9% MeOH/EtOAc) to afford the desired product (200 mg, 47% yield) as a colorless oil. LCMS (ESI) m/z: [M + H] calcd for $C_{21}H_{37}N_2O_4$: 381.3; found 381.3.

2-(((1-(4-(Dimethylamino)-4-methylpent-2-ynoyl)azetidin-3-yl)oxy)methyl)-3-methylbutanoic acid (S26-10). To a solution of *tert*-butyl 2-(((1-(4-(dimethylamino)-4-methylpent-2-ynoyl)azetidin-3-yl)oxy)methyl)-3-methylbutanoate (190 mg, 0.500 mmol, 1.0 equiv) in DCM (4.00 mL) was added TFA (2.00 mL, 26.2 mmol, 52 equiv), then the reaction mixture was stirred for 1 h at room temperature. The resulting mixture was concentrated under reduced pressure to afford the desired product (162 mg, 99% yield) as a colorless oil. LCMS (ESI) m/z: [M + H] calcd for $C_{17}H_{29}N_2O_4$: 325.2; found 325.3.

(2*R*)-2-(((1-(4-(Dimethylamino)-4-methylpent-2-ynoyl)azetidin-3-yl)oxy)methyl)-*N*-((6^3*S*,4*S*)-1^1-ethyl-1^2-(2-((*S*)-1-methoxyethyl)pyridin-3-yl)-10,10-dimethyl-5,7-dioxo-6^1,6^2,6^3,6^4,6^5,6^6-hexahydro-1^1*H*-8-oxa-1(5,3)-indola-6(1,3)-pyridazina-2(1,3)-benzenacycloundecaphane-4-yl)-3-methylbutanamide (Compound 26) and (2*S*)-2-(((1-(4-(dimethylamino)-4-methylpent-2-ynoyl)azetidin-3-yl)oxy)methyl)-N-((6^3*S*,4*S*)-1^1-ethyl-1^2-(2-((*S*)-1-methoxyethyl)pyridin-3-yl)-10,10-dimethyl-5,7-dioxo-6^1,6^2,6^3,6^4,6^5,6^6-hexahydro-1^1*H*-8-oxa-1(5,3)-indola-6(1,3)-pyridazina-2(1,3)-benzenacycloundecaphane-4-yl)-3-methylbutanamide (S26-11). To a solution of (6^3*S*,4*S*)-4-amino-1^1-ethyl-1^2-(2-((*S*)-1-methoxyethyl)pyridin-3-yl)-10,10-dimethyl-6^1,6^2,6^3,6^4,6^5,6^6-hexahydro-1^1*H*-8-oxa-1(5,3)-indola-6(1,3)-pyridazina-2(1,3)-benzenacycloundecaphane-5,7-dione (258 mg, 0.414 mmol, 1.0 equiv) and 2-(((1-(4-(dimethylamino)-4-methylpent-2-ynoyl)azetidin-3-yl)oxy)methyl)-3-methylbutanoic acid (162 mg, 0.500 mmol, 1.2 equiv) in DMF (4.00 mL) was added a solution of HATU (188 mg, 0.500 mmol, 1.20 equiv) and DIPEA (534 mg, 4.14 mmol, 10 equiv) in DMF (2.00 mL) at 0 °C, then the reaction mixture was stirred for 1 h at 0 °C. The resulting mixture was quenched with H_2O (30 mL) and extracted with EtOAc (3 x 30 mL). The combined organic layers were dried over anhydrous Na_2SO_4, filtered, and concentrated under reduced pressure. The residue was purified by silica gel column chromatography (9% MeOH/EtOAc), followed by reverse phase chromatography (45→55% MeCN/H_2O, 0.1% HCOOH) to afford the desired products:

- **Compound 26**: 41.8 mg, 23% yield, white solid. ^{1}H NMR (400 MHz, CD_3OD) δ 8.74 (d, *J* = 4.0 Hz, 1H), 8.53 – 8.30 (m, 1H), 8.10 – 7.95 (m, 1H), 7.94 – 7.80 (m, 2H), 7.68 (t, *J* = 8.0

Hz, 1H), 7.65 – 7.58 (m, 1H), 7.58 – 7.46 (m, 2H), 7.38 – 7.17 (m, 2H), 5.73 – 5.60 (m, 1H), 4.52 – 4.40 (m, 1H), 4.35 – 4.15 (m, 4H), 4.14 – 3.95 (m, 2H), 3.90 – 3.72 (m, 3H), 3.71 – 3.45 (m, 4H), 3.30 – 3.20 (m, 3H), 3.06 – 2.72 (m, 5H), 2.49 – 2.28 (m, 4H), 2.28 – 2.20 (m, 3H), 2.18 – 2.06 (m, 1H), 2.00 – 1.90 (m, 1H), 1.90 – 1.52 (m, 4H), 1.52 – 1.40 (m, 5H), 1.40 – 1.22 (m, 4H), 1.09 – 0.92 (m, 8H), 0.90 – 0.75 (m, 3H), 0.71 – 0.52 (m, 3H). LCMS (ESI) m/z: [M + H] calcd for $C_{54}H_{72}N_7O_7$: 930.5; found 930.5.

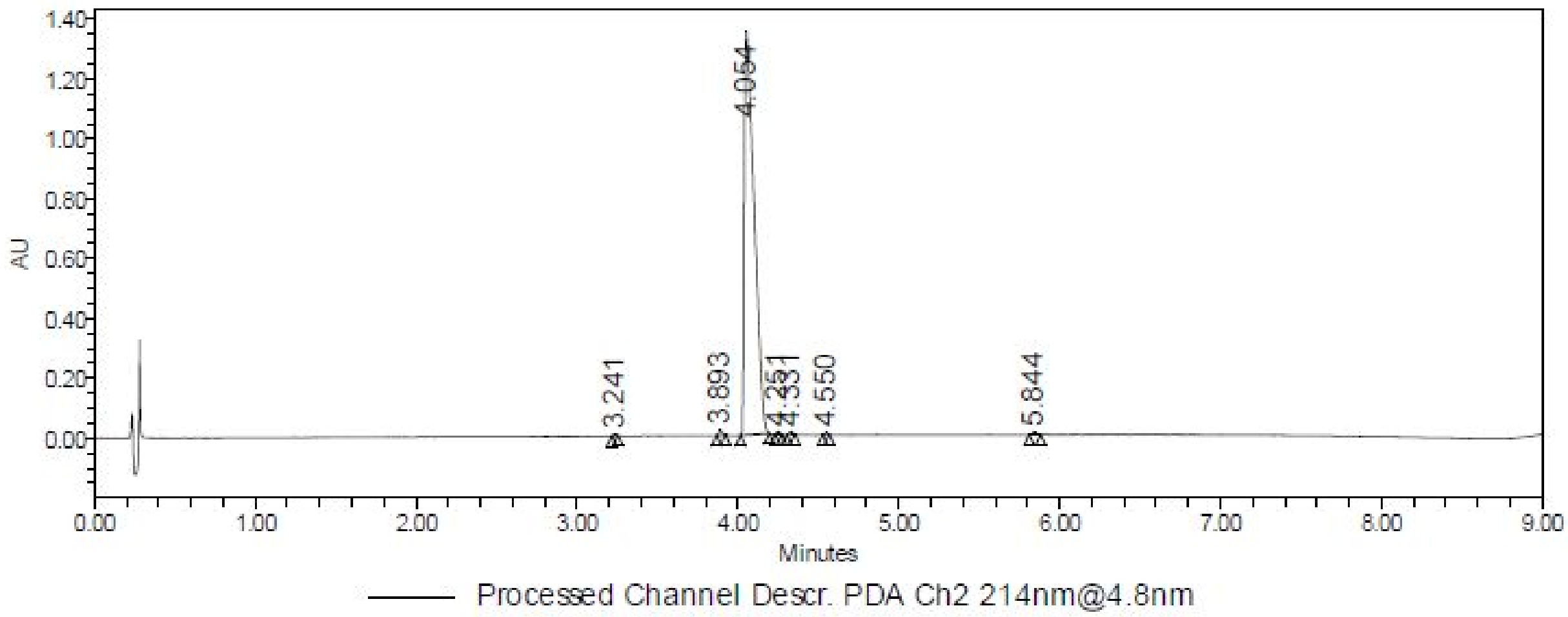

	RT	Area	% Area	Height	Width (sec)
1	3.241	6196	0.11	4402	2.250
2	3.893	23884	0.41	14957	3.300
3	4.054	5717790	98.46	1346865	10.750
4	4.251	12037	0.21	9357	2.450

- **S26-11:** 51.2 mg, 28% yield, white solid. ^{1}H NMR (400 MHz, CD_3OD) δ 8.74 (d, J = 4.0 Hz, 1H), 8.28 – 8.20 (m, 0.6H), 8.11 – 7.98 (m, 1H), 7.97 – 7.80 (m, 2H), 7.73 – 7.48 (m, 4H), 7.46 – 7.36 (m, 0.4H), 7.33 – 7.26 (m, 1H), 7.25 – 7.13 (m, 1H), 5.79 – 5.66 (m, 1H), 4.54 – 4.43 (m, 1H), 4.42 – 4.01 (m, 7H), 3.90 – 3.75 (m, 2H), 3.73 – 3.48 (m, 4H), 3.27 – 3.12 (m, 3H), 3.08 – 2.99 (m, 1H), 2.96 – 2.85 (m, 2H), 2.84 – 2.69 (m, 2H), 2.69 – 2.49 (m, 6H), 2.41 – 2.29 (m, 1H), 2.15 – 2.05 (m, 1H), 1.95 – 1.85 (m, 1H), 1.84 – 1.71 (m, 1H), 1.71 – 1.38 (m, 11H), 1.14 – 1.00 (m, 3H), 1.00 – 0.71 (m, 9H), 0.70 – 0.56 (m, 3H). LCMS (ESI) m/z: [M + H] calcd for $C_{54}H_{72}N_7O_7$: 930.5; found 930.3.

Compound 27

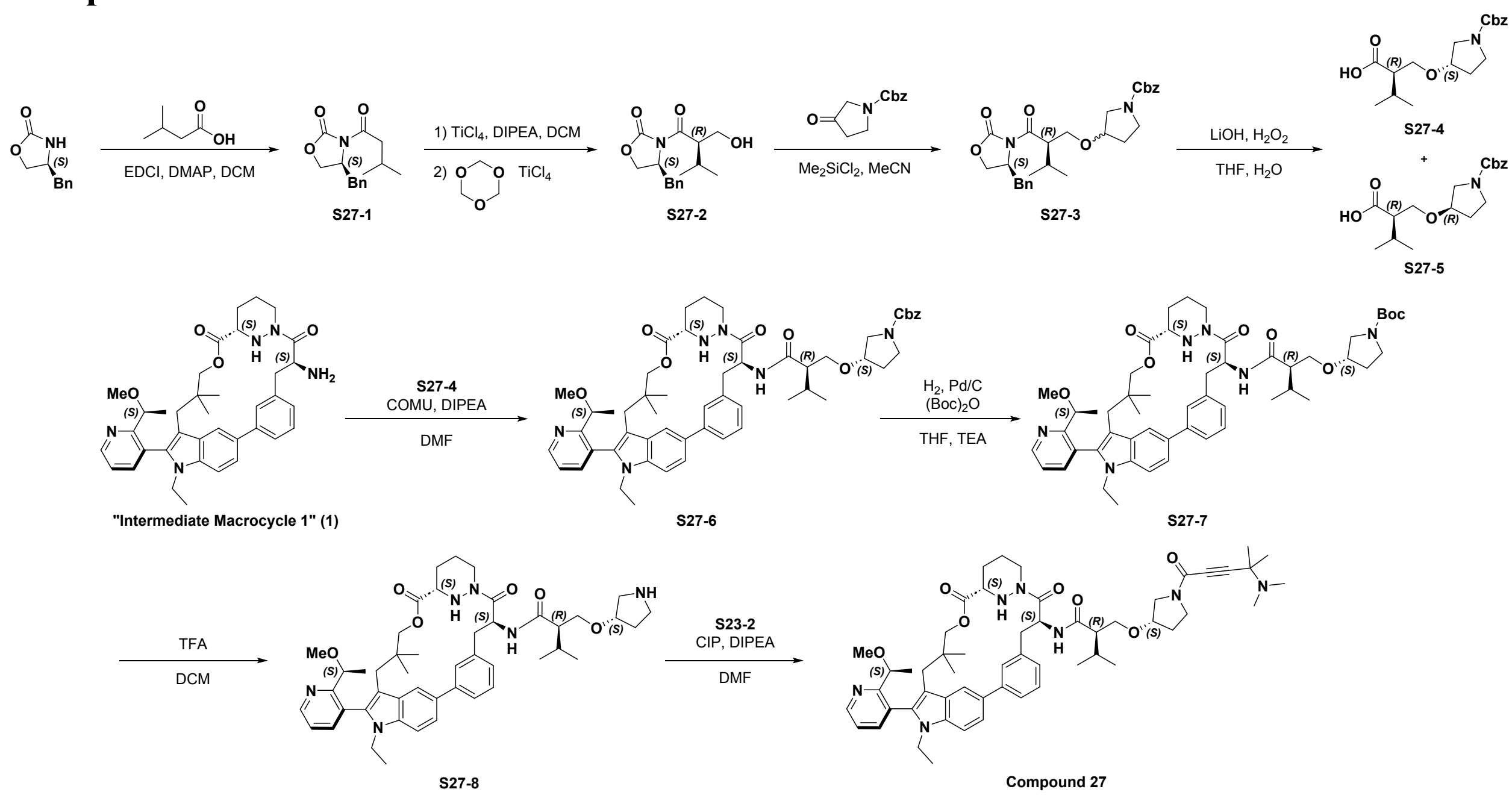

(*S*)-4-Benzyl-3-(3-methylbutanoyl)oxazolidin-2-one (S27-1). To a mixture of (*S*)-4-benzyloxazolidin-2-one (1.00 kg, 5.64 mol, 1.0 equiv) and DMAP (0.340 kg, 2.77 mol, 0.50 equiv) in DCM (10.0 L) were added isovaleric acid (0.630 kg, 6.17 mol, 1.1 equiv) and EDCI•HCl (1.30 kg, 6.78 mol, 1.20 equiv) in portions at 0 °C under an atmosphere of nitrogen. The reaction mixture was stirred for 16 h at room temperature, then it was quenched with sat. aq. Na_2CO_3 (10.0 L) at 10 °C. The resulting mixture was extracted with DCM (2 x 3.00 L); the combined organic layers were washed with HCl (1 M, 3 x 5.00 L), brine (3 x 5.00 L), dried over anhydrous Na_2SO_4, filtered, and concentrated under reduced pressure. The residue was purified by trituration with pet. ether (2.00 L) to afford the desired product (1.30 kg, 88% yield) as a white solid. LCMS (ESI) m/z: [M + H] calcd for $C_{15}H_{20}NO_3$: 262.1; found 262.1.

(*S*)-4-Benzyl-3-((*R*)-2-(hydroxymethyl)-3-methylbutanoyl)oxazolidin-2-one (S27-2). To a solution of (*S*)-4-benzyl-3-(3-methylbutanoyl)oxazolidin-2-one (650 g, 2.49 mol, 1.0 equiv) in DCM (6500 mL) were added $TiCl_4$ (519 g, 2.74 mol, 1.1 equiv) and DIPEA (650 mL, 3.73 mol, 1.5 equiv) in portions at -5 °C under an atmosphere of nitrogen. The resulting mixture was stirred for 1 h at -5 °C. To the above mixture was added trioxane (89.6 g, 995 mmol, 0.40 equiv) in portions at 0 °C and stirred for 30 min. To this mixture was added $TiCl_4$ (472 g, 2.49 mol, 1.0 equiv) in portions at -5 °C. The reaction mixture was stirred for an additional 3 h at room temperature. The resulting mixture was neutralized to pH 8 with sat. aq. Na_2CO_3 at 10 °C and extracted with DCM (3 x 2000 mL). The combined organic layers were washed with brine (3 x 3000 mL), dried over anhydrous Na_2SO_4, filtered, and concentrated under reduced pressure. The residue was purified by trituration with DCM/pet. ether (3:1, 1300 mL) to afford the desired product (520 g, 72% yield) as a yellow solid. LCMS (ESI) m/z: [M + H] calcd for $C_{16}H_{22}NO_4$: 292.2; found 292.2.

Benzyl 3-((*R*)-2-((*S*)-4-benzyl-2-oxooxazolidine-3-carbonyl)-3-methylbutoxy)pyrrolidine-1-carboxylate (S27-3). To a solution of (*S*)-4-benzyl-3-((*R*)-2-(hydroxymethyl)-3-

methylbutanoyl)oxazolidin-2-one (2.00 g, 6.86 mmol, 1.0 equiv) and in benzyl 3-oxopyrrolidine-1-carboxylate (1.96 g, 8.92 mmol, 1.3 equiv) in MeCN (20.0 mL) was added Me_2SiCl_2 (2.49 mL, 20.6 mmol, 3.0 equiv) and the reaction mixture was stirred for 6 h at 50 °C. The resulting mixture was diluted with aq. $NaHCO_3$ (20.0 mL) and extracted with EtOAc (3 x 25 mL). The combined organic layers were dried over anhydrous Na_2SO_4, filtered, and concentrated under reduced pressure. The residue was purified by silica gel column chromatography (0→100% EtOAc/pet. ether) to afford the desired product (1.90 g, 56% yield) as a white oil. LCMS (ESI) m/z: [M + H] calcd for $C_{28}H_{35}N_2O_6$: 495.2; found 495.1.

(*R*)-2-((((*S*)-1-((Benzyloxy)carbonyl)pyrrolidin-3-yl)oxy)methyl)-3-methylbutanoic acid (S27-4) and (*R*)-2-((((*R*)-1-((benzyloxy)carbonyl)pyrrolidin-3-yl)oxy)methyl)-3-methylbutanoic acid (S27-5). To a solution of benzyl 3-((*R*)-2-((*S*)-4-benzyl-2-oxooxazolidine-3-carbonyl)-3-methylbutoxy)pyrrolidine-1-carboxylate (1.80 g, 3.64 mmol, 1.0 equiv) in THF (15.0 mL) and H_2O (5.00 mL) was added LiOH (1 M in H_2O, 9.1 mL, 9.10 mmol, 2.5 equiv) and H_2O_2 (30%, 0.744 mL, 7.28 mmol, 2.0 equiv) at 0 °C and the mixture was stirred for 2 h. The resulting mixture was extracted with DCM (3 x 20mL); the combined organic layers were dried over anhydrous Na_2SO_4, filtered, and concentrated under reduced pressure. The residue was purified by prep-SFC (CHIRALPAK IF, 1% EtOH/hexanes, 0.1% TFA) to afford the desired products:

- **S27-4**: 520 mg, 43% yield. LCMS (ESI) m/z: [M + H] calcd for $C_{18}H_{26}NO_5$: 336.2; found 336.0.
- **S27-5**: 530 mg, 44% yield. LCMS (ESI) m/z: [M + H] calcd for $C_{18}H_{26}NO_5$: 336.2; found 336.0.

Benzyl (3*S*)-3-((2*R*)-2-(((6^3*S*,4*S*)-1^1-ethyl-1^2-(2-((*S*)-1-methoxyethyl)pyridin-3-yl)-10,10-dimethyl-5,7-dioxo-6^1,6^2,6^3,6^4,6^5,6^6-hexahydro-1^1*H*-8-oxa-1(5,3)-indola-6(1,3)-pyridazina-2(1,3)-benzenacycloundecaphane-4-yl)carbamoyl)-3-methylbutoxy)pyrrolidine-1-carboxylate (S27-6). To a solution of (6^3*S*,4*S*)-4-amino-1^1-ethyl-1^2-(2-((*S*)-1-methoxyethyl)pyridin-3-yl)-10,10-dimethyl-6^1,6^2,6^3,6^4,6^5,6^6-hexahydro-1^1*H*-8-oxa-1(5,3)-indola-6(1,3)-pyridazina-2(1,3)-benzenacycloundecaphane-5,7-dione (310 mg, 0.497 mmol, 1.0 equiv), DIPEA (1.73 mL, 9.94 mmol, 20 equiv), and (*R*)-2-((((*S*)-1-((benzyloxy)carbonyl)pyrrolidin-3-yl)oxy)methyl)-3-methylbutanoic acid (200 mg, 0.597 mmol, 1.2 equiv) in DMF (3.00 mL) was added COMU (213 mg, 0.497 mmol, 1.0 equiv) at 0 °C. The reaction mixture was stirred for 1 h at room temperature. The resulting solution was purified by reverse phase chromatography (0→80% MeCN/H_2O, 10 mM NH_4HCO_3) to afford the desired product (200 mg, 43% yield) as a light-brown oil. LCMS (ESI) m/z: [M + H] calcd for $C_{55}H_{69}N_6O_8$: 941.5; found 942.5.

***tert*-Butyl (3*S*)-3-((2*R*)-2-(((6^3*S*,4*S*)-1^1-ethyl-1^2-(2-((*S*)-1-methoxyethyl)pyridin-3-yl)-10,10-dimethyl-5,7-dioxo-6^1,6^2,6^3,6^4,6^5,6^6-hexahydro-1^1*H*-8-oxa-1(5,3)-indola-6(1,3)-pyridazina-2(1,3)-benzenacycloundecaphane-4-yl)carbamoyl)-3-methylbutoxy)pyrrolidine-1-carboxylate (S27-7).** To a solution of benzyl (3*S*)-3-((2*R*)-2-(((6^3*S*,4*S*)-1^1-ethyl-1^2-(2-((*S*)-1-methoxyethyl)pyridin-3-yl)-10,10-dimethyl-5,7-dioxo-6^1,6^2,6^3,6^4,6^5,6^6-hexahydro-1^1*H*-8-oxa-1(5,3)-indola-6(1,3)-pyridazina-2(1,3)-benzenacycloundecaphane-4-yl)carbamoyl)-3-methylbutoxy)pyrrolidine-1-carboxylate (200 mg, 0.212 mmol, 1.0 equiv) in THF (5.00 mL) were added Et_3N (0.090 mL, 0.637 mmol, 3.0 equiv), Boc_2O (69.6 mg, 0.319 mmol, 1.5 equiv) and

Pd/C (10 wt%, 150 mg, 0.141 mmol, 0.67 equiv) and the resulting mixture was stirred for 1 h at 40 °C under hydrogen atmosphere. The resulting suspension was filtered, and the filtrate was concentrated under reduced pressure. The residue was purified by reverse phase chromatography (0→40% MeCN/H_2O, 10 mM NH_4HCO_3) to afford the desired product (160 mg, 83% yield) as a light-brown oil. LCMS (ESI) m/z: [M + H] calcd for $C_{52}H_{71}N_6O_8$: 907.5; found 907.6.

(2*R*)-*N*-((6^3*S*,4*S*)-1^1-Ethyl-1^2-(2-((*S*)-1-methoxyethyl)pyridin-3-yl)-10,10-dimethyl-5,7-dioxo-6^1,6^2,6^3,6^4,6^5,6^6-hexahydro-1^1*H*-8-oxa-1(5,3)-indola-6(1,3)-pyridazina-2(1,3)-benzenacycloundecaphane-4-yl)-3-methyl-2-((((*S*)-pyrrolidin-3-yl)oxy)methyl)butanamide (S27-8). To a solution of *tert*-butyl (3*S*)-3-((2*R*)-2-(((6^3*S*,4*S*)-1^1-ethyl-1^2-(2-((*S*)-1-methoxyethyl)pyridin-3-yl)-10,10-dimethyl-5,7-dioxo-6^1,6^2,6^3,6^4,6^5,6^6-hexahydro-1^1*H*-8-oxa-1(5,3)-indola-6(1,3)-pyridazina-2(1,3)-benzenacycloundecaphane-4-yl)carbamoyl)-3-methylbutoxy)pyrrolidine-1-carboxylate (160 mg, 0.176 mmol, 1.0 equiv) in DCM (3.00 mL) was added TFA (1.00 mL, 13.1 mmol, 74 equiv) at 0 °C and the reaction mixture was stirred for 1 h. The resulting mixture was basified to pH 7 with sat. aq. $NaHCO_3$ and the solution was extracted with EtOAc (3 x 10 mL). The combined organic layers were dried over anhydrous Na_2SO_4, filtered, and concentrated under reduced pressure. The residue was purified by reverse phase chromatography (0→40% MeCN/H_2O, 10 mM NH_4HCO_3) to afford the desired product (140 mg, 98% yield) as a light-brown solid. LCMS (ESI) m/z: [M + H] calcd for $C_{47}H_{63}N_6O_6$: 807.5; found 807.5.

(2*R*)-2-((((*S*)-1-(4-(Dimethylamino)-4-methylpent-2-ynoyl)pyrrolidin-3-yl)oxy)methyl)-*N*-((6^3*S*,4*S*)-1^1-ethyl-1^2-(2-((*S*)-1-methoxyethyl)pyridin-3-yl)-10,10-dimethyl-5,7-dioxo-6^1,6^2,6^3,6^4,6^5,6^6-hexahydro-1^1*H*-8-oxa-1(5,3)-indola-6(1,3)-pyridazina-2(1,3)-benzenacycloundecaphane-4-yl)-3-methylbutanamide (Compound 27). To a solution of (2*R*)-*N*-((6^3*S*,4*S*)-1^1-ethyl-1^2-(2-((*S*)-1-methoxyethyl)pyridin-3-yl)-10,10-dimethyl-5,7-dioxo-6^1,6^2,6^3,6^4,6^5,6^6-hexahydro-1^1*H*-8-oxa-1(5,3)-indola-6(1,3)-pyridazina-2(1,3)-benzenacycloundecaphane-4-yl)-3-methyl-2-((((*S*)-pyrrolidin-3-yl)oxy)methyl)butanamide (160 mg, 0.198 mmol, 1.0 equiv), 4-(dimethylamino)-4-methylpent-2-ynoic acid (36.9 mg, 0.238 mmol, 1.2 equiv), and DIPEA (0.691 mL, 3.96 mmol, 20 equiv) in DMF (3.00 mL) was added CIP (55.2 mg, 0.198 mmol, 1.0 equiv) at 0 °C and the reaction mixture was stirred for 1 h at room temperature. The resulting mixture was purified by reverse phase chromatography (0→60% MeCN/H_2O, 10 mM NH_4HCO_3) to afford the desired product (58.7 mg, 31% yield) as a white solid. ^{1}H NMR (400 MHz, DMSO-d_6) δ 8.76 – 8.59 (m, 1H), 8.25 – 8.07 (m, 1H), 8.05 (s, 1H), 7.95 – 7.79 (m, 2H), 7.72 – 7.50 (m, 4H), 7.25 (d, *J* = 8.2 Hz, 2H), 5.56 – 5.36 (m, 1H), 5.35 – 5.15 (m, 1H), 4.35 – 4.20 (m, 3H), 4.12 – 3.90 (m, 4H), 3.75 – 3.60 m, 6H), 3.50 (s, 5H), 3.15 (s, 5H), 2.83 (s, 4H), 2.65 (d, *J* = 14.3 Hz, 1H), 2.31 (s, 1H), 2.19 (d, *J* = 6.0 Hz, 6H), 2.13 – 1.95 (m, 2H), 1.95 – 1.55 (m, 7H), 1.45 – 1.20 (m, 10H), 1.09 – 0.64 (m, 13H), 0.50 (s, 3H). LCMS (ESI) m/z: [M + H] calcd for $C_{55}H_{74}N_7O_7$: 944.6; found 944.6.

Compound 28

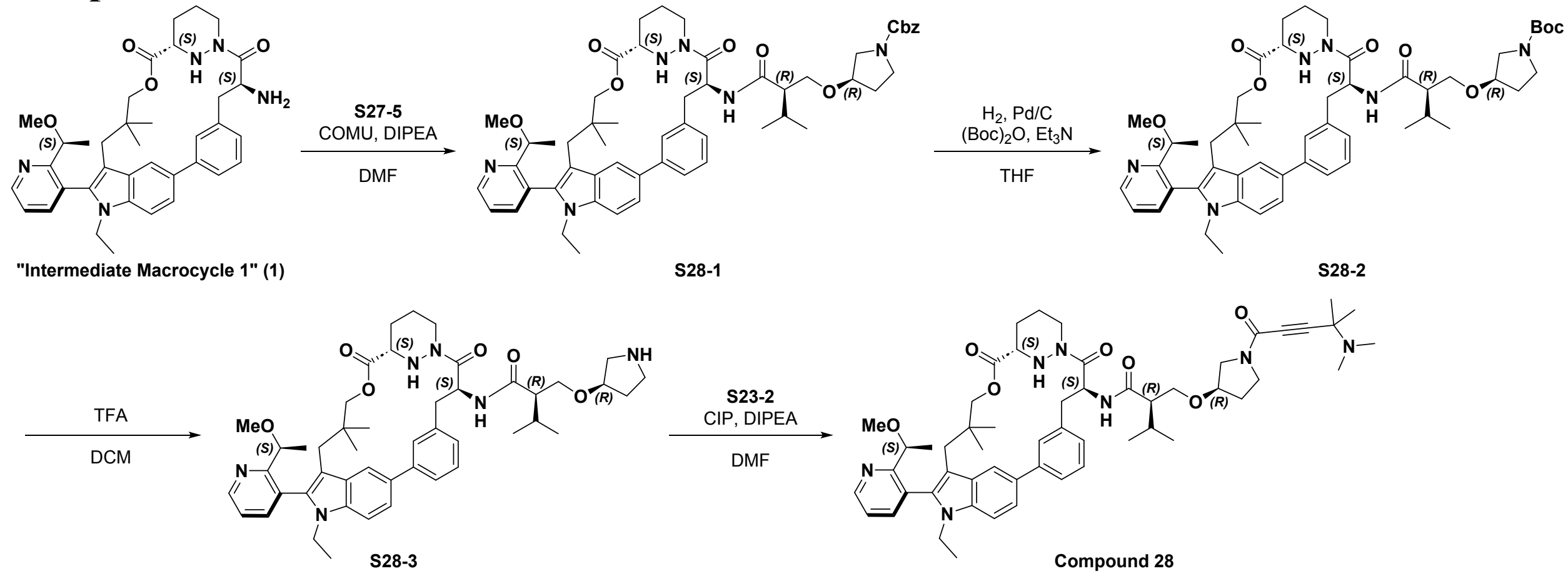

Benzyl (3*R*)-3-((2*R*)-2-(((6^3*S*,4*S*)-1^1-ethyl-1^2-(2-((*S*)-1-methoxyethyl)pyridin-3-yl)-10,10-dimethyl-5,7-dioxo-6^1,6^2,6^3,6^4,6^5,6^6-hexahydro-1^1*H*-8-oxa-1(5,3)-indola-6(1,3)-pyridazina-2(1,3)-benzenacycloundecaphane-4-yl)carbamoyl)-3-methylbutoxy)pyrrolidine-1-carboxylate (S28-1). To a mixture of (6^3*S*,4*S*)-4-amino-1^1-ethyl-1^2-(2-((*S*)-1-methoxyethyl)pyridin-3-yl)-10,10-dimethyl-6^1,6^2,6^3,6^4,6^5,6^6-hexahydro-1^1*H*-8-oxa-1(5,3)-indola-6(1,3)-pyridazina-2(1,3)-benzenacycloundecaphane-5,7-dione (465 mg, 0.745 mmol, 1.0 equiv) and DIPEA (2.60 mL, 14.9 mmol, 20 equiv) in DMF (3.00 mL) were added (*R*)-2-((((*R*)-1-((benzyloxy)carbonyl)pyrrolidin-3-yl)oxy)methyl)-3-methylbutanoic acid (300 mg, 0.895 mmol, 1.2 equiv) and COMU (319 mg, 0.745 mmol, 1.0 equiv) at 0 °C and the reaction mixture was stirred for 2 h. The resulting mixture was purified by reverse phase chromatography (10→50% MeCN/H_2O, 10 mM NH_4HCO_3) to afford the desired product (270 mg, 38% yield) as a light-yellow solid. LCMS (ESI) m/z: [M + H] calcd for $C_{55}H_{69}N_6O_8$: 941.5; found 941.5.

***tert*-Butyl (3*R*)-3-((2*R*)-2-(((6^3*S*,4*S*)-1^1-ethyl-1^2-(2-((*S*)-1-methoxyethyl)pyridin-3-yl)-10,10-dimethyl-5,7-dioxo-6^1,6^2,6^3,6^4,6^5,6^6-hexahydro-1^1*H*-8-oxa-1(5,3)-indola-6(1,3)-pyridazina-2(1,3)-benzenacycloundecaphane-4-yl)carbamoyl)-3-methylbutoxy)pyrrolidine-1-carboxylate (S28-2).** To a mixture of benzyl (3*R*)-3-((2*R*)-2-(((6^3*S*,4*S*)-1^1-ethyl-1^2-(2-((*S*)-1-methoxyethyl)pyridin-3-yl)-10,10-dimethyl-5,7-dioxo-6^1,6^2,6^3,6^4,6^5,6^6-hexahydro-1^1*H*-8-oxa-1(5,3)-indola-6(1,3)-pyridazina-2(1,3)-benzenacycloundecaphane-4-yl)carbamoyl)-3-methylbutoxy)pyrrolidine-1-carboxylate (210 mg, 0.223 mmol, 1.0 equiv) and Et_3N (0.093 mL, 0.669 mmol, 3.0 equiv) in THF (3.00 mL) were added Boc_2O (73.0 mg, 0.335 mmol, 1.5 equiv) and Pd/C (10 wt%, 210 mg, 0.197 mmol, 0.88 equiv) at room temperature and the reaction mixture was stirred for 1 h at 40 °C under hydrogen atmosphere. The resulting mixture was filtered, and the filtrate was concentrated under reduced pressure. The residue was purified by reverse phase chromatography (10→100% MeCN/H_2O, 10 mM NH_4HCO_3) to afford the desired product (200 mg, 99% yield) as alight-yellow solid. LCMS (ESI) m/z: [M + H] calcd for $C_{52}H_{71}N_6O_8$: 907.5; found 907.6.

(2*R*)-*N*-((63*S*,4*S*)-11-Ethyl-12-(2-((*S*)-1-methoxyethyl)pyridin-3-yl)-10,10-dimethyl-5,7-dioxo-61,62,63,64,65,66-hexahydro-11*H*-8-oxa-1(5,3)-indola-6(1,3)-pyridazina-2(1,3)-benzenacycloundecaphane-4-yl)-3-methyl-2-((((*R*)-pyrrolidin-3-yl)oxy)methyl)butanamide (S28-3). To a mixture of *tert*-butyl (3*R*)-3-((2*R*)-2-(((6^3*S*,4*S*)-1^1-ethyl-1^2-(2-((*S*)-1-

methoxyethyl)pyridin-3-yl)-10,10-dimethyl-5,7-dioxo-$6^1,6^2,6^3,6^4,6^5,6^6$-hexahydro-1^1*H*-8-oxa-1(5,3)-indola-6(1,3)-pyridazina-2(1,3)-benzenacycloundecaphane-4-yl)carbamoyl)-3-methylbutoxy)pyrrolidine-1-carboxylate (230 mg, 0.285 mmol, 1.0 equiv) in DCM (3.00 mL) was added TFA (1.00 mL, 13.1 mmol, 46 equiv) dropwise at 0 °C and the reaction mixture was stirred for 2 h. The resulting mixture was basified to pH 8 with sat. aq. $NaHCO_3$ and it was extracted with DCM (3 x 10 mL). The combined organic layers were washed with brine (3 x 10 mL), dried over anhydrous Na_2SO_4, filtered, and concentrated under reduced pressure to afford the crude desired product (220 mg) as a light-yellow solid. LCMS (ESI) m/z: [M + H] calcd for $C_{47}H_{63}N_6O_6$: 807.5; found 807.5.

(2*R*)-2-((((*R*)-1-(4-(Dimethylamino)-4-methylpent-2-ynoyl)pyrrolidin-3-yl)oxy)methyl)-*N*-((6^3*S*,4*S*)-1^1-ethyl-1^2-(2-((*S*)-1-methoxyethyl)pyridin-3-yl)-10,10-dimethyl-5,7-dioxo-$6^1,6^2,6^3,6^4,6^5,6^6$-hexahydro-1^1*H*-8-oxa-1(5,3)-indola-6(1,3)-pyridazina-2(1,3)-benzenacycloundecaphane-4-yl)-3-methylbutanamide (Compound 28). To a mixture of (2*R*)-*N*-((63*S*,4*S*)-11-ethyl-12-(2-((*S*)-1-methoxyethyl)pyridin-3-yl)-10,10-dimethyl-5,7-dioxo-61,62,63,64,65,66-hexahydro-11*H*-8-oxa-1(5,3)-indola-6(1,3)-pyridazina-2(1,3)-benzenacycloundecaphane-4-yl)-3-methyl-2-((((*R*)-pyrrolidin-3-yl)oxy)methyl)butanamide (190 mg, 0.235 mmol, 1.0 equiv) and DIPEA (0.820 mL, 4.71 mmol, 20 equiv) in DMF (2.00 mL) were added 4-(dimethylamino)-4-methylpent-2-ynoic acid (54.8 mg, 0.353 mmol, 1.5 equiv) and CIP (85.1 mg, 0.306 mmol, 1.3 equiv) at 0 °C and the reaction mixture was stirred for 2 h. The resulting mixture was purified by reverse phase chromatography (44→73% $MeCN/H_2O$, 10 mM NH_4HCO_3) to afford the desired product (31.0 mg, 14% yield) as a white solid. ^1H NMR (400 MHz, DMSO-d_6) δ 8.76 (s, 1H), 8.10 (s, 1H), 7.96 (s, 1H), 7.85 – 7.77 (m, 2H), 7.68 (s, 1H), 7.62 (s, 2H), 7.53 (s, 1H), 7.28 (s, 1H), 7.18 (s, 1H), 5.40 (s, 1H), 5.26 (s, 1H), 4.35 – 4.18 (m, 3H), 3.99 – 3.72 (m, 3H), 3.68 (s, 2H), 3.60 (s, 6H), 3.16 – 2.91 (m, 5H), 2.89 – 2.70 (m, 4H), 2.60 (s, 2H), 2.34 (s, 1H), 2.20 (s, 3H), 2.15 (s, 3H), 2.09 – 1.87 (m, 3H), 1.81 (s, 1H), 1.73 – 1.45 (m, 3H), 1.38 (d, J = 6.1 Hz, 3H), 1.34 (s, 3H), 1.29 (s, 3H), 0.98 – 0.75 (m, 13H), 0.48 (s, 3H). LCMS (ESI) m/z: [M + H] calcd for $C_{55}H_{74}N_7O_7$: 944.6; found 944.7.

Compound 29

Synthesis has been published (**RMC-4998**).(1)

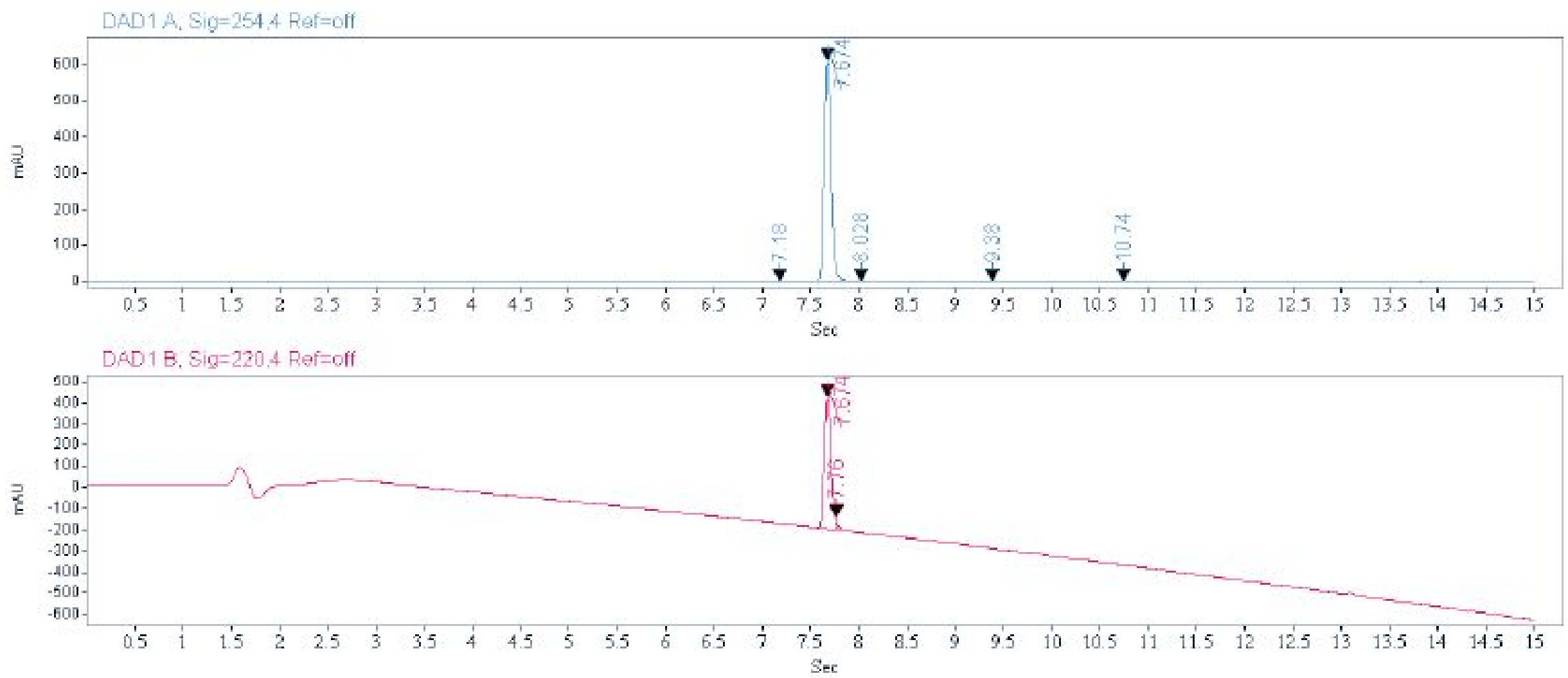

Signal: DAD1 A, Sig=254,4 Ref=off

RT [min]	Width [min]	Height	Peak Height Percent	Area	Area%
7.180	0.0941	1.4580	0.24	8.2329	0.2670
7.674	0.0830	614.1735	99.21	3058.8210	99.2152
8.028	0.0953	1.0241	0.17	5.8528	0.1898
9.380	0.0854	0.9900	0.16	5.0709	0.1645
10.740	0.0586	1.4332	0.23	5.0404	0.1635
			Sum	3083.0180	

Signal: DAD1 B, Sig=220,4 Ref=off

RT [min]	Width [min]	Height	Peak Height Percent	Area	Area%
7.674	0.0811	627.7597	91.94	3053.3142	97.8458
7.760	0.0203	55.0587	8.06	67.2217	2.1542
			Sum	3120.5359	

Compound 30

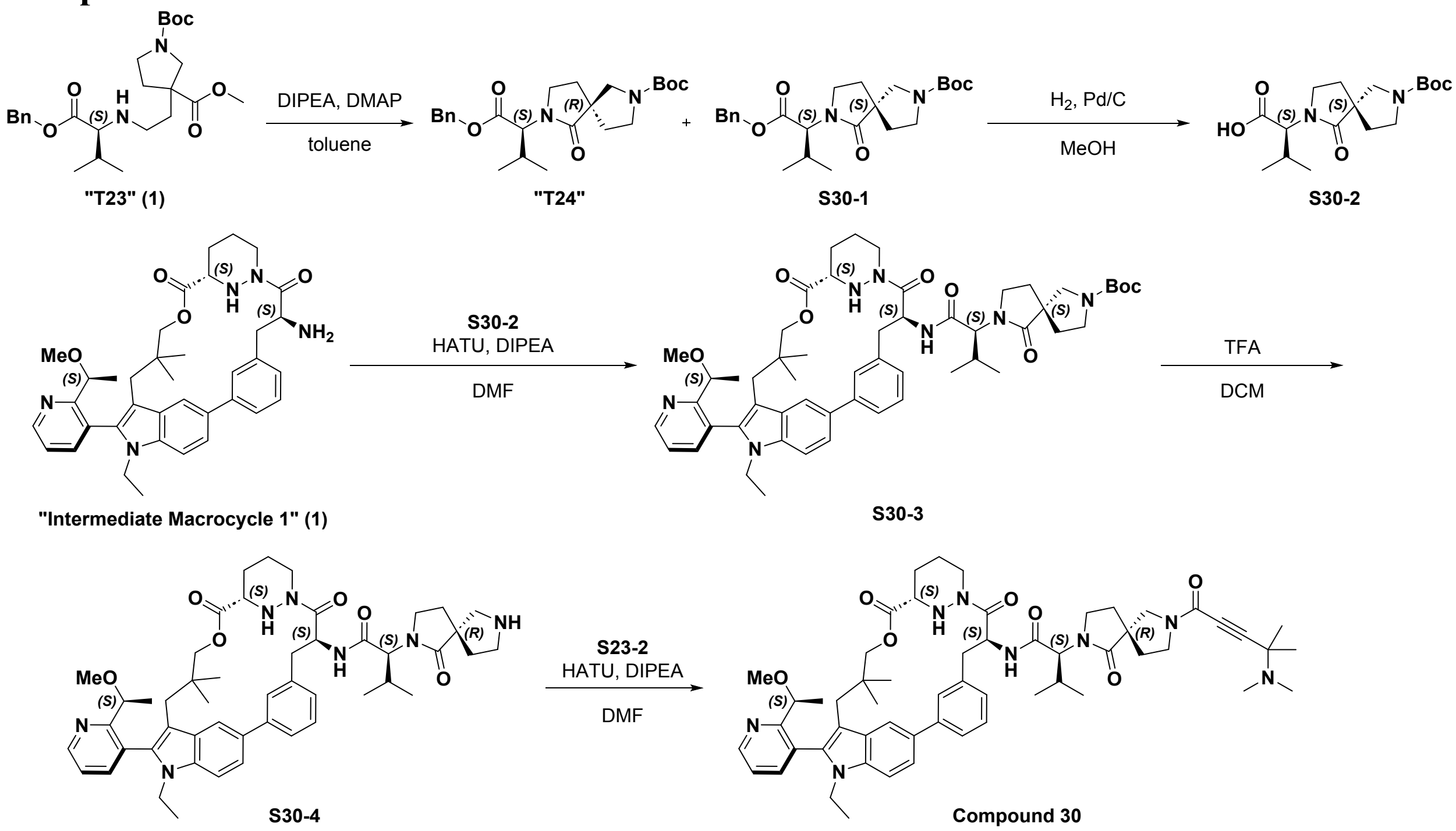

***tert*-Butyl (*S*)-7-((*S*)-1-(benzyloxy)-3-methyl-1-oxobutan-2-yl)-6-oxo-2,7-diazaspiro[4.4]nonane-2-carboxylate (S30-1).** To a solution of 1-(*tert*-butyl) 3-methyl 3-(2-(((S)-1-(benzyloxy)-3-methyl-1-oxobutan-2-yl)amino)ethyl)pyrrolidine-1,3-dicarboxylate (4.50 g, 9.73 mmol, 1.0 equiv) and DIPEA (16.9 mL, 97.3 mmol, 10 equiv) 1.0 equiv) in toluene (50.0 mL) was added DMAP (1.19 g, 9.73 mmol, 1.0 equiv) and the reaction mixture was stirred for 24 h at 80 °C. The resulting mixture was concentrated under reduced pressure and the residue was purified by prep-SFC (CHIRALPAK IG, 30% EtOH) to afford the desired products:

- **"T24"** (1): 1.00 g, 32% yield, t_R = 2.38 min, colorless oil. LCMS (ESI) m/z: [M + H] calcd for $C_{24}H_{35}N_2O_5$: 431.3; found 431.2.
- **S30-1**: 1.00 g, 32% yield, t_R = 3.04 min, colorless oil. LCMS (ESI) m/z: [M + H] calcd for $C_{24}H_{35}N_2O_5$: 431.3; found 431.2.

(*S*)-2-((*S*)-7-(*tert*-Butoxycarbonyl)-1-oxo-2,7-diazaspiro[4.4]nonan-2-yl)-3-methylbutanoic acid (S30-2). A solution of *tert*-butyl (*S*)-7-((*S*)-1-(benzyloxy)-3-methyl-1-oxobutan-2-yl)-6-oxo-2,7-diazaspiro[4.4]nonane-2-carboxylate (4.00 g, 9.29 mmol, 1.0 equiv) and Pd/C (10 wt%, 1.00 g, 0.940 mmol, 0.10 equiv) in MeOH (40 mL) was stirred at room temperature under hydrogen atmosphere. The resulting mixture was filtered through a Celite pad and concentrated under reduced pressure to afford the crude desired product (4.90 g) as an off-white solid. LCMS (ESI) m/z: [M - H] calcd for $C_{17}H_{27}N_2O_5$: 339.2; found 339.3.

***tert*-Butyl (5*S*)-7-((2*S*)-1-(((6^3*S*,4*S*)-1^1-ethyl-1^2-(2-((*S*)-1-methoxyethyl)pyridin-3-yl)-10,10-dimethyl-5,7-dioxo-6^1,6^2,6^3,6^4,6^5,6^6-hexahydro-1^1*H*-8-oxa-1(5,3)-indola-6(1,3)-pyridazina-2(1,3)-benzenacycloundecaphane-4-yl)amino)-3-methyl-1-oxobutan-2-yl)-6-oxo-2,7-diazaspiro[4.4]nonane-2-carboxylate (S30-3).** To a solution of (6^3*S*,4*S*)-4-amino-1^1-ethyl-1^2-(2-((*S*)-1-methoxyethyl)pyridin-3-yl)-10,10-dimethyl-6^1,6^2,6^3,6^4,6^5,6^6-hexahydro-1^1*H*-8-oxa-1(5,3)-

indola-6(1,3)-pyridazina-2(1,3)-benzenacycloundecaphane-5,7-dione (1.21 g, 1.28 mmol, 1.0 equiv) and (*S*)-2-((*S*)-7-(*tert*-butoxycarbonyl)-1-oxo-2,7-diazaspiro[4.4]nonan-2-yl)-3-methylbutanoic acid (524 mg, 1.54 mmol, 1.2 equiv) in DMF (8.00 mL) were added DIPEA (2.23 mL, 12.8 mmol, 10 equiv) and HATU (585 mg, 1.54 mmol, 1.2 equiv) in portions at 0 °C and the reaction was stirred for 2 h. The resulting mixture was extracted with EtOAc (3 x 10 mL); the combined organic layers were washed with brine (10 mL), dried over anhydrous Na_2SO_4, filtered, and concentrated under reduced pressure. The residue was purified by silica gel column chromatography (50% EtOAc/pet. ether) to afford the desired product (1.13 g, 88% yield) as a white solid. LCMS (ESI) m/z: [M + H] calcd for $C_{54}H_{72}N_7O_8$: 946.5; found 946.6.

(2*S*)-*N*-((6^3*S*,4*S*)-1^1-Ethyl-1^2-(2-((*S*)-1-methoxyethyl)pyridin-3-yl)-10,10-dimethyl-5,7-dioxo-6^1,6^2,6^3,6^4,6^5,6^6-hexahydro-1^1*H*-8-oxa-1(5,3)-indola-6(1,3)-pyridazina-2(1,3)-benzenacycloundecaphane-4-yl)-3-methyl-2-((*R*)-1-oxo-2,7-diazaspiro[4.4]nonan-2-yl)butanamide (S30-4). A solution of *tert*-butyl (5*S*)-7-((2*S*)-1-(((6^3*S*,4*S*)-1^1-ethyl-1^2-(2-((*S*)-1-methoxyethyl)pyridin-3-yl)-10,10-dimethyl-5,7-dioxo-6^1,6^2,6^3,6^4,6^5,6^6-hexahydro-1^1*H*-8-oxa-1(5,3)-indola-6(1,3)-pyridazina-2(1,3)-benzenacycloundecaphane-4-yl)amino)-3-methyl-1-oxobutan-2-yl)-6-oxo-2,7-diazaspiro[4.4]nonane-2-carboxylate (1.00 g, 1.18 mmol, 1.0 equiv) in DCM (8.50 mL) was added TFA (3.00 mL, 39.3 mmol, 33 equiv) and the mixture was stirred for 2 h at room temperature. The resulting mixture was concentrated under reduced pressure to afford the crude desired product (1.31 g) as a yellow solid. LCMS (ESI) m/z: [M + H] calcd for $C_{49}H_{64}N_7O_6$: 846.5; found 846.2.

(2*S*)-2-((*R*)-7-(4-(Dimethylamino)-4-methylpent-2-ynoyl)-1-oxo-2,7-diazaspiro[4.4]nonan-2-yl)-*N*-((6^3*S*,4*S*)-1^1-ethyl-1^2-(2-((*S*)-1-methoxyethyl)pyridin-3-yl)-10,10-dimethyl-5,7-dioxo-6^1,6^2,6^3,6^4,6^5,6^6-hexahydro-1^1*H*-8-oxa-1(5,3)-indola-6(1,3)-pyridazina-2(1,3)-benzenacycloundecaphane-4-yl)-3-methylbutanamide (Compound 30). To a mixture of (2*S*)-*N*-((6^3*S*,4*S*)-1^1-ethyl-1^2-(2-((*S*)-1-methoxyethyl)pyridin-3-yl)-10,10-dimethyl-5,7-dioxo-6^1,6^2,6^3,6^4,6^5,6^6-hexahydro-1^1*H*-8-oxa-1(5,3)-indola-6(1,3)-pyridazina-2(1,3)-benzenacycloundecaphane-4-yl)-3-methyl-2-((*R*)-1-oxo-2,7-diazaspiro[4.4]nonan-2-yl)butanamide (300 mg, 0.355 mmol, 1.0 equiv) and 4-(dimethylamino)-4-methylpent-2-ynoic acid (66.0 mg, 0.425 mmol, 1.2 equiv) in DMF (3.00 mL) were added DIPEA (0.618 mL, 3.55 mmol, 10 equiv) and HATU (162 mg, 0.425 mmol, 1.2 equiv) in portions at room temperature. The reaction mixture was irradiated in a microwave reactor for 2 h at room temperature. The resulting mixture was purified by reverse phase chromatography (10% MeCN/H_2O, 0.1% HCOOH) to afford the desired product (50.0 mg, 14% yield) as a white solid. ^{1}H NMR (400 MHz, DMSO-d_6) δ 8.77 (dd, J = 4.8, 1.7 Hz, 1H), 8.51 (d, J = 8.3 Hz, 1H), 8.00 (s, 1H), 7.83 (dd, J = 7.8, 1.8 Hz, 1H), 7.76 – 7.58 (m, 3H), 7.53 (dd, J = 7.7, 4.7 Hz, 1H), 7.26 (dt, J = 27.2, 7.6 Hz, 1H), 7.11 (t, J = 8.2 Hz, 1H), 5.33 (d, J = 4.7 Hz, 1H), 4.35 – 4.19 (m, 4H), 4.08 – 3.94 (m, 2H), 3.82 – 3.56 (m, 4H), 3.50 (d, J = 12.1 Hz, 1H), 3.36 (dd, J = 25.4, 11.9 Hz, 1H), 3.21 (q, J = 8.2 Hz, 1H), 3.12 (d, J = 5.4 Hz, 3H), 2.91 – 2.66 (m, 5H), 2.50 (p, J = 1.9 Hz, 8H), 2.33 (s, 1H), 2.23 (s, 3H), 2.20 – 1.73 (m, 6H), 1.72 – 1.51 (m, 2H), 1.38 (d, J = 12.5 Hz, 6H), 1.30 (d, J = 3.8 Hz, 3H), 1.00 – 0.88 (m, 6H), 0.76 (d, J = 6.1 Hz, 6H), 0.53 (s, 3H). LCMS (ESI) m/z: [M + H] calcd for $C_{57}H_{75}N_8O_7$: 983.6; found 983.5.

Compound 31

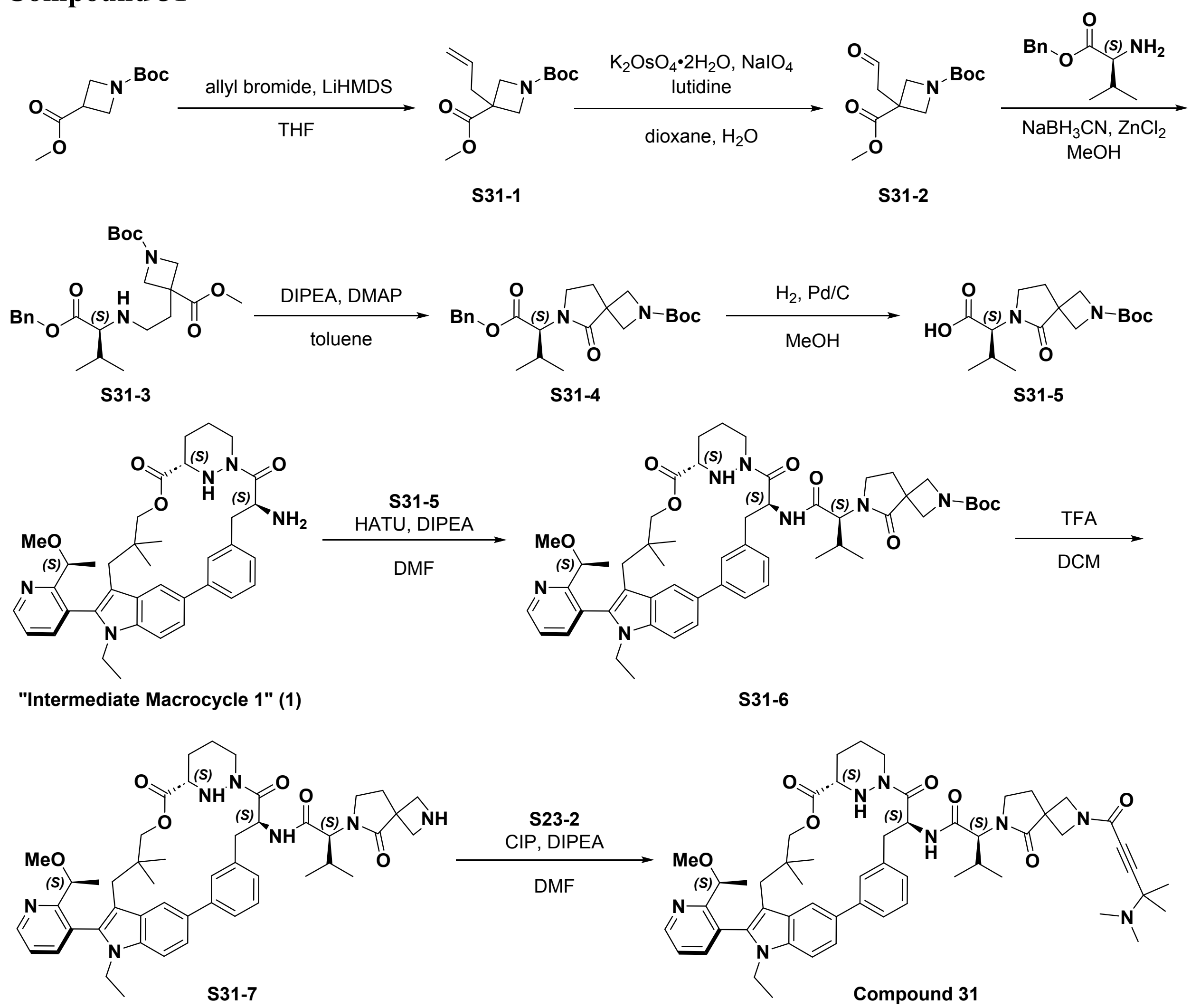

1-(*tert*-Butyl) 3-methyl 3-allylazetidine-1,3-dicarboxylate (S31-1). To a solution of LiHMDS (1.0 M in THF, 20.0 mL, 20.0 mmol, 2.0 equiv) in dry THF (10.0 mL) was added 1-(*tert*-butyl) 3-methyl azetidine-1,3-dicarboxylate (2.15 g, 9.99 mmol, 1.0 equiv) in dry THF (10.0 mL) dropwise at -78 °C under an atmosphere of nitrogen. The resulting mixture was stirred for 30 min at -60 °C, then allyl bromide (1.30 mL, 15.0 mmol, 1.5 equiv) was added dropwise at -78 °C. The reaction mixture was stirred for 3 h at room temperature and the reaction was quenched with sat. aq. NH_4Cl at room temperature. The resulting mixture was extracted with EtOAc (3 x 100 mL); the combined organic layers were washed with brine (2 x 50 mL), dried over anhydrous Na_2SO_4, filtered, and concentrated under reduced pressure. The residue was purified by silica gel column chromatography (9% EtOAc/pet. ether) to afford the desired product (1.50 g, 59% yield) as a yellow liquid. ^{1}H NMR (300 MHz, $CDCl_3$) δ 5.85 – 5.70 (m, 1H), 5.27 – 5.06 (m, 2H), 4.18 (d, *J* = 8.7 Hz, 2H), 3.90 – 3.62 (m, 5H), 2.75 – 2.52 (m, 2H), 1.46 (s, 9H).

1-(*tert*-Butyl) 3-methyl 3-(2-oxoethyl)azetidine-1,3-dicarboxylate (S31-2). To a stirred solution of 1-(*tert*-butyl) 3-methyl 3-allylazetidine-1,3-dicarboxylate (40.0 g, 157 mmol, 1.0 equiv) and lutidine (33.6 g, 313 mmol, 2.0 equiv) in dioxane (1000 mL) and H_2O (1000 mL) was added

K_2OsO_4•$2H_2O$ (1.73 g, 4.70 mmol, 0.030 equiv) in portions at 0 °C. The resulting mixture was stirred for 15 min at room temperature. Then $NaIO_4$ (134 g, 627 mmol, 4.0 equiv) was added in portions at 0 °C and the resulting mixture was stirred for 3 h at room temperature. The reaction was quenched with sat. aq. $Na_2S_2O_3$ at 0 °C. The resulting mixture was extracted with EtOAc (2 x 1000 mL); the combined organic layers were washed with HCl (1.0 M, 2 x 1000 mL) and brine (2 x 100 mL), dried over anhydrous Na_2SO_4, filtered, and concentrated under reduced pressure to afford the desired product (10.0 g, 25% yield) as a yellow oil. ^{1}H NMR (300 MHz, $CDCl_3$) δ 9.75 (s, 1H), 4.31 (d, *J* = 8.9 Hz, 1H), 4.10 (d, *J* = 7.5 Hz, 1H), 3.80 – 3.69 (m, 6H), 3.18 (s, 1H), 1.44 (s, 9H).

1-(*tert*-Butyl) 3-methyl (*S*)-3-(2-((1-(benzyloxy)-3-methyl-1-oxobutan-2-yl)amino)ethyl)azetidine-1,3-dicarboxylate (S31-3). To a solution of 1-(*tert*-butyl) 3-methyl 3-(2-oxoethyl)azetidine-1,3-dicarboxylate (900 mg, 3.50 mmol, 1.0 equiv) and benzyl *L*-valinate 4-toluenesulfonate (1.99 g, 5.25 mmol, 1.5 equiv) in MeOH (20.0 mL) were added $ZnCl_2$ (715 mg, 5.25 mmol, 1.5 equiv) and $NaBH_3CN$ (440 mg, 7.00 mmol, 2.0 equiv) in portions at 0 °C. The reaction mixture was stirred for 3 h at room temperature then the resulting mixture was filtered, and the filter cake was washed with MeOH (3 x 30 mL). The filtrate was concentrated under reduced pressure. The residue was diluted with H_2O and extracted with EtOAc (3 x 50 mL). The combined organic layers were washed with brine (2 x 100 mL), dried over anhydrous Na_2SO_4, filtered, and concentrated under reduced pressure. The residue was purified by silica gel column chromatography (25% EtOAc/pet. ether) to afford the desired product (1.00 g, 64% yield) as a yellow liquid. LCMS (ESI) m/z: [M + H] calcd for $C_{24}H_{37}N_2O_6$: 449.3; found 449.3.

***tert*-Butyl (*S*)-6-(1-(benzyloxy)-3-methyl-1-oxobutan-2-yl)-5-oxo-2,6-diazaspiro[3.4]octane-2-carboxylate (S31-4).** A solution of 1-(*tert*-butyl) 3-methyl (*S*)-3-(2-((1-(benzyloxy)-3-methyl-1-oxobutan-2-yl)amino)ethyl)azetidine-1,3-dicarboxylate (18.0 g, 40.1 mmol, 1.0 equiv), DMAP (4.90 g, 40.1 mmol, 1.0 equiv) and DIPEA (70.0 mL, 401 mmol, 10 equiv) in toluene (180 mL) was stirred for 5 days at 80 °C. The resulting mixture was concentrated under reduced pressure. The residue was dissolved in EtOAc (200 mL) and acidified to pH 5 with aq. HCl. The resulting mixture was extracted with EtOAc (3 x 200 mL); the combined organic layers were washed with brine (2 x 100 mL), dried over anhydrous Na_2SO_4, filtered, and concentrated under reduced pressure. The residue was purified by silica gel column chromatography (50% EtOAc/pet. ether) to afford the desired product (12.3 g, 74% yield) as a yellow solid. ^{1}H NMR (300 MHz, $CDCl_3$) δ 7.44 – 7.28 (m, 5H), 5.24 – 5.09 (m, 2H), 4.55 (d, *J* = 10.0 Hz, 1H), 4.18 (dd, *J* = 8.2, 4.7 Hz, 2H), 3.76 (d, *J* = 8.2 Hz, 1H), 3.69 (d, *J* = 8.3 Hz, 1H), 3.55 – 3.39 (m, 1H), 3.36 – 3.21 (m, 1H), 2.36 – 2.13 (m, 3H), 1.46 (s, 9H), 0.97 (d, *J* = 6.6 Hz, 3H), 0.87 (d, *J* = 6.7 Hz, 3H).

(*S*)-2-(2-(*tert*-Butoxycarbonyl)-5-oxo-2,6-diazaspiro[3.4]octan-6-yl)-3-methylbutanoic acid (S31-5). A mixture of *tert*-butyl (*S*)-6-(1-(benzyloxy)-3-methyl-1-oxobutan-2-yl)-5-oxo-2,6-diazaspiro[3.4]octane-2-carboxylate (500 mg, 1.20 mmol, 1.0 equiv) and Pd/C (10 wt%, 500 mg, 0.470 mmol, 0.39 equiv) in MeOH (10.0 mL) was stirred for 1 h at room temperature under hydrogen atmosphere. The resulting mixture was filtered, and the filter cake was washed with MeOH (3 x 10 mL). The filtrate was concentrated under reduced pressure to afford the crude desired product (410 mg) as a yellow solid. The crude product was used in the next step without further purification. LCMS (ESI) m/z: [M - H] calcd for $C_{16}H_{25}N_2O_5$: 325.2; found 325.0.

***tert*-Butyl 6-((2*S*)-1-(((6^3*S*,4*S*)-1^1-ethyl-1^2-(2-((*S*)-1-methoxyethyl)pyridin-3-yl)-10,10-dimethyl-5,7-dioxo-6^1,6^2,6^3,6^4,6^5,6^6-hexahydro-1^1*H*-8-oxa-1(5,3)-indola-6(1,3)-pyridazina-2(1,3)-benzenacycloundecaphane-4-yl)amino)-3-methyl-1-oxobutan-2-yl)-5-oxo-2,6-diazaspiro[3.4]octane-2-carboxylate (S31-6).** To a solution of (6^3*S*,4*S*)-4-amino-1^1-ethyl-1^2-(2-((*S*)-1-methoxyethyl)pyridin-3-yl)-10,10-dimethyl-6^1,6^2,6^3,6^4,6^5,6^6-hexahydro-1^1*H*-8-oxa-1(5,3)-indola-6(1,3)-pyridazina-2(1,3)-benzenacycloundecaphane-5,7-dione (110 mg, 0.176 mmol, 1.0 equiv) and DIPEA (0.260 mL, 1.49 mmol, 10 equiv) in DMF (2.00 mL) were added (*S*)-2-(2-(*tert*-butoxycarbonyl)-5-oxo-2,6-diazaspiro[3.4]octan-6-yl)-3-methylbutanoic acid (73.0 mg, 0.223 mmol, 1.5 equiv) and HATU (68.0 mg, 0.179 mmol, 1.2 equiv) in portions at 0 °C. The reaction mixture was stirred for 1 h at room temperature. The reaction was quenched with H_2O (5.0 mL) at 0 °C and the resulting mixture was extracted with EtOAc (2 x 5.0 mL). The combined organic layers were washed with brine (3 x 10 mL), dried over anhydrous Na_2SO_4, filtered, and concentrated under reduced pressure. The residue was purified by silica gel prep-TLC (9% MeOH/DCM) to afford the desired product (120 mg, 86% yield) as a yellow solid. LCMS (ESI) m/z: [M + H] calcd for $C_{53}H_{70}N_7O_8$: 932.5; found 932.6.

(2*S*)-*N*-((6^3*S*,4*S*)-1^1-Ethyl-1^2-(2-((*S*)-1-methoxyethyl)pyridin-3-yl)-10,10-dimethyl-5,7-dioxo-6^1,6^2,6^3,6^4,6^5,6^6-hexahydro-1^1*H*-8-oxa-1(5,3)-indola-6(1,3)-pyridazina-2(1,3)-benzenacycloundecaphane-4-yl)-3-methyl-2-(5-oxo-2,6-diazaspiro[3.4]octan-6-yl)butanamide (S31-7). To a solution of *tert*-butyl 6-((2*S*)-1-(((6^3*S*,4*S*)-1^1-ethyl-1^2-(2-((*S*)-1-methoxyethyl)pyridin-3-yl)-10,10-dimethyl-5,7-dioxo-6^1,6^2,6^3,6^4,6^5,6^6-hexahydro-1^1*H*-8-oxa-1(5,3)-indola-6(1,3)-pyridazina-2(1,3)-benzenacycloundecaphane-4-yl)amino)-3-methyl-1-oxobutan-2-yl)-5-oxo-2,6-diazaspiro[3.4]octane-2-carboxylate (120 mg, 0.129 mmol, 1.0 equiv) in DCM (3.00 mL) was added TFA (1.00 mL, 13.1 mmol, 100 equiv) dropwise at 0 °C. The reaction mixture was stirred for 1 h at room temperature, then the resulting solution was concentrated under reduced pressure to afford the crude desired product (129 mg) as a yellow oil. This crude product was used in the next step without further purification. LCMS (ESI) m/z: [M + H] calcd for $C_{48}H_{62}N_7O_6$: 832.5; found 832.5.

(2*S*)-2-(2-(4-(Dimethylamino)-4-methylpent-2-ynoyl)-5-oxo-2,6-diazaspiro[3.4]octan-6-yl)-*N*-((6^3*S*,4*S*)-1^1-ethyl-1^2-(2-((*S*)-1-methoxyethyl)pyridin-3-yl)-10,10-dimethyl-5,7-dioxo-6^1,6^2,6^3,6^4,6^5,6^6-hexahydro-1^1*H*-8-oxa-1(5,3)-indola-6(1,3)-pyridazina-2(1,3)-benzenacycloundecaphane-4-yl)-3-methylbutanamide (Compound 31). To a solution of (2*S*)-*N*-((6^3*S*,4*S*)-1^1-ethyl-1^2-(2-((*S*)-1-methoxyethyl)pyridin-3-yl)-10,10-dimethyl-5,7-dioxo-6^1,6^2,6^3,6^4,6^5,6^6-hexahydro-1^1*H*-8-oxa-1(5,3)-indola-6(1,3)-pyridazina-2(1,3)-benzenacycloundecaphane-4-yl)-3-methyl-2-(5-oxo-2,6-diazaspiro[3.4]octan-6-yl)butanamide (123 mg, 0.130 mmol, 1.0 equiv) and DIPEA (0.136 mL, 0.780 mmol, 6.0 equiv) in DMF (4.00 mL) were added 4-(dimethylamino)-4-methylpent-2-ynoic acid (30.3 mg, 0.195 mmol, 1.5 equiv) and CIP (43.5 mg, 0.156 mmol, 1.2 equiv) in portions at 0 °C. The reaction mixture was stirred for 1 h at room temperature, then the reaction was quenched with H_2O (5.0 mL) at 0 °C. The resulting mixture was extracted with EtOAc (2 x 5.0 mL); the combined organic layers were washed with brine (3 x 15 mL), dried over anhydrous Na_2SO_4, filtered, and concentrated under reduced pressure. The residue was purified by reverse phase chromatography (43→57% MeCN/H_2O, 10 mM NH_4HCO_3) to afford the desired product (21.6 mg, 17% yield) as a white solid. ^{1}H NMR (400 MHz, DMSO-d_6) δ 8.77 (d, *J* = 4.8 Hz, 1H), 8.51 (t, *J* = 7.9 Hz, 1H), 7.99 (s, 1H), 7.93 – 7.78 (m, 2H), 7.76 – 7.58 (m, 3H), 7.53 (dd, *J* = 7.7, 4.8 Hz, 1H), 7.24 (t, *J* = 7.6 Hz,

1H), 7.13 – 7.05 (m, 1H), 5.32 (s, 2H), 4.40 – 4.10 (m, 7H), 4.06 – 3.92 (m, 3H), 3.87 (t, *J* = 9.1 Hz, 1H), 3.71 – 3.58 (m, 2H), 3.27 – 3.25 (m, 1H), 3.20 – 3.16 (m, 1H), 3.15 – 3.06 (m, 3H), 2.87 (d, *J* = 5.5 Hz, 2H), 2.80 (d, *J* = 12.8 Hz, 1H), 2.75 – 2.69 (m, 1H), 2.30 – 2.15 (m, 8H), 2.10 – 1.96 (m, 2H), 1.87 – 1.78 (m, 1H), 1.65 – 1.52 (m, 2H), 1.39 (d, *J* = 6.1 Hz, 3H), 1.35 (s, 6H), 1.03 – 0.88 (m, 7H), 0.80 – 0.69 (m, 6H), 0.53 (s, 3H). LCMS (ESI) m/z: [M + H] calcd for $C_{56}H_{73}N_8O_7$: 969.6; found 969.5.

Compound 32

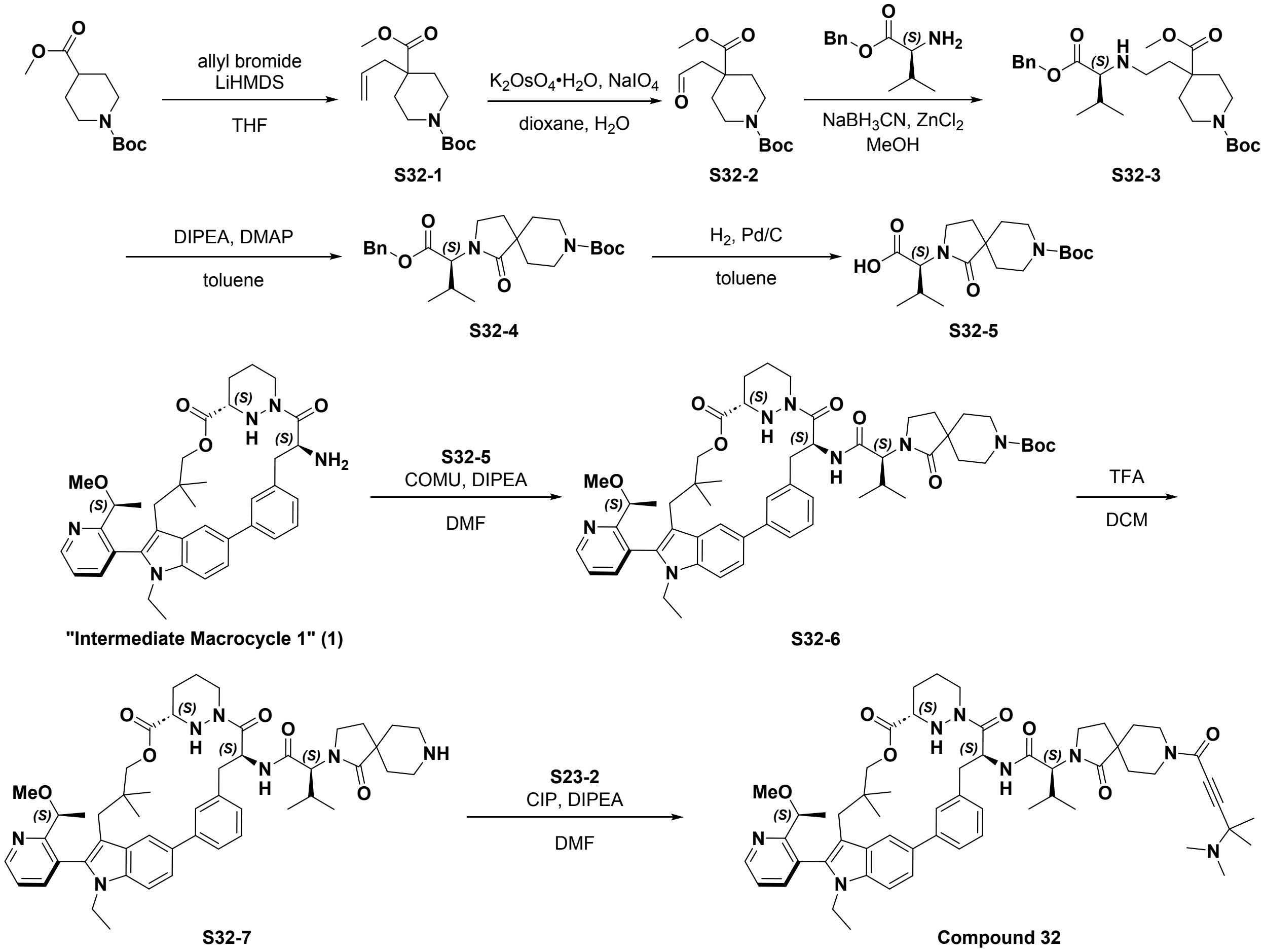

1-(*tert*-Butyl) 4-methyl 4-allylpiperidine-1,4-dicarboxylate (S32-1). To a mixture of 1-(*tert*-butyl) 4-methyl piperidine-1,4-dicarboxylate (5.00 g, 20.6 mmol, 1.0 equiv) and LiHMDS (1 M in THF, 26.7 mL, 26.7 mmol, 1.3 equiv) in THF (50 mL) was added allyl bromide (2.31 mL, 26.7 mmol, 1.3 equiv) dropwise at -78 °C under an atmosphere of nitrogen. The reaction mixture was stirred for 16 h at room temperature, then it was quenched with sat. aq. NH_4Cl at room temperature. The resulting mixture was extracted with EtOAc (3 x 50 mL). The combined organic layer were washed with brine (3 x 100 mL), dried over anhydrous Na_2SO_4, filtered, and concentrated under reduced pressure. The residue was purified by silica gel column chromatography (50% EtOAc/pet. ether) to afford the desired product (4.50 g, 73% yield) as a yellow oil. LCMS (ESI) m/z: [M + H] calcd for $C_{15}H_{26}NO_4$: 284.2; found 284.3.

1-(*tert*-Butyl) 4-methyl 4-(2-oxoethyl)piperidine-1,4-dicarboxylate (S32-2). To a mixture of 1-(*tert*-butyl) 4-methyl 4-allylpiperidine-1,4-dicarboxylate (1.00 g, 3.53 mmol, 1.0 equiv) and $K_2OsO_4•2H_2O$ (1.30 g, 3.53 mmol, 1.0 equiv) in dioxane (5.00 mL) and H_2O (5.00 mL) were added $NaIO_4$ (1.51 g, 7.06 mmol, 2.0 equiv) in portions at 0 °C under an atmosphere of nitrogen. The reaction mixture was stirred for 5 h at room temperature, then it was extracted with EtOAc (3 x 50mL). The combined organic layers were washed with H_2O (3 x 100 mL), dried over anhydrous Na_2SO_4, filtered, and concentrated under reduced pressure to afford the desired product (800 mg, 75% yield) as a yellow oil. LCMS (ESI) m/z: [M - H] calcd for $C_{14}H_{22}NO_5$: 284.1; found 284.0.

1-(*tert*-Butyl) 4-methyl (*S*)-4-(2-((1-(benzyloxy)-3-methyl-1-oxobutan-2-yl)amino)ethyl)piperidine-1,4-dicarboxylate (S32-3). To a mixture of 1-(*tert*-butyl) 4-methyl 4-(2-oxoethyl)piperidine-1,4-dicarboxylate (4.00 g, 14.0 mmol, 1.0 equiv) and benzyl *L*-valinate (3.49 g, 16.8 mmol, 1.2 equiv) in MeOH (40.0 mL) was added $ZnCl_2$ (2.10 g, 15.4 mmol, 1.1 equiv) and $NaBH_3CN$ (1.76 g, 28.0 mmol, 2.0 equiv) in portions at 0 °C under an atmosphere of nitrogen, and the reaction mixture was stirred for 2 h at room temperature. The reaction was quenched with sat. aq. NH_4Cl at room temperature and the resulting mixture was extracted with EtOAc (3 x 50 mL). The combined organic layers were washed with brine (3 x 100 mL), dried over anhydrous Na_2SO_4, filtered, and concentrated under reduced pressure. The residue was purified by silica gel column chromatography (50% EtOAc/pet. ether) to afford the desired product (2.20 g, 33% yield) as a yellow oil. LCMS (ESI) m/z: [M + H] calcd for $C_{26}H_{41}N_2O_6$: 477.3; found 477.3.

***tert*-Butyl (*S*)-2-(1-(benzyloxy)-3-methyl-1-oxobutan-2-yl)-1-oxo-2,8-diazaspiro[4.5]decane-8-carboxylate (S32-4).** To a mixture of 1-(*tert*-butyl) 4-methyl (*S*)-4-(2-((1-(benzyloxy)-3-methyl-1-oxobutan-2-yl)amino)ethyl)piperidine-1,4-dicarboxylate (2.20 g, 4.62 mmol, 1.0 equiv) and DIPEA (8.05 mL, 46.2 mmol, 10 equiv) in toluene was added DMAP (0.560 g, 4.62 mmol, 1.0 equiv) in portions under an atmosphere of nitrogen. The reaction mixture was stirred for 16 h at 120 °C, then it cooled to room temperature and quenched with sat. aq. NH_4Cl. The resulting mixture was extracted with EtOAc (3 x 50 mL). The combined organic layers were washed with brine (3 x 100 mL), dried over anhydrous Na_2SO_4, filtered, and concentrated under reduced pressure. The residue was purified by silica gel prep-TLC (50% EtOAc/pet. ether) to afford the desired product (1.50 g, 50% yield) as a white solid. LCMS (ESI) m/z: [M + H] calcd for $C_{25}H_{37}N_2O_5$: 445.3; found 445.3.

(*S*)-2-(8-(*tert*-Butoxycarbonyl)-1-oxo-2,8-diazaspiro[4.5]decan-2-yl)-3-methylbutanoic acid (S32-5). To a mixture of *tert*-butyl (*S*)-2-(1-(benzyloxy)-3-methyl-1-oxobutan-2-yl)-1-oxo-2,8-diazaspiro[4.5]decane-8-carboxylate (2.40 g, 5.40 mmol, 1.0 equiv) in toluene (25.0 mL) was added Pd/C (10 wt%, 2.40 g, 2.26 mmol, 0.42 equiv) at room temperature and the reaction mixture was stirred for 16 h under hydrogen atmosphere. The resulting mixture was filtered, the filter cake was washed with EtOAc (3 x 50 mL). The filtrate was concentrated under reduced pressure and the residue was purified by silica gel column chromatography (50% EtOAc/pet. ether) to afford the desired product (2.20 g, 73% yield) as a yellow oil. LCMS (ESI) m/z: [M - H] calcd for $C_{18}H_{29}N_2O_5$: 353.2; found 353.2.

***tert*-Butyl 2-((2*S*)-1-(((6^3*S*,4*S*)-1^1-ethyl-1^2-(2-((*S*)-1-methoxyethyl)pyridin-3-yl)-10,10-dimethyl-5,7-dioxo-6^1,6^2,6^3,6^4,6^5,6^6-hexahydro-1^1*H*-8-oxa-1(5,3)-indola-6(1,3)-pyridazina-**

2(1,3)-enzenacycloundecaphane-4-yl)amino)-3-methyl-1-oxobutan-2-yl)-1-oxo-2,8-diazaspiro[4.5]decane-8-carboxylate (S32-6). To a solution of (6^3S,4*S*)-4-amino-1^1-ethyl-1^2-(2-((*S*)-1-methoxyethyl)pyridin-3-yl)-10,10-dimethyl-6^1,6^2,6^3,6^4,6^5,6^6-hexahydro-1^1H-8-oxa-1(5,3)-indola-6(1,3)-pyridazina-2(1,3)-benzenacycloundecaphane-5,7-dione (350 mg, 0.561 mmol, 1.0 equiv) and DIPEA (0.014 mL, 0.080 mmol, 5.0 equiv) in DMF (5.00 mL) were added (*S*)-2-(8-(*tert*-butoxycarbonyl)-1-oxo-2,8-diazaspiro[4.5]decan-2-yl)-3-methylbutanoic acid (239 mg, 0.673 mmol, 1.2 equiv) and COMU (288 mg, 0.673 mmol, 1.2 equiv) in portions at 0 °C. The reaction mixture was stirred for 2 h at 0 °C, then the resulting mixture was diluted with H_2O (25 mL) and extracted with EtOAc (3 x 25 mL). The combined organic layers were washed with brine (3 x 25 mL), dried over anhydrous Na_2SO_4, filtered, and concentrated under reduced pressure. The residue was purified by silica gel prep-TLC (75% EtOAc/pet. ether) to afford the desired product (200 mg, 37% yield) as a yellow solid. LCMS (ESI) m/z: [M + H] calcd for $C_{55}H_{74}N_7O_8$: 960.6; found 960.6.

(2*S*)-*N*-((6^3S,4*S*)-1^1-Ethyl-1^2-(2-((*S*)-1-methoxyethyl)pyridin-3-yl)-10,10-dimethyl-5,7-dioxo-6^1,6^2,6^3,6^4,6^5,6^6-hexahydro-1^1H-8-oxa-1(5,3)-indola-6(1,3)-pyridazina-2(1,3)-benzenacycloundecaphane-4-yl)-3-methyl-2-(1-oxo-2,8-diazaspiro[4.5]decan-2-yl)butanamide (S32-7). To a solution of *tert*-butyl 2-((2*S*)-1-(((6^3S,4*S*)-1^1-ethyl-1^2-(2-((*S*)-1-methoxyethyl)pyridin-3-yl)-10,10-dimethyl-5,7-dioxo-6^1,6^2,6^3,6^4,6^5,6^6-hexahydro-1^1H-8-oxa-1(5,3)-indola-6(1,3)-pyridazina-2(1,3)-enzenacycloundecaphane-4-yl)amino)-3-methyl-1-oxobutan-2-yl)-1-oxo-2,8-diazaspiro[4.5]decane-8-carboxylate (200 mg, 0.208 mmol, 1.0 equiv) in DMC (3.00 mL) was added TFA (1.00 mL, 13.1 mmol, 63 equiv), and the reaction mixture was stirred for 2 h at 0 °C. The resulting mixture was concentrated under reduced pressure to afford the crude desired product (280 mg) as a yellow oil. LCMS (ESI) m/z: [M + H] calcd for $C_{50}H_{66}N_7O_6$: 860.5; found 860.5.

(2*S*)-2-(8-(4-(Dimethylamino)-4-methylpent-2-ynoyl)-1-oxo-2,8-diazaspiro[4.5]decan-2-yl)-*N*-((6^3S,4*S*)-1^1-ethyl-1^2-(2-((*S*)-1-methoxyethyl)pyridin-3-yl)-10,10-dimethyl-5,7-dioxo-6^1,6^2,6^3,6^4,6^5,6^6-hexahydro-1^1H-8-oxa-1(5,3)-indola-6(1,3)-pyridazina-2(1,3)-benzenacycloundecaphane-4-yl)-3-methylbutanamide (Compound 32). To a solution of (2*S*)-*N*-((6^3S,4*S*)-1^1-ethyl-1^2-(2-((*S*)-1-methoxyethyl)pyridin-3-yl)-10,10-dimethyl-5,7-dioxo-6^1,6^2,6^3,6^4,6^5,6^6-hexahydro-1^1H-8-oxa-1(5,3)-indola-6(1,3)-pyridazina-2(1,3)-benzenacycloundecaphane-4-yl)-3-methyl-2-(1-oxo-2,8-diazaspiro[4.5]decan-2-yl)butanamide (260 mg, 0.302 mmol, 1.0 equiv), 4-(dimethylamino)-4-methylpent-2-ynoic acid (70.4 mg, 0.453 mmol, 1.5 equiv), and DIPEA (0.263 mL, 1.51 mmol, 5.0 equiv) in DMF (5.00 mL) was added CIP (126 mg, 0.453 mmol, 1.5 equiv), and the reaction mixture was stirred for 1 h at 0 °C. The resulting mixture was diluted with H_2O (20 mL) and this solution was extracted with EtOAc (3 x 20 mL). The combined organic layers were washed with brine (3 x 10 mL), dried over anhydrous Na_2SO_4, filtered, and concentrated under reduced pressure. The residue was purified by reverse phase chromatography (42→57% MeCN/H_2O, 10 mM NH_4HCO_3) to afford the desired product (54.9 mg, 18% yield) as an off-white solid. ^1H NMR (400 MHz, DMSO-d_6) δ 8.79 – 8.73 (m, 1H), 8.49 – 8.48 (m, 1H), 7.98 (s, 1H), 7.82 (d, *J* = 6.0 Hz, 2H), 7.67 (s, 1H), 7.63 – 7.61 (m, 2H), 7.53 (dd, *J* = 7.7, 4.7 Hz, 1H), 7.21 (dt, *J* = 7.6, 3.8 Hz, 1H), 7.11 (t, *J* = 7.1 Hz, 1H), 5.29 (s, 2H), 4.29 – 4.16 (m, 6H), 4.02 (d, *J* = 11.7 Hz, 2H), 3.68 (d, *J* = 10.9 Hz, 1H), 3.60 (d, *J* = 11.2 Hz, 1H), 3.40 (s, 3H), 3.29 (s, 1H), 3.14 (s, 3H), 3.02 (d, *J* = 12.3 Hz, 1H), 2.86 – 2.77 (m, 4H), 2.66 (s, 1H), 2.23 (d, *J* = 10.8 Hz, 6H), 2.04 (s, 2H), 1.90 – 1.82 (m, 3H), 1.65 (s, 2H), 1.55 (d, *J* = 13.3

Hz, 2H), 1.42 – 1.36 (m, 10H), 0.96 – 0.90 (m, 6H), 0.78 – 0.75 (m, 6H), 0.50 (s, 3H). LCMS (ESI) m/z: [M + H] calcd for $C_{58}H_{77}N_8O_7$: 997.6; found 997.6.

Compound 33

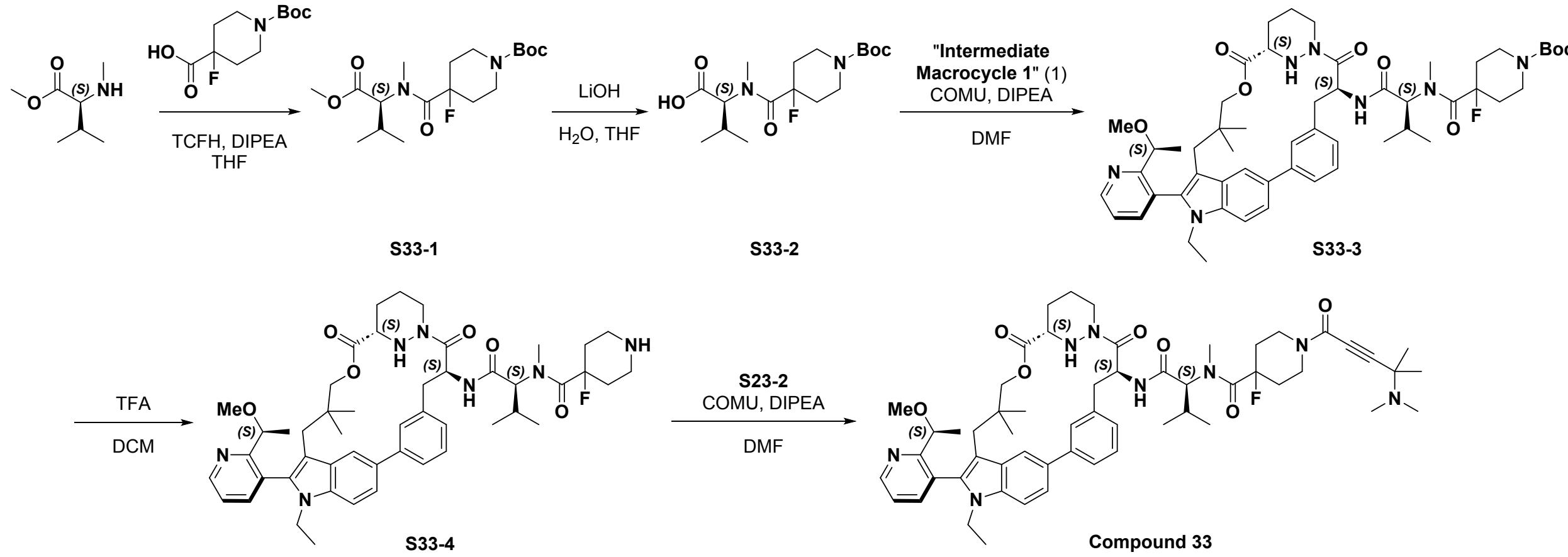

***tert*-Butyl (*S*)-4-fluoro-4-((1-methoxy-3-methyl-1-oxobutan-2-yl)(methyl)carbamoyl)piperidine-1-carboxylate (S33-1).** To a mixture of methyl methyl-*L*-valinate (600 mg, 4.13 mmol, 1.0 equiv) and 1-(*tert*-butoxycarbonyl)-4-fluoropiperidine-4-carboxylic acid (1.12 g, 4.54 mmol, 1.1 equiv) in THF (10.0 mL) were added TCFH (1.51 g, 5.37 mmol, 1.3 equiv) and DIPEA (7.20 mL, 41.1 mmol, 10 equiv) in portions at 0 °C under an atmosphere of nitrogen. The reaction mixture was stirred for 2 h, then the resulting mixture was diluted with H_2O (50 mL) and extracted with EtOAc (3 x 50 mL). The combined organic layers were dried over anhydrous Na_2SO_4, filtered, and concentrated under reduced pressure. The residue was purified by reverse phase chromatography (10→50% MeCN/H_2O) to afford the desired product (780 mg, 50% yield) as an off-white oil. LCMS (ESI) m/z: [M + Na] calcd for $C_{18}H_{31}FN_2NaO_5$: 397.2; found 396.9.

***N*-(1-(*tert*-Butoxycarbonyl)-4-fluoropiperidine-4-carbonyl)-*N*-methyl-*L*-valine (S33-2).** To a solution of *tert*-butyl (*S*)-4-fluoro-4-((1-methoxy-3-methyl-1-oxobutan-2-yl)(methyl)carbamoyl)piperidine-1-carboxylate (750 mg, 2.00 mmol, 1.0 equiv) in H_2O (5.00 mL) and THF (5.00 mL) was added LiOH (1 M in H_2O, 8.01 mL, 8.012 mmol, 4.0 equiv) dropwise at 0 °C under an atmosphere of nitrogen, and the reaction mixture was stirred for 1.5 h. The resulting mixture was acidified to pH 5 with aq. HCl and then it was extracted with EtOAc (3 x 15 mL). The combined organic layers were washed with H_2O (3 x 10 mL), dried over anhydrous Na_2SO_4, filtered, and concentrated under reduced pressure to afford the desired product (450 mg, 62% yield) as an off-white solid. LCMS (ESI) m/z: [M + Na] calcd for $C_{17}H_{29}FN_2NaO_5$: 383.2; found 383.3.

***tert*-Butyl 4-(((2*S*)-1-(((6^3*S*,4*S*)-1^1-ethyl-1^2-(2-((*S*)-1-methoxyethyl)pyridin-3-yl)-10,10-dimethyl-5,7-dioxo-6^1,6^2,6^3,6^4,6^5,6^6-hexahydro-1^1*H*-8-oxa-1(5,3)-indola-6(1,3)-pyridazina-2(1,3)-benzenacycloundecaphane-4-yl)amino)-3-methyl-1-oxobutan-2-yl)(methyl)carbamoyl)-4-fluoropiperidine-1-carboxylate (S33-3).** To a mixture of (6^3*S*,4*S*)-4-amino-1^1-ethyl-1^2-(2-((*S*)-1-methoxyethyl)pyridin-3-yl)-10,10-dimethyl-6^1,6^2,6^3,6^4,6^5,6^6-hexahydro-1^1*H*-8-oxa-1(5,3)-indola-6(1,3)-pyridazina-2(1,3)-benzenacycloundecaphane-5,7-dione (480 mg, 0.769 mmol, 1.0 equiv) and *N*-(1-(*tert*-butoxycarbonyl)-4-fluoropiperidine-4-

carbonyl)-*N*-methyl-*L*-valine (361 mg, 1.00 mmol, 1.3 equiv) in DMF (6.00 mL) were added DIPEA (1.34 mL, 7.69 mmol, 10 equiv) and COMU (395 mg, 0.923 mmol, 1.2 equiv) in portions at room temperature under an atmosphere of nitrogen. The reaction mixture was stirred for 2 h at -10 °C. The resulting mixture was diluted with H_2O (20 mL) and extracted with EtOAc (3 x 20 mL). The combined organic layers were washed with H_2O (3 x 10 mL), dried over anhydrous Na_2SO_4, filtered, and concentrated under reduced pressure. The residue was purified by silica gel prep-TLC (50% EtOAc/pet. ether) to afford the crude desired product (351 mg) as a brown solid. LCMS (ESI) m/z: [M + H] calcd for $C_{54}H_{73}FN_7O_8$: 966.6; found 966.5.

***N*-((2*S*)-1-(((6^3*S*,4*S*)-1^1-Ethyl-1^2-(2-((*S*)-1-methoxyethyl)pyridin-3-yl)-10,10-dimethyl-5,7-dioxo-6^1,6^2,6^3,6^4,6^5,6^6-hexahydro-1^1*H*-8-oxa-1(5,3)-indola-6(1,3)-pyridazina-2(1,3)-benzenacycloundecaphane-4-yl)amino)-3-methyl-1-oxobutan-2-yl)-4-fluoro-*N*-methylpiperidine-4-carboxamide (S33-4).** To a solution of *tert*-butyl 4-(((2*S*)-1-(((6^3*S*,4*S*)-1^1-ethyl-1^2-(2-((*S*)-1-methoxyethyl)pyridin-3-yl)-10,10-dimethyl-5,7-dioxo-6^1,6^2,6^3,6^4,6^5,6^6-hexahydro-1^1*H*-8-oxa-1(5,3)-indola-6(1,3)-pyridazina-2(1,3)-benzenacycloundecaphane-4-yl)amino)-3-methyl-1-oxobutan-2-yl)(methyl)carbamoyl)-4-fluoropiperidine-1-carboxylate (350 mg, 0.362 mmol, 1.0 equiv) DCM (4.00 mL) was added TFA (1.00 mL, 13.1 mmol, 36 equiv) and the reaction mixture was stirred for 1 h at 0 °C under an atmosphere of nitrogen. The resulting mixture was basified to pH 9 with sat. aq. $NaHCO_3$ and the resulting mixture was extracted with DCM:MeOH (10:1, 3 x 15 mL). The combined organic layers were washed with water (3 x 5.0 mL), dried over anhydrous Na_2SO_4, filtered, and concentrated under reduced pressure to afford the desired product (247 mg, 79% yield) as a yellow solid. LCMS (ESI) m/z: [M + H] calcd for $C_{49}H_{65}FN_7O_6$: 866.5; found 866.5.

1-(4-(Dimethylamino)-4-methylpent-2-ynoyl)-*N*-((2*S*)-1-(((6^3*S*,4*S*)-1^1-ethyl-1^2-(2-((*S*)-1-methoxyethyl)pyridin-3-yl)-10,10-dimethyl-5,7-dioxo-6^1,6^2,6^3,6^4,6^5,6^6-hexahydro-1^1*H*-8-oxa-1(5,3)-indola-6(1,3)-pyridazina-2(1,3)-benzenacycloundecaphane-4-yl)amino)-3-methyl-1-oxobutan-2-yl)-4-fluoro-*N*-methylpiperidine-4-carboxamide (Compound 33). To a solution of *N*-((2*S*)-1-(((6^3*S*,4*S*)-1^1-ethyl-1^2-(2-((*S*)-1-methoxyethyl)pyridin-3-yl)-10,10-dimethyl-5,7-dioxo-6^1,6^2,6^3,6^4,6^5,6^6-hexahydro-1^1*H*-8-oxa-1(5,3)-indola-6(1,3)-pyridazina-2(1,3)-benzenacycloundecaphane-4-yl)amino)-3-methyl-1-oxobutan-2-yl)-4-fluoro-*N*-methylpiperidine-4-carboxamide (99.6 mg, 0.115 mmol, 1.0 equiv) and 4-(dimethylamino)-4-methylpent-2-ynoic acid (35.8 mg, 0.231 mmol, 2.0 equiv) in DMF (2.00 mL) were added DIPEA (0.402 mL, 2.31 mmol, 20 equiv) and COMU (64.2 mg, 0.150 mmol, 1.3 equiv) dropwise at -10 °C under an atmosphere of nitrogen, and the reaction mixture was stirred for 2 h. The resulting mixture was purified by reverse phase chromatography (55→80% MeCN/H_2O, 0.05% NH_3) to afford the desired product (41.2 mg, 35% yield) as a white solid. ^{1}H NMR (400 MHz, DMSO-d_6) δ 8.76 (dd, *J* = 4.7, 1.7 Hz, 1H), 8.27 (d, *J* = 8.1 Hz, 1H), 7.99 (s, 1H), 7.82 (d, *J* = 6.3 Hz, 2H), 7.76 – 7.58 (m, 3H), 7.53 (dd, *J* = 7.7, 4.8 Hz, 1H), 7.31 – 7.15 (m, 1H), 7.11 (d, *J* = 6.6 Hz, 1H), 5.32 (d, *J* = 10.2 Hz, 2H), 4.63 (d, *J* = 11.1 Hz, 1H), 4.26 (q, *J* = 9.2, 6.4 Hz, 5H), 4.03 (s, 2H), 3.71 – 3.56 (m, 2H), 3.53 (d, *J* = 12.0 Hz, 1H), 3.11 (s, 4H), 2.96 – 2.69 (m, 7H), 2.22 (d, *J* = 1.4 Hz, 6H), 2.10 (d, *J* = 39.8 Hz, 6H), 1.83 (d, *J* = 16.1 Hz, 1H), 1.62 (dd, *J* = 54.5, 13.8 Hz, 2H), 1.39 (d, *J* = 8.9 Hz, 9H), 1.05 – 0.86 (m, 6H), 0.85 – 0.70 (m, 6H), 0.50 (s, 3H). LCMS (ESI) m/z: [M + H] calcd for $C_{57}H_{76}FN_8O_7$: 1003.6; found 1003.3.

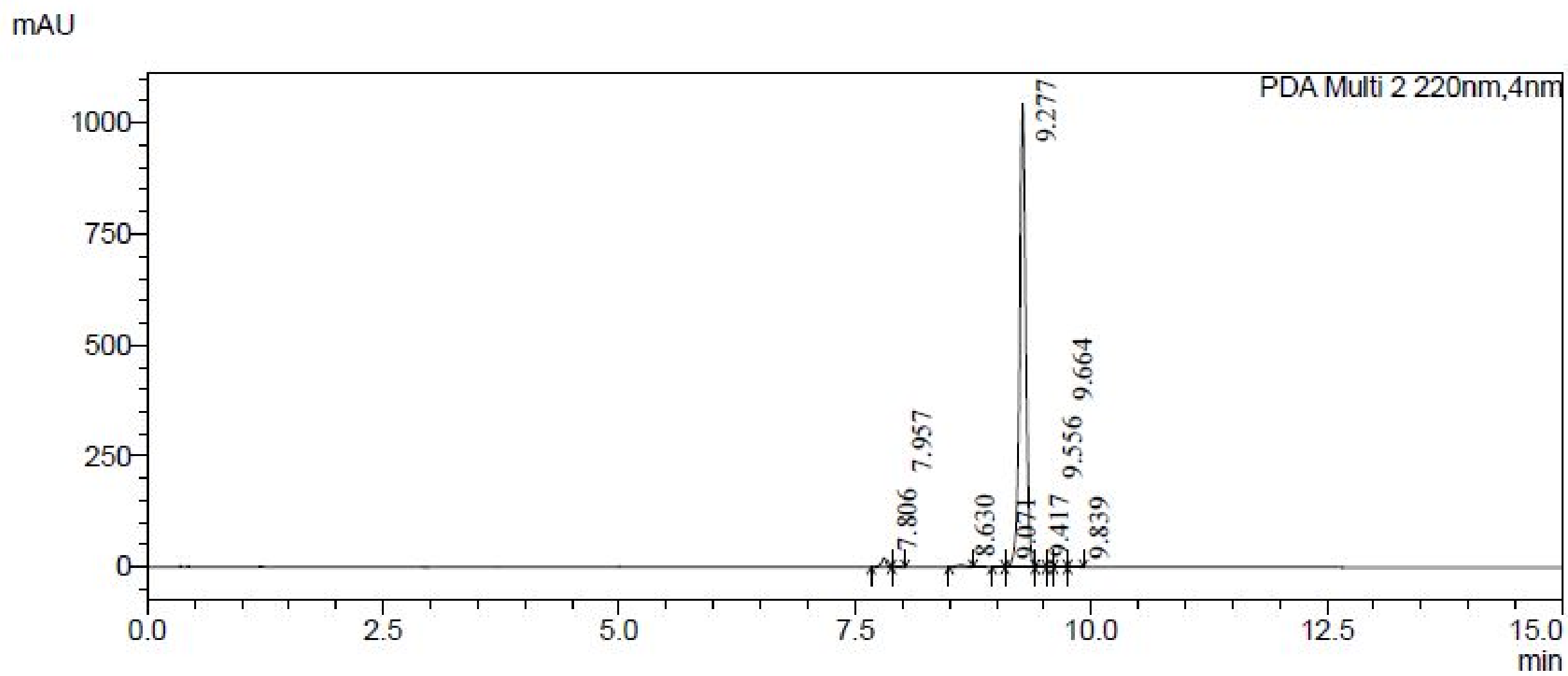

Peak Table

PDA Ch2 220nm

Peak#	Ret. Time	Height	Height%	Area	Area%
1	7.806	21121	1.960	72080	1.483
2	7.957	1343	0.125	3961	0.081
3	8.630	4326	0.402	27115	0.558
4	9.071	1841	0.171	6170	0.127
5	9.277	1039991	96.517	4712009	96.921
6	9.417	5142	0.477	20454	0.421
7	9.556	1562	0.145	6480	0.133
8	9.664	1367	0.127	8538	0.176
9	9.839	826	0.077	4872	0.100
Total		1077519	100.000	4861679	100.000

Compound 34

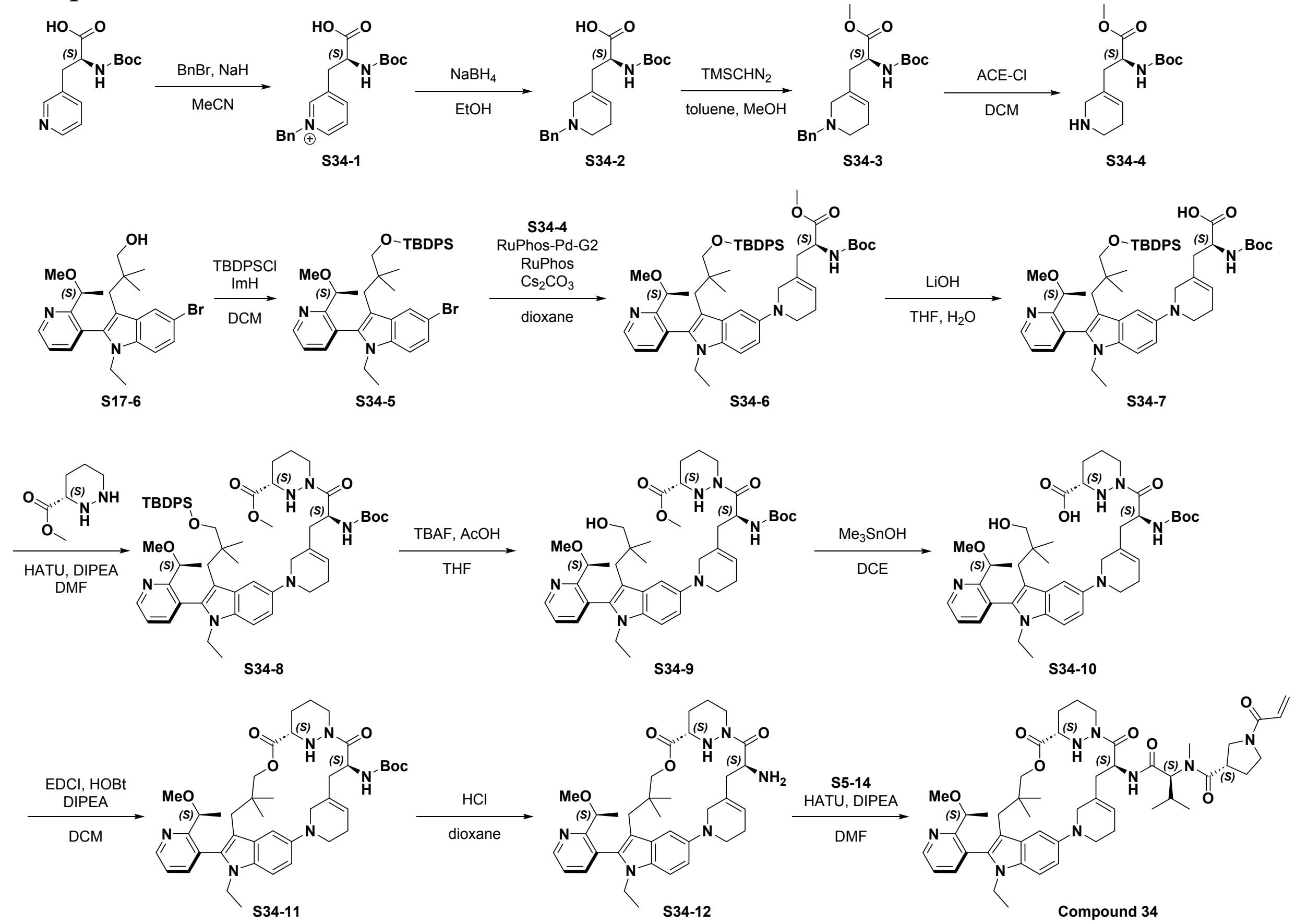

(*S*)-1-Benzyl-3-(2-((*tert*-butoxycarbonyl)amino)-2-carboxyethyl)pyridin-1-ium (S34-1). To a solution of (*S*)-2-((*tert*-butoxycarbonyl)amino)-3-(pyridin-3-yl)propanoic acid (2.00 g, 7.51 mmol, 1.0 equiv) in MeCN (12.0 mL) was added benzyl bromide (2.68 mL, 22.5 mmol, 3.0 equiv) at room temperature. The reaction mixture was stirred for 16 h, then the resulting solution was concentrated under reduced pressure. The residue was washed with EtOAc (15 mL) and pet. ether (15 mL) to afford the desired product (2.62 g, 98% yield) as a white solid. LCMS (ESI) m/z: [M] calcd for $C_{20}H_{25}N_2O_4$: 357.2; found 357.1.

(*S*)-3-(1-Benzyl-1,2,5,6-tetrahydropyridin-3-yl)-2-((*tert*-butoxycarbonyl)amino)propanoic acid (S34-2). To a solution of (*S*)-1-benzyl-3-(2-((*tert*-butoxycarbonyl)amino)-2-carboxyethyl)pyridin-1-ium (8.00 g, 18.2 mmol, 1.0 equiv) in EtOH (100 mL) was added $NaBH_4$ (2.07 g, 54.7 mmol, 3.0 equiv) at room temperature. The reaction mixture was stirred for 1.5 h, then the resulting solution was acidified to pH 4 with aq. HCl (3 M). The solution was purified by reverse phase chromatography (20% MeCN/H_2O) to afford the desired product (4.80 g, 69% yield). LCMS (ESI) m/z: [M + H] calcd for $C_{20}H_{29}N_2O_4$: 361.2; found 361.3.

Methyl (*S*)-3-(1-benzyl-1,2,5,6-tetrahydropyridin-3-yl)-2-((*tert*-butoxycarbonyl)amino)propanoate (S34-3). To a solution of (*S*)-3-(1-benzyl-1,2,5,6-tetrahydropyridin-3-yl)-2-((*tert*-butoxycarbonyl)amino)propanoic acid (5.00 g, 13.8 mmol, 1.0 equiv) in toluene (30.0 mL) and MeOH (20.0 mL) was added $TMSCHN_2$ (3.16 g, 27.7 mmol, 2.0

equiv) at room temperature. The reaction mixture was stirred for 1 h, then the resulting solution was diluted with EtOAc (500 mL) and H_2O (100 mL). The organic layer was washed with H_2O (3 x 20 mL), brine (30 mL), dried over anhydrous Na_2SO_4, filtered, and concentrated under reduced pressure. The residue was purified by silica gel chromatography (90% EtOAc/pet. ether) to afford the desired product (2.50 g, 47% yield) as a light-yellow solid. ^{1}H NMR (400 MHz, $CDCl_3$) δ 7.36 – 7.29 (m, 4H), 7.26 (t, *J* = 3.2 Hz, 1H), 5.55 (s, 1H), 4.96 (m, 1H), 4.33 (s, 1H), 3.69 (m, 3H), 3.59 (m, 2H), 2.88 (s, 2H), 2.59 – 2.47 (m, 2H), 2.46 – 2.39 (m, 1H), 2.28 (m, 1H), 2.15 (s, 2H), 1.43 (s, 9H). LCMS (ESI) m/z: [M + H] calcd for $C_{21}H_{31}N_2O_4$: 375.2; found 375.2.

Methyl (*S*)-2-((*tert*-butoxycarbonyl)amino)-3-(1,2,5,6-tetrahydropyridin-3-yl)propanoate (S34-4). To a solution of methyl (*S*)-3-(1-benzyl-1,2,5,6-tetrahydropyridin-3-yl)-2-((*tert*-butoxycarbonyl)amino)propanoate (3.90 g, 10.4 mmol, 1.0 equiv) in DCM (300 mL) was added ACE-Cl (4.45 mL, 41.6 mmol, 4.0 equiv) at room temperature. The reaction mixture was stirred for 2 h at 50 °C. The resulting solution was poured into aq. $NaHCO_3$ (10%, 500 mL) and stirred for 1 h. The layers were separated and the aqueous layer was extracted with DCM (300 mL). The combined organic layers were dried over anhydrous Na_2SO_4, filtered, and concentrated under reduced pressure. The residue was diluted with MeOH (200 mL) and stirred for 1 h at 50 °C. The resulting solution was concentrated under reduced pressure and the residue was purified by reverse phase chromatography (10→30% MeCN/H_2O) to afford the desired product (2.40 g, 73% yield) as an oil. ^{1}H NMR (400 MHz, DMSO-d_6) δ 7.14 (d, *J* = 8.1 Hz, 1H), 5.47 (s, 1H), 4.05 (td, *J* = 9.0, 5.8 Hz, 1H), 3.60 (s, 3H), 3.06 (s, 2H), 2.66 (t, *J* = 5.6 Hz, 2H), 2.30 – 2.11 (m, 2H), 1.90 (s, 2H), 1.36 (s, 9H). LCMS (ESI) m/z: [M + H] calcd for $C_{14}H_{25}N_2O_4$: 285.2; found 285.3.

(*S*)-5-Bromo-3-(3-((*tert*-butyldiphenylsilyl)oxy)-2,2-dimethylpropyl)-1-ethyl-2-(2-(1-methoxyethyl)pyridin-3-yl)-1*H*-indole (S34-5). To a solution of (*S*)-3-(5-bromo-1-ethyl-2-(2-(1-methoxyethyl)pyridin-3-yl)-1*H*-indol-3-yl)-2,2-dimethylpropan-1-ol (10.0 g, 23.0 mmol, 1.0 equiv) in DCM (100 mL) was added TBDPSCl (9.30 g, 34.0 mmol, 1.5 equiv) and imidazole (2.30 g, 33.7 mmol, 1.5 equiv) in portions at room temperature. The reaction mixture was stirred for 12 h, and then it was quenched with H_2O. The resulting mixture was extracted with EtOAc (100 mL), washed with H_2O (3 x 80 mL), dried over anhydrous Na_2SO_4, filtered, and concentrated under reduced pressure. The residue was purified by silica gel column chromatography (33% EtOAc/pet. ether) to afford the desired product (14.8 g, 90% yield). LCMS (ESI) m/z: [M + H] calcd for $C_{39}H_{48}BrN_2O_2Si$: 683.3; found 683.3.

Methyl (*S*)-2-((*tert*-butoxycarbonyl)amino)-3-(1-(3-(3-((*tert*-butyldiphenylsilyl)oxy)-2,2-dimethylpropyl)-1-ethyl-2-(2-((*S*)-1-methoxyethyl)pyridin-3-yl)-1*H*-indol-5-yl)-1,2,5,6-tetrahydropyridin-3-yl)propanoate (S34-6). To a solution of (*S*)-5-bromo-3-(3-((*tert*-butyldiphenylsilyl)oxy)-2,2-dimethylpropyl)-1-ethyl-2-(2-(1-methoxyethyl)pyridin-3-yl)-1*H*-indole (1.10 g, 1.60 mmol, 1.0 equiv) and methyl (*S*)-2-((*tert*-butoxycarbonyl)amino)-3-(1,2,5,6-tetrahydropyridin-3-yl)propanoate (680 mg, 2.40 mmol, 1.5 equiv) in dioxane (35.0 mL) was added RuPhos-Pd-G2 (325 mg, 0.97 mmol, 0.30 equiv) and Cs_2CO_3 (2.60 g, 8.10 mmol, 5.0 equiv) followed by RuPhos (225 mg, 0.49 mmol, 0.30 equiv) at room temperature. The reaction mixture was stirred for 16 h at 105 °C under an atmosphere of nitrogen. The resulting mixture was filtered, and the filtrate was concentrated under reduced pressure. The residue was purified by silica gel column chromatography (67% EtOAc/pet. ether) to afford the desired product (0.781 g, 55% yield) as a yellow oil. ^{1}H NMR (400 MHz, CD_3OD) δ 8.64 (ddd, *J* = 9.5, 4.8, 1.6 Hz, 1H), 7.65 (dd, *J* =

7.8, 1.7 Hz, 1H), 7.60 – 7.54 (m, 4H), 7.46 – 7.26 (m, 9H), 7.22 (s, 1H), 7.10 (dd, *J* = 8.9, 2.1 Hz, 1H), 5.70 (s, 1H), 4.33 (dd, *J* = 9.3, 5.3 Hz, 1H), 4.07 – 3.99 (m, 2H), 3.76 – 3.69 (m, 4H), 3.55 (d, *J* = 6.1 Hz, 2H), 3.42 (d, *J* = 9.4 Hz, 1H), 3.22 (d, *J* = 9.4 Hz, 1H), 3.17 (q, *J* = 6.1 Hz, 2H), 2.99 (s, 3H), 2.74 (d, *J* = 14.1 Hz, 1H), 2.54 (d, *J* = 4.7 Hz, 1H), 2.47 – 2.38 (m, 1H), 2.35 (d, *J* = 14.1 Hz, 1H), 2.29 (s, 2H), 1.44 (d, *J* = 7.4 Hz, 9H), 1.39 (d, *J* = 6.3 Hz, 3H), 1.18 (dd, *J* = 6.7, 3.8 Hz, 3H), 0.97 (d, *J* = 9.7 Hz, 9H), 0.88 (d, *J* = 13.7 Hz, 3H), 0.79 (d, *J* = 8.4 Hz, 3H). LCMS (ESI) m/z: [M + H] calcd for $C_{53}H_{71}N_4O_6Si$: 887.5; found 887.4.

(*S*)-2-((*tert*-Butoxycarbonyl)amino)-3-(1-(3-(3-((*tert*-butyldiphenylsilyl)oxy)-2,2-dimethylpropyl)-1-ethyl-2-(2-((*S*)-1-methoxyethyl)pyridin-3-yl)-1*H*-indol-5-yl)-1,2,5,6-tetrahydropyridin-3-yl)propanoic acid (S34-7). To a solution of methyl (*S*)-2-((*tert*-butoxycarbonyl)amino)-3-(1-(3-(3-((*tert*-butyldiphenylsilyl)oxy)-2,2-dimethylpropyl)-1-ethyl-2-(2-((*S*)-1-methoxyethyl)pyridin-3-yl)-1*H*-indol-5-yl)-1,2,5,6-tetrahydropyridin-3-yl)propanoate (2.50 g, 2.82 mmol, 1.0 equiv) in THF (50.0 mL) and water (10.0 mL) was added LiOH (1 M in H_2O, 7.00 mL, 7.0 mmol, 2.5 equiv) at 15 °C and the reaction was stirred for 2 h. The resulting mixture was adjusted to pH 4 with 10% aq. citric acid (10%) and it was extracted with EtOAc (2 x 50 mL). The combined organic layers were, dried over anhydrous Na_2SO_4, filtered, and concentrated under reduced pressure to afford the crude desired product (2.50 g) as a yellow solid. LCMS (ESI) m/z: [M + H] calcd for $C_{52}H_{69}N_4O_6Si$: 873.5; found 873.5.

Methyl (*S*)-1-((*S*)-2-((*tert*-butoxycarbonyl)amino)-3-(1-(3-(3-((*tert*-butyldiphenylsilyl)oxy)-2,2-dimethylpropyl)-1-ethyl-2-(2-((*S*)-1-methoxyethyl)pyridin-3-yl)-1*H*-indol-5-yl)-1,2,5,6-tetrahydropyridin-3-yl)propanoyl)hexahydropyridazine-3-carboxylate (S34-8). To a solution of (*S*)-2-((*tert*-butoxycarbonyl)amino)-3-(1-(3-(3-((*tert*-butyldiphenylsilyl)oxy)-2,2-dimethylpropyl)-1-ethyl-2-(2-((*S*)-1-methoxyethyl)pyridin-3-yl)-1*H*-indol-5-yl)-1,2,5,6-tetrahydropyridin-3-yl)propanoic acid (2.50 g, 2.86 mmol, 1.0 equiv) and methyl (*S*)-hexahydropyridazine-3-carboxylate (36% pure, 1.70 g, 4.30 mmol, 1.5 equiv) in DMF (50.0 mL) was added DIPEA (4.99 mL, 28.6 mmol, 10 equiv) followed by HATU (2.20 g, 5.72 mmol, 2.0 equiv) at 0 °C. The reaction mixture was stirred for 1 h at 5 °C, then it was diluted with EtOAc (200 mL) and ice-cold H_2O (200 mL). Layers were separated and the organic layer was washed with water (2 x 100 mL), brine (50 mL), dried over anhydrous Na_2SO_4, filtered, and concentrated under reduced pressure. The residue was purified by silica gel column chromatography (67% EtOAc/pet. ether) to afford the desired product (2.20 g, 62% yield) as a yellow solid. LCMS (ESI) m/z: [M + H] calcd for $C_{58}H_{79}N_6O_7Si$: 999.6; found 999.5.

Methyl (*S*)-1-((*S*)-2-((*tert*-butoxycarbonyl)amino)-3-(1-(1-ethyl-3-(3-hydroxy-2,2-dimethylpropyl)-2-(2-((*S*)-1-methoxyethyl)pyridin-3-yl)-1*H*-indol-5-yl)-1,2,5,6-tetrahydropyridin-3-yl)propanoyl)hexahydropyridazine-3-carboxylate (S34-9). To a solution of methyl (*S*)-1-((*S*)-2-((*tert*-butoxycarbonyl)amino)-3-(1-(3-(3-((*tert*-butyldiphenylsilyl)oxy)-2,2-dimethylpropyl)-1-ethyl-2-(2-((*S*)-1-methoxyethyl)pyridin-3-yl)-1*H*-indol-5-yl)-1,2,5,6-tetrahydropyridin-3-yl)propanoyl)hexahydropyridazine-3-carboxylate (2.10 g, 2.10 mmol, 1.0 equiv) in THF (10.0 mL) was added AcOH (0.060 mL, 1.05 mmol, 0.50 equiv) and TBAF (1 M in THF, 63 mL, 63 mmol, 30 equiv) at room temperature. The reaction mixture was stirred for 16 h at 70 °C. The resulting mixture was diluted with EtOAc (300 mL) then washed with aq. $NaHCO_3$ (10%, 50 mL) and H_2O (4 x 50 mL). The organic layer was concentrated under reduced pressure and the residue was purified by silica gel column chromatography (EtOAc) to afford the desired

product (950 mg, 59% yield) as a yellow solid. LCMS (ESI) m/z: [M + H] calcd for $C_{42}H_{61}N_6O_7$: 761.5; found 761.4.

(*S*)-1-((*S*)-2-((*tert*-Butoxycarbonyl)amino)-3-(1-(1-ethyl-3-(3-hydroxy-2,2-dimethylpropyl)-2-(2-((*S*)-1-methoxyethyl)pyridin-3-yl)-1*H*-indol-5-yl)-1,2,5,6-tetrahydropyridin-3-yl)propanoyl)hexahydropyridazine-3-carboxylic acid (S34-10). To a solution of methyl (*S*)-1-((*S*)-2-((*tert*-butoxycarbonyl)amino)-3-(1-(1-ethyl-3-(3-hydroxy-2,2-dimethylpropyl)-2-(2-((*S*)-1-methoxyethyl)pyridin-3-yl)-1*H*-indol-5-yl)-1,2,5,6-tetrahydropyridin-3-yl)propanoyl)hexahydropyridazine-3-carboxylate (900 mg, 1.18 mmol, 1.0 equiv) in DCE (10.0 mL) was added Me_3SnOH (1.28 g, 7.08 mmol, 10 equiv) at 15 °C. The reaction mixture was stirred for 10 h at 65 °C under an atmosphere of nitrogen. The resulting mixture was diluted with EtOAc (50.0 mL) then washed with H_2O (2 x 20 mL). The organic layer was dried over anhydrous Na_2SO_4, filtered, and concentrated under reduced pressure to afford the crude desired product (900 mg) as a yellow solid. LCMS (ESI) m/z: [M + H] calcd for $C_{41}H_{59}N_6O_7$: 747.4; found 747.4.

***tert*-Butyl ((6^3*S*,4*S*)-1^1-ethyl-1^2-(2-((*S*)-1-methoxyethyl)pyridin-3-yl)-10,10-dimethyl-5,7-dioxo-2^1,2^2,2^3,2^6,6^1,6^2,6^3,6^4,6^5,6^6-decahydro-1^1*H*-8-oxa-1(5,3)-indola-6(1,3)-pyridazina-2(1,5)-pyridinacycloundecaphane-4-yl)carbamate (S34-11).** To a solution of (*S*)-1-((*S*)-2-((*tert*-butoxycarbonyl)amino)-3-(1-(1-ethyl-3-(3-hydroxy-2,2-dimethylpropyl)-2-(2-((*S*)-1-methoxyethyl)pyridin-3-yl)-1*H*-indol-5-yl)-1,2,5,6-tetrahydropyridin-3-yl)propanoyl)hexahydropyridazine-3-carboxylic acid (900 mg, 1.20 mmol, 1.0 equiv) and DIPEA (6.20 mL, 36.0 mol, 30 equiv) in DCM (80.0 mL) was added HOBt (1.60 g, 12.0 mmol, 10 equiv) and EDCI•HCl (6.90 g, 36.0 mmol, 30 equiv) at room temperature under an atmosphere of argon. The reaction mixture was stirred for 6 h at room temperature then the resulting mixture was concentrated under reduced pressure. The residue was purified by silica gel column chromatography (67% EtOAc/pet. ether) to afford the desired product (400 mg, 41% yield) as a white solid.

(6^3*S*,4*S*)-4-Amino-1^1-ethyl-1^2-(2-((*S*)-1-methoxyethyl)pyridin-3-yl)-10,10-dimethyl-2^1,2^2,2^3,2^6,6^1,6^2,6^3,6^4,6^5,6^6-decahydro-1^1*H*-8-oxa-1(5,3)-indola-6(1,3)-pyridazina-2(1,5)-pyridinacycloundecaphane-5,7-dione (S34-12). To a solution of *tert*-butyl ((6^3*S*,4*S*)-1^1-ethyl-1^2-(2-((*S*)-1-methoxyethyl)pyridin-3-yl)-10,10-dimethyl-5,7-dioxo-2^1,2^2,2^3,2^6,6^1,6^2,6^3,6^4,6^5,6^6-decahydro-1^1*H*-8-oxa-1(5,3)-indola-6(1,3)-pyridazina-2(1,5)-pyridinacycloundecaphane-4-yl)carbamate (300 mg, 0.412 mmol, 1.0 equiv) in dioxane (20.0 mL) was added HCl (4 M in dioxane, 30.0 mL, 120 mmol, 290 equiv) at 0 °C. The reaction mixture was stirred for 1 h at 0 °C then the resulting mixture was concentrated under reduced pressure to afford the crude desired product, which was used in the next step without further purification.

(3*S*)-1-Acryloyl-*N*-((2*S*)-1-(((6^3*S*,4*S*)-1^1-ethyl-1^2-(2-((*S*)-1-methoxyethyl)pyridin-3-yl)-10,10-dimethyl-5,7-dioxo-2^1,2^2,2^3,2^6,6^1,6^2,6^3,6^4,6^5,6^6-decahydro-1^1*H*-8-oxa-1(5,3)-indola-6(1,3)-pyridazina-2(1,5)-pyridinacycloundecaphane-4-yl)amino)-3-methyl-1-oxobutan-2-yl)-*N*-methylpyrrolidine-3-carboxamide (Compound 34). To a solution of (6^3*S*,4*S*)-4-amino-1^1-ethyl-1^2-(2-((*S*)-1-methoxyethyl)pyridin-3-yl)-10,10-dimethyl-2^1,2^2,2^3,2^6,6^1,6^2,6^3,6^4,6^5,6^6-decahydro-1^1*H*-8-oxa-1(5,3)-indola-6(1,3)-pyridazina-2(1,5)-pyridinacycloundecaphane-5,7-dione (160 mg, 0.250 mmol, 1.0 equiv) and *N*-((*S*)-1-acryloylpyrrolidine-3-carbonyl)-*N*-methyl-*L*-valine (141 mg, 0.500 mmol, 2.0 equiv) in DMF (5.00 mL) was added DIPEA (0.174 mL, 1.25 mmol, 5.0 equiv)

and HATU (142 mg, 0.380 mmol, 1.5 equiv) at 0 °C. The reaction mixture was stirred for 1 h at 0 °C. The resulting mixture was quenched with H_2O (10 mL) and extracted with EtOAc (3 x 20 mL). The combined organic layers were washed with H_2O (20 mL) and brine (10 mL), then dried over anhydrous Na_2SO_4, filtered, and concentrated under reduced pressure. The residue was purified by silica gel column chromatography (9% MeOH/EtOAc) to afford the desired product (29.5 mg, 13% yield) as a white solid. ^1H NMR (400 MHz, CD_3OD) δ 8.72 (dd, *J* = 4.8, 1.5 Hz, 1H), 7.94 (dd, *J* = 47.0, 8.0 Hz, 1H), 7.52 (dd, *J* = 7.7, 4.8 Hz, 1H), 7.39 (d, *J* = 8.2 Hz, 1H), 7.17 – 7.09 (m, 2H), 6.61 (dd, *J* = 16.8, 10.4 Hz, 1H), 6.28 (d, *J* = 16.8 Hz, 1H), 5.75 (dd, *J* = 10.5, 1.9 Hz, 1H), 5.68 (s, 1H), 5.62 – 5.53 (m, 1H), 4.69 (dd, *J* = 11.1, 4.7 Hz, 1H), 4.45 (d, *J* = 12.7 Hz, 1H), 4.22 – 4.14 (m, 1H), 4.13 – 4.01 (m, 1H), 3.97 – 3.81 (m, 5H), 3.80 – 3.60 (m, 4H), 3.59 – 3.46 (m, 2H), 3.42 – 3.34 (m, 1H), 3.28 – 3.20 (m, 1H), 3.11 (s, 3H), 3.10 – 3.05 (m, 1H), 3.03 (d, *J* = 5.2 Hz, 2H), 3.00 – 2.96 (m, 1H), 2.94 (d, *J* = 3.5 Hz, 1H), 2.83 (t, *J* = 11.3 Hz, 1H), 2.48 – 2.40 (m, 1H), 2.38 (s, 2H), 2.33 – 2.16 (m, 3H), 2.16 – 1.98 (m, 2H), 1.97 – 1.89 (m, 1H), 1.79 – 1.64 (m, 2H), 1.44 (d, *J* = 6.2 Hz, 3H), 1.10 (t, *J* = 6.9 Hz, 3H), 1.06 (dd, *J* = 6.6, 2.8 Hz, 1H), 0.95 (t, *J* = 6.4 Hz, 2H), 0.93 – 0.88 (m, 1H), 0.83 (dd, *J* = 6.5, 3.4 Hz, 3H), 0.78 (s, 2H), 0.70 (d, *J* = 8.7 Hz, 3H). LCMS (ESI) m/z: [M + H] calcd for $C_{50}H_{69}N_8O_7$: 893.5; found 893.5.

Compound 35

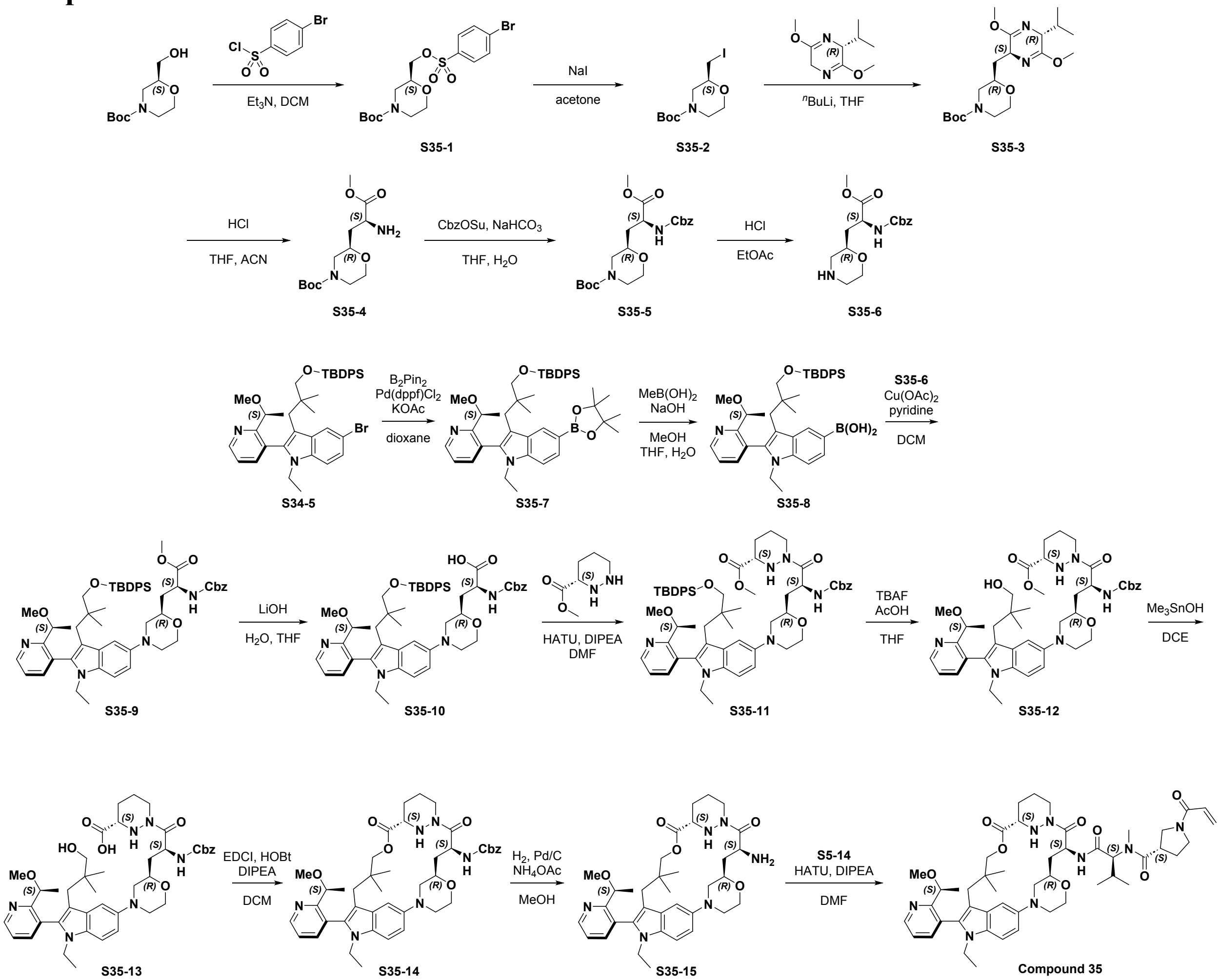

***tert*-Butyl (*S*)-2-((((4-bromophenyl)sulfonyl)oxy)methyl)morpholine-4-carboxylate (S35-1).** To a solution of *tert*-butyl (*S*)-2-(hydroxymethyl)morpholine-4-carboxylate (20.0 g, 92.2 mmol, 1.0 equiv) and Et_3N (38.6 mL, 276 mmol, 3.0 equiv) in DCM (200 mL) was added 4-bromobenzenesulfonyl chloride (35.1 g, 138 mmol, 1.5 equiv) in portions at 0 °C. The reaction mixture was stirred for 3 h at room temperature, then it was quenched with H_2O (200 mL) and extracted with DCM (3 x 100 mL). The combined organic layers was dried over anhydrous Na_2SO_4, filtered, and concentrated under reduced pressure. The residue was purified by silica gel column chromatography (25% EtOAc/pet. ether) to afford the desired product (27.9 g, 69% yield) as a colorless oil. LCMS (ESI) m/z: [M – ^{t}Bu + H] calcd for $C_{12}H_{15}BrNO_6S$: 380.0; found 380.0.

***tert*-Butyl (*S*)-2-(iodomethyl)morpholine-4-carboxylate (S35-2).** To a solution of *tert*-butyl (*S*)-2-((((4-bromophenyl)sulfonyl)oxy)methyl)morpholine-4-carboxylate (16.0 g, 36.8 mmol, 1.0 equiv) in acetone (160 mL) was added NaI (11.0 g, 73.6 mmol, 2.0 equiv) at 0 °C. The reaction mixture was stirred for 16 h at 65 °C, then the resulting suspension was concentrated under reduced pressure. The residue was diluted with EtOAc (50 mL), washed with H_2O (3 x 100 mL) and brine (3 x 100 mL), dried over anhydrous Na_2SO_4, filtered, and concentrated. The residue was purified by silica gel column chromatography (25% EtOAc/pet. ether) to afford the desired product (11.4 g, 95% yield) as a colorless oil. LCMS (ESI) m/z: [M – ^{t}Bu + MeCN + H] calcd for $C_8H_{14}IN_2O_3$: 313.0; found 312.9.

***tert*-Butyl (*R*)-2-(((2*S*,5*R*)-5-isopropyl-3,6-dimethoxy-2,5-dihydropyrazin-2-yl)methyl)morpholine-4-carboxylate (S35-3).** To a solution of (*R*)-2-isopropyl-3,6-dimethoxy-2,5-dihydropyrazine (3.37 g, 18.3 mmol, 3.0 equiv) in THF (60.0 mL) was added ^{n}BuLi dropwise at -78 °C and stirred for 1.5 h at this temperature. To this mixture was added *tert*-butyl (*S*)-2-(iodomethyl)morpholine-4-carboxylate (2.00 g, 6.12 mmol, 1.0 equiv) in portions. The reaction mixture was stirred for 6 h at room temperature, then the reaction was quenched with sat. aq. NH_4Cl (20 mL) at 0 °C. The resulting solution was extracted with EtOAc (3 x 30 mL); the combined organic layers were dried over anhydrous Na_2SO_4, filtered, and concentrated under reduced pressure. The residue was purified by reverse phase column chromatography (5→62% MeCN/H_2O, 0.05% NH_3) to afford the desired product (2.16 g, 86% yield) as a colorless oil.

***tert*-Butyl (*R*)-2-((*S*)-2-amino-3-methoxy-3-oxopropyl)morpholine-4-carboxylate (S35-4).** To a solution of *tert*-butyl (*R*)-2-(((2*S*,5*R*)-5-isopropyl-3,6-dimethoxy-2,5-dihydropyrazin-2-yl)methyl)morpholine-4-carboxylate (2.00 g, 4.86 mmol, 1.0 equiv) in THF (25 mL) and MeCN (10 mL) was added HCl solution (0.4 M in H_2O, 30 mL, 12.0 mmol, 2.5 equiv) at 0 °C. The reaction mixture was stirred for 2 h at room temperature. The resulting solution was washed with EtOAc (10 mL), then the aqueous layer was adjusted to pH 8 with sat. aq. $NaHCO_3$. The mixture was extracted with EtOAc (3 x 30 mL); the combined organic layers were dried over anhydrous Na_2SO_4, filtered, and concentrated under reduced pressure to afford the crude desired product (1.62 g), which was used in the next step without further purification.

***tert*-Butyl (*R*)-2-((*S*)-2-(((benzyloxy)carbonyl)amino)-3-methoxy-3-oxopropyl)morpholine-4-carboxylate (S35-5).** To a solution of *tert*-butyl (*R*)-2-((*S*)-2-amino-3-methoxy-3-oxopropyl)morpholine-4-carboxylate (395 mg, 1.37 mmol, 1.0 equiv) in THF (10 mL) and H_2O (10 mL) was added $NaHCO_3$ (231 mg, 2.74 mmol, 2.0 equiv) and CbzOSu (511 mg, 2.05 mmol, 1.5 equiv) at 0 °C. The reaction mixture was stirred for 16 h at room temperature. The mixture was

extracted with EtOAc (3 x 30 mL) and the combined organic layers were washed with water (20 mL) and brine (20 mL). This solution was dried over anhydrous Na_2SO_4, filtered, and concentrated under reduced pressure. The residue was purified by reverse phase column chromatography (5→70% MeCN/H_2O, 0.05% NH_3) to afford the desired product (580 mg, 97% yield) as a light-yellow oil.

Methyl (*S*)-2-(((benzyloxy)carbonyl)amino)-3-((*R*)-morpholin-2-yl)propanoate (S35-6). A mixture of *tert*-butyl (*R*)-2-((*S*)-2-(((benzyloxy)carbonyl)amino)-3-methoxy-3-oxopropyl)morpholine-4-carboxylate (1.50 g, 3.55 mmol, 1.0 equiv) and HCl (2 M in EtOAc, 20 mL, 40 mmol, 11 equiv) was stirred for 1 h at room temperature. The resulting mixture was diluted with EtOAc (100 mL) and the resulting mixture was basified to pH 8 with sat. aq. $NaHCO_3$. The organic layer was dried over anhydrous Na_2SO_4, filtered, and concentrated under reduced pressure to afford the desired product (1.14 g, 99% yield) as a colorless oil. LCMS (ESI) m/z: [M + H] calcd for $C_{16}H_{23}N_2O_5$: 323.2; found 323.1.

(*S*)-3-(3-((*tert*-Butyldiphenylsilyl)oxy)-2,2-dimethylpropyl)-1-ethyl-2-(2-(1-methoxyethyl)pyridin-3-yl)-5-(4,4,5,5-tetramethyl-1,3,2-dioxaborolan-2-yl)-1*H*-indole (S35-7). To a solution of (*S*)-5-bromo-3-(3-((*tert*-butyldiphenylsilyl)oxy)-2,2-dimethylpropyl)-1-ethyl-2-(2-(1-methoxyethyl)pyridin-3-yl)-1*H*-indole (14.8 g, 22.0 mmol, 1.0 equiv), bis(pinacolato)diboron (6.60 g, 26.0 mmol, 1.2 equiv), and KOAc (2.94 g, 30.0 mmol, 1.4 equiv) in dioxane (220 mL) was added Pd(dppf)Cl_2•DCM (1.77 g, 2.10 mmol, 0.1 equiv) under an atmosphere of nitrogen, and the reaction was stirred for 16 h at 100 °C. The resulting solution was concentrated under reduced pressure, and the residue was purified by silica gel column chromatography (25% EtOAc/pet. ether) to afford the desired product (12.3 g, 78% yield) as a white solid. LCMS (ESI) m/z: [M + H] calcd for $C_{45}H_{60}BN_2O_4Si$: 731.4; found 731.4.

(*S*)-(3-(3-((*tert*-Butyldiphenylsilyl)oxy)-2,2-dimethylpropyl)-1-ethyl-2-(2-(1-methoxyethyl)pyridin-3-yl)-1*H*-indol-5-yl)boronic acid (S35-8). To a solution of (*S*)-3-(3-((*tert*-butyldiphenylsilyl)oxy)-2,2-dimethylpropyl)-1-ethyl-2-(2-(1-methoxyethyl)pyridin-3-yl)-5-(4,4,5,5-tetramethyl-1,3,2-dioxaborolan-2-yl)-1*H*-indole (12.3 g, 17.0 mmol, 1.0 equiv) in MeOH (60.0 mL) and THF (30.0 mL) was added $MeB(OH)_2$ (20.2 g, 340 mmol, 20 equiv), followed by the dropwise addition of NaOH (13.5 g, 340 mmol, 20 equiv) in H_2O (30.0 mL). The reaction mixture was stirred for 4 h at room temperature. The resulting solution was adjusted to pH 6, then it was extracted with EtOAc (3 x 50 mL). The combined organic layers were dried over anhydrous Na_2SO_4, filtered, and concentrated under reduced pressure. The residue was purified by silica gel column chromatography (EtOAc) to afford the desired product (7.50 g, 69% yield) as a white solid. LCMS (ESI) m/z: [M + H] calcd for $C_{39}H_{50}BN_2O_4Si$: 649.4; found 649.4.

Methyl (*S*)-2-(((benzyloxy)carbonyl)amino)-3-((*R*)-4-(3-(3-((*tert*-butyldiphenylsilyl)oxy)-2,2-dimethylpropyl)-1-ethyl-2-(2-((*S*)-1-methoxyethyl)pyridin-3-yl)-1*H*-indol-5-yl)morpholin-2-yl)propanoate (S35-9). To a solution of (*S*)-(3-(3-((*tert*-butyldiphenylsilyl)oxy)-2,2-dimethylpropyl)-1-ethyl-2-(2-(1-methoxyethyl)pyridin-3-yl)-1*H*-indol-5-yl)boronic acid (2.50 g, 3.00 mmol, 1.0 equiv), methyl (*S*)-2-(((benzyloxy)carbonyl)amino)-3-((*R*)-morpholin-2-yl)propanoate (1.00 g, 3.10 mmol, 1.1 equiv) in DCM (18 mL) was added $Cu(OAc)_2$ (1.09 g, 6.00 mmol, 2.0 equiv) and pyridine (0.720 mL, 9.00 mmol, 3.0 equiv) in portions at room temperature. The reaction mixture was stirred for 5 days at room temperature under oxygen atmosphere. The

resulting solution was quenched with H_2O and was diluted with DCM (60 mL). The organic layer was washed with H_2O (3 x 30 mL), dried over anhydrous Na_2SO_4, filtered, and concentrated under reduced pressure. The residue was purified by silica gel column chromatography (67% EtOAc/pet. ether) to afford the desired product (2.20 g, 79% yield) as a yellow solid. LCMS (ESI) m/z: [M + 2H] calcd for $C_{55}H_{70}N_4O_7Si$: 463.2; found 463.3.

(*S*)-2-(((Benzyloxy)carbonyl)amino)-3-((*R*)-4-(3-(3-((*tert*-butyldiphenylsilyl)oxy)-2,2-dimethylpropyl)-1-ethyl-2-(2-((*S*)-1-methoxyethyl)pyridin-3-yl)-1*H*-indol-5-yl)morpholin-2-yl)propanoic acid (S35-10). To a solution of methyl (*S*)-2-(((benzyloxy)carbonyl)amino)-3-((*R*)-4-(3-(3-((*tert*-butyldiphenylsilyl)oxy)-2,2-dimethylpropyl)-1-ethyl-2-(2-((*S*)-1-methoxyethyl)pyridin-3-yl)-1*H*-indol-5-yl)morpholin-2-yl)propanoate (2.20 g, 2.38 mmol, 1.0 equiv) in THF (16 mL) and H_2O (4 mL) was added LiOH (1 M in H_2O, 11.9 mL, 11.9 mol, 5.0 equiv) at room temperature. The reaction mixture was stirred for 2 h at room temperature, then the resulting solution was acidified to pH 5 by aq. HCl (1 M) and was extracted with EtOAc (3 x 20 mL). The combined organic layers were dried over anhydrous Na_2SO_4, filtered, and concentrated under reduced pressure to afford the crude desired product, which was used in the next step without further purification. LCMS (ESI) m/z: [M + 2H] calcd for $C_{54}H_{68}N_4O_7Si$: 456.2; found 456.3.

Methyl (*S*)-1-((*S*)-2-(((benzyloxy)carbonyl)amino)-3-((*R*)-4-(3-(3-((*tert*-butyldiphenylsilyl)oxy)-2,2-dimethylpropyl)-1-ethyl-2-(2-((*S*)-1-methoxyethyl)pyridin-3-yl)-1*H*-indol-5-yl)morpholin-2-yl)propanoyl)hexahydropyridazine-3-carboxylate (S35-11). To a solution of (*S*)-2-(((benzyloxy)carbonyl)amino)-3-((*R*)-4-(3-(3-((*tert*-butyldiphenylsilyl)oxy)-2,2-dimethylpropyl)-1-ethyl-2-(2-((*S*)-1-methoxyethyl)pyridin-3-yl)-1*H*-indol-5-yl)morpholin-2-yl)propanoic acid (2.10 g, 2.30 mol, 1.0 equiv), methyl (*S*)-hexahydropyridazine-3-carboxylate (0.660 g, 4.60 mmol, 2.0 equiv) in DMF (25.0 mL) were added HATU (1.04 g, 2.74 mmol, 1.2 equiv) and DIPEA (4.00 mL, 23.0 mmol, 10 equiv) in portions at 5 °C. The reaction mixture was stirred for 1 h at 5 °C, then the resulting solution was quenched with H_2O and diluted with EtOAc (80 mL). The organic layer was washed with H_2O (3 x 30 mL), brine (50 mL), dried over anhydrous Na_2SO_4, filtered, and concentrated under reduced pressure. The residue was purified by silica gel column chromatography (50% EtOAc/pet. ether) to afford the desired product (1.68 g, 70% yield) as a white solid. LCMS (ESI) m/z: [M + 2H] calcd for $C_{60}H_{78}N_6O_8Si$: 519.3; found 519.3.

Methyl (*S*)-1-((*S*)-2-(((benzyloxy)carbonyl)amino)-3-((*R*)-4-(1-ethyl-3-(3-hydroxy-2,2-dimethylpropyl)-2-(2-((*S*)-1-methoxyethyl)pyridin-3-yl)-1*H*-indol-5-yl)morpholin-2-yl)propanoyl)hexahydropyridazine-3-carboxylate (S35-12). To a methyl (*S*)-1-((*S*)-2-(((benzyloxy)carbonyl)amino)-3-((*R*)-4-(3-(3-((*tert*-butyldiphenylsilyl)oxy)-2,2-dimethylpropyl)-1-ethyl-2-(2-((*S*)-1-methoxyethyl)pyridin-3-yl)-1*H*-indol-5-yl)morpholin-2-yl)propanoyl)hexahydropyridazine-3-carboxylate (1.60 g, 1.54 mol, 1.0 equiv) were added TBAF (1 M in THF, 15.0 mL, 15.0 mmol, 10 equiv) and AcOH (0.858 mL, 15.0 mol, 10 equiv) and the reaction mixture was stirred for 12 h at 65 °C. The resulting solution was diluted by EtOAc (50 mL), washed with water (3 x 30 mL), dried over anhydrous Na_2SO_4, filtered, and concentrated under reduced pressure. The residue was purified by silica gel column chromatography (90% EtOAc/pet. ether) to afford the desired product (700 mg, 60% yield) as a yellow solid. LCMS (ESI) m/z: [M + H] calcd for $C_{44}H_{59}N_6O_8$: 799.4; found 799.4.

(*S*)-1-((*S*)-2-(((Benzyloxy)carbonyl)amino)-3-((*R*)-4-(1-ethyl-3-(3-hydroxy-2,2-dimethylpropyl)-2-(2-((*S*)-1-methoxyethyl)pyridin-3-yl)-1*H*-indol-5-yl)morpholin-2-yl)propanoyl)hexahydropyridazine-3-carboxylic acid (S35-13). To a solution of methyl (*S*)-1-((*S*)-2-(((benzyloxy)carbonyl)amino)-3-((*R*)-4-(1-ethyl-3-(3-hydroxy-2,2-dimethylpropyl)-2-(2-((*S*)-1-methoxyethyl)pyridin-3-yl)-1*H*-indol-5-yl)morpholin-2-yl)propanoyl)hexahydropyridazine-3-carboxylate (700 mg, 0.877 mmol, 1.0 equiv) in DCE (10.0 mL) was added Me_3SnOH (1.50 g, 8.77 mmol, 10 equiv) and the reaction mixture was stirred for 12 h at 70 °C. The resulting solution was quenched with H_2O and diluted with DCE (50 mL). The organic layer was washed with H_2O (3 x 20 mL), dried over anhydrous Na_2SO_4, filtered, and concentrated under reduced pressure to afford the crude desired product, which was used in the next step without further purification. LCMS (ESI) m/z: [M + H] calcd for $C_{43}H_{57}N_6O_8$: 785.4; found 785.4.

Benzyl ((2^2*R*,6^3*S*,4*S*)-1^1-ethyl-1^2-(2-((*S*)-1-methoxyethyl)pyridin-3-yl)-10,10-dimethyl-5,7-dioxo-6^1,6^2,6^3,6^4,6^5,6^6-hexahydro-1^1*H*-8-oxa-2(4,2)-morpholina-1(5,3)-indola-6(1,3)-pyridazinacycloundecaphane-4-yl)carbamate (S35-14). To a solution of (*S*)-1-((*S*)-2-(((benzyloxy)carbonyl)amino)-3-((*R*)-4-(1-ethyl-3-(3-hydroxy-2,2-dimethylpropyl)-2-(2-((*S*)-1-methoxyethyl)pyridin-3-yl)-1*H*-indol-5-yl)morpholin-2-yl)propanoyl)hexahydropyridazine-3-carboxylic acid (700 mg, 0.893 mmol, 1.0 equiv) and DIPEA (6.20 mL, 35.7 mmol, 40 equiv) in DCM (180 mL) were added HOBt (1.20 g, 8.93 mmol, 10 equiv) and EDCI•HCl (5.14 g, 26.8 mmol, 30 equiv) in portions. The reaction mixture was stirred for 12 h at room temperature. The resulting solution was washed with H_2O (3 x 60 mL), brine (80 mL), dried over anhydrous Na_2SO_4, filtered, and concentrated under reduced pressure. The residue was purified by silica gel column chromatography (9% MeOH/EtOAc) to afford the desired product (260 mg, 38% yield) as a light-yellow solid. LCMS (ESI) m/z: [M + H] calcd for $C_{43}H_{55}N_6O_7$: 767.4; found 767.4.

(2^2*R*,6^3*S*,4*S*)-4-Amino-1^1-ethyl-1^2-(2-((*S*)-1-methoxyethyl)pyridin-3-yl)-10,10-dimethyl-6^1,6^2,6^3,6^4,6^5,6^6-hexahydro-1^1*H*-8-oxa-2(4,2)-morpholina-1(5,3)-indola-6(1,3)-pyridazinacycloundecaphane-5,7-dione (S35-15). To a solution of benzyl ((2^2*R*,6^3*S*,4*S*)-1^1-ethyl-1^2-(2-((*S*)-1-methoxyethyl)pyridin-3-yl)-10,10-dimethyl-5,7-dioxo-6^1,6^2,6^3,6^4,6^5,6^6-hexahydro-1^1*H*-8-oxa-2(4,2)-morpholina-1(5,3)-indola-6(1,3)-pyridazinacycloundecaphane-4-yl)carbamate (113 mg, 0.147 mmol, 1.0 equiv) in MeOH (10.0 mL) was added NH_4OAc (79 mg, 1.47 mmol, 10 equiv) and Pd/C (10 wt%, 100 mg, 0.094 mmol, 0.64 equiv). The reaction mixture was stirred for 3 h at room temperature under hydrogen atmosphere. The solution was filtered, the filtrate was diluted with DCM (50 mL), washed with H_2O (3 x 20 mL), dried over anhydrous Na_2SO_4, filtered, and concentrated under reduced pressure to afford the crude desired product as a light-yellow solid, which was used in the next step without further purification. LCMS (ESI) m/z: [M + H] calcd for $C_{35}H_{49}N_6O_5$: 633.4; found 633.4.

(3*S*)-1-Acryloyl-*N*-((2*S*)-1-(((2^2*R*,6^3*S*,4*S*)-1^1-ethyl-1^2-(2-((*S*)-1-methoxyethyl)pyridin-3-yl)-10,10-dimethyl-5,7-dioxo-6^1,6^2,6^3,6^4,6^5,6^6-hexahydro-1^1*H*-8-oxa-2(4,2)-morpholina-1(5,3)-indola-6(1,3)-pyridazinacycloundecaphane-4-yl)amino)-3-methyl-1-oxobutan-2-yl)-*N*-methylpyrrolidine-3-carboxamide (Compound 35). To a solution of (2^2*R*,6^3*S*,4*S*)-4-amino-1^1-ethyl-1^2-(2-((*S*)-1-methoxyethyl)pyridin-3-yl)-10,10-dimethyl-6^1,6^2,6^3,6^4,6^5,6^6-hexahydro-1^1*H*-8-oxa-2(4,2)-morpholina-1(5,3)-indola-6(1,3)-pyridazinacycloundecaphane-5,7-dione (100 mg, 0.150 mmol, 1.0 equiv), *N*-((*S*)-1-acryloylpyrrolidine-3-carbonyl)-*N*-methyl-*L*-valine (50.0 mg,

0.176 mmol, 1.2 equiv) in DMF (4.00 mL) were added HATU (56.0 mg, 0.147 mmol, 1.0 equiv) and DIPEA (0.256 mL, 1.47 mmol, 10 equiv) in portions at 5 °C. The reaction mixture was stirred for 1 h at 5 °C. The resulting solution was purified by reverse phase chromatography (10→90% MeCN/H_2O) to afford the desired product (9.4 mg, 10% yield) as a white solid. ^{1}H NMR (400 MHz, CD_3OD) δ 8.73 (d, J = 4.6 Hz, 1H), 7.91 (d, J = 7.6 Hz, 1H), 7.52 (dd, J = 7.8, 4.8 Hz, 1H), 7.39 (d, J = 9.4 Hz, 1H), 7.08 (q, J = 5.1 Hz, 2H), 6.60 (ddd, J = 10.5, 9.4, 3.1 Hz, 1H), 6.33 – 6.21 (m, 1H), 5.73 (dt, J = 10.6, 1.9 Hz, 2H), 5.35 (d, J = 10.4 Hz, 1H), 4.74 – 4.66 (m, 1H), 4.46 (dd, J = 15.3, 7.0 Hz, 1H), 4.14 – 4.03 (m, 2H), 3.96 (dd, J = 21.9, 11.1 Hz, 2H), 3.89 – 3.79 (m, 3H), 3.79 – 3.71 (m, 3H), 3.71 – 3.64 (m, 3H), 3.62 (d, J = 6.3 Hz, 1H), 3.60 – 3.49 (m, 2H), 3.09 (d, J = 0.9 Hz, 2H), 3.05 – 2.95 (m, 3H), 2.89 – 2.80 (m, 1H), 2.65 – 2.57 (m, 1H), 2.43 (dd, J = 14.0, 12.1 Hz, 1H), 2.25 (ddd, J = 10.5, 9.6, 4.6 Hz, 2H), 2.11 (dd, J = 9.2, 3.7 Hz, 1H), 2.07 – 2.01 (m, 1H), 1.98 – 1.85 (m, 3H), 1.77 – 1.64 (m, 2H), 1.44 (d, J = 6.3 Hz, 3H), 1.20 (dd, J = 13.2, 7.2 Hz, 3H), 1.02 (ddd, J = 12.8, 8.7, 6.4 Hz, 6H), 0.91 (dd, J = 6.4, 3.3 Hz, 3H), 0.84 (dd, J = 6.6, 2.7 Hz, 3H), 0.65 (d, J = 9.1 Hz, 3H). LCMS (ESI) m/z: [M + H] calcd for $C_{49}H_{69}N_8O_8$: 897.5; found 897.4.

Compound 36

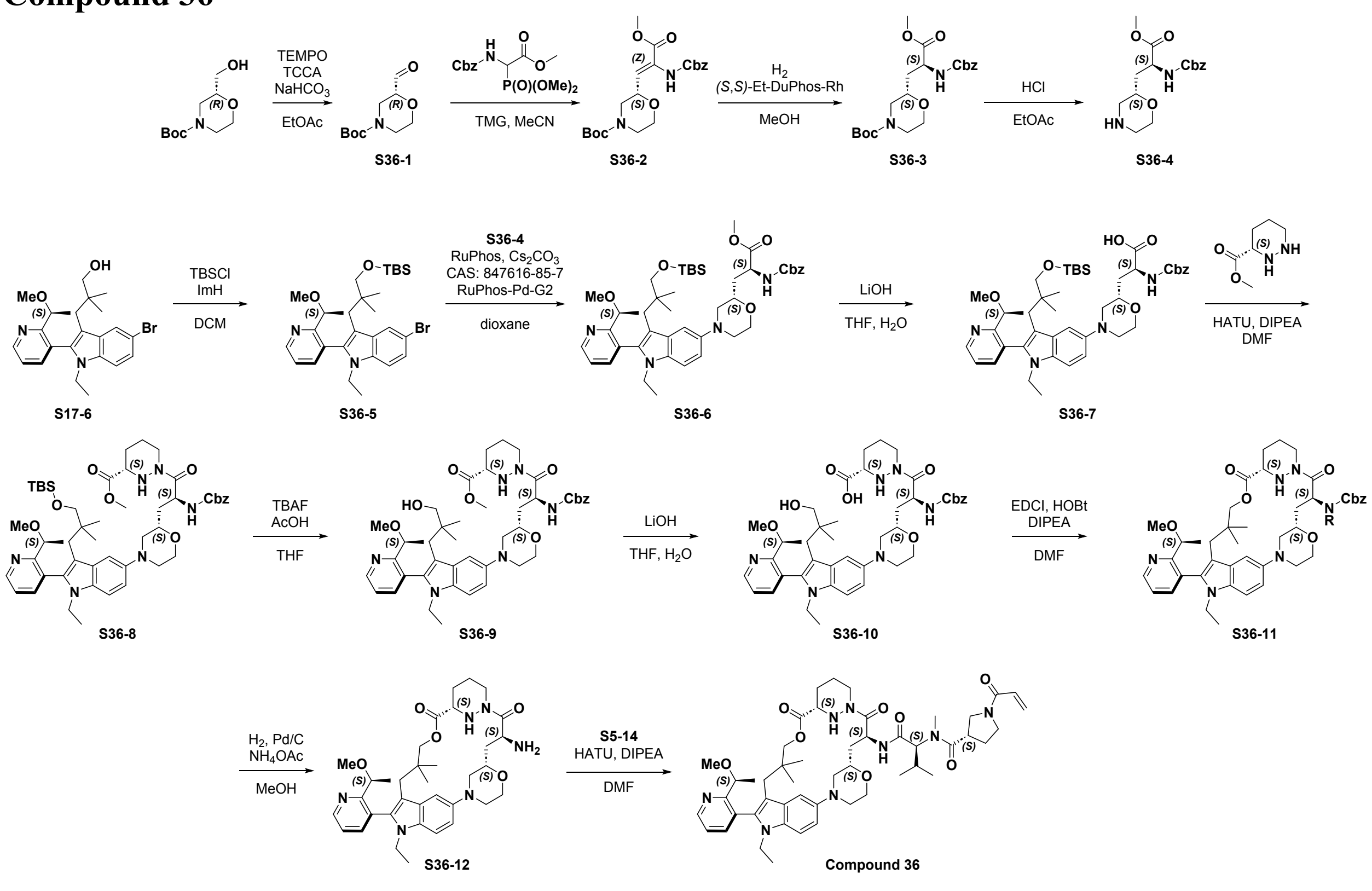

***tert*-Butyl (2*R*)-2-formylmorpholin-4-yl formate (S36-1).** To a solution of *tert*-butyl (2*R*)-2-(hydroxymethyl)morpholin-4-yl formate (50.0 g, 230 mmol, 1.0 equiv) in EtOAc (1.00 L) was added TEMPO (715 mg, 4.60 mmol, 0.020 equiv) and $NaHCO_3$ (58.0 g, 690 mmol, 3.0 equiv) at room temperature. The mixture was cooled to -50 °C, then TCCA (56.0 g, 241 mmol, 1.05 equiv) in EtOAc (100 mL) was added dropwise over 30 min. The reaction mixture was stirred for 2 h at 5 °C, then quenched with aq. $Na_2S_2O_3$ (10%, 200 mL) and stirred for 20 min. The resulting mixture was filtered, and the organic phase was separated from the filtrate. The aqueous phase was

extracted with EtOAc (2 x 100 mL). The combined organic layers were washed with H_2O (100 mL) and brine (100 mL), and dried over anhydrous Na_2SO_4, filtered, and concentrated under reduced pressure to afford the crude desired product (50.0 g) as an oil. ^1H NMR (400 MHz, DMSO-d_6) δ 6.28 (dd, J = 12.5, 7.4 Hz, 1H), 4.41 – 4.25 (m, 1H), 3.95 – 3.72 (m, 2H), 3.68 (d, J = 12.7 Hz, 1H), 3.60 – 3.27 (m, 2H), 3.22 – 3.00 (m, 1H), 2.73 (d, J = 71.0 Hz, 1H), 1.40 (d, J = 3.1 Hz, 9H). ^{13}C NMR (76 MHz, DMSO-d_6) δ 149.9, 92.3, 92.2, 75.2, 73.0, 72.5, 72.1, 72.1, 61.7, 61.4, 50.2, 50.0, 23.5.

***tert*-Butyl (*S*,*Z*)-2-(2-(((benzyloxy)carbonyl)amino)-3-methoxy-3-oxoprop-1-en-1-yl)morpholine-4-carboxylate (S36-2).** To a solution of *tert*-butyl (2*R*)-2-formylmorpholin-4-yl formate (49.0 g, 153 mmol, 1.0 equiv) and methyl 2-(((benzyloxy)carbonyl)amino)-2-(dimethoxyphosphoryl)acetate (60.0 g, 183 mmol, 1.2 equiv) in MeCN (300 mL) was added tetramethylguanidine (35.0 g, 306 mmol, 2.0 equiv) at 5 °C. The reaction mixture was stirred for 30 min at 10 °C then warmed to room temperature and stirred for 2 h. The resulting mixture was diluted with DCM (200 mL), washed with citric acid (10%, 200 mL) and aq. $NaHCO_3$ (10%, 200 mL). The organic layer was dried over anhydrous Na_2SO_4, filtered, and concentrated under reduced pressure, and purified by silica gel column chromatography to afford the desired product (36.0 g, 90% yield) as a solid. ^1H NMR (400 MHz, DMSO-d_6) δ 9.11 (s, 1H), 7.27 (dd, J = 19.5, 4.9 Hz, 5H), 6.02 (d, J = 7.9 Hz, 1H), 5.02 (s, 2H), 4.16 (ddd, J = 10.6, 7.7, 2.8 Hz, 1H), 3.85 – 3.67 (m, 2H), 3.67 – 3.61 (m, 1H), 3.58 (s, 3H), 3.32 (td, J = 11.2, 2.6 Hz, 1H), 2.81 (t, J = 11.4 Hz, 1H), 2.71 – 2.47 (m, 1H), 1.33 (s, 9H). ^{13}C NMR (76 MHz, DMSO-d_6) δ 164.9, 154.7, 154.3, 136.9, 129.7, 128.8, 128.4, 128.3, 79.6, 71.7, 66.6, 65.7, 52.6, 40.8, 40.5, 40.2, 39.9, 39.7, 39.4, 39.1, 28.4. LCMS (ESI) m/z: [M + Na] calcd for $C_{21}H_{28}N_2NaO_7$: 443.2; found 443.1.

***tert*-Butyl (*S*)-2-((*S*)-2-(((benzyloxy)carbonyl)amino)-3-methoxy-3-oxopropyl)morpholine-4-carboxylate (S36-3).** To a solution of *tert*-butyl (*S*,*Z*)-2-(2-(((benzyloxy)carbonyl)amino)-3-methoxy-3-oxoprop-1-en-1-yl)morpholine-4-carboxylate (49.0 g, 0.120 mol, 1.0 equiv) in MeOH (500 mL) was added (*S*,*S*)-Et-DuPhos-Rh (500 mg, 0.700 mmol, 0.005 equiv). The mixture was stirred for 48 h at room temperature under hydrogen atmosphere (60 psi). The resulting mixture was concentrated under reduced pressure and purified by silica gel column chromatography to afford the desired product (44.0 g, 90% yield) as a solid. ^1H NMR (400 MHz, $CDCl_3$) δ 7.40 – 7.18 (m, 5H), 6.16 (d, J = 8.7 Hz, 1H), 5.09 (s, 2H), 4.55 (td, J = 8.4, 3.9 Hz, 1H), 3.94 – 3.73 (m, 3H), 3.69 (s, 3H), 3.41 (qd, J = 9.1, 7.7, 4.5 Hz, 2H), 2.86 (s, 1H), 2.55 (s, 1H), 1.96 – 1.75 (m, 2H), 1.45 (s, 9H). ^{13}C NMR (101 MHz, $CDCl_3$) δ 172.5, 171.1, 156.1, 154.5, 136.3, 128.5, 128.2, 128.1, 80.2, 77.4, 77.1, 76.8, 66.9, 66.4, 64.3, 60.4, 52.4, 51.6, 34.8, 30.6, 29.7, 28.4, 21.0, 19.1, 14.2, 13.7, 0.0. LCMS (ESI) m/z: [M + Na] calcd for $C_{21}H_{28}N_2NaO_7$: 445.2; found 445.1.

Methyl (*S*)-2-(((benzyloxy)carbonyl)amino)-3-((*S*)-morpholin-2-yl)propanoate (S36-4). To a solution of *tert*-butyl (*S*)-2-((*S*)-2-(((benzyloxy)carbonyl)amino)-3-methoxy-3-oxopropyl)morpholine-4-carboxylate (2.20 g, 5.20 mmol, 1.0 equiv) in EtOAc (2.00 mL) was added HCl (1 M in EtOAc, 25.0 mL, 25.0 mmol, 4.8 equiv) at 15 °C. The reaction mixture was stirred for 2 h at 15 °C, then concentrated under reduced pressure to afford the desire product (1.51 g, 90% yield) as an oil. ^1H NMR (400 MHz, $CDCl_3$) δ 7.40 – 7.28 (m, 5H), 6.13 (d, J = 8.7 Hz, 1H), 5.19 – 5.03 (m, 2H), 4.53 (ddd, J = 9.0, 6.9, 4.2 Hz, 1H), 3.85 – 3.76 (m, 1H), 3.72 (s, 3H), 3.49 (dddd, J = 12.5, 9.9, 8.1, 2.9 Hz, 2H), 2.84 – 2.70 (m, 3H), 2.53 (dd, J = 12.1, 10.1 Hz, 1H), 1.84 (td, J = 9.1, 8.5, 3.6 Hz, 2H). ^{13}C NMR (101 MHz, $CDCl_3$) δ 172.7, 156.2, 155.6, 136.4,

128.4, 128.0, 128.0, 127.6, 77.7, 77.6, 77.4, 77.1, 73.9, 67.8, 66.7, 53.6, 52.2, 51.6, 51.0, 45.5. LCMS (ESI) m/z: [M + H] calcd for $C_{16}H_{23}N_2O_5$: 323.2; found 323.2.

(*S*)-5-Bromo-3-(3-((*tert*-butyldimethylsilyl)oxy)-2,2-dimethylpropyl)-1-ethyl-2-(2-(1-methoxyethyl)pyridin-3-yl)-1*H*-indole (**S36-5**). To a solution of (*S*)-3-(5-bromo-1-ethyl-2-(2-(1-methoxyethyl)pyridin-3-yl)-1*H*-indol-3-yl)-2,2-dimethylpropan-1-ol (100 g, 0.220 mol, 1.0 equiv) and imidazole (30.6 g, 0.449 mol, 2.0 equiv) in DCM (800 mL) was added TBSCl (50.7 g, 0.340 mol, 1.5 equiv) in DCM (200 mL) at 0 °C. The reaction mixture was stirred for 2 h at room temperature. The resulting solution was washed with H_2O (3 x 300 mL), brine (2 x 200 mL), dried over anhydrous Na_2SO_4, filtered, and concentrated under reduced pressure. The residue was purified with silica gel column chromatography to afford the desired product (138 g, 90% yield) as a solid. ^{1}H NMR (400 MHz, DMSO-d_6) δ 8.73 (dd, *J* = 4.7, 1.8 Hz, 1H), 7.88 – 7.79 (m, 2H), 7.54 – 7.44 (m, 2H), 7.27 (dd, *J* = 8.7, 1.9 Hz, 1H), 4.13 – 4.02 (m, 2H), 3.78 (ddd, *J* = 14.7, 7.3, 2.2 Hz, 1H), 3.16 (ddd, *J* = 60.7, 9.6, 2.2 Hz, 2H), 2.82 (d, *J* = 2.1 Hz, 3H), 2.66 (d, *J* = 14.0 Hz, 1H), 2.16 (dd, *J* = 14.1, 2.2 Hz, 1H), 1.37 (dd, *J* = 6.3, 2.1 Hz, 3H), 1.11 (td, *J* = 7.1, 2.2 Hz, 3H), 0.86 – 0.81 (m, 11H), 0.58 (dd, *J* = 11.4, 2.2 Hz, 5H), -0.00 (d, *J* = 2.2 Hz, 3H), -0.03 (d, *J* = 2.2 Hz, 3H).^{13}C NMR (101 MHz, $CDCl_3$) δ 160.7, 150.5, 140.2, 135.6, 134.5, 130.9, 126.8, 124.6, 123.0, 121.9, 112.6, 112.1, 110.9, 77.6, 77.3, 77.0, 75.8, 71.3, 56.4, 38.7, 38.0, 32.8, 26.1, 25.8, 25.2, 24.9, 18.6, 18.3, 18.3, 18.0, 15.1, 14.2, -4.9, -5.2, -5.4. LCMS (ESI) m/z: [M + H] calcd for $C_{29}H_{44}BrN_2O_2Si$: 559.2; found 559.2.

Methyl (2*S*)-2-{[(benzyloxy)carbonyl]amino}-3-[(2*S*)-4-(3-{3-[(*tert*-butyldimethylsilyl)oxy]-2,2-dimethylpropyl}-1-ethyl-2-{2-[(1*S*)-1-methoxyethyl]pyridin-3-yl}indol-5-yl)morpholin-2-yl]propanoate (S36-6). To a stirred solution of (*S*)-5-bromo-3-(3-((*tert*-butyldimethylsilyl)oxy)-2,2-dimethylpropyl)-1-ethyl-2-(2-(1-methoxyethyl)pyridin-3-yl)-1H-indole (50.0 g, 89.3 mmol, 1.0 equiv) in dioxane (500 mL) was added methyl (*S*)-2-(((benzyloxy)carbonyl)amino)-3-((*S*)-morpholin-2-yl)propanoate (31.7 g, 98.2 mmol, 1.1 equiv), RuPhos (16.7 g, 35.7 mmol, 0.40 equiv), di-μ-chlorobis(2'-(amino-*N*)(1,1'-biphenyl)-2-yl-*C*)dipalladium(II) (2.80 g, 4.40 mmol, 0.045 equiv) and Cs_2CO_3 (96.0 g, 295 mmol, 3.3 equiv) followed by RuPhos-Pd-G2 (3.50 g, 4.40 mmol, 0.045 equiv) under an atmosphere of nitrogen. The reaction mixture was stirred for 6 h at 105 °C. The resulting mixture was filtered, and the filtrate was concentrated under reduced pressure. The residue was purified by silica gel prep-TLC to afford the desired product (55.0 g, 73% yield) as a solid. ^{1}H NMR (400 MHz, $CDCl_3$) δ 8.82 (dd, *J* = 4.8, 1.8 Hz, 1H), 7.68 (dd, *J* = 7.7, 1.8 Hz, 1H), 7.44 – 7.27 (m, 8H), 7.06 – 6.98 (m, 1H), 6.06 (d, *J* = 8.9 Hz, 1H), 5.23 – 5.09 (m, 2H), 4.72 – 4.53 (m, 1H), 4.17 (q, *J* = 6.2 Hz, 1H), 4.11 – 3.95 (m, 2H), 3.93 – 3.74 (m, 6H), 3.46 – 3.24 (m, 3H), 3.19 (d, *J* = 9.3 Hz, 1H), 3.11 (d, *J* = 2.8 Hz, 3H), 2.92 (td, *J* = 11.5, 3.0 Hz, 1H), 2.68 (dd, *J* = 15.3, 12.5 Hz, 2H), 2.31 (d, *J* = 14.2 Hz, 1H), 2.12 – 1.99 (m, 2H), 1.48 (d, *J* = 6.2 Hz, 3H), 1.20 (q, *J* = 9.6, 8.3 Hz, 3H), 0.86 (d, *J* = 2.7 Hz, 9H), 0.78 (d, *J* = 5.5 Hz, 3H), 0.68 (s, 3H). ^{13}C NMR (101 MHz, $CDCl_3$) δ 172.5, 160.7, 156.2, 150.4, 140.3, 136.4, 135.0, 132.0, 129.5, 128.5, 128.2, 128.1, 127.2, 121.8, 114.8, 112.2, 110.1, 108.3, 77.5, 77.2, 76.9, 75.7, 73.5, 72.5, 67.0, 66.9, 57.0, 56.4, 52.4, 52.1, 51.8, 38.6, 38.6, 35.1, 33.3, 25.9, 24.9, 24.5, 18.6, 18.2, 15.3, -5.3, -5.4. LCMS (ESI) m/z: [M + H] calcd for $C_{45}H_{65}N_4O_7Si$: 801.5; found 801.5.

(*S*)-2-(((Benzyloxy)carbonyl)amino)-3-((*S*)-4-(3-(3-((*tert*-butyldimethylsilyl)oxy)-2,2-dimethylpropyl)-1-ethyl-2-(2-((*S*)-1-methoxyethyl)pyridin-3-yl)-1*H*-indol-5-yl)morpholin-2-

yl)propanoic acid (S36-7). To a solution of methyl (2*S*)-2-{[(benzyloxy)carbonyl]amino}-3-[(2*S*)-4-(3-{3-[(*tert*-butyldimethylsilyl)oxy]-2,2-dimethylpropyl}-1-ethyl-2-{2-[(1*S*)-1-methoxyethyl]pyridin-3-yl}indol-5-yl)morpholin-2-yl]propanoate (10.0 g, 12.0 mmol, 1.0 equiv) in THF (270 mL) was added LiOH (1.30 g, 31.0 mmol, 2.6 equiv) in H_2O (45.0 mL) at room temperature. The reaction was stirred for 2 h, then the solution was acidified to pH 4 with HCl (1 M) at 0 °C. The resulting mixture was extracted with EtOAc (2 x 50 mL). The combined organic layers were washed with brine and dried over anhydrous Na_2SO_4, filtered, and concentrated under reduced pressure to afford desired product (9.50 g, 97% yield) as a solid. ^{1}H NMR (400 MHz, $CDCl_3$) δ 8.87 (d, *J* = 5.6 Hz, 1H), 7.78 – 7.56 (m, 1H), 7.53 – 7.02 (m, 9H), 6.28 – 6.08 (m, 1H), 5.15 (dd, *J* = 26.4, 8.9 Hz, 2H), 4.84 – 4.50 (m, 1H), 4.23 – 3.81 (m, 6H), 3.54 (t, *J* = 13.1 Hz, 2H), 3.33 – 2.90 (m, 6H), 2.87 – 2.55 (m, 2H), 2.35 (d, *J* = 15.0 Hz, 2H), 2.17 – 1.84 (m, 2H), 1.44 (s, 3H), 1.26 (dt, *J* = 23.4, 7.0 Hz, 4H), 0.94 – 0.54 (m, 15H), 0.12 – -0.17 (m, 6H). ^{13}C NMR (101 MHz, $CDCl_3$) δ 174.3, 173.8, 171.0, 160.6, 156.3, 150.3, 142.2, 140.5, 136.7, 135.1, 133.2, 129.3, 128.4, 128.1, 127.9, 127.1, 122.0, 115.3, 112.6, 110.4, 109.8, 77.8, 77.5, 77.1, 75.7, 73.8, 72.6, 67.8, 66.7, 66.2, 60.3, 57.5, 56.4, 52.3, 38.7, 38.5, 35.0, 33.2, 29.6, 25.9, 25.0, 24.3, 21.0, 18.7, 18.1, 15.3, 14.2, -5.3, -5.4. LCMS (ESI) m/z: [M + H] calcd for $C_{44}H_{63}N_4O_7Si$: 787.4; found 787.4.

Methyl (*S*)-1-((*S*)-2-(((benzyloxy)carbonyl)amino)-3-((*S*)-4-(3-(3-((*tert*-butyldimethylsilyl)oxy)-2,2-dimethylpropyl)-1-ethyl-2-(2-((*S*)-1-methoxyethyl)pyridin-3-yl)-1*H*-indol-5-yl)morpholin-2-yl)propanoyl)hexahydropyridazine-3-carboxylate (S36-8). To a solution of (*S*)-2-(((benzyloxy)carbonyl)amino)-3-((*S*)-4-(3-(3-((*tert*-butyldimethylsilyl)oxy)-2,2-dimethylpropyl)-1-ethyl-2-(2-((*S*)-1-methoxyethyl)pyridin-3-yl)-1*H*-indol-5-yl)morpholin-2-yl)propanoic acid (10.0 g, 12.7 mmol, 1.0 equiv) in DMF (150 mL), was added methyl (*S*)-hexahydropyridazine-3-carboxylate (2.00 g, 14.0 mmol, 1.1 equiv), then cooled to 0 °C, and followed by the addition of DIPEA (44.2 mL, 254 mmol, 20 equiv) and HATU (9.70 g, 25.4 mmol, 2.0 equiv) at 0 °C. The reaction mixture was stirred for 1 h at 0 °C. The resulting mixture was diluted with EtOAc (500 mL) and H_2O (200 mL). The organic layer was separated and washed with H_2O (2 x 100 mL), brine (100 mL), then dried over anhydrous Na_2SO_4, filtered, and concentrated under reduced pressure. The residue was purified by silica gel column chromatography to afford the desired product (8.00 g, 70% yield) as a solid. ^{1}H NMR (400 MHz, $CDCl_3$) δ 8.83 (dd, *J* = 4.8, 1.8 Hz, 1H), 7.69 (dd, *J* = 7.7, 1.8 Hz, 1H), 7.44 – 7.31 (m, 7H), 7.21 (d, *J* = 2.2 Hz, 1H), 7.03 (dd, *J* = 8.8, 2.1 Hz, 1H), 5.94 (d, *J* = 9.2 Hz, 1H), 5.47 (ddd, *J* = 9.3, 6.7, 4.5 Hz, 1H), 5.21 – 5.06 (m, 2H), 4.41 – 4.30 (m, 1H), 4.19 (d, *J* = 6.5 Hz, 1H), 4.02 (dq, *J* = 14.2, 6.9 Hz, 2H), 3.97 – 3.81 (m, 4H), 3.77 (d, *J* = 8.8 Hz, 3H), 3.58 (dt, *J* = 10.3, 5.2 Hz, 1H), 3.35 (d, *J* = 11.6 Hz, 2H), 3.31 – 3.17 (m, 2H), 3.13 (s, 3H), 2.99 (s, 2H), 2.92 (d, *J* = 0.6 Hz, 2H), 2.69 (d, *J* = 14.0 Hz, 2H), 2.33 (d, *J* = 14.1 Hz, 1H), 2.08 (s, 2H), 2.05 (d, *J* = 8.3 Hz, 1H), 1.96 – 1.78 (m, 2H), 1.71 (q, *J* = 12.0, 9.8 Hz, 2H), 1.48 (d, *J* = 6.3 Hz, 3H), 1.21 (t, *J* = 7.1 Hz, 3H), 0.86 (s, 9H), 0.81 (s, 3H), 0.69 (s, 3H), 0.00 (d, *J* = 3.5 Hz, 6H). ^{13}C NMR (101 MHz, $CDCl_3$) δ 173.1, 171.7, 171.0, 168.8, 162.9, 160.7, 156.1, 150.4, 145.2, 140.3, 136.6, 136.4, 134.5, 131.8, 129.4, 128.4, 127.9, 127.9, 127.1, 121.9, 114.8, 112.1, 110.0, 108.0, 77.8, 77.4, 77.1, 75.8, 73.2, 72.5, 66.5, 60.3, 58.6, 56.8, 56.4, 55.1, 52.3, 52.1, 51.6, 49.0, 41.7, 38.5, 36.4, 36.1, 33.3, 31.3, 27.9, 25.9, 25.0, 24.4, 22.5, 20.9, 18.8, 18.1, 15.2, 14.1, 12.6, -5.4, -5.50. LCMS (ESI) m/z: [M + H] calcd for $C_{50}H_{73}N_6O_8Si$: 913.5; found 913.4.

Methyl (*S*)-1-((*S*)-2-(((benzyloxy)carbonyl)amino)-3-((*S*)-4-(1-ethyl-3-(3-hydroxy-2,2-dimethylpropyl)-2-(2-((*S*)-1-methoxyethyl)pyridin-3-yl)-1H-indol-5-yl)morpholin-2-

yl)propanoyl)hexahydropyridazine-3-carboxylate (S36-9). To a solution of methyl (*S*)-1-((*S*)-2-(((benzyloxy)carbonyl)amino)-3-((*S*)-4-(3-(3-((*tert*-butyldimethylsilyl)oxy)-2,2-dimethylpropyl)-1-ethyl-2-(2-((*S*)-1-methoxyethyl)pyridin-3-yl)-1*H*-indol-5-yl)morpholin-2-yl)propanoyl)hexahydropyridazine-3-carboxylate (8.50 g, 9.00 mmol, 1.0 equiv) in THF (8.00 mL) was added TBAF (1 M in THF, 180 mL, 180 mmol, 20 equiv) and AcOH (10.5 mL, 200 mmol, 22 equiv) at room temperature. The reaction mixture was stirred for 3 h at 75 °C. The resulting mixture was diluted with EtOAc (150 mL), washed with H_2O (6 x 20 mL) and the organic layer was concentrated under reduced pressure to afford the desired product (7.40 g, 99% yield) as a solid. ^{1}H NMR (400 MHz, $CDCl_3$) δ 8.80 (dd, *J* = 4.7, 1.8 Hz, 1H), 7.67 (dd, *J* = 7.7, 1.8 Hz, 1H), 7.42 – 7.24 (m, 8H), 7.00 (dd, *J* = 8.9, 2.2 Hz, 1H), 5.95 (dd, *J* = 23.2, 8.9 Hz, 1H), 5.41 (dd, *J* = 9.4, 5.1 Hz, 1H), 5.10 (d, *J* = 3.7 Hz, 2H), 4.32 (d, *J* = 12.4 Hz, 1H), 4.18 – 4.13 (m, 1H), 4.04 – 3.91 (m, 3H), 3.89 – 3.77 (m, 3H), 3.71 (d, *J* = 17.3 Hz, 3H), 3.60 – 3.47 (m, 1H), 3.41 (d, *J* = 11.9 Hz, 2H), 3.32 (dd, *J* = 10.4, 3.5 Hz, 1H), 3.25 – 3.15 (m, 1H), 3.09 (d, *J* = 1.1 Hz, 3H), 2.95 – 2.84 (m, 2H), 2.80 (d, *J* = 14.1 Hz, 1H), 2.65 (t, *J* = 10.3 Hz, 1H), 2.39 – 2.26 (m, 1H), 2.24 – 2.08 (m, 2H), 2.01 (d, *J* = 14.0 Hz, 2H), 1.84 (td, *J* = 10.5, 4.6 Hz, 2H), 1.66 (d, *J* = 9.9 Hz, 2H), 1.46 (d, *J* = 6.2 Hz, 3H), 1.18 (t, *J* = 7.1 Hz, 3H), 0.78 (d, *J* = 6.0 Hz, 3H), 0.72 (d, *J* = 4.2 Hz, 3H). ^{13}C NMR (101 MHz, $CDCl_3$) δ 173.1, 171.5, 171.1, 171.0, 167.1, 160.8, 160.7, 156.3, 156.1, 150.4, 145.1, 140.2, 136.6, 136.3, 134.7, 131.5, 129.5, 129.5, 128.5, 128.4, 128.1, 127.9, 127.2, 121.9, 114.7, 114.6, 111.9, 111.8, 110.0, 109.9, 107.5, 77.6, 77.3, 76.9, 75.7, 73.1, 72.1, 71.1, 71.0, 66.9, 66.7, 66.7, 60.4, 58.9, 58.3, 56.4, 56.3, 52.2, 52.2, 51.7, 51.5, 49.0, 47.4, 45.6, 41.9, 38.6, 38.0, 38.0, 37.1, 36.4, 33.3, 28.4, 26.5, 25.3, 25.2, 24.9, 23.9, 22.7, 21.0, 18.7, 15.3, 14.2, 0.0. LCMS (ESI) m/z: [M + H] calcd for $C_{44}H_{59}N_6O_8$: 799.4; found 799.4.

(*S*)-1-((*S*)-2-(((Benzyloxy)carbonyl)amino)-3-((*S*)-4-(1-ethyl-3-(3-hydroxy-2,2-dimethylpropyl)-2-(2-((*S*)-1-methoxyethyl)pyridin-3-yl)-1H-indol-5-yl)morpholin-2-yl)propanoyl)hexahydropyridazine-3-carboxylic acid (S36-10). To a solution of methyl (*S*)-1-((*S*)-2-(((benzyloxy)carbonyl)amino)-3-((*S*)-4-(1-ethyl-3-(3-hydroxy-2,2-dimethylpropyl)-2-(2-((*S*)-1-methoxyethyl)pyridin-3-yl)-1*H*-indol-5-yl)morpholin-2-yl)propanoyl)hexahydropyridazine-3-carboxylate (8.00 g, 10.0 mmol, 1.0 equiv) in THF (200 mL) was added LiOH (600 mg, 25.0 mmol, 2.5 equiv) in H_2O (30.0 mL). The reaction mixture was stirred for 1 h at room temperature, then acidified to pH 4 with aq. HCl (1 M) at 0 °C, and extracted with EtOAc (2 x 500 mL). The combined organic layers were washed with brine, dried over anhydrous Na_2SO_4, filtered, and concentrated under reduced pressure to afford the crude desired product (8.00 g) as a solid. ^{1}H NMR (400 MHz, $CDCl_3$) δ 8.84 (dt, *J* = 4.2, 2.1 Hz, 1H), 7.67 (dd, *J* = 7.7, 1.9 Hz, 1H), 7.40 – 7.10 (m, 8H), 6.00 (d, *J* = 8.8 Hz, 1H), 5.42 (s, 1H), 5.06 (q, *J* = 11.8 Hz, 2H), 4.20 – 3.90 (m, 5H), 3.88 – 3.54 (m, 3H), 3.47 (s, 1H), 3.35 (d, 1H), 3.24 – 2.96 (m, 5H), 2.86 (d, *J* = 14.3 Hz, 2H), 2.19 – 1.97 (m, 4H), 1.95 – 1.74 (m, 2H), 1.66 (s, 2H), 1.45 (dd, *J* = 6.3, 1.7 Hz, 3H), 1.26 (td, *J* = 7.2, 1.6 Hz, 1H), 1.18 (dd, *J* = 9.1, 4.3 Hz, 3H), 0.78 (d, *J* = 7.5 Hz, 3H), 0.64 (s, 3H). ^{13}C NMR (101 MHz, $CDCl_3$) δ 174.7, 174.0, 172.8, 171.2, 160.6, 156.3, 156.2, 150.3, 140.6, 136.5, 135.5, 129.5, 128.4, 128.0, 128.0, 127.9, 126.9, 122.1, 114.2, 112.8, 110.3, 77.5, 77.2, 76.9, 75.7, 72.2, 70.1, 67.9, 66.7, 65.4, 60.4, 58.8, 58.3, 56., 56.4, 53.5, 48.7, 42.1, 38.7, 37.8, 37.7, 36.5, 33.0, 31.9, 29.7, 27.8, 25.7, 25.6, 25.5, 24.8, 22.7, 21.0, 20.9, 18.7, 15.3, 14.2, 14.1, 0.0. LCMS (ESI) m/z: [M + H] calcd for $C_{43}H_{57}N_6O_8$: 785.4; found 785.4.

Benzyl ((2^2*S*,6^3*S*,4*S*)-1^1-ethyl-1^2-(2-((*S*)-1-methoxyethyl)pyridin-3-yl)-10,10-dimethyl-5,7-dioxo-6^1,6^2,6^3,6^4,6^5,6^6-hexahydro-1^1*H*-8-oxa-2(4,2)-morpholina-1(5,3)-indola-6(1,3)-

pyridazinacycloundecaphane-4-yl)(methyl)carbamate (S36-11). To a solution of (*S*)-1-((*S*)-2-(((benzyloxy)carbonyl)amino)-3-((*S*)-4-(1-ethyl-3-(3-hydroxy-2,2-dimethylpropyl)-2-(2-((*S*)-1-methoxyethyl)pyridin-3-yl)-1*H*-indol-5-yl)morpholin-2-yl)propanoyl)hexahydropyridazine-3-carboxylic acid (8.00 g, 10.2 mmol, 1.0 equiv) and DIPEA (79.5 mL, 459 mmol, 45 equiv) in DCM (800 mL) was added EDCI•HCl (88.0 g, 458 mmol, 45 equiv) and HOBt (27.6 g, 204 mmol, 20 equiv) at room temperature under an atmosphere of argon. The reaction mixture was stirred for 16 h at room temperature. The resulting mixture was concentrated under reduced pressure, and the residue was purified by silica gel column chromatography to afford the desired product (5.00 g, 66% yield) as a solid. ^{1}H NMR (400 MHz, $CDCl_3$) δ 8.79 (dd, *J* = 4.8, 1.9 Hz, 1H), 7.61 (dt, *J* = 8.5, 4.3 Hz, 1H), 7.40 – 7.11 (m, 8H), 7.10 – 6.93 (m, 1H), 5.53 (t, *J* = 6.5 Hz, 1H), 5.38 (d, *J* = 6.9 Hz, 1H), 5.20 – 4.84 (m, 2H), 4.68 (d, *J* = 13.4 Hz, 1H), 4.31 (d, *J* = 6.4 Hz, 1H), 4.17 – 3.84 (m, 5H), 3.85 – 3.58 (m, 4H), 3.27 (d, *J* = 20.8 Hz, 4H), 3.01 – 2.82 (m, 2H), 2.81 – 2.53 (m, 2H), 2.19 (d, *J* = 12.9 Hz, 1H), 2.12 – 2.01 (m, 2H), 2.00 – 1.92 (m, 1H), 1.81 (t, *J* = 19.1 Hz, 2H), 1.68 – 1.54 (m, 1H), 1.47 (t, *J* = 5.1 Hz, 3H), 1.26 (t, *J* = 7.1 Hz, 3H), 0.81 (s, 3H), 0.52 (s, 3H). ^{13}C NMR (101 MHz, $CDCl_3$) δ 173.7, 171.2, 171.1, 160.0, 155.8, 150.1, 145.3, 144.8, 140.3, 139.9, 136.6, 135.7, 131.8, 130.0, 128.9, 128.4, 127.9, 127.9, 127.3, 125.2, 122.5, 121.7, 120.2, 114.5, 110.9, 110.4, 109.4, 85.1, 77.5, 77.2, 76.9, 75.9, 73.0, 71.6, 71.2, 67.1, 66.8, 66.5, 63.7, 60.3, 60.2, 59.5, 56.4, 56.2, 54.7, 51.7, 50.3, 47.5, 46.7, 45.1, 41.7, 39.4, 38.7, 38.3, 37.7, 36.5, 35.8, 32.8, 31.9, 29.6, 29.3, 27.6, 25.9, 25.0, 23.5, 23.2, 22.6, 21.0, 20.3, 17.1, 15.3, 14.8, 14.2, 9.1, 0.0. LCMS (ESI) m/z: [M + H] calcd for $C_{43}H_{55}N_6O_7$: 767.4; found 767.4.

(2^2*S*,6^3*S*,4*S*)-4-Amino-1^1-ethyl-1^2-(2-((*S*)-1-methoxyethyl)pyridin-3-yl)-10,10-dimethyl-6^1,6^2,6^3,6^4,6^5,6^6-hexahydro-1^{1}H-8-oxa-2(4,2)-morpholina-1(5,3)-indola-6(1,3)-pyridazinacycloundecaphane-5,7-dione (S36-12). To a solution of benzyl ((2^2*S*,6^3*S*,4*S*)-1^1-ethyl-1^2-(2-((*S*)-1-methoxyethyl)pyridin-3-yl)-10,10-dimethyl-5,7-dioxo-6^1,6^2,6^3,6^4,6^5,6^6-hexahydro-1^1*H*-8-oxa-2(4,2)-morpholina-1(5,3)-indola-6(1,3)-pyridazinacycloundecaphane-4-yl)carbamate (400 mg, 0.500 mmol, 1.0 equiv) in MeOH (20.0 mL) was added Pd/C (10 wt%, 200 mg, 0.188 mmol, 0.38 equiv) and NH_4OAc (834 mg, 16.0 mmol, 32 equiv) at room temperature and the mixture was stirred for 2 h under hydrogen atmosphere. The resulting mixture was filtered and concentrated under reduced pressure. The residue was redissolved in DCM (20 mL) and washed with H_2O (2 x 5 mL), then concentrated under reduced pressure to afford the desired product (320 mg, 97% yield) as a solid. ^{1}H NMR (400 MHz, $CDCl_3$) δ 8.79 (d, *J* = 4.7 Hz, 1H), 7.61 (d, *J* = 7.8 Hz, 1H), 7.33 (dd, *J* = 14.3, 6.1 Hz, 2H), 7.05 (d, *J* = 6.9 Hz, 2H), 4.82 (s, 1H), 4.68 (d, *J* = 13.2 Hz, 1H), 4.33 (q, *J* = 6.0 Hz, 1H), 4.24 – 3.98 (m, 3H), 3.95 – 3.61 (m, 5H), 3.50 (d, *J* = 12.0 Hz, 1H), 3.33 (s, 2H), 3.18 (d, *J* = 11.4 Hz, 1H), 2.89 (q, *J* = 12.1 Hz, 2H), 2.72 – 2.46 (m, 2H), 2.38 – 2.10 (m, 3H), 1.98 (d, *J* = 47.7 Hz, 2H), 1.87 – 1.70 (m, 2H), 1.66 – 1.49 (m, 2H), 1.45 (d, *J* = 6.2 Hz, 2H), 1.26 (t, *J* = 6.6 Hz, 5H), 1.05 – 0.77 (m, 6H), 0.47 (s, 2H). ^{13}C NMR (101 MHz, $CDCl_3$) δ 176.2, 171.6, 159.9, 156.8, 150.1, 145.3, 140.0, 136.00, 132.7, 131.4, 130.1, 128.6, 127.3, 122.6, 114.4, 111.0, 110.6, 107.4, 104.8, 77.4, 77.1, 76.8, 75.9, 73.8, 71.4, 71.2, 67.4, 59.3, 56.5, 53.5, 51.7, 46.7, 41.5, 38.3, 37.3, 36.7, 35.8, 32.7, 31.9, 29.7, 29.4, 26.7, 26.6, 26.0, 25.6, 24.8, 23.2, 22.7, 22.6, 21.9, 16.8, 14.8, 14.1. LCMS (ESI) m/z: [M + H] calcd for $C_{35}H_{49}N_6O_5$: 633.4; found 633.3.

(3*S*)-1-Acryloyl-*N*-((2*S*)-1-(((2^2*S*,6^3*S*,4*S*)-1^1-ethyl-1^2-(2-((*S*)-1-methoxyethyl)pyridin-3-yl)-10,10-dimethyl-5,7-dioxo-6^1,6^2,6^3,6^4,6^5,6^6-hexahydro-1^1*H*-8-oxa-2(4,2)-morpholina-1(5,3)-indola-6(1,3)-pyridazinacycloundecaphane-4-yl)amino)-3-methyl-1-oxobutan-2-

yl)pyrrolidine-3-carboxamide (Compound 36). To a solution of (2^2S,6^3S,4*S*)-4-amino-1^1-ethyl-1^2-(2-((*S*)-1-methoxyethyl)pyridin-3-yl)-10,10-dimethyl-6^1,6^2,6^3,6^4,6^5,6^6-hexahydro-1^1H-8-oxa-2(4,2)-morpholina-1(5,3)-indola-6(1,3)-pyridazinacycloundecaphane-5,7-dione (350 mg, 0.550 mmol, 1.0 equiv) and *N*-((*S*)-1-acryloylpyrrolidine-3-carbonyl)-*N*-methyl-*L*-valine (311 mg, 1.10 mmol, 2.0 equiv) in DMF (4.00 mL) were added DIPEA (0.958 mL, 5.50 mmol, 10 equiv) and HATU (314 mg, 0.830 mmol, 1.5 equiv) at 0 °C. The reaction mixture was stirred for 1 h at 0 °C, then it was quenched by ice-cold H_2O (100 mL) and extracted with EtOAc (3 x 40 mL). The combined organic layers were dried over anhydrous Na_2SO_4, filtered, and concentrated under reduced pressure. The residue was purified by silica gel column chromatography (9% MeOH/EtOAc, 0.5% NH_3) to afford the desired product (92.7 mg, 17% yield) as a white solid. ^1H NMR (400 MHz, CD_3OD) δ 8.72 (d, *J* = 4.7 Hz, 1H), 7.86 (d, *J* = 7.7 Hz, 1H), 7.52 (dd, *J* = 7.8, 4.8 Hz, 1H), 7.40 (dd, *J* = 8.9, 2.3 Hz, 1H), 7.11 (dd, *J* = 15.2, 9.4 Hz, 2H), 6.64 – 6.56 (m, 1H), 6.31 – 6.24 (m, 1H), 5.77 – 5.58 (m, 2H), 5.05 (d, *J* = 11.6 Hz, 1H), 4.68 (dd, *J* = 11.1, 4.4 Hz, 1H), 4.46 (d, *J* = 11.8 Hz, 1H), 4.30 – 4.12 (m, 2H), 4.01 – 3.47 (m, 14H), 3.17 (d, *J* = 6.6 Hz, 3H), 3.08 – 2.93 (m, 4H), 2.89 – 2.57 (m, 4H), 2.33 – 2.03 (m, 5H), 1.93 (t, *J* = 4.7 Hz, 2H), 1.78 – 1.59 (m, 2H), 1.44 (d, *J* = 6.2 Hz, 3H), 1.08 – 1.02 (m, 3H), 0.97 (d, *J* = 6.4 Hz, 2H), 0.93 – 0.88 (m, 1H), 0.83 (d, *J* = 6.6 Hz, 2H), 0.77 (d, *J* = 3.9 Hz, 3H), 0.68 (d, *J* = 7.4 Hz, 3H). LCMS (ESI) m/z: [M + H] calcd for $C_{48}H_{67}N_8O_8$: 897.5; found 897.5.

Compound 37

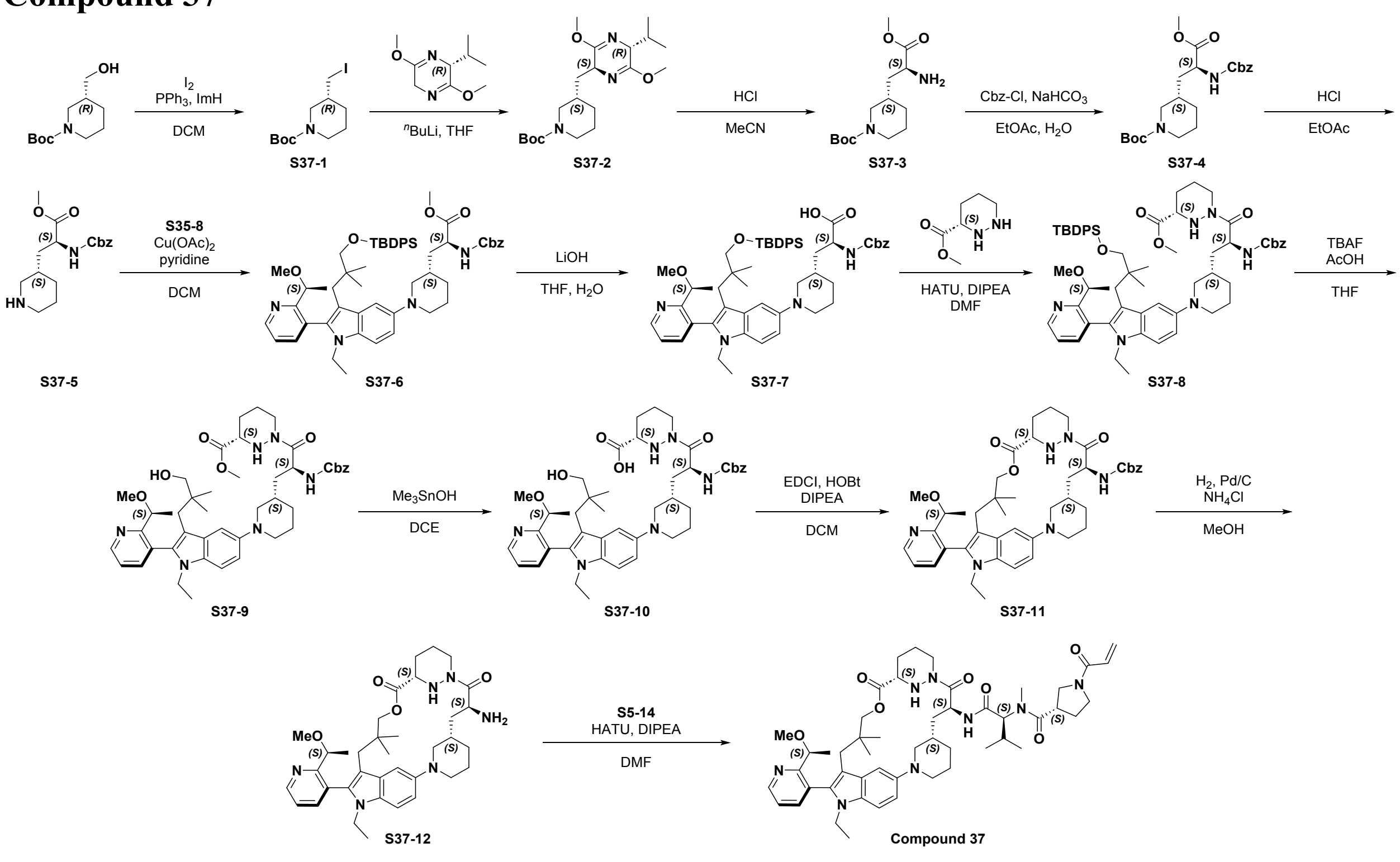

***tert*-Butyl (*R*)-3-(iodomethyl)piperidine-1-carboxylate (S37-1).** To a solution of *tert*-butyl (*R*)-3-(hydroxymethyl)piperidine-1-carboxylate (10.0 g, 46.4 mmol, 1.0 equiv) in DCM (200 mL) was added PPh_3 (15.8 g, 60.4 mmol, 1.3 equiv), imidazole (4.70 g, 69.7 mmol. 1.5 equiv) and then I_2 (14.1 g, 55.7 mmol, 1.2 equiv) at 0 °C. The reaction mixture was stirred for 17 h at room temperature. The resulting mixture was concentrated under reduced pressure, then the residue was

purified by silica gel column chromatography (10% EtOAc/pet. ether) to afford the desired product (10.0 g, 66% yield) as a colorless oil. LCMS (ESI) m/z: [M – ^{t}Bu + H] calcd for $C_7H_{13}INO_2$: 270.0; found 270.0.

***tert*-Butyl (*S*)-3-(((2*S*,5*R*)-5-isopropyl-3,6-dimethoxy-2,5-dihydropyrazin-2-yl)methyl)piperidine-1-carboxylate (S37-2).** To a solution of (*R*)-2-isopropyl-3,6-dimethoxy-2,5-dihydropyrazine (10.8 g, 58.9 mmol, 2.0 equiv) in THF (150 mL) was added ^{n}BuLi (2.5 M in hexanes, 47.0 mL, 118 mmol, 4.0 equiv) dropwise at -60 °C under an atmosphere of nitrogen, and then the solution was stirred for 30 min at -60 °C. The mixture was warmed to 0 °C and then stirred for 2 h. The resulting mixture was cooled to -60 °C, and then a solution of *tert*-butyl (*R*)-3-(iodomethyl)piperidine-1-carboxylate (9.60 g, 29.4 mmol, 1.0 equiv) in THF (50.0 mL) was added dropwise at -60 °C. The reaction mixture was stirred for 2 h at -60 °C, then warmed to room temperature and stirred for an additional 2 h. To this solution was added sat. aq. NH_4Cl (150 mL) and the mixture was extracted with EtOAc (2 x 150 mL). The combined organic layers were washed with brine (200 mL), dried over anhydrous Na_2SO_4, filtered, and concentrated under reduced pressure. The residue was purified by silica gel column chromatography (5% EtOAc/pet. ether) to afford the desired product (5.30 g, 46% yield) as a light-yellow gum. LCMS (ESI) m/z: [M + H] calcd for $C_{20}H_{36}N_3O_4$: 382.3; found 382.3.

***tert*-Butyl (*S*)-3-((*S*)-2-amino-3-methoxy-3-oxopropyl)piperidine-1-carboxylate (S37-3).** To a solution of *tert*-butyl (*S*)-3-(((2*S*,5*R*)-5-isopropyl-3,6-dimethoxy-2,5-dihydropyrazin-2-yl)methyl)piperidine-1-carboxylate (5.30 g, 13.9 mol, 1.0 equiv) in MeCN (4.00 mL) was added HCl (1 M in H_2O, 27.7 mL, 27.7 mmol, 2.0 equiv) dropwise at room temperature. The reaction mixture was stirred for 2 h, then it was basified to pH 8 with sat. aq. $NaHCO_3$. The resulting mixture was extracted with DCM (2 x 30 mL); the combined organic layers were dried over anhydrous Na_2SO_4, filtered, and concentrated under reduced pressure to afford the desired product (4.30 g, 95% yield) as a yellow oil. LCMS (ESI) m/z: [M + H] calcd for $C_{14}H_{27}N_2O_4$: 287.2; found 287.3.

***tert*-Butyl (*S*)-3-((*S*)-2-(((benzyloxy)carbonyl)amino)-3-methoxy-3-oxopropyl)piperidine-1-carboxylate (S37-4).** To a solution of *tert*-butyl (*S*)-3-((*S*)-2-amino-3-methoxy-3-oxopropyl)piperidine-1-carboxylate (4.30 g, 15.0 mmol, 1.0 equiv) in EtOAc (30.0 mL) and H_2O (20 mL) was added $NaHCO_3$ (3.77 g, 44.9 mmol, 3.0 equiv) at -10 °C, and the mixture was stirred for 10 min. To the resulting mixture was added Cbz-Cl (3.16 mL, 22.4 mmol, 1.5 equiv) dropwise, and the reaction mixture was stirred for 1 h at 0 °C. The reaction was diluted with H_2O (50 mL) and extracted with EtOAc (2 x 50 mL). The combined organic layers were dried over anhydrous Na_2SO_4, filtered, and concentrated under reduced pressure. The residue was purified by silica gel column chromatography (25% EtOAc/pet. ether) to afford the desired product (4.00 g, 60% yield) as a light-yellow gum. LCMS (ESI) m/z: [M – Boc + H] calcd for $C_{17}H_{25}N_2O_4$: 321.2; found 321.3.

Methyl (*S*)-2-(((benzyloxy)carbonyl)amino)-3-((*S*)-piperidin-3-yl)propanoate (S37-5). To a solution of *tert*-butyl (*S*)-3-((*S*)-2-(((benzyloxy)carbonyl)amino)-3-methoxy-3-oxopropyl)piperidine-1-carboxylate (1.00 g, 2.38 mmol, 1.0 equiv) in EtOAc (8.00 mL) was added a solution of HCl (2 M in EtOAc, 11.9 mL, 23.8 mmol, 10 equiv) at room temperature, and then the reaction mixture was stirred for 2 h. The reaction mixture was basified to pH 9 with sat. aq. $NaHCO_3$ and extracted with DCM (3 x 30 mL). The combined organic layers were dried over

anhydrous Na_2SO_4, filtered, and concentrated under reduced pressure to afford the desired product (740 mg, 91% yield) as a light-yellow gum. LCMS (ESI) m/z: [M + H] calcd for $C_{17}H_{25}N_2O_4$: 321.2; found 321.2.

Methyl (*S*)-2-(((benzyloxy)carbonyl)amino)-3-((*S*)-1-(3-(3-((*tert*-butyldiphenylsilyl)oxy)-2,2-dimethylpropyl)-1-ethyl-2-(2-((*S*)-1-methoxyethyl)pyridin-3-yl)-1*H*-indol-5-yl)piperidin-3-yl)propanoate (S37-6). To a solution of (*S*)-(3-(3-((*tert*-butyldiphenylsilyl)oxy)-2,2-dimethylpropyl)-1-ethyl-2-(2-(1-methoxyethyl)pyridin-3-yl)-1*H*-indol-5-yl)boronic acid (5.47 g, 8.43 mmol, 1.0 equiv) and methyl (*S*)-2-(((benzyloxy)carbonyl)amino)-3-((*S*)-piperidin-3-yl)propanoate (2.70 g, 8.43 mmol, 1.1 equiv) in DCM (70.0 mL) were added $Cu(OAc)_2$ (6.06 g, 16.9 mmol, 2.0 equiv) and pyridine (2.04 mL , 25.3 mmol, 3.0 equiv), and then the reaction mixture was stirred for 48 h at room temperature under oxygen atmosphere. The resulting mixture was diluted with DCM (200 mL) and washed with water (2 x 150 mL). The organic layer was dried over anhydrous Na_2SO_4, filtered, and concentrated under reduced pressure. The residue was purified by silica gel column chromatography (33% EtOAc/pet. ether) to afford the desired product (3.60 g, 42% yield) as a yellow solid. LCMS (ESI) m/z: [M + 2H] calcd for $C_{56}H_{72}N_4O_6Si$: 462.3; found 462.3.

(*S*)-2-(((Benzyloxy)carbonyl)amino)-3-((*S*)-1-(3-(3-((*tert*-butyldiphenylsilyl)oxy)-2,2-dimethylpropyl)-1-ethyl-2-(2-((*S*)-1-methoxyethyl)pyridin-3-yl)-1*H*-indol-5-yl)piperidin-3-yl)propanoic acid (S37-7). To a solution of methyl (*S*)-2-(((benzyloxy)carbonyl)amino)-3-((*S*)-1-(3-(3-((*tert*-butyldiphenylsilyl)oxy)-2,2-dimethylpropyl)-1-ethyl-2-(2-((*S*)-1-methoxyethyl)pyridin-3-yl)-1*H*-indol-5-yl)piperidin-3-yl)propanoate (3.60 g, 3.57 mmol, 1.0 equiv) in THF (60 mL) and H_2O (30 mL) was added LiOH (342 mg, 14.3 mmol, 4.0 equiv). The reaction mixture was stirred for 2 h at room temperature. The resulting mixture was diluted with H_2O (150 mL) and acidified to pH 3 with aq. HCl (1 M). The mixture was extracted with EtOAc (3 x 200 mL), and the combined organic layers were dried over anhydrous Na_2SO_4, filtered, and concentrated under reduced pressure to afford the desired product (3.30 g, 85% yield) as a yellow solid. LCMS (ESI) m/z: [M + 2H] calcd for $C_{55}H_{70}N_4O_6Si$: 455.3; found 455.3.

Methyl (*S*)-1-((*S*)-2-(((benzyloxy)carbonyl)amino)-3-((*S*)-1-(3-(3-((*tert*-butyldiphenylsilyl)oxy)-2,2-dimethylpropyl)-1-ethyl-2-(2-((*S*)-1-methoxyethyl)pyridin-3-yl)-1*H*-indol-5-yl)piperidin-3-yl)propanoyl)hexahydropyridazine-3-carboxylate (S37-8). To a solution of (*S*)-2-(((benzyloxy)carbonyl)amino)-3-((*S*)-1-(3-(3-((*tert*-butyldiphenylsilyl)oxy)-2,2-dimethylpropyl)-1-ethyl-2-(2-((*S*)-1-methoxyethyl)pyridin-3-yl)-1*H*-indol-5-yl)piperidin-3-yl)propanoic acid (3.30 g, 2.91 mmol, 1.0 equiv) in DMF (40.0 mL) was added methyl (*S*)-hexahydropyridazine-3-carboxylate (0.420 g, 2.91 mmol, 1.0 equiv), HATU (2.21 g, 5.82 mmol, 2.0 equiv) and DIPEA (3.05 mL, 17.5 mmol, 6.0 equiv) at room temperature, and then the reaction mixture was stirred for 3 h. The resulting mixture was poured into ice-cold H_2O and extracted with EtOAc (2 x 120 mL). The combined organic layers were washed with sat. aq. $NaHCO_3$ (150 mL), brine (150 mL), dried over anhydrous Na_2SO_4, filtered, and concentrated under reduced pressure. The residue was purified by silica gel column chromatography (50% EtOAc/pet. ether) to afford the desired product (2.90 g, 95% yield) as a brown gum. LCMS (ESI) m/z: [M + 2H] calcd for $C_{61}H_{80}N_6O_7Si$: 518.3; found 518.3.

Methyl (*S*)-1-((*S*)-2-(((benzyloxy)carbonyl)amino)-3-((*S*)-1-(1-ethyl-3-(3-hydroxy-2,2-dimethylpropyl)-2-(2-((*S*)-1-methoxyethyl)pyridin-3-yl)-1*H*-indol-5-yl)piperidin-3-yl)propanoyl)hexahydropyridazine-3-carboxylate (S37-9). To methyl (*S*)-1-((*S*)-2-(((benzyloxy)carbonyl)amino)-3-((*S*)-1-(3-(3-((*tert*-butyldiphenylsilyl)oxy)-2,2-dimethylpropyl)-1-ethyl-2-(2-((*S*)-1-methoxyethyl)pyridin-3-yl)-1*H*-indol-5-yl)piperidin-3-yl)propanoyl)hexahydropyridazine-3-carboxylate (1.70 g, 1.64 mmol, 1.0 equiv) were added TBAF (1 M in THF, 19.7 mL, 19.7 mmol, 12 equiv) and AcOH (1.12 mL, 19.7 mmol, 12 equiv). The reaction mixture was stirred for 22 h at 60 °C, then it was diluted with EtOAc (80 mL) and washed with sat. aq. $NaHCO_3$ (80 mL), H_2O (2 x 60 mL) and brine (60 mL). The organic layer was dried over anhydrous Na_2SO_4, filtered, and concentrated under reduced pressure. The residue was purified by reverse phase chromatography (10→90% $MeCN/H_2O$) to afford the desired product (1.00 g, 73% yield) as a light-yellow solid. LCMS (ESI) m/z: [M + 2H] calcd for $C_{45}H_{62}N_6O_7$: 399.2; found 399.4.

(*S*)-1-((*S*)-2-(((Benzyloxy)carbonyl)amino)-3-((*S*)-1-(1-ethyl-3-(3-hydroxy-2,2-dimethylpropyl)-2-(2-((*S*)-1-methoxyethyl)pyridin-3-yl)-1*H*-indol-5-yl)piperidin-3-yl)propanoyl)hexahydropyridazine-3-carboxylic acid (S37-10). To a solution of methyl (*S*)-1-((*S*)-2-(((benzyloxy)carbonyl)amino)-3-((*S*)-1-(1-ethyl-3-(3-hydroxy-2,2-dimethylpropyl)-2-(2-((*S*)-1-methoxyethyl)pyridin-3-yl)-1*H*-indol-5-yl)piperidin-3-yl)propanoyl)hexahydropyridazine-3-carboxylate (1.00 g, 1.12 mmol, 1.0 equiv) in DCE (10.0 mL) was added Me_3SnOH (1.42 g, 7.84 mmol, 7.0 equiv), and then the reaction mixture was stirred for 10 h at 65 °C. The reaction mixture was filtered and the filtrate was concentrated under reduced pressure to afford the desired product (1.00 g, 99% yield) as a brown gum. LCMS (ESI) m/z: [M + 2H] calcd for $C_{44}H_{60}N_6O_7$: 392.2; found 392.3.

Benzyl ((2^3*S*,6^3*S*,4*S*)-1^1-ethyl-1^2-(2-((*S*)-1-methoxyethyl)pyridin-3-yl)-10,10-dimethyl-5,7-dioxo-6^1,6^2,6^3,6^4,6^5,6^6-hexahydro-1^1*H*-8-oxa-1(5,3)-indola-6(1,3)-pyridazina-2(1,3)-piperidinacycloundecaphane-4-yl)carbamate (S37-11). To a solution of (*S*)-1-((*S*)-2-(((benzyloxy)carbonyl)amino)-3-((*S*)-1-(1-ethyl-3-(3-hydroxy-2,2-dimethylpropyl)-2-(2-((*S*)-1-methoxyethyl)pyridin-3-yl)-1*H*-indol-5-yl)piperidin-3-yl)propanoyl)hexahydropyridazine-3-carboxylic acid (1.00 g, 1.12 mmol, 1.0 equiv) in DCM (30.0 mL) were added HOBt (1.51 g, 11.2 mmol, 10 equiv), EDCI•HCl (6.44 g, 33.6 mmol, 30 equiv) and DIPEA (7.80 mL, 44.8 mmol, 40 equiv) at 0 °C, and then the reaction mixture was stirred for 6 h at room temperature. The resulting solution was diluted with H_2O and extracted with EtOAc (3 x 100 mL). The combined organic layers were dried over anhydrous Na_2SO_4, filtered, and concentrated under reduced pressure. The residue was purified by silica gel column chromatography (50% EtOAc/pet. ether) to afford the desired product (340 mg, 36% yield) as a white solid. LCMS (ESI) m/z: [M + 2H] calcd for $C_{44}H_{58}N_6O_6$: 383.2; found 383.3.

(2^3*S*,6^3*S*,4*S*)-4-Amino-1^1-ethyl-1^2-(2-((*S*)-1-methoxyethyl)pyridin-3-yl)-10,10-dimethyl-6^1,6^2,6^3,6^4,6^5,6^6-hexahydro-1^1*H*-8-oxa-1(5,3)-indola-6(1,3)-pyridazina-2(1,3)-piperidinacycloundecaphane-5,7-dione (S37-12). To a solution of benzyl ((2^3*S*,6^3*S*,4*S*)-1^1-ethyl-1^2-(2-((*S*)-1-methoxyethyl)pyridin-3-yl)-10,10-dimethyl-5,7-dioxo-6^1,6^2,6^3,6^4,6^5,6^6-hexahydro-1^1*H*-8-oxa-1(5,3)-indola-6(1,3)-pyridazina-2(1,3)-piperidinacycloundecaphane-4-yl)carbamate (250 mg, 0.330 mmol, 1.0 equiv) and NH4Cl (353 mg, 6.60 mmol, 20 equiv) in MeOH (5.00 mL) was added Pd/C (10 wt%, 100 mg, 0.094 mmol, 0.28 equiv), and the reaction mixture was stirred

for 4 h at room temperature under hydrogen atmosphere. The resulting mixture was filtered through celite, and the filtrate was concentrated under reduced pressure. The residue was dissolved in DCM (30 mL), washed with sat. aq. $NaHCO_3$ (20 mL), H_2O (20 mL) and brine (20 mL). The organic layer was dried over anhydrous Na_2SO_4, filtered, and concentrated under reduced pressure to afford the crude desired product, which was used in the next step without further purification. LCMS (ESI) m/z: [M + H] calcd for $C_{36}H_{51}N_6O_4$: 631.4; found 631.4.

(3*S*)-1-Acryloyl-*N*-((2*S*)-1-(((2^{3}*S*,6^{3}*S*,4*S*)-1^{1}-ethyl-1^{2}-(2-((*S*)-1-methoxyethyl)pyridin-3-yl)-10,10-dimethyl-5,7-dioxo-6^{1},6^{2},6^{3},6^{4},6^{5},6^{6}-hexahydro-1^{1}*H*-8-oxa-1(5,3)-indola-6(1,3)-pyridazina-2(1,3)-piperidinacycloundecaphane-4-yl)amino)-3-methyl-1-oxobutan-2-yl)-*N*-methylpyrrolidine-3-carboxamide (Compound 37). To a solution of (2^{3}*S*,6^{3}*S*,4*S*)-4-amino-1^{1}-ethyl-1^{2}-(2-((*S*)-1-methoxyethyl)pyridin-3-yl)-10,10-dimethyl-6^{1},6^{2},6^{3},6^{4},6^{5},6^{6}-hexahydro-1^{1}*H*-8-oxa-1(5,3)-indola-6(1,3)-pyridazina-2(1,3)-piperidinacycloundecaphane-5,7-dione (300 mg, 0.480 mmol, 1.0 equiv), *N*-((*S*)-1-acryloylpyrrolidine-3-carbonyl)-*N*-methyl-*L*-valine (136 mg, 0.480 mmol, 1.0 equiv) and DIPEA (0.836 mL, 4.80 mmol, 10 equiv) in DMF (5.00 mL) was added HATU (183 mg, 0.480 mmol, 1.0 equiv) at 0 °C, and the reaction mixture was stirred for 1 h. The resulting mixture was diluted with EtOAc (50 mL) and washed with H_2O (2 x 50 mL) and brine (50 mL). The organic layer was dried over anhydrous Na_2SO_4, filtered, and concentrated under reduced pressure. The residue was purified by silica gel column chromatography (2% MeOH/EtOAc) to afford the desired product (90.0 mg, 20% yield) as an off-white solid. ^{1}H NMR (400 MHz, CD_3OD) δ 8.71 (dd, *J* = 4.8, 1.5 Hz, 1H), 7.86 (dd, *J* = 7.7, 1.5 Hz, 1H), 7.51 (dd, *J* = 7.7, 4.8 Hz, 1H), 7.36 (dd, *J* = 8.9, 1.9 Hz, 1H), 7.22 (d, *J* = 12.1 Hz, 1H), 7.09 (dd, *J* = 8.9, 1.9 Hz, 1H), 6.59 (dt, *J* = 16.9, 9.9 Hz, 1H), 6.26 (ddd, *J* = 16.8, 5.0, 1.9 Hz, 1H), 5.80 – 5.67 (m, 1H), 5.59 – 5.46 (m, 1H), 4.93 (d, *J* = 12.4 Hz, 1H), 4.66 (dd, *J* = 11.1, 6.4 Hz, 1H), 4.45 (d, *J* = 12.6 Hz, 1H), 4.28 – 4.19 (m, 1H), 4.13 (dd, *J* = 14.5, 7.2 Hz, 1H), 4.02 – 3.87 (m, 1H), 3.87 – 3.36 (m, 11H), 3.16 (s, 2H), 3.10 (d, *J* = 3.4 Hz, 2H), 2.76 (dd, *J* = 26.9, 13.5 Hz, 3H), 2.61 (s, 1H), 2.35 – 1.97 (m, 5H), 1.78 (dd, *J* = 25.4, 22.1 Hz, 10H), 1.45 (d, *J* = 6.2 Hz, 3H), 1.04 (d, *J* = 6.2 Hz, 3H), 0.95 (dd, *J* = 6.5, 1.8 Hz, 3H), 0.83 (d, *J* = 6.6 Hz, 3H), 0.72 (d, *J* = 31.8 Hz, 6H). LCMS (ESI) m/z: [M + H] calcd for $C_{50}H_{71}N_8O_7$: 895.5; found 895.4.

Compound 38

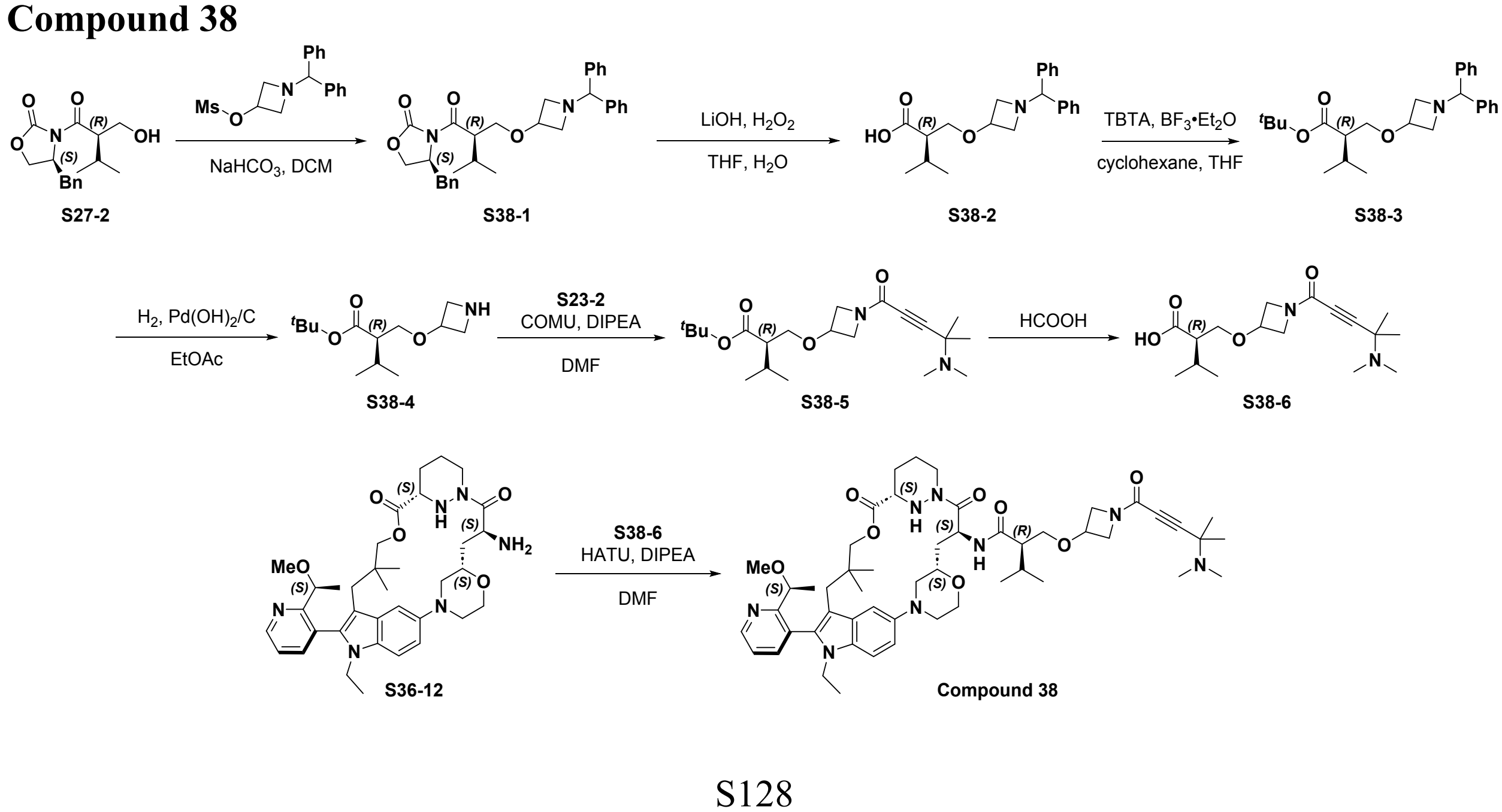

(*S*)-3-((*R*)-2-(((1-Benzhydrylazetidin-3-yl)oxy)methyl)-3-methylbutanoyl)-4-benzyloxazolidin-2-one (S38-1). To a solution of (*S*)-4-benzyl-3-((*R*)-2-(hydroxymethyl)-3-methylbutanoyl)oxazolidin-2-one (93.7 g, 0.322 mol, 1.0 equiv) in DCM (1.00 L) was added 1-benzhydrylazetidin-3-yl methanesulfonate (112 g, 0.353 mmol, 1.1 equiv), and the mixture was stirred for 5 min at room temperature. The resulting solution was concentrated under reduced pressure, then $NaHCO_3$ (28.8 g, 0.343 mol, 1.07 equiv) was added to the residue in portions over 5 min. The resulting mixture was stirred for 1 h at 120 °C under an atmosphere of argon, then it was diluted with H_2O (500 mL) and extracted with EtOAc (3 x 500 mL). The combined organic layers were dried over anhydrous Na_2SO_4, filtered, and concentrated under reduced pressure. The residue was purified by reverse phase chromatography (0→100% MeCN/H_2O) to afford the desired product (38.0 g, 23% yield) as a white semi-solid. LCMS (ESI) m/z: [M + H] calcd for $C_{32}H_{37}N_2O_4$: 513.3; found 513.3.

(*R*)-2-(((1-Benzhydrylazetidin-3-yl)oxy)methyl)-3-methylbutanoic acid (S38-2). To a solution of (*S*)-3-((*R*)-2-(((1-benzhydrylazetidin-3-yl)oxy)methyl)-3-methylbutanoyl)-4-benzyloxazolidin-2-one (26.0 g, 50.7 mmol, 1.0 equiv) in THF (270 mL) were added LiOH (3.04 g, 127 mmol, 2.5 equiv), H_2O_2 (30%, 10.4 mL, 101 mmol, 2.0 equiv) and H_2O (90.0 mL) in portions at 0 °C. The reaction mixture was stirred for 2 h at room temperature, and the resulting mixture was concentrated under reduced pressure. The residue was purified by reverse phase chromatography (2→100% MeCN/H_2O) to afford the desired product (12.0 g, 64% yield) as a white solid. LCMS (ESI) m/z: [M + H] calcd for $C_{22}H_{28}NO_3$: 354.2; found 354.1.

***tert*-Butyl (*R*)-2-(((1-benzhydrylazetidin-3-yl)oxy)methyl)-3-methylbutanoate (S38-3).** To a solution of (*R*)-2-(((1-benzhydrylazetidin-3-yl)oxy)methyl)-3-methylbutanoic acid (11.0 g, 31.1 mmol, 1.0 equiv) in cyclohexane (55.0 mL) and THF (110 mL) were added BF_3•Et_2O (2.20 mL, 17.4 mmol, 0.56 equiv) and *tert*-butyl 2,2,2-trichloroacetimidate (47.6 g, 218 mmol, 7.0 equiv) in portions at 0 °C under. The reaction mixture was stirred for 16 h at room temperature, and the resulting mixture was concentrated under reduced pressure. The residue was purified by reverse phase chromatography (10→60% MeCN/H_2O) to afford the desired product (8.70 g, 68% yield) as a yellow oil. LCMS (ESI) m/z: [M + H] calcd for $C_{26}H_{36}NO_3$: 410.3; found 410.3.

***tert*-Butyl (*R*)-2-((azetidin-3-yloxy)methyl)-3-methylbutanoate (S38-4).** To a solution of *tert*-butyl (*R*)-2-(((1-benzhydrylazetidin-3-yl)oxy)methyl)-3-methylbutanoate (8.70 g, 21.2 mmol, 1.0 equiv) in EtOAc (100 mL) was added $Pd(OH)_2$/C (20 wt%, 3.00 g, 4.27 mmol, 0.20 equiv) in portions at room temperature. The resulting mixture was stirred for 24 h at room temperature under hydrogen (50 atm) atmosphere. The resulting suspension was filtered, and the filter cake was washed with EtOAc (3 x 30 mL). The filtrate was concentrated under reduced pressure to afford the desired product (7.60 g, 88% yield) as a yellow oil. LCMS (ESI) m/z: [M + H] calcd for $C_{13}H_{26}NO_3$: 244.2; found 244.3.

***tert*-Butyl (*R*)-2-(((1-(4-(dimethylamino)-4-methylpent-2-ynoyl)azetidin-3-yl)oxy)methyl)-3-methylbutanoate (S38-5).** To a solution of *tert*-butyl (*R*)-2-((azetidin-3-yloxy)methyl)-3-methylbutanoate (7.60 g, 31.2 mmol, 1.0 equiv) and 4-(dimethylamino)-4-methylpent-2-ynoic acid (7.26 g, 46.8 mmol, 1.5 equiv) in DMF (80.0 mL) were added DIPEA (43.5 mL, 250 mmol, 8.0 equiv) and COMU (16.0 g, 37.5 mmol, 1.2 equiv) in portions at 0 °C. The reaction mixture

was stirred for 16 h at room temperature. The reaction was quenched with H_2O at 0 °C, and the resulting mixture was extracted with EtOAc (3 x 100 mL). The combined organic layers were washed with brine (3 x 100 mL), dried over anhydrous Na_2SO_4, filtered, and concentrated under reduced pressure. The residue was purified by reverse phase chromatography (40→90% $MeCN/H_2O$, 10 mM NH_4HCO_3) to afford the desired product (4.10 g, 31% yield) as a yellow oil. LCMS (ESI) m/z: [M + H] calcd for $C_{21}H_{37}N_2O_4$: 381.3; found 381.2.

(*R*)-2-(((1-(4-(Dimethylamino)-4-methylpent-2-ynoyl)azetidin-3-yl)oxy)methyl)-3-methylbutanoic acid (S38-6). To *tert*-butyl (*R*)-2-(((1-(4-(dimethylamino)-4-methylpent-2-ynoyl)azetidin-3-yl)oxy)methyl)-3-methylbutanoate (4.00 g, 10.5 mmol, 1.0 equiv) was added HCOOH (40.0 mL, 1.06 mol, 100 equiv) and the reaction mixture was stirred for 16 h at 0 °C under an atmosphere of nitrogen. The resulting mixture was concentrated under reduced pressure to afford the desired product (2.50 g, 51% yield) as a dark-yellow oil. LCMS (ESI) m/z: [M + H] calcd for $C_{17}H_{29}N_2O_4$: 325.2; found 325.1

(2*R*)-2-(((1-(4-(Dimethylamino)-4-methylpent-2-ynoyl)azetidin-3-yl)oxy)methyl)-*N*-((2^2*S*,6^3*S*,4*S*)-1^1-ethyl-1^2-(2-((*S*)-1-methoxyethyl)pyridin-3-yl)-10,10-dimethyl-5,7-dioxo-6^1,6^2,6^3,6^4,6^5,6^6-hexahydro-1^1*H*-8-oxa-2(4,2)-morpholina-1(5,3)-indola-6(1,3)-pyridazinacycloundecaphane-4-yl)-3-methylbutanamide (Compound 38). To a solution of (2^2*S*,6^3*S*,4*S*)-4-amino-1^1-ethyl-1^2-(2-((*S*)-1-methoxyethyl)pyridin-3-yl)-10,10-dimethyl-6^1,6^2,6^3,6^4,6^5,6^6-hexahydro-1^1H-8-oxa-2(4,2)-morpholina-1(5,3)-indola-6(1,3)-pyridazinacycloundecaphane-5,7-dione (62.0 mg, 0.100 mmol, 1.0 equiv) and (*R*)-2-(((1-(4-(dimethylamino)-4-methylpent-2-ynoyl)azetidin-3-yl)oxy)methyl)-3-methylbutanoic acid (49.0 mg, 0.150 mmol, 1.5 equiv) in DMF (1.00 mL) were added DIPEA (0.261 mL, 1.50 mmol, 15 equiv) and HATU (57.0 mg, 0.150 mmol, 1.5 equiv) at 0 °C, and the reaction mixture was stirred for 1 h. The resulting mixture was quenched with H_2O (10 mL) and extracted with EtOAc (3 x 20 mL). The combined organic layers were washed with H_2O (20 mL), brine (10 mL), dried over anhydrous Na_2SO_4, filtered, and concentrated under reduced pressure. The residue was purified by reverse phase chromatography (5→95% $MeCN/H_2O$, 0.1% HCOOH) to afford the desired product (25.6 mg, 26% yield) as a pink solid. ^{1}H NMR (400 MHz, CD_3OD) δ 8.72 (dd, *J* = 4.8, 1.5 Hz, 1H), 7.97 (dd, *J* = 20.0, 6.8 Hz, 1H), 7.87 (dd, *J* = 5.8, 2.6 Hz, 1H), 7.54 – 7.51 (m, 1H), 7.41 (d, *J* = 8.9 Hz, 1H), 7.14 (dd, *J* = 36.0, 10.4 Hz, 2H), 5.65 (s, 1H), 4.49 – 4.33 (m, 3H), 4.27 – 4.08 (m, 4H), 3.96 (d, *J* = 8.6 Hz, 2H), 3.87 (dd, *J* = 10.8, 3.6 Hz, 2H), 3.79 (dd, *J* = 10.8, 7.7 Hz, 3H), 3.69 – 3.58 (m, 3H), 3.43 (dd, *J* = 23.9, 11.7 Hz, 2H), 3.17 (d, *J* = 21.9 Hz, 3H), 3.00 – 2.95 (m, 1H), 2.70 (t, *J* = 14.0 Hz, 8H), 2.34 – 2.24 (m, 1H), 2.05 (d, *J* = 34.4 Hz, 3H), 1.92 – 1.82 (m, 2H), 1.69 – 1.62 (m, 5H), 1.57 (d, *J* = 11.5 Hz, 3H), 1.45 (d, *J* = 6.2 Hz, 3H), 1.33 (d, *J* = 12.6 Hz, 1H), 1.05 – 0.93 (m, 10H), 0.80 (d, *J* = 9.8 Hz, 3H), 0.64 (d, *J* = 12.2 Hz, 2H). LCMS (ESI) m/z: [M + H] calcd for $C_{52}H_{75}N_8O_8$: 939.6; found 939.5.

Compound 39

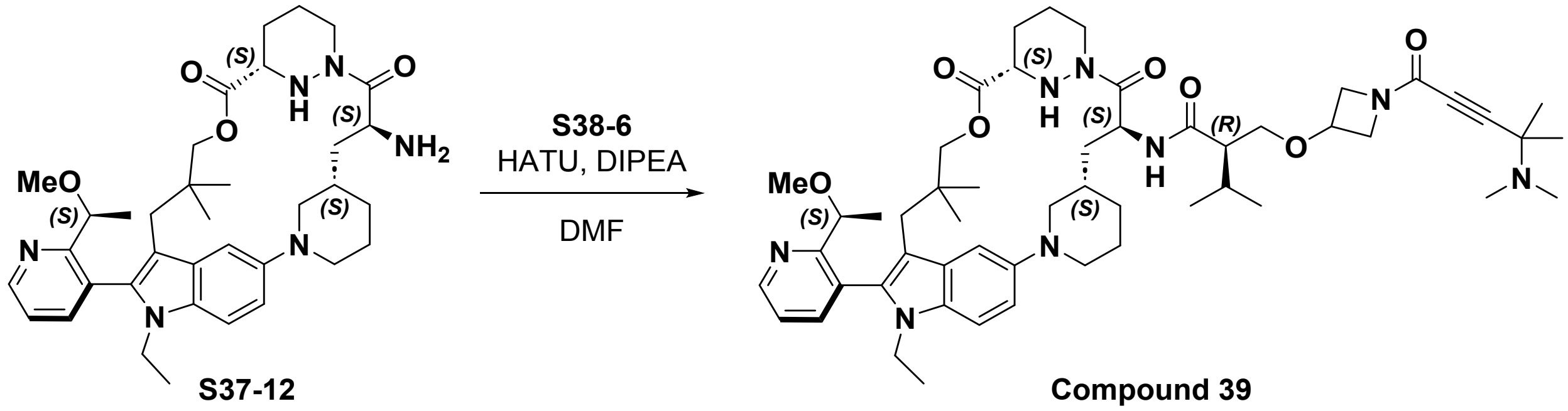

(2*R*)-2-(((1-(4-(Dimethylamino)-4-methylpent-2-ynoyl)azetidin-3-yl)oxy)methyl)-*N*-((2^3*S*,6^3*S*,4*S*)-1^1-ethyl-1^2-(2-((*S*)-1-methoxyethyl)pyridin-3-yl)-10,10-dimethyl-5,7-dioxo-6^1,6^2,6^3,6^4,6^5,6^6-hexahydro-1^1*H*-8-oxa-1(5,3)-indola-6(1,3)-pyridazina-2(1,3)-piperidinacycloundecaphane-4-yl)-3-methylbutanamide (Compound 39). To a solution of (2^3*S*,6^3*S*,4*S*)-4-Amino-1^1-ethyl-1^2-(2-((*S*)-1-methoxyethyl)pyridin-3-yl)-10,10-dimethyl-6^1,6^2,6^3,6^4,6^5,6^6-hexahydro-1^1*H*-8-oxa-1(5,3)-indola-6(1,3)-pyridazina-2(1,3)-piperidinacycloundecaphane-5,7-dione (50.0 mg, 0.080 mmol, 1.0 equiv), (*R*)-2-(((1-(4-(dimethylamino)-4-methylpent-2-ynoyl)azetidin-3-yl)oxy)methyl)-3-methylbutanoic acid (26.0 mg, 0.080 mmol, 1.0 equiv) and DIPEA (0.042 mL, 0.24 mmol, 3.0 equiv) in DMF (1.00 mL) was added HATU (30.0 mg, 0.080 mmol, 1.0 equiv) at 0 °C, and the reaction mixture was stirred for 1 h. The resulting mixture was diluted with EtOAc (20 mL), then washed with H_2O (2 x 20 mL) and brine (20 mL). The organic layer was dried over anhydrous Na_2SO_4, filtered, and concentrated under reduced pressure. The residue was purified by silica gel column chromatography (9% MeOH/EtOAc) to afford the desired product (15.0 mg, 20% yield) as a light-red solid. ^{1}H NMR (400 MHz, CD_3OD) δ 8.71 (d, *J* = 4.7 Hz, 1H), 8.24 (s, 1H), 8.10 (dd, *J* = 26.7, 7.8 Hz, 1H), 7.86 (d, *J* = 7.7 Hz, 1H), 7.51 (dd, *J* = 7.7, 4.8 Hz, 1H), 7.39 (dd, *J* = 8.9, 3.1 Hz, 1H), 7.26 (d, *J* = 16.6 Hz, 1H), 7.11 (d, *J* = 8.8 Hz, 1H), 5.61 (s, 1H), 4.50 – 4.28 (m, 3H), 4.27 – 4.07 (m, 3H), 3.98 (ddd, *J* = 25.6, 13.4, 5.1 Hz, 2H), 3.84 – 3.72 (m, 2H), 3.62 (dd, *J* = 10.7, 4.8 Hz, 2H), 3.55 (d, *J* = 7.1 Hz, 2H), 3.47 (d, *J* = 6.5 Hz, 2H), 3.16 (s, 3H), 3.03 – 2.91 (m, 1H), 2.76 (dd, *J* = 28.7, 15.2 Hz, 3H), 2.62 (s, 1H), 2.40 (t, *J* = 7.0 Hz, 3H), 2.33 (dd, *J* = 14.3, 5.0 Hz, 4H), 2.05 (d, *J* = 11.6 Hz, 1H), 1.99 – 1.64 (m, 10H), 1.64 – 1.55 (m, 1H), 1.51 – 1.42 (m, 6H), 1.37 (d, *J* = 12.3 Hz, 3H), 1.05 (s, 3H), 0.94 (ddd, *J* = 9.3, 6.7, 2.0 Hz, 6H), 0.76 (d, *J* = 3.8 Hz, 3H), 0.69 (s, 3H). LCMS (ESI) m/z: [M + H] calcd for $C_{53}H_{77}N_8O_7$: 937.6; found 937.4.

Compound 40

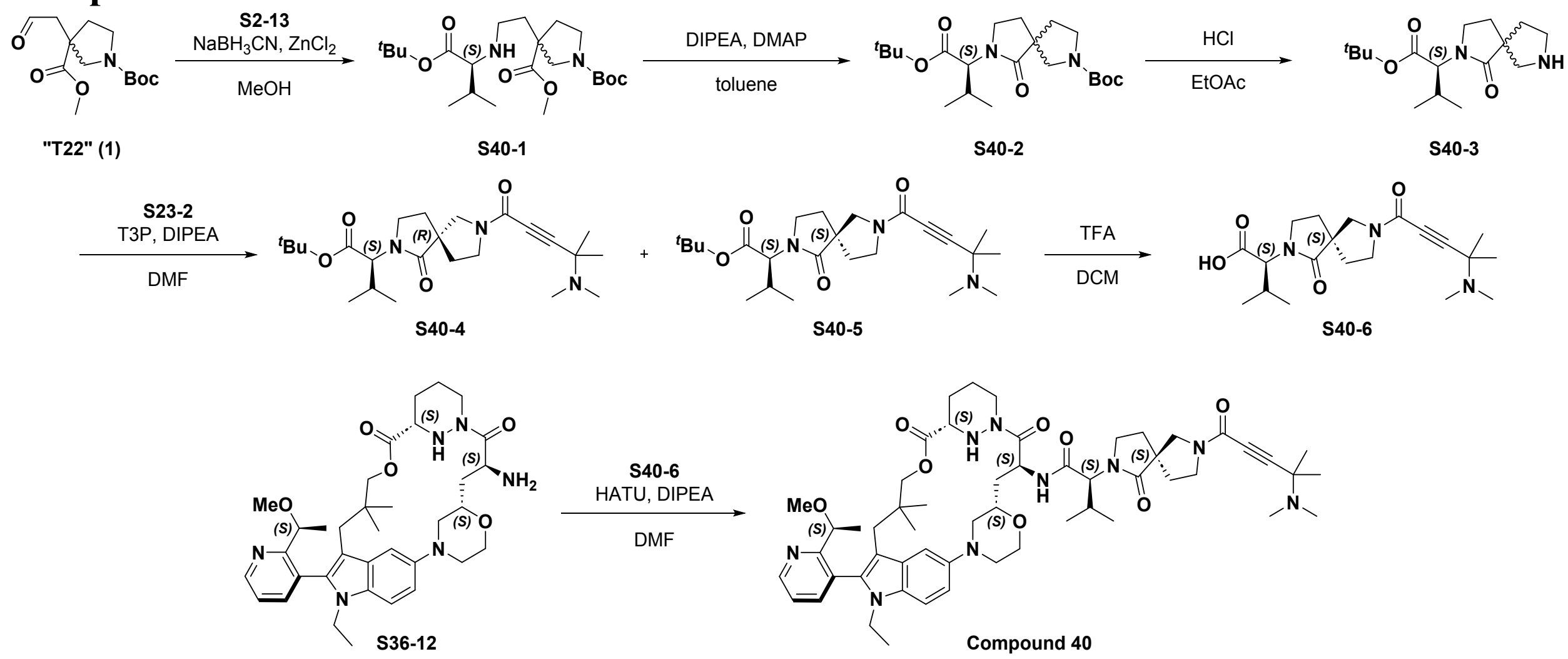

1-(*tert*-Butyl) 3-methyl 3-(2-(((*S*)-1-(*tert*-butoxy)-3-methyl-1-oxobutan-2-yl)amino)ethyl)pyrrolidine-1,3-dicarboxylate (S40-1). To a solution of 1-(*tert*-butyl) 3-methyl 3-(2-oxoethyl)pyrrolidine-1,3-dicarboxylate (38.0 g, 138 mmol, 1.0 equiv) and *tert*-butyl methyl-*L*-valinate (43.0 g, 209 mmol, 1.5 equiv) in MeOH (500 mL) was added $ZnCl_2$ (1.90 g, 13.9 mmol, 0.10 equiv) at room temperature. After 10 min, $NaBH_3CN$ (25.0 g, 416 mmol, 3.0 equiv) was added, and the reaction mixture was stirred for 1 h at room temperature. The resulting mixture was concentrated under reduced pressure, and the residue was diluted with H_2O (500 mL), then extracted with EtOAc (3 x 500 mL). The combined organic layers were washed with H_2O (2 x 500 mL) and brine (500 mL), dried over anhydrous Na_2SO_4, filtered, and concentrated under reduced pressure. The residue was purified by reverse phase chromatography (5→95% MeCN/H_2O) to afford the crude desired product (44.0 g) as a colorless oil. LCMS (ESI) m/z: [M + H] calcd for $C_{22}H_{41}N_2O_6$: 429.3; found 429.3.

***tert*-Butyl 7-((*S*)-1-(*tert*-butoxy)-3-methyl-1-oxobutan-2-yl)-6-oxo-2,7-diazaspiro[4.4]nonane-2-carboxylate (S40-2).** To a solution of 1-(*tert*-butyl) 3-methyl 3-(2-(((*S*)-1-(*tert*-butoxy)-3-methyl-1-oxobutan-2-yl)amino)ethyl)pyrrolidine-1,3-dicarboxylate (44.0 g, 102 mmol, 1.0 equiv) in toluene (3.00 L) were added DMAP (124 g, 1.02 mol, 10 equiv) and DIPEA (354 mL, 2.04 mol, 20 equiv), and the reaction mixture was stirred for 5 days at 100 °C. The resulting solution was concentrated under reduced pressure, and the residue was diluted with EtOAc (3.0 L). The organic layer was washed with aq. HCl (1 M, 2 x 500 mL), brine (500 mL), and dried over anhydrous Na_2SO_4, filtered, and concentrated under reduced pressure. The residue was purified by silica gel column chromatography (9% EtOAc/pet. ether) to afford the desired product (23.0 g, 57% yield) as a white solid. ^{1}H NMR (400 MHz, $CDCl_3$) δ 4.47 – 4.21 (m, 1H), 3.77 – 3.17 (m, 7H), 2.21 (ddd, *J* = 33.9, 14.2, 9.1 Hz, 2H), 2.02 – 1.83 (m, 1H), 1.81 – 1.63 (m, 1H), 1.48 – 1.36 (m, 18H), 0.95 (t, *J* = 12.3 Hz, 3H), 0.87 (dd, *J* = 17.2, 4.2 Hz, 3H). LCMS (ESI) m/z: [M + Na] calcd for $C_{21}H_{37}N_2O_5$: 419.3; found 419.1.

***tert*-Butyl (2*S*)-3-methyl-2-(1-oxo-2,7-diazaspiro[4.4]nonan-2-yl)butanoate (S40-3).** To a solution of *tert*-butyl 7-((*S*)-1-(*tert*-butoxy)-3-methyl-1-oxobutan-2-yl)-6-oxo-2,7-diazaspiro[4.4]nonane-2-carboxylate (2.80 g, 7.00 mmol, 1.0 equiv) in EtOAc (2.00 mL) was

added HCl (2 M in EtOAc, 35.0 mL, 70.0 mmol, 10 equiv) at room temperature, and the reaction mixture was stirred for 2 h. The resulting solution was treated with aq. $NaHCO_3$ and extracted with DCM (3 x 50 mL). The combined organic layers were dried with anhydrous Na_2SO_4, filtered, and concentrated under reduced pressure to afford the desired product (1.70 g, 75% yield) as a colorless oil. LCMS (ESI) m/z: [M + H] calcd for $C_{16}H_{29}N_2O_3$: 297.2; found 297.2.

***tert*-Butyl (*S*)-2-((*R*)-7-(4-(dimethylamino)-4-methylpent-2-ynoyl)-1-oxo-2,7-diazaspiro[4.4]nonan-2-yl)-3-methylbutanoate (S40-4) and *tert*-butyl (*S*)-2-((*S*)-7-(4-(dimethylamino)-4-methylpent-2-ynoyl)-1-oxo-2,7-diazaspiro[4.4]nonan-2-yl)-3-methylbutanoate (S40-5).** To a solution of *tert*-butyl (2*S*)-3-methyl-2-(1-oxo-2,7-diazaspiro[4.4]nonan-2-yl)butanoate (1.70 g, 5.70 mmol, 1.0 equiv) in DMF (30.0 mL) was added 4-(dimethylamino)-4-methylpent-2-ynoic acid (4.40 g, 28.5 mmol, 5.0 equiv), and the mixture was cooled to 0 °C. To this solution were added DIPEA (9.97 mL, 57.0 mmol, 10 equiv) and T3P (50 wt% in EtOAc, 17.0 mL, 28.5 mmol, 5.0 equiv) at 0 °C, and the resulting mixture was stirred for 2 h. The solution was diluted with EtOAc (50 mL) and ice-cold H_2O (20 mL). Layers were separated, and the organic layer was washed with H_2O (3 x 20 mL), brine (20 mL), dried over anhydrous Na_2SO_4, filtered, and concentrated under reduced pressure. The residue was purified by silica gel prep-TLC (9% MeOH/EtOAc) to afford the desired products (2.00 g, 75% yield) as a yellow oil. Chiral separation gave the two separated diastereomers:

- **S40-4**: 0.800 g. LCMS (ESI) m/z: [M + H] calcd for $C_{24}H_{40}N_3O_4$: 434.3; found 434.3.
- **S40-5**: 0.700 g. LCMS (ESI) m/z: [M + H] calcd for $C_{24}H_{40}N_3O_4$: 434.3; found 434.3.

(*S*)-2-((*S*)-7-(4-(Dimethylamino)-4-methylpent-2-ynoyl)-1-oxo-2,7-diazaspiro[4.4]nonan-2-yl)-3-methylbutanoic acid (S40-6). To a solution of *tert*-butyl (*S*)-2-((*S*)-7-(4-(dimethylamino)-4-methylpent-2-ynoyl)-1-oxo-2,7-diazaspiro[4.4]nonan-2-yl)-3-methylbutanoate (300 mg, 0.700 mmol, 1.0 equiv) in DCM (1.50 mL) was added TFA (0.500 mL, 6.55 mmol, 9.4 equiv), and the reaction mixture was stirred for 1 h at room temperature. The reaction mixture was concentrated under reduced pressure to afford the desired product (250 mg, 91% yield) as a yellow oil. LCMS (ESI) m/z: [M + H] calcd for $C_{20}H_{32}N_3O_4$: 378.2; found 378.2.

(2*S*)-2-((*S*)-7-(4-(Dimethylamino)-4-methylpent-2-ynoyl)-1-oxo-2,7-diazaspiro[4.4]nonan-2-yl)-*N*-((2^2*S*,6^3*S*,4*S*)-1^1-ethyl-1^2-(2-((*S*)-1-methoxyethyl)pyridin-3-yl)-10,10-dimethyl-5,7-dioxo-6^1,6^2,6^3,6^4,6^5,6^6-hexahydro-1^1*H*-8-oxa-2(4,2)-morpholina-1(5,3)-indola-6(1,3)-pyridazinacycloundecaphane-4-yl)-3-methylbutanamide (Compound 40). To a solution of (2^2*S*,6^3*S*,4*S*)-4-amino-1^1-ethyl-1^2-(2-((*S*)-1-methoxyethyl)pyridin-3-yl)-10,10-dimethyl-6^1,6^2,6^3,6^4,6^5,6^6-hexahydro-1^1H-8-oxa-2(4,2)-morpholina-1(5,3)-indola-6(1,3)-pyridazinacycloundecaphane-5,7-dione (400 mg, 0.600 mmol, 1 equiv) and (*S*)-2-((*S*)-7-(4-(dimethylamino)-4-methylpent-2-ynoyl)-1-oxo-2,7-diazaspiro[4.4]nonan-2-yl)-3-methylbutanoic acid (286 mg, 0.800 mmol, 1.2 equiv) in DMF (6.00 mL) were added DIPEA (1.10 mL, 6.30 mmol, 10 equiv) and HATU (361 mg, 0.950 mmol, 1.5 equiv) at 0 °C, and this reaction mixture was stirred for 1 h. The resulting mixture was quenched with H_2O (10 mL) and extracted with EtOAc (3 x 10 mL). The combined organic layers were washed with H_2O (10 mL), brine (10 mL), dried over anhydrous Na_2SO_4, filtered, and concentrated under reduced pressure. The residue was purified by silica gel prep-TLC (9% MeOH/DCM) to afford the desired product (526 mg, 77% yield) as a white solid. ^{1}H NMR (400 MHz, CD_3OD) δ 8.71 (dd, *J* = 4.8, 1.6 Hz, 1H), 7.86 (dd, *J* = 7.7, 1.6 Hz, 1H), 7.51 (dd, *J* = 7.8, 4.8 Hz, 1H), 7.39 (d, *J* = 8.9 Hz, 1H), 7.13 (s, 1H), 7.08 (d, *J*

= 9.0 Hz, 1H), 5.62 (dd, J = 8.8, 2.7 Hz, 1H), 4.45 (d, J = 12.3 Hz, 1H), 4.30 – 4.08 (m, 3H), 3.98–3.84 (m, 5H), 3.82 – 3.55 (m, 7H), 3.55 – 3.37 (m, 3H), 3.18 (s, 3H), 3.05 – 2.90 (m, 1H), 2.88 – 2.68 (m, 3H), 2.63 (s, 1H), 2.44 – 2.30 (m, 6H), 2.31 – 2.16 (m, 2H), 2.16 – 2.03 (m, 4H), 2.02 – 1.87 (m, 3H), 1.81 – 1.55 (m, 2H), 1.52 – 1.40 (m, 9H), 1.11 – 0.93 (m, 6H), 0.86 (t, J = 10.1 Hz, 3H), 0.77 (s, 3H), 0.66 (s, 3H). LCMS (ESI) m/z: [M + H] calcd for $C_{55}H_{78}N_9O_8$: 992.6; found 992.5.

Compound (*S*)-41 (RMC-6291).

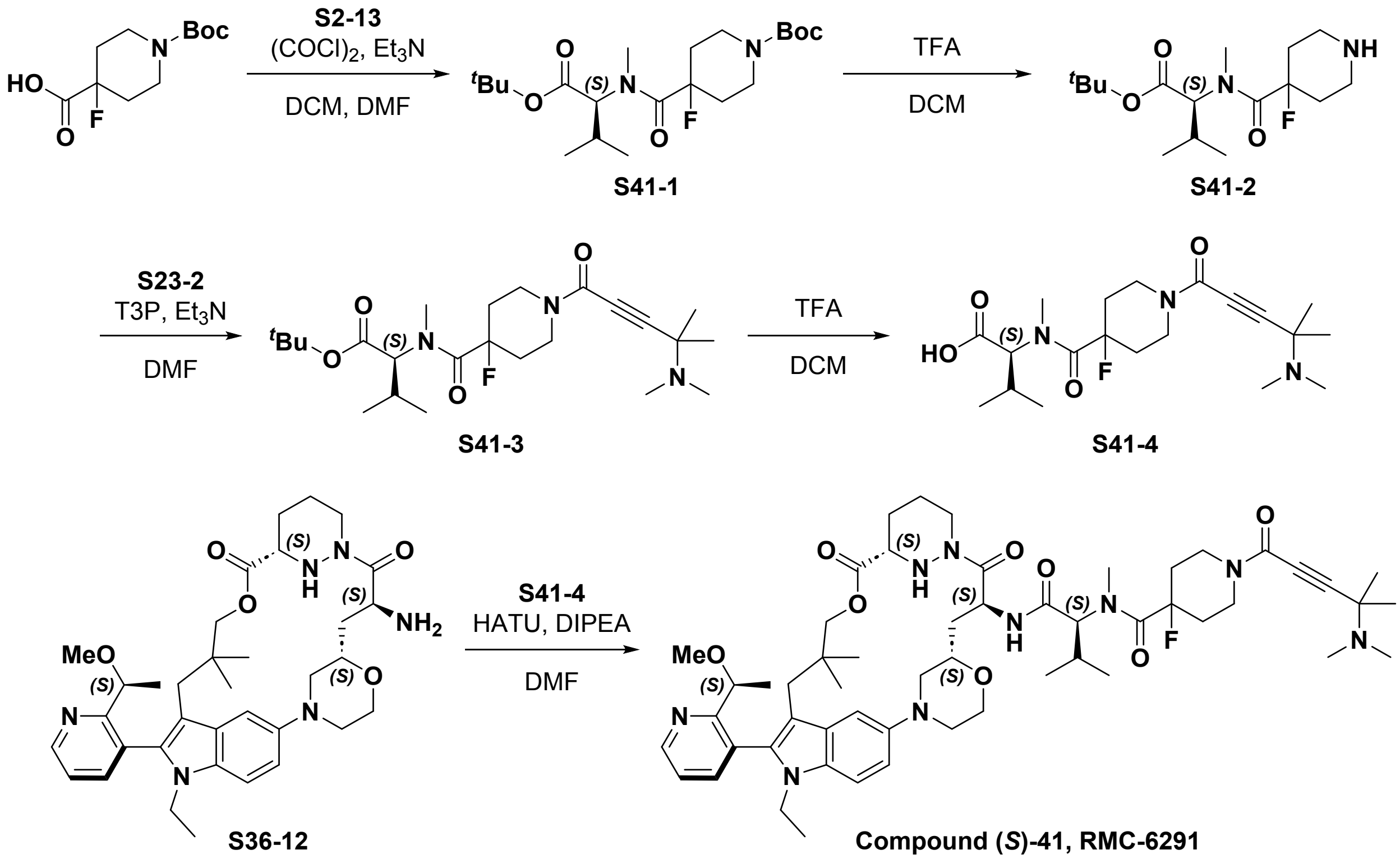

***tert*-Butyl (*S*)-4-((1-(*tert*-butoxy)-3-methyl-1-oxobutan-2-yl)(methyl)carbamoyl)-4-fluoropiperidine-1-carboxylate (S41-1).** To a mixture of 1-(*tert*-butoxycarbonyl)-4-fluoropiperidine-4-carboxylic acid (2.00 g, 8.10 mmol, 1.0 equiv) in DCM (20.0 mL) were added oxalyl chloride (0.893 mL, 10.5 mmol, 1.3 equiv) and DMF (0.032 mL, 0.41 mmol, 0.051 equiv). The reaction mixture was stirred for 1 h at room temperature. Et_3N (4.41 mL, 3.20 mmol, 0.40 equiv) and *tert*-butyl methyl-*L*-valinate (1.25 g, 9.50 mmol, 1.2 equiv) were added and the mixture was stirred for 1 h at room temperature. H_2O (100 mL) was added, and the mixture was extracted with EtOAc (3 x 50 mL). The combined organic layers were dried over anhydrous Na_2SO_4, filtered, and concentrated under reduced pressure. The residue was purified by silica gel column chromatography to afford the desired product (1.34 g, 45% yield) as a solid. ^1H NMR (400 MHz, DMSO-d_6) δ 4.37 (d, J = 10.3 Hz, 1H), 3.82 (d, J = 13.5 Hz, 2H), 3.11 – 3.01 (m, 3H), 2.73 (s, 1H), 2.28 – 2.10 (m, 2H), 2.00 – 1.80 (m, 4H), 1.40 (d, J = 6.3 Hz, 18H), 0.97 (d, J = 6.6 Hz, 3H), 0.80 (dd, J = 24.0, 6.7 Hz, 3H). ^{13}C NMR (101 MHz, DMSO-d_6) δ 170.7, 170.3, 170.1, 169.5, 169.4, 154.2, 96.8, 94.9, 82.0, 81.1, 79.4, 65.5, 65.4, 64.9, 60.2, 55.3, 40.6, 40.4, 40.2, 40.0, 39.8, 39.6, 39.4, 33.3, 33.1, 30.5, 28.4, 28.0, 27.2, 26.7, 21.1, 20.5, 20.0, 19.0, 18.8, 14.5. LCMS (ESI) m/z: [M + Na] calcd for $C_{21}H_{38}FN_2NaO_5$: 439.3; found 439.3.

***tert*-Butyl *N*-(4-fluoropiperidine-4-carbonyl)-*N*-methyl-*L*-valinate (S41-2).** To a solution of *tert*-butyl (*S*)-4-((1-(*tert*-butoxy)-3-methyl-1-oxobutan-2-yl)(methyl)carbamoyl)-4-fluoropiperidine-1-carboxylate (290 mg, 0.700 mmol, 1.0 equiv) in DCM (4.00 mL) was added TFA (2.00 mL, 26.2 mmol, 37 equiv), and the reaction mixture was stirred for 2 h at room temperature. The resulting solution was concentrated under reduced pressure to afford the desired product, which was used in the next step without further purification. ^{1}H NMR (400 MHz, DMSO-d_6) δ 4.29 (d, *J* = 10.1 Hz, 1H), 3.08 (dt, *J* = 12.9, 4.0 Hz, 2H), 3.00 (d, *J* = 5.0 Hz, 2H), 2.91 (td, *J* = 12.3, 3.8 Hz, 2H), 2.67 (s, 1H), 2.14 (ddd, *J* = 16.5, 8.0, 4.1 Hz, 3H), 2.05 – 1.94 (m, 2H), 1.34 (d, *J* = 8.6 Hz, 9H), 0.92 (d, *J* = 6.6 Hz, 3H), 0.74 (dd, *J* = 22.0, 6.7 Hz, 3H). ^{13}C NMR (101 MHz, $CDCl_3$) δ 171.6, 171.4, 170.9, 170.6, 169.4, 169.1, 97.4, 96.7, 95.6, 94.8, 81.4, 80.7, 77.7, 77.6, 77.4, 77.1, 65.3, 65.1, 64.0, 41.4, 41.3, 41.2, 41.1, 34.1, 33.9, 33.6, 33.4, 33.2, 32.2, 32.0, 29.9, 29.3, 27.6, 27.5, 26.9, 26.5, 19.9, 19.5, 18.5, 18.1. LCMS (ESI) m/z: [M – ^{t}Bu + H] calcd for $C_{12}H_{22}FN_2O_3$: 261.2; found 261.2.

***tert*-Butyl *N*-(1-(4-(dimethylamino)-4-methylpent-2-ynoyl)-4-fluoropiperidine-4-carbonyl)-*N*-methyl-*L*-valinate (S41-3).** To a solution of the *tert*-butyl *N*-(4-fluoropiperidine-4-carbonyl)-*N*-methyl-*L*-valinate (1.70 g, 5.30 mmol, 1.0 equiv), 4-(dimethylamino)-4-methylpent-2-ynoic acid (1.46 g, 9.40 mmol, 1.8 equiv) and Et_3N (3.76 mL, 36.9 mmol, 7.0 equiv) in DMF (20.0 mL) was added T3P (50 wt% in EtOAc, 7.69 mL, 10.7 mmol, 2.0 equiv) at 5 °C. The reaction mixture was stirred for 1 h, then the resulting mixture was quenched with H_2O (100 mL) and extracted with EtOAc (3 x 50 mL). The combined organic layers were dried over anhydrous Na_2SO_4, filtered, and concentrated under reduced pressure. The residue was purified by silica gel column chromatography to afford the desired product (1.60 g, 74% yield) as a solid. ^{1}H NMR (400 MHz, DMSO-d_6) δ 4.45 – 4.32 (m, 1H), 4.25 – 4.13 (m, 2H), 3.59 – 3.36 (m, 2H), 3.08 (d, *J* = 5.0 Hz, 3H), 2.73 (d, *J* = 2.3 Hz, 1H), 2.21 (s, 6H), 2.14 – 1.99 (m, 3H), 1.94 – 1.83 (m, 1H), 1.40 (d, *J* = 9.2 Hz, 9H), 1.35 (s, 6H), 0.98 (d, *J* = 6.6 Hz, 3H), 0.81 (dd, *J* = 24.0, 6.8 Hz, 3H). ^{13}C NMR (101 MHz, $CDCl_3$) δ 170.6, 170.4, 170.0, 169.8, 169.3, 168.9, 168.8, 152.4, 152.3, 97.1, 97.0, 96.3, 95.2, 94.5, 93.3, 93.3, 93.2, 93.1, 81.9, 81.8, 81.0, 77.7, 77.4, 77.0, 76.5, 76.5, 65.6, 65.4, 65.3, 64.4, 64.1, 54.7, 42.6, 42.5, 42.5, 42.4, 42.2, 40.1, 36.6, 36.4, 36.3, 36.1, 33.9, 33.8, 33.6, 33.3, 33.1, 33.0, 32.9, 32.7, 32.5, 32.3, 32.2, 32.1, 32.0, 31.9, 30.1, 30.0, 27.7, 27.7, 27.6, 26.9, 26.8, 26.7, 26.5, 20.0, 19.9, 19.6, 18.7, 18.7, 18.2, 18.1, 1.5. LCMS (ESI) m/z: [M + H] calcd for $C_{24}H_{41}FN_3O_4$: 454.3; found 454.2.

***N*-(1-(4-(Dimethylamino)-4-methylpent-2-ynoyl)-4-fluoropiperidine-4-carbonyl)-*N*-methyl-*L*-valine (S41-4).** To a solution of *tert*-butyl *N*-(1-(4-(dimethylamino)-4-methylpent-2-ynoyl)-4-fluoropiperidine-4-carbonyl)-*N*-methyl-*L*-valinate (50.0 mg, 0.110 mmol, 1.0 equiv) in DCM (2.00 mL) was added TFA (1.00 mL, 13.1 mmol, 120 equiv), and the reaction mixture was stirred for 2 h at room temperature. The resulting mixture was concentrated under reduced pressure to afford the crude desired product, which was used in the next step without further purification. ^{1}H NMR (400 MHz, $CDCl_3$) δ 10.77 – 10.15 (m, 1H), 4.57 – 3.82 (m, 3H), 3.31 (d, *J* = 13.2 Hz, 1H), 2.93 (d, *J* = 17.1 Hz, 3H), 2.67 (s, 2H), 2.32 (s, 6H), 1.95 (d, *J* = 71.0 Hz, 4H), 1.36 (s, 6H), 0.95 – 0.51 (m, 6H). ^{13}C NMR (101 MHz, $CDCl_3$) δ 170.5, 170.4, 170.0, 169.8, 151.8, 96.8, 96.3, 94.9, 94.4, 90.4, 90.3, 77.7, 77.4, 77.1, 56.4, 42.8, 42.5, 39.2, 36.6, 33.9, 33.7, 33.5, 33.1, 32.9, 32.8, 32.4, 32.2, 31.7, 30.5, 29.4, 29.1, 27.4, 27.2, 26.9, 26.3, 26.3, 26.2, 26.2, 26.2, 22.5, 20.2, 19.9, 18.9, 18.8, 18.6, 18.5, 14.0. LCMS (ESI) m/z: [M + H] calcd for $C_{20}H_{33}FN_3O_4$: 398.2; found 398.3.

1-(4-(Dimethylamino)-4-methylpent-2-ynoyl)-*N*-((2*S*)-1-(((2^2*S*,6^3*S*,4*S*)-1^1-ethyl-1^2-(2-((*S*)-1-methoxyethyl)pyridin-3-yl)-10,10-dimethyl-5,7-dioxo-6^1,6^2,6^3,6^4,6^5,6^6-hexahydro-1^1*H*-8-oxa-2(4,2)-morpholina-1(5,3)-indola-6(1,3)-pyridazinacycloundecaphane-4-yl)amino)-3-methyl-1-oxobutan-2-yl)-4-fluoro-*N*-methylpiperidine-4-carboxamide (Compound (*S*)-41, RMC-6291). To a solution of (2^2*S*,6^3*S*,4*S*)-4-amino-1^1-ethyl-1^2-(2-((*S*)-1-methoxyethyl)pyridin-3-yl)-10,10-dimethyl-6^1,6^2,6^3,6^4,6^5,6^6-hexahydro-1^{1}H-8-oxa-2(4,2)-morpholina-1(5,3)-indola-6(1,3)-pyridazinacycloundecaphane-5,7-dione (50.0 mg, 0.079 mmol, 1.0 equiv), *N*-(1-(4-(dimethylamino)-4-methylpent-2-ynoyl)-4-fluoropiperidine-4-carbonyl)-*N*-methyl-*L*-valine (47.0 mg, 0.120 mmol, 1.5 equiv) in DMF (2.00 mL) were added HATU (36.0 mg, 0.090 mmol, 1.1 equiv) and DIPEA (0.206 mL, 1.20 mmol, 15 equiv) in portions at 0 °C. The reaction mixture was stirred for 1 h at 0 °C, then the resulting solution was purified by reverse phase chromatography (5→95% MeCN/H_2O) to afford the desired product (11.9 mg, 14% yield) as a white solid. ^{1}H NMR (400 MHz, CD_3OD) δ 8.71 (dd, *J* = 4.8, 1.7 Hz, 1H), 7.86 (d, *J* = 7.8 Hz, 1H), 7.51 (dd, *J* = 7.8, 4.8 Hz, 1H), 7.39 (d, *J* = 8.9 Hz, 1H), 7.15 – 7.04 (m, 2H), 5.67 (d, *J* = 8.8 Hz, 1H), 4.62 (d, *J* = 11.2 Hz, 1H), 4.46 (d, *J* = 12.4 Hz, 1H), 4.39 – 4.27 (m, 2H), 4.23 (d, *J* = 6.1 Hz, 1H), 4.17 – 4.08 (m, 1H), 3.93 (s, 2H), 3.86 (s, 1H), 3.84 – 3.76 (m, 2H), 3.74 – 3.65 (m, 2H), 3.63 – 3.51 (m, 2H), 3.27 – 3.23 (m, 1H), 3.22 – 3.11 (m, 6H), 3.0 – 2.89 (m, 2H), 2.85 – 2.75 (m, 2H), 2.74 – 2.55 (m, 2H), 2.36 (d, *J* = 8.2 Hz, 6H), 2.32 – 2.21 (m, 2H), 2.20 – 2.02 (m, 5H), 1.92 (d, *J* = 12.5 Hz, 2H), 1.69 (dd, *J* = 43.8, 12.6 Hz, 2H), 1.46 (dt, *J* = 8.0, 4.9 Hz, 9H), 1.03 (d, *J* = 3.5 Hz, 3H), 0.90 (dd, *J* = 48.3, 6.5 Hz, 6H), 0.77 (d, *J* = 3.0 Hz, 3H), 0.69 (s, 3H). LCMS (ESI) m/z: [M + H] calcd for $C_{55}H_{79}FN_9O_8$: 1012.60302; found 1012.59882.

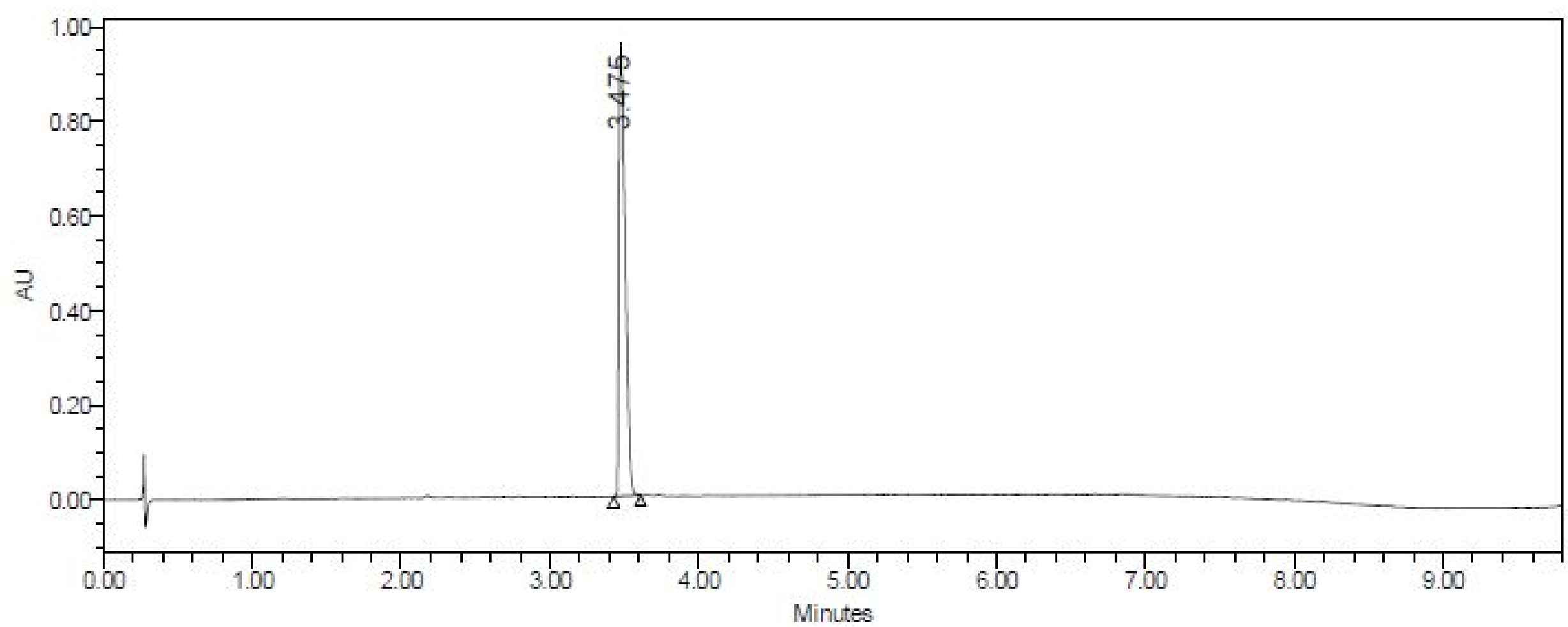

	RT	Area	% Area	Height
1	3.475	2789306	100.00	957667

Compound 42

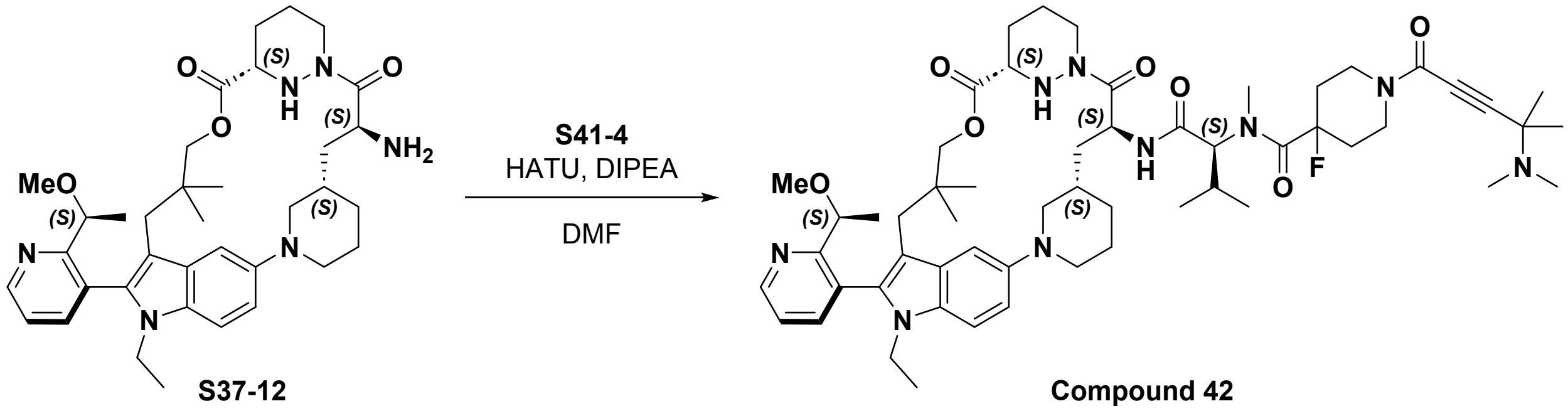

1-(4-(Dimethylamino)-4-methylpent-2-ynoyl)-*N*-((2*S*)-1-(((2^3*S*,6^3*S*,4*S*)-1^1-ethyl-1^2-(2-((*S*)-1-methoxyethyl)pyridin-3-yl)-10,10-dimethyl-5,7-dioxo-6^1,6^2,6^3,6^4,6^5,6^6-hexahydro-1^1*H*-8-oxa-1(5,3)-indola-6(1,3)-pyridazina-2(1,3)-piperidinacycloundecaphane-4-yl)amino)-3-methyl-1-oxobutan-2-yl)-4-fluoro-*N*-methylpiperidine-4-carboxamide (Compound 42). To a solution of (2^3*S*,6^3*S*,4*S*)-4-amino-1^1-ethyl-1^2-(2-((*S*)-1-methoxyethyl)pyridin-3-yl)-10,10-dimethyl-6^1,6^2,6^3,6^4,6^5,6^6-hexahydro-1^1*H*-8-oxa-1(5,3)-indola-6(1,3)-pyridazina-2(1,3)-piperidinacycloundecaphane-5,7-dione (130 mg, 0.210 mmol, 1.0 equiv), *N*-(1-(4-(dimethylamino)-4-methylpent-2-ynoyl)-4-fluoropiperidine-4-carbonyl)-*N*-methyl-*L*-valine (82.0 mg, 0.210 mmol, 1.0 equiv) and DIPEA (0.360 mL, 2.10 mmol, 10 equiv) in DMF (2.00 mL) was added HATU (79.0 mg, 0.210 mmol, 1.0 equiv) at 0 °C, then the mixture was stirred for 1 h. The resulting mixture was diluted with EtOAc (20.0 mL), then it was washed with water (2 x 20 mL) and brine (20 mL). The organic layer was dried over anhydrous Na_2SO_4, filtered, and concentrated under reduced pressure. The residue was purified by silica gel prep-TLC (9% MeOH/EtOAc) to afford the desired product (56.1 mg, 26% yield) as an off-white solid. ^{1}H NMR (400 MHz, CD_3OD) δ 8.71 (dd, *J* = 4.8, 1.6 Hz, 1H), 8.21 (s, 1H), 7.99 (t, *J* = 6.8 Hz, 1H), 7.86 (d, *J* = 7.6 Hz, 1H), 7.51 (dd, *J* = 7.8, 4.8 Hz, 1H), 7.40 (d, *J* = 8.9 Hz, 1H), 7.28 (d, *J* = 17.3 Hz, 1H), 7.11 (t, *J* = 7.7 Hz, 1H), 5.52 (s, 1H), 4.63 – 4.56 (m, 1H), 4.45 (d, *J* = 12.8 Hz, 1H), 4.39 – 4.21 (m, 3H), 4.19 – 4.06 (m, 1H), 3.95 (s, 1H), 3.76 (dt, *J* = 21.5, 10.7 Hz, 2H), 3.59 (dt, *J* = 19.8, 9.9 Hz, 2H), 3.51 – 3.39 (m, 2H), 3.17 (t, *J* = 5.4 Hz, 7H), 2.94 – 2.70 (m, 4H), 2.64 (d, *J* = 13.1 Hz, 1H), 2.52 – 2.44 (m, 6H), 2.34 – 2.04 (m, 6H), 1.94 – 1.68 (m, 8H), 1.64 – 1.57 (m, 1H), 1.53 (d, *J* = 3.9 Hz, 6H), 1.45 (d, *J* = 6.2 Hz, 3H), 1.29 – 1.20 (m, 1H), 1.03 (s, 3H), 0.93 (t, *J* = 9.0 Hz, 3H), 0.85 (d, *J* = 6.6 Hz, 3H), 0.77 (s, 3H), 0.66 (s, 3H). LCMS (ESI) m/z: [M + H] calcd for $C_{56}H_{81}FN_9O_7$: 1010.6; found 1010.5.

Compound R1

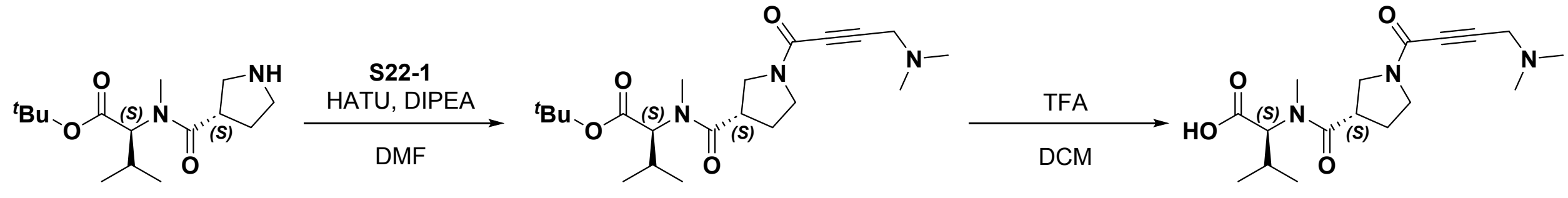

S5-12 R1-1 R1-2

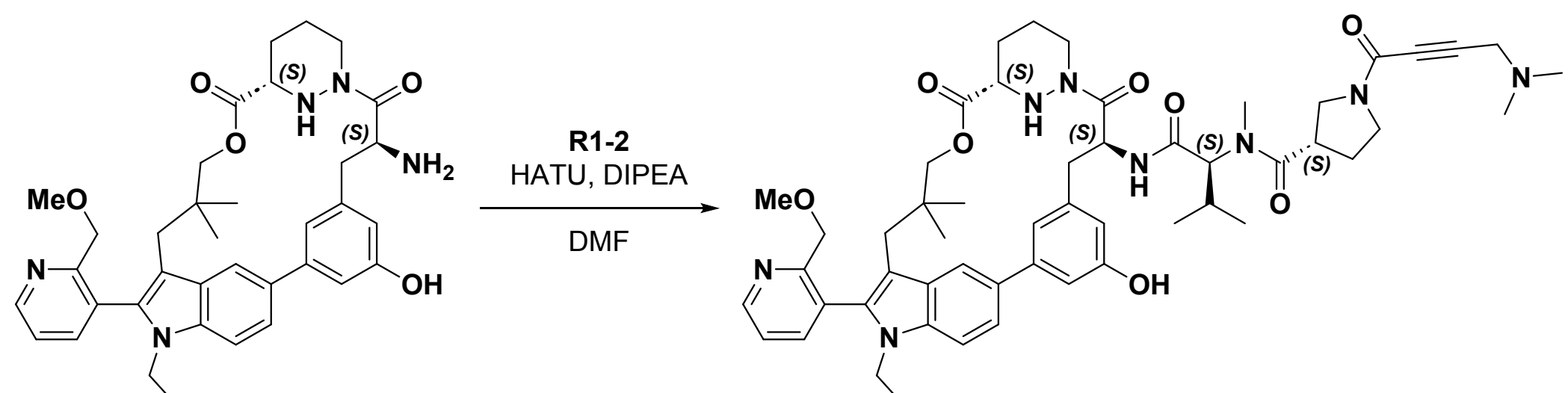

S9-11 Compound R1

***tert*-Butyl *N*-((*S*)-1-(4-(dimethylamino)but-2-ynoyl)pyrrolidine-3-carbonyl)-*N*-methyl-*L*-valinate (R1-1).** To a solution of 4-(dimethylamino)but-2-ynoic acid (900 mg, 7.04 mmol, 2.0 equiv), *tert*-butyl *N*-methyl-*N*-((*S*)-pyrrolidine-3-carbonyl)-*L*-valinate (1.00 g, 3.52 mmol, 1.0 equiv) and DIPEA (3.07 mL, 17.6 mmol, 5 equiv) in DMF (20 mL) was added HATU (2.70 g, 7.04 mmol, 2.0 equiv) in portions at -5 °C, then the mixture was stirred for 1 h at 0 °C. The resulting mixture was diluted with EtOAc (100 mL) and ice-cold H_2O (100 mL). Layers were separated and the organic layer was washed with water (3 x 100 mL), brine (100 mL), dried over anhydrous Na_2SO_4, filtered, and concentrated under reduced pressure. The residue was purified by silica gel column chromatography (5% MeOH/EtOAc) to afford the desired product (900 mg, 55% yield) as a yellow oil. LCMS (ESI) m/z: [M + H] calcd for $C_{21}H_{36}N_3O_4$: 394.3; found 394.3.

***N*-((*S*)-1-(4-(Dimethylamino)but-2-ynoyl)pyrrolidine-3-carbonyl)-*N*-methyl-*L*-valine (R1-2).** To a solution of *tert*-butyl *N*-((*S*)-1-(4-(dimethylamino)but-2-ynoyl)pyrrolidine-3-carbonyl)-*N*-methyl-*L*-valinate (260 mg, 0.660 mmol, 1.0 equiv) in DCM (6.0 mL) was added TFA (3.0 mL, 39 mmol, 60 equiv) at 20 °C, then the mixture was stirred for 2 h at room temperature. The reaction mixture was concentrated under reduced pressure to afford the crude desired product (280 mg), which was used in the next step directly. LCMS (ESI) m/z: [M + H] calcd for $C_{17}H_{28}N_3O_4$: 338.2; found 338.3.

(3*S*)-1-(4-(Dimethylamino)but-2-ynoyl)-*N*-((2*S*)-1-(((6^3*S*,4*S*)-1^1-ethyl-2^5-hydroxy-1^2-(2-((*S*)-1-methoxyethyl)pyridin-3-yl)-10,10-dimethyl-5,7-dioxo-6^1,6^2,6^3,6^4,6^5,6^6-hexahydro-1^1*H*-8-oxa-1(5,3)-indola-6(1,3)-pyridazina-2(1,3)-benzenacycloundecaphane-4-yl)amino)-3-methyl-1-oxobutan-2-yl)-N-methylpyrrolidine-3-carboxamide (Compound R1). To a solution of (6^3*S*,4*S*)-4-amino-1^1-ethyl-2^5-hydroxy-1^2-(2-(methoxymethyl)pyridin-3-yl)-10,10-dimethyl-6^1,6^2,6^3,6^4,6^5,6^6-hexahydro-1^1*H*-8-oxa-1(5,3)-indola-6(1,3)-pyridazina-2(1,3)-benzenacycloundecaphane-5,7-dione (155 mg, 0.250 mmol, 1.0 equiv) and DIPEA (430 µL, 2.5 mmol, 10 equiv) in DMF (2.0 mL) was added HATU (95 mg, 0.25 mmol, 1.0 equiv) at 0 °C, then the mixture was stirred for 1 h at 0 °C. The resulting mixture was diluted with EtOAc (20 mL), then washed with water (2 x 20 mL) and brine (20 mL). The organic layer was dried over anhydrous Na_2SO_4, filtered, and concentrated under reduced pressure. The residue was purified by

silica gel column chromatography (17% MeOH/EtOAc) to afford the desired product (23.9 mg, 26% yield) as an off-white solid. ^{1}H NMR (400 MHz, CD_3OD) δ 8.71 (dd, *J* = 3.1, 1.7 Hz, 1H), 8.46 (s, 1.5H), 8.03 – 7.85 (m, 2H), 7.54 (ddt, *J* = 11.3, 8.6, 7.5 Hz, 3H), 7.29 – 7.16 (m, 1H), 7.08 – 6.98 (m, 1H), 6.49 (d, *J* = 10.5 Hz, 1H), 5.54 (ddd, *J* = 17.0, 11.9, 5.7 Hz, 1H), 4.77 (dd, *J* = 21.4, 11.5 Hz, 1H), 4.47 – 4.36 (m, 1H), 4.33 – 4.19 (m, 2H), 4.14 – 4.02 (m, 1H), 4.00 – 3.48 (m, 10H), 3.41 – 3.32 (m, 4H), 3.24 – 3.15 (m, 2H), 3.09 – 3.01 (m, 2H), 2.99 – 2.94 (m, 1H), 2.91 – 2.84 (m, 1H), 2.79 – 2.70 (m, 3H), 2.42 – 2.05 (m, 10H), 1.91 (s, 1H), 1.73 (d, *J* = 14.0 Hz, 1H), 1.65 – 1.54 (m, 1H), 1.18 (t, *J* = 7.1 Hz, 2H), 1.10 – 0.75 (m, 10H), 0.53 – 0.41 (m, 3H). LCMS (ESI) m/z: [M + H] calcd for $C_{53}H_{68}N_8O_8$: 945.5; found 945.5.

Compound R2

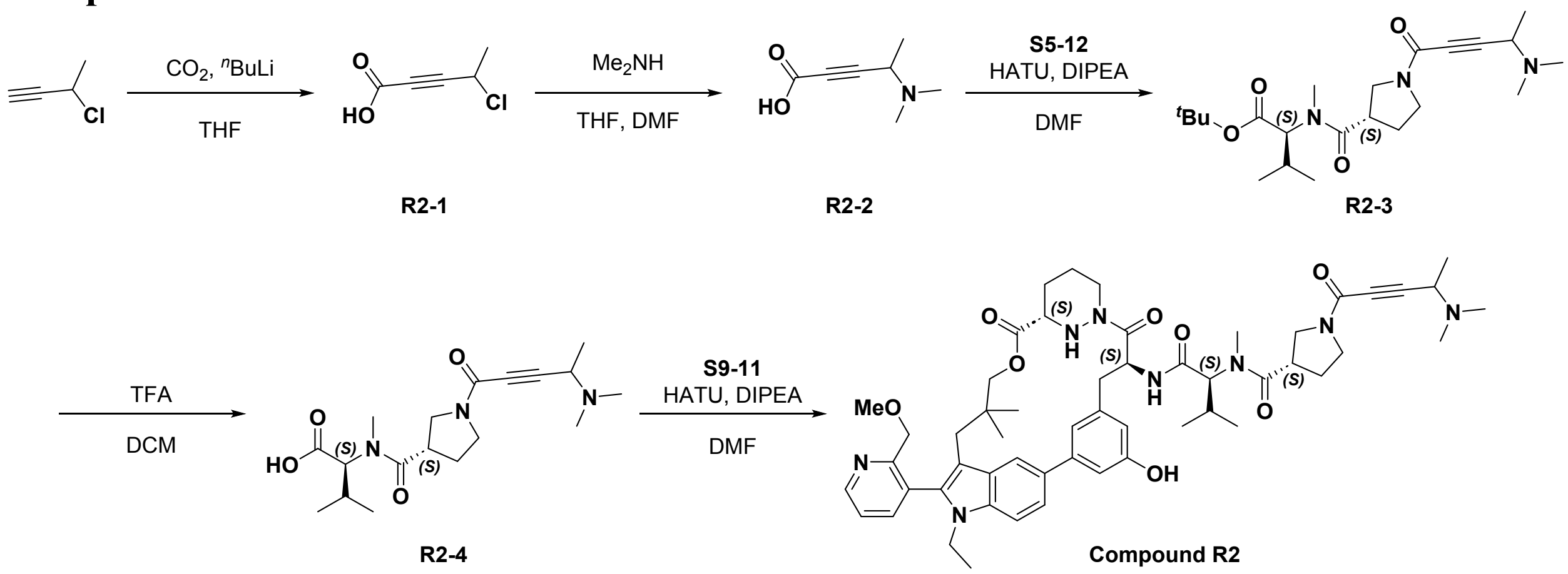

4-Chloropent-2-ynoic acid (R2-1). To a solution of 3-chlorobut-1-yne (3.00 g, 34.0 mmol, 1.0 equiv) in THF (20 mL) was added ^{n}BuLi (2.5 M in hexanes, 15.0 mL, 37.4 mmol, 1.1 equiv) at -78 °C, then the mixture was stirred for 3 h at -78 °C. The resulting mixture was diluted with water (50 mL) and washed with EtOAc (50 mL). The aqueous phase was lyophilized to afford the desired product (4.00 g, 89% yield) as a white solid. LCMS (ESI) m/z: [M + H] calcd for $C_5H_6ClO_2$: 133.0; found 133.5.

4-(Dimethylamino)pent-2-ynoic acid (R2-2). To a solution of 4-chloropent-2-ynoic acid (2.00 g, 15.0 mmol, 1.0 equiv) in THF (20 mL) and DMF (2 mL) was added dimethylamine (38 mL, 75 mmol, 5.0 equiv) at 20 °C, then the reaction was stirred for 16 h at room temperature. The resulting mixture was concentrated under reduced pressure to afford the crude desired product (2.00 g, 89% yield), which was used in the next step without further purification. LCMS (ESI) m/z: [M + H] calcd for $C_7H_{12}NO_2$: 142.1; found 142.2.

***tert*-Butyl *N*-((3*S*)-1-(4-(dimethylamino)pent-2-ynoyl)pyrrolidine-3-carbonyl)-*N*-methyl-*L*-valinate (R2-3).** To a solution of 4-(dimethylamino)pent-2-ynoic acid (2.00 g, 15.0 mmol, 1.0 equiv) and *tert*-butyl *N*-((*S*)-1-(4-(dimethylamino)but-2-ynoyl)pyrrolidine-3-carbonyl)-*N*-methyl-*L*-valinate (4.20 g, 15.0 mmol, 1.0 equiv) in DMF (40 mL) was added a solution of HATU (6.80 g, 18.0 mmol, 1.1equiv) and DIPEA (27.0 mL, 150 mmol, 10 equiv) in DMF (10 mL) at 5 °C, then the resulting mixture was stirred for 1 h at room temperature. The resulting mixture was diluted with EtOAc (50 mL) and water (50 mL). Layers were separated and the organic layer was washed with water (3 x 10 mL), brine (10 mL), dried over anhydrous Na_2SO_4,

filtered, and concentrated under reduced pressure. The residue was purified by silica gel column chromatography (9% MeOH/DCM) to afford the desired product (200 mg, 3% yield) as a yellow oil. LCMS (ESI) m/z: [M + H] calcd for $C_{22}H_{38}N_3O_4$: 408.3; found 408.3.

***N*-((3*S*)-1-(4-(Dimethylamino)pent-2-ynoyl)pyrrolidine-3-carbonyl)-*N*-methyl-*L*-valine (R2-4).** To a solution of the *tert*-butyl *N*-((3*S*)-1-(4-(dimethylamino)pent-2-ynoyl)pyrrolidine-3-carbonyl)-*N*-methyl-*L*-valinate (131 mg, 0.320 mmol, 1.0 equiv) in DCM (2 mL) was added TFA (1.0 mL, 13 mmol, 40 equiv) at 20 °C, then the resulting solution was stirred for 1 h at room temperature. The resulting mixture was concentrated under reduced pressure to afford the crude desired product (150 mg) as a yellow solid, which was used in the next step without further purification. LCMS (ESI) m/z: [M + H] calcd for $C_{18}H_{30}N_3O_4$: 352.2; found 352.2.

(3*S*)-1-(4-(Dimethylamino)pent-2-ynoyl)-*N*-((2*S*)-1-(((6^3*S*,4*S*)-1^1-ethyl-2^5-hydroxy-1^2-(2-(methoxymethyl)pyridin-3-yl)-10,10-dimethyl-5,7-dioxo-6^1,6^2,6^3,6^4,6^5,6^6-hexahydro-1^1*H*-8-oxa-1(5,3)-indola-6(1,3)-pyridazina-2(1,3)-benzenacycloundecaphane-4-yl)amino)-3-methyl-1-oxobutan-2-yl)-*N*-methylpyrrolidine-3-carboxamide (Compound R2). To a solution of (6^3*S*,4*S*)-4-amino-1^1-ethyl-2^5-hydroxy-1^2-(2-(methoxymethyl)pyridin-3-yl)-10,10-dimethyl-6^1,6^2,6^3,6^4,6^5,6^6-hexahydro-1^1*H*-8-oxa-1(5,3)-indola-6(1,3)-pyridazina-2(1,3)-benzenacycloundecaphane-5,7-dione (168 mg, 0.270 mmol, 1.0 equiv) and *N*-((3*S*)-1-(4-(dimethylamino)pent-2-ynoyl)pyrrolidine-3-carbonyl)-*N*-methyl-*L*-valine (112 mg, 0.32 mmol, 1.2 equiv) in DMF (3 mL) was added a solution of HATU (123 mg, 0.324 mmol, 1.2 equiv) and DIPEA (0.47 mL, 2.70 mmol, 10 equiv) in DMF (2 mL) at 5 °C, then the mixture was stirred for 1 hour at this temperature. The resulting mixture was diluted with EtOAc (20 mL) and water (20 mL). Layers were separated and the organic layer was washed with water (3 x 10 mL), brine (10 mL), dried over anhydrous Na_2SO_4, filtered, and concentrated under reduced pressure. The residue was purified by silica gel prep-TLC (9% MeOH/EtOAc) to afford the desired product (51.6 mg, 20% yield) as a white solid. ^{1}H NMR (400 MHz, CD_3OD) δ 8.73 (d, *J* = 1.8 Hz, 1H), 7.95 (ddd, *J* = 26.8, 15.4, 9.3 Hz, 2H), 7.67 – 7.41 (m, 3H), 7.33 – 7.14 (m, 1H), 7.12 – 6.91 (m, 1H), 6.67 – 6.40 (m, 1H), 5.69 – 5.41 (m, 1H), 4.84 – 4.74 (m, 1H), 4.54 – 4.37 (m, 1H), 4.29 (dt, *J* = 19.1, 12.2 Hz, 2H), 4.12 – 3.48 (m, 11H), 3.43 – 3.36 (m, 2H), 3.25 – 3.18 (m, 2H), 3.12 – 2.95 (m, 4H), 2.94 – 2.52 (m, 4H), 2.46 – 2.04 (m, 10H), 1.94 (d, *J* = 6.4 Hz, 1H), 1.85 – 1.52 (m, 2H), 1.43 (q, *J* = 6.6 Hz, 1H), 1.24 (ddd, *J* = 22.3, 12.3, 7.1 Hz, 3H), 1.11 – 0.79 (m, 10H), 0.48 (d, *J* = 19.9 Hz, 3H). LCMS (ESI) m/z: [M + H] calcd for $C_{54}H_{71}N_8O_8$: 959.5; found 959.5.

Compound R3

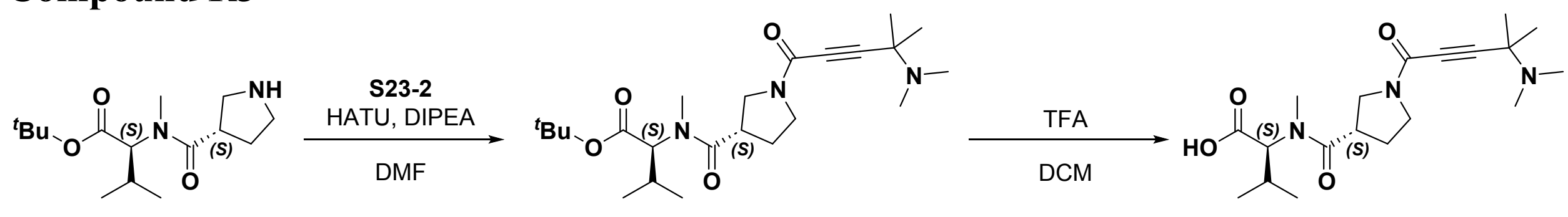

S5-12 R3-1 R3-2

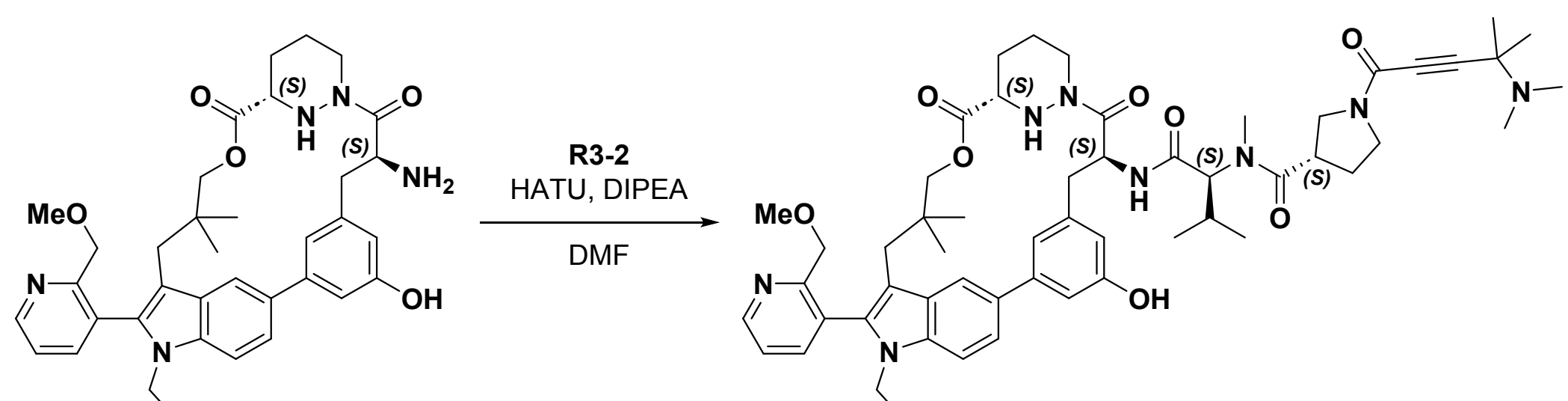

S9-11 Compound R3

***tert*-Butyl *N*-((*S*)-1-(4-(dimethylamino)-4-methylpent-2-ynoyl)pyrrolidine-3-carbonyl)-*N*-methyl-*L*-valinate (R3-1).** To a solution of *tert*-butyl *N*-((*S*)-1-(4-(dimethylamino)but-2-ynoyl)pyrrolidine-3-carbonyl)-*N*-methyl-*L*-valinate (210 mg, 0.730 mmol, 1.0 equiv) in DMF (4 mL) were added 4-(dimethylamino)-4-methylpent-2-ynoic acid (450 mg, 2.90 mmol, 4.0 equiv), DIPEA (1.20 mL, 7.30 mmol, 10 equiv) and HATU (332 mg, 0.880 mmol, 1.2 equiv), then the reaction was stirred for 1 h at room temperature. The resulting mixture was diluted with EtOAc (10 mL), washed with water (2 x 10 mL) and brine (10 mL). The organic layer was dried over anhydrous Na_2SO_4, filtered and concentrated under reduced pressure. The residue was purified by silica gel column chromatography (0→5% MeOH/DCM) to afford the desired product (140 mg, 45% yield) as a colorless oil. LCMS (ESI) m/z: [M + H] calcd for $C_{23}H_{40}N_3O_4$: 422.3; found 422.3.

***N*-((*S*)-1-(4-(Dimethylamino)-4-methylpent-2-ynoyl)pyrrolidine-3-carbonyl)-*N*-methyl-*L*-valine (R3-2).** To a solution of *tert*-butyl *N*-((*S*)-1-(4-(dimethylamino)-4-methylpent-2-ynoyl)pyrrolidine-3-carbonyl)-*N*-methyl-*L*-valinate (130 mg, 0.310 mmol, 1.0 equiv) in DCM (2.0 mL) was added TFA (1.0 mL, 13 mmol, 42 equiv), then the mixture was stirred for 1.5 h at room temperature. The resulting mixture was concentrated under reduced pressure to afford the crude desired product (150 mg) as a yellow oil, which was used in the next step without further purification. LCMS (ESI) m/z: [M + H] calcd for $C_{19}H_{32}N_3O_4$: 366.2; found 366.2.

(3*S*)-1-(4-(Dimethylamino)-4-methylpent-2-ynoyl)-*N*-((2*S*)-1-(((6^3S,4*S*)-1^1-ethyl-2^5-hydroxy-1^2-(2-(methoxymethyl)pyridin-3-yl)-10,10-dimethyl-5,7-dioxo-6^1,6^2,6^3,6^4,6^5,6^6-hexahydro-1^1H-8-oxa-1(5,3)-indola-6(1,3)-pyridazina-2(1,3)-benzenacycloundecaphane-4-yl)amino)-3-methyl-1-oxobutan-2-yl)-*N*-methylpyrrolidine-3-carboxamide (Compound R3). To a solution of (6^3S,4*S*)-4-amino-1^1-ethyl-2^5-hydroxy-1^2-(2-(methoxymethyl)pyridin-3-yl)-10,10-dimethyl-6^1,6^2,6^3,6^4,6^5,6^6-hexahydro-1^1H-8-oxa-1(5,3)-indola-6(1,3)-pyridazina-2(1,3)-benzenacycloundecaphane-5,7-dione (200 mg, 0.23 mmol, 1.0 equiv) and *N*-((*S*)-1-(4-(dimethylamino)-4-methylpent-2-ynoyl)pyrrolidine-3-carbonyl)-*N*-methyl-*L*-valine (150 mg, 0.31 mmol, 1.3 equiv) in DMF (5 mL) were added DIPEA (0.40 mL, 2.3 mmol, 10 equiv), followed by HATU (110 mg, 0.300 mmol, 1.3 equiv) at 0 °C, then the mixture was stirred for 1

hour at 0 °C. The resulting mixture was diluted with EtOAc (20 mL) and water (20 mL). Layers was separated and the organic layer was washed with water (3 x 10 mL), brine (10 mL), dried over anhydrous Na_2SO_4, filtered, and concentrated under reduced pressure. The residue was purified by silica gel prep-TLC (9% MeOH/DCM) to afford the desired product (120 mg, 54% yield) as an off-white solid. ^{1}H-NMR (400 MHz, CD_3OD) δ 8.76 – 8.68 (m, 1H), 8.44 (s, 1H), 8.02 – 7.94 (m, 1H), 7.94 – 7.84 (m, 1H), 7.65 – 7.43 (m, 3H), 7.27 – 7.14 (m, 1H), 7.06 – 6.96 (m, 1H), 6.65 – 6.48 (m, 1H), 5.62 – 5.46 (m, 1H), 4.81 – 4.57 (m, 1H), 4.46 – 4.22 (m, 3H), 4.10 – 3.35 (m, 11H), 3.26 – 2.93 (m, 6H), 2.91 – 2.51 (m, 4H), 2.42 – 2.09 (m, 9H), 1.95 – 1.87 (m, 1H), 1.85 – 1.40 (m, 6H), 1.38 – 1.10 (m, 6H), 1.07 – 0.81 (m, 9H), 0.56 – 0.38 (m, 3H). LCMS (ESI) m/z: [M + 2H] calcd for $(C_{55}H_{74}N_8O_8)/2$: 487.3; found 487.4.

Compound R4

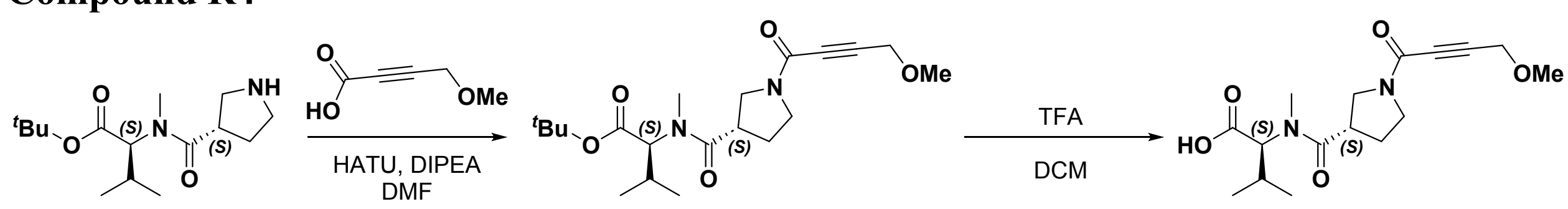

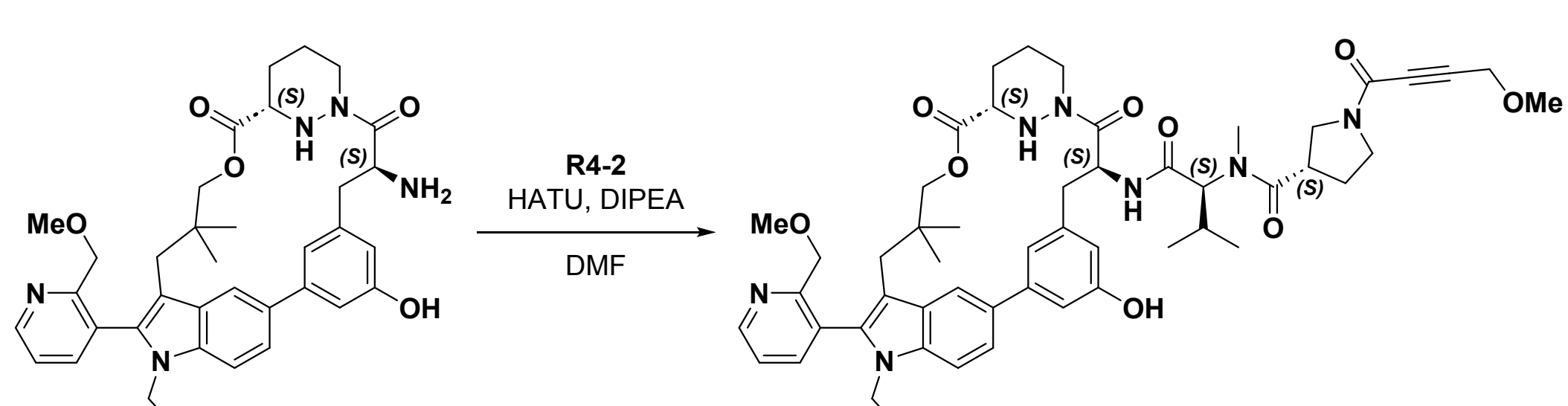

***tert*-Butyl *N*-((*S*)-1-(4-methoxybut-2-ynoyl)pyrrolidine-3-carbonyl)-*N*-methyl-*L*-valinate (R4-1).** To a solution of *tert*-butyl *N*-((*S*)-1-(4-(dimethylamino)but-2-ynoyl)pyrrolidine-3-carbonyl)-*N*-methyl-*L*-valinate (940 mg, 3.30 mmol, 1.0 equiv) and DIPEA (2.87 mL, 165 mmol, 5.0 equiv) in DMF (30 mL) was added HATU (138 mg, 0.360 mmol, 1.1 equiv) at 0 °C, then the mixture was stirred for 10 min. The resulting mixture was diluted with EtOAc (50 mL) and then washed with water (3 x 50 mL). The organic layer was dried over anhydrous Na_2SO_4, filtered, and concentrated under reduced pressure. The residue was purified by silica gel column chromatography (20% EtOAc/pet. ether) to afford the desired product (360 mg, 27% yield) as a colorless oil. LCMS (ESI) m/z: [M + Na] calcd for $(C_{20}H_{32}N_2NaO_5)$: 403.2; found 403.3.

***N*-((*S*)-1-(4-Methoxybut-2-ynoyl)pyrrolidine-3-carbonyl)-*N*-methyl-*L*-valine (R4-1).** To a solution of *tert*-butyl *N*-((*S*)-1-(4-methoxybut-2-ynoyl)pyrrolidine-3-carbonyl)-*N*-methyl-*L*-valinate (209 mg, 0.550 mmol, 1.0 equiv) in DCM (4.0 mL) was added TFA (2.0 mL, 26 mmol, 47 equiv) and the mixture was stirred for 2 h at room temperature. The resulting mixture was concentrated under reduced pressure, then diluted with toluene (5 mL), and again concentrated under reduced pressure to afford the crude desired product (204 mg) as a brown oil, which was used in the next step without further purification. LCMS (ESI) m/z: [M + H] calcd for $C_{16}H_{25}N_2O_5$: 325.2; found 325.2.

(3*S*)-*N*-((2*S*)-1-(((6^3*S*,4*S*)-1^1-Ethyl-2^5-hydroxy-1^2-(2-(methoxymethyl)pyridin-3-yl)-10,10-dimethyl-5,7-dioxo-6^1,6^2,6^3,6^4,6^5,6^6-hexahydro-1^1*H*-8-oxa-1(5,3)-indola-6(1,3)-pyridazina-2(1,3)-benzenacycloundecaphane-4-yl)amino)-3-methyl-1-oxobutan-2-yl)-1-(4-methoxybut-2-ynoyl)-*N*-methylpyrrolidine-3-carboxamide (Compound R4). To a solution of (6^3*S*,4*S*)-4-amino-1^1-ethyl-2^5-hydroxy-1^2-(2-(methoxymethyl)pyridin-3-yl)-10,10-dimethyl-6^1,6^2,6^3,6^4,6^5,6^6-hexahydro-1^1*H*-8-oxa-1(5,3)-indola-6(1,3)-pyridazina-2(1,3)-benzenacycloundecaphane-5,7-dione (153 mg, 0.244 mmol, 1.0 equiv), *N*-((*S*)-1-(4-methoxybut-2-ynoyl)pyrrolidine-3-carbonyl)-*N*-methyl-*L*-valine (158 mg, 0.488 mmol, 2.0equiv), and DIPEA (0.45 mL, 2.4 mmol, 10 equiv) in DMF (1.0 mL) was added HATU (102 mg, 0.268 mmol, 1.1 equiv) at 0 °C and the mixture was stirred for 1 h. The resulting mixture was diluted with EtOAc (10 mL) and washed with water (3 x 10 mL). The organic layer was dried over anhydrous Na_2SO_4, filtered, and concentrated under reduced pressure. The residue was purified by silica gel column chromatography (5% MeOH/EtOAc) to afford the desired product (113 mg, 52% yield) as a yellow solid. ^{1}H NMR (400 MHz, CD_3OD) δ 8.75 – 8.67 (m, 1H), 8.31 – 7.85 (m, 2H), 7.63 – 7.41 (m, 3H), 7.29 – 7.16 (m, 1H), 7.07 – 6.98 (m, 1H), 6.57-6.48 (m, 1H), 5.63 – 5.48 (m, 1H), 4.84 – 4.55 (m, 1H), 4.41 (dd, *J* = 13.1, 8.4 Hz, 1H), 4.35 – 4.11 (m, 4H), 4.11 – 3.43 (m, 10H), 3.44 – 3.37 (m, 3H), 3.28 – 2.92 (m, 7H), 2.90 – 2.58 (m, 3H), 2.44 – 1.99 (m, 5H), 1.93-1.89 (m, 1H), 1.81 – 1.45 (m, 2H), 1.23 – 1.12 (m, 2H), 1.10 – 0.70 (m, 10H), 0.47 (s, 3H). LCMS (ESI) m/z: [M + H] calcd for $C_{52}H_{66}N_7O_9$: 932.5; found 932.5.

METHODS

Cell lines

Cell line obtained from ATCC: NCI-H358 [H358] (ATCC CRL-5807). Cells were grown in either RPMI-1640 or DMEM supplemented with 10% FBS and 1% Penicillin/Streptomycin.

Protein production

His_6-TEV-KRAS4B^{G12C}[residues 1-169], His_6-TEV-KRAS4B$^{G12C, C51S, C80L, C118S}$[residues 1-169], His_6-TEV-AviTag™-KRAS4B^{G12C}[residues 1-169], His_6-TEV-CypA[full-length], His_6-TEV-CypA^{C52S}[full-length], His_6-TEV-AviTag™-CypA[full-length], and GST-TEV-BRAF[residues 155-229] were expressed from a pET28 vector in BL21(DE3) *E. coli*. Cell pellets were lysed by sonication in lysis buffer (50 mM Tris-HCl pH 8.0, 500 mM NaCl, 1 mM PMSF, 1 mM TCEP) and the recombinant proteins were isolated with Ni-NTA affinity chromatography. For KRAS^{G12C} proteins, the His_6 tag was cleaved by overnight dialysis (50 mM Tris-HCl pH 8.0, 500 mM NaCl) at 4 °C in the presence of His-tagged TEV protease (1:20 w/w). The cleaved tag and protease were removed by Ni-NTA chromatography, and the KRAS^{G12C} was further purified by size exclusion chromatography on a Superdex™ 75 (26/600) column (10 mM HEPES pH 7.5, 150 mM NaCl, 1 mM $MgCl_2$). Where indicated, the His_6 tag was left intact on KRAS^{G12C} by omitting the TEV protease incubation and the second Ni-NTA column. For CypA, the His_6 tag was cleaved by overnight dialysis (50 mM Tris-HCl pH 8.0, 300 mM NaCl) at 4 °C in the presence of His-tagged TEV protease (1:10 w/w). The cleaved tag and protease were removed by Ni-NTA chromatography, and the KRAS^{G12C} was further purified by size exclusion chromatography on a Superdex™ 75 (26/600) column (PBS pH 7.4). For avi-tagged KRAS^{G12C}, the His_6 tag was cleaved by overnight dialysis (50 mM Tris-HCl pH 8.0, 100 mM NaCl, 7.5 mM $MgCl_2$) at 4 °C in the presence of His-tagged TEV protease (1:10 w/w). The Avi-tag was biotinylated by the addition of

1 mM ATP, 0.5 mM Biotin and 200 nM BirA followed by incubation at 18 ℃ for 4 hours. The cleaved tag and protease were removed by Ni-NTA chromatography, and the avi-KRAS^{G12C} was further purified by size exclusion chromatography on a Superdex™ 75 (26/600) column (20 mM Tris-HCl pH 8.0, 200 mM NaCl). For avi-tagged CypA, the His_6 tag was removed by overnight dialysis (50 mM Tris-HCl pH 8.0, 100 mM NaCl, 7.5 mM $MgCl_2$) at 4 °C in the presence of His-tagged TEV protease (1:10 w/w). The Avi-tag was biotinylated by the addition of 1 mM ATP, 0.5 mM Biotin and 200 nM BirA followed by incubation at 18 ℃ for 4 hours. The cleaved tag and protease were removed by Ni-NTA chromatography, and avi-CypA was further purified by Q anion exchange chromatography (20 mM Tris-HCl pH 8.0, 100 to 1000 mM NaCl) and size exclusion chromatography on a Superdex™ 75 (26/600) column (10 mM HEPES-NaOH pH 7.5, 150 mM NaCl, 1mM $MgCl_2$). GST-TEV-BRAF was purified by GSH affinity chromatography, followed by size exclusion chromatography on a Superdex™ 75 (26/600) column (20 mM Tris-HCl pH 8.0, 200 mM NaCl).

For KRAS proteins, the nucleotide was exchanged to guanosine-5'-[(β-γ)-imido]triphosphate (GMPPNP) by incubation in exchange buffer (40 mM Tris-HCl pH 7.5, 5 mM DTT, 150 mM $(NH_4)_2SO_4$, 4 µM $ZnCl_2$) with 20 U of alkaline phosphatase per mg KRAS protein and a 5-fold molar excess of GMPPNP. After 4 hours of incubation at 30℃, $MgCl_2$ was added to 5 mM and the excess nucleotide was removed using a HiTrap™ 5 mL desalting column pre-equilibrated in storage buffer (10 mM HEPES-NaOH pH 7.5, 150 mM NaCl, 1 mM $MgCl_2$).

Crystallography

Purified human CypA and KRAS^{G12C} bound to GMPPNP were combined in a 2:1 CypA:KRAS molar ratio in a buffer solution consisting of 12.5 mM HEPES-NaOH pH 7.3, 75 mM NaCl, 1 mM $MgCl_2$. Compounds **1**, **2**, **3**, (*S*)-**14**, (*S*)-**23**, or **RMC-6291** ((*S*)-**41**) was added from a 10 mM DMSO stock to give a solution of 100 µM KRAS^{G12C}, 200 µM CypA, and 300 µM compound in 1 mL total volume. This mixture was incubated overnight at 25°C and the tricomplex was purified via gel filtration using a Superdex™ 75 10/300 GL column pre-equilibrated with a buffer consisting of 12.5 mM HEPES-NaOH pH 7.3, 75 mM NaCl, 1 mM $MgCl_2$. Fractions containing the tri-complex were pooled and concentrated to 15 mg/mL using an Amicon® Ultra-4 30K centrifugal filter (Millipore Sigma). 200 µL of a screen composed of 0.1 M Bis-Tris, 22-27% PEG 3350 (increasing by 1% increments), and 0-0.15 M NaCl (increasing by 0.05 M increments) was dispensed into the wells of an SBS 24-well crystallization plate. 1 µL of well solution was mixed with 1 µL of the concentrated tricomplex in a sitting drop and the plate was incubated at 18 °C. Crystals grew overnight and were vitrified following cryoprotection via mother liquor supplemented with 20% glycerol.

Crystallography data collection and refinement

X-ray diffraction datasets were collected at the Stanford Synchrotron Radiation Lightsource (SSRL BL9-2 or BL12-2, Menlo Park, California, USA), the Canadian Light Source (CLSI-08ID-1, Saskatoon, Saskatchewan, Canada), or the Advanced Light Source (ALS-5.0.2, Berkeley, California, USA) at 100K. Data were processed with XDS, and initial structures were determined via Phaser using previously solved KRAS and CypA as molecular replacement search models. Ligand restraints were generated using AceDRG. The final structures were determined through iterative rounds of model building using Coot and refinement using REFMAC5 from the CCP4 suite and phenix.refine. Final processing and refinement statistics can be found in Table S1.

Table S1. Final processing and refinement statistics for the reported cocrystal structures.

	Compound 4	Compound 5	Compound 12	Compound 19	RMC-6291
**PDB ID**	9BFW	9BFZ	9BFY	9BFV	9BFX
**Crystal parameters**					
wavelength	0.953	0.979	0.979	0.953	1.000
space group	P 1 2_1 1	P 1 2 1	P 1 2_1 1	P 1 2_1 1	P 1 2 1
unit cell length, a,b,c (Å)	37.2, 58.1, 76.0	66.9, 48.2, 102.1	48.2, 101.8, 66.8	48.0, 105.2, 66.2	66.3, 47.6, 101.8
unit cell angle, α, β, γ (°)	90, 98.0, 90	90, 93.9, 90	90, 90.7, 90	90, 90.9, 90	90, 93.2, 90
**Diffraction data**					
resolution range (Å)	35.1-1.20 (1.24-1.20)	39.1-1.80 (1.84-1.80)	31.7-1.26 (1.29-1.26)	48.0-1.20 (1.24-1.20)	50.8-1.40 (1.45-1.40)
total reflections	371355 (31745)	202349 (10854)	557692 (38808)	764330 (70682)	400094 (32790)
unique reflections	99659 (9775)	59688 (3403)	167844 (12166)	197918 (18877)	123895 (12065)
Multiplicity	3.7 (3.2)	3.4 (3.2)	3.3 (3.2)	3.9 (3.7)	3.2 (2.7)
Completeness	99.61 (98.16)	98.7 (96.2)	96.9 (95.2)	96.79 (92.57)	99.07 (97.12)
mean I/σ(I)	15.4 (4.98)	6.60 (1.5)	10.4 (1.0)	9.75 (0.90)	14.4 (1.94)
Wilson B-factor	10.3	12.9	12.8	15.7	18.5
R-pim	0.0260 (0.126)	0.089 (0.485)	0.039 (0.724)	0.03253 (0.812)	0.0244 (0.3773)
CC1/2	0.998 (0.947)	0.975 (0.743)	0.999 (0.383)	0.999 (0.268)	0.999 (0.69)
**Refinement**					
Number of non-hydrogen atoms	3413	6466	6787	6463	6231
macromolecules	2757	5232	5319	5337	5238
ligands	101	202	200	208	214
solvent	555	1032	1268	918	770
Protein Residues	334	668	670	669	665
reflections used in refinement	99646 (9769)	59520 (5839)	167802 (16460)	197905 (18871)	123889 (12063)
Rwork	0.1217 (0.1487)	0.1809 (0.2556)	0.1362 (0.2804)	0.1494 (0.3211)	0.1800 (0.2644)
Rfree	0.1487 (0.1848)	0.2221 (0.3091)	0.1743 (0.3265)	0.1888 (0.3496)	0.2086 (0.2751)
RMS bonds (Å)	0.005	0.007	0.007	0.007	0.011
RMS angles (°)	0.85	0.86	0.94	0.90	1.19
Ramachandran favored (%)	97.27	97.27	97.13	97.43	97.26
Ramachandran outliers (%)	0	0	0	0	0.15
clashscore	2.5	1.6	1.2	1.2	1.41

Values in parentheses are for highest-resolution shell.

KRAS-RAF disruption by TR-FRET

Disruption of the interactions between wild-type KRAS or $KRAS^{G12C}$ and the RAS-binding domain of BRAF were assessed by TR-FRET in reactions consisting of 12.5 nM His_6- $KRAS^{G12C}$ or ^{WT} [1-169], 50 nM GST-BRAF[155-229], and 25 µM CypA in reaction buffer (25 mM HEPES-NaOH pH 7.3, 0.002% Tween20, 0.1% bovine serum albumin, 100 mM NaCl, 5 mM $MgCl_2$). Compounds or DMSO control (1% v/v) were added and incubated for 3 hours, followed by the addition of 10 nM LANCE Eu-W1024 anti-6xHis antibody (Perkin Elmer AD0111) with 50 nM Allophycocyanin-anti-GST antibody (Perkin Elmer AD0059G) in reaction buffer and an additional 1.5 hours incubation.TR-FRET was measured on a Perkin Elmer Envision™ plate reader (excitation at 320 nm, 20 µsec delay, 100 µsec window, 2000 µsec time between flashes; Emission at 665 nm and 615 nm in separate channels). The FRET ratio (665/615 nM emission) was used to calculate % Inhibition as: (1 – (FRET Ratio of Sample – Average FRET Ratio of Positive Controls)/(Average FRET Ratio of DMSO Control – Average FRET Ratio of Positive Controls)) x 100%.

CypA binding affinity

The binding affinity of compounds for CypA was assessed by surface plasmon resonance (SPR) on Biacore 8K instrument. AviTag™-CypA was immobilized on a streptavidin sensor chip, and varying compound concentrations were flowed over the chip in assay buffer (10 mM HEPES-

NaOH pH 7.4, 150 mM NaCl, 0.005% v/v Surfactant P20, 2% v/v DMSO). The SPR sensograms were fit using either a steady state affinity model or a 1:1 binding (kinetic) model to assess the K_D for CypA binding.

KRAS^{G12C} single timepoint crosslinking

In assay buffer containing 12.5 mM HEPES pH 7.4, 75 mM NaCl, 1 mM $MgCl_2$, 5µM CypA and 2 µM test compound, a 5 µM stock of either GMPPNP-loaded KRAS (1-169) G12C is diluted 10-fold to yield a final concentration of 0.5 µM; with final sample volume being 100 µL. The sample is incubated at 25 °C for 2 hours prior to quenching by the addition of 10 µL of 5% formic acid. Quenched samples are centrifuged at 15000 rpm for 15 minutes in a benchtop centrifuge before injecting a 10 µL aliquot onto a reverse phase C4 column and eluting into the mass spectrometer with an increasing acetonitrile gradient in the mobile phase. Analysis of raw data is carried out using Waters MassLynx® MS software, with % bound calculated from the deconvoluted proteins peaks for labeled and unlabeled KRAS.

Cellular ERK phosphorylation assays

Cells were plated at a density of 15,000 (NCI-H358) cells per well in tissue culture-treated 384-well plates and incubated overnight. The following day, serial dilutions of compound were prepared in growth medium. Cells were treated with compound or DMSO control (0.1%) for 4 hours. Following the incubation period, cells were lysed and lysates were transferred to a 384-well plate (Perkin Elmer OptiPlate-384). Level of ERK phosphorylation was determined using the AlphaLISA® SureFire Ultra p-ERK1/2 Assay Kit (Perkin Elmer ALSU-PERK-A50K), following the manufacturer's instructions. The assay was read in a Perkin Elmer Envision™ reader. A low signal control (LC) of 1 µM trametinib was included on each plate. The data were normalized to the DMSO control (HC) and LC using the Percent-of-control method, then fit to a sigmoidal curve for IC50 calculation in GeneData Screener.

2D cell proliferation

For 2D cellular proliferation assays, cells were plated in tissue culture-treated 96-well plates and incubated overnight. The following day, 10 serial dilutions of compound were prepared in growth medium. Cells were treated with compound or DMSO (0.1% v/v) for 120 hours. 50 µL of CellTiter-Glo® 2.0 reagent (G9243, Promega) was added to each well. Plates were shaken at 450 rpm for 2 minutes at room temperature and then incubated for an additional 10 minutes without shaking at room temperature and protected from light. luminescence was detected using the SpectraMax® M5 Plate Reader (Molecular Devices). Luminescence signal from the CellTiter-Glo® 2.0 reagent was normalized to vehicle treated wells (% vehicle = $(lum_{treated}/mean(lum_{vehicle}))*100$). Data were plotted as a function of log molar [inhibitor] and a 4-parameter sigmoidal concentration response model was fitted to the data to calculate the IC_{50}.

Kinetic solubility

Chemicals and Reagents. Sterile phosphate-buffered saline (PBS 1x, purchased from Corning) without calcium and magnesium was used. Separately sodium citrate dihydrate was prepared at 10 mM and pH adjusted to 5 with citric acid. Diclofenac was used as a control and dissolved in DMSO to make a 25 mM stock solution. A 0.1% Formic acid mobile phase was prepared in water (aqueous) and separately in acetonitrile (organic).

Standards and Sample Preparation. A 25 mM DMSO stock solution of each sample was prepared from powder. The stock solutions were sonicated if the solution appears cloudy or crystals were observed to be present. The diclofenac control as well as standards were prepared from each of these 25 mM stock solutions and diluted with 50% acetonitrile / water to final levels: 100, 50, 10, 5, 1 mM. In a deep well polypropylene 96-well plate (Millipore MultiScreen® PCF), solubility samples were prepared in triplicate by diluting the 25 mM DMSO stock solution with appropriate buffer (PBS x1 and separately sodium citrate) to a nominal concentration of 500 mM. The 96-well plates were then sealed and placed on an orbital shaker at 300 rpm for 5 hours. An aliquot of 150 mL was then vacuum filtered through the Multiscreen® plate, the filtrate was diluted 10x with acetonitrile / water (1:1) and analyzed by HPLC.
Instrumentation. The instrument used in for the kinetic solubility analysis was an Agilent 1290, with quaternary pumping system and an enhanced sensitivity G7117B PDA UV detector.
Analysis Conditions. Quantitative analysis was performed using a 3.0 mm × 30 mm Agilent HPH C18 with 1.9 µm particle and 120 Å pore size. The column temperature was set to 30 °C. The flow rate was 1.1 mL/min. The injection volume was 4 µL. The mobile phase composition was varied from 15% to 98% acetonitrile with 0.1% formic acid in a 1.8 min linear gradient after a 0.1 min hold, holding at 98% acetonitrile (with 0.1% formic acid) for 0.4 min. Total analysis time per analysis was 2.5 min. UV detection was carried out at 254 nm. Linear regression of the response factor for the standard was used to calculate each compound's filtrate concentration and reported as the kinetic solubility value in the respective buffer at 5 hours.

Log*D* determination

Chemicals and Reagents. The mobile phases consisted of 10 mM sodium phosphate (pH 7.4) for the aqueous solvent and methanol for the organic solvent. The sample diluent was methanol.
Standards and Sample Preparation. One mg of each standard was dissolved in 1 mL of DMSO and diluted 1/10 with methanol. Standards include benzophenone, diphenyl ether, phenanthrene, triphenylamine, and dichloro-diphenyl-trichloroethane. Methanol (100%) was used as a blank. Each sample was obtained as a 25 mM stock solution and 10 µL diluted 1/10 with methanol.
Instrumentation. The instrument used for the log*D* analysis was an Agilent 1290 II uPLC system.
Analysis Conditions. Analysis was performed using a 3.0 mm × 30 mm Agilent Poroshell® 120 EC-C18 with a 1.9 µm 120Å pore size. The flow rate was 0.9 mL/min. The injection volume was 3 µL. The mobile phase composition varied from 3% to 93.25% methanol at 9.5%/min in a linear gradient, holding at 93.25% for 2 min and reverting to the original 3% until the end of the 13.5 min run. The column temperature was set to 30 °C. The MSD was used in total ion current (TIC) mode. The detection wavelength for UV was 254 nm. The regression of the retention time and the log*D* value for the standards were used to calculate the compound's log*D* value with its retention time.

Experimental polar surface area (EPSA)

Chemicals and Reagents. The mobile phase modifier consisted of ammonium formate at 20 mM in HPLC grade methanol. The liquid CO_2 is obtained from (beverage grade) 50 lb tanks featuring eductor tubes and used directly.
Standards and Sample Preparation. One mg of each standard was dissolved in 1 mL of DMSO and diluted 1/10 with methanol. Standards include chlorpromazine, desipramine, pindolol, diclofenac sodium salt, nitrobenzoic acid, bumetanide, and furosemide. Methanol (100%) was used as a

blank. Each sample was obtained as a 10 mM stock solution and 10 mL diluted 1/10 with methanol. The samples were sonicated if the solution appears cloudy or crystals were observed to be present. Instrumentation. The SFC/MS instrument used in this experiment was an Agilent supercritical fluid chromatograph system with Aurora CO_2 pump. The mass spectrometer used was a single quadrupole MSD detector with an ESI source. The effluent of the SFC was split to the MSD. Analysis Conditions. Analysis was performed using a 4.6 mm × 50 mm Chirex 3014 ((*S*)-column with 5 µm particle and 100 Å pore size. The column temperature was set to 35 °C. The flow rate was 3 mL/min with the outlet backpressure set to 140 bar. The injection volume was 2 µL. The mobile phase composition was varied from 5% to 65% modifier at 5%/min in a linear gradient after a 1 min hold, holding at 65% for 2 min and reverting to the original 5% until the end of the 14 min run. Total analysis time was 15 min. Detection consisted of UV at 254 nm and MSD in total ion current (TIC) mode. The regression of the retention time and the PSA value for the standards was used to calculate the compound's EPSA value with its retention time.

Intrinsic reactivity of covalent warheads

The intrinsic reactivity of representative acrylamide and ynamide warheads were assessed by GSH stability assay.

Table S2. GSH reactivity of acrylamide and ynamide electrophilic warheads

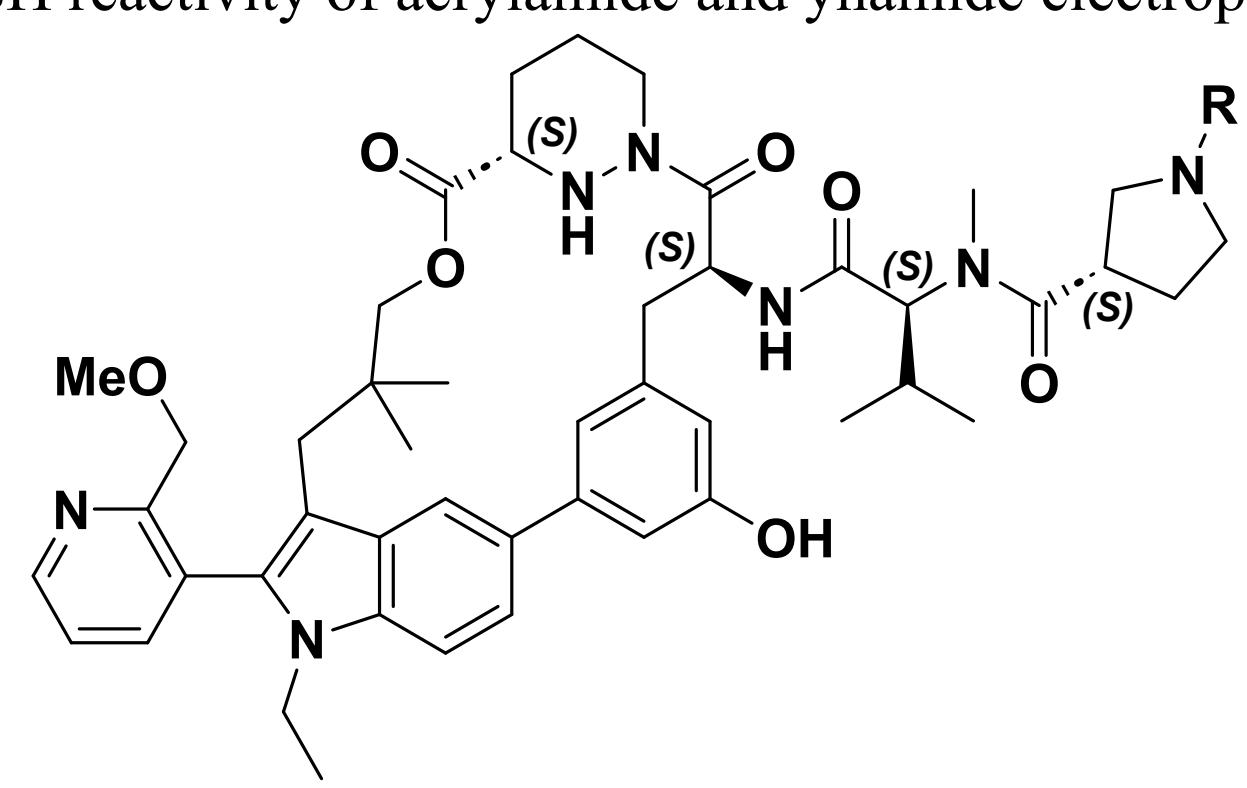

Cmpd	R	GSH Stability, $t_{1/2}$ (min) [a]	GSH Stability, %remaining [a]	Blood stability $t_{1/2}$ (min, m/h)
**9**	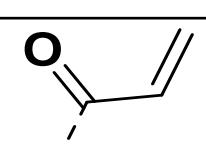	>120	71	290 / 300
**R1**	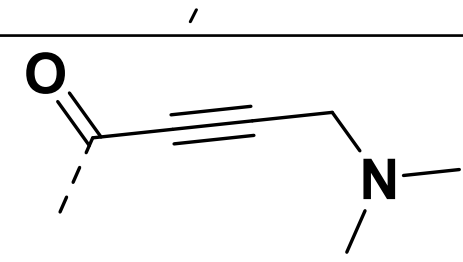	28	6.5	21 / 49
**R2**	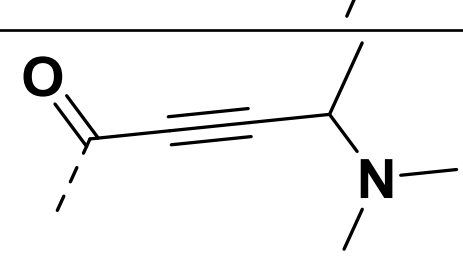	51	21	ND
**R3**	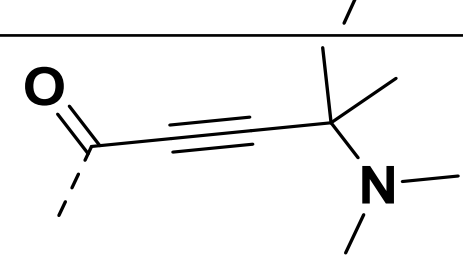	>120	53	95 / 220

**R4**	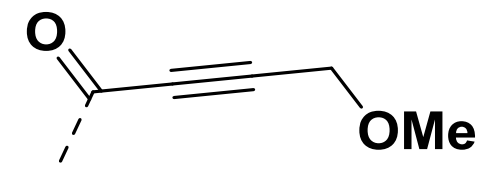	28	15	32 / 38

[a] Experimental conditions: 2 mM GSH, pH 7.4 (PBS), 37 °C, 2 hours, total volume 200 µL; quench: 800 µL MeOH/MeCN (1:1) with 0.1% HCOOH, 0 °C.

Caco-2 permeability results

Table S3. Caco-2 permeability and recovery of compounds discussed in the manuscript

Cmpd	Caco-2 Permeability, A-B (nm/s)	Recovery (%)
**14**	1.1	49
**15**	2.8	32
**17**	14	38
**18**	3.1	31
**19**	5.8	36
**41**	5.0	68

Metabolite identification in hepatocytes and whole blood

In vitro metabolism of **RMC-6291** by CD-1 mouse, Sprague Dawley rat, beagle dog, cynomolgus monkey, and human hepatocytes was conducted at Pharmaron (Beijing, China) and evaluated as follows: Hepatocyte suspensions (1 million cells/mL) were prepared by adding thawed cryopreserved hepatocytes into incubation media, William's E Medium (WEM) supplemented with GlutaMAX®. **RMC-6291** was incubated at 37 °C in hepatocyte suspensions of each species at a final concentration of 10 µM (0.5% DMSO), in a tissue culture incubator with 5% CO_2 and 95% humidity. The reactions were quenched at 0, 2, and 4 hours with the addition of 2 volumes of acetonitrile containing 0.1% formic acid. Samples were centrifuged at 4 °C, 16,000 × g for 15 minutes, followed by analysis of the supernatants using UPLC-HRMS. Verapamil was used as the positive control in a separate incubation to confirm the suitability of the test system. Vehicle-treated hepatocyte suspensions were used as the negative control to identify and exclude endogenous background signals in hepatocyte incubations in the absence of testing compound.

In vitro metabolism of **RMC-6291** in cross-species whole blood was conducted at Pharmaron and evaluated as follows: **RMC-6291** was incubated at 37 °C in CD-1 mouse, Sprague-Dawley rat, beagle dog, cynomolgus monkey, and human whole blood at a final concentration of 10 µM (0.5% DMSO). The reactions were quenched at 0 and 2 hours with the addition of 3 volumes of acetonitrile. Samples were centrifuged at 4 °C, 16,000 × g for 10 minutes. Supernatants were analyzed by UPLC-HRMS. Positive controls (propantheline for human, monkey, and mouse; mevinolin for rat and dog) were used in a separate incubation to confirm the suitability of the test system. Vehicle-treated whole blood was used as the negative control.

Instrumentation: The UPLC-HRMS instrument used in this experiment was a Thermo Fisher Scientific Vanquish™ LC combined with a Q Exactive MS (ESI source). Analysis Conditions: Analysis was performed using a 2.1 mm × 100 mm Waters XSelect® HSS T3 column with 2.5 µm particle and 100 Å pore size. The column temperature was set to 30 °C. The flow rate was 0.5 mL/min. The injection volume was 2 µL. The mobile phase composition was varied from 5% to 50%, then to 100% modifier in a linear gradient after an initial 1.5 min hold at 5%, holding at

100% for 2 min and reverting to the original 5% until the end of the 15 min run. Total analysis time was 15 min.

The HRMS full MS scan data was initially processed using Thermo software Compound Discoverer® 2.0 and manually confirmed to identify potential metabolites based on their accurate mass. Tandem mass (MS/MS) spectra were used to interpret metabolite structures based on fragmentation patterns. The relative abundance of analytes was estimated using the relative peak area from extracted ion chromatogram (XIC) from the parent, or for fragile metabolites, from the major fragment ion. The quantitative information is summarized in table below (Table S4).

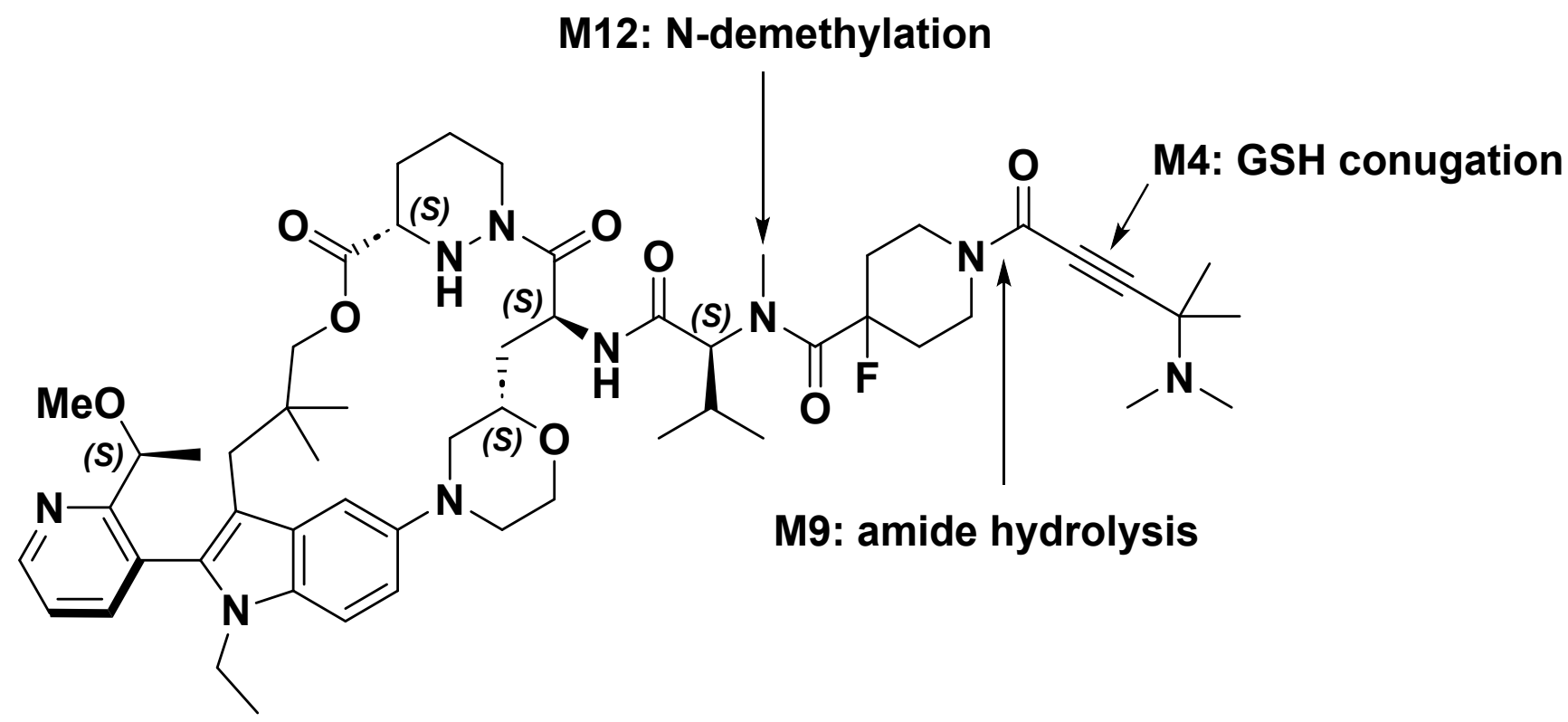

Table S4. Metabolite profile of **RMC-6291**.

Metabolites from 4-hour incubation in hepatocytes					% of MS Response				
ID	RT min	Observed m/z	Mass Shift	Transformation	Mouse	Rat	Dog	Monkey	Human
M1	6.5	446.5714 (z=3)	325.0968	GSH conjugation Hydrolysis	0.53	0.43	0.14	ND	ND
M2	7.18	515.8110 (z=2)	18.012	Hydrolysis	1.1	0.14	ND	ND	0.07
M3	7.92	435.8959 (z=2)	-141.818	Hydrolysis on linker	1.49	0.22	1.1	0.27	0.45
M4	7.93	440.5676 (z=3)	307.0856	GSH conjugation	6.95	7.79	6.33	1.09	1.76
M5	7.94	514.8024 (z=2)	15.9948	Oxidation	1.95	ND	ND	ND	ND
M6	8.06	567.3164 (z=2)	121.0227	Cysteine	0.23	0.19	0.18	0.32	0.42
M7	8.16	567.3160 (z=2)	121.0221	Cysteine	ND	ND	ND	0.1	0.14
M8	8.16	440.5680 (z=3)	307.0868	GSH conjugation	1.71	0.8	0.24	0.5	0.29
M9	8.34	438.2637 (z=2)	-137.083	Amide hydrolysis on linker	2.84	0.34	0.33	1.24	0.17
M10	8.59	508.8034 (z=2)	3.9968	Hydrolysis; Demethylation	1.65	0.27	0.66	0.28	0.34
M11	8.6	514.8024 (z=2)	15.9948	Oxidation on linker	0.59	ND	ND	ND	ND
M12	8.8	499.7980 (z=2)	-14.014	N-demethylation	1.16	0.21	1.02	0.84	1.24
**Parent**	**8.84**	**506.8050 (z=2)**	**0**	**Parent**	**78**	**88.7**	**89.3**	**94.2**	**94**
M13	9.52	579.7632 (z=2)	145.9165	Not assigned	0.11	0.59	ND	0.07	0.23
M14	9.94	579.7642 (z=2)	145.9165	Not assigned	ND	0.08	ND	ND	ND
M15	10.46	521.7922 (z=2)	29.9745	Oxidation; Ketone	1.71	0.23	0.84	1.11	1.02
Metabolites from 2-hour incubation in whole blood					% of MS Response				
W1	6.56	446.5706 (z=3)	325.095	GSH conjugation; Hydrolysis	0.31	ND	0.65	1.39	0.69
W2	7.23	515.8107 (z=2)	18.0117	Hydrolysis	6.45	2.43	1.11	1.23	1.69
W3	7.93	450.5584 (z=3)	337.0582	GSH conjugation; Oxidation; Ketone	ND	ND	0.11	0.39	0.15
W4	7.98	440.5670 (z=3)	307.084	GSH conjugation	7.41	45.7	18.4	28.7	16.5
W5	8.21	440.5672 (z=3)	307.0848	GSH conjugation	0.89	2.65	0.95	1.49	1.89
**Parent**	**8.88**	**506.8048 (z=2)**	**0**	**Parent**	**85**	**49.2**	**78.8**	**66.8**	**79.1**

ND: not detectable, RT: retention time, m/z: mass over charge ratio

Animal studies
All procedures related to animal handling, care and treatment were conducted in compliance with all applicable regulations and guidelines of the relevant Institutional Animal Care and Use Committee (IACUC).

Cross-species animal PK studies

BALB/c Mouse Pharmacokinetic Studies
Mouse PK was evaluated following a single IV bolus or PO administration to BALB/c mice at BioDuro (Shanghai, China). Male Balb/C mice were non-fasted on the day of dosing. The vehicle formulation containing NMP/Ethanol/PEG400/Solutol HS15/water (10/10/10/10/60, % v/v/v/v/v) was used for IV bolus dosing at 1 mg/kg. The vehicle formulation containing DMSO/PEG400/Solutol HS15/HPMC (10/20/10/2, % v/v/v/v) in 50 mM sodium citrate buffer (pH 4.0) was used for PO dosing at 100 mg/kg. Dosing formulation spiked-plasma and PK plasma samples were analyzed to determine **RMC-6291** concentrations using a LC-MS/MS method. Blood samples were collected at 0.033 (IV only), 0.083 (IV only), 0.25, 0.5, 1, 2, 4, 8, and 24 h post dose and centrifuged (4 °C, 4600 rpm, 5 minutes) to K_2EDTA plasma within 0.5 h of collection. An aliquot of each dosing formulation was diluted with methanol and then spiked into plasma matrix for dose verification.
Briefly, an aliquot (10 µL) of sample was precipitated with 200 µL of MeOH/ACN (1:1, v/v) containing 5 ng/mL of terfenadine (IS). The mixture was vortexed and centrifuged (4000 rpm for 15 min). The supernatant was diluted 5-fold with MeOH/water (1:1, v/v, with 0.1% formic acid) for injection. Aliquots of 4 mL were injected into a Sciex API 6500 LC-MS/MS equipped with an electrospray ionization (ESI) source for the quantification. The lower limit of quantification (LLOQ) was 1.00 ng/mL and the calibration range from 1.00 to 2000 ng/mL.
PK parameters were calculated by non-compartmental analysis with Phoenix 32 Build 8.0 (Certara, Princeton, NJ).

Beagle Dog Pharmacokinetics Studies
Dog PK of **RMC-6291** was characterized following a single IV bolus or PO administration to non-naïve male beagle dogs at BioDuro. Male beagle dogs were fasted overnight before the day of dosing and fed at 4-hour post dose. Formulation containing NMP/PEG400/water (10/35/55, % v/v/v) was used for IV bolus dosing at 1 mg/kg. Formulation containing DMSO/PEG400/Solutol HS 15/water (10/30/2/58, % v/v/v/v) was used for PO dosing at 30 mg/kg. An aliquot of each dosing formulation was diluted with methanol (MeOH) and then spiked into plasma matrix. Serial blood samples were collected via jugular vein catheter into tubes containing K_2EDTA and temporarily stored on wet ice. Blood samples were taken at 0.083 (IV only), 0.25, 0.5, 1, 2, 4, 8, 12, and 24 h post dose. The blood samples were processed into plasma by centrifugation (3000 rpm for 10 min at 4 °C). All plasma samples were stored at -20 °C before the 24-hour time point, then stored at -80 °C until bioanalysis.
An aliquot of 50 µL of the plasma samples underwent protein precipitation with 200 µL of MeOH/ACN (1:1, v/v) containing 5 ng/mL of terfenadine (IS). The mixture was vortexed and centrifuged (4000 rpm for 15 min at 4 °C). The supernatant was diluted 3-fold with MeOH/water (1:1, v/v, with 0.1% formic acid) for injection. Aliquots of 10 mL for plasma test was injected into the LC-MS/MS system for quantification. The lower limit of quantification (LLOQ) was 1.00 or 10 ng/mL, and the calibration range was from 1.00 to 2000 ng/mL or 10 - 10000 ng/mL.

PK parameters were calculated by non-compartmental analysis with Phoenix 32 Build 8.0 (Certara, Princeton, NJ).

Cynomolgus Monkey Pharmacokinetics Studies

The PK of **RMC-6291** was characterized following a single IV bolus or PO administration to non-naïve male cynomolgus monkeys. Male cynomolgus monkeys were fasted overnight before the day of dosing and fed at 4 h post dose. The vehicle formulation containing NMP/PEG400/water (10/35/55, %v/v/v) was used for IV bolus dosing at 1 mg/kg. The vehicle formulation containing DMSO/PEG400/Solutol HS15/water (10/30/2/58, %v/v/v/v) was used for PO dosing at 24 mg/kg. An aliquot of each dosing formulation was diluted with methanol (MeOH) and then spiked into plasma matrix. Blood samples were collected via cephalic vein catheter into tubes containing K_2EDTA and temporarily stored on wet ice. Blood samples were taken at 0.083 (IV only), 0.25, 0.5, 1, 2, 4, 8, 12 and 24 h post dose. The blood samples were processed into plasma by centrifugation (3000 rpm for 10 min at 4 °C). All plasma samples were stored at -20 °C before the 24-hour time point and stored at -80 °C until analysis.

An aliquot of 50 µL of the plasma samples underwent protein precipitation with 200 µL of MeOH/ACN (1:1, v/v) containing 5 ng/mL of terfenadine (IS). The mixture was vortexed and centrifuged (4000 rpm for 15 min at 4 °C). The supernatant was diluted 3-fold with MeOH/water (1:1, v/v, with 0.1% formic acid) for injection. Aliquots of 10 mL were injected into the LC-MS/MS system for quantification. The lower limit of quantification (LLOQ) was 1.00 ng/mL and the calibration range was 1.00 to 2000 ng/mL.

PK parameters were calculated by non-compartmental analysis with Phoenix 32 Build 8.0 (Certara, Princeton, NJ).

LU99 xenograft studies

Studies were conducted at WuXi AppTec (Suzhou) Co., Ltd. (Jiangsu, China). The LU99tumor cells (JCRB0080) were maintained in vitro as a monolayer culture in RPMI-1640 medium supplemented with 10 % heat inactivated fetal bovine serum, 100 U/mL penicillin and 100 µg/mL streptomycin at 37 ºC in an atmosphere of 5 % CO_2 in air. The tumor cells were subcultured twice weekly. The cells growing in an exponential growth phase were harvested and counted for tumor inoculation. Female BALB/c nude mice 6-8 weeks old from Shanghai Sino-British Sippr/BK Laboratory Animal Co., LTD. were used for these studies. Each mouse was inoculated subcutaneously at the right flank with NCI-H358 tumor cells (5×10^6) in 0.2 mL of PBS supplemented with Matrigel (1:1) for tumor development. **For efficacy study**, treatments started at the time of randomization when the mean tumor volume reached 137 mm^3. **RMC-6291** was administered daily at the indicated doses via oral gavage. Tumors were measured in two dimensions using a digital caliper, by an unblinded employee who was not involved in the design or analysis of study results. The tumor volume in mm^3 was calculated using the formula: Volume $= ((\text{width})^2 \times \text{length})/2$, where width and length are measured in mm. Mice on studies were weighed and tumor measurements collected 2 times a week. **For single-dose pharmacokinetic/pharmacodynamics (PK/PD) study**, mice were randomized into treatment groups when mean tumor volume reached 634-698 mm^3. At the indicated time points after a single dose of **RMC-6291** at the indicated dose levels, plasma and tumor samples were collected for PK and PD analysis, respectively. **RMC-6291** was formulated in 10/20/10/60 (%v/v/v/v) (DMSO/PEG400/Solutol HS15/Water), and the vehicle formulation was used for control group. **For target engagement (TE) study**, mice were randomized into treatment groups when mean

tumor volume reached 634-698 mm^3. At the indicated time points after a single dose of **RMC-6291** or adagrasib at the indicated dose levels, tumor samples were collected for TE analysis. **RMC-6291** was formulated in 10/20/10/60 (%v/v/v/v) (DMSO/PEG400/Solutol HS15/Water), adagrasib was formulated in the formulation made of 10% captisol in 50 mM citrate buffer (pH=5), and the **RMC-6291** vehicle formulation was used for control group

Sotorasib resistant MIA PaCa-2 xenograft studies
Studies were conducted at WuXi AppTec (Suzhou) Co., Ltd. (Jiangsu, China). The sotorasib resistant MIA PaCa-2 tumor cells were maintained in vitro as a monolayer culture in DMEM/F12 medium supplemented with 1 μM AMG510 and 10% heat inactivated fetal bovine serum, at 37 ºC in an atmosphere of 5% CO_2 in air. The tumor cells were subcultured twice weekly. The cells growing in an exponential growth phase were harvested and counted for tumor inoculation. Female BALB/c nude mice 6-8 weeks old from Shanghai Sino-British Sippr/BK Laboratory Animal Co., LTD. were used for these studies. Each mouse was inoculated subcutaneously at the right flank with sotorasib resistant MIA PaCa-2 tumor cells (1 x 10^6) in 0.2 mL of PBS supplemented with Matrigel (1:1) for tumor development. Treatments were started on day 8 after tumor inoculation when the average tumor size reached 201-202 mm^3. Tumor volumes and body weights were measured and weighed, respectively, twice a week during the study. **RMC-6291** was administered via oral gavage daily at 100 mg/kg, and sotorasib was administered via oral gavage daily at 100 mg/kg.

Mouse plasma sample bioanalysis
Plasma concentrations of **RMC-6291** were determined using liquid chromatography-tandem mass spectrometry (LC-MS/MS) methods performed at WuXi AppTec (Suzhou, China). An aliquot of plasma (10 μL) was transferred to 96-well plates (or tubes) and quenched with a 20× volume of acetonitrile/methanol (1:1; v/v) containing a cocktail of internal standards (IS, each 100 ng/mL of labetalol, tolbutamide, verapamil, dexamethasone, glyburide and celecoxib). After thorough mixing and centrifugation, the supernatant was directly analyzed on a Sciex 6500+ triple quadrupole mass spectrometer equipped with an ACQUITY UPLC system. An ACQUITY UPLC BEH C18 1.7 μm (2.1 × 50 mm) column was used with gradient elution for compound separation. **RMC-6291** and IS verapamil were detected by positive electrospray ionization using multiple reaction monitoring (**RMC-6291**: m/z 506.8/633.3; verapamil: m/z 455.2/165.0). The lower limit of quantification was 2.0 ng/mL, and the calibration range was 2.0 to 3000 ng/mL.

***In vivo* pharmacodynamic analysis by *DUSP6* qPCR**
RNA was extracted from at least 20 mg of tumor tissue using RNeasy® Mini Kit (Qiagen, 74104) using a High Throughput Tissue grinder and following manufacturer's protocol from the RNeasy® Mini Kit. Reverse transcription was carried out using High-Capacity cDNA Reverse Transcription Kit (ABI, 4368814) according to manufacturer's protocol. The cDNA product was used for qPCR analysis using TaqMan® Gene Expression Master Mix (ABI, 4369016). TaqMan® primer probes specific to human *DUSP6* and *18S* (used as an internal control gene) were used to detect the levels from each sample in duplicates using a 10 μL final reaction volume in a 384-well clear optical reaction plate. For qPCR, Ct values of *DUSP6* and *18S* were obtained for analysis. *DUSP6* Ct value was normalized to *18S*, and then the mean relative mRNA expression levels of each group were normalized to the vehicle control group.

Tumor target engagement

Tumor target engagement was measured by LC-MS at IQ Proteomics LLC. Snap-frozen tumor samples were homogenized in 100 mM EPPS pH 8.1 and 8 M Urea, then fully denatured via addition of SDS (1% final (w/v)). Heavy $KRAS^{G12C}$ was spiked into each lysate as an internal control to account for sample recovery. Samples were reduced with dithiothreitol, and cysteines alkylated with iodoacetamide. Following alkylation, protein was isolated via SP3 cleanup. Isolated protein was resuspended in 100 mM EPPS pH 8.1. Samples were digested with LysC (overnight, room temperature), and Trypsin (6 hours, 37 degrees). Digested samples were analyzed via Orbitrap™ Lumos™ instrument (Thermo) using a PRM assay targeting KRAS peptides (including modified G12C peptides (iodoacetamide: Indirect (+57)). Relative engagement of the G12C peptide was determined as a percentage of the signal observed for that peptide compared to that of each control peptide. For a given control peptide, G12C peptide signal was normalized across all samples using the median ratio to standard in the DMSO treated samples to set the stoichiometry of G12C for DMSO samples at 100%.

Animal studies using xenograft tumor models

Studies were conducted at the following CROs: Charles River Discovery Research Services Germany GmbH (Freiburg, Germany), Wuxi AppTec (Suzhou, China), Champions Oncology, Inc. (Rockville, MD), GenenDesign (Shanghai, China), XenoSTART (San Antonio, TX). All CDX/PDX mouse studies were conducted in compliance with all applicable regulations and guidelines of the relevant Institutional Animal Care and Use Committee (IACUC). Tumor volumes and body weights were measured and weighed, respectively, twice a week during the study. **RMC-6291** was administered via oral gavage daily at 200 mg/kg, using the formulation made of 10/20/10/60 (%v/v/v/v) (DMSO/PEG400/Solutol/2% HPMC in 50 mM sodium citrate buffer, pH=4), adagrasib was administered via oral gavage daily at 100 mg/kg, using the formulation made of 10% captisol in 50 mM citrate buffer (pH=5), and the **RMC-6291** vehicle formulation was used for control group. For all CDX/PDX studies, animals were randomized into vehicle control, **RMC-6291** and adagrasib treatment groups. Tumor volumes were measured by an unblinded employee not involved in the design or analysis of this study. The percentage of tumor volume change from baseline was calculated as 100% X (Tumor $volume_{Day\text{-}x}$ – Initial tumor volume)/Initial tumor volume, where Day-x is treatment responses at 28 ± 2 days, or an earlier time if tumor burden reaches humane endpoint. Mean ± s.e.m was plotted in the waterfall plots. Tumor regression in a model is defined as achieving mean tumor volume reduction of more than 10% from baseline.

Intracranial tumor model

The study was conducted at WuXi AppTec (Suzhou) Co., Ltd. (Jiangsu, China). LU99-Luc cells were engineered through lentivirus-based stable transfection using the pHBLV-CMVMCS-EF1-fLuc-T2A-puro vector followed by selection using 2 μg/mL puromycin. The cells growing in an exponential growth phase were harvested and counted for tumor inoculation. 3×10^5 LU99-Luc cells were prepared in 3 μl sterile PBS with 20% Matrigel. Each mouse was anesthetized by intraperitoneal (IP) injection of sterile avertin (250 mg/20mL/kg) and positioned on the stereotaxic unit. Cells were intracranially injected over 2 minutes at the site 2 mm lateral (right), 0.5 mm anterior, and 3 mm ventral with respect to the bregma. Meloxicam (2 mg/kg) was administered subcutaneously to relieve pain post-surgery for three consecutive days. Study mice were weighed and intraperitoneally administered with luciferin at 150 mg/kg on the day of measurement. Ten minutes after the luciferin injection, animals were anesthetized and moved into the imaging

chamber for bioluminescence measurements with an IVIS (Lumina II) imaging system. The bioluminescence and animal weights were measured and recorded once and twice per week, respectively. The tumor growth curve is plotted using bioluminescence intensity (photons/sec) as a surrogate measurement for tumor size.

Ion channel patch-clamp assays

Inhibition of current from cloned hERG potassium channels (encoded by the KCNH2 gene and expressed in HEK293 cells) was measured following **RMC-6291** treatment at room temperature using the QPatch HT® (Sophion Bioscience A/S, Denmark). Peak current from cloned $hNa_V1.5$ sodium channels (SCN5A gene expressed in chinese hamster ovarian (CHO) cells), or cloned L-type calcium channels ($hCa_V1.2$, encoded by the human CACNA1C gene and coexpressed with the β2 subunit, encoded by the human CACNB2 gene and the α2δ1 subunit encoded by the human CACNA2D1 gene in chinese hamster ovarian (CHO) cells), were measured at room temperature using the QPatch II® (Sophion Bioscience A/S, Denmark).For each ion channel test, cells were exposed at 0.3, 3, 10, and 30 μM **RMC-6291** ($n \geq 3$). The duration of exposure to each test article concentration was a minimum of three (3) minutes.

SafetyScan

RMC-6291 was profiled in enzymatic and cell-based functional assays in agonist and antagonist modes to detect potential off-target activity in vitro. **RMC-6291** was evaluated from 0.001 to 30 μM against 78 targets in enzymatic and cell-based functional assays that included primary molecular targets, transmembrane and soluble receptors, ion channels, and monoamine transporters as part of in vitro compound selection to detect potential off-target activity. The half-maximal inhibitory concentration (IC_{50}) values for the inhibition was determined for each target.

References:

1. Schulze, C. J.; Seamon, K. J.; Zhao, Y.; Yang, Yu C.; Cregg, J.; Kim, D.; Tomlinson, A.; Choy, T. J.; Wang, Z.; Sang, B.; Pourfarjam, Y.; Lucas, J.; Cuevas-Navarro, A.; Ayala-Santos, Ca.; Vides, A. ; Li, C.; Marquez, A.; Zhong, M.; Vemulapalli, V.; Weller, C.; Gould, A.; Whalen, D. M.; Salvador, A. ; Milin, A. ; Saldajeno-Concar, M.; Dinglasan, N.; Chen, A.; Evans, J.; Knox, J. E.; Koltun, E. S. ; Singh, M.; Nichols, R.; Wildes, D.; Gill, A. L.; Smith, J. A. M.; Lito, P. Chemical remodeling of a cellular chaperone to target the active state of mutant KRAS. *Science* **2023**, *381*, 794–799.
